# Supplementary material for: Multi-omics analyses reveal that the gut microbiome and its metabolites promote milk fat synthesis in Zhongdian yak cows
Source: PeerJ. 2022 Dec 2;10:e14444. doi: 10.7717/peerj.14444 (PMC9744170; doi:10.7717/peerj.14444)
Supplement: Supplemental Information 13 [file peerj-10-14444-s013.zip › Web_Report/HTML/metabolites_exp.xls.html]

metabolites\_exp.xls


## metabolites\_exp.xls

| ID | name | H1 | H2 | H3 | H4 | H5 | H6 | L2 | L6 | L1 | L4 | L5 | L3 |
| --- | --- | --- | --- | --- | --- | --- | --- | --- | --- | --- | --- | --- | --- |
| pos\_1 | D-erythro-Dihydrosphingosine | 0.000143373613404701 | 0.000280516099889959 | 0.000224718909490409 | 0.000254417617725337 | 0.00016750424081482 | 0.000200086248543327 | 0.000139869306485217 | 0.000141074891906182 | 0.000130812981686961 | 0.000127429588182838 | 0.000157863228276111 | 0.000124289239886146 |
| pos\_10 | Deoxycholic acid | 0.0271025015361995 | 0.022745320979762 | 0.0670788851770679 | 0.0597087546104036 | 0.0754371883493432 | 0.0578048511713294 | 0.0490250201416075 | 0.0440506703924097 | 0.0684691105283223 | 0.0536639780874919 | 0.0570077802127478 | 0.0520879671705176 |
| pos\_100 | Dihydroscoparin | 3.39668072962394e-05 | 4.77892415245145e-05 | 7.52120583590671e-05 | 0.000224025674881867 | 8.73793923402068e-05 | 2.03705463933448e-05 | 4.8823197771671e-05 | 3.2263567000144e-05 | 6.93457125590401e-05 | 4.25746516619048e-05 | 0.000130961626000574 | 9.17680508017233e-05 |
| pos\_1000 | Lansioside C | 0.000923806234985915 | 0.000463087908401238 | 0.000410119036086324 | 0.00040159160998322 | 0.000476206062915908 | 0.000493218711631254 | 0.000509510990697741 | 0.000487006349781475 | 0.000460741887322162 | 0.000435062366240488 | 0.000467326727086756 | 0.000474187485748591 |
| pos\_1001 | Hexylamine | 4.01384358402482e-12 | 3.9576159497502e-12 | 6.57739001429416e-07 | 0 | 0 | 0 | 2.40099525812225e-06 | 2.22730488235693e-06 | 0 | 4.64508343563833e-09 | 1.20430046582174e-06 | 0.000485108730413289 |
| pos\_1002 | Ginsenoyne I | 4.53350425687984e-06 | 9.75242210291292e-06 | 8.47109894285107e-06 | 1.02142773637819e-05 | 1.21264494948e-05 | 9.52843959345179e-06 | 8.35859896511495e-06 | 9.45917829860012e-06 | 1.02390284793562e-05 | 9.27516222460088e-06 | 7.36279430579978e-06 | 1.0737592159788e-05 |
| pos\_1003 | Testolactone | 0.00026366640565466 | 0.000202893278554169 | 0.000180401894400444 | 0.000173868741781195 | 0.000181821552691564 | 0.000196281611761064 | 0.000208573667764698 | 0.000196480787650124 | 0.00020561505916938 | 0.000182802184655176 | 0.000190343364900372 | 0.000193710422490535 |
| pos\_1004 | DG(20:4(5Z,8Z,11Z,14Z)/22:5(4Z,7Z,10Z,13Z,16Z)/0:0) | 0.000327490794697234 | 0.000315296315648543 | 0.000403287167075199 | 0.000205049280907728 | 0.000181584705302854 | 0.000259374678213042 | 0.00016032175080653 | 0.000265785271783621 | 0.00039475618961812 | 0.000193419478373732 | 0.000285777252556653 | 0.000411026873674963 |
| pos\_1005 | Bovinic acid | 7.27384813662508e-05 | 5.06632478351181e-05 | 4.66893124317539e-05 | 4.74674269697067e-05 | 3.34830463533819e-05 | 3.23635637828467e-05 | 3.76905549919153e-05 | 4.30996677093678e-05 | 3.10740657141884e-05 | 3.51713540738406e-05 | 3.54024016204916e-05 | 3.0586890091983e-05 |
| pos\_1006 | (S)-2-Azetidinecarboxylic acid | 1.72745918968914e-05 | 2.47177461398509e-05 | 2.27768189119798e-05 | 1.84247640538446e-05 | 1.53026882583098e-05 | 1.85653416621923e-05 | 1.68719669039963e-05 | 1.80124418692465e-05 | 2.20337767924986e-05 | 1.67980938319025e-05 | 1.85996129911411e-05 | 1.57316370359164e-05 |
| pos\_1007 | PE(14:0/22:4(7Z,10Z,13Z,16Z)) | 0.000196979699388231 | 0.000200310234098613 | 0.000183365212790727 | 0.000165943433760962 | 0.000158517170678757 | 0.000190478596602469 | 0.000176210202172162 | 0.000180250520336071 | 0.000189487228029412 | 0.000175811633231088 | 0.000183338783203375 | 0.00017458366169039 |
| pos\_1008 | Leonoside B | 0.000142653956932748 | 8.72088866303195e-06 | 1.04118572430884e-05 | 9.10522179364048e-06 | 6.12985367675102e-05 | 5.08121153938634e-05 | 4.37900442346611e-05 | 2.23778429359536e-05 | 1.40803454408975e-05 | 2.79843707502444e-05 | 3.04005617349897e-05 | 2.04188469745136e-05 |
| pos\_1009 | Desvenlafaxine | 0.000130567132300891 | 6.53539738190708e-05 | 5.42750682991678e-05 | 5.12001162243997e-05 | 8.59789527535932e-05 | 8.35458509062857e-05 | 8.35168750835916e-05 | 7.33718251360655e-05 | 6.81885946387154e-05 | 6.42461891698447e-05 | 7.24074750376975e-05 | 6.30756837493085e-05 |
| pos\_101 | 8Z,11Z,14Z-heptadecatrienal | 0.000218844998153951 | 0.000283005428505909 | 0.000241268861184055 | 0.000259448116643435 | 0.000196857544018961 | 0.000235037786833169 | 0.000222024530905838 | 0.000227157492014482 | 0.000254989501805454 | 0.000237195932263134 | 0.000235600251803542 | 0.00023038271458957 |
| pos\_1011 | 2-hydroxymexiletine | 1.02634560807322e-05 | 1.56398481226908e-05 | 9.36898363307684e-06 | 1.24012646877024e-05 | 1.74441928975579e-05 | 1.17357847045627e-05 | 1.18026347276937e-05 | 1.11112141878373e-05 | 1.29025062641266e-05 | 1.32517570750664e-05 | 1.07573074468865e-05 | 1.34581563554048e-05 |
| pos\_1012 | Isoleucyl-Valine | 0.000129268442310593 | 0.00014164509497671 | 0.000101915934954295 | 0.000105425743673157 | 0.00022160111000702 | 0.000116664968544212 | 0.000166848524208239 | 0.000153487108197949 | 0.000178843486427081 | 0.000132189139822453 | 0.000142588787132708 | 0.000143473454137317 |
| pos\_1013 | Milrinone | 0.00023957760883478 | 0.000336998962200162 | 0.000224990212454456 | 0.000535031412238406 | 0.000300744223137582 | 0.000332302368057264 | 0.000187323065049999 | 0.000263334244137771 | 0.000622073540974261 | 0.000373149085909829 | 0.00036937799320492 | 0.000353661978446681 |
| pos\_1014 | MG(0:0/24:6(6Z,9Z,12Z,15Z,18Z,21Z)/0:0) | 4.37299840012898e-06 | 1.12810205371654e-06 | 3.07986457919072e-06 | 1.84034817413664e-06 | 1.46172732824887e-07 | 0 | 2.04544548982886e-06 | 0 | 3.15696813997665e-06 | 0 | 8.62691129430312e-07 | 1.63600205982887e-06 |
| pos\_1015 | 1,5,5,8-Tetramethyl-12-thiabicyclo[9.1.0]dodeca-3,7-diene | 6.60150270610574e-05 | 8.05127870043297e-05 | 0.000114183785426355 | 5.55116393512457e-05 | 6.81076963838517e-05 | 7.32266006934514e-05 | 4.87484712011346e-05 | 6.94523374467758e-05 | 5.94118478793743e-05 | 0.000117603899395477 | 7.8047810888355e-05 | 9.21345724874752e-05 |
| pos\_1016 | Monobenzone | 1.70970709138999e-06 | 1.17544913988774e-06 | 3.00076340529876e-06 | 6.8328783204821e-07 | 2.10228488865334e-06 | 1.38518669456126e-06 | 4.56565857708574e-07 | 6.86109385224535e-07 | 5.26378196677087e-07 | 5.94116154509174e-06 | 1.70142465147202e-06 | 2.46511636008939e-06 |
| pos\_1017 | PE(15:0/18:4(6Z,9Z,12Z,15Z)) | 0.000197631208990756 | 7.91304040780834e-05 | 0.000212285799860619 | 8.33473322646422e-05 | 7.70840802711044e-05 | 9.56980163979704e-05 | 8.39818671919653e-05 | 9.53021572679631e-05 | 7.5459557798376e-05 | 0.000130972154679974 | 9.46887785304565e-05 | 0.000115368586481174 |
| pos\_1018 | PC(22:2(13Z,16Z)/22:5(7Z,10Z,13Z,16Z,19Z)) | 0.00011567053295177 | 7.20058131103287e-05 | 0.000100220916383672 | 7.37978102172504e-05 | 7.61088800513174e-05 | 7.99836207724934e-05 | 9.70593884024652e-05 | 9.76318945493499e-05 | 7.5373485159326e-05 | 9.85552802862361e-05 | 8.43031729508929e-05 | 9.70642213696361e-05 |
| pos\_1019 | 20-Hydroxy-leukotriene B4 | 0.000147809485138006 | 0.000248899361063524 | 0.000180268402874634 | 0.000169850298601026 | 0.000177614021224948 | 0.000159383913203389 | 0.00017261692787171 | 0.000167745855211777 | 0.000206922018130803 | 0.000153561557838801 | 0.000140557428464811 | 0.000266592041189631 |
| pos\_102 | 12-HYDROXY-4,4-BISNOR-4,8,11,13-PODOCARPATETRAEN-3-ONE | 1.45481436537097e-05 | 1.82179286117428e-05 | 9.8759297569512e-05 | 0.000297473347623487 | 2.07959488898238e-05 | 5.43385413474046e-05 | 2.16957315160989e-05 | 9.58601595748861e-05 | 1.27949870047946e-05 | 1.76429735909797e-05 | 6.04980849726973e-05 | 0.000248155857441198 |
| pos\_1020 | Nitrendipine | 0.000130515254435659 | 0.000136576528429339 | 9.86868794673674e-05 | 0.000124345562049056 | 0.00013974574587356 | 0.00013000532466692 | 9.67006130125323e-05 | 0.000102652883857016 | 0.000151865360049453 | 0.000119878110085546 | 0.000124409428177317 | 0.000118990999471144 |
| pos\_1021 | (gamma-Glutamyl-gamma-glutamyl)-S-methylcysteine | 1.53826395984939e-08 | 7.19218356952668e-07 | 8.60569223439073e-07 | 4.53994841015798e-08 | 0 | 0 | 1.01712286311919e-06 | 1.71049665724877e-05 | 4.31136470232878e-08 | 8.60230561575565e-08 | 2.48656980276972e-06 | 1.21621895831902e-05 |
| pos\_1022 | Nicotinate D-ribonucleoside | 7.88952842929272e-06 | 8.49016026475634e-06 | 6.5950092328667e-06 | 9.13162637975416e-06 | 4.12888314561963e-06 | 6.01474248247494e-06 | 5.6163545938856e-06 | 7.05735831314195e-06 | 5.58338101111197e-06 | 7.36721135427609e-06 | 8.4263373459177e-06 | 4.58247731302156e-06 |
| pos\_1023 | Cyclotricuspidogenin C | 0.00188466527979725 | 0.00467659207960052 | 0.00213749646637568 | 0.00196588754562336 | 0.00367339361712428 | 0.00283756381021971 | 0.00197145856971725 | 0.00215727760089143 | 0.00236979627014512 | 0.00325414620279952 | 0.00251666725247531 | 0.00197994318704191 |
| pos\_1024 | Glabric acid | 7.95408355230361e-05 | 0.000136287085239189 | 0.000227185083441511 | 0.00014486077212907 | 0.000112209392958009 | 0.000132446524187877 | 0.000117745978646472 | 0.00019541351393701 | 9.39244518671275e-05 | 8.65885816035481e-05 | 0.000156795406691767 | 0.000912863528452739 |
| pos\_1025 | Deoxyadenosine | 3.31712095827085e-05 | 4.24179239581275e-05 | 1.38459947583294e-05 | 3.37056786855578e-05 | 2.9779773344249e-05 | 2.05954725566098e-05 | 3.70986910070258e-05 | 2.35992230274572e-05 | 2.20521897609213e-05 | 5.35145271190117e-05 | 2.5964880660197e-05 | 2.08005372583303e-05 |
| pos\_1026 | DG(18:3(6Z,9Z,12Z)/20:3(5Z,8Z,11Z)/0:0) | 1.26496819423674e-05 | 1.15753400944933e-05 | 7.29505368242742e-06 | 2.62656852047981e-06 | 3.19647613513613e-06 | 3.07482324843295e-06 | 0 | 0 | 4.76423958899552e-06 | 5.73888393949322e-06 | 5.65855175137993e-06 | 0 |
| pos\_1027 | 2-Oxoarginine | 6.37607035005415e-06 | 6.3760944522566e-06 | 1.55219252327998e-05 | 1.35472748766896e-05 | 7.50157362108919e-06 | 1.0896199223601e-05 | 2.7518925262541e-06 | 1.56624226149819e-05 | 2.51956827626205e-06 | 1.54813399589345e-05 | 1.12406700387779e-05 | 2.56215701831606e-05 |
| pos\_1028 | 2-(Methoxycarbonyl)-5-methyl-2,4-bis(3-methyl-2-butenyl)-6-(2-methyl-1-oxopropyl)-5-(4-methyl-3-pentenyl)cyclohexanone | 0.000335925392619886 | 0.000491701782245311 | 0.000374344931054307 | 0.00035607055681734 | 0.000263913325159958 | 0.000387462016191042 | 0.000398309458896361 | 0.000402576667104042 | 0.000422899241487336 | 0.000297541943475914 | 0.0004149926375508 | 0.000301941756381162 |
| pos\_1029 | 9-Hydroxy-10-O-D-glucuronoside-12Z-octadecenoate | 0.00170900231310066 | 0.000917489045291512 | 0.000883812339359156 | 0.000781236859343664 | 0.000973241299536761 | 0.00102815469571858 | 0.00107903433341381 | 0.000979933634195125 | 0.00100207129344455 | 0.000866043233923529 | 0.000960731683482896 | 0.000951781571227786 |
| pos\_103 | Arg Trp Leu His | 0.000253641287489722 | 0.000627454603311231 | 0.000182075805204227 | 0.000236711438357549 | 0.000133778343427407 | 0.000299137794318512 | 0.000451983366397605 | 0.000205518026770613 | 0.000184075878059742 | 0.000222142348487212 | 0.000361691712134722 | 0.000135796396338343 |
| pos\_1030 | 2-Hydroxymyristic acid | 1.50006194693166e-05 | 2.500343685475e-05 | 2.65159635352961e-05 | 2.11871145050238e-05 | 1.80390130283816e-05 | 2.21685974572231e-05 | 2.77348794845154e-05 | 2.70127601652787e-05 | 2.35002469005625e-05 | 2.1293966480851e-05 | 1.90571567842604e-05 | 2.0473473916051e-05 |
| pos\_1032 | Goyaglycoside c | 0.00524062172752608 | 0.00594276153487909 | 0.00550554738381607 | 0.00553578266534895 | 0.00443961360598091 | 0.00503802144734157 | 0.00447256771631958 | 0.00500483915482376 | 0.00581930663581306 | 0.00471871350224968 | 0.00507980218488596 | 0.00470490906251234 |
| pos\_1033 | Xanthomicrol | 1.79760620797199e-05 | 2.57784588767777e-05 | 8.79106904868518e-06 | 1.43953389558529e-05 | 2.01152618894345e-05 | 1.6737677783045e-05 | 1.48698217802675e-05 | 1.38021768939549e-05 | 4.30252896156245e-05 | 2.1526083522143e-05 | 2.46666195805544e-05 | 2.02892492084095e-05 |
| pos\_1034 | Annoglaxin | 0.00089693260787426 | 0.00125388733205447 | 0.000678267918230677 | 0.00164604650860901 | 0.000735088087412063 | 0.0010463553227684 | 0.0011729876330823 | 0.000893781010032995 | 0.000715083172958179 | 0.00103939153409306 | 0.00088023195271656 | 0.000695585388653633 |
| pos\_1035 | Methoxybrassinin | 3.29815537642633e-05 | 1.42698449329147e-05 | 1.58153663943261e-05 | 2.00484653632003e-05 | 1.94747890727391e-05 | 2.05420892883088e-05 | 1.93886947255518e-05 | 1.88863075543529e-05 | 2.33390471447748e-05 | 2.01661658879195e-05 | 2.04420209543451e-05 | 2.05203808470549e-05 |
| pos\_1036 | Amdinocillin | 4.04685467791073e-05 | 3.31040638736945e-05 | 1.04396121130422e-05 | 2.75704117629626e-05 | 2.94162813958151e-05 | 2.46818120341552e-05 | 1.98238493376738e-05 | 1.38018582876664e-05 | 1.92815349281629e-05 | 3.72522925387449e-05 | 2.12441891596672e-05 | 1.46318229130821e-05 |
| pos\_1037 | (1(10)E,4E,6a,9b)-9-(2-Methylbutanoyloxy)-1(10),4,11(13)-germacratrien-12,6-olide | 6.11900843656508e-05 | 7.11896943135269e-05 | 1.87811708489621e-05 | 2.60670889239992e-05 | 3.24662978750877e-05 | 4.07943492282159e-05 | 3.16565850810706e-05 | 2.42514713152644e-05 | 1.90325913036559e-05 | 3.00379859576685e-05 | 2.41745936623587e-05 | 1.68941038980148e-05 |
| pos\_1038 | Tacrolimus | 0.000270981688950648 | 8.45945156076044e-05 | 7.4616203637102e-05 | 6.45977245453701e-05 | 0.000158932039942592 | 0.000159941548848687 | 0.000147291553558647 | 0.00012309667651938 | 9.83127705511557e-05 | 0.000110439929644604 | 0.000119619409770769 | 0.000109928612167097 |
| pos\_104 | Cyperotundone | 0.000275545001922607 | 0.000297481899373465 | 0.000289622048872899 | 0.000274233222757969 | 0.000241125578671086 | 0.000264612452363184 | 0.000252826717742442 | 0.000272586432439615 | 0.000251636125132692 | 0.000281984393654512 | 0.00026428928610927 | 0.000275326482903984 |
| pos\_1040 | Soyasapogenol B 3-O-b-D-glucuronide | 7.83874230516209e-06 | 1.32057949160496e-05 | 6.49935365262856e-06 | 1.27859033210459e-05 | 1.08304882609944e-05 | 1.40586535633724e-05 | 1.09252006312268e-05 | 1.09920550458089e-05 | 9.93422330866367e-06 | 1.25904643474031e-05 | 1.49470807513284e-05 | 9.4973341927714e-06 |
| pos\_1041 | TG(22:0/14:1(9Z)/20:5(5Z,8Z,11Z,14Z,17Z)) | 3.05907624428032e-05 | 3.39704411351982e-05 | 0.000119758349790108 | 8.37984759495923e-05 | 4.15285105715499e-05 | 6.2424667479201e-05 | 0.000138254864072635 | 0.000107731696437159 | 2.33067561681828e-05 | 7.16130463190421e-05 | 8.54059145037519e-05 | 6.47272241650251e-05 |
| pos\_1042 | E,e-Carotene-3,3'-dione | 2.38073706366802e-05 | 3.15346324626208e-05 | 1.90178501652335e-05 | 3.51624289122003e-05 | 2.87970028442047e-05 | 3.53810185779563e-05 | 3.48927632532693e-05 | 3.19153538720688e-05 | 3.1830993998298e-05 | 2.6364737581942e-05 | 3.51468647677599e-05 | 2.47076748620908e-05 |
| pos\_1043 | L-2-Amino-3-methylenehexanoic acid | 1.49528463334129e-05 | 1.85454321130782e-05 | 1.83700704777963e-05 | 2.11531297895208e-05 | 1.56724643899687e-05 | 1.6882398136324e-05 | 1.79207832897366e-05 | 1.82507549532422e-05 | 1.75349572122101e-05 | 1.94224810346297e-05 | 1.72505345870155e-05 | 1.97517120085228e-05 |
| pos\_1044 | L-Leucine | 1.16750803603863e-05 | 1.74041849493857e-05 | 1.58470883845763e-05 | 1.55457499249035e-05 | 1.43621862859223e-05 | 1.49516045430557e-05 | 1.25936573676898e-05 | 1.34719762888928e-05 | 1.77897620811349e-05 | 1.51711960971822e-05 | 1.62705380741971e-05 | 1.71135078766226e-05 |
| pos\_1045 | Isolimonic acid | 7.3054960651191e-05 | 1.50242949698664e-06 | 1.44831518902254e-07 | 2.23022790559257e-07 | 8.75992347225844e-07 | 5.99732268142455e-07 | 5.86106733992646e-07 | 1.11809097899302e-06 | 3.9710502329982e-07 | 1.37294654883651e-06 | 7.36162590950042e-07 | 1.56562794991941e-07 |
| pos\_1046 | Lucyoside R | 0.000121914279679639 | 4.34258375184982e-05 | 4.01999203982618e-05 | 3.55609604720819e-05 | 7.8805713351428e-05 | 7.54037427049922e-05 | 7.84818249263588e-05 | 6.10434689114453e-05 | 5.52443279306352e-05 | 5.80299527459421e-05 | 5.95030908565949e-05 | 6.15957869410449e-05 |
| pos\_1047 | N-Lactoyl ethanolamine | 0.000106684095280591 | 0.000111323525618239 | 8.69121051277265e-05 | 8.85622266420574e-05 | 0.000131990404978723 | 8.32228439425084e-05 | 0.000109674911445925 | 0.000191055941835352 | 0.000101551963976015 | 9.55689418410067e-05 | 8.65152051064008e-05 | 0.000131786455720573 |
| pos\_1048 | L-Threonine | 4.05166412149602e-06 | 4.95019749289224e-06 | 4.48707564812494e-06 | 4.10440011067578e-06 | 4.11317627433434e-06 | 4.63741768143139e-06 | 4.56469262162905e-06 | 4.25400835658093e-06 | 4.97641098872676e-06 | 3.85511733014764e-06 | 4.4420061594083e-06 | 9.490464280209e-06 |
| pos\_1049 | Lubiminol | 0.000134791036243688 | 0.000132298128344631 | 0.000126502125239268 | 0.000163141350875234 | 0.000157904095739051 | 0.000144757964596 | 0.000156027234307739 | 0.000147799865177536 | 0.00014058914522196 | 0.000149419229044028 | 0.000147110862476837 | 0.000155817190723342 |
| pos\_1050 | Brassinolide | 3.05413245354179e-06 | 4.78830947158439e-06 | 2.94779821333209e-05 | 6.5600688612823e-06 | 1.56123685783382e-06 | 6.89497571708909e-06 | 2.98166526330824e-05 | 2.67377848591743e-05 | 5.1795548620942e-06 | 1.08121866553027e-05 | 2.11903416894288e-05 | 2.50637559369956e-05 |
| pos\_1051 | Netilmicin | 7.235472780647e-06 | 1.77661218021961e-06 | 8.51425814103647e-07 | 3.82347619266298e-06 | 2.82432922456346e-06 | 3.92245308759846e-06 | 2.73199173761384e-06 | 1.89644771330146e-06 | 3.61854362311447e-06 | 2.71007949489542e-06 | 3.99634273557305e-06 | 4.00040334133602e-06 |
| pos\_1053 | DG(18:0e/2:0/0:0) | 0.000377622751092367 | 0.00024374083359807 | 0.000221812722810056 | 0.000426373391695709 | 0.000491933272263962 | 0.000367733956907418 | 0.000483518032929507 | 0.000363545410655954 | 0.000168644014344577 | 0.000330362429322766 | 0.000311991908719469 | 0.000168908051351534 |
| pos\_1054 | Farnesylcysteine | 5.69997995278618e-05 | 7.47177204875475e-05 | 0.000124096641775704 | 4.26122010928391e-05 | 4.04167306961233e-05 | 6.02313836532509e-05 | 3.84132371728819e-05 | 6.47861547949556e-05 | 8.04637621646833e-05 | 7.60027141821194e-05 | 6.4313804244299e-05 | 9.6351813876898e-05 |
| pos\_1055 | 7alpha,12alpha,26-trihydroxycholest-4-en-3-one | 0.000264842635770179 | 0.00029094232585622 | 0.000194010216677286 | 0.000306926742897239 | 0.000177412652330676 | 0.00024319723052925 | 0.000183618251754766 | 0.000182325865224609 | 0.000203820898416213 | 0.000308720350218827 | 0.000216376193362925 | 0.00016395292846032 |
| pos\_1056 | 4-(Methylthio)-2-butanol | 4.11006374474745e-06 | 5.877128266319e-06 | 7.27122116521195e-06 | 5.90479668295649e-06 | 4.73002107837413e-06 | 5.06780022314919e-06 | 3.8921768737568e-06 | 5.4961929984789e-06 | 4.87142827350352e-06 | 4.77756741257408e-06 | 5.32383788010287e-06 | 6.96429706786519e-06 |
| pos\_1057 | Temocapril | 3.57113202529351e-05 | 5.39616406115212e-06 | 7.21233539976199e-06 | 6.76813108145168e-06 | 1.96802930785152e-05 | 1.94792209586903e-05 | 1.85416559759462e-05 | 1.28829022980987e-05 | 1.09811893421723e-05 | 1.29074839303804e-05 | 1.34559738238059e-05 | 1.2214719856032e-05 |
| pos\_1058 | 4,5-Dihydro-1-benzoxepin-3(2H)-one | 2.11209365741551e-05 | 2.76998133988738e-05 | 0.000133949541380594 | 4.82801954081291e-05 | 2.78623617172906e-05 | 5.77249699886979e-05 | 9.95615975779274e-05 | 7.90604604235603e-05 | 2.48689226720371e-05 | 2.86097329984861e-05 | 6.16331586940204e-05 | 8.01655579499623e-05 |
| pos\_1059 | 5-Carboxy-2'-deoxyuridine | 1.62461122593232e-05 | 1.99073918968174e-05 | 1.89028051723122e-05 | 1.71402719856131e-05 | 1.45490464524617e-05 | 1.32522915911089e-05 | 1.48491285281678e-05 | 1.52688977357164e-05 | 1.76978895363328e-05 | 1.4463700509179e-05 | 1.67018873117699e-05 | 1.74435485424086e-05 |
| pos\_106 | (+)-4-Isocedren-15-al | 0.000141830648301967 | 0.000159877513935321 | 0.000249258399322105 | 0.000237653388025998 | 0.000251985950295482 | 0.000221803645428043 | 0.000208170969335697 | 0.000215631634365546 | 0.000235570024572302 | 0.000212218618308592 | 0.000219245484744823 | 0.000260934290521194 |
| pos\_1060 | Sinapoylspermine | 2.43199929846309e-05 | 1.25084416238952e-05 | 5.9454356726755e-05 | 5.604195120563e-06 | 7.00954897594608e-06 | 1.92277791159387e-05 | 1.51226318597512e-05 | 4.60217290882986e-05 | 5.94550073521482e-05 | 2.2454193507884e-05 | 4.47825968234503e-05 | 9.91541774393212e-05 |
| pos\_1061 | Sarcodon scabrosus Depsipeptide | 1.31911265762948e-05 | 1.81224268231802e-05 | 9.86619563924405e-06 | 1.28268848210931e-05 | 1.13772257807579e-05 | 1.15977214567806e-05 | 1.30896540438401e-05 | 9.18338762931178e-06 | 7.58244055129016e-06 | 2.59216216750135e-05 | 1.12505769763983e-05 | 7.27046661870651e-06 |
| pos\_1062 | Annosquamosin B | 1.98937896620918e-05 | 6.35303672962545e-05 | 2.97379404502007e-05 | 1.70416470523192e-05 | 2.45591872657214e-05 | 3.27378501633593e-05 | 1.33273430374733e-05 | 2.62779653633443e-05 | 1.49999959088291e-05 | 5.77467472167914e-05 | 3.15043265688175e-05 | 4.89034882978643e-05 |
| pos\_1064 | Bupivacaine | 5.58127523046011e-05 | 6.22016430072608e-05 | 5.66910247113692e-05 | 6.65220547301747e-05 | 6.27387756747094e-05 | 6.17502116698422e-05 | 6.62956306499126e-05 | 6.19526557237452e-05 | 5.46312564693785e-05 | 6.14406928879402e-05 | 5.93551273347659e-05 | 5.32608118475179e-05 |
| pos\_1065 | alpha-Ionol O-[arabinosyl-(1->6)-glucoside] | 0.000476443568174675 | 0.000528862192049414 | 0.000607797587199994 | 0.000444095255899901 | 0.000373715309317348 | 0.000417532075406124 | 0.000700744682964467 | 0.000626208795270372 | 0.000385047212255852 | 0.000595971791728165 | 0.000594726250361319 | 0.000337702726877087 |
| pos\_1066 | cis-Uvariamicin IB | 0.000809367854853137 | 0.000796504721403142 | 0.000687937341581874 | 0.000513703242652272 | 0.00039336653218025 | 0.000581272001597639 | 0.000425968546430929 | 0.00100028256204726 | 0.000763608961560663 | 0.000692555379911259 | 0.00105314800324955 | 0.00126448301628163 |
| pos\_1067 | PE(22:0/24:1(15Z)) | 0.000226223369723558 | 0.000147065998330439 | 0.000147966099856976 | 0.000136042311992654 | 0.000132917657056432 | 0.000126000240511002 | 0.000148426391208315 | 0.000125265946560295 | 0.00013004756865257 | 0.000119848894970489 | 0.000121700280385796 | 0.000123066284109277 |
| pos\_1068 | Soyacerebroside I | 0.000256315408306972 | 0.000467384706124616 | 0.000352274144028354 | 0.000303270718411685 | 0.000422923136947403 | 0.000337327107287331 | 0.000462868662184941 | 0.000431787108479591 | 0.000283156459695236 | 0.000274574382880544 | 0.000344745062895244 | 0.000349958961317058 |
| pos\_1069 | Octadecyl cis-p-coumarate | 0.00019012631339607 | 0.000189073327747081 | 0.000152261376143215 | 0.000181194660253643 | 0.000249811358353206 | 0.000187231039857354 | 0.000215401552400508 | 0.000199900093824618 | 0.000199708711226894 | 0.000188691673855588 | 0.000185905942233582 | 0.000152314984363187 |
| pos\_107 | Isomonospermoside | 0.000151490010039457 | 0.000211260915116656 | 7.16071816742406e-05 | 0.000103305386915879 | 0.000102466999656699 | 0.000134213918630688 | 0.000260406503380859 | 0.000137893598976338 | 5.18515099828944e-05 | 0.000169091399700766 | 0.00013715045572474 | 4.37213602160968e-05 |
| pos\_1070 | (R)-N-Methylsalsolinol | 2.02453616612103e-05 | 2.1700550838048e-05 | 3.6253236876486e-05 | 3.91436800305081e-05 | 2.6621985381773e-05 | 2.7631231371766e-05 | 2.48250639533082e-05 | 2.64392643339594e-05 | 2.19882767187633e-05 | 2.20846782323623e-05 | 2.44250796653759e-05 | 3.19122158939323e-05 |
| pos\_1071 | (9E)-10-nitrooctadecenoic Acid | 5.90089593573648e-05 | 6.82108549705032e-05 | 6.36604820308705e-05 | 7.07145080791977e-05 | 6.57869459843519e-05 | 7.14883622091864e-05 | 6.99545191003274e-05 | 7.86101463131096e-05 | 6.90957967183923e-05 | 6.67823083039699e-05 | 7.53861434366368e-05 | 7.1814292664596e-05 |
| pos\_1072 | 2-Acetyl-3-ethylpyrazine | 5.99389371988428e-05 | 5.50165872452064e-05 | 3.65361556875447e-05 | 4.03444460976603e-05 | 5.57250026429055e-05 | 4.85752372912038e-05 | 5.07791397237571e-05 | 4.9708740440557e-05 | 4.67247793521062e-05 | 5.13802913241004e-05 | 5.21211222822574e-05 | 6.22919185682759e-05 |
| pos\_1073 | Neolinustatin | 5.96503890331222e-06 | 9.85186204859414e-06 | 6.88259674573533e-06 | 7.88622271234797e-06 | 8.65184137415296e-06 | 8.04562355504655e-06 | 1.11270187582944e-05 | 9.08477593083803e-06 | 1.27405048068724e-05 | 8.4786020981128e-06 | 9.8469071217803e-06 | 1.46742998177055e-05 |
| pos\_1074 | 1-Phenyl-1,3-eicosanedione | 7.41246626883363e-05 | 6.30209587896793e-05 | 7.6604573052796e-05 | 9.55346294869445e-05 | 0.000112836711365787 | 8.50015428757227e-05 | 0.000141975325089897 | 0.000100832009255239 | 8.57165103864387e-05 | 8.12473920405083e-05 | 0.000103822166707404 | 8.53410898646764e-05 |
| pos\_1075 | 14,15-EpETE | 3.42658009668608e-05 | 3.61829149192699e-05 | 2.93476991371288e-05 | 3.76211291157617e-05 | 3.31849553628311e-05 | 3.73866626978872e-05 | 3.73073499524725e-05 | 3.36160818731272e-05 | 4.0337761902668e-05 | 3.90011510949411e-05 | 3.74717070930615e-05 | 3.22958507905294e-05 |
| pos\_1077 | Flavoxanthin | 0.0100772326560888 | 0.0136151297478074 | 0.00987668440453529 | 0.0118528972552607 | 0.00796542522667326 | 0.0120770241814317 | 0.0122096116136428 | 0.0114217718921549 | 0.00888232041948897 | 0.010560858486968 | 0.0121256103539167 | 0.00761184382388798 |
| pos\_1078 | Vinaginsenoside R14 | 0.000429671630750674 | 0.000170526039036574 | 0.000110252243989198 | 8.59901306632165e-05 | 0.00022735378676196 | 0.000218982469080407 | 0.000195742395904942 | 0.000167556552895359 | 0.000130972138286168 | 0.000153978294013791 | 0.000157237068157553 | 0.000142924880564021 |
| pos\_1079 | Cer(d18:1/14:0) | 3.07946144923707e-05 | 4.01110645253076e-05 | 4.33888662074285e-05 | 4.47438196116195e-05 | 3.4857537630565e-05 | 4.21815937718274e-05 | 4.60028511024693e-05 | 4.13993081395398e-05 | 4.13122601478002e-05 | 4.44303844599318e-05 | 4.33327653199249e-05 | 3.95087610353954e-05 |
| pos\_108 | RHOIFOLIN | 3.78775372116092e-06 | 1.82688113090293e-05 | 3.57014084103671e-05 | 2.0370241796344e-06 | 9.23534477713109e-06 | 1.09351704987591e-05 | 8.49582130333884e-05 | 6.2976908725647e-05 | 1.71236608976507e-05 | 3.84908284112322e-05 | 4.17430306845687e-05 | 7.69590820373692e-05 |
| pos\_1080 | MG(0:0/15:0/0:0) | 0.000205671062942439 | 0.000151397921924067 | 0.000248769758624607 | 0.000111950576707639 | 9.64819907105611e-05 | 0.000144366584022575 | 8.46359258154754e-05 | 0.000145253670917212 | 0.000238935285218082 | 9.45524965266186e-05 | 0.000158444785084034 | 0.000427374927766811 |
| pos\_1081 | Trifluridine | 1.38639346664249e-05 | 3.46024532309961e-06 | 6.79214281852925e-06 | 5.29873849732072e-05 | 0 | 1.53633845157463e-05 | 0 | 2.28186124481502e-06 | 1.03863726550481e-06 | 5.56081043657279e-07 | 1.90508204404573e-06 | 6.24506821834786e-06 |
| pos\_1082 | Austalide J | 7.72436681914082e-06 | 5.77212411042449e-06 | 5.1208646200273e-06 | 4.68771791326203e-06 | 4.79907987979426e-06 | 6.06185117303307e-06 | 5.61928116122186e-06 | 5.80946092072386e-06 | 5.02492358592258e-06 | 5.35646762911605e-06 | 4.77932757289988e-06 | 6.16523289543051e-06 |
| pos\_1083 | Pitheduloside I | 0.00235551463032745 | 0.00341165809646247 | 0.00505944210863012 | 0.00373293426472837 | 0.00232697166691747 | 0.00338554841617111 | 0.00381583706193539 | 0.00464236761803373 | 0.00417403503272438 | 0.00315293510489168 | 0.00442686937113818 | 0.00583910517955017 |
| pos\_1084 | Annuolide B | 9.57631855856293e-06 | 1.39749129746319e-05 | 1.34336529147653e-05 | 2.30985608998526e-05 | 1.50685926124131e-05 | 1.57294189659119e-05 | 1.54944063392194e-05 | 1.46292748731029e-05 | 1.18354641529786e-05 | 1.28126796351198e-05 | 1.53153596341487e-05 | 1.84003657502491e-05 |
| pos\_1085 | Arginyl-Proline | 2.43903308982333e-05 | 3.60239900448318e-05 | 2.52747472328279e-05 | 2.83507358472855e-05 | 3.19221103502873e-05 | 2.87378333398314e-05 | 2.60012524182189e-05 | 3.02066046488444e-05 | 2.98336580654547e-05 | 3.38894340348599e-05 | 2.99671702921087e-05 | 2.81945111423615e-05 |
| pos\_1086 | triazolopropionic acid | 3.96353834726837e-06 | 6.71277070187676e-06 | 9.41857263518503e-06 | 6.81694841920746e-06 | 5.03068245011862e-06 | 6.38822379027588e-06 | 7.92841909838451e-06 | 6.08353300441985e-06 | 1.12923341496878e-05 | 4.23585775414029e-06 | 8.53512893355488e-06 | 5.14692809809177e-06 |
| pos\_1087 | Galactosylglycerol | 7.80644405300538e-05 | 0.000135269494208746 | 6.26963342866832e-05 | 8.5621692013966e-05 | 0.000115878860875204 | 8.98401119649737e-05 | 6.88038660247903e-05 | 6.76347063075463e-05 | 0.000125382611155969 | 0.000105747463116877 | 8.95006734654301e-05 | 7.68204472448298e-05 |
| pos\_1088 | 19-hydroxyprostaglandin H2(1-) | 2.26265739660345e-06 | 3.96131447372995e-06 | 4.46835936187634e-06 | 1.2541344308847e-06 | 4.29698679763036e-06 | 3.07362252810833e-06 | 8.26785297774938e-06 | 4.92944987623281e-06 | 1.85808221680399e-06 | 2.76221726519672e-06 | 4.16349399814787e-06 | 4.02361528007517e-06 |
| pos\_1089 | Glaucarubolone 15-O-beta-D-glucopyranoside | 7.65698855760424e-06 | 1.41900294702073e-05 | 1.36305416207823e-06 | 4.39290733422478e-06 | 5.40339946003512e-06 | 3.63996885034775e-06 | 2.67242603534655e-06 | 1.67494870400126e-06 | 2.12659001542272e-06 | 5.94840317309045e-06 | 3.07367748450785e-06 | 5.86217773241514e-05 |
| pos\_109 | Acetylenic acids; 10,16-Heptadecadien-8-ynoic acid, (E)- | 5.76642073329297e-05 | 5.66529643482266e-05 | 0.000119815665977017 | 0.000107651681836772 | 0.000138369539419973 | 0.000103935077167936 | 9.02499663551588e-05 | 9.48566256793469e-05 | 0.000119014444993059 | 9.59629377293781e-05 | 0.000102414351168837 | 9.85139171833944e-05 |
| pos\_1090 | (R)-Heraclenol 2'-(3-methylbutanoate) | 3.89749162081281e-05 | 3.92726282854609e-05 | 1.63605619778763e-05 | 3.2591341187647e-05 | 3.34692201431646e-05 | 3.0735537792979e-05 | 3.12949710646439e-05 | 2.37348248760016e-05 | 1.92021975567236e-05 | 3.95544524420193e-05 | 2.8264547677999e-05 | 2.44610562191136e-05 |
| pos\_1091 | (E,E)-2,4-Hexadienal | 1.75696915153705e-05 | 3.14949645694637e-05 | 1.64544514059761e-05 | 1.91109690762449e-05 | 2.63300397983134e-05 | 2.13289794650217e-05 | 2.24351982186845e-05 | 1.93717138763694e-05 | 1.92186147174324e-05 | 2.56613483427326e-05 | 2.01477374306232e-05 | 1.8223637975959e-05 |
| pos\_1092 | Octreotide | 7.14977686995547e-06 | 9.30338242779698e-06 | 1.62827032372552e-07 | 3.14979490949438e-07 | 5.61791181903807e-06 | 5.3263695338409e-06 | 1.69221391984726e-05 | 4.3660220099043e-06 | 1.1421920744868e-05 | 1.42980946645792e-07 | 9.73857669869703e-06 | 1.14818203561305e-06 |
| pos\_1093 | 9'-desmethylgranisetron | 1.42933039010433e-06 | 3.05192593619281e-06 | 5.15165623688812e-06 | 1.26225453010114e-06 | 1.29277253618395e-06 | 1.12511602897135e-06 | 1.02401960343051e-06 | 9.84106773636294e-07 | 3.09341004224328e-07 | 3.80840861187576e-07 | 7.82614102030438e-07 | 2.75770008607565e-05 |
| pos\_1094 | N-Monodemethyl roxithromycin | 8.95558216869551e-05 | 0.000109137806607339 | 0.000111233920960069 | 0.000140849449494431 | 8.23801980893444e-05 | 8.47044669545542e-05 | 8.81782702601784e-05 | 7.93709580857676e-05 | 9.32476229105806e-05 | 9.7455858850682e-05 | 0.000104634600741942 | 8.40825755182909e-05 |
| pos\_1096 | 20-oxo-leukotriene B4 | 6.97059379009096e-06 | 8.82416436862381e-06 | 7.33869270545804e-06 | 5.35188354301671e-06 | 2.57266876458473e-06 | 7.50615976033672e-06 | 4.10093553136026e-06 | 5.94746074923862e-06 | 7.79471130239226e-06 | 3.86645759580093e-06 | 6.79336496773085e-06 | 7.57344274390507e-06 |
| pos\_1097 | DG(15:0/18:4(6Z,9Z,12Z,15Z)/0:0) | 0.000140286683351745 | 0.00016709267303867 | 0.000151522325007385 | 0.000148123294552938 | 0.000119040218748802 | 0.000130605438083034 | 0.000136654178525408 | 0.000145985097081291 | 0.000159518780344662 | 0.000141430562969018 | 0.000141245855012461 | 0.000137381942570948 |
| pos\_1098 | 6,9,12,15,18,21-Tetracosahexaenoic acid | 7.02460324981086e-06 | 1.13052721499416e-05 | 7.93586077599539e-06 | 8.35754388439846e-06 | 5.66949130613408e-06 | 8.91701389460395e-06 | 7.39728927974189e-06 | 8.51153418171144e-06 | 9.4273529924651e-06 | 6.41236106448897e-06 | 9.09130623038826e-06 | 8.4916195554742e-06 |
| pos\_1099 | noroxymorphone | 0.000198608540701613 | 0.000153472507352637 | 0.000165361913492948 | 0.000107625960241052 | 0.000217508638735704 | 0.000184102689038013 | 0.000132324096121992 | 0.000149084632352314 | 0.00017518225452934 | 0.000135336151002151 | 0.000149811990007791 | 0.000196080947393376 |
| pos\_110 | 4,6-heptadienal | 4.86935893708583e-05 | 6.95369741017906e-05 | 6.54676775662883e-05 | 6.53812813151344e-05 | 4.92239768212523e-05 | 5.36520858142986e-05 | 4.7276642951447e-05 | 6.05170668987901e-05 | 5.97552828726393e-05 | 5.28060527328542e-05 | 5.96050365416467e-05 | 5.65044764439405e-05 |
| pos\_1100 | 3a,7a-Dihydroxycoprostanic acid | 0.000348403837339213 | 0.000280366265856763 | 0.000408539559064586 | 0.000505773212758651 | 0.00058793819024831 | 0.000433524118633258 | 0.000301090542259615 | 0.000427113451534103 | 0.000513888875433055 | 0.00041382674643983 | 0.000469214020758835 | 0.000600349470391374 |
| pos\_1101 | Licorice glycoside D1 | 0.000376554780975181 | 0.000347596813525257 | 0.000127040415818954 | 0.000235565599602341 | 0.000402181536871603 | 0.000321049085193097 | 0.000175167601524036 | 0.000137995092024966 | 0.000194095616979737 | 0.000369473113207881 | 0.000199042838561865 | 0.000104396872529043 |
| pos\_1102 | alpha-D-Xylopyranosyl-(1->6)-beta-D-glucopyranosyl-(1->4)-D-glucose | 4.17826609103079e-06 | 1.2343519330315e-05 | 4.93544574096887e-06 | 2.1104414017877e-06 | 6.05881533456244e-06 | 7.07134039249448e-06 | 4.89751170458604e-06 | 3.54730352353346e-06 | 8.59151737455894e-06 | 1.19312306463555e-05 | 1.03722428993571e-05 | 7.7278223224841e-06 |
| pos\_1103 | 6-Angeloylfuranofukinol | 2.33144710307891e-05 | 0.000100573096788742 | 7.00542892026619e-05 | 8.21962207895589e-05 | 3.36432406352874e-05 | 4.22300861467842e-05 | 4.30936665999658e-05 | 4.64521608095571e-05 | 4.02417848417049e-05 | 2.02638922500499e-05 | 2.58301467075833e-05 | 3.42668655474869e-05 |
| pos\_1104 | Doxycycline | 1.51847464137205e-05 | 2.3714761190215e-05 | 3.73206597040463e-06 | 1.89948814421773e-05 | 1.54653550351419e-05 | 1.36884069876972e-05 | 9.16652951424469e-06 | 3.88837308366332e-06 | 2.93370288136774e-06 | 2.53773336780352e-05 | 6.95040072269308e-06 | 1.98470096910529e-06 |
| pos\_1105 | Saringosterol 3-glucoside | 0.00802894209949478 | 0.00306693255619533 | 0.00283654525033538 | 0.00234284006369174 | 0.00361473611207225 | 0.00360471379228895 | 0.00388364126982978 | 0.00329642526713872 | 0.00317942922297111 | 0.00306515257830478 | 0.00331253585764484 | 0.0032705274509047 |
| pos\_1106 | (3beta,11alpha,13beta)-3,11,13-Oleananetriol | 1.48309608401691e-06 | 3.7717309116365e-06 | 9.28578968775202e-06 | 9.68805848424686e-07 | 3.70749393628482e-06 | 3.58853850560766e-06 | 2.30100974847732e-06 | 3.29578918434069e-06 | 1.66150943007112e-06 | 2.19724539154711e-06 | 5.79069714071338e-06 | 3.9984441778844e-05 |
| pos\_1107 | (5R)-5-Hydroxyhexanoic acid | 0.000417252134260373 | 0.000285253272032136 | 0.000273654475579724 | 0.000241455495777314 | 0.000263878477496856 | 0.000282729788503351 | 0.000302364826527774 | 0.000290480453869925 | 0.000282091505160416 | 0.000269369118546198 | 0.000276866804161708 | 0.000293018987018842 |
| pos\_1108 | 13'-Carboxy-alpha-tocotrienol | 0.000221343588337577 | 0.000231350867734143 | 0.000310437504709171 | 0.000319160919013412 | 0.000252019133767807 | 0.000266960951232361 | 0.000325985651588932 | 0.000301838242901421 | 0.000271753427785651 | 0.00022194302997239 | 0.000280242476367214 | 0.000252808746057233 |
| pos\_1109 | Digitoxin | 0.000467780380003551 | 0.000120557581392474 | 0.000121793393565843 | 9.94234514465586e-05 | 0.000264111947792101 | 0.000270202750779115 | 0.000236394691762213 | 0.0002118110683788 | 0.000155729816269607 | 0.000178885282087988 | 0.000194759842382242 | 0.000175242116450201 |
| pos\_111 | PE(16:0/0:0) | 0.00300536694767331 | 0.00287317947780359 | 0.0024631817672551 | 0.00356735876284141 | 0.00211097139637633 | 0.00278271686302296 | 0.00313293383483993 | 0.00322939376462565 | 0.00223801583863511 | 0.00339960244002344 | 0.00303257364417858 | 0.00292537918702298 |
| pos\_1110 | 6-Keto-prostaglandin F1a | 0.00261119157726958 | 0.00119421857920501 | 0.0011072154675643 | 0.000973760660622241 | 0.00171342686552684 | 0.00170067363067146 | 0.00186587952420425 | 0.00145519318562285 | 0.00138065391212545 | 0.00136161529085922 | 0.00146959423021067 | 0.001479899465742 |
| pos\_1111 | 1-(2,4,5-Trimethoxyphenyl)-1,2-propanedione | 3.68700298951886e-05 | 4.36711143151978e-05 | 1.89431433666181e-05 | 2.78684382529368e-05 | 3.97009119435493e-05 | 3.14942863943825e-05 | 0.00104800478639478 | 0.00044738959164134 | 2.85653600017452e-05 | 3.17071074912861e-05 | 0.000302737116599381 | 2.3287784083775e-05 |
| pos\_1112 | Glyceryl 5-hydroxydecanoate | 6.77034764234987e-05 | 0.000199503242433711 | 0.000113197627407317 | 6.12561653500554e-05 | 8.65168984283526e-05 | 0.000102883819629769 | 3.85361605420345e-05 | 7.83200041314213e-05 | 4.54812453062609e-05 | 0.000179776135777695 | 9.15499545411537e-05 | 0.000148823078189182 |
| pos\_1113 | 2-Acetyl-3,5,5,6,8,8-hexamethyl-5,6,7,8- tetrahydronaphthalene | 6.99532046500781e-05 | 6.94462005082805e-05 | 7.27930094738459e-05 | 7.04567381823745e-05 | 8.125182362927e-05 | 7.06058457743423e-05 | 6.83211200526863e-05 | 7.30215672613618e-05 | 9.48518186162534e-05 | 8.02868839081512e-05 | 8.27403261278513e-05 | 7.80319747376229e-05 |
| pos\_1114 | N-Docosahexaenoyl phenylalanine | 0.000139887308165014 | 0.000156327255379418 | 0.000148411258153763 | 0.000141061048581078 | 0.000122653602883356 | 0.000142436192189861 | 0.000140566608155614 | 0.0001408078107194 | 0.000165643621269518 | 0.000137062501575508 | 0.000141061456907141 | 0.000128006357010359 |
| pos\_1115 | PG(16:1(9Z)/18:1(11Z)) | 0.0116257898999275 | 0.0131488642717179 | 0.0120538823347262 | 0.0111446662415583 | 0.00927481642302788 | 0.0103771866617167 | 0.00910210362427596 | 0.0101800268680763 | 0.0116963889010826 | 0.00938919024202001 | 0.0102229163609914 | 0.00946744352577267 |
| pos\_1116 | 2,6-Cyclolycopene-1,5-diol | 0.000338852021423393 | 0.00020383752304253 | 0.000205733896018339 | 0.000207759096788693 | 0.000250177229786744 | 0.000195395653190675 | 0.000219284538445175 | 0.000302765803733405 | 0.000199569601447179 | 0.000239097655305419 | 0.000178564972444387 | 0.000225494797609368 |
| pos\_1117 | Auxin b | 7.99613042420407e-05 | 9.84664924173189e-05 | 9.02332922295829e-05 | 8.73042741896484e-05 | 7.39020915505291e-05 | 8.52521423628869e-05 | 8.46350356818322e-05 | 9.02908432093084e-05 | 9.20981677883024e-05 | 9.34448069613459e-05 | 8.90519554028468e-05 | 8.32993539662929e-05 |
| pos\_1118 | Yucalexin P21 | 8.58020868255383e-05 | 0.000103294663878938 | 9.13838619354542e-05 | 0.000103967252323942 | 9.53344354820337e-05 | 9.54624785657737e-05 | 9.59687544299865e-05 | 9.29520787556944e-05 | 8.58470345846735e-05 | 0.000104391523725997 | 9.55020449736752e-05 | 8.95953387513134e-05 |
| pos\_1119 | Lyciumin A | 7.32134153706363e-06 | 2.13452097940918e-05 | 5.26630187804305e-06 | 8.26771638220247e-06 | 9.12581753817921e-06 | 8.85557408903864e-06 | 7.89847011834225e-06 | 5.51255477622023e-06 | 1.01068734701599e-05 | 8.75526514705158e-06 | 8.43550830482454e-06 | 6.2563397242445e-06 |
| pos\_112 | Hyacinthin | 6.01415391333233e-05 | 0.000131747586482899 | 8.17747557657224e-05 | 0.00014190716213136 | 7.9042942086049e-05 | 0.000104040528510986 | 6.90257693794996e-05 | 8.73760345642211e-05 | 0.000206964308262416 | 7.52547751045857e-05 | 0.000152742794127259 | 0.000141495622725756 |
| pos\_1120 | Ascochitine | 3.72094681091607e-05 | 5.84673907299818e-05 | 3.66921520925922e-05 | 4.6084716757116e-05 | 4.68834527389815e-05 | 3.85345558656832e-05 | 4.0914225485486e-05 | 4.08196601983785e-05 | 4.51073953877123e-05 | 4.88223530133163e-05 | 4.18790551012635e-05 | 2.83054114477801e-05 |
| pos\_1122 | Desflurane | 1.65948588385725e-05 | 2.78308373801605e-05 | 1.35583073022323e-05 | 1.43343709854028e-05 | 1.51104607376437e-05 | 1.39409874422138e-05 | 1.53706661499096e-05 | 1.11870432211643e-05 | 1.63928559579705e-05 | 8.60115055409808e-06 | 1.39566420908446e-05 | 1.23260315040459e-05 |
| pos\_1123 | (S)-N-(4,5-Dihydro-1-methyl-4-oxo-1H-imidazol-2-yl)alanine | 5.60765745920808e-06 | 2.12216575324237e-06 | 1.45832819164012e-06 | 1.35005777165612e-06 | 3.29609581388344e-06 | 2.04759010308574e-06 | 2.17214296713505e-06 | 1.35629491896643e-06 | 2.50961986237688e-06 | 1.47554508830827e-06 | 1.92264647165297e-06 | 5.22162436743991e-06 |
| pos\_1124 | Butenylcarnitine | 3.16992475366062e-05 | 1.73989049749822e-05 | 0.000183366365940754 | 0.000476829500493218 | 8.76193668648426e-05 | 0.000145990403408562 | 7.27474710592147e-05 | 0.000330713610661672 | 1.35173456853546e-05 | 2.31771690295756e-05 | 0.000218141383527701 | 0.000877607082103626 |
| pos\_1125 | PS(18:1(9Z)/18:2(9Z,12Z)) | 0.00381095036607501 | 0.00403521611338266 | 0.00371600337784222 | 0.00352513244755268 | 0.00282111122145249 | 0.00312373436888315 | 0.0026824840005579 | 0.00314700302186808 | 0.00349878381084893 | 0.00287484637814352 | 0.00309494237336883 | 0.00294072329005139 |
| pos\_1127 | 11'-Carboxy-gamma-chromanol | 0.000284122315862938 | 0.000439178697424055 | 0.000234789331557595 | 0.000427814996000948 | 0.00036642503477518 | 0.000357659054770402 | 0.000559934531808377 | 0.000428727106197649 | 0.000432440428959989 | 0.000363195343851445 | 0.000453171014557383 | 0.000234476615781695 |
| pos\_1128 | Germacrenone | 1.23250554389982e-05 | 1.69399771067969e-05 | 1.582401495655e-05 | 1.34977744508363e-05 | 1.49095384966467e-05 | 1.34011167263024e-05 | 1.7930277194757e-05 | 1.78366551089813e-05 | 1.55487727746986e-05 | 1.53698330271106e-05 | 1.61047057375462e-05 | 2.56768917624143e-05 |
| pos\_1129 | Octanoylglucuronide | 1.51104462466777e-05 | 2.25253991540762e-05 | 8.67357509760161e-06 | 1.54023494951205e-05 | 2.21730229200184e-05 | 1.63984381364678e-05 | 1.91707303967034e-05 | 1.49879124301617e-05 | 1.26574669936424e-05 | 1.95293616848153e-05 | 1.72183793885763e-05 | 1.16486103263412e-05 |
| pos\_113 | TyrMe-Ile-OH | 7.59913891908409e-06 | 4.03876118827933e-05 | 7.26499846841624e-07 | 8.50517912699751e-07 | 8.76331155561977e-08 | 3.00777569941681e-07 | 3.97003263777179e-07 | 6.94079648427177e-07 | 2.25883483501505e-07 | 1.29876554444314e-06 | 5.11563828792648e-07 | 3.43192494458743e-07 |
| pos\_1130 | Tripelennamine | 2.90570387738273e-05 | 2.87973609376459e-05 | 1.88618060688662e-05 | 1.94858454173233e-05 | 2.59397252245837e-05 | 2.27119592211572e-05 | 2.31502859121247e-05 | 2.12076415659828e-05 | 1.82904622729169e-05 | 2.35595518337223e-05 | 2.28694287896063e-05 | 2.35281474622392e-05 |
| pos\_1131 | DG(22:6(4Z,7Z,10Z,13Z,16Z,19Z)/18:0/0:0) | 6.23874571125796e-05 | 1.02617891288301e-05 | 7.0759421750313e-06 | 2.5239081290448e-06 | 6.80651826173019e-05 | 2.82746672837383e-05 | 6.19867694682542e-05 | 5.86829021339649e-05 | 1.74685799221951e-05 | 0.000106095541287247 | 5.62905087017876e-05 | 3.8325855891683e-05 |
| pos\_1132 | Trihexosylceramide (d18:1/22:0) | 7.03650421149509e-05 | 4.80698628523893e-05 | 4.24548342221493e-05 | 4.42589598145811e-05 | 4.41580164442006e-05 | 4.56949921851893e-05 | 4.69726648132722e-05 | 4.84396873837109e-05 | 4.66904538360203e-05 | 4.8504777301827e-05 | 4.65408309641998e-05 | 4.71565564362917e-05 |
| pos\_1133 | 1-(3,5-Dihydroxyphenyl)-2-pentadecanone | 4.16938957993708e-05 | 4.3483998952872e-05 | 4.10149859993149e-05 | 3.53537382108312e-05 | 3.85768279915472e-05 | 4.08617090644012e-05 | 2.95133028410148e-05 | 3.75435339553621e-05 | 3.71797809782533e-05 | 5.28320332949445e-05 | 3.99828080276213e-05 | 4.30012938813296e-05 |
| pos\_1135 | (S)-Argpyrimidine | 0.000111460767771895 | 9.48839835491075e-05 | 9.08262437363655e-05 | 7.95503210924722e-05 | 8.10656502145795e-05 | 8.80450874044308e-05 | 8.80273457404817e-05 | 8.9319423076233e-05 | 9.15076169598596e-05 | 8.42795701736856e-05 | 8.77557499777824e-05 | 8.87314301080973e-05 |
| pos\_1136 | Uralenolide | 0.00012797224835355 | 0.000183101625081416 | 8.43552777705601e-05 | 0.000105063865685357 | 8.05523664038767e-05 | 0.000116179910723084 | 0.000127743084391693 | 0.000166082524328515 | 0.000117622743659662 | 0.000156538429151503 | 0.000154565350589436 | 0.000244564840102378 |
| pos\_1137 | Vinaginsenoside R5 | 0.000116418634861374 | 1.98794122218047e-05 | 2.49497297635004e-05 | 2.51049798328565e-05 | 6.97442521018488e-05 | 7.29398998724739e-05 | 5.83592612988263e-05 | 4.24907457432618e-05 | 3.8473049213513e-05 | 3.94197160017706e-05 | 4.48043131147436e-05 | 3.78692521300802e-05 |
| pos\_1138 | Epitheaflagallin 3-O-gallate | 3.37406665788188e-05 | 5.42621412567624e-06 | 8.85400566269635e-06 | 7.10115578981515e-06 | 1.7850008927888e-05 | 1.81948714754241e-05 | 1.65510857245035e-05 | 1.44662932352948e-05 | 1.2502424900545e-05 | 1.06436489570425e-05 | 1.22429452810956e-05 | 1.26799818235e-05 |
| pos\_1139 | dapsone hydroxylamine | 4.91549039822314e-05 | 7.78231001790698e-05 | 5.04066395086156e-05 | 7.87428874656303e-05 | 4.70654619208365e-05 | 6.02093298775753e-05 | 0.000179488170271839 | 4.16674624583095e-05 | 7.61516256586816e-05 | 6.60173186837613e-05 | 6.75615977205772e-05 | 7.53934047928043e-05 |
| pos\_114 | 1-Hydroxy-2-(beta-D-glucosyloxy)-9,10-anthraquinone | 1.10732069209245e-06 | 2.59014230757853e-06 | 8.09291477016776e-07 | 2.62057201112266e-06 | 1.60859380685155e-06 | 1.64538471902959e-06 | 1.87860000070578e-06 | 1.25016957399187e-06 | 3.21281996425466e-06 | 3.18093650072597e-06 | 1.94239446494537e-06 | 0.000213845546092148 |
| pos\_1140 | 2,3-Dihydro-5,5',7,7'-tetrahydroxy-2-(4-hydroxyphenyl)[3,8'-bi-4H-1-benzopyran]-4,4'-dione | 2.2901186222075e-05 | 3.0272290577616e-05 | 9.8877927459786e-06 | 1.00482930718052e-05 | 1.14103120878369e-05 | 2.04703569829563e-05 | 4.6891298583239e-05 | 1.3876221230749e-05 | 6.54238048184648e-06 | 1.65711829316501e-05 | 2.58303236804164e-05 | 5.27856590930183e-06 |
| pos\_1141 | Isovalerylglutamic acid | 1.72535810088484e-05 | 2.5607171467383e-05 | 8.31919499750707e-06 | 1.77626953626292e-05 | 2.03288102216096e-05 | 1.50831721446601e-05 | 4.73858259215339e-05 | 3.1145888413144e-05 | 3.6666681758369e-05 | 2.53988822599635e-05 | 3.35311177847392e-05 | 1.32588457165065e-05 |
| pos\_1142 | 8-Oxohexadecanoic acid | 3.2615809818917e-05 | 3.60292019592936e-05 | 6.25403914675215e-05 | 4.31917720790492e-05 | 5.31229229463143e-05 | 4.84376994141158e-05 | 3.15311670743297e-05 | 3.39644574406476e-05 | 4.90673620473906e-05 | 4.71906010790507e-05 | 3.8942897480649e-05 | 4.24379806984463e-05 |
| pos\_1143 | PE(15:0/20:2(11Z,14Z)) | 7.45195400458137e-06 | 6.70664798489389e-06 | 1.85788846995226e-06 | 1.71658318304358e-06 | 6.36832831399463e-06 | 5.25051210775874e-06 | 2.1367799335147e-06 | 1.36535314825349e-06 | 9.18290147686821e-07 | 1.30459372443255e-06 | 5.13156736766791e-06 | 1.34561322289143e-06 |
| pos\_1145 | PC(15:0/22:5(4Z,7Z,10Z,13Z,16Z)) | 0.000102013880147647 | 0.000152492251334751 | 0.000407752578443822 | 9.12910474417522e-05 | 0.000181876562755282 | 0.000191998596943191 | 0.000220988191379428 | 0.000236587057216014 | 0.000112738624568061 | 0.000322449850516079 | 0.000384371970696536 | 0.000392483684688169 |
| pos\_1146 | Norethindrone | 8.47239892865223e-05 | 0.000155847164350949 | 0.000143401406012619 | 0.000130384728874745 | 9.68454630025855e-05 | 0.000118961840770104 | 0.000103515328635137 | 0.000118042771719072 | 0.000135496642751571 | 0.000113086140570205 | 0.000121810563324284 | 0.000113360305802659 |
| pos\_1147 | DG(14:1(9Z)/15:0/0:0) | 8.96548462783599e-06 | 2.02362385694286e-05 | 0 | 0 | 4.45196872364774e-05 | 1.31697119191229e-05 | 2.27399563169784e-07 | 0 | 2.64768061513801e-05 | 0 | 3.98258333199166e-06 | 7.26461297244015e-08 |
| pos\_1148 | Corticosterone | 0.00020104773142442 | 7.4984878856135e-05 | 7.66539621135424e-05 | 6.60637051393494e-05 | 0.000125723395393402 | 0.000130840524541348 | 0.000122560342350822 | 0.000109910181933234 | 9.20818829296306e-05 | 9.86295659519886e-05 | 0.000102096119773093 | 0.000167010045114473 |
| pos\_1149 | (S)-p-Mentha-1,8-dien-10-yl acetate | 2.67649475323759e-05 | 2.8043185333518e-05 | 8.48594187551465e-06 | 1.23069515301275e-05 | 1.81491007307866e-05 | 1.14505992239769e-05 | 1.46363256260179e-05 | 1.44242793184093e-05 | 1.33265414602857e-05 | 1.01618175905275e-05 | 1.25482840767014e-05 | 1.65558279172854e-05 |
| pos\_115 | PA(P-16:0/14:0) | 0.00506188740020014 | 0.00536967912439326 | 0.00406019796121629 | 0.00530592904020369 | 0.00493869805917119 | 0.00497134095145947 | 0.00550036010334791 | 0.00483675853494804 | 0.00569455205229251 | 0.00517424344022865 | 0.00524502520519983 | 0.00414121041665525 |
| pos\_1150 | 1-Acetoxy-2-hydroxy-16-heptadecen-4-one | 0.000207613115783886 | 0.000185180540263676 | 0.000240533712572556 | 0.000255722475878777 | 0.000151575413079437 | 0.000214015643375558 | 0.000280175496118886 | 0.000278484596259226 | 0.000268864722093845 | 0.000184649679281645 | 0.000267203344635357 | 0.000313248453386198 |
| pos\_1151 | Phenylethyl primeveroside | 4.70068569405083e-06 | 6.53219214955791e-06 | 1.79883462074518e-05 | 1.04757410864096e-05 | 6.76348696763214e-06 | 6.59214754729847e-06 | 6.14750064857477e-06 | 1.00631988580853e-05 | 5.83772009783282e-06 | 6.84443891330851e-06 | 6.81660772171375e-06 | 9.91840365328589e-06 |
| pos\_1153 | 4-Hydroxydebrisoquine | 2.18528060986624e-05 | 5.45436881692451e-06 | 0 | 2.90987740056424e-07 | 1.64633062837045e-06 | 5.05294929951965e-06 | 4.04306628669005e-06 | 1.1735699118844e-06 | 0 | 0 | 0 | 0 |
| pos\_1155 | 5b-Cholestane-3a,7a,12a,23S,25-pentol | 6.41637638040231e-05 | 9.49540066476692e-05 | 5.79661265875022e-05 | 9.23141099584598e-05 | 5.81576886457793e-05 | 7.61861389172702e-05 | 9.66554160328032e-05 | 7.93749891437825e-05 | 7.15845012550829e-05 | 9.91737868011721e-05 | 8.62579961548542e-05 | 6.98309031909511e-05 |
| pos\_1156 | 1-Hydroxyacorenone | 7.57635131255473e-07 | 3.7721006870286e-07 | 2.303363839234e-06 | 2.57409455984561e-06 | 2.71229015489524e-06 | 1.34339108536546e-06 | 2.13960544094797e-06 | 1.73335960087176e-06 | 1.30777119548321e-06 | 1.8942153058477e-06 | 1.91540687131701e-06 | 1.9518390898796e-06 |
| pos\_1157 | Naematolone | 0.000441134243019972 | 0.000661633500709085 | 0.000316031454755349 | 0.000506855039887657 | 0.000592816494814073 | 0.000470871235824212 | 0.00049127291294457 | 0.000385759024601094 | 0.000523410850766736 | 0.000503201263972108 | 0.000441354384224113 | 0.000344922253205908 |
| pos\_1158 | Withanolide A | 0.00167354398457423 | 0.00170610866701526 | 0.00172242064773414 | 0.0016415314575875 | 0.00151676998908912 | 0.00174186385580735 | 0.0012472056205587 | 0.00159483573109679 | 0.00185589968652304 | 0.00141692985722118 | 0.00161095370815057 | 0.00184176502157113 |
| pos\_1159 | DG(18:1n7/0:0/18:2n6) | 0.00280774946773264 | 0.00320313011525535 | 0.00496570074758182 | 0.00226699347917109 | 0.00188405874891731 | 0.00286067585539471 | 0.00143027441120738 | 0.00321670608353567 | 0.0027899642333211 | 0.00121733153612624 | 0.00280803961239483 | 0.00596769047487424 |
| pos\_116 | Isobutyl 2-furanpropionate | 3.46106764447667e-05 | 3.43305429033652e-05 | 1.88028214463842e-05 | 3.45922918430992e-05 | 2.33561139709314e-05 | 2.67624397873465e-05 | 3.15861659630621e-05 | 3.20805753371542e-05 | 1.60144873594596e-05 | 4.87858759702224e-05 | 2.83737606502682e-05 | 2.39468904701266e-05 |
| pos\_1160 | Threoninyl-Glycine | 0.000487231045954051 | 0.000507813274749488 | 0.000173158597197415 | 0.000400538020043473 | 0.000249847994954624 | 0.000297753021899771 | 0.000241717017345679 | 0.000214434924352907 | 0.000143518336416612 | 0.000472881090668238 | 0.000256270912919329 | 0.000186320053002659 |
| pos\_1161 | PS(18:0/18:3(9Z,12Z,15Z)) | 0.000334731008373767 | 0.000126364365919649 | 0.000113803236067314 | 0.000109484399267175 | 0.000158793011226878 | 0.000148430618618313 | 0.000178243671836602 | 0.000141546586336688 | 0.000130171629492069 | 0.000130786806833907 | 0.000139031145452527 | 0.000134985136835462 |
| pos\_1162 | 3-Hydroxy-1-phenyl-1-eicosanone | 0.00016022154882097 | 0.000197319688761785 | 0.000156249457871854 | 0.000166298727663172 | 0.000177772311582501 | 0.000166845769563076 | 0.000211251558892516 | 0.000184778116373585 | 0.000240291484580454 | 0.000143066796086331 | 0.000188658447466419 | 0.00012449608555896 |
| pos\_1163 | Meprobamate | 4.0330391202795e-05 | 4.57508328991549e-05 | 7.09559302630991e-05 | 6.24256012837863e-05 | 4.50187907730334e-05 | 5.34900303905838e-05 | 4.85884477477697e-05 | 5.37409279564555e-05 | 4.76990073017062e-05 | 4.32354813197675e-05 | 5.21823273738139e-05 | 5.99287901637545e-05 |
| pos\_1164 | 4-Hydroxyretinoic acid glucuronide | 0 | 0 | 0 | 9.38222855853046e-06 | 0 | 0 | 0 | 2.54810904074486e-06 | 0 | 0 | 0 | 2.11442330509771e-05 |
| pos\_1165 | Estradiol | 9.72910895784071e-06 | 1.42217739365219e-05 | 1.6074157687488e-05 | 1.42935051796849e-05 | 1.05058109237541e-05 | 1.27157765188178e-05 | 1.0281122720025e-05 | 1.44283852437373e-05 | 1.34545587987123e-05 | 1.18322987420619e-05 | 1.48127799042924e-05 | 2.05088469744169e-05 |
| pos\_1166 | Neogrifolin | 1.54523656377695e-05 | 2.30353321215552e-05 | 1.93920862846525e-05 | 1.85197432520951e-05 | 2.30376297396652e-05 | 2.14101906417835e-05 | 2.08824165718144e-05 | 2.08019215723215e-05 | 5.7438009301598e-05 | 2.25539544085542e-05 | 3.08533291292342e-05 | 2.23292057192543e-05 |
| pos\_1167 | Estriol-16-Glucuronide | 0.000107331036752208 | 0.000131781555058187 | 0.000117861596532799 | 0.000119799557457828 | 0.000100130169239552 | 9.47796740869775e-05 | 0.000103147160278868 | 0.000114601138512599 | 0.000113038977622427 | 0.000100809889606698 | 0.000113299447059447 | 0.000122889704269997 |
| pos\_1168 | PA(16:0/18:2(9Z,12Z)) | 0.000963512694833286 | 0.00100351216732148 | 0.000779589658603413 | 0.000687125002357326 | 0.000713153165304309 | 0.000765165104175288 | 0.000655533212288351 | 0.00072020720581387 | 0.000876762130938084 | 0.000713894341443796 | 0.000741441187960727 | 0.000704735400805731 |
| pos\_1169 | Testosterone | 3.52334022937249e-05 | 3.74662998881151e-05 | 0.000118525344119826 | 2.61448164987287e-05 | 9.02929485547147e-05 | 5.38055243073749e-05 | 2.95488721811823e-05 | 3.74731015696618e-05 | 2.49600123750229e-05 | 0.000139376879512194 | 5.03456419622985e-05 | 6.61926775162656e-05 |
| pos\_117 | 4'-METHOXYCHALCONE | 0.000319224355702004 | 0.000500199869520371 | 0.000250335235302967 | 0.000411703784645202 | 0.000396359370701674 | 0.000352159472519696 | 0.000375843887734663 | 0.000332756342991808 | 0.000438827696772404 | 0.000376745691050184 | 0.000359538603605168 | 0.000251030498066759 |
| pos\_1170 | 27-Nor-5b-cholestane-3a,7a,12a,24,25-pentol | 0.000171956897011215 | 0.000224558725044886 | 0.000280856172480143 | 0.000286387735323078 | 0.000179020414986807 | 0.000230103276014475 | 0.000268892118271439 | 0.000261846266401785 | 0.000199464139574081 | 0.000213131043437795 | 0.00024010197996104 | 0.0002440726672036 |
| pos\_1171 | (all-Z)-8,11,14-Heptadecatrienal | 5.01115788628649e-06 | 5.3193014876712e-06 | 7.05906392484538e-06 | 8.53223326568722e-06 | 5.82932272762863e-06 | 6.43541383145748e-06 | 6.5855495334698e-06 | 1.0151030130447e-05 | 7.5998279758267e-06 | 4.72660452595878e-06 | 6.46026809507901e-06 | 9.30280746989828e-06 |
| pos\_1173 | Hypusine | 4.6725233567475e-06 | 4.03124956104254e-06 | 4.34089734338993e-06 | 4.88349552466205e-06 | 3.13382266253474e-06 | 3.99135745086751e-06 | 4.13212960573114e-06 | 3.83261952349001e-06 | 6.14087552629792e-06 | 4.21282328819459e-06 | 4.18313821267656e-06 | 4.90190422908859e-06 |
| pos\_1174 | Shyobunyl acetate | 5.15321245560088e-05 | 5.77487442971125e-05 | 3.11544653659706e-05 | 2.93919591378517e-05 | 4.4500774628491e-05 | 3.44486126936039e-05 | 3.73109741983188e-05 | 2.77542519474266e-05 | 3.57716059074474e-05 | 3.80702370807094e-05 | 2.92121863400397e-05 | 2.30445573178113e-05 |
| pos\_1176 | (all-E)-3,5,7-Tridecatriene-9,11-diyn-1-ol | 6.66875805786219e-06 | 7.44954550190186e-06 | 8.63624924448722e-06 | 9.88825141955328e-06 | 8.2494085322093e-06 | 6.28050202393656e-06 | 1.31304190694113e-05 | 9.35110685086418e-06 | 9.11592246827236e-06 | 9.80945721714828e-06 | 9.38974836625896e-06 | 8.00716960728467e-06 |
| pos\_1177 | p-HPEA-EDA | 5.64328849183e-05 | 8.80198132548607e-05 | 3.51379079239619e-05 | 6.58382242530094e-05 | 7.79457981781084e-05 | 6.08135129909972e-05 | 4.78710808460535e-05 | 4.28211018465857e-05 | 7.8290253772601e-05 | 5.725663601693e-05 | 5.70419004385635e-05 | 4.24449523073203e-05 |
| pos\_1178 | N(6)-(Octanoyl)lysine | 6.45386550613315e-06 | 1.28926497953856e-05 | 2.28749387987893e-06 | 7.25729573055605e-06 | 1.09200658830872e-05 | 7.27901679518629e-06 | 7.02222957314404e-06 | 4.85745675383886e-06 | 7.9762084661753e-06 | 6.24227986096052e-06 | 6.65840794369298e-06 | 4.39837109569169e-06 |
| pos\_1179 | Lippioside I | 1.95033602417343e-05 | 7.37538413463455e-05 | 2.27409180382226e-05 | 1.73914194839274e-05 | 5.37996668210903e-05 | 2.9456582670697e-05 | 2.62925735688172e-05 | 2.13501452315037e-05 | 1.6158577987881e-05 | 5.56373898996237e-05 | 2.54468697752923e-05 | 2.36545107024681e-05 |
| pos\_118 | Oxindole | 0.000130709892398887 | 0.000308579475960313 | 0.000210894466875224 | 0.000259220620083501 | 0.000223465762720542 | 0.000230581417960194 | 0.000249136475925548 | 0.000189156860074903 | 0.000320615130899104 | 0.000169249936075886 | 0.000270073359849918 | 0.000182440355888007 |
| pos\_1180 | Ustiloxin B | 0.000326569621825361 | 0.000320636273840527 | 3.22435747774862e-07 | 0.000112274177783131 | 0.000376152762860401 | 0.000211077003869773 | 8.73226451032093e-07 | 2.672021042301e-07 | 1.41789851109209e-06 | 0.000134511448926682 | 1.23058559592231e-05 | 8.35321758639335e-07 |
| pos\_1181 | 4-Chlorocatechol | 6.38540997188843e-06 | 8.69797085797087e-06 | 8.65570836067598e-06 | 8.52294538719693e-06 | 4.75608511426741e-06 | 6.14793079171506e-06 | 6.07690300366647e-06 | 5.83215717024448e-06 | 7.93923238371409e-06 | 6.21727125392358e-06 | 6.67991868069516e-06 | 6.79606276683937e-06 |
| pos\_1182 | Methyl 1-(1-propenylthio)propyl disulfide | 2.77438995365808e-05 | 3.25796969452639e-05 | 2.74541075018535e-05 | 2.24844143861157e-05 | 1.69917414841016e-05 | 1.43306927991166e-05 | 1.70329367851055e-05 | 1.4954831019804e-05 | 2.24233712535002e-05 | 1.70308439652936e-05 | 1.40731768496414e-05 | 1.63494054119529e-05 |
| pos\_1184 | 3,5,7-Trihydroxy-4',6-dimethoxyflavanone | 1.13147678862185e-05 | 1.57748100882483e-05 | 2.04048680462709e-05 | 1.53043780059665e-05 | 9.11228236566148e-06 | 1.59259234009478e-05 | 1.52227353247831e-05 | 1.37694732615305e-05 | 1.53986802890716e-05 | 1.50589009258112e-05 | 1.55297888975934e-05 | 1.35170953551426e-05 |
| pos\_1185 | (S,E)-Zearalenone | 1.17628972188077e-05 | 1.92279821489656e-05 | 7.20876206235966e-06 | 1.14806579224712e-05 | 1.18727614289545e-05 | 1.0376791696216e-05 | 1.08712267400451e-05 | 9.59396101548244e-06 | 1.42061362744709e-05 | 1.67203906021446e-05 | 1.27584214581746e-05 | 1.18838231391143e-05 |
| pos\_1186 | 4-Coumaroyl-2-hydroxyputrescine | 1.00344915669112e-06 | 2.63674617260979e-06 | 6.22450561546419e-06 | 6.27908554981337e-07 | 1.30758658211719e-06 | 2.34725421440534e-06 | 2.94223909325942e-07 | 2.38639173595499e-06 | 1.00435315318265e-07 | 9.78752525127604e-06 | 2.54187670002325e-06 | 9.23976695593568e-06 |
| pos\_1187 | Rutalinium | 0.000209110797246378 | 0.00023994826332225 | 0.000228895965686212 | 0.000251404330226527 | 0.000206351446990471 | 0.000341816885626828 | 0.000233039266567345 | 0.000273994051316073 | 0.000208454926273643 | 0.000281855018191014 | 0.000297194034140286 | 0.000213472305723146 |
| pos\_1188 | N,O-Didesmethyltramadol | 5.05307675124353e-05 | 5.05385497845603e-05 | 4.47738218199831e-05 | 4.6064129759449e-05 | 4.62093873094232e-05 | 4.72180022372575e-05 | 4.90635570364796e-05 | 4.45403396135036e-05 | 4.16330713943557e-05 | 4.55929806076963e-05 | 4.52721231546013e-05 | 4.00798112833955e-05 |
| pos\_1189 | 3-Hydroxy-1-phenyl-1-heptadecanone | 1.43709706159502e-05 | 2.99164123466321e-05 | 1.8655299851253e-07 | 7.79187537300014e-06 | 6.90046832370286e-06 | 8.28628871433116e-06 | 1.63979051229e-05 | 1.26588388217618e-05 | 1.16578047795421e-05 | 3.67745873409417e-05 | 1.50433487612291e-05 | 6.16439834750084e-06 |
| pos\_119 | 3,4-Dihydrocadalene | 0.000172409947216234 | 0.000218346483538432 | 0.000213456325755737 | 0.000205149213162387 | 0.000166700000050388 | 0.000191979470120804 | 0.000182252058374789 | 0.000209132322269042 | 0.000189263163250823 | 0.000205295982412063 | 0.000203765423415896 | 0.000214618573468933 |
| pos\_1190 | Isopersin | 9.57324346709939e-05 | 0.000133461397572635 | 0.000193619274485094 | 0.000166142525401075 | 0.000108525518552379 | 0.000152496727479017 | 0.000103005353129861 | 0.000144251672416413 | 0.000108879503595463 | 0.000106423070163992 | 0.000138590192841285 | 0.000216237920605999 |
| pos\_1191 | 4alpha-Carboxy-5alpha-cholesta-8-en-3beta-ol | 8.81785420039309e-07 | 1.03716539760797e-07 | 0 | 3.87049556188928e-05 | 2.49318155527821e-07 | 6.20085336290039e-06 | 1.37063108238134e-07 | 3.83447972477814e-08 | 6.45426134157477e-07 | 3.3606831067074e-05 | 4.12452507045952e-06 | 7.18416541189985e-08 |
| pos\_1192 | Phenazopyridine | 1.75416392364577e-06 | 1.65518518203319e-06 | 6.77295444204834e-06 | 1.43413837309896e-05 | 3.79053709347649e-06 | 4.92323055837557e-06 | 2.4996315331831e-06 | 5.64335818124763e-06 | 1.38705541171489e-06 | 4.9162578972551e-06 | 5.62579401867548e-06 | 1.38089649954442e-05 |
| pos\_1193 | Glabrene | 1.25755117577039e-05 | 4.06400340502843e-07 | 6.56302637897225e-06 | 7.28933145519653e-07 | 9.75408685702034e-06 | 4.09114880872701e-06 | 1.28377994319121e-05 | 8.48222617949732e-06 | 9.17425573205258e-06 | 3.63963447847239e-06 | 1.19606263383936e-05 | 1.98381796981374e-06 |
| pos\_1194 | ADP-ribose 1"-2" cyclic phosphate | 9.47372036539313e-05 | 2.09013595582538e-05 | 2.33542062997844e-05 | 1.91717627562723e-05 | 4.90033873844317e-05 | 5.05083582002249e-05 | 4.82276989306768e-05 | 3.89726485320359e-05 | 2.87549541575747e-05 | 3.44207169622916e-05 | 3.75874967197057e-05 | 3.65917280815506e-05 |
| pos\_1195 | 3-Succinoylpyridine | 8.00925281493265e-05 | 0.0001067172431558 | 3.92127875513909e-05 | 6.54323086112259e-05 | 8.14291686934893e-05 | 6.93814690118712e-05 | 5.23491349460536e-05 | 4.49385213106939e-05 | 7.70511486578844e-05 | 6.9921780486347e-05 | 6.31206987376127e-05 | 4.17883327700698e-05 |
| pos\_1196 | Cytochalasin Npho | 0.00406042275329915 | 0.00157601417443791 | 0.00139526583965853 | 0.00127620455586174 | 0.00260934140790528 | 0.0026356874584959 | 0.00277889593437475 | 0.00206120868087667 | 0.00190879026492888 | 0.00201911150101793 | 0.00210499278755635 | 0.00208677787088566 |
| pos\_1197 | 2-Stearyl citrate | 0.0106368884850162 | 0.0105518052526958 | 0.0106950825080261 | 0.00932097206661127 | 0.00857811032230362 | 0.0101455526405621 | 0.0095803801950581 | 0.0101783383448625 | 0.0103758643356988 | 0.00906968688086284 | 0.00990403147157265 | 0.00970424927119713 |
| pos\_1198 | all-trans-3,4-Didehydroretinoate | 0.000342272827744288 | 0.00024376518502065 | 0.000912872532294292 | 4.81850012599377e-05 | 0.000242465521814707 | 0.000308732795691025 | 2.04362796491063e-05 | 0.000192720483159707 | 3.78574891814208e-05 | 0.00129861022025365 | 0.000271073742372919 | 0.000520793153117441 |
| pos\_1199 | Antibiotic GR 95647X | 6.25878855365323e-05 | 5.31058372403036e-05 | 5.64170580083998e-05 | 4.6141516747142e-05 | 4.96084790559978e-05 | 5.42254927266013e-05 | 5.56158816511819e-05 | 4.9690102763663e-05 | 5.61910413396207e-05 | 5.27975018922765e-05 | 5.10688115475977e-05 | 5.16844207757809e-05 |
| pos\_12 | Hypoxanthine | 0.000371812854488535 | 0.000480574393653544 | 0.000353765727995651 | 0.000306175872046285 | 0.000503559273685996 | 0.000310580700323051 | 0.000378543400659467 | 0.000435501006544614 | 0.000386280193092769 | 0.00061293130214648 | 0.00035793589936024 | 0.000546282514999064 |
| pos\_120 | BETAMIPRON | 0.00057658384549959 | 0.000332279293012875 | 9.98167349937268e-05 | 0.000248114843348025 | 0.000324707344172667 | 0.000253069277176471 | 0.000162963529955938 | 0.000123274947594184 | 0.000123212390438883 | 0.000215840701637864 | 0.000157430254546731 | 8.31983989788877e-05 |
| pos\_1200 | Flunarizine | 0.000112454196172507 | 0.000143897676090168 | 3.24825321289872e-05 | 0.000116206217063481 | 0.00010346045139021 | 0.000101631029279653 | 9.96674156560633e-05 | 7.39741329467523e-05 | 5.77624317846522e-05 | 0.000115717016977754 | 8.06384090298102e-05 | 9.42791056991674e-05 |
| pos\_1201 | TG(8:0/8:0/i-17:0) | 2.49406551559252e-05 | 1.16105382223053e-06 | 1.36651848285784e-06 | 8.6627879199415e-07 | 9.83263340990301e-06 | 8.12634167804689e-06 | 1.14496850704128e-05 | 8.89919146711319e-06 | 2.1251823969744e-06 | 1.8140348010319e-06 | 5.05142442534705e-06 | 5.43970853663058e-06 |
| pos\_1202 | Musanolone D | 4.58651798769956e-05 | 6.43882268553837e-05 | 6.19882652972679e-05 | 5.43414876849152e-05 | 4.59770147057022e-05 | 5.08335504653149e-05 | 5.51398458625842e-05 | 5.39381544496468e-05 | 6.17879773022642e-05 | 4.41009999140548e-05 | 5.28947931271143e-05 | 5.47872466502316e-05 |
| pos\_1203 | 2,2,4-Trimethyl-1,3-pentadienol diisobutyrate | 2.11339601041437e-05 | 2.47872019802938e-05 | 2.64119332214224e-05 | 2.05151027219585e-05 | 1.90025810602068e-05 | 2.23510862224258e-05 | 1.80913438146229e-05 | 2.17193507340687e-05 | 1.96867786161383e-05 | 2.61026812283933e-05 | 2.11896698499266e-05 | 2.17461099646628e-05 |
| pos\_1204 | Androsterone | 1.72244956987785e-05 | 1.77801948055602e-05 | 1.81616907916737e-05 | 1.71872965971099e-05 | 1.46376610766885e-05 | 1.7658859978177e-05 | 1.69863165196673e-05 | 1.76414911637018e-05 | 1.59793806380109e-05 | 2.00138942751765e-05 | 1.82490370048613e-05 | 1.70243635171296e-05 |
| pos\_1206 | 2-hydroxymethylolanzapine | 7.27064369007252e-06 | 1.42939610549604e-05 | 1.24884891290246e-05 | 1.17328990151361e-05 | 8.9749711747386e-06 | 1.04669160986437e-05 | 9.66996525850471e-06 | 1.17467942485928e-05 | 1.22896722389786e-05 | 1.04929347351666e-05 | 1.12422895067308e-05 | 1.01098258739133e-05 |
| pos\_1207 | PC(18:4(6Z,9Z,12Z,15Z)/22:6(4Z,7Z,10Z,13Z,16Z,19Z)) | 0.0378001120221765 | 0.0164226100707242 | 0.0159910134296621 | 0.0139827933719229 | 0.0188551984654582 | 0.0198098868938308 | 0.020713899892171 | 0.0180980567695413 | 0.0185195088653322 | 0.0165624036232202 | 0.0178380463850451 | 0.0176888317465008 |
| pos\_1208 | Cucurbitacin I 2-glucoside | 8.11568059747142e-06 | 3.82331506179012e-06 | 3.28052832232762e-06 | 1.52479123979369e-06 | 5.02321649782053e-06 | 4.81251205191058e-06 | 6.43588399893152e-06 | 4.59287671018415e-06 | 5.0220265463621e-06 | 3.98641552458347e-06 | 5.02746874715059e-06 | 4.78667827729612e-06 |
| pos\_1209 | (1S,2R,4R,8S)-p-Menthane-2,8,9-triol 9-glucoside | 0 | 0 | 0 | 3.75332484477294e-06 | 0 | 2.65766938136049e-07 | 2.43383066350026e-06 | 1.277622722622e-06 | 0 | 2.11825078987818e-05 | 2.30164018934591e-06 | 0 |
| pos\_1210 | Alloxanthin | 0.0239125187953439 | 0.0405514462783118 | 0.0270830576579331 | 0.0273565230349164 | 0.0355509159582051 | 0.0305591326157406 | 0.0223420201161471 | 0.0272499275170894 | 0.0292650871941349 | 0.0356308210430733 | 0.029650640811362 | 0.0278621244798936 |
| pos\_1211 | (3E,5E)-2,6-Dimethyl-1,3,5,7-octatetraene | 0.000174104116468933 | 0.00018838323546873 | 0.000263373137876945 | 0.000148265007912193 | 0.000182920841111364 | 0.000180727647742911 | 0.000130210238128672 | 0.00016851742195176 | 0.000140426444223453 | 0.000284654561680296 | 0.000176789562073087 | 0.000226592456305404 |
| pos\_1212 | Arachidonoyl Serinol | 0.000974146211597171 | 0.000897064246290581 | 0.0007955980938466 | 0.00105349856170596 | 0.000729536475570372 | 0.00087132241235236 | 0.000953318817376734 | 0.000872557944304068 | 0.000634693927605113 | 0.000967605372137651 | 0.00085394081881542 | 0.000796117564478134 |
| pos\_1213 | Phytosulfokine a | 6.65758865645494e-05 | 7.55310277230213e-06 | 1.09730462532556e-05 | 7.58957807217e-06 | 2.7035266764841e-05 | 3.20194893393443e-05 | 3.19235002208992e-05 | 1.85366289893347e-05 | 9.76800080897671e-06 | 1.69228323206997e-05 | 1.41911726151981e-05 | 2.09806494500525e-05 |
| pos\_1214 | Methyl (R)-9-hydroxy-10-undecene-5,7-diynoate glucoside | 0.000173130060294408 | 0.000218991172164207 | 9.0619762672661e-05 | 0.000162022083114365 | 0.000158496245686544 | 0.000149530729216108 | 0.000192661565698242 | 0.000153493436200468 | 0.00014649869225184 | 0.000175564771023193 | 0.000160053877145915 | 0.000137084353207465 |
| pos\_1215 | O-Ureidohomoserine | 2.74519273968644e-05 | 7.04107748450474e-05 | 2.84497431000411e-05 | 3.70599448406702e-05 | 5.38278588346377e-05 | 4.15665899158852e-05 | 3.8332004161351e-05 | 3.42311612210816e-05 | 6.25842827402732e-05 | 3.75165500771201e-05 | 4.07142522472441e-05 | 2.87006512446362e-05 |
| pos\_1216 | 3-Allyl-1-cyclohexene | 1.03229778062465e-05 | 1.27569989535509e-05 | 2.21793283612695e-05 | 7.5466516699437e-06 | 1.18876614731386e-05 | 1.37762793985493e-05 | 5.63351898581675e-06 | 1.21198431968689e-05 | 5.84035204632501e-06 | 3.78569810483843e-05 | 1.22161202863824e-05 | 2.22299291596516e-05 |
| pos\_1217 | 3,5,5-Trimethyl-2-cyclohexen-1-one | 9.06395076807128e-06 | 1.29118750872189e-05 | 1.1952858368598e-05 | 9.92857915432556e-06 | 7.60104666030503e-06 | 9.03246116885924e-06 | 9.07406107505502e-06 | 1.07364661622379e-05 | 1.10881490077738e-05 | 8.94566019761052e-06 | 9.61923895050197e-06 | 9.34733912384253e-06 |
| pos\_1218 | Cholesta-4,6-dien-3-one | 0.0013244722464755 | 0.00101217075441263 | 0.0030907150975056 | 0.00344519819936599 | 0.00155186871121292 | 0.00211085677247863 | 0.00278587040486815 | 0.00300522327453124 | 0.00157508736788115 | 0.00186318225228101 | 0.00263259129534245 | 0.00326796412218832 |
| pos\_1219 | 2,4,7-Decatrienoic acid | 2.81979153729803e-05 | 3.33497938078675e-05 | 2.74329525883606e-05 | 3.18827628577893e-05 | 2.51890096943184e-05 | 2.86816528686418e-05 | 2.93098359927693e-05 | 2.80298111615228e-05 | 2.97578288067171e-05 | 2.80732762260975e-05 | 2.83205081829568e-05 | 2.7547815663624e-05 |
| pos\_122 | 3α,12α-Dihydroxy-5β-chola-7,14-dien-24-oic Acid | 0.000744432693421582 | 0.00139284137338049 | 0.000722670538416346 | 0.000995175229361018 | 0.000849109847668764 | 0.000633091498444832 | 0.00225033079697748 | 0.00260738071814385 | 0.000779218126890647 | 0.000844113838468565 | 0.000722707450698383 | 0.000538536275679211 |
| pos\_1220 | Vignatic acid A | 9.50233074890048e-05 | 8.01644476988885e-05 | 7.96064822127622e-05 | 6.58081309963895e-05 | 6.8175518591291e-05 | 8.06016540686159e-05 | 7.8214088094193e-05 | 8.1280591453114e-05 | 8.19658227193692e-05 | 7.50767370477914e-05 | 7.89732302249451e-05 | 7.31707755972831e-05 |
| pos\_1221 | Lucuminoside | 1.01553565482658e-05 | 1.53394458821668e-05 | 6.15877895837802e-06 | 2.86560387778068e-05 | 1.3040520144071e-05 | 1.51902160413273e-05 | 4.02188171482553e-06 | 9.81160848460546e-06 | 4.44356795723264e-05 | 1.44792622841412e-05 | 1.61918532730549e-05 | 1.50952640470213e-05 |
| pos\_1224 | Dimethyl fukiic acid | 5.27584751086724e-05 | 8.67553092461363e-05 | 0.000179171679732794 | 5.33862509010606e-05 | 5.49310325729846e-05 | 8.03897269121016e-05 | 5.08409899363779e-05 | 0.000259250259446479 | 0.000253270141258379 | 6.49422172520781e-05 | 0.000124165948086115 | 8.06833479466078e-05 |
| pos\_1225 | Tiapride | 3.78911852012375e-05 | 3.55184979874749e-05 | 1.63597721319554e-05 | 3.02066592455512e-05 | 2.41401546664236e-05 | 2.68081211699761e-05 | 2.23310982261632e-05 | 1.98454065279401e-05 | 1.98482433498423e-05 | 3.83834962538582e-05 | 2.4988203840938e-05 | 1.89258918608982e-05 |
| pos\_1226 | 13-Demethyl tacrolimus | 0.000202744528461997 | 5.30335711597023e-05 | 4.41139432231638e-05 | 4.29135181213593e-05 | 0.000113511631796722 | 0.000106607764333562 | 9.97647295786356e-05 | 8.38144433249028e-05 | 6.27210466527866e-05 | 7.28238024527418e-05 | 7.90986978772711e-05 | 7.20415973555153e-05 |
| pos\_1227 | Pollenin B | 1.06856288332028e-05 | 1.71666484385848e-06 | 2.61211180807269e-06 | 8.55877555583779e-07 | 5.77012854597569e-06 | 5.26667509018376e-06 | 4.94650955083144e-06 | 3.87507241039423e-06 | 2.47527174518631e-06 | 3.2821512736514e-06 | 3.98962960217833e-06 | 4.45883495507905e-06 |
| pos\_1229 | N-di-Demethyl roxithromycin | 0.00011626163242678 | 8.96932270458001e-05 | 8.28434829739223e-05 | 0.000159788714577956 | 7.94082742365563e-05 | 0.000111318842078631 | 9.29557447745144e-05 | 9.50736826332742e-05 | 7.96341443515625e-05 | 8.70293348552678e-05 | 8.95858180071673e-05 | 8.79386341226224e-05 |
| pos\_123 | 6,10,14-Trimethyl-5,9,13-pentadecatrien-2-one | 0.000190584419882656 | 0.000159050504757414 | 0.000182537599705521 | 0.000190368100470689 | 0.00020454182101673 | 0.000184398375635752 | 0.000175888180242607 | 0.000187204361390829 | 0.000215858044604718 | 0.000160739511055567 | 0.000204973018838231 | 0.000188679522573335 |
| pos\_1230 | gamma-Chaconine | 0.000544332669163716 | 0.00048888523869062 | 0.00123449028547808 | 0.000833169182637674 | 0.000731794950336345 | 0.000711933429856529 | 0.000807697844768544 | 0.000955490214295763 | 0.000495654859072989 | 0.000679472192115623 | 0.00075435746246056 | 0.0011473890281302 |
| pos\_1231 | 12-Oxo-2,3-dinor-10,15-phytodienoic acid | 0.000220156950249646 | 0.000398043036262886 | 0.000416473022537625 | 0.000386962631947701 | 0.00025290209752821 | 0.000330516098883709 | 0.000268364909778666 | 0.000321418311142168 | 0.000320091196143625 | 0.000294081695065719 | 0.000326569134706138 | 0.000378037478442783 |
| pos\_1232 | Quinoside A | 0.000322283979409195 | 0.000294701936119664 | 0.000355528523281453 | 0.000317141597524994 | 0.000200430616309999 | 0.000309679760145413 | 0.000170442562183947 | 0.000271309761480326 | 0.000236198165949944 | 0.000434100889349508 | 0.000299512882663672 | 0.000508900733877194 |
| pos\_1233 | Ganglioside GM3 (d18:0/12:0) | 5.28336628936216e-06 | 1.33146093109919e-05 | 1.63112533106235e-05 | 5.35495370896571e-06 | 7.9214646979517e-06 | 6.94377845767474e-06 | 8.31791640188218e-06 | 1.25314541666141e-05 | 6.18527830170169e-06 | 1.05352337359554e-06 | 7.03260740957289e-06 | 1.77978414849392e-05 |
| pos\_1234 | Varanic acid | 1.73854149123221e-05 | 1.19095146915479e-05 | 9.52028845998836e-06 | 5.21041799458749e-06 | 5.79391805090507e-06 | 8.73075507730343e-06 | 1.14667854592764e-05 | 6.61069281763903e-05 | 1.00965430419372e-05 | 1.04807385877206e-05 | 3.89463068118819e-05 | 0.000112365518768835 |
| pos\_1235 | Cotinine glucuronide | 1.48589202007149e-05 | 2.51138674708265e-05 | 5.19243094708861e-06 | 6.59816951931783e-06 | 2.99263188442984e-06 | 2.12948806554525e-06 | 1.07236752037211e-05 | 8.29178257943762e-06 | 5.80004288174907e-06 | 3.84968938332281e-06 | 6.06622203928355e-06 | 3.74240681996325e-06 |
| pos\_1236 | 2-Amino-4-hydroxy-6-pteridinecarboxylic acid | 7.95920540193564e-06 | 8.56464444417682e-06 | 8.91338995645133e-06 | 7.80364086874883e-06 | 8.21813897199378e-06 | 8.501382455938e-06 | 9.27591399812564e-06 | 7.25767990051907e-06 | 8.11135437315039e-06 | 7.49642118966452e-06 | 9.21918174995967e-06 | 6.56191653586186e-06 |
| pos\_1237 | Steviobioside | 0.000393428503509828 | 0.00025214182307183 | 0.000317343727321993 | 0.000102802382292668 | 0.000196254986737796 | 0.00027269653254057 | 0.000187919416181553 | 0.000450486977041453 | 0.000285271270746657 | 0.000156338379220311 | 0.000357088945347356 | 0.000993366671710627 |
| pos\_1238 | Torachrysone 8-(2-apiosylglucoside) | 4.19309673041866e-06 | 3.21051517948193e-05 | 1.83742709214116e-06 | 1.69307114441672e-06 | 3.03516425763923e-06 | 1.2256680007008e-05 | 3.4574583835064e-05 | 1.11726822252722e-05 | 1.24676754001095e-05 | 7.48027749232234e-06 | 1.43071542890101e-05 | 1.22908408500751e-05 |
| pos\_1239 | L-alpha-Amino-1H-pyrrole-1-hexanoic acid | 0.000169233132300719 | 9.19641506547501e-05 | 8.15113326120257e-05 | 8.19472131736676e-05 | 0.000109737777972239 | 0.000113494722942454 | 0.000121233551072285 | 0.000102509420748998 | 9.64479420710925e-05 | 8.97231374633736e-05 | 0.000100172514318797 | 0.000103303048265132 |
| pos\_124 | Ergosta-5,7,22,24(28)-tetraen-3β-ol | 0.000944136509828013 | 0.00100425267069513 | 0.000783231133227658 | 0.00118552052240655 | 0.00102718376732819 | 0.00103434031270918 | 0.000720395503365171 | 0.000968004232060847 | 0.00104673468587717 | 0.000934298921256409 | 0.00104092664270014 | 0.00111229433981143 |
| pos\_1240 | Penicillamine | 3.88967345830555e-06 | 1.33615726966181e-05 | 3.41981749968606e-06 | 1.11241757334634e-05 | 3.21594093460237e-06 | 6.34202507584265e-06 | 2.01979521015085e-05 | 1.09491604923756e-05 | 1.81877034261182e-06 | 6.91936609180835e-05 | 2.40757810065867e-05 | 2.52476504513961e-06 |
| pos\_1241 | Acarbose | 6.40474668878078e-06 | 5.4275043755997e-06 | 1.77381825455426e-06 | 1.23599381832875e-06 | 4.43892150244346e-06 | 3.17792738853481e-06 | 3.97999287191784e-06 | 2.99268812382905e-06 | 2.48061825677569e-06 | 3.057057835594e-06 | 2.45960383250038e-06 | 2.99997305126388e-06 |
| pos\_1242 | Amrinone | 0.000253729221796327 | 0.000304215273023664 | 0.000270527076890444 | 0.000217691027623626 | 0.000343679019204951 | 0.00023504715892137 | 0.000349623906650789 | 0.000317875510526501 | 0.000301414835293382 | 0.000346407012910035 | 0.000277764968648351 | 0.000626951107535811 |
| pos\_1244 | L-Threoneopterin | 5.13335975554487e-05 | 7.05472930812631e-05 | 3.01405139716053e-05 | 4.2648790810515e-05 | 4.55448718442607e-05 | 5.08441724792163e-05 | 4.42019113522735e-05 | 3.53512036245618e-05 | 6.89212176761567e-05 | 6.7455348391231e-05 | 5.05690135460079e-05 | 2.79203489917398e-05 |
| pos\_1246 | beta-D-Galactopyranosyl-(1->4)-2-amino-2-deoxy-beta-D-glucopyranosyl-(1->6)-D-mannose | 2.02898459363018e-06 | 1.24725359196909e-05 | 2.26931379182282e-07 | 1.58372526623925e-06 | 1.61974438810895e-05 | 4.96464669913431e-06 | 0 | 4.0953178913026e-07 | 6.47739268287377e-06 | 5.48207896692395e-06 | 9.91258494617116e-07 | 1.07475362888202e-06 |
| pos\_1247 | PE(18:3(6Z,9Z,12Z)/22:5(4Z,7Z,10Z,13Z,16Z)) | 1.88218697838874e-05 | 1.89444217785478e-05 | 2.28515494341957e-05 | 3.52157024132453e-05 | 9.99833150662132e-06 | 1.59698526925857e-05 | 2.15698022564588e-05 | 2.24014864392668e-05 | 2.390673330215e-05 | 4.58808658390273e-06 | 6.08334434442515e-06 | 7.99450142967156e-06 |
| pos\_1248 | Cerebroside B | 7.27937688743303e-06 | 1.91119367320607e-06 | 2.80738716624481e-06 | 1.07254822091939e-06 | 2.09961928551289e-06 | 5.61763743287234e-06 | 0 | 8.4742868203384e-07 | 1.09353927396022e-06 | 0 | 9.35717321816451e-07 | 2.08240165138295e-06 |
| pos\_1249 | Quinquenoside III | 2.62968983836385e-06 | 4.41150192560898e-06 | 5.63090614321353e-06 | 3.6449387498287e-06 | 4.97797653238683e-06 | 5.72121314580859e-06 | 6.67223761888877e-06 | 5.65853649207721e-06 | 5.45775415226494e-06 | 7.24801579382881e-06 | 8.55070703871328e-06 | 5.15841637454096e-06 |
| pos\_125 | 1-Anilino-9,10-dioxo-2-anthroic acid | 0.000256160134123388 | 4.71827098438621e-05 | 0.000107154097990112 | 6.47918688738566e-05 | 4.99470015802263e-05 | 0.000106359715257182 | 0.000345987530630575 | 0.000121471365390295 | 0.000106020523426411 | 0.000120251929709865 | 0.000167803291459831 | 0.00056741406962414 |
| pos\_1250 | Valyl-Threonine | 1.75440657145956e-05 | 1.58229605070514e-05 | 1.57547045522289e-05 | 2.70927339743539e-05 | 2.08618820619726e-05 | 1.83695402777165e-05 | 2.54804544408265e-05 | 1.91631539647252e-05 | 1.7347378926386e-05 | 2.00871276946789e-05 | 1.98629543647338e-05 | 1.7214925456892e-05 |
| pos\_1251 | Ganoderic acid H | 0.00366670270108889 | 0.00795256350489812 | 0.00235193312388676 | 0.00080558790094079 | 0.00157232206628091 | 0.00297783730389027 | 0.000502131894403779 | 0.00143644885750896 | 0.00185081996234674 | 0.000404693316551636 | 0.00133452765920286 | 0.00265995420577307 |
| pos\_1252 | Docosahexaenoic acid | 1.5328847718536e-05 | 2.26342887299154e-05 | 2.20279358418742e-05 | 2.71127299144234e-05 | 2.78413099175684e-05 | 2.22052798261632e-05 | 2.54963841426652e-05 | 2.6939016057046e-05 | 2.74253794777999e-05 | 2.12312140777076e-05 | 2.2875150758575e-05 | 2.42391509710697e-05 |
| pos\_1253 | Prostaglandin H1 | 0.000169647263078527 | 6.25511942467572e-05 | 3.98161215471729e-05 | 9.55913194388082e-05 | 2.9482717779923e-05 | 7.45147774621749e-05 | 3.64813264297464e-05 | 3.74842382331219e-05 | 4.38547751207406e-05 | 0.000104016943806432 | 4.95499027861068e-05 | 4.27274932208108e-05 |
| pos\_1254 | Dihydrocapsiate | 1.27308079629967e-05 | 1.75937475465662e-05 | 7.77088248667844e-06 | 1.57463879694438e-05 | 8.13701466674423e-06 | 1.17484386679186e-05 | 1.19090704087284e-05 | 9.86866181457923e-06 | 1.34922485078557e-05 | 1.47544616130387e-05 | 1.32764459191864e-05 | 1.05227069330702e-05 |
| pos\_1255 | Graecunin E | 1.91400038761641e-05 | 1.10764153517085e-05 | 1.0168705385975e-05 | 1.9067164606927e-05 | 1.25078357281229e-05 | 1.77587787598511e-05 | 1.62428732483834e-05 | 2.78702079372639e-06 | 7.89777717165462e-06 | 2.61542542847531e-05 | 1.04460961960309e-05 | 3.46135309996147e-06 |
| pos\_1256 | Dehydrophytosphingosine | 3.23917904731263e-05 | 3.62826334965168e-05 | 2.84739918096514e-05 | 3.68161203804869e-05 | 2.27113594420094e-05 | 3.1206626774988e-05 | 3.09063107300321e-05 | 3.22730243258409e-05 | 2.90639985151665e-05 | 2.66352475563798e-05 | 3.39299950177038e-05 | 2.70993092585127e-05 |
| pos\_1257 | PE(20:1(11Z)/22:6(4Z,7Z,10Z,13Z,16Z,19Z)) | 0.00081588957918522 | 0.00107367541780933 | 0.00087665293474652 | 0.00136856786925809 | 0.00101907419198513 | 0.00106571321750132 | 0.000969433401412826 | 0.00092194563835079 | 0.00111326944175967 | 0.00101232113716361 | 0.00104588461741053 | 0.000860326310179975 |
| pos\_126 | 4-Coumaroylshikimate | 3.661009436374e-05 | 4.23581233202253e-05 | 3.77713135933783e-05 | 4.99596228142002e-05 | 8.15010222561057e-05 | 6.14036788680704e-05 | 4.846166514604e-05 | 5.08828422860509e-05 | 6.2774417177857e-05 | 7.534938574164e-05 | 5.23000787907787e-05 | 4.41647389152784e-05 |
| pos\_1260 | Vitamin A2 | 4.60237710142283e-05 | 3.8628921720046e-05 | 4.32311090433325e-05 | 4.12902427147342e-05 | 3.61192064790383e-05 | 3.98669725760947e-05 | 4.13617462310764e-05 | 4.31265979127535e-05 | 3.34444995108296e-05 | 5.88927951278319e-05 | 4.19093954210075e-05 | 4.28120903408603e-05 |
| pos\_1261 | Goldinodox | 7.20879649672561e-05 | 6.75384328557026e-05 | 6.46527214954552e-05 | 5.81546404347082e-05 | 5.24248681060879e-05 | 5.92450594857874e-05 | 5.77162942862406e-05 | 6.35000070100167e-05 | 6.35953373938214e-05 | 5.40231180282244e-05 | 5.78430641177295e-05 | 5.56837157894601e-05 |
| pos\_1264 | 1-Methylhistidine | 7.08923715315909e-06 | 1.22489627101115e-05 | 2.34232010237597e-06 | 6.77830490939731e-06 | 6.5639036220524e-06 | 4.27929417164292e-06 | 6.64960586052129e-06 | 4.38686985335451e-06 | 6.82827278831093e-06 | 3.07660632471831e-06 | 6.25291595546122e-06 | 4.76193966816973e-06 |
| pos\_1265 | 11-Keto-beta-boswellic acid | 0.0273831575505985 | 0.0341560532208161 | 0.0326151415264218 | 0.0281711864656317 | 0.0249425544199016 | 0.0295713171382779 | 0.0264078599026415 | 0.0294020233508656 | 0.0313157670455457 | 0.0271714701039639 | 0.0296890458942431 | 0.0283702881919584 |
| pos\_1266 | N-Methylschinifoline | 0.000204886106694779 | 0.000380105584755001 | 0.000314578995703898 | 0.000174982708211149 | 0.000159505151539248 | 0.000217349770564892 | 0.000198777158643796 | 0.000210802988932367 | 0.000293733312554703 | 0.000403640525728823 | 0.000284875793930853 | 0.000284502880589004 |
| pos\_1267 | L-Histidine | 1.93751603107188e-06 | 2.79186850519182e-06 | 3.44992897085688e-07 | 1.86235703023599e-06 | 1.35273506666863e-06 | 1.44177537932596e-06 | 1.48357559239846e-06 | 3.6806880823238e-07 | 9.22935738076064e-07 | 1.31433319527206e-06 | 1.18166544261477e-06 | 3.48494179041384e-07 |
| pos\_1268 | Chondroitin D-glucuronate | 0.000356204075269974 | 4.7764238476627e-05 | 6.16391443414938e-05 | 4.54494311437554e-05 | 0.000190579970578576 | 0.000176271335100537 | 0.000150480213698157 | 0.000124860235824254 | 7.05654657990971e-05 | 0.000106539391291202 | 0.00012138861774631 | 0.000104461596420196 |
| pos\_127 | Parsonsine | 0.000117916938390634 | 0.000113325431419415 | 0.000145030011238655 | 0.000195749992287513 | 0.000169691645531653 | 0.00019741420336357 | 0.00015427288294963 | 0.00018457185312733 | 0.000130435700537528 | 0.000244646794679133 | 0.000170608791513432 | 0.000168351928750424 |
| pos\_1270 | PC(14:1(9Z)/22:5(4Z,7Z,10Z,13Z,16Z)) | 6.87083562982464e-06 | 6.88064879033784e-06 | 1.18578102874268e-05 | 2.08103996091174e-06 | 6.1357186610487e-06 | 1.77380669725318e-06 | 2.39117913289514e-06 | 0 | 2.27158870131552e-06 | 1.58358658921546e-06 | 7.85798908996056e-07 | 4.35514326331569e-06 |
| pos\_1271 | DG(20:0/0:0/20:2n6) | 0.000900193621821249 | 0.00116745479826781 | 0.000833253996191566 | 0.00122216732120617 | 0.000856755365326855 | 0.000944910345857375 | 0.00107780384434071 | 0.000909827402220409 | 0.000718654222522459 | 0.000997531381329263 | 0.000896447060394754 | 0.000536087716049343 |
| pos\_1272 | Ceramide (d18:1/20:0) | 0.000751036707108483 | 0.00100823049760102 | 0.000784135350974936 | 0.000690693543085367 | 0.000567946060953283 | 0.000519904154029455 | 0.000534891532301538 | 0.000552748751587922 | 0.000709057961681978 | 0.000578532060155003 | 0.000567001771730197 | 0.000566415241551046 |
| pos\_1273 | 20a,22b-Dihydroxycholesterol | 9.57424638271329e-06 | 1.06413847412881e-05 | 1.27695428063535e-05 | 1.71896257814561e-05 | 2.4779527873667e-05 | 1.22797762987137e-05 | 1.79375259340876e-05 | 1.12307510319837e-05 | 2.3117934120808e-05 | 1.60948925241109e-05 | 1.66148210454949e-05 | 8.12460209772071e-06 |
| pos\_1274 | MG(22:1(13Z)/0:0/0:0) | 0.00119747635924779 | 0.000873971346949496 | 0.00113406586125886 | 0.00114223807058921 | 0.000710317443318702 | 0.00103661141687738 | 0.00108420003496155 | 0.00115019017385554 | 0.000748098160269071 | 0.00113999403130631 | 0.00117894969530224 | 0.00123284302226703 |
| pos\_1275 | L-Histidinol | 3.5920692379029e-05 | 4.27930356680697e-05 | 3.73658616619703e-05 | 4.66502916904127e-05 | 3.59764742905705e-05 | 3.9791922746997e-05 | 4.67374721615677e-05 | 4.3733396624098e-05 | 4.01329259775033e-05 | 3.91132912237082e-05 | 4.23080115509129e-05 | 4.15206411646782e-05 |
| pos\_1276 | PE(16:0/22:5(4Z,7Z,10Z,13Z,16Z)) | 0.000285125594691438 | 0.000264595848030734 | 0.000246030934667277 | 0.000221946702853504 | 0.000221865977192165 | 0.000259525604766046 | 0.000244096166113425 | 0.00024388291586211 | 0.000258774635023333 | 0.000236960401366221 | 0.000254707479628878 | 0.000241060963434663 |
| pos\_1277 | 5-Methyl-2,4-bis(3-methyl-2-butenyl)-6-(2-methyl-1-oxopropyl)-5-(4-methyl-3-pentenyl)cyclohexanone | 0.00148649110231964 | 0.000586284274561943 | 0.000797197420942104 | 0.00131050146581341 | 0.000853367572472783 | 0.0010419288821342 | 0.00103441148188206 | 0.000918768669407387 | 0.000869642041822622 | 0.000813043866101831 | 0.000950793483040174 | 0.000943944218627365 |
| pos\_1278 | 3,4-Dimethyl-5-pentyl-2-furanundecanoic acid | 0.00013912864910182 | 0.000241357899995707 | 0.000293001622406208 | 0.000214838685598167 | 0.000284327842172399 | 0.000161981437337455 | 0.00043887199605653 | 0.000415742149348578 | 0.000274902819119204 | 0.000326173860451405 | 0.000225349364937808 | 0.000173076054396913 |
| pos\_1279 | Gemifloxacin | 2.07181025713067e-05 | 2.26451275596956e-05 | 1.26998080314125e-05 | 1.76318431477406e-05 | 1.08790432417491e-05 | 1.87878993547062e-05 | 1.41072414543993e-05 | 1.17176603418483e-05 | 1.25403145694828e-05 | 1.95642575110907e-05 | 1.40351261882824e-05 | 1.25927314247665e-05 |
| pos\_128 | 1α,25-dihydroxy-26,27-dimethyl-20,21-methano-23-oxavitamin D3 / 1α,25-dihydroxy-26,27-dimethyl-20,21-methano-23-oxacholecalciferol | 0.00956558682159917 | 0.0143297336343759 | 0.0102240649876713 | 0.0136819585983459 | 0.0134292719554022 | 0.0121394465314152 | 0.0160254196754113 | 0.0127535159539324 | 0.0133394586205607 | 0.013063516863591 | 0.0125514098279118 | 0.0106102718475036 |
| pos\_1282 | Dolichyl diphosphate | 5.83022182144808e-06 | 9.51563599067805e-06 | 4.71485338449148e-06 | 8.93989725126179e-06 | 7.38134211987054e-06 | 6.90996158831925e-06 | 5.78122065302132e-06 | 6.53494079080158e-06 | 1.43259258857345e-05 | 7.4810495800482e-06 | 1.07290476746594e-05 | 8.04570452808457e-06 |
| pos\_1283 | Phenobarbital | 8.30257452235671e-06 | 1.27933191689965e-05 | 6.37129067048897e-06 | 7.7616990525659e-06 | 1.08669551466249e-05 | 8.12195615639727e-06 | 8.77635691880582e-06 | 6.99779995028148e-06 | 8.52182203612567e-06 | 9.25632710818309e-06 | 8.23523900061851e-06 | 6.88155360595564e-06 |
| pos\_1284 | Dacarbazine | 0.000932394074494164 | 0.000795458295448874 | 0.000740531422106218 | 0.000675413854450315 | 0.000685435962281301 | 0.000754916321404609 | 0.000747979463426558 | 0.000752610016036701 | 0.000788985751756498 | 0.000693785258125828 | 0.000740091995070497 | 0.000723802341021184 |
| pos\_1285 | 1-Cyano-2-hydroxy-3-butene | 1.34209110384786e-06 | 2.10761227848343e-06 | 1.00605860318523e-06 | 1.38570542026231e-06 | 1.31444890643732e-06 | 1.14449977224226e-06 | 1.50426688192594e-06 | 1.28418589184881e-06 | 1.23349923185804e-06 | 1.3509593285567e-06 | 1.36035427288381e-06 | 2.05537806017737e-06 |
| pos\_1286 | L-Urobilinogen | 4.26901354056363e-05 | 1.74527944164384e-05 | 1.20111010406428e-05 | 9.04711991661521e-06 | 2.61039086955695e-05 | 2.42298041878089e-05 | 2.78860717235945e-05 | 2.05436992850005e-05 | 1.83224423560462e-05 | 1.87617864248305e-05 | 1.79847804410333e-05 | 2.0358168331753e-05 |
| pos\_1287 | [8]-Paradol | 3.15819127196942e-05 | 4.1703513761059e-05 | 2.43623133979205e-05 | 3.97516893156749e-05 | 3.94884818077197e-05 | 3.68199555190028e-05 | 3.8204416277576e-05 | 3.05695090883221e-05 | 2.64077377676559e-05 | 3.31674378085728e-05 | 3.5517573779251e-05 | 2.69006246779705e-05 |
| pos\_1288 | Momordicilin | 0.000298983448504834 | 0.000333160194702935 | 0.000922010839103759 | 0.000237711639051813 | 0.000339844487902542 | 0.000415954133962222 | 0.000459809636128674 | 0.000533123964049495 | 0.000316922027553701 | 0.000635647233470722 | 0.000813868840427879 | 0.000562744346580251 |
| pos\_1289 | Licorisoflavan A | 8.66128013940997e-06 | 2.44885693084576e-06 | 1.01773730805491e-05 | 4.36398779334032e-06 | 1.34113334924117e-05 | 4.06298411274431e-06 | 2.00774994787984e-06 | 3.43518893349756e-06 | 2.48344026139341e-06 | 5.96114808376895e-06 | 3.01206995342089e-06 | 5.79197739616079e-06 |
| pos\_129 | Gibberellin A24 | 0.00019973834686524 | 0.000200685094757627 | 0.000853172922397732 | 8.31295217846434e-05 | 0.000272870431932781 | 0.000307933810777474 | 8.43395146213834e-05 | 0.000232642697461796 | 7.75318674444681e-05 | 0.00163517440191459 | 0.000307892729822417 | 0.000532219066130487 |
| pos\_1290 | 4,6-Icosanedione | 1.13243495007456e-05 | 1.3219259260806e-05 | 1.09790979527635e-05 | 1.28848506259087e-05 | 1.15670747941141e-05 | 1.12403039859795e-05 | 1.05285917803596e-05 | 1.08636545029521e-05 | 1.27007573760859e-05 | 1.07766621258677e-05 | 1.08463306424422e-05 | 1.18302098192058e-05 |
| pos\_1291 | 3-Methyl-5-pentyl-2-furanundecanoic acid | 0.000178914152131474 | 0.000142082766026002 | 0.000115348449439559 | 0.000149846492850154 | 0.000122856549454064 | 0.000134162919588135 | 0.000145432615028673 | 0.00016583056884375 | 0.000183687088843243 | 0.000153420026786609 | 0.000158066007904133 | 0.000191745113113411 |
| pos\_1292 | 1-(beta-D-Glucopyranosyloxy)-3-octanone | 0.000922609764032186 | 0.00134595370473771 | 0.00183771201257685 | 0.000802237618657016 | 0.00116481311059918 | 0.00121866613149198 | 0.000740460318858703 | 0.00112341824275895 | 0.000919002232272736 | 0.00178593710541726 | 0.00126826499969806 | 0.00136936628329615 |
| pos\_1293 | 3-Mercapto-2-methyl-1-butanol | 5.70622305543822e-06 | 4.89903656397404e-06 | 4.6908256243583e-06 | 4.25501685587033e-06 | 4.20873790839255e-06 | 4.98978654556474e-06 | 4.70863533849879e-06 | 4.91452501111544e-06 | 4.88594529045165e-06 | 4.45716187300884e-06 | 4.34316440855743e-06 | 4.83573424096396e-06 |
| pos\_1295 | PE(20:3(8Z,11Z,14Z)/22:0) | 1.26729803605664e-05 | 6.24730321695186e-06 | 3.29665821910793e-06 | 9.81137529856532e-07 | 1.34483003283512e-06 | 7.1300865169679e-06 | 7.48812236036562e-07 | 8.59906481880739e-07 | 1.0848958248778e-06 | 2.81674896187754e-06 | 3.0697949566729e-06 | 0 |
| pos\_1296 | 4-Amino-1-piperidinecarboxylic acid | 3.01089096771921e-05 | 4.10865619023338e-05 | 5.79191938927649e-05 | 5.8990026504973e-05 | 6.27490298586381e-05 | 4.76486184481597e-05 | 5.78025380562578e-05 | 6.24012316396137e-05 | 4.8948828051064e-05 | 4.57709542615969e-05 | 5.00261263020441e-05 | 5.4030951876026e-05 |
| pos\_1297 | 2-(4-Methyl-5-thiazolyl)ethyl decanoate | 0.000203914155447108 | 0.000175582827115808 | 0.000395912160540605 | 6.26513655319519e-05 | 0.000421874467468651 | 0.000252174168424208 | 3.86774362113439e-05 | 0.000103642392599656 | 9.7484462176299e-05 | 0.000846123042744953 | 0.000248702586465807 | 0.000201752467597731 |
| pos\_1298 | L-Hexahydro-3-imino-1,2,4-oxadiazepine-3-carboxylic acid | 2.44697944391922e-06 | 3.18532889338232e-06 | 2.00597701437951e-06 | 1.95376005855351e-06 | 4.60060547685507e-06 | 2.43390653120548e-06 | 3.4360324293688e-06 | 2.39572474601243e-06 | 3.90253805193798e-06 | 3.34008643663647e-06 | 2.15348334147023e-06 | 3.70968056096887e-06 |
| pos\_1299 | 5beta-1,3,7(11)-Eudesmatrien-8-one | 6.11850076560762e-05 | 6.33463725808219e-05 | 0.000115363617158126 | 9.24705308434257e-05 | 0.00011409148490573 | 8.7913497328082e-05 | 5.74331009644497e-05 | 6.36539804891353e-05 | 9.70098157044989e-05 | 9.04270807990136e-05 | 7.29949988195938e-05 | 8.11542309276455e-05 |
| pos\_13 | Guanosine | 2.23969131286124e-05 | 2.45634066321134e-05 | 1.62846721844283e-05 | 1.83684777618099e-05 | 3.06808072090743e-05 | 1.80085358199754e-05 | 2.32739563592637e-05 | 2.38366194607509e-05 | 2.20794098373643e-05 | 3.07768986298694e-05 | 2.12526076891193e-05 | 2.78755243416126e-05 |
| pos\_130 | Lecanoric acid | 1.64463899194351e-05 | 8.03777086085386e-05 | 9.73882416445587e-06 | 1.58271814701463e-05 | 2.51108658476852e-05 | 1.76293528950409e-05 | 1.3604381688604e-05 | 1.66058653963087e-05 | 2.55159686389316e-05 | 1.84902808914146e-05 | 1.52767136095026e-05 | 1.27971799704256e-05 |
| pos\_1300 | Laccarin | 0.000516177259545004 | 0.000307650675408676 | 0.000254183040458891 | 0.000252684547149847 | 0.000355979416085232 | 0.000312988965324206 | 0.000381544960738237 | 0.000327626993588758 | 0.000307203140802959 | 0.000283728953774324 | 0.000332283104344526 | 0.000336292210379265 |
| pos\_1301 | PS(14:1(9Z)/18:2(9Z,12Z)) | 3.20517760485464e-05 | 4.17087549303255e-05 | 4.20472383059359e-05 | 3.5340788118126e-05 | 3.05843560319044e-05 | 4.56051805142697e-05 | 3.356548323395e-05 | 4.0912149073291e-05 | 4.57232198446704e-05 | 4.07814627549097e-05 | 4.04359524062511e-05 | 3.64230470370473e-05 |
| pos\_1303 | 3-Acetoxyscirpene-4,15-diol | 2.16293973709398e-05 | 3.20586450074868e-05 | 1.74500153253142e-05 | 2.51789291274788e-05 | 2.2929694208359e-05 | 2.25208831862733e-05 | 2.13057430271401e-05 | 2.16944288012366e-05 | 1.76580048486725e-05 | 2.76695274020105e-05 | 2.05714950741328e-05 | 1.84228784949229e-05 |
| pos\_1304 | Norambreinolide | 8.27538699079665e-05 | 9.82526541448008e-05 | 0.000104456262470658 | 9.45787698874181e-05 | 8.07370970899906e-05 | 8.94933208072982e-05 | 8.56199298465793e-05 | 9.44291549689873e-05 | 9.51883621521759e-05 | 9.70911994352748e-05 | 9.41664173183019e-05 | 8.86065612991397e-05 |
| pos\_1305 | (7b,10a)-3-Hydroxy-1,3,5-cadinatrien-9-one | 4.37752120325568e-05 | 4.88599569577114e-05 | 4.37172205812665e-05 | 4.75383991364898e-05 | 3.94465643252729e-05 | 4.45766943964743e-05 | 4.05666837766621e-05 | 4.4215201580114e-05 | 3.9507557899205e-05 | 4.16809678594724e-05 | 4.38236525570408e-05 | 4.02445979033122e-05 |
| pos\_1306 | 2-(2-Furanyl)-3-piperidinol | 4.81448042470606e-05 | 5.78660839260259e-05 | 5.26647981351269e-05 | 4.98970936001761e-05 | 4.48317738257783e-05 | 4.92163117057226e-05 | 4.78284186052174e-05 | 5.17078678276201e-05 | 5.369684017699e-05 | 4.79680545125029e-05 | 5.21847437642022e-05 | 5.01532952251061e-05 |
| pos\_1307 | DG(14:1(9Z)/20:0/0:0) | 0.00216949753161676 | 0.00166345128763775 | 0.0014882139028658 | 0.00167534961564716 | 0.00131368858250914 | 0.00151887869208602 | 0.00153363350371889 | 0.00148988129499353 | 0.00162724523989405 | 0.0015055779900238 | 0.00155028443301969 | 0.00133971181005205 |
| pos\_1308 | ent-8(17),13(16),14-Labdatrien-18-oic acid | 6.17645902587467e-05 | 6.52661947738841e-05 | 6.04704345811661e-05 | 5.59757404560689e-05 | 5.32827301523296e-05 | 5.72108178971103e-05 | 5.24982833267222e-05 | 5.83158011068176e-05 | 5.39996342417839e-05 | 6.31710249172925e-05 | 5.63176573170488e-05 | 5.83845686364583e-05 |
| pos\_1309 | Azukisaponin VI | 8.9534843481527e-06 | 6.99805113265509e-06 | 3.13860346135632e-06 | 6.12594773370443e-06 | 1.26043944454872e-05 | 8.33647030811281e-06 | 1.49528661025606e-05 | 9.98507361711235e-06 | 7.29661020501872e-06 | 3.2012313508246e-06 | 9.20127269392335e-06 | 4.3690880524619e-06 |
| pos\_1310 | gamma-Eudesmol rhamnoside | 1.880146243414e-05 | 2.07891579137137e-05 | 1.7214056067928e-05 | 2.42531632168578e-05 | 0.000543584306261054 | 2.1453249131386e-05 | 2.40625232459175e-05 | 2.2676445876785e-05 | 1.99191016346778e-05 | 2.09512860985764e-05 | 2.27169584446e-05 | 2.24872126549293e-05 |
| pos\_1311 | 1-Deoxy-D-glucitol | 0.000101037532027429 | 7.77899387493278e-05 | 8.70331859208876e-05 | 5.98753450772786e-05 | 7.49816952073128e-05 | 7.74804940920492e-05 | 7.35623716034624e-05 | 7.88843704154777e-05 | 7.24427231728927e-05 | 9.14825177592364e-05 | 8.00039469143523e-05 | 8.84863177208163e-05 |
| pos\_1312 | 8-Hydroxyluteolin 4'-methyl ether 8-glucuronide | 1.66947190811225e-05 | 1.05881210588029e-06 | 2.28750088019599e-06 | 1.59936902347235e-06 | 9.01294083674315e-06 | 7.1774265957978e-06 | 8.26362612441436e-06 | 6.75096009497921e-06 | 2.33896454727637e-06 | 5.45487601429261e-06 | 4.9491536723369e-06 | 3.80475227353352e-06 |
| pos\_1313 | LysoPE(0:0/20:3(11Z,14Z,17Z)) | 2.78865517310007e-05 | 5.94365845055209e-05 | 3.32686472043953e-05 | 0.000125580944934329 | 1.17337145263852e-05 | 4.70014656972196e-05 | 2.50801436619003e-05 | 2.27095491718361e-05 | 7.07762466778637e-05 | 4.60526121164873e-05 | 3.49250803410823e-05 | 2.1914166394652e-05 |
| pos\_1314 | Panaxydol | 8.32677852614526e-05 | 0.000114633372203692 | 4.27434314488199e-05 | 5.87785358204991e-05 | 9.65364218267481e-05 | 7.34904115621484e-05 | 5.39637452942184e-05 | 4.27046724304506e-05 | 5.2759117371608e-05 | 7.65019602748674e-05 | 5.67294220553027e-05 | 4.45751910200535e-05 |
| pos\_1315 | Isopropylmaleate | 1.7344746416432e-05 | 2.48742239286796e-05 | 1.25964797854797e-05 | 2.11276772671357e-05 | 2.00022846694726e-05 | 1.75999854662526e-05 | 1.94735988577458e-05 | 1.68070395227514e-05 | 2.08822698184919e-05 | 2.10441658315305e-05 | 1.80701641688068e-05 | 1.32266832218429e-05 |
| pos\_1316 | Tsugaric acid B | 0.00814858214442947 | 0.00296824087484921 | 0.00297369924001983 | 0.00255114294921671 | 0.0037340099923378 | 0.00374999204560251 | 0.00401301564950852 | 0.00347163469339546 | 0.00335976075546833 | 0.00322260473126622 | 0.00352531118540033 | 0.00352455074223971 |
| pos\_1317 | Pipazethate | 7.04973001533332e-05 | 0.00011443115944705 | 7.6182975702631e-05 | 6.83695146135636e-05 | 7.66735589359853e-05 | 7.48446207895527e-05 | 0.000114417989239475 | 0.00010504134835483 | 7.13548946824478e-05 | 7.53873262770238e-05 | 9.698949181474e-05 | 9.03841217838162e-05 |
| pos\_1318 | Deoxyloganic acid | 0.000176174331844072 | 0.000178064962101222 | 0.000197283549066299 | 0.00016260095370794 | 0.000166826813774261 | 0.000178597746060186 | 0.000125215589890289 | 0.00016244245702283 | 0.00019213156828298 | 0.000174737708123971 | 0.000167919886783104 | 0.000196832166950055 |
| pos\_132 | 14-methyl palmitic acid | 0.000127558715619372 | 0.000113611209359602 | 0.000171969931895308 | 0.000184571664217063 | 7.20911765895505e-05 | 0.000133113990206956 | 0.000161421821618466 | 0.000152388478310156 | 0.000156238813546515 | 0.000118852363636095 | 0.000152498285543868 | 0.00015562813017119 |
| pos\_1320 | Momordicinin | 0.00202323143465156 | 0.00180201663882979 | 0.00160180934016776 | 0.00181835338793389 | 0.00170551126233748 | 0.00172142235207509 | 0.00199173198764084 | 0.00190849374685039 | 0.00196406490718556 | 0.00188976725775007 | 0.00183996293703257 | 0.00189404331041662 |
| pos\_1321 | Montecristin | 0.000327967264273695 | 0.000862189086946261 | 9.98503111623536e-05 | 9.29602844452244e-05 | 7.40989823107653e-05 | 0.000214948970037813 | 0.000106432772485059 | 0.000114327533787874 | 9.96347372999422e-05 | 8.93894307566803e-05 | 9.42412352465548e-05 | 0.00013905065211803 |
| pos\_1322 | Linalool oxide D 3-[apiosyl-(1->6)-glucoside] | 0.0001891907221572 | 0.000184391094398995 | 0.000186179616068265 | 0.000158264846555223 | 0.000189069136368396 | 0.000175748202956967 | 0.00019544767021446 | 0.000185944965197339 | 0.000154761556899056 | 0.0002012480283098 | 0.00015472344687666 | 0.000197991869108207 |
| pos\_1323 | Guanidinosuccinic acid | 1.81222276171407e-05 | 1.85047438939659e-05 | 1.3802855521764e-05 | 2.27998558003795e-05 | 1.52709612350273e-05 | 1.9161265097041e-05 | 1.87153264668074e-05 | 1.75190611026465e-05 | 1.53643167161108e-05 | 1.71162889530957e-05 | 1.92887702243487e-05 | 1.44993640831987e-05 |
| pos\_1325 | Pergolide | 0.000303669113240613 | 0.000305794179840574 | 0.000316664948751881 | 0.000153894303978439 | 0.000216386057733653 | 0.000265598092152241 | 9.56301147257149e-05 | 0.000188733331174315 | 0.000106346235310959 | 0.000322505435158013 | 0.00017169334010077 | 0.00040554955430879 |
| pos\_1326 | Nabilone | 0.000218497942052943 | 0.000253316040996134 | 0.000426560126972914 | 0.000514238552458472 | 0.000610196352340334 | 0.000364434657827048 | 0.0004923676577913 | 0.000510134581270596 | 0.00041588986965582 | 0.000318810524463975 | 0.000365672083883234 | 0.000336775495966155 |
| pos\_1327 | O-acetyl-ADP-ribose | 2.47839804968465e-05 | 6.1059696391284e-06 | 8.71787606722655e-06 | 7.49127240745354e-06 | 9.61115883272584e-06 | 8.76283551695161e-06 | 1.209976075925e-05 | 8.23049306348961e-06 | 1.0674384094721e-05 | 8.76793689051337e-06 | 9.16079349602323e-06 | 1.03376790390848e-05 |
| pos\_1328 | (Z)-3-Oxo-2-(2-pentenyl)-1-cyclopenteneacetic acid | 1.8814823067755e-05 | 4.60840265327482e-05 | 1.78962766627701e-05 | 2.01162827468139e-05 | 2.45673449442638e-05 | 2.43162178694514e-05 | 4.28630834050164e-05 | 3.60262904381374e-05 | 2.53429394700381e-05 | 2.51657649496546e-05 | 3.29211663591323e-05 | 0.00223679593485489 |
| pos\_1329 | Indolylacryloylglycine | 5.75292886424592e-06 | 7.85184886707646e-06 | 8.13294922482475e-06 | 5.21323356165369e-06 | 3.75248309755147e-06 | 5.96901064683354e-06 | 4.02432046093245e-06 | 6.02879655339154e-06 | 6.8106195620399e-06 | 5.5523799028788e-06 | 4.92378624667949e-06 | 4.73208789418985e-06 |
| pos\_133 | 1α,25-dihydroxy-24-nor-22-oxavitamin D3 / 1α,25-dihydroxy-24-nor-22-oxacholecalciferol | 0.000103118850128283 | 0.000109063317709569 | 9.64100774997213e-05 | 8.9872428638604e-05 | 0.000109880167922847 | 9.58178089315817e-05 | 0.000160481239787251 | 0.000123965532079287 | 0.000161581551878289 | 0.000113844242670593 | 0.000131320970480963 | 8.03247388867606e-05 |
| pos\_1330 | Peltatol C | 0.000460556499509836 | 0.000191307418356675 | 0.000178377954977959 | 0.000171249526430633 | 0.000207444634495452 | 0.000211593462989187 | 0.000236019582605146 | 0.000198317396286914 | 0.000193173244676762 | 0.000177143033636242 | 0.000192064421838601 | 0.000184410961591127 |
| pos\_1332 | erythro-6,8-Pentacosanediol | 0.000500449163567048 | 0.000540766376498502 | 0.000519434745797191 | 0.000613444286314808 | 0.00048103096352461 | 0.00056390862885142 | 0.000567130944758369 | 0.000574502764623688 | 0.000558364927434623 | 0.000567466841334785 | 0.000587790723345189 | 0.000547181324354769 |
| pos\_1333 | Alliosterol 1-(4''-galactosylrhamnoside) 16-galactoside | 5.87076575108998e-05 | 6.41237118290316e-05 | 5.19127096878858e-05 | 0.000174709824766309 | 4.98791157192279e-05 | 5.69617267699342e-05 | 4.95466294011468e-05 | 0.000112983712230291 | 0.000267934085034741 | 7.97093606466462e-05 | 0.000213247532131607 | 0.00014042050970485 |
| pos\_1334 | 1,24-Tetracosanediol | 0.000254399988050147 | 0.000762739202962404 | 0.00107482059960423 | 0.000542228070809575 | 0.000434822675468574 | 0.000524058972487762 | 0.000576998075166454 | 0.000988384093429528 | 0.000628770182933209 | 0.000627204499842167 | 0.00073015790217037 | 0.00102759437664897 |
| pos\_1336 | Neotussilagine | 1.10143275689103e-05 | 2.31595750292418e-05 | 2.14060042750917e-05 | 2.09439965449765e-05 | 9.41871854216108e-06 | 1.94859342056183e-05 | 1.69415846838409e-05 | 2.95510901394166e-05 | 1.60516246212235e-05 | 1.58074107108644e-05 | 1.52973067358869e-05 | 1.84286882994678e-05 |
| pos\_1338 | 6-Succinoaminopurine | 3.06504766705764e-05 | 4.51665606307399e-05 | 2.34860611438098e-05 | 3.0817497537213e-05 | 3.24169172222506e-05 | 2.91580919887087e-05 | 3.2075769330625e-05 | 2.82471638607767e-05 | 3.13709833730862e-05 | 3.84328448606904e-05 | 2.80675322326827e-05 | 2.75070603977905e-05 |
| pos\_1339 | PE(18:3(6Z,9Z,12Z)/20:5(5Z,8Z,11Z,14Z,17Z)) | 2.6737422015098e-05 | 1.06320367149939e-05 | 6.12161318031332e-05 | 2.38207796162021e-05 | 3.67652222287132e-05 | 0.000219668919948046 | 5.6437120313746e-05 | 6.2945311180036e-05 | 8.03107635436269e-05 | 0.000124542637431557 | 5.17243721510084e-05 | 3.91435682518875e-05 |
| pos\_134 | 3-Oxo-7α-hydroxy-5α-cholestan-26-oic acid | 0.000597888574619807 | 0.000809571516450661 | 0.000402010106996638 | 0.00061205327314991 | 0.000692521707040265 | 0.000630516685064865 | 0.00051559979328173 | 0.000372823786799877 | 0.000402592706906503 | 0.000760776025471454 | 0.000473564140212548 | 0.00025765746727218 |
| pos\_1341 | Isoleucyl-Tryptophan | 0.00649811595198651 | 0.00293477443289898 | 0.00271437686388168 | 0.00249570365767038 | 0.00438688205007149 | 0.00446498034389829 | 0.00480372025142202 | 0.00385208975071607 | 0.00357112643550484 | 0.00356931586040785 | 0.00379450102718991 | 0.0040797248078488 |
| pos\_1343 | Procurcumenol | 3.16717766270441e-05 | 3.85051518905014e-05 | 3.43853873818067e-05 | 3.4151243273245e-05 | 2.60870942943951e-05 | 3.22925731498529e-05 | 3.1779555503327e-05 | 3.3299571693899e-05 | 3.53332791573456e-05 | 3.02187771727824e-05 | 3.26610406959695e-05 | 3.15807340422501e-05 |
| pos\_1344 | beta-Carotenone | 0.0022761867463638 | 0.00318423756925844 | 0.00256279140412057 | 0.00261435669431372 | 0.00172562366324456 | 0.00291905290085321 | 0.00238878150544501 | 0.00269775461754015 | 0.00280758670856616 | 0.00196207725064265 | 0.00280412881400834 | 0.00228338392682814 |
| pos\_1345 | Ganoderic acid S | 4.49652912993909e-05 | 2.19621806563784e-05 | 2.42535871999318e-05 | 2.10549758138459e-05 | 2.4922907857798e-05 | 2.24476041671605e-05 | 4.74933058891599e-05 | 3.56479349312388e-05 | 4.77356813389328e-05 | 1.9268488434717e-05 | 2.64519816698155e-05 | 4.77973745079864e-05 |
| pos\_1347 | 1(10),11-Eremophiladiene-2,9-dione | 4.24099807436897e-06 | 5.38725000230506e-06 | 5.88934074340801e-06 | 5.80958631703303e-06 | 5.46529717413635e-06 | 5.36196926011316e-06 | 5.39266050009931e-06 | 6.90302407684911e-06 | 5.73398984873798e-06 | 5.95688276336644e-06 | 6.20198315186751e-06 | 6.8049647913759e-06 |
| pos\_1348 | Glucoconvallatoxoloside | 7.65683111923769e-06 | 1.91755344570296e-05 | 3.57636969231502e-06 | 6.6459214561802e-06 | 8.63219155798208e-06 | 8.39543580882237e-06 | 7.02185774582547e-06 | 4.43972974620776e-06 | 1.03849644230368e-05 | 1.23294820172559e-05 | 9.12642297284605e-06 | 5.63712527588574e-06 |
| pos\_1349 | 4,6-Heptadiyne-1,3-diol | 2.421141771244e-05 | 3.04387274509037e-05 | 2.7298347758023e-05 | 2.53206073315873e-05 | 2.26713872592574e-05 | 2.47734927623419e-05 | 2.23717341569504e-05 | 2.49347026720107e-05 | 2.78098103379943e-05 | 2.17498432384074e-05 | 2.46424778713394e-05 | 2.46888131854905e-05 |
| pos\_135 | 1α-hydroxy-24-(dimethylphosphoryl)-25,26,27-trinorvitamin D3 / 1α-hydroxy-24-(dimethylphosphoryl)-25,26,27-trinorcholecalciferol | 0.00028219052587091 | 0.000237526266822087 | 0.000192372339695398 | 0.000299684189845267 | 0.000229327687949681 | 0.000252811172449093 | 0.000482333114813914 | 0.000361695335913765 | 0.000277944861038184 | 0.000284480114025575 | 0.000343538576855507 | 0.000244510545152894 |
| pos\_1350 | Nandrolone phenpropionate | 4.95239835243586e-06 | 7.43772390751014e-06 | 3.08264539801232e-05 | 2.87257484499613e-06 | 1.9952011404309e-06 | 9.05116240347787e-06 | 5.86586358898253e-06 | 0.000134314166112847 | 7.31486270957778e-05 | 2.12612561358357e-06 | 9.82651524912269e-05 | 0.000343470149132853 |
| pos\_1351 | 4-Acetylzearalenone | 2.3035408682377e-05 | 2.13801697994952e-05 | 4.93267348738042e-06 | 1.90088976699097e-05 | 1.76366283178207e-05 | 1.57909754403001e-05 | 5.12636503859076e-05 | 3.33282324464124e-05 | 9.78353023097308e-06 | 5.25061922141539e-05 | 3.30485603454829e-05 | 1.06366986049921e-05 |
| pos\_1352 | Zingerone | 2.7484524312487e-06 | 4.08493705002979e-06 | 2.40585776387901e-06 | 2.64954467435271e-06 | 2.66559191767664e-06 | 2.67650041940721e-06 | 3.04368811483279e-06 | 2.25612406509945e-06 | 5.18875944269072e-06 | 2.58512243940153e-06 | 3.26067539179862e-06 | 3.14781838338323e-06 |
| pos\_1353 | PS(DiMe(13,5)/MonoMe(11,5)) | 1.5012588045451e-05 | 5.27202238242029e-06 | 3.23542116623596e-05 | 1.45630821490225e-05 | 2.28837085222071e-05 | 0.000132849772580879 | 3.70851718160335e-05 | 3.72435581405282e-05 | 4.89439409068876e-05 | 7.05321040245912e-05 | 3.40903751996958e-05 | 2.57829644613861e-05 |
| pos\_1354 | Desonide | 7.31213702834783e-06 | 1.26205376156141e-05 | 3.35859033563193e-06 | 5.07090953040899e-05 | 5.07034771009731e-06 | 1.79081233408276e-05 | 1.98401319700363e-05 | 1.28904077747235e-05 | 6.08703867226003e-09 | 2.61098220449751e-06 | 1.1113344764176e-05 | 4.16174321273513e-07 |
| pos\_1355 | Stearic acid | 0.000247035128140017 | 0.000240938133782215 | 0.000513792119563923 | 0.000421044669512726 | 0.000372094378878419 | 0.000349220790032932 | 0.000228422021064922 | 0.000497563358540792 | 0.000270030499518995 | 0.000238512289588293 | 0.000340596370960418 | 0.000482508198303188 |
| pos\_1356 | Tri-N-acetylchitotriose | 6.254774774763e-06 | 3.48335474280711e-05 | 6.63899876223385e-07 | 2.35415141405784e-06 | 2.0799618747688e-05 | 1.19897950151721e-05 | 7.93030852926571e-06 | 2.30894918046743e-06 | 9.11494385960132e-06 | 2.39848961826292e-05 | 8.95207514029987e-06 | 2.83117892518686e-06 |
| pos\_1358 | Phytocassane B | 4.23482387234368e-05 | 6.07662891009611e-05 | 5.54048509199359e-05 | 5.59494189725341e-05 | 3.73490795335637e-05 | 5.24327806963118e-05 | 4.28047019229227e-05 | 5.31157719394843e-05 | 5.79901350944425e-05 | 4.38845140039926e-05 | 4.85354846542403e-05 | 4.7024983320669e-05 |
| pos\_136 | Ala Phe Ile | 0.00587146015636069 | 0.00304392233094023 | 0.00102204846443927 | 0.00261636085813087 | 0.000453814922389576 | 0.00243252080725715 | 0.00064638126608757 | 0.000871155695349433 | 0.00139788880346509 | 0.00747615734486529 | 0.00226674945931043 | 0.0013437158991428 |
| pos\_1360 | 3b,17b-Dihydroxyetiocholane | 2.09527933839213e-05 | 2.97526053201771e-05 | 2.9799969085188e-05 | 6.64977917681452e-06 | 7.33245523830438e-06 | 7.86335817362492e-05 | 5.97754242503466e-06 | 6.91401888843919e-05 | 6.5930802590592e-06 | 7.56990367163447e-05 | 8.61492390273227e-05 | 7.56425234322213e-05 |
| pos\_1361 | 6b-Hydroxy-8a-methoxy-7(11)-eremophilen-12,8-olide | 0.000108834444216852 | 0.000174335743349524 | 0.00013775391221936 | 0.00014805059891577 | 0.00010835119050981 | 0.000141462757847652 | 0.000134071075486489 | 0.000127384809983552 | 0.000151478344842971 | 0.000138760016018241 | 0.000138062938716541 | 0.000111685438905082 |
| pos\_1362 | Sparfloxacin | 1.18706276380756e-05 | 8.38718685838071e-06 | 7.12383378181231e-06 | 8.4194443664096e-06 | 6.15727168790657e-06 | 7.33849452667918e-06 | 8.1918816201105e-06 | 7.73448638941517e-06 | 7.03314019384478e-06 | 8.86465961327815e-06 | 7.60120215440479e-06 | 1.12771296499337e-05 |
| pos\_1363 | L-trans-5-Hydroxy-2-piperidinecarboxylic acid | 5.64151332686819e-05 | 5.44113921284071e-05 | 7.05899073762935e-05 | 4.02058227496322e-05 | 5.42766070013896e-05 | 5.27275393890799e-05 | 4.74176287445445e-05 | 5.54502446676625e-05 | 4.24018095365618e-05 | 7.59268065483188e-05 | 5.8976468293509e-05 | 6.97515453303567e-05 |
| pos\_1364 | Quinquenoside F1 | 8.73342581337702e-05 | 5.19874603002746e-05 | 3.64068191130312e-05 | 5.04620943632213e-05 | 5.1263371577389e-05 | 5.79595569979693e-05 | 5.18063183053235e-05 | 5.04276587871877e-05 | 5.00512201598686e-05 | 4.32251125060748e-05 | 4.45671124362657e-05 | 5.39535575346622e-05 |
| pos\_1365 | AS 1-1 | 0.000324776566342586 | 0.00012598224483792 | 0.000113674188593046 | 0.000104442582157014 | 0.000154165391173389 | 0.000147816911768643 | 0.000173003373165158 | 0.000139478821321343 | 0.000128509855616811 | 0.000127669264071462 | 0.00013815440172884 | 0.00013711928705234 |
| pos\_1366 | TG(14:1(9Z)/18:4(6Z,9Z,12Z,15Z)/22:6(4Z,7Z,10Z,13Z,16Z,19Z)) | 7.49093799587881e-05 | 0.000101168809196972 | 8.67575935486547e-05 | 0.000107564658642619 | 9.19548409346223e-05 | 0.000104732433851904 | 9.96645591203828e-05 | 8.72235647282508e-05 | 0.00012082639579915 | 0.000100952519615512 | 0.000104948095875248 | 8.30049751558049e-05 |
| pos\_1367 | 3-hydroxypentadecanoyl carnitine | 3.25581024774294e-06 | 4.80509939227293e-06 | 1.39054168676293e-06 | 4.52601929323536e-06 | 2.74374877358906e-06 | 4.26802516362791e-06 | 4.79785954308387e-06 | 3.36548561054646e-06 | 4.74112351231887e-06 | 5.3378833228614e-06 | 5.28641887875219e-06 | 1.82694800437445e-06 |
| pos\_1368 | lysoPC(26:1(5Z)) | 1.83898722185486e-05 | 5.94463922817574e-06 | 1.70832327473927e-06 | 2.536549891259e-08 | 6.13665070420939e-07 | 4.91497284627991e-06 | 2.97140099125371e-05 | 1.72482269308471e-05 | 1.39719236069853e-06 | 6.50027549007252e-06 | 7.02330149916486e-06 | 1.38168195829853e-06 |
| pos\_1369 | Alanyltryptophan | 2.47062469849395e-05 | 3.256175312242e-05 | 1.42272060706712e-05 | 2.2896397187451e-05 | 2.66153220706785e-05 | 2.29508609885507e-05 | 2.14982203690657e-05 | 1.76514686604683e-05 | 2.11796690154195e-05 | 2.71578847165911e-05 | 2.03595537494195e-05 | 1.69660300378409e-05 |
| pos\_137 | 2,6,6-Trimethyl-2-cyclohexene-1,4-dione | 0.000173893928184645 | 0.000177294662685662 | 2.24096864328154e-05 | 0.000109162126041124 | 0.000179773753278698 | 0.000137010659799997 | 2.41227576760009e-05 | 2.43181109419071e-05 | 1.97511558758124e-05 | 0.000131901885625438 | 5.0594031133571e-05 | 2.83048422175909e-05 |
| pos\_1370 | FMNH2 | 1.85471589702265e-05 | 1.41158741298001e-05 | 0 | 2.74876998979499e-06 | 1.3822956366106e-05 | 6.66522024259159e-06 | 3.46808654564704e-07 | 2.7550334907704e-07 | 1.8953720964621e-07 | 4.52544860509124e-06 | 5.08894023222634e-07 | 9.17553807195752e-08 |
| pos\_1371 | Aeglin | 3.06705046384331e-06 | 4.89357185441307e-06 | 4.41569515365794e-07 | 3.89884570725048e-06 | 2.6436838958978e-06 | 2.76205523266864e-06 | 3.41668430026941e-06 | 1.9180868558296e-06 | 5.4104279012127e-07 | 3.81054398751788e-06 | 2.60007083478185e-06 | 3.05544254430245e-07 |
| pos\_1372 | Indole-3-carboxilic acid-O-sulphate | 8.89509012233907e-06 | 2.2056008512307e-05 | 9.06191591890368e-06 | 1.66615568582796e-05 | 2.03101605761738e-05 | 1.2721520254882e-05 | 1.79801407545588e-05 | 1.46244377369279e-05 | 7.62254377149198e-06 | 1.23882707710748e-05 | 1.41315821630702e-05 | 1.02471654851816e-05 |
| pos\_1373 | Roxithromycin | 0.00314695761816335 | 0.00106490295244397 | 0.0010132594593653 | 0.000865466511225282 | 0.001416330214597 | 0.00136124006851067 | 0.00149500608188409 | 0.00122371177250459 | 0.00119557500250566 | 0.00115359698737976 | 0.00124429627225821 | 0.00120184367435356 |
| pos\_1374 | Nookatone | 5.3796619703416e-05 | 5.99853689220266e-05 | 6.23422800987689e-05 | 7.92900108071659e-05 | 0.000105835340355188 | 6.7540808977089e-05 | 6.69396544230485e-05 | 6.97869495532966e-05 | 7.08145597297352e-05 | 6.86928102085621e-05 | 6.9620814319309e-05 | 7.67442906257143e-05 |
| pos\_1375 | Phaeophorbide b | 0.000149423863082705 | 0.000206605961466955 | 0.000132932608977143 | 0.000132244364865358 | 0.000155242634599261 | 0.00017091433106524 | 0.000269311673112893 | 0.000241852240413771 | 0.000137529256797135 | 0.000177755440418584 | 0.000192645867807034 | 0.000155638910445327 |
| pos\_1376 | Brassicoside | 1.0054481960694e-05 | 1.16962725187996e-05 | 0 | 7.98012419853088e-06 | 9.71768486525661e-06 | 7.19593711026315e-06 | 0 | 0 | 0 | 6.89429874645566e-06 | 7.16181342852175e-07 | 0 |
| pos\_1379 | Cyclotricuspidoside C | 7.77392250774873e-05 | 9.40483606809514e-05 | 0.000122511097266598 | 3.19403944603029e-05 | 9.04932048430892e-05 | 0.000100048525741076 | 2.06119197202191e-05 | 7.04253314938337e-05 | 4.2996249554783e-05 | 0.000123298862162946 | 9.28658533688427e-05 | 0.000102567568741046 |
| pos\_138 | 11-(4-acetoxymethylphenyl)-1α,25-dihydroxy-9,11-didehydrovitamin D3 / 11-(4-acetoxymethylphenyl)-1α,25-dihydroxy-9,11-didehydrocholecalciferol | 0.00307162306466618 | 0.00354014715810102 | 0.00254830677685345 | 0.00319221571706638 | 0.00368773956450683 | 0.0031275528941251 | 0.00267903361422274 | 0.00290814239815041 | 0.00366051057399974 | 0.00365843118780879 | 0.00315445974843328 | 0.00277640563721515 |
| pos\_1380 | (R)-1-O-[b-D-Apiofuranosyl-(1->2)-b-D-glucopyranoside]-1,3-octanediol | 3.8533823709197e-05 | 8.92111201940979e-06 | 3.36055727216099e-06 | 8.93930421140541e-05 | 1.24252103485763e-05 | 2.5110660341257e-05 | 2.77675418304424e-05 | 3.07455295139841e-05 | 3.95747349173901e-06 | 0.000120031862252035 | 4.15111999460586e-05 | 3.77139177942038e-05 |
| pos\_1381 | Araliasaponin III | 2.49349552505218e-05 | 3.63332145908761e-05 | 4.73268578129351e-05 | 4.04423943027185e-05 | 3.15883599279883e-05 | 4.86669122471633e-05 | 4.15491509500115e-05 | 5.44700190339456e-05 | 4.95540982352512e-05 | 4.9075064340246e-05 | 4.55691760025956e-05 | 4.9763153662264e-05 |
| pos\_1382 | 1-Lyso-2-arachidonoyl-phosphatidate | 7.76268984074505e-06 | 3.16758228187999e-05 | 9.21120666325102e-07 | 1.02287799920914e-06 | 6.32221619147672e-07 | 5.45236247942563e-06 | 4.67720626791323e-07 | 1.15414333412603e-05 | 7.0478241718551e-06 | 1.71312029987501e-06 | 8.62272188364581e-06 | 2.79031438474011e-05 |
| pos\_1383 | 3-Hexaprenyl-4-hydroxybenzoic acid | 0.00252282694360387 | 0.00135399977874569 | 0.00125615210114881 | 0.00111134664783451 | 0.00136494687467216 | 0.00141317538142883 | 0.00143685247714904 | 0.00134027670508201 | 0.0014574985080816 | 0.00128968349326358 | 0.00136422772813082 | 0.00129998999729388 |
| pos\_1385 | (Z)-6-(2-Methoxyvinyl)-7-methyl-2H-1-benzopyran-2-one | 2.93920584981554e-06 | 2.68926007441298e-06 | 2.80390228039052e-06 | 2.63477613414478e-05 | 4.72595285324771e-06 | 4.94292311320814e-06 | 4.22979710440418e-06 | 6.75921830407586e-06 | 2.74767866217556e-06 | 3.69926393683203e-06 | 5.12638561747279e-06 | 1.28353345348789e-05 |
| pos\_1386 | Yohimbine | 6.44850184610335e-06 | 1.28514119654289e-05 | 1.78616975243178e-05 | 3.98289676486193e-06 | 9.69427562872753e-06 | 1.43166620884793e-05 | 2.96329475040052e-06 | 8.87541448168644e-06 | 4.07183893969935e-06 | 6.45148083356849e-05 | 1.4942324116472e-05 | 2.00757588680286e-05 |
| pos\_1387 | Lyoniresinol 9'-sulfate | 3.08535465441221e-06 | 4.36112629158822e-06 | 1.83356285771595e-06 | 8.79837456478572e-06 | 1.64972708955126e-07 | 2.17453389570295e-06 | 8.27645202096129e-06 | 1.34003361273079e-06 | 1.06517403470054e-06 | 8.57703935342127e-06 | 2.80674415183221e-06 | 5.39292529443982e-07 |
| pos\_1388 | 2,3-Dehydrosilybin | 0 | 0 | 4.9564236760293e-06 | 8.5901054589374e-07 | 1.69888160291032e-07 | 0 | 0 | 2.09422619478384e-06 | 0 | 5.2066867750608e-06 | 0 | 3.17906709192295e-06 |
| pos\_1389 | N-Ornithyl-L-taurine | 5.96204382176876e-06 | 5.66769610115565e-06 | 2.97796051560321e-06 | 5.76885868273064e-06 | 5.55463672641555e-06 | 6.65789389596654e-06 | 6.22553634272795e-06 | 4.18881557049488e-06 | 5.26684746104937e-06 | 3.22861380077625e-05 | 1.11631703287356e-05 | 3.63854913717342e-06 |
| pos\_1390 | Polypropylene glycol (m w 1,200-3,000) | 0.000118384729520067 | 0.000324743305284892 | 4.08872626528533e-05 | 5.07723036994431e-05 | 0.00014596073807235 | 0.000118214211779411 | 6.03008970572202e-05 | 5.29636032679468e-05 | 5.67750199523391e-05 | 5.54571809088907e-05 | 5.6122251592352e-05 | 4.68321649480113e-05 |
| pos\_1391 | (+)-4,11-Eudesmadien-3-one | 0.000205081204713271 | 0.000306509382455052 | 0.000403070812608325 | 0.00028677026593061 | 0.000350954639181389 | 0.000316237246173316 | 0.000286903720769829 | 0.000357878490225375 | 0.000280845161155917 | 0.000437252477602177 | 0.000336685785197329 | 0.000437396613291853 |
| pos\_1392 | 6-Gingesulfonic acid | 4.75208938254074e-05 | 8.04112672829539e-05 | 4.14503411315364e-05 | 5.70868383649864e-05 | 5.34230141338005e-05 | 4.99490811097614e-05 | 6.42993209612325e-05 | 5.25196030137494e-05 | 6.4838023985904e-05 | 5.63789184149961e-05 | 5.61003592355329e-05 | 4.44612007810351e-05 |
| pos\_1393 | Murrayacinine | 7.36305987918733e-06 | 1.71486937577001e-05 | 1.75732672666085e-05 | 4.75840353792433e-05 | 1.72063008455686e-05 | 2.32980803363806e-05 | 1.05922450286028e-05 | 1.83680249435241e-05 | 8.85157650162239e-06 | 1.03271040396871e-05 | 1.60330131228418e-05 | 4.56613723619494e-05 |
| pos\_1394 | Nandrolone decanoate | 0.000139457250575324 | 0.000174247360561005 | 0.000270380805770083 | 0.000254274820689011 | 0.000135111627790851 | 0.000187588088624909 | 0.000176575498449584 | 0.000211171561531781 | 0.000167055006041947 | 0.000205555001519346 | 0.000208075670080589 | 0.000269680744839691 |
| pos\_1396 | Myristoylglycine | 0.000142768116863129 | 0.000186646356196267 | 0.000172917240210142 | 0.00015820504838833 | 0.00013250174724531 | 0.000152143507383544 | 0.00014584165251344 | 0.000158407381944778 | 0.000173473488736085 | 0.000154471513975638 | 0.000151945523129179 | 0.000147912436145578 |
| pos\_1397 | Glucosylceramide (d18:1/12:0) | 2.65911707281154e-05 | 1.37332970356156e-05 | 6.95819882434727e-06 | 1.21071728812781e-05 | 1.81379135113827e-05 | 1.68474768246097e-05 | 8.2194410937336e-05 | 3.95378746917453e-05 | 6.98886337784321e-06 | 2.47080764053181e-05 | 3.4338581581159e-05 | 1.24528382291068e-06 |
| pos\_1398 | Gemfibrozil | 4.25034274246691e-05 | 5.31746026636744e-05 | 3.72814781927738e-05 | 4.2656282310661e-05 | 4.09883459991341e-05 | 3.92204388896029e-05 | 3.10966700280669e-05 | 3.86845975148939e-05 | 4.73275410381517e-05 | 3.92891622949329e-05 | 3.97238610570243e-05 | 5.23552650896038e-05 |
| pos\_1399 | Nepafenac | 5.25720419294241e-05 | 7.63586896931866e-05 | 3.1725711643577e-05 | 5.24027536340587e-05 | 5.58811634304612e-05 | 4.96445403309895e-05 | 5.20071358031181e-05 | 4.62086970580991e-05 | 5.04951474517517e-05 | 5.75266748579212e-05 | 4.68590702623293e-05 | 3.5376134621047e-05 |
| pos\_14 | Decanoic acid | 8.72341904861399e-07 | 1.28076847728259e-06 | 2.86446709936845e-06 | 4.10845802779418e-06 | 2.99538296233773e-06 | 3.16988921531297e-06 | 3.54799515097164e-06 | 3.28141099553559e-06 | 3.65019464165561e-06 | 2.70894810953893e-06 | 3.74133783717818e-06 | 3.53914311987931e-06 |
| pos\_140 | (24R)-1α,24,25-trihydroxy-22-oxavitamin D3 / (24R)-1α,24,25-trihydroxy-22-oxacholecalciferol | 0.000725802760022383 | 0.000503918261020971 | 0.000476495549673714 | 0.000606214314634699 | 0.000468334289480201 | 0.000573192427469647 | 0.000622162502390334 | 0.000917976341366234 | 0.000745895634078685 | 0.000537248937726507 | 0.000803244960149637 | 0.00130789714618476 |
| pos\_1400 | (±)-(Z)-2-(5-Tetradecenyl)cyclobutanone | 0.0136869845971094 | 0.0205875463456008 | 0.0184105544930216 | 0.0197947455156409 | 0.0151575713392229 | 0.0157125505671298 | 0.0117520446741079 | 0.0161335432430645 | 0.0181678577313539 | 0.0152919984637677 | 0.0173533986361573 | 0.0162338653995277 |
| pos\_1401 | Chlorprothixene | 0.000477937932386951 | 0.000137791279467944 | 0.000154675202051936 | 0.000118271482628789 | 0.000273894889113452 | 0.000279135031140794 | 0.000247602494008394 | 0.000220925308016467 | 0.000182818189914009 | 0.000186546092693689 | 0.000211566494969743 | 0.000200963490140788 |
| pos\_1402 | (E,E)-11,13-Octadecadien-9-ynoic acid | 0.000151585544599397 | 0.000195955818073042 | 0.000181503600402172 | 0.000176196950167957 | 0.00015063652819877 | 0.000177616104988897 | 0.000201969805855275 | 0.000189037083223811 | 0.000197962916260091 | 0.000170442908957652 | 0.000185319707241882 | 0.000164265782977297 |
| pos\_1403 | PE(14:0/P-18:0) | 0.00607455983589868 | 0.00676178218458976 | 0.00680735785038745 | 0.00352954376692546 | 0.00322008672234822 | 0.00509691608259597 | 0.00226511916412327 | 0.00444004671895304 | 0.00411842229003799 | 0.00363511354087203 | 0.00429576025309229 | 0.00616907062951013 |
| pos\_1404 | Haloperidol glucuronide | 3.86778959094141e-06 | 1.49046159881476e-06 | 3.01136244634738e-07 | 1.04372578064994e-06 | 3.50862047660615e-06 | 2.34029552167867e-06 | 3.01949625035258e-06 | 1.33915161065539e-06 | 1.36376694574029e-06 | 1.23206790121027e-06 | 1.85967431214129e-06 | 1.03849906310489e-05 |
| pos\_1405 | 3-Nonyl-1H-pyrazole | 3.59184521611174e-05 | 3.99513079773181e-05 | 2.68845216465845e-05 | 3.27781641715589e-05 | 5.33968577159656e-05 | 2.64527138168968e-05 | 3.60222831901359e-05 | 3.58681963882764e-05 | 4.12912004505729e-05 | 2.65539713024974e-05 | 3.35540549825908e-05 | 4.10845821956566e-05 |
| pos\_1406 | Patuletin 3-gentiobioside | 0.0005358755494294 | 0.000140999727690652 | 0.000143050680553857 | 0.00011535507795624 | 0.00030954156176188 | 0.000308184013517262 | 0.000271852027999197 | 0.000229324236629694 | 0.000186558771701023 | 0.000203187045691415 | 0.000229977543762353 | 0.00021073361013034 |
| pos\_1407 | 21-Deoxycortisol | 5.18855724099993e-05 | 6.79199437292507e-05 | 0.000469484921772352 | 4.89162292712085e-05 | 4.87536631886777e-05 | 6.11622150866823e-05 | 0.00018190384911729 | 0.0002142192943961 | 6.50062299058375e-05 | 0.000219815959119168 | 0.000370299957269009 | 6.19503530038587e-05 |
| pos\_1408 | PE(22:5(7Z,10Z,13Z,16Z,19Z)/dm18:1(9Z)) | 0.000318633126348585 | 0.000416452668446025 | 0.000537908005592419 | 0.000392698300311282 | 0.000307323035793485 | 0.000362414567739016 | 0.00033250011599045 | 0.000404156794748062 | 0.000429616260718163 | 0.000322871504174112 | 0.000392113260425972 | 0.000387928911270317 |
| pos\_1409 | 13-Nor-6-eremophilene-8,11-dione | 2.34089967837454e-05 | 2.92703862385402e-05 | 2.59232366198029e-05 | 2.51849466616745e-05 | 1.91709423452221e-05 | 2.40741222220352e-05 | 2.15181906830996e-05 | 2.45844381075372e-05 | 2.45325858475857e-05 | 2.27379808367324e-05 | 2.48740718624218e-05 | 2.22568471809125e-05 |
| pos\_141 | 2,2,4,4,6,6-Hexamethyl-1,3,5-trithiane | 2.7315854878403e-05 | 3.0896373119871e-05 | 2.8780594031392e-05 | 2.76599478468852e-05 | 2.64641948937256e-05 | 3.0802103605661e-05 | 2.90485952422033e-05 | 2.99687639523779e-05 | 3.03876586517139e-05 | 2.7568468786667e-05 | 2.94910524081698e-05 | 2.54741952418978e-05 |
| pos\_1410 | S-Japonin | 9.71708229583566e-05 | 0.000111902191277403 | 0.000114885981956088 | 0.000102064926532246 | 8.38251442898298e-05 | 0.000106638841296889 | 6.79614593548627e-05 | 9.64876052526072e-05 | 0.000109279891068432 | 7.9983547623316e-05 | 0.00010375299449096 | 0.000110555795503347 |
| pos\_1411 | Carvyl acetate | 0.000281282565115092 | 0.000317042029006708 | 0.000299938339572597 | 0.000310218203202648 | 0.000256934178059219 | 0.000291235701865309 | 0.000285782633120436 | 0.000303737549476096 | 0.00029386581841357 | 0.000287184828049044 | 0.000298838582123826 | 0.00029298945854252 |
| pos\_1412 | N-[(4E,8Z)-1,3-dihydroxyoctadeca-4,8-dien-2-yl]hexadecanamide 1-glucoside | 6.33368410011311e-05 | 7.32880846793761e-05 | 0.000306931813704299 | 8.93346301373953e-05 | 6.37478113824632e-05 | 0.000102731766646412 | 5.07831606335262e-05 | 8.48761933690261e-05 | 6.4963332544345e-05 | 5.15622318717536e-05 | 8.61203090004835e-05 | 0.000191627615490944 |
| pos\_1413 | 3-Carboxy-2,3,4,9-tetrahydro-1H-pyrido[3,4-b]indole-1-propanoic acid | 1.68642401286173e-05 | 2.64248257985128e-05 | 2.66534190805228e-05 | 2.31610548018973e-05 | 1.83217033170653e-05 | 2.25747690951709e-05 | 1.90531723205353e-05 | 2.24639456198562e-05 | 2.45926285021102e-05 | 2.2028479153388e-05 | 2.09168980885557e-05 | 1.89355672565866e-05 |
| pos\_1414 | Temsirolimus | 4.44252181290654e-06 | 8.51425680552953e-06 | 1.06058280130606e-05 | 5.87255973595387e-06 | 2.77903257403155e-06 | 8.88042792350293e-06 | 5.0902754262607e-06 | 9.50519036546963e-06 | 5.39640316676741e-06 | 8.23997367213829e-06 | 1.08536583522035e-05 | 1.47977859059105e-05 |
| pos\_1415 | trans-S-(1-Propenyl)-L-cysteine | 1.00962632234265e-05 | 1.39767077848365e-05 | 9.74432823574698e-06 | 1.33766721863117e-05 | 2.03520517645275e-05 | 1.55234300980372e-05 | 1.55987702967491e-05 | 1.30098164449847e-05 | 1.73725105071152e-05 | 2.06846410137802e-05 | 1.43451283263212e-05 | 1.24310471306385e-05 |
| pos\_1416 | Ustiloxin A | 1.95941392294346e-05 | 2.15490776012567e-05 | 2.053886257447e-06 | 2.06806474879969e-05 | 1.23202706812642e-05 | 1.45556154669689e-05 | 1.09096801294586e-05 | 5.99054979302188e-06 | 1.99129687168756e-07 | 2.87668817690254e-05 | 8.11719767153633e-06 | 2.09524117012354e-06 |
| pos\_1417 | Ursonic acid | 0.00372727035375964 | 0.00208588783256993 | 0.00229606312251254 | 0.00142193192308578 | 0.00111634189418271 | 0.00153883988076728 | 0.00178833742837957 | 0.0016410687015265 | 0.0019664402654644 | 0.00152948243871405 | 0.00153920222350909 | 0.00218104526261526 |
| pos\_1419 | Lysyl-Valine | 5.80926537704282e-07 | 4.16649503382433e-07 | 0 | 5.61097088673743e-07 | 1.57590578792141e-07 | 4.52259700699055e-07 | 1.09832503656776e-06 | 7.60868640609944e-06 | 1.05115872883216e-06 | 1.0193306834001e-06 | 6.79210217549946e-06 | 1.48362709209945e-05 |
| pos\_142 | Kazinol E | 0.000260933917392523 | 0.000316028710731955 | 0.000330874953366578 | 0.000280656893341279 | 0.000232463031851669 | 0.000265509146408973 | 0.000271853485133268 | 0.000298372154618967 | 0.000294922222505039 | 0.000286142502239049 | 0.000268102298470207 | 0.00028395320253425 |
| pos\_1421 | DG(22:1(13Z)/22:6(4Z,7Z,10Z,13Z,16Z,19Z)/0:0) | 0.00194937887700943 | 0.00298057290385279 | 0.00258016317010493 | 0.00257125254207051 | 0.00203883161756703 | 0.00240836535992201 | 0.00218715896937978 | 0.00224942362537646 | 0.00275121162099079 | 0.00210398624717277 | 0.002359164166568 | 0.00201890870935798 |
| pos\_1422 | Ciprofloxacin | 2.50695161742949e-06 | 2.09143916317985e-06 | 2.14831770818233e-06 | 9.70820131563611e-07 | 2.91470428962713e-06 | 1.99563974116143e-06 | 2.51645980768433e-06 | 1.65445501434722e-06 | 2.16481332228005e-06 | 2.84881508724646e-06 | 1.52734191520034e-06 | 2.98233361373026e-06 |
| pos\_1423 | PI(16:0/20:2(11Z,14Z)) | 0.000451645536934755 | 0.000239086105730576 | 0.000223612594032521 | 0.000199709308635109 | 0.000258291427474514 | 0.000271430259227628 | 0.000283908063983365 | 0.000254363297639658 | 0.000253225245247888 | 0.000227181418422569 | 0.000250487190971992 | 0.000248414064673109 |
| pos\_1424 | Dimethylbenzyl carbinyl hexanoate | 2.61199187498536e-05 | 3.04675540844145e-05 | 3.89943592105319e-05 | 4.04256320262097e-05 | 3.99108058748449e-05 | 3.59464789884733e-05 | 3.76081749306729e-05 | 4.08795048943768e-05 | 3.7734694879279e-05 | 3.61454723192851e-05 | 3.65117649683873e-05 | 3.83577528051271e-05 |
| pos\_1425 | Hydroxyhomodestruxin B | 0.000895887155734537 | 0.000484960962334095 | 0.00042730325305655 | 0.000382482872206491 | 0.000440777250460541 | 0.000507367032503551 | 0.000524976442294909 | 0.000488777860998454 | 0.000475527515127428 | 0.000435278333814951 | 0.000460005126719515 | 0.00046052840491012 |
| pos\_1426 | DG(20:5(5Z,8Z,11Z,14Z,17Z)/22:0/0:0) | 4.55946621097985e-06 | 4.48234601909865e-06 | 2.23662808495101e-06 | 0 | 1.13876770129807e-06 | 0 | 8.87681700629777e-07 | 7.30840607897929e-07 | 0 | 1.5300939836399e-06 | 0 | 3.19222856908632e-06 |
| pos\_1427 | Erythrohydrobupropion | 4.80657260613842e-05 | 9.05271118975394e-05 | 5.94553037187002e-05 | 4.30059997401419e-05 | 6.46634519806556e-05 | 6.45324593104561e-05 | 4.146495687853e-05 | 5.48972544949759e-05 | 2.99283787918882e-05 | 9.57284104939816e-05 | 5.95807425554096e-05 | 8.05936269724254e-05 |
| pos\_1428 | Phenylalanyl-Valine | 0.000274735337700586 | 0.000325439656776066 | 0.00014958567199912 | 0.000213165716057321 | 0.000316867269241765 | 0.000260191586113282 | 0.000378162369558615 | 0.000272677800874275 | 0.000195171005299132 | 0.00026151702435539 | 0.000265986437914571 | 0.000197094135900606 |
| pos\_1429 | 5-Taurinomethyl-2-thiouridine | 1.98406885052625e-07 | 0 | 3.64036886042437e-05 | 5.41765593965526e-06 | 7.13762865769611e-07 | 5.94792938132178e-06 | 1.37749174347705e-05 | 1.21149173586827e-05 | 2.07764125951192e-06 | 4.18241254994777e-07 | 9.63720132814341e-06 | 1.47889056684228e-05 |
| pos\_143 | 4-Propylphenol | 1.78411908643306e-05 | 4.77748789948398e-05 | 2.40252147261781e-05 | 1.85515719120548e-05 | 3.57938920748589e-05 | 2.70576283803588e-05 | 2.40140879105685e-05 | 2.31592963061077e-05 | 1.89432357989266e-05 | 3.64271560192121e-05 | 2.26742491684904e-05 | 2.1171355080781e-05 |
| pos\_1430 | PC(20:4(5Z,8Z,11Z,14Z)/18:0) | 3.04019721285508e-05 | 1.13200268662948e-05 | 1.27021904646864e-05 | 5.51533224624606e-05 | 6.0165930369644e-05 | 2.21702668495888e-05 | 5.34315531671597e-05 | 2.23622650244671e-05 | 8.1694445830069e-06 | 1.38327138714235e-05 | 2.21562653149535e-05 | 6.44357210471336e-06 |
| pos\_1431 | Niazimicin A | 0.000139478196403485 | 0.000140232466046002 | 0.000145264041795434 | 0.000132751667076805 | 0.000135631218984116 | 0.000140809495101008 | 0.000101994699532006 | 0.000134225701024944 | 0.000158291902281442 | 0.000118750350793208 | 0.000133975134490186 | 0.000160657068716216 |
| pos\_1432 | Goshonoside F3 | 5.59293455279314e-05 | 7.65688896979071e-05 | 8.25044088866702e-05 | 7.0393814916351e-05 | 7.42032617588857e-05 | 7.12186968812788e-05 | 6.05704270500826e-05 | 5.95658368497939e-05 | 6.17093190887472e-05 | 5.44749631189395e-05 | 7.04236711333495e-05 | 6.50439455868172e-05 |
| pos\_1433 | 3-Hydroxy-1-phenyl-1-hexadecanone | 0.000518425994795781 | 0.000235444025996873 | 0.000301407774482569 | 0.000254614809597739 | 0.000351826523135804 | 0.000367281203785349 | 0.000159529846376317 | 0.000248133412945093 | 0.000233289164279591 | 0.000142679032145728 | 0.000309627291370091 | 0.000241269437658232 |
| pos\_1434 | Salviaflaside | 0.000992764592237792 | 0.000973770939158533 | 2.34873176818525e-05 | 0.000593033460751 | 0.00105425588001819 | 0.000695105137800081 | 1.786934163129e-05 | 1.93499548715374e-05 | 1.74591784618121e-05 | 0.000683988168007077 | 0.000142304531724802 | 4.16647037278124e-05 |
| pos\_1435 | Olopatadine n-oxide | 6.86725863378854e-06 | 5.21884366312044e-06 | 1.33767283311509e-05 | 2.86000372497837e-06 | 4.14455991407715e-06 | 1.23701968629965e-05 | 3.45687915362548e-06 | 3.42366351996854e-06 | 1.51298485756461e-05 | 1.48655250576978e-05 | 2.78613008268273e-06 | 4.3134747976523e-06 |
| pos\_1436 | Aflatoxin B1 dialcohol | 0.000445445989379767 | 0.000816052949230896 | 0.000408308757898333 | 0.000530585405319597 | 0.000650526124794848 | 0.000538791657779086 | 0.000438287992537197 | 0.000401869125262863 | 0.000755083222010888 | 0.000544121038486897 | 0.000540054385978997 | 0.000451979850919408 |
| pos\_1437 | (24E)-3alpha,15alpha-Diacetoxy-23-oxo-7,9(11),24-lanostatrien-26-oic acid | 0.00349889040344954 | 0.00495391871603615 | 0.0031719960686941 | 0.00290366999179442 | 0.0026390831179657 | 0.00325638390463585 | 0.00274525396830345 | 0.00308464343475361 | 0.00346925739161279 | 0.00292436426394281 | 0.00316419591273775 | 0.00282479567109977 |
| pos\_1438 | Isometheptene | 9.73542520159421e-05 | 0.00010437843257906 | 3.76651217656453e-05 | 0.00015091296186761 | 8.08352729451344e-06 | 7.10072129077629e-05 | 9.69744316466603e-05 | 6.70861878063754e-05 | 1.17726796441373e-05 | 0.000158100604737809 | 7.81340096292719e-05 | 4.23671815246446e-05 |
| pos\_1439 | Glutamyl-Phenylalanine | 0.000238292316763963 | 0.000286778574132965 | 0.000143006439524929 | 0.000217965631209045 | 0.000203191968138086 | 0.000208197110069296 | 0.000185575992501548 | 0.000167793897779353 | 0.000180060744194065 | 0.000238879577719562 | 0.000185992082461225 | 0.00016486322077723 |
| pos\_144 | Ethyl 3-(methylthio)propanoate | 7.22908400399026e-06 | 9.56182153976499e-06 | 4.45506246763293e-06 | 6.87363163275651e-06 | 5.69833954476718e-06 | 4.77117607453887e-06 | 6.67674925135422e-06 | 6.15271632854029e-06 | 6.53632366628727e-06 | 7.19579643800773e-06 | 6.29473807187495e-06 | 7.4912397116021e-05 |
| pos\_1440 | Ethyl glucuronide | 9.92559823142782e-06 | 1.50063257821066e-05 | 8.17576384341854e-06 | 1.26223372240964e-05 | 1.13281987567802e-05 | 1.05794357146965e-05 | 1.1412070638197e-05 | 1.04157601938623e-05 | 1.29342809857649e-05 | 1.17552234771556e-05 | 1.14817231237669e-05 | 7.96964130449787e-06 |
| pos\_1441 | 4-Deoxyannoreticuin | 3.67945462582968e-05 | 1.45047963159538e-06 | 1.29971125837907e-06 | 1.61094540375955e-06 | 1.85200085552307e-08 | 3.57192277937502e-07 | 7.06569193340622e-07 | 1.39613412817037e-06 | 3.06414344504459e-07 | 5.8290464824084e-07 | 7.87917344586548e-07 | 4.87147863099091e-07 |
| pos\_1442 | DG(16:0/18:1(11Z)/0:0) | 0.00047971170460823 | 0.00045598432015738 | 0.000505165499736643 | 0.000463929202416089 | 0.000325035648823167 | 0.000443763479555213 | 0.000372235343578963 | 0.000424816666680195 | 0.000508561403829601 | 0.000382181220930233 | 0.00042088198656314 | 0.000381436362981082 |
| pos\_1443 | Amikacin | 1.48026104285618e-05 | 5.84231531342804e-06 | 2.23080776978103e-05 | 3.82790248831603e-05 | 1.60217276313887e-05 | 2.04586130487497e-05 | 4.06747556049016e-05 | 2.36087572698905e-05 | 7.22383155703889e-06 | 2.42155795404148e-05 | 2.69849913803945e-05 | 1.44389440195688e-05 |
| pos\_1444 | Vanillic acid-4-O-glucuronide | 5.57019587481693e-05 | 8.43034361349733e-05 | 5.26199981646856e-05 | 7.70092838835187e-05 | 6.17190958427398e-05 | 6.18919721666037e-05 | 6.14017246035174e-05 | 6.20536332653763e-05 | 7.3051967367023e-05 | 6.80045064987503e-05 | 6.44656616997701e-05 | 4.69605016144348e-05 |
| pos\_1445 | Koryoginsenoside R1 | 0.00130947633871794 | 0.000540023530760108 | 0.000448677721101859 | 0.000457489961339681 | 0.00090791725863714 | 0.00091843260165972 | 0.00102428346833605 | 0.000732343403528805 | 0.00063761172322785 | 0.000731455414957518 | 0.000767404725912712 | 0.000758739382340593 |
| pos\_1446 | Alanyl-Hydroxyproline | 1.7382559074019e-05 | 2.49684940363453e-05 | 1.94663229958678e-06 | 1.62744431428987e-05 | 1.78264609152552e-05 | 1.34447859824585e-05 | 1.07028923273481e-06 | 1.7428096494242e-06 | 5.75051457643992e-07 | 2.70433295514558e-05 | 6.03490324589901e-06 | 3.22781196473988e-06 |
| pos\_1447 | 3-alpha-Androstanediol glucuronide | 0.00156016193484195 | 0.00146747546184149 | 0.00169604194534389 | 0.0019747651947715 | 0.00121521239066344 | 0.00160212775828501 | 0.0021028801180075 | 0.00328448839939032 | 0.00156393397906907 | 0.00154203061915042 | 0.00270236894317677 | 0.00629668142426586 |
| pos\_1448 | Valyl-Isoleucine | 2.1986586789826e-05 | 3.3555346675685e-05 | 3.84643472431875e-05 | 2.96412362651269e-05 | 8.18774435053564e-05 | 3.46249901711134e-05 | 3.1339622064845e-05 | 4.04414218554274e-05 | 6.18140091466301e-05 | 3.05969854256309e-05 | 4.06810974346567e-05 | 5.30272506197202e-05 |
| pos\_1449 | Isofucosterol glucoside | 0.000103243618503701 | 7.83937194420573e-05 | 1.96737453416548e-06 | 4.51110998867978e-06 | 1.92272262493809e-06 | 4.89880844634856e-05 | 3.78323754798253e-06 | 3.36367223595415e-06 | 3.37097580768196e-06 | 3.3183485001123e-06 | 2.95540915838002e-06 | 4.39617167286922e-06 |
| pos\_145 | Lactarofulvene | 2.28982074371825e-05 | 2.90895867919958e-05 | 2.99978350687773e-05 | 2.30648703536848e-05 | 2.01210731200835e-05 | 2.64766459691888e-05 | 1.99496003966507e-05 | 2.44689026269598e-05 | 2.21747618872663e-05 | 2.95228188180133e-05 | 2.62647899278384e-05 | 2.62308571327497e-05 |
| pos\_1451 | Polypodosaponin | 3.56497328012115e-06 | 4.90814608097661e-06 | 1.86369517319443e-05 | 0 | 4.17549076894032e-06 | 5.42184636354355e-06 | 0 | 1.21819993721312e-06 | 0 | 1.77536965426426e-05 | 3.07693996146021e-06 | 8.10847002664054e-06 |
| pos\_1452 | 2-Methyl-2-phenyl-undecane | 5.51703742215851e-05 | 6.06306328854297e-05 | 5.6012205209551e-05 | 6.07470863076723e-05 | 5.34374185239403e-05 | 6.14743794270948e-05 | 5.9223691788947e-05 | 6.21984171884682e-05 | 5.35041466488998e-05 | 6.2921727920686e-05 | 5.87521091165813e-05 | 5.90480604124604e-05 |
| pos\_1453 | Alliospiroside C | 1.8306323272196e-05 | 1.10269284012528e-05 | 2.53884692892191e-06 | 7.01990120642511e-06 | 5.1599383931059e-06 | 5.95249274722118e-06 | 5.61448628660907e-06 | 4.37392946335754e-06 | 2.76904833344519e-06 | 2.58362754015176e-06 | 4.44379067469533e-06 | 1.82181262946048e-06 |
| pos\_1455 | (E,E,E)-Sylvatine | 0.005156100627607 | 0.00212783895325885 | 0.00183007180226311 | 0.0017323425869552 | 0.00335436293290007 | 0.00335250719431764 | 0.00361139688880948 | 0.00272336836562363 | 0.00251631728188281 | 0.00264730476813082 | 0.00269549300920425 | 0.00272828947524702 |
| pos\_1457 | 2-Methyl-1-propanethiol | 3.87091255920347e-05 | 6.00497397677017e-05 | 4.02110838358465e-05 | 3.798429600696e-05 | 5.00701044968743e-05 | 3.92214519345502e-05 | 3.90871553295008e-05 | 4.00773050398752e-05 | 4.54491040055343e-05 | 3.75695925809917e-05 | 3.89317313851007e-05 | 4.42693179900766e-05 |
| pos\_1459 | Amitriptyline | 6.64360117622537e-05 | 6.22507220680008e-05 | 4.74664267894667e-05 | 5.17095859048296e-05 | 5.12544387240314e-05 | 5.33844626835507e-05 | 4.46652007767236e-05 | 4.45609595969707e-05 | 5.61927392043636e-05 | 5.33999646576313e-05 | 4.87146548623488e-05 | 4.58011059214922e-05 |
| pos\_146 | 5,4'-DIMETHOXY-7-HYDROXYFLAVONE | 9.76625929954469e-05 | 3.86868543343806e-05 | 9.24082529346755e-05 | 4.7513502483356e-06 | 4.5595000836264e-05 | 4.65300654160622e-05 | 5.90603197597709e-06 | 1.9084844394594e-05 | 3.08347191768599e-06 | 0.000101716819404861 | 1.98022489013428e-05 | 6.13341037351045e-05 |
| pos\_1460 | (S)-Pterosin K | 4.54610144685102e-05 | 4.7945878947958e-05 | 1.62533822646223e-05 | 3.25091546576116e-05 | 3.32445327157844e-05 | 3.28988709829566e-05 | 3.17115890652437e-05 | 2.70860477506513e-05 | 4.05826510189553e-05 | 2.20714053374937e-05 | 3.39689411279222e-05 | 3.02548413345463e-05 |
| pos\_1461 | 2-(3-Phenylpropyl)pyridine | 5.3326205846522e-06 | 6.55618294135143e-06 | 5.95783836523769e-06 | 6.96900780441084e-06 | 4.27802300596761e-06 | 4.58627987233314e-06 | 5.12351201604764e-06 | 3.90733545309731e-06 | 5.55656203694149e-06 | 5.42877377159833e-06 | 6.10279084062323e-06 | 4.48066913720659e-06 |
| pos\_1462 | 2-Ethyl-4-(2-furanyl)-2-propenal | 0.000204872605409028 | 0.000158115370892441 | 0.000178145241328375 | 0.000121565580424783 | 0.00015021976019087 | 0.000152839495749657 | 0.000143068379426793 | 0.000155916786264633 | 0.000137483439804246 | 0.000180031572890826 | 0.000157909877383695 | 0.000172132959541439 |
| pos\_1463 | Prenyl cis-caffeate | 5.85958398674742e-05 | 8.10545539611924e-05 | 7.44335507349324e-05 | 6.62756480912903e-05 | 5.1769911548028e-05 | 7.05155332193995e-05 | 5.84480292511759e-05 | 6.61591936682468e-05 | 7.22984296677582e-05 | 7.18723924305748e-05 | 6.57101708610646e-05 | 6.69181100265044e-05 |
| pos\_1464 | Carbetocin | 6.21371490972505e-06 | 5.59250355242077e-06 | 3.56283271844803e-06 | 4.81960336675853e-06 | 8.81003654408211e-06 | 3.56455484570823e-06 | 8.37116054566304e-06 | 6.33727530320368e-06 | 7.33154072938898e-06 | 3.69440358058765e-06 | 4.39168421865174e-06 | 3.95248606603502e-06 |
| pos\_1465 | Karpoxanthin | 0.000298060808034202 | 0.000308306461761247 | 0.000255507953473721 | 0.000343645639514947 | 0.000265572853307441 | 0.000294897199436826 | 0.000224279408882384 | 0.000245490462272402 | 0.000400266181295923 | 0.000286207950832796 | 0.000271319978024003 | 0.000250693841488546 |
| pos\_1466 | Kanzonol V | 0.000138208544290893 | 0.000140077398936398 | 0.000142303178596263 | 0.000133108624239859 | 0.00013184946281891 | 0.000142199695094175 | 0.000104764816016356 | 0.000133511589799841 | 0.000153843530469054 | 0.000117575990789082 | 0.0001329006510864 | 0.000150897844226701 |
| pos\_1467 | Cholic acid glucuronide | 4.28100064905182e-05 | 4.08901664113575e-05 | 4.04839096030536e-05 | 3.98647506577236e-05 | 5.18461174795863e-05 | 4.39497742271092e-05 | 4.45467876320196e-05 | 4.82523466275863e-05 | 3.0529355364323e-05 | 4.39450496795672e-05 | 3.72028512041229e-05 | 5.2833171980942e-05 |
| pos\_1468 | Neoherculin | 9.60217590147377e-06 | 4.72139950253401e-06 | 4.28652942458864e-06 | 4.66904378281165e-06 | 5.47798543949841e-06 | 5.29220971118143e-06 | 5.38919147097482e-06 | 5.019325573828e-06 | 4.89166009573375e-06 | 5.27248117713613e-06 | 5.34026852259473e-06 | 5.17301695293223e-06 |
| pos\_147 | Dehydroabietic acid | 0.000207236047757252 | 0.000257736506065935 | 0.000319044609836735 | 0.000232549643525521 | 0.000312166985305371 | 0.000286686212893575 | 0.000185098932974272 | 0.000236633200174777 | 0.000215253514544818 | 0.000371931292327055 | 0.000264588710186816 | 0.000353301359319051 |
| pos\_1470 | PE(20:5(5Z,8Z,11Z,14Z,17Z)/24:1(15Z)) | 1.6414578379195e-05 | 3.34050094705181e-06 | 2.61887049408084e-06 | 9.63116209112822e-07 | 2.13275026856456e-06 | 2.8342229877929e-06 | 3.98128342641029e-06 | 8.63017722765214e-07 | 1.8263074557716e-06 | 1.46412049644e-06 | 1.33541450169905e-06 | 2.08190489620223e-06 |
| pos\_1471 | Physagulin G | 1.46438064256234e-05 | 1.08812060232341e-05 | 1.30996860034014e-06 | 1.00327875855826e-06 | 3.41974589289367e-06 | 3.09327903662634e-06 | 2.30368107020059e-06 | 9.37266104570808e-07 | 2.46102018806218e-06 | 1.4030555041018e-06 | 7.74752185056753e-07 | 1.73466085655507e-06 |
| pos\_1472 | Isoglobotriaose | 2.52500111470752e-05 | 3.64373211673396e-05 | 1.81940356122449e-05 | 0.000175892880880259 | 1.69813549502235e-06 | 2.88221253113965e-05 | 0.000101004005435755 | 2.31192071436725e-05 | 6.87577522896817e-06 | 4.80485647129006e-05 | 4.73212504705734e-05 | 2.05567347971367e-05 |
| pos\_1474 | (9Z,11E,13E,15Z)-4-Oxo-9,11,13,15-octadecatetraenoic acid | 0.000212789327424455 | 0.000281871255343513 | 0.000161879732851776 | 0.000140547540676426 | 0.000188446318716434 | 0.000193700873417095 | 0.00016550239970144 | 0.00016340351707854 | 0.000144207198226718 | 0.000139649746321003 | 0.000155999443904217 | 0.00018071320645647 |
| pos\_1475 | 1-Hydroxy-1-phenyl-3-octadecanone | 0.000105164732454102 | 9.80306290752893e-05 | 0.000121108135287998 | 0.000166621446886194 | 0.000125492732283598 | 0.000155859884786584 | 0.000183840474654909 | 0.000201857729324394 | 0.000151906027425918 | 0.000155001770446212 | 0.00015254499069377 | 0.000147274719374718 |
| pos\_1477 | Resolvin D5 | 0.000129005200832969 | 0.000136502854032981 | 0.000130971552188565 | 0.000119017208555818 | 0.000104093384664551 | 0.000117494670676946 | 0.00010933142111952 | 0.000121708833105914 | 0.000131408879998542 | 0.000115871571164218 | 0.000119016804781645 | 0.000116414011651266 |
| pos\_1478 | 3-Hydroxy-cis-5-tetradecenoylcarnitine | 0.000229329175893584 | 0.000225211596956495 | 0.000288900978630953 | 0.000307627497903921 | 0.00143351542348622 | 0.000460757151839451 | 0.000252330031182567 | 0.000240728003620664 | 0.000661342331225688 | 0.000258406988620117 | 0.000342820334086978 | 0.000227824073088706 |
| pos\_1479 | Alanyl-Proline | 3.92895756361606e-06 | 5.30745971183094e-06 | 9.42881751523334e-06 | 4.89706594009595e-06 | 4.18228290751799e-06 | 5.89383479177933e-06 | 5.15154685150624e-06 | 5.68996200318646e-06 | 5.01558432588958e-06 | 6.36799650426316e-06 | 8.55061376521766e-06 | 6.65166644389425e-06 |
| pos\_148 | Manumycin A | 5.90634475165761e-05 | 6.04671858198486e-05 | 8.05875774831514e-05 | 9.48257431033798e-05 | 4.99388968878232e-05 | 7.35679929783673e-05 | 7.71322523439102e-05 | 0.000112896183002214 | 3.75915513611593e-05 | 4.98064658287855e-05 | 7.90600502633391e-05 | 7.21872230579102e-05 |
| pos\_1480 | Mesterolone | 1.39333330392685e-05 | 1.24164875430238e-05 | 1.25431662672957e-05 | 1.18332864083133e-05 | 1.38611699571701e-05 | 1.21679897725406e-05 | 1.14405555939765e-05 | 1.19097048775731e-05 | 1.09242611069736e-05 | 1.89601134794295e-05 | 1.1593548923851e-05 | 1.26539655704281e-05 |
| pos\_1481 | delta-Methylionone | 1.90243330382238e-05 | 2.27812108930566e-05 | 2.62212430282011e-05 | 2.7922854169156e-05 | 2.64565469560753e-05 | 2.28599633643146e-05 | 2.61152140212493e-05 | 2.30014130240343e-05 | 2.27791015332917e-05 | 2.42275709319252e-05 | 2.46108242613776e-05 | 2.74332684776845e-05 |
| pos\_1482 | Demissine | 8.27796422103389e-06 | 1.90739985362411e-05 | 2.51577947865528e-05 | 7.65960812633728e-06 | 1.00782480373674e-05 | 1.14407813776991e-05 | 1.33900549424563e-05 | 2.39381111218229e-05 | 7.94592011567484e-06 | 5.27483766294921e-06 | 1.72218301650656e-05 | 3.92852870264968e-05 |
| pos\_1483 | trans-O-Methylgrandmarin | 7.39775912285082e-06 | 6.9875062212539e-06 | 1.01852643574624e-05 | 1.06815058936513e-05 | 7.41080959072032e-06 | 9.23814304104522e-06 | 8.69172842030321e-06 | 9.67105837336489e-06 | 6.71805599406304e-06 | 5.76766521574105e-06 | 1.07342249392642e-05 | 1.4678905957615e-05 |
| pos\_1485 | 2-Isopropyl-1,4-benzenediol | 0.000963368577336913 | 0.000718579684531306 | 0.000403209377426308 | 0.000611554762024493 | 0.000677079152647821 | 0.000586276832969409 | 0.000630690128378299 | 0.000512212741820121 | 0.000390623784798205 | 0.000932277091783391 | 0.00058931543270564 | 0.000341436836942071 |
| pos\_1487 | 2,3-Diaminosalicylic acid | 8.78567505463626e-06 | 1.28739349931572e-05 | 9.96350243293132e-06 | 9.98862382779125e-06 | 8.19975153054298e-06 | 9.78438666035388e-06 | 8.46780995991721e-06 | 9.83025050377112e-06 | 1.02430647923677e-05 | 9.14031993224753e-06 | 9.40137899578068e-06 | 9.08912084597514e-06 |
| pos\_1489 | Docosanamide | 0.00313916288651984 | 0.00398619985902129 | 0.00381742394952307 | 0.00403161052308723 | 0.00268540416122214 | 0.0034613337414954 | 0.00345418569943483 | 0.00370945203799495 | 0.00338335997333663 | 0.00332229943091989 | 0.00358739068883766 | 0.00331794793294372 |
| pos\_149 | cis-Resveratrol | 5.37628225912502e-05 | 8.24045162090828e-05 | 5.19392129635753e-05 | 6.21763467805897e-05 | 4.96988331609571e-05 | 6.15458774188248e-05 | 5.80914236245346e-05 | 4.58661744649823e-05 | 5.50982688823776e-05 | 8.12520333572361e-05 | 5.86455517186285e-05 | 4.39440256785937e-05 |
| pos\_1490 | Tetrahydropersin | 5.52871745009169e-05 | 6.84348165333377e-05 | 8.703857841726e-05 | 8.04692875087803e-05 | 8.16375693645312e-05 | 7.42037706044718e-05 | 6.36482675971939e-05 | 6.26792758762834e-05 | 9.15535475530209e-05 | 8.1867674309806e-05 | 7.80022969858541e-05 | 6.79985649733955e-05 |
| pos\_1491 | 3b-Hydroxy-6b-(3-chloro-2-hydroxy-2-methylbutanoyloxy)-7(11)-eremophilen-12,8b-olide | 1.0119897651374e-05 | 7.12851333616228e-06 | 7.66695340153774e-06 | 6.30255888434136e-06 | 9.14356625304842e-06 | 7.1535619553953e-06 | 7.78568538625378e-06 | 7.8774049889834e-06 | 6.80022234885965e-06 | 8.58216636711882e-06 | 5.98947364126779e-06 | 1.06342199621841e-05 |
| pos\_1492 | TG(10:0/8:0/8:0) | 2.19480116756749e-05 | 3.19755118720681e-05 | 2.98468075325199e-05 | 4.74733861018259e-05 | 2.20192127940175e-05 | 3.34950691699162e-05 | 2.23721390076129e-05 | 3.23684760144076e-05 | 3.29706136245377e-05 | 1.92240518197116e-05 | 3.94272120821879e-05 | 3.21839595528772e-05 |
| pos\_1493 | TG(8:0/8:0/i-12:0) | 5.75915108150401e-05 | 6.01099723321237e-05 | 6.12099790902772e-05 | 6.00545821926813e-05 | 5.23096092752831e-05 | 5.91662032177553e-05 | 6.286621172343e-05 | 6.6248303263982e-05 | 6.08126089556001e-05 | 5.42999132754483e-05 | 6.46750009206818e-05 | 5.13654670270111e-05 |
| pos\_1495 | 2-methoxy-6-all trans-decaprenyl-2-methoxy-1,4-benzoquinol | 2.7239912664624e-05 | 2.85846513104778e-05 | 1.96528127412068e-05 | 2.92731877559642e-05 | 2.09381533267035e-05 | 2.12336178709015e-05 | 8.08308470322769e-05 | 3.70136084881363e-05 | 3.9404555727847e-05 | 3.4881334148068e-05 | 3.97719851330087e-05 | 1.63648102538405e-05 |
| pos\_1497 | Isorhamnetin 3-O-[a-L-rhamnopyranosyl-(1->3)-a-L-rhamnopyranosyl-(1->6)-b-D-glucopyranoside] | 6.19516993494602e-06 | 3.22785758487621e-06 | 5.22372575260606e-06 | 2.96358556286128e-06 | 7.93883681957811e-06 | 6.84888620712267e-06 | 5.29693059642305e-06 | 7.70211122500004e-06 | 4.66206034419028e-06 | 6.14939604735114e-06 | 4.84448827393792e-06 | 2.06314962365794e-05 |
| pos\_1498 | PC(DiMe(11,5)/MonoMe(11,3)) | 0.00154321226296999 | 0.000502798033225409 | 0.00048198643366335 | 0.000432273652509745 | 0.000691663290622983 | 0.00066024590127428 | 0.000746646572911972 | 0.000616933118365105 | 0.000585960900992651 | 0.000575049524256641 | 0.00061983064441119 | 0.000588275225379627 |
| pos\_1499 | Flavone | 9.19683321576528e-05 | 0.000124099672107564 | 4.12478883768456e-05 | 0.000105732765702879 | 3.55194049989861e-05 | 7.24948791448637e-05 | 8.56049286429558e-05 | 7.07155151790364e-05 | 3.56432085555882e-05 | 0.000110535630088319 | 8.02109082118578e-05 | 4.73955682497099e-05 |
| pos\_15 | Guanine | 0.000679697857844718 | 0.000858472194877804 | 0.000475416156282614 | 0.000490930418124927 | 0.000686524604643087 | 0.000433770694859097 | 0.00073354935723783 | 0.000685171865404108 | 0.000586239526709773 | 0.000777522421335236 | 0.000577880334397036 | 0.000755436383509417 |
| pos\_150 | Lentiginosine | 0.000192763131508112 | 0.000206350176913844 | 0.000128270966574903 | 0.000563023950820582 | 0.000204210143487219 | 0.000238873144141349 | 0.000593640186752635 | 0.000448896660910875 | 0.000149773700879272 | 0.000925177211819921 | 0.000554984502064514 | 0.000216486927404874 |
| pos\_1500 | Aesculin | 0.00033124020237564 | 8.67547420488175e-05 | 9.48328940475704e-05 | 7.83341752839285e-05 | 0.000188776285416121 | 0.000190774859724975 | 0.000168997444165292 | 0.000150224454754005 | 0.000109718832698371 | 0.000129961853244618 | 0.000145030862158755 | 0.000136575790919278 |
| pos\_1501 | (24E)-15alpha-Acetoxy-3alpha-hydroxy-23-oxo-7,9(11),24-lanostatrien-26-oic acid | 0.00190605191067706 | 0.0013311021915979 | 0.00155192141578759 | 0.00199358203114592 | 0.00148418591938345 | 0.00222551463266763 | 0.00172463882022271 | 0.0015185373805459 | 0.00173508155807356 | 0.00160065530332431 | 0.00215840120028248 | 0.00143952452784144 |
| pos\_1502 | Ganglioside GA2 (d18:1/16:0) | 0.000117592607182848 | 0.000155476740368967 | 0.000124430243566759 | 9.96551733646186e-05 | 7.99604501454314e-05 | 8.16356293165536e-05 | 0.00011260425675157 | 0.000141539632725705 | 0.000199457001047959 | 0.000139928231633722 | 0.000145031723297999 | 0.000144527328613111 |
| pos\_1503 | PE(22:2(13Z,16Z)/22:6(4Z,7Z,10Z,13Z,16Z,19Z)) | 0.00160107828365188 | 0.000728358511860144 | 0.000707001611785195 | 0.000612906062581029 | 0.000782071701454471 | 0.000831871557622864 | 0.00087105767926529 | 0.000768402602682283 | 0.000755037212256931 | 0.000717713827677062 | 0.000782846463758418 | 0.000760173010304113 |
| pos\_1504 | Cadinene | 7.38949305335764e-05 | 9.79403143741926e-05 | 9.49905850998644e-05 | 0.000102614174409837 | 7.45802823787135e-05 | 8.5723039180781e-05 | 8.42063054668049e-05 | 9.43247731020852e-05 | 7.87352046052687e-05 | 9.55154004910622e-05 | 8.36975832310842e-05 | 9.20258778391244e-05 |
| pos\_1505 | 3alpha-Corosolic acid | 0.000118177169509134 | 8.73884620356105e-05 | 9.40016214440017e-05 | 0.000151910913608394 | 0.000101940802446945 | 0.000109612154330245 | 0.00021184812853473 | 0.000511539106952053 | 9.99531879871171e-05 | 0.000141457727865477 | 0.000266890892485841 | 0.00076093931167674 |
| pos\_1506 | OR-1855 | 0.000939847354119013 | 0.000544039790372981 | 0.000520244460306078 | 0.00045764104574574 | 0.000561602080917343 | 0.000582349583590377 | 0.000613898926810313 | 0.000575850963290809 | 0.000580825702693234 | 0.00052343245699527 | 0.00055736473345237 | 0.000558770061883352 |
| pos\_1507 | Ginsenoside Rh3 | 0.00155526959894682 | 0.0010007284200908 | 0.000911204910713631 | 0.000848539298201163 | 0.000998366486985496 | 0.00108399403860617 | 0.00105164158006616 | 0.000986421926605423 | 0.00104540165468193 | 0.000922293938309687 | 0.000992944075499502 | 0.000930611546941604 |
| pos\_1508 | Telithromycin | 0.000498530076441526 | 0.000281718992300684 | 0.000186915051289055 | 0.000133571647444143 | 0.000219020988224835 | 0.000210746920444462 | 0.000192305612589844 | 0.000162819423240384 | 0.000195189995441558 | 0.000146445622492147 | 0.000172534761031269 | 0.000167892930077103 |
| pos\_1509 | PE(22:5(4Z,7Z,10Z,13Z,16Z)/dm18:0) | 5.1285710602548e-06 | 3.92383898305321e-06 | 0 | 1.24640828015713e-06 | 2.67611363850322e-06 | 3.55704313657996e-06 | 0 | 9.17865890860539e-07 | 9.50334006318637e-07 | 0 | 0 | 7.97310652938733e-07 |
| pos\_151 | Moronic acid | 0.000756804250428318 | 0.000737862019337232 | 0.00126800868245409 | 0.00104682494616254 | 0.000800600685462132 | 0.000947656160026501 | 0.00133962676675956 | 0.00117325082031878 | 0.000708624625387625 | 0.000946509555845842 | 0.00109488267053375 | 0.0009566437225474 |
| pos\_1510 | DG(20:5(5Z,8Z,11Z,14Z,17Z)/22:6(4Z,7Z,10Z,13Z,16Z,19Z)/0:0) | 6.42549176166961e-05 | 0.000258766900997378 | 0.000143091571987829 | 3.48875474689253e-05 | 1.86164153880705e-05 | 9.80390082959116e-05 | 4.69481804699171e-05 | 6.50737532526035e-05 | 5.92339111182418e-05 | 8.35927026945196e-05 | 6.12873674816218e-05 | 9.58426618909603e-05 |
| pos\_1511 | Cinchonain Id 7-glucoside | 0.000452984621595739 | 9.049780146076e-05 | 0.000111109385859455 | 8.33759019852527e-05 | 0.000251093114183514 | 0.000246923347255092 | 0.000216610879937505 | 0.00019577081942602 | 0.000138500815259053 | 0.000150031126592963 | 0.00018147440501759 | 0.000161289454719068 |
| pos\_1512 | Paxilline | 0.00100036215617496 | 0.000389735106748937 | 0.000345306973184229 | 0.000313856584173827 | 0.000695383932958468 | 0.000698937648886703 | 0.000752736648895307 | 0.000524128928858591 | 0.00053085630726788 | 0.000511000207707165 | 0.000556353548557317 | 0.000542118733327973 |
| pos\_1513 | L-Thyronine | 8.72882580079925e-05 | 9.33453320689016e-05 | 3.0602974589997e-05 | 7.58821820421707e-05 | 4.89686036088167e-05 | 6.21549218728722e-05 | 5.01233684313944e-05 | 3.90934469985783e-05 | 2.04472037642368e-05 | 8.40518385153635e-05 | 4.93028639168345e-05 | 2.64299662797725e-05 |
| pos\_1515 | Ubiquinone-2 | 3.3505787445601e-05 | 3.42558654384758e-05 | 1.96189722936115e-05 | 2.03076257564728e-05 | 2.05125415776946e-05 | 2.51346218925268e-05 | 2.09257406550205e-05 | 2.32326417302956e-05 | 3.24863454422449e-05 | 2.21115084508951e-05 | 2.6982864646933e-05 | 3.37852475498052e-05 |
| pos\_1517 | Dethiobiotin | 1.43573833160033e-05 | 1.41966680311024e-05 | 1.1009524057577e-05 | 1.09542792590636e-05 | 1.24007991957222e-05 | 1.22123700813334e-05 | 1.22348578331371e-05 | 1.09453141585854e-05 | 1.12793509666618e-05 | 1.23548163401998e-05 | 1.08193554738623e-05 | 1.17028687112189e-05 |
| pos\_1518 | L-phenylalanyl-L-proline | 1.79545154711262e-05 | 2.05983539136477e-05 | 2.11712509555619e-05 | 2.32530515150676e-05 | 2.02694449756663e-05 | 2.02506065358722e-05 | 2.23557233997376e-05 | 2.10440514564414e-05 | 2.06442634975489e-05 | 1.92052523023621e-05 | 1.87473572378072e-05 | 1.79084418817951e-05 |
| pos\_152 | Biliverdin IX | 0.000302935150633915 | 0.000354951659676516 | 0.000237877699987874 | 0.000276243528592757 | 0.000370415801642368 | 0.000327236369101438 | 0.000296712847219435 | 0.000265650767694174 | 0.000255703986782533 | 0.00032569825387281 | 0.000269735498348561 | 0.000275135280819351 |
| pos\_1521 | Matesaponin 3 | 0.000739775453151963 | 0.000508532880000026 | 0.000552669162302267 | 0.00044999139792144 | 0.000822901104095299 | 0.00060165492178201 | 0.000748135988216084 | 0.000638828051138164 | 0.000480559470717719 | 0.000691087389721162 | 0.000429389103433364 | 0.000719619712491353 |
| pos\_1522 | DG(14:0/16:1(9Z)/0:0) | 1.77858626200862e-05 | 4.53097073081658e-07 | 1.40907827373731e-06 | 1.23231484394912e-06 | 8.06467078286858e-06 | 6.30287054124413e-06 | 3.48880159467498e-06 | 2.26998001313084e-06 | 1.90586330109934e-06 | 2.44601158664826e-06 | 5.45788684047187e-06 | 2.12460372611107e-06 |
| pos\_1523 | 3-Hydroxyhexadecanoylcarnitine | 9.85984422473146e-05 | 4.20973635087766e-05 | 2.51247022282717e-05 | 2.14074501152174e-05 | 4.2851998915324e-06 | 3.87081265495862e-05 | 1.98120512525695e-05 | 8.02993743649772e-06 | 4.08518285705479e-05 | 0.000192176802880356 | 4.43451116017082e-05 | 1.76293984375643e-07 |
| pos\_1524 | Piperoic acid | 3.08321877691417e-05 | 2.73504167772727e-05 | 4.32974316242995e-05 | 4.57448797987397e-05 | 1.7467361949402e-05 | 2.98656340679874e-05 | 3.74131948632115e-05 | 2.88066289829039e-05 | 7.15485054335662e-05 | 3.75222609887933e-05 | 3.80717718473687e-05 | 2.30410664437645e-05 |
| pos\_1525 | Propylene glycol alginate | 9.04275346891121e-06 | 9.76067837167067e-06 | 6.6960510921941e-06 | 5.53026156787633e-06 | 1.01190940769255e-05 | 6.47366813049021e-06 | 1.33780050411634e-05 | 1.03411199497976e-05 | 7.7777650873554e-06 | 1.05333546232825e-05 | 7.8388282003383e-06 | 1.5000174217554e-05 |
| pos\_1526 | Polyporusterone B | 0.00318087224331394 | 0.00143405500368446 | 0.00132416431835951 | 0.00119438592007317 | 0.00209202872790322 | 0.002084593486621 | 0.00225717812468318 | 0.00178852783212265 | 0.00167254559005677 | 0.00162155728494933 | 0.00175517548819204 | 0.00178998331672538 |
| pos\_1527 | Pregabalin | 0.00048348993384729 | 0.000593097460802505 | 0.00122712310114696 | 0.000790306756348946 | 0.000418652037190582 | 0.000698672639808911 | 0.00067302857144053 | 0.000882091723524844 | 0.000632016229680207 | 0.00087342472016851 | 0.000865649591469124 | 0.00124315097052743 |
| pos\_153 | Ursolic acid | 0.00577117801889694 | 0.00552012078055313 | 0.00496568403988439 | 0.00523332699076843 | 0.00403743807541336 | 0.00494695449536953 | 0.00594726241609609 | 0.00515955368850496 | 0.0047016175546309 | 0.00606020759701801 | 0.00508354603304198 | 0.00460152689799435 |
| pos\_1532 | Preisocalamendiol | 8.97341725473336e-06 | 7.46558908978184e-06 | 2.14095318087241e-05 | 4.55114316418157e-06 | 5.19785136868026e-06 | 8.75887542032555e-06 | 4.30929199250938e-06 | 9.4993215330757e-06 | 4.95553931704611e-06 | 1.46768658007626e-05 | 1.09114343685865e-05 | 1.765315185793e-05 |
| pos\_1533 | iso-A2E(11-cis) | 0.00048262597899549 | 0.000339142988314339 | 0.000365537662556899 | 0.000276775834023216 | 0.000209684071745277 | 0.0002853378994442 | 0.0002287505065789 | 0.000291480058073916 | 0.000255296709285971 | 0.0003118878298957 | 0.00028909076973166 | 0.000320231468753871 |
| pos\_1534 | 2-Methyl-3-(2-pentenyl)-2-cyclopenten-1-one | 3.63811072416151e-05 | 3.77794381751597e-05 | 3.25636457174487e-05 | 3.8198469205698e-05 | 3.45079780121485e-05 | 3.62583770274909e-05 | 3.83516385775183e-05 | 3.8820388195475e-05 | 3.851614028093e-05 | 4.01323976587732e-05 | 3.85132611496119e-05 | 3.64893783176681e-05 |
| pos\_1536 | Dihydroergotamine | 0.000112971069146283 | 0.000117583351568936 | 0.000116487037867756 | 0.000129980674789884 | 0.00013960918073186 | 0.000142074178189556 | 0.00018209420710565 | 0.00014285371145522 | 0.000100615914113628 | 0.000162627956006422 | 0.000147162877023359 | 0.000125881617511946 |
| pos\_1537 | Lycopersiconol | 6.69914892044677e-05 | 6.36161497048928e-05 | 4.61037335326694e-05 | 6.25144763898645e-05 | 5.94923088277146e-05 | 5.99459165709283e-05 | 9.76846956783469e-05 | 7.56896442150788e-05 | 5.63250255097165e-05 | 6.3926018000137e-05 | 7.58366433480132e-05 | 4.09304236478106e-05 |
| pos\_1539 | alpha-Fluoro-beta-ureidopropionic acid | 1.13734088023686e-05 | 1.65022855804753e-05 | 1.38774773303446e-05 | 1.22681468944696e-05 | 1.21602707986316e-05 | 1.25120490543796e-05 | 1.16175131893683e-05 | 1.18434282500991e-05 | 1.36287740545991e-05 | 1.08659197155866e-05 | 1.1960528153888e-05 | 1.17847489963322e-05 |
| pos\_154 | Necatorine | 1.53299186434102e-05 | 3.07982392185398e-05 | 1.13864895241435e-05 | 5.00329796138122e-05 | 5.02041982577127e-05 | 2.89333784342312e-05 | 1.51556947143601e-05 | 1.75889247308192e-05 | 5.25771772526077e-05 | 2.96697786624641e-05 | 2.97709003508549e-05 | 2.85787428666506e-05 |
| pos\_1541 | Tryptophanol | 8.03977744000692e-05 | 0.000233285017239768 | 9.55104707771093e-05 | 7.36695479603395e-05 | 0.000171006875732191 | 0.000119498338724636 | 0.000102068345515496 | 9.38328176911695e-05 | 7.36429791710171e-05 | 0.000169609945148665 | 0.000102362622481194 | 8.94312854291287e-05 |
| pos\_1542 | Tetracosapentaenoic acid (24:5n-3) | 0.00155769508502495 | 0.00156978557554441 | 0.00226479464203812 | 0.00268382134059426 | 0.00321104065553672 | 0.00244517994331522 | 0.00195422953728463 | 0.00258598380515572 | 0.0029129294238698 | 0.00237871008952655 | 0.00258435916069421 | 0.00272957224881751 |
| pos\_1543 | Monomenthyl succinate | 0.000645905639795778 | 0.000512852989242155 | 0.00043279437335921 | 0.000463764579150755 | 0.000448383109395422 | 0.000493716087517378 | 0.000456705325816567 | 0.000475047516547374 | 0.000632503168250189 | 0.000362701897901769 | 0.000493114956877432 | 0.000472693004670614 |
| pos\_1544 | 4R,5R,6S-Trihydroxy-2-hydroxymethyl-2-cyclohexen-1-one 6-(2-hydroxy-6-methylbenzoate) | 9.9706469529894e-05 | 0.000157503145683247 | 9.76932032700066e-05 | 0.000173740420831154 | 0.00011680264824741 | 0.000134432222345831 | 0.000112473275592376 | 0.000116338775851633 | 0.000261054580911855 | 0.000108449144687068 | 0.000189154125465807 | 0.000173043998088926 |
| pos\_1545 | 2-(Arabinosylamino)-3-(glucosylamino)propanenitrile | 8.19418553666161e-06 | 1.02227646751622e-05 | 1.80430203678585e-05 | 9.10981777012089e-06 | 7.14304363414474e-06 | 9.13587373055323e-06 | 3.89148320799565e-06 | 1.20158869516469e-05 | 6.57955046599233e-06 | 2.26131332784054e-05 | 1.0006511196837e-05 | 1.89306803429876e-05 |
| pos\_1546 | 2'-Oxoaloesol 7-glucoside | 7.91942607584604e-06 | 5.26074150433464e-06 | 2.93358432022411e-06 | 9.57611083174629e-07 | 3.87515457683865e-06 | 4.54710221193541e-06 | 5.38230429134271e-07 | 5.37294853708217e-06 | 2.25451138498614e-05 | 2.09789890090295e-07 | 9.51978769078453e-06 | 1.424930432088e-05 |
| pos\_1547 | Gravacridonolchlorine | 7.51153619623032e-07 | 7.85243725189995e-06 | 8.6112947525965e-07 | 1.51726024243088e-06 | 5.835638015303e-06 | 3.08582658123483e-06 | 2.91848659052754e-06 | 2.28194215380504e-06 | 7.73887217390713e-06 | 2.18051813639932e-07 | 2.6907528660658e-06 | 6.53723192514468e-07 |
| pos\_1548 | Tertatolol | 6.34245424339913e-06 | 7.59131330330145e-06 | 5.93302887202048e-06 | 8.39518789700579e-06 | 1.41749938591243e-05 | 1.02325612456489e-05 | 1.09153552799492e-05 | 5.57577327042314e-06 | 8.75806339426587e-06 | 1.47338926367159e-05 | 9.91479263874482e-06 | 7.28223359620694e-06 |
| pos\_1549 | 4-Methyl-2-pentyl-1,3-dioxolane | 0.000367409891145014 | 0.000367570032899753 | 0.000527167251586901 | 0.000615208377537651 | 0.000492369989726271 | 0.000605351347258427 | 0.0006787652795462 | 0.000754160362633074 | 0.00063395215042419 | 0.000634031674178458 | 0.000575665738729368 | 0.000521891276911758 |
| pos\_155 | Alpha-Calacorene | 0.00012213912036194 | 0.000196715811787959 | 0.000238628403180554 | 0.000173217714633264 | 0.00011733676585209 | 0.000168264943496405 | 0.000205546423920098 | 0.00022266117923702 | 0.000214530516039301 | 0.000171396089046219 | 0.000222927567100571 | 0.000261807355813688 |
| pos\_1550 | (S)-3-Octanol glucoside | 2.79539595785827e-05 | 4.56161908281232e-05 | 3.24912946030436e-05 | 3.2906212295816e-05 | 3.77281558744027e-05 | 3.40009802319984e-05 | 3.38479870213846e-05 | 3.85907975583936e-05 | 3.36141330341347e-05 | 3.10892026501341e-05 | 3.35292143067039e-05 | 4.51459649326354e-05 |
| pos\_1551 | cis-3-Hexenyl b-primeveroside | 1.43222536176739e-05 | 9.9166692305305e-06 | 9.80907695063077e-06 | 1.0711699056726e-05 | 1.11747147044582e-05 | 1.1674018197449e-05 | 9.96073331806735e-06 | 1.13530521154881e-05 | 1.19255985736145e-05 | 8.4978345703292e-06 | 9.52819090663082e-06 | 1.40705334444275e-05 |
| pos\_1552 | 5-b-Cholestane-3a ,7a ,12a-triol | 7.88115603720309e-05 | 7.43497074135176e-05 | 7.76720233602588e-05 | 9.4451614577203e-05 | 0.000184621729597308 | 9.83062415813327e-05 | 6.54121329968684e-05 | 7.20416417522624e-05 | 0.000123509799300051 | 8.67427205998138e-05 | 8.40607794902039e-05 | 7.26088235135738e-05 |
| pos\_1553 | 3-Methylcyclopentene | 1.52042240466445e-05 | 2.1385675154906e-05 | 2.00121392070273e-05 | 1.5655738892449e-05 | 1.38880521974006e-05 | 1.71291579595866e-05 | 1.4145306226956e-05 | 1.65058002234726e-05 | 1.79384487764261e-05 | 1.61349408424922e-05 | 1.68630124610177e-05 | 1.51160673238928e-05 |
| pos\_1554 | 2-Hydroxylauroylcarnitine | 5.37432482965262e-05 | 3.91269092606239e-05 | 8.93817441365659e-05 | 5.08065590441961e-05 | 3.31518934462394e-05 | 5.9142154771679e-05 | 2.68664167226302e-05 | 3.19356723328974e-05 | 4.10659339223321e-05 | 7.30159072184593e-05 | 4.77409933942625e-05 | 4.36625081931601e-05 |
| pos\_1556 | 4b-Hydroxystanozolol | 5.68570358288097e-05 | 7.55204542455022e-05 | 0.000112858389423605 | 9.10867910581932e-05 | 6.13776549197161e-05 | 8.13009142468758e-05 | 5.96237272303631e-05 | 9.37503697112992e-05 | 6.36823545681576e-05 | 6.63898068995419e-05 | 8.1408704376118e-05 | 0.00012709794059251 |
| pos\_1557 | Mesuagin | 5.77662575100288e-06 | 2.7171963813625e-06 | 8.41618070734015e-06 | 7.10055688580617e-06 | 3.67692261918262e-06 | 4.84818962866377e-06 | 4.56834538368613e-06 | 5.20486591640956e-06 | 2.47426723899179e-06 | 8.25140318782153e-06 | 5.29026733233419e-06 | 8.2192762083177e-06 |
| pos\_1559 | L-Citronellol glucoside | 4.52291617573153e-05 | 6.69199366195705e-05 | 5.40081663170335e-05 | 3.83072052948338e-05 | 5.12880378651323e-05 | 5.11608518280147e-05 | 3.41055032826785e-05 | 4.36686740832197e-05 | 3.20186743573651e-05 | 9.17649619942896e-05 | 5.2064871285958e-05 | 6.52605445629934e-05 |
| pos\_156 | N,N-Dimethylethanolamine N-oxide | 7.12811894446391e-06 | 8.30617995789254e-06 | 9.67793316730731e-06 | 9.17053605149765e-06 | 6.94861968769878e-06 | 8.4298782372866e-06 | 9.53107194706139e-06 | 9.35614246585868e-06 | 8.36229886337797e-06 | 7.84225425650422e-06 | 8.42453412359562e-06 | 9.52015685965688e-06 |
| pos\_1561 | Eremopetasitenin B2 | 7.7823317372028e-06 | 1.0755756451776e-05 | 3.54341407333062e-06 | 6.81824519820132e-06 | 6.52400713151173e-06 | 5.16045248976173e-06 | 1.82376774281502e-06 | 1.55746163172678e-06 | 9.70414945874733e-06 | 5.39407816811216e-06 | 9.85455417069282e-07 | 3.29946266136884e-06 |
| pos\_1562 | Prostaglandin F1a | 5.52998123354008e-05 | 8.35900999331551e-05 | 7.71076759658416e-05 | 6.75917085917391e-05 | 5.85715455418072e-05 | 6.83089129006078e-05 | 5.41909956212396e-05 | 6.72299392954742e-05 | 7.89204526801208e-05 | 5.59996097694856e-05 | 6.60484728679815e-05 | 6.64758600494145e-05 |
| pos\_1563 | Glycinoeclepin B | 5.3489717754977e-05 | 5.52650476417317e-05 | 5.771873224126e-05 | 5.36763096923107e-05 | 5.74857116417559e-05 | 5.87550400700865e-05 | 4.74861500994766e-05 | 5.57289557682116e-05 | 5.99235677002829e-05 | 5.2478078783406e-05 | 5.3276188799121e-05 | 6.33314493040139e-05 |
| pos\_1565 | DG(18:1(11Z)/22:4(7Z,10Z,13Z,16Z)/0:0) | 1.37123127564489e-05 | 0.000157504185702642 | 2.15023568621184e-05 | 7.21058971178399e-06 | 3.24780659329387e-06 | 4.26662476184286e-05 | 3.32256172629368e-06 | 3.0226002917432e-06 | 3.24518959225514e-06 | 4.27168385839935e-06 | 4.63718660053309e-06 | 6.24164561155585e-06 |
| pos\_1566 | (-)-alpha-Narcotine | 2.8756706294032e-06 | 1.84229950731844e-06 | 4.93041664779372e-05 | 8.9780414563924e-05 | 6.45383773243398e-06 | 2.11375178548606e-05 | 7.71785811276086e-06 | 8.60559149714214e-05 | 1.65479972261727e-06 | 2.97375862164694e-06 | 3.59207419486262e-05 | 0.000182906415833266 |
| pos\_1567 | Neoacrimarine F | 0.000119561412929794 | 3.06131066431554e-05 | 3.51729142747158e-05 | 2.75569473936721e-05 | 7.12329626012113e-05 | 7.12030114746806e-05 | 6.47135606237066e-05 | 5.62945285307876e-05 | 4.58868265638038e-05 | 4.81228954097824e-05 | 5.64323372307722e-05 | 4.89684707958534e-05 |
| pos\_1568 | Cabergoline | 2.25109964855567e-05 | 1.24670446242854e-05 | 6.34210779782219e-05 | 2.36354871519745e-05 | 3.49026475413875e-05 | 0.000207081889978568 | 4.95448376511682e-05 | 5.22044684628868e-05 | 7.83767194189476e-05 | 0.000121003083800439 | 4.72761191163586e-05 | 3.26091882930914e-05 |
| pos\_1569 | Propoxyphene | 0.00560832226043319 | 0.00252651953479776 | 0.00228334756684678 | 0.00214102112447971 | 0.00372575356999332 | 0.00384294794364622 | 0.00411461275312139 | 0.00311287874382986 | 0.00306148188778012 | 0.00307684419515744 | 0.00313868081558733 | 0.00317269246559547 |
| pos\_157 | 3,6,9,12,15-octadecapentaenoic acid | 0.000186807393645338 | 0.000184851268244152 | 0.000339755768511375 | 0.000321710656587187 | 0.000398160022244346 | 0.00030987087132725 | 0.000269992956811034 | 0.000255140015417993 | 0.000350008319381872 | 0.000289760727843082 | 0.000296385993775979 | 0.000299799945642085 |
| pos\_1570 | Panaxynol | 0.000100114132013049 | 0.000160765651276873 | 8.471475325333e-05 | 8.5413747403304e-05 | 0.00011939130639797 | 0.000106759916475803 | 0.000112806718278201 | 8.95784411701687e-05 | 0.000127079880750226 | 0.000119447558377082 | 0.000103485265467269 | 7.52315142826712e-05 |
| pos\_1572 | (R)-Laudanidine | 0.000420899549046064 | 0.000224995017531134 | 2.11460843023292e-05 | 0.000125715761802576 | 0.000286487559230178 | 0.000210856228464749 | 2.27918021040984e-05 | 2.17310713916936e-05 | 2.08085554706771e-05 | 0.000119117176752181 | 4.83642476357147e-05 | 2.56210229887475e-05 |
| pos\_1573 | Nicotinuric acid | 7.39900254137842e-06 | 9.00038084332621e-06 | 6.40463882975908e-06 | 8.97271510644905e-06 | 9.1462677708159e-06 | 7.9687854834553e-06 | 9.4307195191991e-06 | 7.78261505232425e-06 | 7.76847053599923e-06 | 7.6425795734828e-06 | 7.43362012332862e-06 | 8.99414768810566e-06 |
| pos\_1574 | MG(0:0/20:3(11Z,14Z,17Z)/0:0) | 0.000275395978068345 | 0.000343749818629357 | 0.000347686981165068 | 0.000303482359268216 | 0.000249104034203198 | 0.000302505190312165 | 0.000268626748749635 | 0.000298911996494173 | 0.000341659833842891 | 0.000298256532538179 | 0.000281607312148153 | 0.0002828025909721 |
| pos\_1575 | 5,6-Dihydro-5,6-dihydroxy-y,y-carotene | 3.26295050276937e-07 | 0 | 4.77564251325971e-07 | 2.51858530274683e-05 | 3.44416419811438e-07 | 2.50355607685478e-06 | 2.06710705068851e-07 | 2.20542925337834e-06 | 0 | 0 | 0 | 8.12528849821173e-06 |
| pos\_1576 | Bn-NCC-1 | 3.32769288423287e-06 | 1.95997406956248e-05 | 7.68887407320849e-06 | 2.33707958397171e-05 | 8.25963900193273e-06 | 1.49599258827055e-05 | 2.12437561577371e-05 | 1.43893468558585e-05 | 1.60306318505991e-05 | 1.11323847032808e-05 | 1.41438878615305e-05 | 1.31674943857454e-06 |
| pos\_1577 | Octadecyl fumarate | 4.50761190582334e-05 | 5.3946012978328e-05 | 4.35945702685142e-05 | 4.54123496107853e-05 | 4.88751283236021e-05 | 4.73416620956311e-05 | 6.02851497753293e-05 | 5.47440409860796e-05 | 4.84007096586657e-05 | 4.49608579174202e-05 | 4.88395299688381e-05 | 4.23926429967971e-05 |
| pos\_1578 | Paramethasone | 6.61891043759364e-06 | 1.24377696659462e-05 | 1.29877700828047e-05 | 7.56582027671859e-06 | 8.31626859677461e-06 | 8.28111940882954e-06 | 6.22710686963737e-06 | 8.90402077392389e-06 | 1.2415752769592e-05 | 6.34103807306437e-06 | 7.67469774468486e-06 | 8.21658694413479e-06 |
| pos\_1579 | Ginsenoside Rh1 | 0.000256889827136537 | 9.59515759885036e-05 | 8.76836238098489e-05 | 9.28368006301119e-05 | 0.000120384592442054 | 0.000118150719877655 | 0.0001386323030964 | 0.000108918745479981 | 0.000102712869092841 | 0.000101762105892822 | 0.000109342559699374 | 0.000104498184678378 |
| pos\_1580 | Cytochalasin Ppho | 2.02231513768766e-06 | 5.85942586300369e-06 | 8.58159420779866e-06 | 2.80950331407087e-06 | 1.48545138810819e-06 | 2.7675325121469e-06 | 5.94030257461597e-07 | 2.42042217029902e-06 | 2.23592371688657e-06 | 2.0798195431665e-06 | 2.58804585484567e-06 | 1.43492734033384e-05 |
| pos\_1581 | Bicine | 3.32465260830242e-05 | 4.43551517234953e-05 | 6.01573571510839e-05 | 6.2727860533269e-05 | 6.77129142431036e-05 | 5.17899351532141e-05 | 6.11486570405317e-05 | 6.72517600660919e-05 | 5.31740606793428e-05 | 4.94444347734626e-05 | 5.47715157364273e-05 | 6.03420588372879e-05 |
| pos\_1583 | Adenosine thiamine diphosphate | 0.000110538770041842 | 3.70429024710394e-05 | 2.62784758635501e-05 | 2.68874386852305e-05 | 7.16706232597671e-05 | 6.11305609306422e-05 | 5.48430639860791e-05 | 4.71001467406968e-05 | 3.36862494711639e-05 | 4.89989394372847e-05 | 4.46460300737138e-05 | 3.97683634499194e-05 |
| pos\_1584 | N-Desmethyl-O-hydroxy rosiglitazone | 1.23919114923134e-06 | 4.94836916839181e-07 | 9.24369904152406e-06 | 4.20005541935442e-06 | 1.93711057715684e-07 | 2.14372710224146e-06 | 1.57377923848678e-06 | 1.09142629410219e-05 | 3.79149121931355e-06 | 5.1121475529999e-06 | 3.48680807247641e-06 | 3.48679662782461e-06 |
| pos\_1585 | Clozapine N-oxide | 5.62217009762899e-05 | 8.13321018175917e-05 | 4.98820178578077e-05 | 6.0507094328142e-05 | 6.02429590410528e-05 | 5.4546947671355e-05 | 5.63384365971026e-05 | 5.42279572007889e-05 | 6.06076717451043e-05 | 5.60964293630448e-05 | 5.7288828040887e-05 | 4.42153830561035e-05 |
| pos\_1586 | Momordin Ie | 5.81811040720406e-06 | 1.64746524336741e-06 | 3.98500723253615e-06 | 3.95224286929608e-06 | 5.01424239803321e-06 | 3.92671285420272e-06 | 5.83190709415525e-06 | 2.18854206820317e-06 | 2.49329087224965e-06 | 4.99696268081312e-06 | 5.55813578540371e-06 | 2.25716125548669e-06 |
| pos\_1587 | Citranaxanthin | 0.00799363296520638 | 0.0095927733102461 | 0.0161755212895619 | 0.0130461855660852 | 0.00968451990832075 | 0.011164745666345 | 0.0107797991828605 | 0.0124917206865548 | 0.0137371178822304 | 0.0121838170929562 | 0.0132692369971618 | 0.0154726534488106 |
| pos\_1588 | Americanin A | 0.000299001638564793 | 0.000703678033063322 | 0.000417483991614722 | 0.00028324510163985 | 0.000379281817548722 | 0.000393596118378069 | 0.000283097049232954 | 0.000353047963712016 | 0.00044327830663772 | 0.000451968912748708 | 0.000384619817565793 | 0.000420544629074801 |
| pos\_159 | 3-Phenoxypropionic acid | 0.000267085998283779 | 0.000253766721764984 | 0.000219091532630167 | 0.000197918336437227 | 0.000387835709291643 | 0.000205377130073441 | 0.00032102722446668 | 0.000294744358121307 | 0.000241163630919486 | 0.000305325728047262 | 0.000240489970750206 | 0.000331684523196313 |
| pos\_1590 | D-Leucic acid | 7.35008909937239e-05 | 4.30822588590672e-05 | 3.25275798175808e-05 | 3.20820776024331e-05 | 5.3054400642062e-05 | 4.982869749553e-05 | 5.58784924871053e-05 | 4.58752345517965e-05 | 4.26541026528532e-05 | 4.14271827572827e-05 | 4.33828638600441e-05 | 4.19381133903216e-05 |
| pos\_1592 | Momilactone C | 6.25832720459067e-05 | 6.09828200300735e-05 | 4.0290737017433e-05 | 4.56346265759407e-05 | 3.96746818633165e-05 | 5.83152882311266e-05 | 3.67073210793872e-05 | 4.46022484192763e-05 | 3.06009738363214e-05 | 5.17871069702495e-05 | 3.97842748559111e-05 | 4.75297064523908e-05 |
| pos\_1593 | PG(16:0/18:2(9Z,12Z)) | 0.00358197465816406 | 0.00206570760671435 | 0.00195240461451631 | 0.00168230169400267 | 0.00194893987871906 | 0.00198369739311698 | 0.0021192676902532 | 0.00193471938099015 | 0.00202101024305674 | 0.00180430118109081 | 0.00187766596805002 | 0.00190392706219286 |
| pos\_1594 | Kanokoside A | 2.24607244132842e-06 | 5.87124758376792e-06 | 8.17574005933286e-07 | 4.03714347340454e-06 | 1.17372832603651e-05 | 4.56412780325508e-06 | 8.52291121903629e-06 | 6.38198627546893e-06 | 4.30528390121334e-06 | 3.76614241623263e-06 | 5.51192736128855e-06 | 8.33522481992369e-06 |
| pos\_1595 | Triamcinolone | 4.23587054685506e-05 | 6.3638303863147e-05 | 3.3316144929404e-05 | 4.41608310269453e-05 | 5.66554328660266e-05 | 4.34642034285764e-05 | 5.16042126286092e-05 | 4.64061093969875e-05 | 4.06785590786299e-05 | 5.83688961645769e-05 | 4.25733788489232e-05 | 5.14717388543697e-05 |
| pos\_1597 | 3',5'-Diiodo-L-thyronine-beta-D-glucuronoside | 3.47221615334842e-05 | 1.882461933281e-05 | 1.66573930610963e-05 | 1.40716601011524e-05 | 2.13662551688396e-05 | 2.16912261764233e-05 | 2.11801862347038e-05 | 1.8712990434916e-05 | 2.22659158233891e-05 | 1.65698598387661e-05 | 1.6114329415782e-05 | 1.66601883040283e-05 |
| pos\_1598 | Cinitapride | 6.05416001138834e-06 | 1.13090867963499e-05 | 1.14109998629875e-05 | 7.97982561162873e-06 | 6.5219052401703e-06 | 7.97527382157739e-06 | 8.58831762322314e-06 | 8.75559483236435e-06 | 9.19343091892664e-06 | 8.02808948476939e-06 | 8.56899349422263e-06 | 7.10598469224083e-06 |
| pos\_16 | Rosmarinic acid | 0.000106549323105267 | 0.000152630676273147 | 7.66172275526354e-05 | 0.00011297110099191 | 9.70269591251537e-05 | 0.000107730813811139 | 9.52129694287776e-05 | 8.83630304853633e-05 | 7.71409622867316e-05 | 9.14951771930313e-05 | 8.44387760788476e-05 | 7.49904948636808e-05 |
| pos\_160 | Digeranyl | 1.25789557762962e-05 | 9.94107925631597e-07 | 4.84332043522883e-06 | 5.73116915222786e-06 | 7.82004673792524e-06 | 5.03615710442826e-06 | 2.20382177434675e-06 | 4.13421219777507e-06 | 4.72116212247764e-06 | 2.32450045588991e-06 | 4.07072254609721e-06 | 7.17590743767209e-06 |
| pos\_1600 | Galabiosylceramide (d18:1/20:0) | 0.000172176001606859 | 0.00016554193557608 | 0.000193453919316712 | 0.00183039845475258 | 0.000184884400322876 | 3.45536872216312e-05 | 0.000148951063190657 | 0.000156135676987071 | 0.000223630229684894 | 5.10408100647003e-05 | 4.64463013482697e-05 | 6.93661305190577e-05 |
| pos\_1601 | Prostaglandin E1 | 1.91593972699193e-05 | 2.38641258358305e-05 | 2.05176944393085e-05 | 2.00825220135139e-05 | 1.61592030643433e-05 | 2.06645577475575e-05 | 1.78624531565596e-05 | 2.1554358583368e-05 | 1.96380588935261e-05 | 1.91167914549937e-05 | 2.13174024148074e-05 | 1.75238617953666e-05 |
| pos\_1602 | (3S,5R,6R,6'S)-6,7-Didehydro-5,6-dihydro-3,5,6'-trihydroxy-13,14,20-trinor-3'-oxo-beta,epsilon-caroten-19',11'-olide 3-acetate | 0.000680035087823227 | 0.000767394285518406 | 0.00094253058130084 | 0.000731669822805304 | 0.0011029450373854 | 0.000761578112811825 | 0.00113160465649987 | 0.000843613470129495 | 0.000631118716047596 | 0.000694200079269839 | 0.000748061790490779 | 0.000824286672404965 |
| pos\_1603 | 1-heneicosanoyl-glycero-3-phosphate | 9.42844527616412e-06 | 9.62491307559328e-06 | 4.53952124822957e-06 | 5.38136178431674e-06 | 1.21484453391365e-05 | 9.29352485376643e-06 | 1.02377373795328e-05 | 1.35635712370537e-05 | 8.97440719709027e-06 | 1.1760934477947e-05 | 6.11081150915174e-06 | 1.46289139893164e-05 |
| pos\_1604 | PS(20:3(8Z,11Z,14Z)/18:0) | 0.000244067663619478 | 0.000174504920762197 | 0.000162110767413334 | 0.000147840718654545 | 0.000164376700427672 | 0.000181871357062615 | 0.00018143630882601 | 0.000172779290581625 | 0.000178730486502056 | 0.000159974316871194 | 0.000175905902776867 | 0.000168012748777542 |
| pos\_1606 | 9-Oxoasimicinone | 9.59560698251885e-06 | 5.23168316002417e-06 | 2.80385970353847e-06 | 1.31654607631304e-06 | 2.04777327338823e-06 | 8.31779586635507e-07 | 2.3061787551492e-06 | 7.09185036523205e-07 | 1.20249699866545e-06 | 4.97258196635482e-07 | 1.33427229552302e-06 | 1.54890931039916e-06 |
| pos\_1607 | Naringenin | 5.63174852543907e-05 | 3.97383309847816e-05 | 2.43081124628347e-05 | 2.50765861262354e-05 | 2.57773574427496e-05 | 3.28175833933744e-05 | 2.4018482673364e-05 | 5.13658638431454e-05 | 8.91669215447366e-05 | 1.65611102830575e-05 | 5.24246728191248e-05 | 9.4091609529793e-05 |
| pos\_1608 | 3-Methyl-2-cyclohexen-1-one | 1.07641129535574e-05 | 3.13081450404998e-05 | 1.55587512672173e-05 | 8.7036246128396e-06 | 1.68010559922686e-05 | 1.54439431194422e-05 | 1.29240570225116e-05 | 1.25784390820072e-05 | 8.46399490718897e-06 | 2.58095071685192e-05 | 1.45310466811327e-05 | 1.21965816923747e-05 |
| pos\_161 | 17-Hydroxylinolenic acid | 7.89481005169067e-05 | 8.4574127444939e-05 | 7.23647571538107e-05 | 8.72012637572123e-05 | 7.52126870360692e-05 | 7.63658937539096e-05 | 8.26695658547687e-05 | 8.29774248967317e-05 | 7.74996187063859e-05 | 7.27282872283349e-05 | 7.70777839100036e-05 | 8.42936269707808e-05 |
| pos\_1611 | TG(18:2(9Z,12Z)/14:0/20:4(5Z,8Z,11Z,14Z))[iso6] | 9.57141780985631e-06 | 2.51218084335653e-06 | 1.43977612697394e-06 | 1.186131088711e-06 | 2.01588530738649e-06 | 2.11610292482058e-06 | 2.35385674181386e-06 | 0 | 0 | 0 | 1.95313377470013e-06 | 5.23493790777615e-07 |
| pos\_1613 | Feruloyl-beta-sitosterol | 0.000282397329577954 | 0.000310546816488232 | 0.000246492109366684 | 0.000241776326991667 | 0.000258866032217042 | 0.000274943898255317 | 0.000253733056945859 | 0.000265549431241939 | 0.000273089548525294 | 0.000273829267690725 | 0.000263417352169935 | 0.000247197607711498 |
| pos\_1615 | Ganglioside GA2 (d18:1/25:0) | 2.97034406782987e-05 | 4.26751858501643e-05 | 3.63692192368714e-05 | 2.93955264217929e-05 | 3.13123528204733e-05 | 3.27922837013199e-05 | 2.96896753278467e-05 | 3.25527796682084e-05 | 3.74241661111108e-05 | 3.10429101299852e-05 | 3.39212074167424e-05 | 2.37789398308487e-05 |
| pos\_1616 | DG(14:0/20:5(5Z,8Z,11Z,14Z,17Z)/0:0) | 0.000352682469001256 | 0.000441885432569148 | 0.000927133460981403 | 0.000320119474223815 | 0.000473274608305006 | 0.000500635966465577 | 0.000530534624055912 | 0.000607434605271728 | 0.000388106533103674 | 0.000736191326838663 | 0.000843134583448218 | 0.000654212290703394 |
| pos\_1617 | 3a,20b-Pregnanediol | 3.16236185059429e-06 | 6.32058290304689e-06 | 1.1564157443689e-06 | 1.85388723885207e-06 | 1.01288886662699e-06 | 8.2463432631686e-07 | 1.21964978334529e-06 | 3.31712120714758e-06 | 1.22052989610274e-06 | 1.46397988794263e-06 | 1.25639659951134e-06 | 1.36317017472516e-06 |
| pos\_1618 | (1R,2R,4S,6R)-2,6-Fenchanediol 2-O-b-D-glucoside | 4.19644053786627e-06 | 5.64650553161975e-06 | 4.45305040887509e-06 | 4.04346061968461e-06 | 4.30605841059636e-06 | 4.72730808805617e-06 | 4.60795257094901e-06 | 3.4334910306479e-06 | 5.08278708000809e-06 | 4.29815972667049e-06 | 4.05802633115663e-06 | 5.16618888163797e-06 |
| pos\_1619 | LysoPC(20:0) | 5.47525504728951e-06 | 7.81402419860919e-08 | 1.07197648392311e-06 | 1.22792305766584e-06 | 2.92375407808484e-06 | 1.67764641876979e-06 | 3.09674757754416e-06 | 2.45883804999262e-06 | 1.16141578616859e-06 | 1.4242817037816e-06 | 1.25534951049541e-06 | 2.4840527512071e-06 |
| pos\_1621 | 4-(3-Pyridyl)-3-butenoic acid | 0.000112070737253826 | 6.61803270347901e-05 | 5.598866846837e-05 | 5.14717379069964e-05 | 7.99001818813745e-05 | 8.95628874411903e-05 | 9.22304183589085e-05 | 7.08732865615029e-05 | 7.36563586802849e-05 | 7.17370009932758e-05 | 7.37749564945971e-05 | 9.84213880841594e-05 |
| pos\_1622 | Dimethylamphetamine | 2.98187236064786e-05 | 4.91203788506302e-05 | 3.09617375828855e-05 | 2.43302893985713e-05 | 2.89325200807554e-05 | 3.21690000718588e-05 | 1.54686675384258e-05 | 2.41575652965338e-05 | 1.94499274950036e-05 | 3.70453378319352e-05 | 2.83226356938317e-05 | 4.56360140008576e-05 |
| pos\_1623 | Nonivamide | 6.35340978612345e-05 | 3.65764378166219e-05 | 1.4632278924909e-05 | 8.28638047619001e-05 | 1.38549669418466e-06 | 3.7152147158804e-05 | 2.15370733757334e-05 | 1.66368135511556e-05 | 4.55808533874801e-06 | 7.99201991972338e-05 | 2.56321313611458e-05 | 1.33097198084846e-05 |
| pos\_1624 | Janthitrem B | 0.000149029000104752 | 0.000111748707320662 | 9.76676222075067e-05 | 8.94228996275647e-05 | 9.57083184583631e-05 | 0.000110043152610107 | 0.000109706340186989 | 0.000106034933128472 | 0.000110171355698059 | 9.71058335463032e-05 | 0.000104337588335263 | 0.000101840377035803 |
| pos\_1625 | Ganoderic acid alpha | 4.23279831873222e-05 | 4.90376901774035e-05 | 5.89756677415335e-05 | 5.43774228263243e-05 | 2.86621710715897e-05 | 4.09791572549043e-05 | 3.51921345354418e-05 | 4.88508148337297e-05 | 5.18487900872888e-05 | 4.1125938820292e-05 | 4.5178908424475e-05 | 3.98436562606408e-05 |
| pos\_1626 | 6,8-Tricosanedione | 0.000128296888814361 | 0.000134483435930325 | 0.000141120252279814 | 0.000144478263331222 | 9.7014767292097e-05 | 0.000125317786909469 | 0.000125557323573814 | 0.00014256414317575 | 0.00013297951817512 | 0.000121466993667867 | 0.00013285525592535 | 0.000152999615859303 |
| pos\_1628 | (-)-Wikstromol | 8.36015418159192e-07 | 5.3941741910649e-07 | 1.64095145242075e-06 | 3.68810805778988e-10 | 9.68286533483116e-07 | 3.5112930338493e-07 | 5.05206278600753e-08 | 9.03042577753792e-08 | 1.94429339467174e-07 | 1.54713062525534e-06 | 8.09103145323347e-07 | 4.0622155153074e-05 |
| pos\_1629 | PE(18:4(6Z,9Z,12Z,15Z)/P-16:0) | 0.000199023354274727 | 0.000236699912332784 | 0.000216916735809925 | 0.000217102349369922 | 0.000173328560908109 | 0.0002131036716824 | 0.000190920512365926 | 0.000210697565912414 | 0.00022232976180513 | 0.000195410387299717 | 0.000206533524548154 | 0.000199014046756049 |
| pos\_163 | 2-Ethoxy-4-(methoxymethyl)phenol | 0.000154677524274375 | 0.000141002155800252 | 0.000212425013112057 | 9.04179474058488e-05 | 0.000149436927734999 | 0.000143293699323823 | 9.47509082247864e-05 | 0.000128508465401547 | 0.000103563951814578 | 0.00021802410902794 | 0.000149826536592861 | 0.000164095730975772 |
| pos\_1630 | PE(14:1(9Z)/22:1(13Z)) | 0.000164965874678097 | 0.000153469267772032 | 4.8914318304038e-06 | 2.62947519467306e-06 | 5.91474745947369e-06 | 7.32097858797043e-05 | 1.67414798942142e-06 | 0 | 4.46792369598116e-06 | 0 | 2.37792264427274e-06 | 1.9006238593453e-06 |
| pos\_1631 | Tracheloside | 1.66452373578376e-05 | 1.77956979358661e-05 | 5.34882567686472e-07 | 1.23479593262546e-05 | 1.28909158167391e-05 | 1.12371211042084e-05 | 5.92487308046816e-06 | 3.86411728399051e-06 | 2.21324170519567e-06 | 1.39265107170662e-05 | 7.46050978581358e-06 | 2.34637703859357e-06 |
| pos\_1632 | Physapruin B | 0.0196621530718676 | 0.012081738598319 | 0.011386381577055 | 0.0102584370355734 | 0.0117196476801887 | 0.0126345106777243 | 0.0126860163558693 | 0.0119041124200605 | 0.0124022990613122 | 0.0111396672651146 | 0.0119575395698322 | 0.0115861200037584 |
| pos\_1633 | Tetracosahexaenoic acid | 0.000159379367504349 | 0.000183401989900968 | 0.000154698471403379 | 0.000165046142573777 | 0.000213731043092909 | 0.000168610103711334 | 0.000318394322657193 | 0.000234845069770126 | 0.000193238291807224 | 0.000225713467567169 | 0.000193225132662388 | 0.000232211492262035 |
| pos\_1634 | Ustiloxin D | 0.000322314490112389 | 0.000278986620757007 | 0.000282167294121911 | 0.000236713031378756 | 0.000318645996362427 | 0.000300117359067059 | 0.000246096166259851 | 0.000283695664274312 | 0.000271489744994839 | 0.000259739079328867 | 0.000278558359206925 | 0.000379298873185858 |
| pos\_1635 | Physoperuvine | 2.5152093612814e-05 | 1.1239285310179e-05 | 9.21100074419592e-06 | 1.21216878139533e-05 | 1.84883315504883e-05 | 1.89497067481965e-05 | 1.41647722788119e-05 | 0.00010854589383931 | 1.44545904383333e-05 | 9.11434389196969e-06 | 2.53353333545625e-05 | 7.58540244361584e-06 |
| pos\_1636 | Clemastine | 7.31393086903216e-05 | 8.28802015039324e-05 | 3.43223581016808e-05 | 7.66851536073604e-05 | 4.99552057000779e-05 | 5.95051010062279e-05 | 6.47811874935401e-05 | 5.08029551131451e-05 | 4.75758354564678e-05 | 9.64038081574031e-05 | 5.92930485629737e-05 | 4.00301345873166e-05 |
| pos\_1637 | Jurubine | 0.000380332323064151 | 0.000564024648187336 | 0.0004528892472529 | 0.00047007748517538 | 0.000350375223114639 | 0.000516050059095745 | 0.000432392328253198 | 0.000508747117818037 | 0.00048140283866023 | 0.000391985131396682 | 0.000515946944796944 | 0.000426855941264943 |
| pos\_1638 | Medroxyprogesterone | 4.62082282165642e-06 | 7.88782391233393e-06 | 2.27317462473964e-06 | 3.08029667935298e-06 | 2.58274211590196e-06 | 3.28507832294601e-06 | 1.50005230396757e-08 | 1.26790864598689e-06 | 5.47319587381108e-06 | 2.06197923004617e-06 | 2.95892353530915e-06 | 1.23022942247728e-06 |
| pos\_1639 | (Z)-1,5-Tridecadiene | 2.20375012419654e-07 | 4.91313685478066e-07 | 8.09690350823846e-07 | 1.48391461418599e-06 | 7.55462601628791e-07 | 7.10097639153105e-07 | 1.23799679878923e-07 | 4.35734484718335e-07 | 8.42839280149113e-07 | 6.11823501749816e-07 | 3.55200149260156e-07 | 6.26577220993217e-07 |
| pos\_164 | delta3,5-Deoxytigogenin | 0.00014429204914987 | 0.000127758672645708 | 7.34291006266935e-05 | 0.000155827395256847 | 0.000116557046928398 | 0.000127960021843908 | 0.000166810355639073 | 0.000128430077174762 | 9.60295839375154e-05 | 0.000279338064091995 | 0.000154008559841529 | 0.000104258293628264 |
| pos\_1640 | Curdione | 0.000156550109802598 | 0.000209425324660752 | 0.000265044962585841 | 0.000216368092922744 | 0.000189179369953509 | 0.000208651292711416 | 0.000158548691953624 | 0.00021570604149642 | 0.00020780409084531 | 0.000180771522158723 | 0.000203911723894399 | 0.000263020881431366 |
| pos\_1641 | (3a,5b,7a)-23-Carboxy-7-hydroxy-24-norcholan-3-yl-b-D-Glucopyranosiduronic acid | 0.00109591638421683 | 0.000639156903715317 | 0.000876167474293206 | 0.000808159890270523 | 0.000717519419663392 | 0.000831303658877772 | 0.000931784038154627 | 0.000904758208538929 | 0.00062002096435547 | 0.000761048142123979 | 0.000824844512144125 | 0.000861023566658243 |
| pos\_1642 | Farnesiferol A | 0.000188190404887723 | 0.000250160072430365 | 0.000232520130119022 | 0.000218608809766453 | 0.000177840855389861 | 0.000210304823669846 | 0.00019544740465807 | 0.000217301756970972 | 0.000227513976616376 | 0.000199357203463169 | 0.000212925547718925 | 0.000204703001142343 |
| pos\_1643 | leukotriene-f4 | 0.00136558368433184 | 0.00112288270810912 | 0.00110718632956502 | 0.00117375777808562 | 0.00137908268799373 | 0.00143232484508309 | 0.00209937118917144 | 0.0015001887296781 | 0.00108708412526509 | 0.00168871281252921 | 0.00162841223424897 | 0.00143503074960802 |
| pos\_1644 | Soyasaponin A3 | 2.59582313932326e-05 | 4.25335591256605e-06 | 3.3953377746853e-05 | 1.40747292008032e-05 | 6.45860166166418e-06 | 2.26115925714578e-05 | 3.50285823757499e-05 | 3.03717792430619e-05 | 1.4354238348867e-05 | 2.33296503603823e-05 | 2.02690464406225e-05 | 2.22959317961755e-05 |
| pos\_1645 | Cyanidin 3-(6''-acetyl-galactoside) | 0 | 0 | 0 | 0 | 1.95660208234629e-05 | 1.63487232297772e-06 | 0.000172531415981951 | 0.000102641217331303 | 0 | 0 | 4.96795931878143e-05 | 2.91251027465481e-06 |
| pos\_1646 | (S)-Nerolidol 3-O-[a-L-rhamnopyranosyl-(1->2)-b-D-glucopyranoside] | 0.000125093462258903 | 5.07921955399136e-05 | 4.5012015369398e-05 | 3.90102319255918e-05 | 7.54777202942564e-05 | 7.74434296426746e-05 | 7.54167387217144e-05 | 6.28389542911405e-05 | 5.53473779009427e-05 | 5.64819706156489e-05 | 6.50453480593191e-05 | 6.27936995694222e-05 |
| pos\_1647 | clofarabind-5'-monophosphate | 9.21157385396969e-06 | 5.29076884541887e-07 | 4.10094324177461e-07 | 9.65395706192268e-07 | 4.27957049332872e-06 | 3.10469487821626e-06 | 3.20387159838713e-06 | 2.28806570864135e-06 | 1.81197151896192e-06 | 1.47898277579508e-06 | 2.25322985379714e-06 | 2.42540624836847e-06 |
| pos\_1648 | Madlongiside C | 0.000100620959773235 | 5.37207217997651e-05 | 3.95328431112611e-05 | 3.89203864234746e-05 | 6.47860650972345e-05 | 6.58723650229123e-05 | 6.95139617916968e-05 | 6.17906091993504e-05 | 5.51498049694056e-05 | 5.59812067694381e-05 | 5.67666552057748e-05 | 6.00227758342579e-05 |
| pos\_165 | 1-(4-Methoxyphenyl)-2-propanone | 0.000353011038432772 | 0.000256361877136096 | 0.000247308818224101 | 0.000215938648237596 | 0.000239781989303339 | 0.000248740563352113 | 0.0002575588917472 | 0.000250701871704514 | 0.000255878978663856 | 0.000247255884769618 | 0.000249128818532192 | 0.000250397201886985 |
| pos\_1650 | Laurotetanine | 4.17754905934843e-05 | 5.28676126261833e-05 | 3.16799116959406e-05 | 5.01418439340297e-05 | 4.52247893172907e-05 | 4.24352152758201e-05 | 4.07413740632583e-05 | 3.95459796145418e-05 | 4.40534826420378e-05 | 5.71261047642463e-05 | 4.98238509557601e-05 | 5.31323119719305e-05 |
| pos\_1651 | Levobunolol | 2.14796746432432e-05 | 1.95618571136604e-05 | 1.22211083507173e-05 | 3.89402509101125e-05 | 1.74477180946582e-05 | 2.10506792101879e-05 | 1.3345941559181e-05 | 1.18996835492435e-05 | 1.2853802632223e-05 | 1.81956248382584e-05 | 1.48261488998643e-05 | 1.63555907943916e-05 |
| pos\_1652 | (7Z,10Z,13Z,16Z)-docosatetraenoate | 3.8222530913392e-05 | 5.76585430775405e-05 | 7.74337357029886e-05 | 0.00012278307452229 | 6.3028252875321e-05 | 6.95625012603061e-05 | 7.64957418204415e-05 | 9.60198725092242e-05 | 4.86228360901885e-05 | 6.12540852279696e-05 | 7.72818129602872e-05 | 7.69507790383796e-05 |
| pos\_1653 | 3,8-Dihydroxy-6-methoxy-7(11)-eremophilen-12,8-olide | 6.06324947033425e-06 | 8.57988856873519e-06 | 1.26511166039424e-06 | 4.53849361562092e-06 | 8.88643537148372e-06 | 5.93706403160147e-06 | 1.60317723886771e-06 | 1.19583604818397e-06 | 2.15225514203341e-06 | 5.84955415754121e-06 | 1.49648939819358e-06 | 1.20357869491511e-06 |
| pos\_1654 | (3E,5Z)-1,3,5-Tridecatriene-7,9,11-triyne | 9.06541925384348e-06 | 1.36964598706159e-05 | 8.06091432434758e-06 | 1.27691508060175e-05 | 1.12247242211114e-05 | 1.15113795518312e-05 | 9.3593937336065e-06 | 9.01917779240651e-06 | 1.17808246486053e-05 | 1.1652984602397e-05 | 9.48864788831384e-06 | 8.62406407764855e-06 |
| pos\_1655 | 3-O-Protocatechuoylceanothic acid | 5.18095489239987e-06 | 1.99381059651376e-05 | 8.65047908198874e-08 | 1.32110313482201e-07 | 3.09477453660808e-06 | 7.60859794427739e-06 | 2.14106870653884e-06 | 5.55643947747025e-06 | 9.32724645184409e-07 | 5.64896428912855e-06 | 9.28569429033314e-06 | 1.9030771646134e-06 |
| pos\_1656 | 3-O-Sulfogalactosylceramide (d18:1/24:1(15Z)) | 2.36439943498236e-06 | 3.26678607256323e-06 | 1.09331147437155e-05 | 1.88454742719494e-05 | 1.19126488056312e-05 | 1.39426664300017e-05 | 2.71662582380538e-05 | 1.45823699649534e-05 | 5.01549469323858e-06 | 4.46071574390799e-06 | 1.34455021925829e-05 | 1.66073311497819e-05 |
| pos\_1657 | 3-O-Sulfogalactosylceramide (d18:1/20:0) | 0.000401397362036678 | 0.000254520483641941 | 0.000236081385440137 | 0.000210471899698022 | 0.000253511225179201 | 0.000279996516761332 | 0.000276523317943342 | 0.0002580631357844 | 0.000263579714874825 | 0.000236918093424841 | 0.000256918566116986 | 0.000246662623981176 |
| pos\_1658 | Serratol | 4.55942843215629e-05 | 2.63921903545958e-05 | 2.2050409910681e-05 | 2.27953527641292e-05 | 1.49432289940826e-05 | 1.67321665351241e-05 | 1.66811205257417e-05 | 2.09247199462063e-05 | 2.11925019243239e-05 | 2.08456456676908e-05 | 1.95824234500931e-05 | 1.78222028363768e-05 |
| pos\_1659 | 2-Propenyl cyclohexanehexanoate | 6.6820657276052e-06 | 7.34377256156783e-06 | 1.14067538931306e-05 | 1.03331136015566e-05 | 5.1197913333794e-06 | 6.20221546194353e-06 | 5.45252281328195e-06 | 6.17834877146806e-06 | 1.87273852356053e-05 | 7.74246170525618e-06 | 9.32337719329765e-06 | 6.06283283289072e-06 |
| pos\_166 | Maslinic Acid | 0.00076307701483257 | 0.000606030855513788 | 0.000416390005206997 | 0.000520553562459443 | 0.000328941823073782 | 0.000537534741752601 | 0.00063635478459471 | 0.000671547527114256 | 0.000367817996141012 | 0.000714395733712287 | 0.000608934902311823 | 0.000744963611385631 |
| pos\_1660 | Talaromycin A | 0.000105286828510063 | 9.96814092220441e-05 | 0.000147530937334115 | 0.000138883908889475 | 0.000155579116157586 | 0.000138370049804309 | 0.000126742240851358 | 0.000126276224750595 | 0.000149533432019621 | 0.000133685812854779 | 0.00013598707261168 | 0.000135547772012717 |
| pos\_1661 | PGP(16:1(9Z)/18:2(9Z,12Z)) | 2.93281299241309e-05 | 1.09743705561238e-05 | 1.06105331922654e-05 | 9.55467889055617e-06 | 1.72354402308556e-05 | 1.73349859393937e-05 | 1.46261285697008e-05 | 1.38891337057238e-05 | 1.53588434529149e-05 | 1.40357306840507e-05 | 1.29185647853683e-05 | 1.37897644623439e-05 |
| pos\_1662 | Linoleoyl ethanolamide | 1.04558333504073e-05 | 1.1984695330253e-05 | 1.09375573644376e-05 | 1.24195061970836e-05 | 1.01236618399633e-05 | 1.10536026872802e-05 | 1.07733449820655e-05 | 8.79488281743722e-06 | 7.87724213085164e-06 | 1.02609217450509e-05 | 9.17628165015446e-06 | 7.73941146716757e-06 |
| pos\_1663 | 3-(10-Heptadecenyl)phenol | 8.91714440756696e-05 | 0.000135254985997463 | 0.000137440983802143 | 0.000108267423178583 | 0.00010871552639758 | 0.000111147003512164 | 9.35188970788987e-05 | 0.000155322839693277 | 0.000135196560297011 | 0.000103535482011753 | 0.000139323951338686 | 0.000260791220035099 |
| pos\_1664 | PC(15:0/15:0) | 0.000178610305347425 | 0.000229343342415696 | 0.00074949462253572 | 0.000139912419704229 | 0.000281277035256201 | 0.000324300897435274 | 0.000394046732472684 | 0.000447281254579183 | 0.000183684590320682 | 0.000574161195527899 | 0.000706408094896231 | 0.000584689563430437 |
| pos\_1666 | Cohibin A | 4.1687988350618e-06 | 1.05105567210309e-05 | 8.47709174694284e-06 | 1.19597276066396e-05 | 4.74523764292399e-06 | 8.95389388389224e-06 | 2.20692649365523e-05 | 6.6424215276938e-06 | 1.40918728939529e-05 | 1.26929645698226e-05 | 1.14401285172593e-05 | 4.71664554004009e-06 |
| pos\_1667 | (3alphaOH,20S,24S)-3,19:20,24-Diepoxydammarane-3,25-diol | 6.38718472195445e-05 | 0.00010097940963295 | 6.9172858740221e-05 | 7.86197518642519e-05 | 5.10903685191579e-05 | 7.98088908741634e-05 | 6.71735134628036e-05 | 6.86728575872906e-05 | 9.49016911727572e-05 | 7.34704786407031e-05 | 0.00011109292306772 | 9.18798259333791e-05 |
| pos\_1668 | LysoPE(0:0/20:0) | 0.000258673887873408 | 0.000257763867564519 | 0.000258717956283284 | 0.000249819483903825 | 0.0002451147725585 | 0.00027173818294925 | 0.0001946568253492 | 0.000235988033214815 | 0.000284115975797601 | 0.000217513370310954 | 0.000246629357341745 | 0.000280649098022003 |
| pos\_1669 | Epimetendiol | 4.31545741645924e-05 | 5.0495137192861e-05 | 4.57364107528493e-05 | 4.94550253269777e-05 | 4.0901727250148e-05 | 4.79685760872012e-05 | 4.18091555913318e-05 | 4.25008422378778e-05 | 5.08048571777306e-05 | 5.12286675812032e-05 | 4.69083069423442e-05 | 4.27234572354362e-05 |
| pos\_1670 | Arvensoside D | 2.00895816150626e-05 | 2.30854086036715e-05 | 3.04226188805094e-05 | 2.32505355303372e-05 | 2.51663916528978e-05 | 2.15299692998726e-05 | 2.66274400238484e-05 | 3.27803271495349e-05 | 2.72583683622384e-05 | 2.12411641810007e-05 | 2.88056887052572e-05 | 3.71119987131265e-05 |
| pos\_1672 | CE(20:4(5Z,8Z,11Z,14Z) | 0.000291185389947436 | 6.33061966217912e-05 | 7.16307028178639e-05 | 5.2854109670935e-05 | 0.000164696527168898 | 0.000157700784083215 | 0.000134494442137282 | 0.000120773532901511 | 8.78929733670662e-05 | 9.73983809162574e-05 | 0.000113697520208945 | 9.55453264033622e-05 |
| pos\_1674 | (3S)-3,6-Diaminohexanoate | 3.11638858498119e-06 | 4.75339500616657e-06 | 1.53766140368263e-06 | 6.87064121847629e-06 | 4.26530710484217e-07 | 3.21610352205684e-06 | 2.67526520302476e-06 | 1.48111586792127e-06 | 3.38703332466646e-07 | 9.58351085696189e-07 | 1.05807828428554e-06 | 3.91072437034555e-07 |
| pos\_1675 | 3-Sulfinato-L-alaninate | 2.37744363783643e-06 | 3.25179782042851e-06 | 2.47359880321855e-06 | 2.67678013017315e-06 | 2.22738708530807e-06 | 2.22936024867083e-06 | 2.12369467613576e-06 | 1.58892366632577e-06 | 3.09479034665259e-06 | 1.49324905903285e-06 | 2.2280171737769e-06 | 1.65074179546724e-06 |
| pos\_1676 | Ginsenoyne N | 0.00455336917297641 | 0.00423234147582069 | 0.00378564900452259 | 0.0043546032465507 | 0.003703951013433 | 0.00403273216633599 | 0.00484202130750251 | 0.00439865903996059 | 0.00540457427028605 | 0.00484703067662243 | 0.00485232555865995 | 0.00442753570163052 |
| pos\_1677 | Isolinderenolide | 0.000601507552745697 | 0.000502270632683005 | 0.000311707435230691 | 0.000390632577413616 | 0.000422819375709939 | 0.000440870720926479 | 0.000342761895923309 | 0.000387205561279824 | 0.000442379615862584 | 0.000480291983192327 | 0.000393481335292569 | 0.000423768395121964 |
| pos\_1678 | Retinyl beta-glucuronide | 0.00259505628705893 | 0.00200869499196989 | 0.00272277037266165 | 0.00248252757186305 | 0.00239008605435897 | 0.00228281277821788 | 0.0020407739433884 | 0.0019687490174695 | 0.00377165027352014 | 0.00250864961058845 | 0.00240350856971212 | 0.0028893461443729 |
| pos\_1679 | Oxandrolone | 0.000154701973038114 | 0.000179144084840424 | 0.000162498498848484 | 0.000173998486513487 | 0.000148415600484578 | 0.000175657608569503 | 0.000148305006257035 | 0.000160329562274518 | 0.000169891187214366 | 0.000195941432172398 | 0.000172629777011495 | 0.000156150243010567 |
| pos\_168 | 1,2,3-Trimethylbenzene | 0.000190550640218208 | 0.000231419459599165 | 0.00020434724361837 | 0.000207725557289141 | 0.000175384271417748 | 0.000194272306153621 | 0.000192038250146887 | 0.000209294504265113 | 0.000205525193680461 | 0.000207701953229701 | 0.000202528157514259 | 0.000193445442489469 |
| pos\_1680 | Agavoside B | 4.4591580881838e-05 | 1.8079348089892e-05 | 1.51709777008543e-05 | 1.23774071286486e-05 | 2.60853649265531e-05 | 2.70862836250048e-05 | 2.93140251514539e-05 | 2.21963362237789e-05 | 1.95299066203794e-05 | 2.03275646305358e-05 | 2.0762318912379e-05 | 1.96709230697185e-05 |
| pos\_1682 | N2-(3-Carboxy-2-hydroxy-1-oxopropyl)arginine | 2.22671967949894e-05 | 1.07995588050793e-05 | 9.96085994029507e-06 | 9.40445888650934e-06 | 1.3103687928958e-05 | 1.33327060926436e-05 | 1.21744174481169e-05 | 9.70673931416209e-06 | 1.32180594271931e-05 | 1.09994696699278e-05 | 1.16833902759275e-05 | 1.16283085334943e-05 |
| pos\_1683 | (E,Z)-2,4-Dodecadiene | 1.39101369344688e-07 | 6.42358860183553e-07 | 3.32000375378446e-07 | 6.76143441198811e-07 | 5.7384249154764e-07 | 6.12619907773862e-07 | 3.03772001321601e-07 | 6.38134804412926e-07 | 5.27099013389315e-07 | 7.27011343137818e-07 | 5.36983903840289e-07 | 3.29642776497084e-07 |
| pos\_1684 | PC(14:1(9Z)/22:1(13Z)) | 0.000187942254127175 | 9.57895912648183e-05 | 9.54573529234897e-05 | 8.37881970874973e-05 | 0.000103004881969453 | 0.000108643877830416 | 0.000111394863164749 | 0.000103129198244543 | 9.95144019664621e-05 | 9.73832374758075e-05 | 0.000105154847311524 | 0.000101604695491652 |
| pos\_1686 | ent-16-Kauren-19-ol | 5.32854898021934e-06 | 5.07211396991826e-06 | 5.68680025615159e-06 | 4.76678870174779e-06 | 4.89957447716534e-06 | 4.99698372056481e-06 | 3.71874014091772e-06 | 4.79386122688191e-06 | 3.9177818644376e-06 | 5.7759079185885e-06 | 4.66911488136348e-06 | 3.41161419362915e-06 |
| pos\_1687 | Safflomin A | 0.000437731545525791 | 0.000114628770298697 | 0.000123793442606082 | 0.000102612008076234 | 0.00025228018743299 | 0.000238796969327646 | 0.000223339341124301 | 0.000192577602570266 | 0.00015085698691146 | 0.000169345726503481 | 0.000183365425870375 | 0.000166726001423726 |
| pos\_1688 | 4-Hydroxy-5-(dihydroxyphenyl)-valeric acid-O-sulphate | 2.43891346815227e-06 | 3.8044157857758e-06 | 3.43702715315719e-06 | 3.12283596439653e-06 | 3.21470006688795e-06 | 3.29623300898938e-06 | 2.31074834124647e-06 | 3.02918566700402e-06 | 3.58198376207135e-06 | 3.65484557545162e-06 | 3.0639560468967e-06 | 4.12519911377543e-06 |
| pos\_1689 | Dihydrocarvone | 0.000113328607360694 | 7.22194685705657e-05 | 3.60631308419172e-05 | 1.5824099079707e-05 | 3.97395830252954e-05 | 4.54513255765654e-05 | 1.18126750348717e-05 | 1.81660439951603e-05 | 1.32601589896243e-05 | 7.63255723457371e-05 | 2.84470157921794e-05 | 2.37498069802743e-05 |
| pos\_169 | 1,5-Octadien-3-one | 0.000119906615485683 | 0.000116451307149837 | 8.84149593907544e-06 | 7.12925741751359e-05 | 0.000119241974338063 | 8.96296971051631e-05 | 5.9508560456319e-06 | 8.1738278484605e-06 | 5.50319443792603e-06 | 8.62031203040669e-05 | 2.60203497421662e-05 | 1.18676738753736e-05 |
| pos\_1691 | Auberganol | 6.1258975942542e-05 | 7.38651770359333e-05 | 0.000104377734811213 | 8.86089405180926e-05 | 8.71143687048161e-05 | 7.93717033113129e-05 | 7.45984130536873e-05 | 9.30430829314456e-05 | 9.02108673189502e-05 | 8.51473906998125e-05 | 8.88138837160781e-05 | 0.000115518771348553 |
| pos\_1692 | Muridienin 4 | 0.000182984915513868 | 0.000326607329891002 | 0.000200829066037412 | 0.000201391512007549 | 0.000120365080148818 | 0.000186590120578839 | 0.000181429132854848 | 0.000207187604677146 | 0.000159926942036401 | 0.000184137930710417 | 0.000198360433591504 | 0.000194256009945069 |
| pos\_1693 | Neuromedin C 1-8 | 6.31972055989501e-05 | 0.000106161406159333 | 3.96918083748305e-05 | 6.88790815217544e-05 | 8.73076514033346e-05 | 7.06532950683414e-05 | 8.89629997357187e-05 | 5.58802965164961e-05 | 8.96136341834682e-05 | 6.3094606849026e-05 | 7.15463035481279e-05 | 4.13594405134489e-05 |
| pos\_1694 | Alkaloid AQC2 | 1.89349760503542e-05 | 2.16578386978095e-05 | 2.17031219490459e-05 | 2.2326630218398e-05 | 2.62741575750777e-05 | 2.64788092924277e-05 | 1.78123310548742e-05 | 2.28772607405822e-05 | 2.15660713620689e-05 | 2.90596230220805e-05 | 2.10902941853741e-05 | 1.99177824000697e-05 |
| pos\_1695 | Ganglioside GM3 (d18:1/9Z-18:1) | 1.97165222729978e-05 | 2.69008309569543e-05 | 7.49144555295668e-06 | 1.25038534481419e-05 | 1.88684999885434e-05 | 1.33094382484854e-05 | 1.70514421616551e-05 | 1.7306345263887e-05 | 1.03396743251483e-05 | 1.41732854488828e-05 | 1.43930926130949e-05 | 1.21023252893121e-05 |
| pos\_1696 | Alanyl-Glutamine | 5.95306119265903e-07 | 2.1837075428064e-07 | 2.05051050762454e-06 | 1.25693388165942e-08 | 1.4314947566839e-06 | 5.8528271930074e-07 | 8.37985276855478e-09 | 1.35545632577128e-07 | 7.09684337742579e-09 | 3.07128675833908e-06 | 8.28763233833851e-07 | 1.30750998154206e-06 |
| pos\_1697 | Moxifloxacin | 0.000239907155797156 | 0.000256389762174175 | 0.000247462343200507 | 0.000216552557877786 | 0.000222231431596781 | 0.000251099311097747 | 0.000173946784047897 | 0.000230256179991444 | 0.000285793036643446 | 0.000199716749806718 | 0.000234677036624321 | 0.000278034148713012 |
| pos\_1699 | Vaporole | 4.82372108923713e-06 | 5.83657858443786e-06 | 5.188186654822e-06 | 5.67058722298253e-06 | 3.85507325871418e-06 | 5.17780222293154e-06 | 4.97105775785184e-06 | 4.94200981260805e-06 | 3.99839978333265e-06 | 4.98981822245919e-06 | 4.85600069373712e-06 | 5.46165321853888e-06 |
| pos\_170 | 3-tert-Butyl-5-methylcatechol | 0.000104892787317473 | 0.000116527884416833 | 9.72280796003271e-05 | 0.000114456048917621 | 0.000107623206550963 | 0.000110597291682777 | 9.13927356322552e-05 | 0.00010355539564864 | 0.000112730558342516 | 0.000100494240704979 | 0.000111836777785499 | 0.000126864460914013 |
| pos\_1700 | Isofucosterol 3-O-[6-O-(9,12-Octadecadienoyl)-b-D-glucopyranoside] | 8.55052736769193e-06 | 1.30675658830625e-06 | 1.0781186028824e-05 | 1.29684648062224e-06 | 1.80500997873465e-06 | 5.17037831069622e-06 | 1.38214374655663e-06 | 4.75445879113451e-06 | 5.71614490860371e-06 | 2.31907347823757e-06 | 2.9800153400283e-06 | 1.11358534014882e-06 |
| pos\_1702 | Evobioside | 0.000218401036935887 | 0.000215131886060313 | 0.000547426367213283 | 0.000423004480919701 | 0.000152699139260766 | 0.000297419130424203 | 0.000201797496683435 | 0.000178186455521349 | 0.000476858555883738 | 0.00047295953284918 | 0.000300430135384774 | 0.000211294280544556 |
| pos\_1703 | (6beta,7alpha,12beta,13beta)-7-Hydroxy-11,16-dioxo-8,14-apianadien-22,6-olide | 0.000528610235528307 | 0.000628004292676776 | 0.000405284541434092 | 0.00050345981880702 | 0.000459415776139955 | 0.000502100002725138 | 0.000515565207520802 | 0.00044472571926599 | 0.000463605794168513 | 0.000587309474454455 | 0.000472103222714873 | 0.000429536886496704 |
| pos\_1705 | 11,14,17-Eicosatrienoic acid | 0.000109090506091833 | 0.000120061956872326 | 0.000103980389631637 | 0.000106856693638802 | 9.8285138009341e-05 | 9.67741039855449e-05 | 0.000116940920258706 | 0.000110746208066976 | 0.000115769523073113 | 0.000111626262362691 | 0.000103581576980053 | 0.000126921212177519 |
| pos\_1706 | Capsianoside V | 1.38133233370974e-05 | 6.51815116112446e-06 | 7.1586707135576e-06 | 5.8995446184461e-06 | 7.05194699730317e-06 | 8.51653061816629e-06 | 2.72373844841597e-06 | 1.43391087439973e-05 | 2.34323822011468e-05 | 4.89630080061971e-06 | 1.47742875805196e-05 | 3.38194569808675e-05 |
| pos\_1707 | Withaphysacarpin | 1.75181705053232e-05 | 3.45639880913016e-06 | 3.24345650144179e-06 | 3.68260678371876e-06 | 1.07336023973126e-05 | 6.92260415647678e-06 | 8.74273734922756e-06 | 5.48056011075394e-06 | 4.87065629908478e-06 | 5.94035983644189e-06 | 5.91860579717586e-06 | 6.58262454769287e-06 |
| pos\_1709 | SM(d18:1/18:1(11Z)) | 1.80215762227263e-05 | 1.12998992373128e-05 | 3.24019917249736e-06 | 3.24934219614462e-06 | 7.39459374326103e-06 | 2.43670719000913e-06 | 8.3170423297223e-07 | 2.02252000986187e-06 | 6.74515129807414e-06 | 7.79686902947528e-07 | 3.01600652147112e-06 | 0 |
| pos\_171 | 6-Methyl-3,5-heptadien-2-one | 0.00015727986599281 | 0.000152712851748616 | 9.02602190435668e-06 | 7.36182320650965e-05 | 0.000154594245890527 | 0.000106074078167348 | 1.20708137270274e-05 | 9.95783215804234e-06 | 6.56725330852377e-06 | 8.77134152610845e-05 | 2.62328037146796e-05 | 1.09637230685815e-05 |
| pos\_1711 | DG(14:0/22:1(13Z)/0:0) | 0.000300152717651202 | 0.000465469675404944 | 0.000267053870325292 | 0.000268055353518002 | 0.000237519444123103 | 0.000265985471659474 | 0.000267364582960366 | 0.000277248394028891 | 0.000343434213852087 | 0.000266910605150075 | 0.000291489320228667 | 0.000270030568001878 |
| pos\_1713 | Eicosapentaenoyl Ethanolamide | 8.28308384472386e-06 | 8.21797898361499e-06 | 7.91367859341036e-06 | 9.89075632430076e-06 | 1.78821892721777e-06 | 8.38387125573093e-06 | 6.96972886713317e-06 | 9.81934858903561e-06 | 5.8688189093146e-06 | 4.60443360702987e-06 | 8.01368983508635e-06 | 9.36809835662936e-06 |
| pos\_1714 | Arachidonic acid | 0.000139259416459181 | 0.00014478183124908 | 0.000147805661678119 | 0.00013781714715075 | 0.000130859305980443 | 0.000140715246726468 | 0.000131230049748656 | 0.000147467977614299 | 0.000122272856051475 | 0.000166412727785426 | 0.000137263917647773 | 0.000147358391702204 |
| pos\_1715 | 12-O-D-Glucuronoside-13-hydroxyoctadec-9Z-enoate | 1.73473046639594e-05 | 7.33345067075387e-06 | 3.2888562741343e-06 | 1.19170715825092e-05 | 4.09904386036592e-06 | 7.54537595893937e-06 | 1.47500355124676e-05 | 6.92949419670757e-06 | 1.64567148033015e-05 | 3.08477631543283e-06 | 3.91576430701058e-06 | 4.2651089497517e-06 |
| pos\_1716 | PE(18:1(11Z)/22:5(4Z,7Z,10Z,13Z,16Z)) | 3.80248569577814e-05 | 3.5827792338234e-05 | 1.896351725351e-05 | 3.00234487085515e-05 | 2.51758806320402e-05 | 4.0931276313577e-05 | 2.31967294255918e-05 | 3.11968740110539e-05 | 2.32444505552389e-05 | 2.81144095345592e-05 | 3.20947290309215e-05 | 3.42440811760526e-05 |
| pos\_1717 | 2-Isopropylmalic acid | 1.66838670949737e-06 | 8.79966780141515e-07 | 2.24200832169205e-07 | 2.12106263873587e-07 | 1.37427532440006e-05 | 4.14360776614945e-06 | 8.98484851468932e-06 | 6.44061340869411e-06 | 2.01936639575558e-07 | 1.98573839728502e-06 | 6.26792862606943e-06 | 8.94955058241274e-06 |
| pos\_1718 | Cascarillin | 0.000204151957317787 | 0.00029308837063977 | 0.000152719961908959 | 0.000215160876792639 | 0.000213914055781569 | 0.00021259985310358 | 0.000196869296751499 | 0.000178417245076463 | 0.000199845758243052 | 0.000222494261459411 | 0.000187205568352583 | 0.00016747023700492 |
| pos\_1719 | N1,N5,N10-Tris-trans-p-coumaroylspermine | 1.02065955258591e-05 | 1.54922334069978e-05 | 4.05903592204418e-06 | 6.1451089625676e-06 | 1.05197468694688e-05 | 9.62362677719145e-06 | 6.28314197221827e-06 | 1.20006473971978e-05 | 7.88862864007418e-06 | 6.97651341861615e-06 | 9.38554148254921e-06 | 1.50436248439672e-05 |
| pos\_172 | N-Methyl-2-pyrrolidinone | 4.80571804963517e-06 | 6.57501182818388e-06 | 4.79334354647363e-06 | 4.0759749584598e-06 | 6.47310384576403e-06 | 5.0600136860702e-06 | 4.68350431135087e-06 | 4.79900945552291e-06 | 6.11354966159747e-06 | 5.13783025678612e-06 | 5.17299079707074e-06 | 5.92733566584872e-06 |
| pos\_1720 | N-arachidonoylglycinate | 2.01990518871537e-06 | 5.27837810689005e-06 | 4.33462836762712e-06 | 5.87724486977586e-06 | 4.84866143431312e-06 | 3.90289099711463e-06 | 1.23017882522485e-05 | 8.21921502245616e-06 | 7.90830401737774e-06 | 9.85687455618273e-06 | 1.02711284390286e-05 | 9.73017249343252e-06 |
| pos\_1721 | 2-Decarboxybetanin | 2.74641391893889e-06 | 3.06171957188357e-06 | 1.16573106096352e-06 | 1.89050128230932e-06 | 3.22542650478325e-06 | 2.09032644274083e-06 | 2.46833646982472e-06 | 2.59587767055232e-06 | 1.38330523748287e-06 | 2.21710088481495e-06 | 1.46798941475643e-06 | 3.42252913905054e-06 |
| pos\_1722 | 7-Hydroxy-3,4',8-trimethoxyflavone | 0.000723560492164848 | 0.00138000142104095 | 0.000575575124328803 | 0.000680178005887673 | 0.000845579251113736 | 0.000773889177834578 | 0.000636987992786188 | 0.000673486242598312 | 0.000837907883930781 | 0.00109979476295091 | 0.000816408612057262 | 0.000778739833701833 |
| pos\_1723 | 4-cholesten-7α,12α,24-triol-3-one | 0.00119387954012124 | 0.00144539670573145 | 0.00130432450223398 | 0.00118667962821314 | 0.00121437942561365 | 0.0013385520391035 | 0.00120801857517681 | 0.00128655714540516 | 0.00139000236392995 | 0.00127272914373688 | 0.00131393942189509 | 0.00129081274376345 |
| pos\_1725 | 2-Hydroxypropyl 2-isopropyl-5-methylcyclohexyl carbonate | 2.09278170651115e-05 | 1.77866347355723e-05 | 2.066835480451e-05 | 2.46945626260658e-05 | 1.54578744515377e-05 | 2.02795948297334e-05 | 1.92263613040122e-05 | 2.26595107887944e-05 | 2.15424660561936e-05 | 1.72400436838785e-05 | 2.07912227634211e-05 | 2.73997442816518e-05 |
| pos\_1727 | Nb-Hexacosanoyltryptamine | 9.04821679503414e-05 | 0.000142778121411443 | 0.000112798729084826 | 0.000127622106124926 | 0.000190946471749622 | 0.000146829402707336 | 0.000152345388507972 | 0.000140437655710937 | 0.000196360366588521 | 0.000142739631677436 | 0.00012862741897866 | 0.000109139972527181 |
| pos\_1728 | Sulfathiazole | 3.1253973927457e-05 | 5.53352825304665e-05 | 1.73110039783025e-05 | 2.8660782497949e-05 | 4.58965045342667e-05 | 3.16762802406695e-05 | 2.95474968157812e-05 | 2.09315669744282e-05 | 2.41301718757584e-05 | 2.67104881219098e-05 | 2.74515183858826e-05 | 1.60666100073082e-05 |
| pos\_1729 | hydroxyitraconazole | 0.000460477975257688 | 5.46566999187526e-05 | 7.78775591480883e-05 | 5.37244571722479e-05 | 0.000223832341738389 | 0.000208239074909163 | 0.000168234704179892 | 0.000141350675403352 | 9.72705314080921e-05 | 0.000121813176400878 | 0.000139212526585238 | 0.000109209432930639 |
| pos\_173 | 5,7,4'-TRIMETHOXYFLAVONE | 8.53458785707154e-06 | 2.49977325457512e-06 | 1.32022284439624e-05 | 2.59903837379596e-05 | 9.91070185106767e-05 | 1.58191376894898e-05 | 3.42252210938672e-05 | 1.85448817273612e-05 | 1.44515947007586e-05 | 5.62217425128206e-06 | 1.9057286505457e-05 | 2.61845901671307e-06 |
| pos\_1730 | Sudachiin A | 0.000122160156394738 | 0.000102783544569331 | 9.36410999714494e-06 | 7.72639396614687e-05 | 4.15910803960001e-05 | 5.93546909192091e-05 | 3.93272584337605e-05 | 1.95271151699511e-05 | 4.27452819437731e-06 | 0.000124615243745449 | 3.13432819544717e-05 | 6.2556614153961e-06 |
| pos\_1731 | 3,4-Hexahydroxydiphenoylarabinose | 1.06738798745847e-05 | 1.15815484150759e-05 | 1.07143239483805e-05 | 9.82657753352065e-06 | 1.02039261541273e-05 | 1.09521855855982e-05 | 9.61770666655533e-06 | 9.92204125044085e-06 | 1.0798769609046e-05 | 9.87678543414568e-06 | 1.08839620731623e-05 | 1.53210974843675e-05 |
| pos\_1732 | 3-Hydroxy-4-butanolide | 8.85106409143989e-05 | 0.000128036017580714 | 6.96146804107134e-05 | 7.94749403291091e-05 | 8.75141557608903e-05 | 8.69732235554546e-05 | 8.58117430169648e-05 | 8.73528381648788e-05 | 8.87386225983425e-05 | 0.000121995038848819 | 9.49381564665335e-05 | 9.33500667641152e-05 |
| pos\_1733 | (Z)-3-(1-Butenyl)-1H-2-benzopyran-1-one | 6.97649604455309e-05 | 0.000118918054334656 | 5.87519406523004e-05 | 8.49032085951869e-05 | 9.28438994394634e-05 | 8.11464359093359e-05 | 8.65408493380565e-05 | 6.29523550534195e-05 | 0.00010480390415649 | 8.11979254758273e-05 | 8.43292675508575e-05 | 5.6631503149427e-05 |
| pos\_1734 | Frovatriptan | 0.000120289679109441 | 0.000129352610952777 | 0.000310030048763818 | 4.39285279552157e-05 | 0.000176681802840507 | 0.000156832984712659 | 1.94345220119232e-05 | 0.000120300952077259 | 4.22464584467693e-05 | 0.000495184406677385 | 0.000146619171073995 | 0.000238236891895102 |
| pos\_1736 | 6-beta-Naltrexol | 1.76025123661979e-05 | 1.96745516466281e-05 | 5.66569396753066e-06 | 9.89521327108511e-07 | 4.35777363985879e-06 | 5.04821930572054e-06 | 5.12802522900038e-06 | 4.95758315793548e-06 | 8.1266861582023e-07 | 3.7392416590155e-06 | 3.31778370109317e-06 | 2.69219919906764e-06 |
| pos\_1737 | PE(20:0/22:5(4Z,7Z,10Z,13Z,16Z)) | 0.000148593398225745 | 0.000198570219984799 | 0.000357885212929226 | 0.000126700998281548 | 0.00017790927854759 | 0.000206813156144413 | 0.000205867906126906 | 0.000223593568860273 | 0.000151475545421543 | 0.000273486771842719 | 0.0003120989552192 | 0.000319114626249863 |
| pos\_1738 | Cryptomeridiol 11-rhamnoside | 0.000173512513109574 | 0.000203409072534852 | 0.000284738366605009 | 0.000293013189593312 | 0.000259895521826315 | 0.000250190939852955 | 0.000240084336739703 | 0.000246857247804157 | 0.000188740840423079 | 0.000233580017066765 | 0.000288842804123026 | 0.000207299292628006 |
| pos\_174 | 3beta-[(Tetrahydro-2H-pyran-2-yl)oxy]androst-5-en-17beta-ol | 0.000164009331365679 | 0.000144877219723187 | 0.000298506519070849 | 0.000261869952878053 | 0.000273669812175889 | 0.000227016698926071 | 0.00020316124815276 | 0.000250192623462344 | 0.000274112947892093 | 0.00022741640568434 | 0.000218409261747057 | 0.000207745621894746 |
| pos\_1740 | Thiamine monophosphate | 0 | 0 | 3.10516390444384e-06 | 0.000136038485298921 | 0.000110208977249046 | 4.76574571455428e-05 | 9.76445807497481e-05 | 4.74597848214134e-05 | 0 | 1.40695424420054e-07 | 2.78540437800712e-05 | 1.30679056632118e-06 |
| pos\_1741 | SM(d18:0/26:1(17Z)) | 6.48178100858287e-06 | 7.87731590058815e-06 | 2.024447483173e-06 | 1.23867316745793e-06 | 1.13833841050177e-06 | 0 | 6.38669320173664e-07 | 2.06274175985181e-06 | 9.92690932523739e-07 | 8.1223137889473e-07 | 0 | 0 |
| pos\_1742 | trans-Zeatin-O-glucoside riboside | 1.86134104995092e-05 | 4.59083070021864e-07 | 1.12156859930729e-06 | 2.58307188013838e-07 | 7.77642650391878e-06 | 4.82202632601587e-06 | 3.17684267138322e-06 | 3.1722653757021e-06 | 1.5235437927964e-06 | 4.1305583828196e-06 | 3.92362274270034e-06 | 1.59580990581582e-06 |
| pos\_1743 | 6''-O-(3-Hydroxy-3-methylglutaroyl)astragalin | 0.000421483983737816 | 0.000117269754734374 | 0.000126901068408755 | 9.97076822744594e-05 | 0.000251252286152617 | 0.00024078451223041 | 0.000225045432326893 | 0.00019870752611748 | 0.000155087160841657 | 0.000172602395694812 | 0.000184427367375585 | 0.000174304974003735 |
| pos\_1744 | N-a-Acetyl-L-arginine | 2.0042367151966e-05 | 1.912729014115e-05 | 1.38024323060824e-05 | 1.42244550029976e-05 | 1.87766807961872e-05 | 1.6695845563934e-05 | 1.69860757245339e-05 | 1.62764597999826e-05 | 1.7000492216962e-05 | 1.44482470976156e-05 | 1.51617883513682e-05 | 1.59769870502382e-05 |
| pos\_1745 | Cysteamine | 2.05546588562757e-05 | 2.80757907429807e-05 | 2.51158392249376e-05 | 2.88665021749051e-05 | 2.03449707637331e-05 | 2.49247296832423e-05 | 2.14322250279891e-05 | 2.33661190282197e-05 | 2.75749458446274e-05 | 2.29097265853325e-05 | 2.47261699058407e-05 | 2.05044600755337e-05 |
| pos\_1746 | Streptomycin | 1.05578862561435e-05 | 6.63500953523452e-06 | 9.44373250376803e-08 | 4.23725525096492e-06 | 2.19922765593095e-06 | 4.79706231050792e-06 | 1.00266391267448e-06 | 2.88869275526269e-07 | 1.00414105924756e-06 | 1.18349003383332e-05 | 1.69599212347563e-06 | 9.74691288409544e-07 |
| pos\_1748 | Murrayenol | 0.000739070936396338 | 0.00116183960014767 | 0.00264380790903809 | 0.00159304370610495 | 0.00079189780045183 | 0.00130210880700326 | 0.00144261569257606 | 0.00202305849884612 | 0.0016326040923172 | 0.00116854563934902 | 0.00187253078554046 | 0.00274294887137333 |
| pos\_175 | estra-5,7,9-triene-3β,17β-diol | 0.000127133414608659 | 0.000158654999248865 | 0.000233831424325996 | 0.000191350392439507 | 0.00023402360994505 | 0.00018472997143095 | 0.000126836298690216 | 0.000131312213104527 | 0.000200241702171412 | 0.000197950748655216 | 0.000154993456927617 | 0.000167317431364636 |
| pos\_1750 | Bifonazole | 1.09978091894438e-05 | 1.55372567130016e-05 | 4.75281137310044e-06 | 5.91540828202743e-05 | 9.50591314915873e-06 | 2.18367643650018e-05 | 1.22732788828335e-05 | 9.44870080200524e-06 | 1.24066883509565e-05 | 1.43293494724698e-05 | 1.43502418701124e-05 | 7.86946714491023e-06 |
| pos\_1751 | Levonorgestrel | 0.000661553157691906 | 0.000630050072978549 | 0.000516548445121884 | 0.000742423425941775 | 0.000563058366748869 | 0.000636529605483986 | 0.000598009439582229 | 0.000600191089141148 | 0.00061852607701281 | 0.000614228629132123 | 0.000623088866558912 | 0.000618937185255966 |
| pos\_1752 | Alpha-Tocotrienol | 0.000113989652637459 | 8.36332072589258e-05 | 0.000228787805257989 | 0.000205949191212577 | 0.000112617429374135 | 0.000154960946275455 | 0.000167680094971369 | 0.000222005564865477 | 0.000151263216142103 | 0.000190146395045011 | 0.000229131317016632 | 0.000345332814689888 |
| pos\_1753 | Isoorientin 2''-[feruloyl-(->6)-glucoside] | 4.56874305413289e-06 | 9.18583571866851e-06 | 2.84327179556007e-05 | 1.53087052737516e-05 | 1.08193359546098e-05 | 2.50493870940448e-05 | 1.53342734409507e-05 | 2.06351207637483e-05 | 1.81909928563235e-05 | 1.90795882782787e-05 | 2.56460038298737e-05 | 1.31867230480053e-05 |
| pos\_1754 | Calendasaponin D | 3.19602605331815e-05 | 3.59250842701231e-05 | 3.43273145969852e-05 | 2.72237289541733e-05 | 3.4475099553468e-05 | 3.62992743224212e-05 | 3.03524710854786e-05 | 4.61372287373914e-05 | 4.0112370967e-05 | 3.27035826152421e-05 | 4.00228058365836e-05 | 4.00317128071781e-05 |
| pos\_1756 | cis-Quinceoxepane | 4.70374672663128e-05 | 6.9594716375232e-05 | 6.00982870780788e-05 | 6.72387386424992e-05 | 5.06933921334488e-05 | 5.24601934783265e-05 | 4.14457362299246e-05 | 5.38558900205773e-05 | 5.90132631709965e-05 | 5.2904288192798e-05 | 6.07183519281036e-05 | 5.4546993435761e-05 |
| pos\_1757 | 3b,18b-3-Methoxy-11-oxo-12-oleanen-30-oic acid | 0.000105241495425336 | 0.000117033464426682 | 0.000123751346033085 | 0.000211908483617171 | 9.8242238940333e-05 | 0.000142694644671408 | 0.000137941254083015 | 0.000141043604190917 | 9.28968389172717e-05 | 0.000106585986820096 | 0.000140750806066574 | 0.000175853202086324 |
| pos\_1759 | Imipenem | 0.000130551985388323 | 2.11722959424531e-05 | 2.31648938132097e-05 | 3.4897716331555e-05 | 6.87680769013443e-05 | 6.96039695665301e-05 | 6.61931306972448e-05 | 3.86986719732703e-05 | 2.70285511924143e-05 | 4.57454149670718e-05 | 4.88201874703329e-05 | 3.87963523228917e-05 |
| pos\_176 | 11-Deoxocucurbitacin I | 0.000119322834999122 | 0.000142934495283886 | 0.000171639029531946 | 0.000144290531198277 | 7.26458031238438e-05 | 0.000128138604692808 | 8.15383609665462e-05 | 0.000274872644615187 | 0.000110640941489071 | 0.000104680758878439 | 0.000219111309992251 | 0.000600404185020769 |
| pos\_1760 | 3,4-Dicaffeoyl-1,5-quinolactone | 0.000120135182165868 | 3.1269855415353e-05 | 3.26961512607324e-05 | 2.38873785772441e-05 | 6.44334322020317e-05 | 6.09186349871659e-05 | 5.63109472083788e-05 | 5.06099153446408e-05 | 3.87095732925807e-05 | 4.14673888753594e-05 | 4.6352226559145e-05 | 4.67481916997024e-05 |
| pos\_1762 | Kaempferol 3-rhamnoside 7-xyloside | 7.95496945695739e-06 | 7.45851955496537e-06 | 4.97067360893642e-06 | 4.29911115646937e-06 | 1.45439763251111e-05 | 1.05338756974463e-05 | 7.56400729866278e-06 | 8.92456278959625e-06 | 8.09487551359164e-06 | 4.97150838892401e-06 | 5.56447009619415e-06 | 2.34590552457351e-05 |
| pos\_1763 | Maysin | 1.02259767177147e-05 | 7.81712782653714e-06 | 6.08535612786916e-06 | 6.65926193445824e-06 | 8.83284695703955e-06 | 1.01557386802847e-05 | 8.39573075231249e-06 | 1.15292204090302e-05 | 9.08409574175966e-06 | 8.40621636466778e-06 | 7.99333995857176e-06 | 1.08013343938058e-05 |
| pos\_1764 | (Z)-1,3-Octadiene | 2.34567052014201e-05 | 3.29584618938512e-05 | 3.66196781243977e-05 | 3.55868596152083e-05 | 3.67771965030342e-05 | 3.17086266847333e-05 | 3.5414501740554e-05 | 3.93652513046372e-05 | 3.41199369491159e-05 | 3.21597900467577e-05 | 3.39511193243244e-05 | 3.79384647407912e-05 |
| pos\_1765 | Avocadyne 2-acetate | 0.000652544139941165 | 0.000701288732228615 | 0.00107062710001758 | 0.00130053866685194 | 0.0015239221223317 | 0.000960376051699276 | 0.00126205308689085 | 0.00128277417330579 | 0.00103589275897658 | 0.000825980884544365 | 0.000944342017465196 | 0.000807306491957894 |
| pos\_1766 | Camelliagenin A | 0.00091563116451158 | 0.00139307750880937 | 0.00160371587296767 | 0.0016721149434674 | 0.00395615752349757 | 0.0020199557564007 | 0.00137744831170148 | 0.00135195741958825 | 0.005077935702051 | 0.00119257939560304 | 0.00244435827368675 | 0.00177157867469447 |
| pos\_1767 | Diphenhydramine N-glucuronide | 0.00017822576748854 | 9.22929767535195e-06 | 2.16895833397924e-05 | 1.09838372633736e-05 | 9.20776557211954e-05 | 8.83470418727337e-05 | 7.35067243354207e-05 | 5.30791607347756e-05 | 2.57936916498723e-05 | 5.56448550327602e-05 | 5.68290887864712e-05 | 4.49896419985009e-05 |
| pos\_177 | Sorbaldehyde | 9.29527433516892e-05 | 9.40567269680116e-05 | 7.84459323925632e-06 | 5.58567045964917e-05 | 9.63911449398946e-05 | 7.27452671784096e-05 | 5.84347274634726e-06 | 8.20323888911842e-06 | 6.11422266290188e-06 | 6.93662648711207e-05 | 2.28107930156945e-05 | 1.10735785570469e-05 |
| pos\_1770 | Tetracosapentaenoic acid (24:5n-6) | 1.22213565541176e-07 | 2.23715777754506e-06 | 1.40623667838907e-06 | 1.07084689954604e-05 | 4.82191981642704e-06 | 3.29404028239673e-06 | 2.68554004847188e-06 | 3.79114748749307e-06 | 6.09589405359998e-06 | 3.83523135768428e-06 | 4.84928112242332e-06 | 4.45156334984099e-06 |
| pos\_1773 | Perindopril | 2.15864136315186e-05 | 2.36183277511375e-05 | 2.57258809102752e-05 | 2.15362683656342e-05 | 1.85071623132884e-05 | 2.13869300909875e-05 | 1.53355544080098e-05 | 1.9543131176057e-05 | 2.34004391618313e-05 | 2.11572659203152e-05 | 2.13369338575117e-05 | 2.15424890952992e-05 |
| pos\_1774 | Furfuryl propyl disulfide | 3.69357506256958e-06 | 6.33661351734882e-06 | 5.88386263258845e-06 | 4.1171188469027e-06 | 3.55907202572199e-06 | 4.97719640621853e-06 | 3.69161138043991e-06 | 3.83134956079861e-06 | 4.84300915955722e-06 | 4.35258038064259e-06 | 4.79979677862768e-06 | 3.99688419343959e-06 |
| pos\_1777 | Xerocomic acid | 9.0932478072819e-05 | 7.37021277532856e-06 | 1.28989599626927e-05 | 1.03931652792132e-05 | 5.05787587224109e-05 | 5.05751465918503e-05 | 3.81001427921116e-05 | 3.66149570509109e-05 | 1.4690038309292e-05 | 1.85565994205018e-05 | 2.52406759868839e-05 | 2.42092461701424e-05 |
| pos\_1778 | 1,4,6-Trihydroxy-5-methoxy-7-prenylxanthone | 7.7565567341005e-07 | 1.11271980472457e-06 | 2.43511742405756e-06 | 1.78361646407776e-05 | 1.67664592645928e-06 | 4.85681595418129e-06 | 4.93657809839184e-06 | 6.68663503766007e-06 | 7.7329392330319e-07 | 1.81711386510647e-06 | 3.9707835475289e-06 | 1.28922712222103e-05 |
| pos\_1779 | 1-(Methylthio)propyl propyl disulfide | 3.45624171584128e-05 | 4.93903849502792e-05 | 3.35843019762062e-05 | 3.26759247436176e-05 | 2.79106884762443e-05 | 2.77104446519265e-05 | 3.15365409214896e-05 | 3.28974215514883e-05 | 3.5424033523577e-05 | 2.90601550847732e-05 | 3.3335976533988e-05 | 3.2761525272162e-05 |
| pos\_178 | Gibberellin A15 | 0.000335286039018982 | 0.000385460469870052 | 0.000743329172578542 | 0.000104297563290553 | 0.000427657609532506 | 0.000400157379128532 | 4.31511252653077e-05 | 0.000277758621299484 | 9.8086556152395e-05 | 0.000862693748722429 | 0.000392855441378666 | 0.000623827029424629 |
| pos\_1781 | 1-Caffeoyl-5-feruloylquinic acid | 2.9021360726673e-05 | 3.29876639221123e-05 | 7.5777366337567e-07 | 2.15489884073793e-05 | 2.86815025615071e-05 | 2.10782468048016e-05 | 5.82606866402045e-06 | 2.63788816008932e-06 | 3.46742107997454e-06 | 2.43405496352548e-05 | 8.17103702515063e-06 | 2.67110300241194e-06 |
| pos\_1782 | PS(DiMe(11,5)/MonoMe(11,5)) | 2.44273669189824e-05 | 8.26636080585226e-06 | 6.24928729423117e-06 | 8.03818541394759e-06 | 1.46919023410909e-05 | 1.43686527299744e-05 | 1.56013634668346e-05 | 1.05083324624279e-05 | 1.01758758165614e-05 | 1.06784708043204e-05 | 1.20451541881197e-05 | 9.69331353673275e-06 |
| pos\_1783 | Farnesyl pyrophosphate | 0.000279038533354788 | 4.85598159836186e-05 | 3.83641578176847e-05 | 3.11866272568979e-05 | 0.000128811357768203 | 0.000118444148482162 | 0.000105552879951898 | 8.17071782631454e-05 | 5.92095244190923e-05 | 6.44547135769943e-05 | 6.91592821456209e-05 | 6.30209955801619e-05 |
| pos\_1787 | Calenduloside E | 0.00100526683187332 | 0.000614086804970598 | 0.000530234195210695 | 0.000525245654618866 | 0.000553186864046073 | 0.000640476078125512 | 0.000609372885423717 | 0.000597960540329256 | 0.000610007789450364 | 0.000542606022091618 | 0.000582330593401447 | 0.000600874396306345 |
| pos\_1788 | lysoPC(26:0) | 0.00276752128088893 | 0.00079194193092646 | 0.000789141225298231 | 0.000735544519799722 | 0.00121735626887987 | 0.00116575246078691 | 0.00140404577113039 | 0.0010371732859331 | 0.000940978896031397 | 0.000940983745232361 | 0.00102835197846709 | 0.00104308579782491 |
| pos\_1789 | Ganoderic acid Mi | 0.00173670135870755 | 0.000546292776914039 | 0.000488564306150751 | 0.000490462242661713 | 0.000769601965354044 | 0.000758376929346838 | 0.000946319078359371 | 0.000703707371337925 | 0.000638595151243396 | 0.000685746160597184 | 0.000726256196144979 | 0.000714235387738719 |
| pos\_179 | Cyclokievitone | 1.46855057528133e-05 | 0 | 2.16488550951972e-05 | 1.99033731753148e-05 | 5.09720963919988e-06 | 1.31947987202626e-05 | 7.0312011372502e-06 | 8.31964487240326e-06 | 2.38723064616179e-06 | 7.49853684292155e-06 | 6.69786683141053e-06 | 1.26755452778363e-05 |
| pos\_1790 | DG(20:3(5Z,8Z,11Z)/22:2(13Z,16Z)/0:0) | 1.16236010142847e-05 | 4.89618952298011e-06 | 2.60841888241088e-06 | 2.49495247989789e-06 | 0 | 1.23891014815486e-06 | 2.7070604712863e-06 | 9.06237348942013e-07 | 9.58325823605224e-07 | 1.20090390511309e-06 | 3.02329591633743e-06 | 1.31577411844522e-06 |
| pos\_1791 | 6-Sinapoylglucoraphenin | 0.000122434348692279 | 1.99928171829801e-06 | 1.01497427155545e-05 | 4.70201675256348e-06 | 5.27613460114432e-05 | 3.74033102108925e-05 | 2.75416216063363e-05 | 2.16373586567615e-05 | 9.48272546839042e-06 | 9.94522035992775e-06 | 1.81971444047242e-05 | 1.21799116608959e-05 |
| pos\_1792 | 2-Hydroxypentyl glucosinolate | 1.81567274537633e-06 | 2.46844927263787e-06 | 3.69342081991291e-06 | 2.81234109167332e-06 | 2.28739998560167e-06 | 2.1492514289354e-06 | 1.25346876885529e-06 | 2.17377511684007e-06 | 3.20214857970551e-06 | 1.45968110268748e-06 | 2.27234711719208e-06 | 2.05243808246008e-06 |
| pos\_1793 | Acetaminophen cystein | 2.27775417458567e-05 | 1.26956796700991e-05 | 1.16629284254545e-05 | 1.2048956312164e-05 | 1.86132207791146e-05 | 1.89937338336005e-05 | 1.86918617781086e-05 | 1.8703175661326e-05 | 1.601087965719e-05 | 1.68294611248557e-05 | 1.47368169611882e-05 | 1.86911607220098e-05 |
| pos\_1794 | 5-O-a-L-Arabinofuranosyl-L-arabinose | 0.000501287318757429 | 0.000900538154465287 | 0.000359428672995427 | 0.000437299164873043 | 0.00055529719494412 | 0.000517474639481919 | 0.000421398192939336 | 0.000426830983992844 | 0.000556658408579359 | 0.000673585033163359 | 0.000515841223995247 | 0.000476055141424907 |
| pos\_1795 | PI(16:0/16:1(9Z)) | 0.000418816485934457 | 0.000227843116370226 | 0.000202863056074225 | 0.000207981192812023 | 0.000229724197844223 | 0.000248416480630155 | 0.000247448646251824 | 0.000231205655864724 | 0.000227048133760822 | 0.000217691502014069 | 0.00022532144693185 | 0.000229773566736774 |
| pos\_1796 | Ganglioside GA2 (d18:1/24:1(15Z)) | 0.00226004631086865 | 0.000988975842833614 | 0.00155640539628691 | 0.0014578220095188 | 0.00206272619273655 | 0.00188678680629111 | 0.00193269732428273 | 0.00161043605617688 | 0.00180586055358262 | 0.00161813789600939 | 0.00174263572632141 | 0.00171647482642371 |
| pos\_1797 | (R)-2-Amino-N-(2,2,4,4-tetramethyl-3-thietanyl)propanamide | 4.79099202650126e-05 | 4.07098365451548e-05 | 2.57609693052612e-05 | 3.47611516921107e-05 | 4.21563152961804e-05 | 4.07252808984417e-05 | 4.38598177148665e-05 | 3.47917751362522e-05 | 3.99146223323496e-05 | 4.01945676709413e-05 | 3.49951792975117e-05 | 4.86103884225934e-05 |
| pos\_1798 | Malformin | 6.44030748863139e-06 | 9.0182961545723e-06 | 1.01352202366171e-05 | 9.53337405349974e-06 | 6.8851790412444e-06 | 7.3981329413254e-06 | 6.47934049515573e-06 | 7.40769495709745e-06 | 8.41225100887304e-06 | 6.88073914220843e-06 | 8.1590851641134e-06 | 7.8472066077226e-06 |
| pos\_1799 | Arjunolic acid | 1.02623627347179e-05 | 1.39533912637114e-05 | 1.52789566634557e-05 | 1.21660489791269e-05 | 9.15061692391124e-06 | 1.20997001342599e-05 | 9.50003609602716e-06 | 1.05041220189331e-05 | 1.51376179095604e-05 | 1.04813763579049e-05 | 1.23675757693311e-05 | 1.18931584500344e-05 |
| pos\_18 | L-Homocystine | 2.5059674096117e-05 | 2.05771464551447e-05 | 1.80556187279243e-05 | 1.91580165072646e-05 | 2.03206416157462e-05 | 2.15534316956841e-05 | 2.14695059927134e-05 | 1.9903341274391e-05 | 2.45399453072706e-05 | 1.62800226911684e-05 | 1.96545347958101e-05 | 7.85363457708559e-05 |
| pos\_180 | 2,4,7-tridecatrienal | 0.000261051009976637 | 0.00031433141842704 | 0.000279295327645306 | 0.000312505839364568 | 0.000261179775814943 | 0.000288235682776544 | 0.000256180956054207 | 0.000283062440105652 | 0.000338701576292874 | 0.000291223465759997 | 0.000301560696604535 | 0.000281326843284253 |
| pos\_1800 | LysoSM(d18:0) | 9.16700377712325e-06 | 4.98131910286795e-05 | 9.06538699226857e-06 | 7.4361812844962e-06 | 8.63058449462806e-06 | 1.63035336499094e-05 | 8.06769388220034e-07 | 1.07901083051023e-06 | 5.10595017161661e-06 | 9.2823968137827e-06 | 1.64649293625618e-06 | 4.96204566475225e-07 |
| pos\_1801 | Pokeberrygenin | 1.24617112695528e-05 | 1.44298784094535e-05 | 1.33738038861187e-05 | 1.11627227549141e-05 | 1.22382946755805e-05 | 1.21689827657182e-05 | 1.26873850463375e-05 | 1.33764992444398e-05 | 1.49696321101888e-05 | 1.12042122794441e-05 | 1.31336686168575e-05 | 1.03556832573645e-05 |
| pos\_1802 | 1,2-Dehydro-alpha-cyperone | 6.94972447461493e-05 | 7.40682498338968e-05 | 6.15983431038904e-05 | 8.23755492019107e-05 | 5.87583835812377e-05 | 7.39054590596905e-05 | 6.84223773398651e-05 | 7.22541206864015e-05 | 6.64314314972537e-05 | 6.73076799593883e-05 | 7.38807187909402e-05 | 6.7697432078476e-05 |
| pos\_1803 | N,2,3-Trimethyl-2-(1-methylethyl)butanamide | 7.3423072928433e-05 | 7.55052064226236e-05 | 7.12373025757671e-05 | 7.16127022804521e-05 | 6.40831991854902e-05 | 6.78436261148825e-05 | 4.20768710541711e-05 | 5.68277208343466e-05 | 8.81443552623008e-05 | 7.76926881431378e-05 | 6.79957019786151e-05 | 7.05107427117208e-05 |
| pos\_1805 | Withaperuvin H | 5.21293201883158e-05 | 0.000101521593578776 | 8.56623636499869e-05 | 4.95876009278504e-05 | 6.23936379023866e-05 | 5.02566900795618e-05 | 9.63016895922865e-05 | 9.44177201633733e-05 | 4.61965526108461e-05 | 5.02089581889148e-05 | 8.21834736525927e-05 | 9.50306239019724e-05 |
| pos\_1806 | PC(22:5(4Z,7Z,10Z,13Z,16Z)/24:1(15Z)) | 0.000385184996734078 | 0.000205491738336921 | 0.000191353502412688 | 0.000176211655272923 | 0.000199023448920674 | 0.000217081361684951 | 0.000213846347513485 | 0.000201724522861855 | 0.000210450664504938 | 0.000194113281129144 | 0.000209030980710837 | 0.000193628631024685 |
| pos\_1808 | GDP-alpha-D-mannose(2-) | 0.000242167546551452 | 4.06366632505115e-05 | 4.14686479909091e-05 | 2.7176086751944e-05 | 0.000133725997690077 | 0.000117093150902544 | 0.0001049441200001 | 8.70235082674327e-05 | 5.31203500542044e-05 | 7.25938140987641e-05 | 8.13939101896643e-05 | 7.29389995668773e-05 |
| pos\_1809 | Lyciumoside IX | 4.43782497711469e-05 | 5.52205019654041e-05 | 4.76637679553783e-05 | 6.54054825990044e-05 | 4.11367622080833e-05 | 4.93062433354302e-05 | 4.60872186660768e-05 | 3.84363191846255e-05 | 6.78072144921744e-05 | 6.94340682468809e-05 | 4.97265770749784e-05 | 3.30134975581554e-05 |
| pos\_181 | 1-Nitronaphthalene | 3.93122394914488e-05 | 6.37941569541106e-05 | 3.57057377315358e-05 | 3.99270560785498e-05 | 6.1392676665506e-05 | 3.93033270347627e-05 | 2.57183798920808e-05 | 3.17361112643395e-05 | 4.82750469101071e-05 | 1.92047066357992e-05 | 3.34101140680352e-05 | 4.2058865642878e-05 |
| pos\_1810 | Deoxyadenosine monophosphate | 7.62779449984339e-07 | 8.06509462893727e-07 | 4.27300177008652e-05 | 6.07743470926873e-06 | 1.0559898066231e-06 | 6.78169024184288e-06 | 1.85787338865426e-05 | 1.40879254433435e-05 | 2.98327363235206e-06 | 3.07778497463131e-07 | 8.50461097608078e-06 | 1.46552803337834e-05 |
| pos\_1811 | 4-O-Caffeoyl-3-O-feruloylquinic acid | 1.18181996675146e-05 | 2.10541909219676e-05 | 6.60702972406035e-06 | 1.01498042014873e-05 | 1.41728211770825e-05 | 1.12909311316777e-05 | 1.15038979731332e-05 | 9.4450218817416e-06 | 1.44504346442182e-05 | 1.65676694248015e-05 | 1.28705445944625e-05 | 1.02182552559822e-05 |
| pos\_1812 | Isoxanthohumol | 6.31761511484638e-05 | 5.44026583913967e-05 | 1.64536333754164e-05 | 3.78082352215316e-05 | 3.95302860565324e-05 | 4.37797159486178e-05 | 3.02245251531555e-05 | 2.32644487894956e-05 | 3.04316996001323e-05 | 6.56323039502375e-05 | 3.43995704570414e-05 | 2.55070269047271e-05 |
| pos\_1813 | Feruperine | 5.72454957552116e-06 | 3.26604857169769e-05 | 1.3117135371083e-05 | 1.96728752545129e-05 | 6.95758463153017e-05 | 2.64126778551546e-05 | 8.38261298123941e-05 | 6.00295303725122e-05 | 6.22585069152787e-06 | 1.91904013730521e-05 | 4.23860619805084e-05 | 1.38767754862767e-05 |
| pos\_1814 | (S)-4',5,7-Trihydroxy-6-prenylflavanone | 7.97114844209501e-06 | 5.29944600299884e-06 | 0 | 4.06620153865435e-06 | 1.62208016224712e-06 | 2.20523172335332e-06 | 1.21168733167241e-06 | 0 | 0 | 8.16029984253256e-06 | 2.87034654307352e-07 | 0 |
| pos\_1815 | Fluvoxamine | 8.92844746332223e-05 | 4.50319038111495e-05 | 0.00018279665348197 | 0.000183718003816828 | 2.95633812354508e-05 | 8.33381299334042e-05 | 2.63016318488782e-05 | 0.000192617534421654 | 0.000122591986113484 | 2.84615227774906e-05 | 0.000173233576750277 | 0.000464466733626295 |
| pos\_1817 | 3,4-Dihydroxymandelic acid | 1.40676863905235e-05 | 2.21521206751895e-05 | 4.38401597277128e-05 | 1.68856597627503e-05 | 1.16663428921606e-05 | 2.29299507863105e-05 | 3.00014578994418e-05 | 3.10066145562298e-05 | 1.71223757155686e-05 | 1.2919630997126e-05 | 2.39867140172656e-05 | 3.45785278214677e-05 |
| pos\_1819 | PS(14:0/18:3(9Z,12Z,15Z)) | 5.12902784984379e-06 | 5.58367665666403e-06 | 4.43077800120981e-05 | 1.63724977987577e-05 | 1.08445866270275e-05 | 8.48429036378211e-05 | 1.89498700718786e-05 | 2.97049131060438e-05 | 4.7146383592421e-05 | 6.21645734533185e-05 | 2.40267206198694e-05 | 1.80857377810863e-05 |
| pos\_1820 | Edulisin I | 0.000293776266539317 | 0.000261108258228178 | 0.000102898963993551 | 0.000304810735446982 | 0.000233921772395635 | 0.000241912534329992 | 0.000208691657446914 | 0.000138510136732917 | 8.86960650881635e-05 | 0.000133518097459212 | 0.000131180981887268 | 9.46055752621127e-05 |
| pos\_1821 | D-1-[(3-Carboxypropyl)amino]-1-deoxyfructose | 3.39416661547027e-05 | 5.62682486984017e-05 | 4.23137676962804e-05 | 1.06169848002743e-05 | 1.6463528590477e-05 | 2.94157510420876e-05 | 7.25947193899308e-06 | 1.44540750761969e-05 | 1.50468338750919e-05 | 1.10903338585928e-05 | 1.31978608859946e-05 | 2.38204541273966e-05 |
| pos\_1822 | cis-p-Menthane-1,7,8-triol | 7.96652387019884e-05 | 9.70267358950815e-05 | 8.54200149830693e-05 | 0.000165207142377339 | 0.00038181674351964 | 8.85681205374321e-05 | 8.64777972966203e-05 | 8.78406218262906e-05 | 9.10284578752133e-05 | 8.38960951768116e-05 | 8.84991011762871e-05 | 9.06770755890402e-05 |
| pos\_1823 | Elatoside D | 1.44816853046485e-05 | 2.82334730359694e-05 | 2.84172494842616e-05 | 1.17468221179745e-05 | 2.41309253237203e-05 | 1.5193994501868e-05 | 4.31246151748593e-05 | 3.37855165508547e-05 | 1.46950874602359e-05 | 2.59563886615813e-05 | 2.01261951896895e-05 | 2.035720121594e-05 |
| pos\_1826 | (3beta,5alpha,9alpha,14alpha,22E,24R)-3,5,9,14-Tetrahydroxyergosta-7,22-dien-6-one | 0.000375349016531848 | 0.000221309157888244 | 5.6569959462156e-05 | 0.000206591709431079 | 0.000245856230292932 | 0.000234145884860547 | 0.000162585610680252 | 0.000177212093914633 | 0.000213261647170761 | 0.000248661172828877 | 0.00017857964339544 | 0.000184387448396526 |
| pos\_1829 | (+)-Myrtenyl formate | 5.29996927882098e-05 | 6.19213453831317e-05 | 5.96877064709682e-05 | 5.75165517189185e-05 | 5.40245833311757e-05 | 5.58579442010288e-05 | 6.61816488802401e-05 | 6.12967800149565e-05 | 6.63289120257442e-05 | 5.73661574059156e-05 | 6.18162964604773e-05 | 5.24794233737961e-05 |
| pos\_183 | Oleoyl 3-carbacyclic Phosphatidic Acid | 0.000209487815180613 | 0.000208923617902992 | 0.000200666247742172 | 0.000262102787907193 | 0.000179110533652231 | 0.000209613131616949 | 0.000199256224383812 | 0.000199101214225797 | 0.000202704330948903 | 0.000185616111629341 | 0.000217518864892113 | 0.000226584446202372 |
| pos\_1830 | 3a,4b,12a-Trihydroxy-5b-cholanoic acid | 0.000880769040229019 | 0.00106462527190413 | 0.00104216894900294 | 0.00187446539094871 | 0.00162243467843926 | 0.00136526858066136 | 0.00159236038565578 | 0.00145565643752487 | 0.000885659808400376 | 0.000968553005545629 | 0.00122780988914734 | 0.000894931948343417 |
| pos\_1831 | Cycloalliin | 3.2649805806983e-06 | 5.03352416599201e-06 | 5.23068747360298e-06 | 3.89157064663828e-06 | 1.86191362072863e-06 | 3.50011161257519e-06 | 3.29015414159718e-06 | 4.19888271682618e-06 | 4.63786592189737e-06 | 2.77886957619795e-06 | 4.41515252952794e-06 | 3.97283302105025e-06 |
| pos\_1832 | TG(20:0/a-13:0/8:0) | 0.00050808026209082 | 0.000127920664697351 | 0.000133359215381043 | 0.000109712733953596 | 0.000297363345055639 | 0.000291433332294566 | 0.000259367486948867 | 0.00022444273124541 | 0.000167181041063151 | 0.000191393761602523 | 0.000210251631997905 | 0.000194339433143153 |
| pos\_1833 | PC(22:5(4Z,7Z,10Z,13Z,16Z)/22:6(4Z,7Z,10Z,13Z,16Z,19Z)) | 4.57768659351666e-05 | 3.54200402164468e-05 | 2.28822135078201e-05 | 1.45656315496084e-05 | 1.77280417822524e-05 | 1.53670280439253e-05 | 1.6613017082694e-05 | 1.22990768183517e-05 | 1.48719286962278e-05 | 8.95333046315138e-06 | 1.33626234625021e-05 | 1.69128485358349e-05 |
| pos\_1834 | (Z,Z)-2,9,16-Heptadecatriene-4,6-diyn-8-ol | 2.64768517463062e-06 | 6.25310223604237e-06 | 3.4404814179057e-07 | 3.76702358034263e-06 | 1.01149832115804e-05 | 5.11569007523731e-06 | 3.41886783988525e-07 | 7.31359577874189e-07 | 3.30834818012272e-07 | 6.07781045097487e-07 | 4.1644510230494e-07 | 3.76551337480178e-07 |
| pos\_1835 | Calcidiol | 2.51535704549311e-05 | 2.63232688925491e-05 | 3.62129539431437e-05 | 5.19042773729954e-05 | 3.91335754994398e-05 | 3.61216095551689e-05 | 2.74558701475993e-05 | 2.88560342356184e-05 | 3.1676019848187e-05 | 2.85895636406529e-05 | 3.05586799567012e-05 | 3.42200894490071e-05 |
| pos\_1836 | Achyranthoside C | 3.20457939447881e-05 | 3.88756741274461e-05 | 3.52851613270342e-05 | 0.00020113064978134 | 2.30018630560856e-05 | 2.9142222482522e-05 | 1.11900094690031e-05 | 7.10074829563466e-05 | 0.000183607047098556 | 4.83897669372865e-05 | 0.000206297084862995 | 0.000135733166855599 |
| pos\_1837 | 5,8-Dihydroxy-3-(4-hydroxybenzyl)-7-methoxy-4-chromanone | 8.66413325229808e-06 | 1.22270326948571e-05 | 1.71316835786575e-05 | 1.03157062605716e-05 | 8.0594063418319e-06 | 1.18846850857517e-05 | 1.18259017482657e-05 | 1.10751447789398e-05 | 1.19616374908309e-05 | 1.10691972615474e-05 | 1.29367669447655e-05 | 9.3077401090876e-06 |
| pos\_1838 | LysoPC(P-18:1(9Z)) | 0.000219076987837851 | 0.000118719522161429 | 0.000110821579474404 | 9.43258947899731e-05 | 0.000111273660569086 | 0.000116465936873307 | 0.00011650868068699 | 0.000113397848866988 | 0.000115886153382838 | 0.000107210330936086 | 0.000114426456602728 | 0.000111576610825224 |
| pos\_1840 | 3-hydroxynonanoyl carnitine | 4.01773069758105e-06 | 2.30725640191827e-06 | 2.87783483191556e-06 | 1.81502185936671e-06 | 4.26788161376076e-06 | 2.51349680601565e-06 | 2.56800271779896e-06 | 2.46851323683173e-06 | 2.05445566649193e-06 | 4.20125839127579e-06 | 2.02503150533961e-06 | 2.79526878529403e-06 |
| pos\_1842 | Sinigrin | 2.21432395844564e-05 | 1.06702192937112e-05 | 8.34902446820339e-06 | 2.14363994591658e-06 | 1.81171183413774e-06 | 1.08859272837227e-05 | 1.89021792065968e-07 | 1.13532727934514e-05 | 1.63739987330864e-06 | 1.54979197464912e-06 | 7.36815361932153e-06 | 2.57406045726595e-05 |
| pos\_1843 | Ceforanide | 4.22964510294169e-05 | 5.25493629615794e-06 | 5.48686405123823e-06 | 4.48744799518083e-06 | 2.12678035242103e-05 | 1.83244679028179e-05 | 1.87760424050133e-05 | 1.56910248798796e-05 | 1.11201167038703e-05 | 1.31906186550595e-05 | 1.47764532728295e-05 | 1.08913563313027e-05 |
| pos\_1844 | Vitamin K1 | 4.75534372355787e-05 | 8.51019687260532e-05 | 0.000275359716003577 | 1.42103319834188e-05 | 2.36331340806615e-05 | 7.54562444120095e-05 | 2.76546945854469e-05 | 1.7308716180676e-05 | 2.90238347949456e-05 | 9.7792605550552e-05 | 3.18378679725374e-05 | 5.484068101831e-06 |
| pos\_1846 | YGM 1B | 8.65292108336615e-06 | 9.39914737414905e-06 | 8.03196264611969e-06 | 9.46095229737463e-06 | 8.88633069375188e-06 | 9.93853587531579e-06 | 9.17940182173547e-06 | 9.88094851798314e-06 | 1.08884927133192e-05 | 9.36457591678983e-06 | 1.07421114692693e-05 | 6.6423905843958e-06 |
| pos\_1847 | 1-(s-glutathionyl)-2,4-dinitrobenzene | 2.48770295543089e-06 | 4.65141340178657e-06 | 3.50555198984895e-06 | 5.32087233800263e-06 | 3.59607618662845e-06 | 2.360190042434e-06 | 2.35807700407698e-06 | 2.12543587387192e-06 | 4.38028076702811e-06 | 2.68643438116944e-06 | 2.17915068375752e-06 | 3.73533849508442e-06 |
| pos\_1848 | 2,3-Dihydro-6-methyl-5-(5-methyl-2-furanyl)-1H-pyrrolizine | 0.00045642889853701 | 0.000237330003857737 | 0.000213398238886033 | 0.000207571654112803 | 0.000298273534707146 | 0.000306949638217472 | 0.000332698344978805 | 0.000280624589015913 | 0.000262865492297255 | 0.000279370120252115 | 0.000275590472635206 | 0.000344430996801579 |
| pos\_1849 | Phytosphingosine-1-P | 8.07793583503549e-06 | 1.26003435124141e-05 | 3.73798915093088e-05 | 4.55679484892008e-05 | 2.0191087701515e-05 | 2.24202226763921e-05 | 1.16690790132747e-05 | 1.9947000615495e-05 | 6.62883778362147e-05 | 2.88967850880732e-05 | 2.91536988213633e-05 | 2.797759324207e-05 |
| pos\_185 | Ala Gly Pro Pro | 0.00064373954121452 | 0.000315545584513917 | 0.000214277476444738 | 0.0002390688098436 | 0.000444891344226715 | 0.000423845788879487 | 0.000508576216767506 | 0.000384181467096169 | 0.000307084251457447 | 0.00035531993575325 | 0.000366841992884954 | 0.000337974722648386 |
| pos\_1850 | Armillane | 1.04705033960149e-05 | 1.01489850135547e-05 | 8.82654668607301e-06 | 1.81259604747405e-05 | 7.08275685417249e-06 | 1.05482961252467e-05 | 7.75108333072887e-06 | 1.17598857437999e-05 | 9.51153336073506e-06 | 7.15725551598737e-06 | 9.90340235868403e-06 | 2.15318758601406e-05 |
| pos\_1851 | Calendulaglycoside E | 0.000132822540850954 | 0.000148270299055897 | 0.000147156947091601 | 0.000144231827536965 | 0.000124382819968874 | 0.000157924283989786 | 0.000129999555720575 | 0.000148144097950403 | 0.000153008966162701 | 0.000130415001790724 | 0.000144258234959912 | 0.000140479249979975 |
| pos\_1852 | PE(20:4(8Z,11Z,14Z,17Z)/P-18:0) | 0.000348419551016475 | 0.000484160568568865 | 0.000749238937629108 | 0.000286552803508835 | 0.000354901179914772 | 0.000419755899958163 | 0.000398409710625293 | 0.000441828083380145 | 0.000313350365621672 | 0.000527364655848045 | 0.000627066713707547 | 0.000534674310649874 |
| pos\_1853 | Scillipheosidin 3-[glucosyl-(1->2)-rhamnoside] | 1.90961921195889e-05 | 2.30898844166812e-05 | 2.65985683510646e-05 | 9.05991186210647e-06 | 1.18574120574159e-05 | 1.50671975320074e-05 | 8.40743874852833e-06 | 1.40489944380899e-05 | 6.51696906743386e-06 | 3.12511041656452e-05 | 2.01246883759551e-05 | 1.80423143656985e-05 |
| pos\_1855 | Trihexosylceramide (d18:1/20:0) | 5.11299896581051e-06 | 1.04392103619159e-05 | 1.86298810762523e-05 | 4.48820615235947e-05 | 0.000116155780070843 | 9.34153303326884e-05 | 1.29941849011675e-05 | 9.69744227236938e-06 | 0.00014418193024676 | 1.34126875042269e-05 | 7.7054992247146e-05 | 1.40789426193632e-05 |
| pos\_1856 | Irbesartan | 0.000238543959102406 | 0.000252549670350144 | 0.000201369907420874 | 0.000185897259181561 | 0.000175250161613544 | 0.000204244268074264 | 0.000231861562041354 | 0.000232384991348686 | 0.000210949506964022 | 0.000188952089138042 | 0.000206889615463226 | 0.000184299975882561 |
| pos\_1857 | DG(18:4(6Z,9Z,12Z,15Z)/22:2(13Z,16Z)/0:0) | 0.000207241032433704 | 1.91728127179022e-05 | 1.60481104108214e-05 | 1.06989384326274e-05 | 0.000286548118717452 | 0.000106611725781386 | 0.000440589993149029 | 0.000337675903058598 | 9.7349183604498e-05 | 0.00028077190391138 | 0.000269232889687937 | 0.000241362338933146 |
| pos\_1859 | Rhamnazin 3-rutinoside | 0.000532816607614986 | 0.000137802050249471 | 0.000146580377165243 | 0.00011905315023161 | 0.000308843925864103 | 0.000303483992491548 | 0.000278530361629373 | 0.000243935419697271 | 0.000185418526834008 | 0.000207049766843209 | 0.000232837033365943 | 0.00020991619945318 |
| pos\_186 | 4-[5]-ladderane-butanoic acid | 0.000249903670483553 | 0.0003291033527194 | 0.000316805583261269 | 0.0002672664616519 | 0.000234287594227188 | 0.000272871991009683 | 0.000251248727081232 | 0.000293123479153476 | 0.000266099786300316 | 0.000285103163744463 | 0.000291257094476047 | 0.000273925889420618 |
| pos\_1860 | dGDP | 5.36194776305766e-06 | 9.59947985395949e-06 | 8.21079980613258e-06 | 6.30663737391549e-06 | 3.76773739213682e-06 | 6.67982246669361e-06 | 5.56944669359989e-06 | 5.19487600988901e-06 | 6.14839577525948e-06 | 4.70848474358245e-06 | 5.74903842035625e-06 | 4.40890537639447e-06 |
| pos\_1861 | DG(20:4(5Z,8Z,11Z,14Z)/22:1(13Z)/0:0) | 1.91343086496616e-05 | 1.58963837871072e-05 | 3.92105184237952e-06 | 1.9927297555419e-06 | 3.60979011439667e-06 | 3.09993471116805e-06 | 5.10480013278946e-06 | 4.77580828205097e-06 | 2.19311903681972e-06 | 1.97813556556714e-06 | 2.08182361169291e-06 | 1.23450433978481e-06 |
| pos\_1862 | Ganglioside GA2 (d18:1/9Z-18:1) | 0.000195685648717073 | 0.000115280054291874 | 0.000111975710103413 | 9.94459823802204e-05 | 0.00012463692319795 | 0.000137238802980992 | 0.000152082456062925 | 0.000136267743164824 | 0.00015008577301288 | 0.000120539980208193 | 0.000117928206878564 | 0.000140903826509261 |
| pos\_1863 | 1-hexadecyl-glycero-3-phosphate | 4.36797697104276e-05 | 4.94213275830597e-05 | 7.93597909097052e-05 | 3.95943898380715e-05 | 3.98482286667636e-05 | 5.13414756187831e-05 | 3.37179585462918e-05 | 4.52252285564801e-05 | 3.75497433399768e-05 | 8.31620588674587e-05 | 4.62060941584418e-05 | 5.17439796004807e-05 |
| pos\_1864 | Notoginsenoside H | 0.000477851443701027 | 0.000292116754349483 | 0.000287848421160282 | 0.000262013726027366 | 0.00029247581345377 | 0.000332341130110216 | 0.000337392064467322 | 0.000324297898022127 | 0.000323359637407932 | 0.000295850628928937 | 0.000308112283681169 | 0.000297290257072991 |
| pos\_1865 | PC(18:1(9Z)/18:1(9Z)) | 1.74251640526998e-05 | 4.30335723769222e-06 | 7.24375830260851e-05 | 6.83924884790294e-05 | 0.000189939649822443 | 6.74116426341473e-05 | 5.86976414825567e-05 | 3.45202913937273e-05 | 0.000103227014331044 | 6.70504966670438e-05 | 7.36960862036011e-05 | 6.04933197411385e-05 |
| pos\_1866 | Betavulgaroside II | 3.48805519089673e-05 | 7.36515745014983e-06 | 8.58274188024672e-06 | 3.51491826280872e-06 | 1.24038208235677e-05 | 1.44031629532757e-05 | 2.82425675287365e-06 | 5.65700513800528e-06 | 3.20677413021196e-06 | 2.9531056483882e-06 | 8.08796722486222e-06 | 5.79598025086132e-06 |
| pos\_1868 | 16-Ketoestradiol | 0.000169407912439368 | 9.17935869360749e-05 | 3.28116853793894e-05 | 8.13076425328247e-05 | 3.13708816441336e-05 | 7.4638936097628e-05 | 4.54578118789696e-05 | 4.13982214541523e-05 | 4.61003280706009e-05 | 0.000205342119397906 | 7.62087253477864e-05 | 4.8671607652025e-05 |
| pos\_187 | 2-Hydroxyhomopterocarpin | 3.4978166851647e-05 | 1.76177127601099e-05 | 2.59090717553542e-05 | 1.53408116321071e-06 | 1.52575685264162e-05 | 1.39391178041244e-05 | 2.11893756682834e-07 | 6.43075667347016e-06 | 1.00520447467019e-06 | 4.81004718336389e-05 | 9.2403979987654e-06 | 2.72738989053438e-05 |
| pos\_1872 | Vulgaxanthin I | 0.000122898251414371 | 0.000154331308628527 | 0.000136456291763261 | 0.000133277857875635 | 0.000127381631771754 | 0.000138924162621536 | 0.000117959128973294 | 0.000125259534281989 | 0.000156307068957504 | 0.000109776439741095 | 0.000134962612325546 | 0.000142424888348292 |
| pos\_1873 | (E)-2-(2,4-Hexadiynylidene)-1,6-dioxaspiro[4.5]dec-3-ene | 5.37056926179794e-05 | 3.38183559650815e-05 | 6.5089136020914e-06 | 2.91189895098786e-05 | 4.08540143258882e-05 | 2.85592028570444e-05 | 1.19121087403973e-05 | 8.63384731051796e-06 | 1.01018745243659e-05 | 3.93587871638971e-05 | 1.37538366960678e-05 | 8.96939489082515e-06 |
| pos\_1874 | Sulfametopyrazine | 3.84874219146559e-05 | 2.98795644263889e-05 | 1.68230319433694e-05 | 2.31391146064446e-05 | 2.29437073957809e-05 | 3.35272376636528e-05 | 3.32330072672231e-05 | 2.31313664949635e-05 | 1.95991335248957e-05 | 2.9589022535689e-05 | 2.53374185079077e-05 | 3.91024085339217e-05 |
| pos\_1875 | Kuwanon Q | 0.000321769494594104 | 4.55394591818622e-05 | 3.99310694778268e-05 | 4.07728842991283e-05 | 0.000153477667063586 | 0.000148276061902861 | 0.000114247278352166 | 8.39405257136697e-05 | 4.41084486017371e-05 | 7.23045491770086e-05 | 8.04452018521303e-05 | 6.88633088734392e-05 |
| pos\_1876 | PGP(16:0/18:1(11Z)) | 1.51914396233899e-05 | 8.84029485166375e-06 | 2.18277155739451e-05 | 1.78499448690112e-05 | 2.46919725145635e-06 | 1.20402017821468e-05 | 9.32890528082435e-06 | 8.53743634395508e-06 | 6.28904353379302e-06 | 3.56313439779402e-06 | 1.15408746058953e-05 | 8.07690802173327e-06 |
| pos\_1877 | Albigenic acid | 4.19735032777377e-05 | 3.84090468112887e-05 | 2.19137536897679e-05 | 3.75527428666188e-05 | 2.96926505206843e-05 | 3.22957082162594e-05 | 6.11479702723905e-05 | 5.67899374111139e-05 | 3.54110472094531e-05 | 6.57971778770263e-05 | 4.73116385046316e-05 | 6.00754296054173e-05 |
| pos\_1878 | (±)-Rollipyrrole | 2.79921133933176e-06 | 2.64195719880272e-06 | 1.49592524057624e-06 | 1.1630285569869e-06 | 6.40538748190744e-06 | 2.16521993632177e-06 | 2.10830993248764e-05 | 1.06088306992744e-05 | 1.20814007541974e-06 | 1.09936800026234e-05 | 1.58349182974329e-05 | 7.70444353794624e-06 |
| pos\_1879 | 4-Hydroxy-16,18-tritriacontanedione | 1.18822384149492e-05 | 2.45160549810586e-05 | 1.99056614388159e-05 | 2.14489787316075e-05 | 1.46604925081418e-05 | 1.90429023459422e-05 | 2.05340990150392e-05 | 1.6368358705574e-05 | 2.09686258269192e-05 | 2.53028507636978e-05 | 2.46609552221435e-05 | 2.40307595272882e-05 |
| pos\_188 | Dihydroactinidiolide | 5.02243212753483e-05 | 8.57321255893612e-05 | 0.000128866015966484 | 0.000113169510207808 | 8.1872590999019e-05 | 9.26379843735886e-05 | 6.50443652376988e-05 | 8.81511729233323e-05 | 7.21410860377687e-05 | 6.45535509640268e-05 | 8.04772941601401e-05 | 0.000109711442949573 |
| pos\_1880 | MG(0:0/22:4(7Z,10Z,13Z,16Z)/0:0) | 0.000300382686781557 | 0.000351333638397156 | 0.000335808214146301 | 0.000424817173192034 | 0.000356708664632436 | 0.000354916456006169 | 0.000351550840764704 | 0.000330765053852649 | 0.000382194403070598 | 0.000331866015108443 | 0.000315657609215568 | 0.000234473050345133 |
| pos\_1881 | Cryptochrome | 0.0146605944639966 | 0.0196004426935957 | 0.0147763987766176 | 0.0170699912854881 | 0.0130739883438543 | 0.0170806006182452 | 0.0141215871450358 | 0.0163151220223048 | 0.0189226233069434 | 0.0151526120308055 | 0.0166762294377603 | 0.012708989570621 |
| pos\_1882 | Trigofoenoside E1 | 0.000600385099403999 | 0.000613447060791648 | 0.000613722141896291 | 0.000378178675627813 | 0.00052815791301476 | 0.000603263612760782 | 0.000302368622407031 | 0.000552285504631489 | 0.000849166638828303 | 0.00037684714950454 | 0.000569490568498356 | 0.000916967952098008 |
| pos\_1883 | 3-Ethyl-5-methyl-1,2-cyclopentanedione | 7.10283099679514e-06 | 7.85031714985735e-06 | 7.32404934443545e-06 | 6.92374781121181e-06 | 6.10549485097133e-06 | 6.72893045230431e-06 | 6.62403303344139e-06 | 6.63318818753565e-06 | 7.92065944733279e-06 | 6.40004069848746e-06 | 6.31140065179e-06 | 6.15569353722852e-06 |
| pos\_1884 | Punicacortein A | 0.000145339029515776 | 1.40209311263639e-05 | 1.58916567909615e-05 | 1.1033769308921e-05 | 7.91556201108646e-05 | 5.40564881099323e-05 | 5.60910655539934e-05 | 3.97421439750373e-05 | 2.04563411825201e-05 | 2.98100644303512e-05 | 3.01666840903204e-05 | 3.65177212592981e-05 |
| pos\_1885 | Clindamycin | 6.1967426161066e-06 | 9.09721323088739e-06 | 5.48110285369143e-06 | 8.25355837979242e-06 | 7.15969631700574e-06 | 6.34345642189667e-06 | 9.45251022014605e-06 | 6.98958141751179e-06 | 7.89574530565825e-06 | 5.81967851394129e-06 | 6.55453635948191e-06 | 5.73226634176144e-06 |
| pos\_1887 | Mesoridazine | 4.9550189732132e-05 | 7.98154300433005e-05 | 5.11860923515612e-05 | 4.65369378386832e-05 | 5.60982961821115e-05 | 4.9949659542925e-05 | 8.56447392562329e-05 | 7.42307517431072e-05 | 4.58884276753859e-05 | 5.09832152626698e-05 | 6.78811868076883e-05 | 5.91536720364751e-05 |
| pos\_1888 | Garcilivin A | 3.01386712666339e-05 | 5.40038178010607e-05 | 4.78373524626097e-05 | 4.07208032761835e-05 | 3.5364194032039e-05 | 4.52829074759436e-05 | 3.61276211745353e-05 | 4.4888303341361e-05 | 5.69258942153764e-05 | 4.22672931571562e-05 | 4.18842280787776e-05 | 3.91414093765988e-05 |
| pos\_1889 | 14,17-epoxy-15-methyldocosa-14,16-dienoic acid | 0.000133799114460643 | 0.000192550682729663 | 0.000174744564697065 | 0.000153356147810821 | 0.000136427020189274 | 0.000157880714294325 | 0.000157484000505971 | 0.000156488232008712 | 0.000178473767624696 | 0.000150818917145546 | 0.000144398243084318 | 0.000147716268628443 |
| pos\_189 | His Glu Phe | 6.02527411205878e-05 | 0.000191717232958813 | 0.000138603443740746 | 3.58150164075961e-05 | 1.34149303000417e-05 | 8.47721333403703e-05 | 3.45505899078211e-05 | 3.61713476141253e-05 | 2.37802965140885e-05 | 0.000136440383239046 | 5.06090222973678e-05 | 3.67845727214878e-05 |
| pos\_1891 | PC(18:1(11Z)/22:5(4Z,7Z,10Z,13Z,16Z)) | 0.000279503929559622 | 0.000105102869113957 | 9.68991924865246e-05 | 9.17019414097382e-05 | 0.000129593595841726 | 0.000132596651952638 | 0.000150593835817696 | 0.000124516143924237 | 0.000112783601138514 | 0.000115424855369849 | 0.000122264269026927 | 0.000120778604176657 |
| pos\_1892 | Cyclomammeisin | 1.27824179754574e-05 | 1.20502880933244e-05 | 2.22714960691813e-05 | 6.64729764461047e-06 | 4.4540443394335e-05 | 2.01646237064945e-05 | 4.19098357006188e-05 | 3.48563195236227e-05 | 1.23743473636353e-05 | 3.97914996106718e-05 | 3.1915986322656e-05 | 3.30955921979514e-05 |
| pos\_1893 | Ethambutol | 2.62662318812692e-05 | 3.19623384376055e-05 | 2.9609456919924e-05 | 2.65341701097922e-05 | 2.35820136609035e-05 | 2.6581858896287e-05 | 2.50257181257686e-05 | 2.55141154817294e-05 | 2.95510427657528e-05 | 2.52517048892366e-05 | 2.68552428403513e-05 | 2.41607022457286e-05 |
| pos\_1894 | Spirolide E | 0.00271842813609266 | 0.00317422551682698 | 0.00238131815377766 | 0.00298835816244602 | 0.00235411601952061 | 0.0024139222719569 | 0.00212992903861921 | 0.00219841098038403 | 0.00273402001687506 | 0.00222939130271802 | 0.00252620170869663 | 0.00228296407589385 |
| pos\_1895 | Nilutamide | 9.20638121374917e-07 | 1.77092920985009e-06 | 4.29691165464405e-06 | 1.80472277674227e-06 | 1.36859838490032e-06 | 2.3345174884994e-06 | 2.19373599677917e-06 | 2.06263854088426e-06 | 2.09121532420964e-06 | 1.96196238791097e-06 | 2.65850130128806e-06 | 1.48358111761689e-06 |
| pos\_1896 | Glycyl-glycine | 5.70509514790489e-06 | 2.080286302458e-05 | 1.0014141656536e-06 | 8.65496524987746e-07 | 2.79272530432352e-06 | 4.7725883584028e-06 | 0 | 1.46376495364347e-06 | 0 | 3.03101046567287e-06 | 1.87841296235361e-06 | 1.04183849458684e-06 |
| pos\_1897 | Cyclopassifloic acid E | 3.38499430355422e-05 | 5.45126039171563e-06 | 2.3242123993819e-05 | 0.000304749959923254 | 3.20029896290795e-06 | 6.77758547840616e-05 | 2.46007615857704e-05 | 3.66596476083036e-05 | 5.66897629908805e-06 | 1.41668732791946e-05 | 2.83203343616589e-05 | 6.0810608257061e-05 |
| pos\_1898 | PS(18:0/20:0) | 5.642284929301e-05 | 8.62067304939466e-05 | 0.000247896859520216 | 4.70501638659576e-05 | 0.000110763223856392 | 0.000122187301134695 | 0.000128641170721635 | 0.000139986729184714 | 6.69299489325627e-05 | 0.000198096920080098 | 0.000233127377764849 | 0.000263253589638172 |
| pos\_1899 | Ergothioneine | 1.19213902522001e-05 | 1.17015607027979e-05 | 1.12151515883082e-05 | 1.23708602862353e-05 | 1.2795645856073e-05 | 1.27251366028077e-05 | 6.91123286441066e-06 | 7.84265873337014e-06 | 1.52353829006076e-05 | 1.14299260964761e-05 | 1.22399272377404e-05 | 1.04039022995409e-05 |
| pos\_19 | Cellobiose | 0.000754628799211186 | 0.000689185942934045 | 0.000750556103184053 | 0.000621720538083001 | 0.000592356481098528 | 0.000797793232519584 | 0.000725419231660012 | 0.000700631676020376 | 0.00081862163465382 | 0.000756055464885327 | 0.000778064113374993 | 0.000777630754093582 |
| pos\_190 | 1-Linoleoyl Glycerol | 0.000545204300247028 | 0.000353798051885545 | 0.0005061831348606 | 0.000495874049657757 | 0.000299944515356895 | 0.000467596168335896 | 0.000339283339385255 | 0.00037649492751046 | 0.000284576462966253 | 0.000275214110136873 | 0.000487094802511239 | 0.000449220664709464 |
| pos\_1900 | Prednisone | 6.72733902283146e-05 | 0.000100115290555478 | 5.35039802318278e-05 | 7.446678574823e-05 | 6.40790461219009e-05 | 6.68002800462343e-05 | 7.09084368705511e-05 | 6.10700957073848e-05 | 6.24281347757275e-05 | 7.47466513543505e-05 | 9.79081453429503e-05 | 5.15757227541451e-05 |
| pos\_1901 | Nb-Feruloyltryptamine | 5.55280073315982e-05 | 7.48858238438787e-05 | 4.87660089169946e-05 | 5.70357538647828e-05 | 5.55404620180546e-05 | 5.30548255524653e-05 | 6.14141822401016e-05 | 5.59347345288305e-05 | 5.62800939656655e-05 | 5.52260413849149e-05 | 5.51557872303681e-05 | 4.7561240957276e-05 |
| pos\_1902 | Hygromycin B | 5.52059532889937e-06 | 4.96674919147518e-06 | 1.63917689020252e-06 | 4.16438822473417e-06 | 6.3949279062178e-06 | 4.37099854969071e-06 | 4.8303268503691e-06 | 4.20287869903565e-06 | 4.08526353596306e-06 | 5.91479793466062e-06 | 3.63602485900873e-06 | 4.80014953387578e-06 |
| pos\_1903 | Gladiatoside B3 | 3.17200811460363e-05 | 3.05114252440815e-05 | 1.50766246608634e-05 | 1.84251653178494e-05 | 4.33383379188747e-05 | 3.54028123822028e-05 | 3.20385233819809e-05 | 2.24879592667725e-05 | 2.96254241263195e-05 | 1.74549533845537e-05 | 2.67792413975358e-05 | 1.98393828470214e-05 |
| pos\_1904 | Linoleic acid | 1.87766409880479e-05 | 1.83875779879536e-05 | 1.24145871789618e-05 | 1.53436541184723e-05 | 1.64412058384966e-05 | 1.3506173089652e-05 | 9.3103330168534e-06 | 2.2536110288283e-05 | 1.35935557105537e-05 | 9.33605066108909e-06 | 1.77229168107357e-05 | 1.44932792586674e-05 |
| pos\_1905 | Lisdexamfetamine | 0.000413274830484313 | 0.000258791020114456 | 0.000209965043247201 | 0.000241806277349171 | 0.000295092503158559 | 0.000291515972432752 | 0.000306866111918839 | 0.000289983834997495 | 0.00060072978905 | 0.000174622660577447 | 0.000334780957474246 | 0.000260394467729121 |
| pos\_1907 | Methyl (9Z)-6'-oxo-6,5'-diapo-6-carotenoate | 0.000125055143019783 | 3.53104599891683e-05 | 3.54029029639712e-05 | 2.83571267390091e-05 | 7.58997668875713e-05 | 7.1130594358771e-05 | 7.05046535778113e-05 | 6.23363034920216e-05 | 5.07468841702161e-05 | 4.84920113849488e-05 | 5.86937742520264e-05 | 5.18316757536237e-05 |
| pos\_1908 | Calendasaponin B | 6.62961648231401e-05 | 4.15257327742404e-05 | 4.12751908370587e-05 | 3.20705248359784e-05 | 3.46795220020815e-05 | 2.65541554270807e-05 | 2.31711022761307e-05 | 1.52433673859452e-05 | 2.94412644439757e-05 | 2.38587833094869e-05 | 1.88076684602546e-05 | 1.81368316413224e-05 |
| pos\_191 | Pfaffic acid | 0.00554351973029737 | 0.00571596478492247 | 0.00368682698774621 | 0.00777895726917029 | 0.00695663621137438 | 0.00655274581861659 | 0.00492967113784619 | 0.00535610215235919 | 0.00684328706256829 | 0.00559686117797696 | 0.00636412168836654 | 0.0049261333355405 |
| pos\_1912 | Quercetin 4',7-diglucoside | 0.000403893034863225 | 9.97300755453373e-05 | 0.000121432305972942 | 8.97063878801197e-05 | 0.000233419186266953 | 0.000235571982419755 | 0.000206801254025396 | 0.000186213003935038 | 0.00015019005574806 | 0.000155867953299674 | 0.000175029618031978 | 0.000162786888003209 |
| pos\_1913 | Nummularine B | 4.45778072659227e-05 | 1.26206588659166e-05 | 9.25099977192354e-06 | 8.75760792785651e-06 | 3.49643113532501e-05 | 2.7003416082868e-05 | 3.07055082640842e-05 | 2.35458784096291e-05 | 1.6103884216685e-05 | 1.83361507455985e-05 | 2.31644860122834e-05 | 2.02342464515656e-05 |
| pos\_1915 | 1-(9H-Pyrido[3,4-b]indol-1-yl)-1,4-butanediol | 6.38221313054648e-05 | 1.57963597609369e-05 | 6.86080281406071e-06 | 6.36254267756249e-06 | 3.06569246034305e-05 | 2.83017395418819e-05 | 2.1797171804062e-05 | 1.47873848898957e-05 | 1.49972726156039e-05 | 1.19628483564284e-05 | 1.34299124446631e-05 | 1.41644890293342e-05 |
| pos\_1917 | 3'-Deaminofusarochromanone | 0.000119273773592025 | 9.364225030884e-05 | 9.52876515081652e-05 | 0.000106436028235151 | 0.000129007367765583 | 0.000119197334859798 | 0.000199888085784674 | 0.000153731121093064 | 9.38370821228119e-05 | 0.000150717419275161 | 0.000154289162044842 | 0.000145279763124455 |
| pos\_1918 | Molindone | 0 | 0 | 0 | 2.7083449092956e-06 | 0 | 0 | 0 | 1.09973198402141e-06 | 0 | 0 | 0 | 1.27985486626865e-05 |
| pos\_192 | 4-Acetyl-6-tert-butyl-1,1-dimethylindane | 3.98174131875597e-05 | 5.58109192592473e-05 | 9.30065714280904e-05 | 7.52896012683023e-05 | 4.83477493303108e-05 | 6.30000878513963e-05 | 4.41148936112086e-05 | 7.00310179265976e-05 | 4.58701072525676e-05 | 4.5495419060149e-05 | 6.46040828687306e-05 | 0.00010850939742781 |
| pos\_1920 | CPA(18:1(11Z)/0:0) | 9.29124664832951e-06 | 7.25071159165278e-06 | 3.89768601548959e-05 | 4.64903492065118e-07 | 7.80167264004255e-07 | 1.01472073225573e-05 | 3.2508651414416e-06 | 1.90683853140584e-05 | 2.96722507653615e-06 | 9.30341767442417e-06 | 1.4096640039322e-05 | 4.49713096392043e-05 |
| pos\_1921 | Corchoionoside B | 4.09051787153936e-06 | 1.32392366628679e-05 | 1.18383802405526e-06 | 4.07154536565783e-06 | 3.00846012199697e-06 | 3.95833422563687e-06 | 1.0347153838228e-05 | 6.65270777730367e-06 | 1.73984663795045e-06 | 1.10337989166651e-05 | 5.61152918230637e-06 | 1.60894566889954e-06 |
| pos\_1922 | Monocrotaline | 2.7943239101155e-05 | 3.41248874677211e-05 | 1.37472530848427e-05 | 2.1184922695033e-05 | 3.044901909106e-05 | 2.26181013898501e-05 | 1.9717604934982e-05 | 1.76174340760125e-05 | 2.13505545281667e-05 | 1.69836496717694e-05 | 1.90550168837915e-05 | 1.91626392904881e-05 |
| pos\_1923 | Miltirone | 0.00212536859683121 | 0.00116645787902525 | 0.000968259729594823 | 0.000934181234403141 | 0.00144812385075895 | 0.00144344141221121 | 0.00154681656773176 | 0.00126921572116091 | 0.00121920998418896 | 0.00123052739807571 | 0.00134437672978144 | 0.00129292138800214 |
| pos\_1924 | Tyrosyl-Hydroxyproline | 5.13875207692201e-05 | 5.85552593990586e-05 | 5.87293982983448e-05 | 7.29550844498793e-05 | 4.91699857006227e-05 | 5.17713488715683e-05 | 5.52870448067867e-05 | 5.27970889907015e-05 | 5.44229518495115e-05 | 5.9608384860465e-05 | 6.26485309374183e-05 | 5.23028827488291e-05 |
| pos\_1926 | PG(16:1(9Z)/18:1(9Z)) | 0.000540373907298384 | 0.000780711279997142 | 0.00105334643483915 | 0.00073768780637041 | 0.000835390688619478 | 0.000871048928010462 | 0.000615272000318006 | 0.000701361812082495 | 0.000871981633588756 | 0.000721713475649827 | 0.000707448615937722 | 0.000991463382810812 |
| pos\_1927 | Glutamyl-Lysine | 0.00824769522277654 | 0.00645656766729617 | 0.0108089688380562 | 0.00679009668278375 | 0.0110423999920611 | 0.00795290404372468 | 0.00515252143144847 | 0.00599918616472132 | 0.00528452113251467 | 0.00728689010436488 | 0.00576137187116136 | 0.00682219592238915 |
| pos\_1928 | 15(S)-Hydroxyeicosatrienoic acid | 0.000308801079713891 | 0.000378208678944288 | 0.000357816457316942 | 0.000825652318184179 | 0.000336631631052369 | 0.000451986744667861 | 0.000514053959532839 | 0.000357171642100264 | 0.000551175809995332 | 0.000424883737032964 | 0.000442817137322634 | 0.000453819368171485 |
| pos\_1929 | Levorphanol | 0.000172507209194963 | 0.000214119237067817 | 0.000202156538397303 | 0.000181759540923747 | 0.000154817763892128 | 0.000182950210425409 | 0.000162588470786407 | 0.000184463473241919 | 0.000195964665191149 | 0.000195125136054076 | 0.000187999758533603 | 0.000180017181619562 |
| pos\_193 | 1,3-Diisopropylbenzene | 8.03485514577717e-06 | 1.33983148908262e-05 | 1.34943478521546e-05 | 1.31324870418638e-05 | 1.0815575729779e-05 | 1.08122022204921e-05 | 1.12038074992114e-05 | 1.2773401643972e-05 | 1.17949907510007e-05 | 1.04725804766796e-05 | 9.66421070090681e-06 | 1.09628587007271e-05 |
| pos\_1930 | Cyanidin 3-(6''-p-coumarylsambubioside) | 4.01633414125464e-05 | 3.12678243343938e-06 | 4.19974866261451e-06 | 2.01525228250082e-06 | 2.12428215620013e-05 | 1.78154063012919e-05 | 1.25956621556011e-05 | 7.53640500279863e-06 | 4.83997474639403e-06 | 6.36516774957489e-06 | 6.49931458935444e-06 | 2.67194512717079e-06 |
| pos\_1931 | DG(18:3(6Z,9Z,12Z)/22:5(4Z,7Z,10Z,13Z,16Z)/0:0) | 1.48821082908495e-05 | 1.29746975472143e-05 | 3.39741508096164e-05 | 3.14832122391749e-05 | 2.52865083682045e-06 | 6.67815458858633e-06 | 1.84383797048345e-05 | 1.27174527514353e-05 | 4.49696036733975e-06 | 3.13056578795409e-06 | 4.44765589026226e-06 | 3.3214979698296e-06 |
| pos\_1934 | PC(20:3(5Z,8Z,11Z)/24:0) | 0.000221950349615676 | 0.000306435749234891 | 0.000298669164519401 | 0.000329000545403334 | 0.000180334369153527 | 0.000209980530803575 | 0.00017645741929698 | 0.000253943246111637 | 0.000215937837632288 | 0.000198213559792141 | 0.000209708738198085 | 0.000237401091790106 |
| pos\_1936 | Etamiphylline | 0.00051432578939224 | 0.000702397700926801 | 0.000410895667784308 | 0.000497314982864111 | 0.000685637358845924 | 0.000598151322234691 | 0.000499023381679631 | 0.000525105722562929 | 0.000801327850053749 | 0.000745190517734713 | 0.00065841689215097 | 0.000525821073342249 |
| pos\_1937 | Tryptophyl-Threonine | 5.07451722535593e-06 | 4.85281029151619e-06 | 7.05560328798261e-06 | 5.82491277172949e-06 | 6.05168970024091e-06 | 7.15987453934819e-06 | 4.16737481898311e-06 | 5.5752248801832e-06 | 6.75928871413763e-06 | 3.66666313649895e-06 | 5.98466024975989e-06 | 4.92248984874438e-06 |
| pos\_194 | Tetrahydrofurfuryl cinnamate | 2.85243731828288e-05 | 1.89089468573599e-05 | 0.000108639168909307 | 1.2080725749077e-05 | 1.17827857711677e-05 | 3.28361214331345e-05 | 7.78635798089974e-06 | 3.78758821383126e-05 | 6.57066834671759e-06 | 6.17469624723199e-05 | 4.51957639139785e-05 | 9.61672779542794e-05 |
| pos\_1940 | Hydroxy-alpha-sanshool | 3.22279300983568e-05 | 3.47172011439958e-05 | 7.03356367145444e-05 | 8.92884688866555e-05 | 2.90703905643892e-05 | 4.58166253492989e-05 | 2.9036415165411e-05 | 4.48077774516632e-05 | 4.88703153085361e-05 | 3.1844438350767e-05 | 4.4334495571632e-05 | 6.86740609244335e-05 |
| pos\_1941 | Argininic acid | 1.42391152401105e-05 | 8.80010145045111e-06 | 7.01832171255975e-06 | 7.68302404830194e-06 | 1.2049344381399e-05 | 9.48644958076488e-06 | 1.08916283761377e-05 | 8.94509246762977e-06 | 9.53807129229539e-06 | 8.7479477805328e-06 | 9.20903278077072e-06 | 1.04933979728445e-05 |
| pos\_1943 | Melilotoside D | 3.18000425263636e-05 | 3.79238844326184e-05 | 1.62867970747932e-05 | 1.02172091244492e-05 | 1.08241338668666e-05 | 1.23490084823195e-05 | 1.41344025579401e-05 | 1.58184242404423e-05 | 1.34172487593235e-05 | 1.21838798149784e-05 | 1.1366498958756e-05 | 1.83659643088841e-05 |
| pos\_1944 | 5,8-Epoxy-5,8-dihydro-3-hydroxy-8'-apo-b,y-carotenal | 1.37295998850329e-05 | 8.88995552939456e-06 | 1.7106977437975e-06 | 6.90560160122048e-06 | 9.68033933449528e-06 | 8.11037418995656e-06 | 8.54983568588769e-06 | 7.67029205983283e-06 | 7.23572597756248e-06 | 8.84322122677202e-06 | 9.68254225566155e-06 | 1.11108762874544e-05 |
| pos\_1946 | Acetylsoyasaponin A5 | 1.33689523227887e-05 | 1.54459421138921e-05 | 1.98432481374343e-05 | 1.17413345207605e-05 | 2.02783079555416e-05 | 1.17622085743386e-05 | 3.62524236384399e-05 | 1.82268898658428e-05 | 1.13281165965038e-05 | 2.362755812304e-05 | 2.02753610346067e-05 | 1.85517439686118e-05 |
| pos\_1947 | PS(DiMe(11,5)/MonoMe(13,5)) | 2.41497568145594e-05 | 1.70131795978375e-05 | 1.88189811482142e-05 | 0.000218007054595087 | 0.000332979476930998 | 2.06721150141573e-05 | 1.36084254379078e-05 | 5.52287112111273e-05 | 0.000248994536486595 | 5.86825180590602e-05 | 1.59791044265863e-05 | 1.84184459170915e-05 |
| pos\_1949 | 1-Palmitoylglycerophosphoinositol | 0 | 8.97364696760771e-08 | 2.77529189722473e-05 | 1.96719930579031e-05 | 3.26629683610684e-06 | 8.14610724505924e-06 | 1.03229196633667e-05 | 2.64398569936636e-05 | 2.20803628177473e-09 | 1.26198644707979e-05 | 1.1280813800308e-05 | 1.0010410570775e-05 |
| pos\_195 | (E,E)-3,7,11-Trimethyl-2,6,10-dodecatrienyl heptanoate | 4.89671071181358e-05 | 0.000108878453967328 | 9.0170640961469e-05 | 6.83106216193696e-05 | 6.17377871256416e-05 | 6.68315939332568e-05 | 6.16979989755248e-05 | 0.000113942311801838 | 7.76578505192973e-05 | 6.25960347649567e-05 | 7.20834316047353e-05 | 7.42455107914801e-05 |
| pos\_1950 | Ophiopogonin C' | 1.34391661076663e-06 | 1.87164990928872e-06 | 7.91287438017276e-06 | 2.11182366064583e-06 | 2.57169297800504e-06 | 2.95401876770764e-06 | 7.16990381440334e-07 | 2.27029618541872e-06 | 2.25328890778704e-06 | 1.61718482675643e-06 | 2.0862869668866e-06 | 2.33643442665576e-06 |
| pos\_1952 | 4beta-(2-Aminoethylthio)catechin | 0.000139572638926339 | 1.8568234338175e-05 | 2.82907047304351e-05 | 1.18962902095494e-05 | 6.68194710878924e-05 | 5.90429535002912e-05 | 5.29615481034754e-05 | 4.07839377315685e-05 | 3.08347111556299e-05 | 3.61952837833301e-05 | 3.64836325056442e-05 | 3.38777470906418e-05 |
| pos\_1953 | Sulfaphenazole | 0.000326292128761341 | 6.53499891525992e-05 | 7.08791096731433e-05 | 5.89170405246448e-05 | 0.000181763338278337 | 0.000169994069194221 | 0.000158149570939544 | 0.000132644296507808 | 9.45477203652707e-05 | 0.000115040750995332 | 0.00012522003591469 | 0.000113468358878611 |
| pos\_1955 | Hippuric acid | 2.4966517693735e-05 | 2.68406521361577e-05 | 1.90285232426181e-05 | 2.67973110607934e-05 | 3.50483841346585e-05 | 2.37565008721312e-05 | 3.93610556582615e-05 | 2.79297418875909e-05 | 4.00034411734337e-05 | 2.65497178214066e-05 | 3.12066565417249e-05 | 3.07082338711168e-05 |
| pos\_1956 | Bisnorcholic acid | 9.94317950489739e-06 | 3.45069808580186e-06 | 1.58461474081699e-06 | 3.57868071196798e-06 | 9.48616971071794e-07 | 1.90760907589158e-06 | 5.7154499612554e-07 | 2.26461126052198e-06 | 1.30831027332239e-06 | 1.18900590962605e-06 | 0 | 0 |
| pos\_1957 | Corchorusoside A | 5.76712492941287e-05 | 4.60321601165649e-05 | 8.4632016870739e-05 | 0.000140591534988268 | 5.54821051741818e-05 | 4.71609317332337e-05 | 4.14640829783984e-05 | 6.00256081333341e-05 | 6.34252852615175e-05 | 7.38138569884517e-05 | 8.72556155800473e-05 | 6.88655382653114e-05 |
| pos\_1958 | N-Acetylprocainamide | 4.68692683815884e-05 | 4.89340990191025e-05 | 4.74478834890435e-05 | 3.99327338688535e-05 | 3.89990633381878e-05 | 3.99208459308388e-05 | 3.8669204603036e-05 | 3.60973057358352e-05 | 4.18931632688941e-05 | 4.01876870897351e-05 | 3.9535056514044e-05 | 4.04358220637326e-05 |
| pos\_1959 | Cis-8,11,14,17-Eicosatetraenoic acid | 1.31844467005389e-05 | 1.44579466792835e-05 | 1.84318072607794e-05 | 1.45084760990583e-05 | 1.31469509562629e-05 | 1.67515603129401e-05 | 1.30786150468309e-05 | 1.77617498506769e-05 | 1.43584974131885e-05 | 2.08177657397047e-05 | 1.87866978775031e-05 | 1.70342675931505e-05 |
| pos\_196 | Docosahexaenoic Acid methyl ester | 0.00024999995365682 | 0.000309201081500457 | 0.000269513666815374 | 0.000318222996063203 | 0.000256412801942215 | 0.000295561783495157 | 0.000245657819668826 | 0.000257623532015817 | 0.000357741404010935 | 0.000309015758925985 | 0.000305374756491085 | 0.00026606135890497 |
| pos\_1960 | Momordicin I | 0.000240120400989028 | 0.00102639617705921 | 0.00110199314289853 | 0.000775431583084256 | 0.000584796595524747 | 0.000656951828942373 | 0.000625971729194094 | 0.00105429772761441 | 0.000969903228876971 | 0.000785374431105196 | 0.000856199333464628 | 0.00106663387407993 |
| pos\_1961 | 2,5-Dimethyl-1H-pyrrole | 2.24797596234739e-06 | 4.36317794658196e-06 | 3.95043843379779e-06 | 5.14159294019862e-06 | 3.99483339731665e-06 | 4.37050816202117e-06 | 6.68217700144557e-06 | 4.51118574250379e-06 | 4.75525521506014e-06 | 5.54396655533109e-06 | 5.20078918992936e-06 | 3.86148600154103e-06 |
| pos\_1963 | PE(14:0/18:3(6Z,9Z,12Z)) | 7.94818670626784e-06 | 7.92369416589862e-06 | 5.80110019663289e-05 | 1.98496899204645e-05 | 1.40505523995177e-05 | 0.000100521589164162 | 2.16883015786834e-05 | 3.46292581080434e-05 | 5.50550309631967e-05 | 7.05353257418116e-05 | 2.78697592469254e-05 | 2.10829116876056e-05 |
| pos\_1964 | Dextromethorphan | 5.40435980661928e-05 | 7.18141798454803e-05 | 2.57283803551826e-05 | 6.0684847081642e-05 | 1.03069524534048e-05 | 4.03385480581344e-05 | 4.0381410395248e-05 | 3.72163471528587e-05 | 1.35243754433511e-05 | 0.000107106399005673 | 4.47486218854613e-05 | 4.13638901452549e-05 |
| pos\_1965 | Nephritogenoside | 3.1062193514061e-05 | 6.12582282859952e-09 | 0 | 2.95711144139477e-09 | 0 | 0 | 8.81421898296672e-09 | 0 | 0 | 3.85462702654363e-08 | 0 | 7.08070511695076e-06 |
| pos\_1966 | TG(22:4(7Z,10Z,13Z,16Z)/22:4(7Z,10Z,13Z,16Z)/22:6(4Z,7Z,10Z,13Z,16Z,19Z)) | 0.000160669960051709 | 0.000185068270878769 | 0.00019442508528251 | 0.000117408571350816 | 0.00014818366181893 | 0.000157795514695252 | 0.000108892362421367 | 0.000149307247490287 | 0.000132519085279396 | 0.0002617111418666 | 0.000152221629967227 | 0.000178455227003092 |
| pos\_1967 | Camellioside C | 1.11768470231135e-05 | 7.77732118898564e-06 | 7.52276755626137e-06 | 5.65992324056351e-06 | 1.49665784122258e-05 | 1.00116494123963e-05 | 1.51726048254587e-05 | 1.1110281587688e-05 | 8.6932976897136e-06 | 5.35733099320635e-06 | 1.10618727134586e-05 | 4.45178079753873e-06 |
| pos\_1968 | Maduramicin | 0.000257659521947679 | 8.16921237193912e-05 | 7.58465850052795e-05 | 7.10641626073898e-05 | 0.000161856603493227 | 0.000156040385841142 | 0.00015249999499527 | 0.000121616682276594 | 0.000102720491407836 | 0.000122669887664943 | 0.000121483675733468 | 0.000119369448459336 |
| pos\_1969 | Hexyl 2-furoate | 0.000126824722112037 | 0.000149696187069212 | 3.76849144891364e-05 | 0.000113139911411909 | 6.0765211421867e-05 | 7.6894578064875e-05 | 7.60897849360533e-05 | 5.94386310695596e-05 | 1.95738042537935e-05 | 0.000159878335333575 | 6.2149243606464e-05 | 2.85428096267157e-05 |
| pos\_197 | DUARTIN (-) | 6.02319271523333e-05 | 0.000267534939037873 | 5.41108243239207e-05 | 0.000125685115640383 | 0.000222860836899508 | 0.000128858306312428 | 0.000436197207864248 | 0.000285716556646832 | 5.53089183560567e-05 | 5.93755918863959e-05 | 0.00022185249680222 | 7.00584813167757e-05 |
| pos\_1970 | Naloxone | 3.32942300577236e-05 | 4.21931665669188e-05 | 1.15938900851151e-05 | 2.95094124344216e-05 | 2.45788015508688e-05 | 2.7236774048994e-05 | 2.97985594281425e-05 | 2.205605001993e-05 | 1.95906487424953e-05 | 3.10067472819072e-05 | 2.54089479068459e-05 | 1.74909345732565e-05 |
| pos\_1971 | S-Nitrosoglutathione | 8.34574262599038e-05 | 0.000121381605283522 | 6.26243993983978e-05 | 7.78773434813997e-05 | 0.000117756700279125 | 9.19928192602539e-05 | 8.11788946904684e-05 | 7.68448315303553e-05 | 0.000103368762792812 | 5.66805652449267e-05 | 8.18576006175576e-05 | 8.15950979321166e-05 |
| pos\_1972 | Asparaginyl-Glutamine | 4.59256277644604e-05 | 0.000243723776536416 | 0.000102195024711567 | 3.10818231924051e-05 | 0.0001181328762495 | 9.15226523945548e-05 | 7.95949291338333e-05 | 7.57462975348782e-05 | 2.83935323439068e-05 | 0.0002228049621796 | 8.71999750985462e-05 | 6.74488547975612e-05 |
| pos\_1973 | 13-Oxo-9,11-tridecadienoic acid | 0.00019423090985256 | 0.000405423257382851 | 0.000831139553270019 | 0.000122468366594471 | 0.00043766608189136 | 0.000389303816656001 | 0.000104493076956339 | 0.000345185845794915 | 0.000132716548729562 | 0.000886390488592457 | 0.000450961676865976 | 0.000584904287658363 |
| pos\_1975 | Norelgestromin | 0 | 1.78467200938661e-06 | 5.8289113371244e-06 | 5.65289879799164e-06 | 3.43764603952478e-06 | 2.71012028581748e-06 | 1.67561601336712e-07 | 8.33726361260251e-07 | 1.8418914852112e-06 | 2.62889591047413e-06 | 1.25461380345017e-06 | 2.06577718880294e-07 |
| pos\_1977 | Muridienin 1 | 0.000293741593655451 | 0.000414597351461274 | 0.000338250457744441 | 0.000349949951193085 | 0.000259571851265347 | 0.000320762218614795 | 0.00026512134162911 | 0.000323475544354347 | 0.00033353099729771 | 0.000283625263611721 | 0.000311371771480149 | 0.000354848707627092 |
| pos\_1978 | 1,26-Hexacosanediol diferulate | 0.0035722615944212 | 0.00397942285261598 | 0.00378482320285296 | 0.00345341482626284 | 0.00343088662947887 | 0.00355424224728841 | 0.00363698281264001 | 0.00392826858459731 | 0.00370156221355206 | 0.0030327630683213 | 0.00325214721932 | 0.00687931778216734 |
| pos\_1979 | DG(20:2n6/0:0/18:3n3) | 0.000339590887695498 | 0.000355820393801864 | 0.000564856998401551 | 0.000392557414688674 | 0.000410412904265629 | 0.000388971901837306 | 0.000337047227429983 | 0.000355241508389342 | 0.00053811796505235 | 0.000342271655276803 | 0.000372474823672456 | 0.000364148380225852 |
| pos\_198 | DIOSGENIN | 0.000234972751636768 | 0.000218988954192471 | 8.92606256495759e-05 | 0.000298225711916072 | 0.000190560949563502 | 0.000209840471825749 | 0.000309358236378692 | 0.000223311914308416 | 0.00013342972807266 | 0.000580563280131757 | 0.000277067428701396 | 0.000147232936458473 |
| pos\_1980 | 9(S)-HPODE | 3.54846446758948e-05 | 3.68479666342847e-05 | 3.92381557037019e-05 | 3.84886188988517e-05 | 2.95416872968544e-05 | 3.12242674707439e-05 | 2.86635296718168e-05 | 3.42020513823562e-05 | 3.32186447199945e-05 | 2.93267454880865e-05 | 3.27895819802912e-05 | 3.63616225457437e-05 |
| pos\_1981 | N-Nonanoylglycine | 4.58746306146212e-05 | 5.38257729815847e-05 | 5.81808382274859e-05 | 5.67027677966866e-05 | 4.37801097682763e-05 | 5.17208374215431e-05 | 5.0509980159021e-05 | 5.35642257376413e-05 | 5.21519349749362e-05 | 4.80138247789009e-05 | 5.43139061237886e-05 | 5.48546619694256e-05 |
| pos\_1982 | Prostaglandin E2 | 8.28317349717533e-05 | 6.73308691190149e-05 | 5.73595863972568e-05 | 6.77323968423533e-05 | 7.07617929876374e-05 | 6.81607603479972e-05 | 7.11671472991338e-05 | 6.84265529873736e-05 | 7.15918627957332e-05 | 6.73663053307542e-05 | 6.45210381763395e-05 | 6.75669987318749e-05 |
| pos\_1983 | CerP(d18:1/16:0) | 1.77460712299299e-05 | 1.74285249320361e-05 | 1.53333725858858e-05 | 1.50329557652063e-05 | 1.11723136870947e-05 | 2.09860736728627e-05 | 1.86146284266752e-05 | 2.35247667403183e-05 | 1.67971737510355e-05 | 1.16381397186572e-05 | 1.56166361957998e-05 | 1.84484466283848e-05 |
| pos\_1984 | Finasteride | 0.000371604868550072 | 0.000420070603368443 | 0.00032617398284123 | 0.000388138575175155 | 0.00144050857494063 | 0.000404395127362369 | 0.00302141091367934 | 0.00186313222228631 | 0.000439595848441088 | 0.00172895775378487 | 0.000753720471452505 | 0.00101557933062582 |
| pos\_1985 | 3-(4-Hydroxy-2-methoxyphenyl)-2-propenal | 7.8318006045478e-05 | 0.000132155833367526 | 5.0456803707516e-05 | 9.51244207130928e-05 | 9.3130641978998e-05 | 8.70548615049354e-05 | 8.46771954721927e-05 | 7.4028367157881e-05 | 0.000131305028979744 | 8.5063793798152e-05 | 9.26976213103232e-05 | 6.91344597338598e-05 |
| pos\_1986 | Dihydrobiopterin | 8.04756303356965e-05 | 0.000121423669393084 | 4.78793490034611e-05 | 8.56400783427822e-05 | 8.01272266657229e-05 | 7.96035404539519e-05 | 0.000180304233377729 | 0.000110579681340398 | 6.646108857507e-05 | 7.72594029715695e-05 | 0.000104928036588063 | 6.3977195612451e-05 |
| pos\_1988 | 26-Methyl nigranoate | 0.000435891480581534 | 0.000497994266319559 | 0.00102301510896417 | 0.000769517853443241 | 0.000517216481944949 | 0.00064391476790191 | 0.000595781764411558 | 0.000819488268436621 | 0.000555897583957409 | 0.000632145583562107 | 0.000738353292485177 | 0.0012039922531715 |
| pos\_1989 | (E)-2-Octen-1-ol | 8.51892870362725e-06 | 1.75656808251116e-05 | 1.40096490556335e-05 | 1.16342709206156e-05 | 8.06578869760758e-06 | 1.08513883407423e-05 | 9.60465554106687e-06 | 1.32570797906345e-05 | 1.49842192232464e-05 | 8.80808568036865e-06 | 1.03354396262317e-05 | 1.08760718713834e-05 |
| pos\_199 | alpha-curcumene | 0.000355232325585467 | 0.000403701404186682 | 0.000384836903878457 | 0.000386449156010971 | 0.000336769059934051 | 0.00036433881786338 | 0.000393975453489075 | 0.000379045753613406 | 0.000338669906120263 | 0.000414224279858156 | 0.000370888022775937 | 0.000362078002878098 |
| pos\_1990 | LysoPE(0:0/20:1(11Z)) | 0.000471293469783493 | 0.000596924798247757 | 0.000443525286735043 | 0.000568579926339429 | 0.00045271999423763 | 0.000499475897397354 | 0.000583950593397757 | 0.000508797558287925 | 0.000498462122550359 | 0.000483933733842879 | 0.000501954198725392 | 0.000456057390880926 |
| pos\_1992 | PE(15:0/P-18:1(11Z)) | 6.88666101877418e-05 | 7.81077118535546e-05 | 7.30698004919111e-05 | 0.000216287428586647 | 9.93663553477767e-05 | 0.000120240054605747 | 0.000220001074373392 | 0.000143349732664436 | 6.3741362320515e-05 | 8.33969845276074e-05 | 0.000118398396020083 | 6.65331936416426e-05 |
| pos\_1993 | PE(22:4(7Z,10Z,13Z,16Z)/24:0) | 0.00171089183137644 | 0.00190735868538432 | 0.00223181772692591 | 0.00146364134061935 | 0.0010194696577724 | 0.00104586078922201 | 0.00114246102062286 | 0.00118475303260891 | 0.00155718870508103 | 0.00103368150746819 | 0.00109812674191054 | 0.00108759832508539 |
| pos\_1997 | Isohumulone A | 4.86050602222576e-05 | 5.04396257230263e-05 | 4.02568682748194e-05 | 7.84973357639485e-05 | 0.00019413443336799 | 9.14652657538567e-05 | 4.46782749267985e-05 | 8.94580658769003e-05 | 8.37020978703936e-05 | 3.08340877604999e-05 | 7.20162425448855e-05 | 0.000161204994793004 |
| pos\_1998 | DG(20:0/0:0/18:2n6) | 0.000147032126055515 | 6.8438251906686e-05 | 1.80217001211618e-05 | 6.0845689386799e-05 | 6.31460518140218e-05 | 7.39665928458984e-05 | 0.00016216767190321 | 8.77953524493747e-05 | 3.66398572149115e-05 | 4.35000032971882e-05 | 7.70690178998009e-05 | 8.46022406734766e-06 |
| pos\_1999 | N-Despropyl-rotigotine | 1.13302883446215e-06 | 9.72647091982189e-07 | 1.52436126276636e-06 | 1.07369833522837e-06 | 1.01179920859605e-06 | 1.19858386841974e-06 | 1.16409051919916e-05 | 6.1038881716978e-06 | 1.83344726643403e-05 | 7.48235544717182e-06 | 1.13950472584905e-05 | 1.48854435387125e-06 |
| pos\_2 | Betaine | 0.000170517427450722 | 0.000115928081646225 | 9.53278014037365e-05 | 0.000112962988959792 | 5.64667956690802e-05 | 0.000101066774704444 | 7.10969080891213e-05 | 8.02729325549578e-05 | 0.000113535904143407 | 9.45290568703257e-05 | 7.54080686988058e-05 | 0.00123212144890043 |
| pos\_20 | Arachidonic Acid (peroxide free) | 4.71468057745086e-05 | 4.14959096363205e-05 | 3.66888700120988e-05 | 3.43492284790783e-05 | 3.24155842812871e-05 | 3.60235708304138e-05 | 3.96612497992118e-05 | 3.79862932494564e-05 | 3.62471932942054e-05 | 3.71638593499063e-05 | 3.62384394427455e-05 | 3.48306273791625e-05 |
| pos\_200 | 10-propyl-5,9-tridecadien-1-ol | 2.19687459989903e-05 | 3.35944518638194e-05 | 3.06169672388928e-05 | 3.13219285862832e-05 | 2.60075412506689e-05 | 2.53545043479808e-05 | 2.64404894723304e-05 | 2.71950812241594e-05 | 2.38984070326303e-05 | 3.26239773902518e-05 | 2.80217705015906e-05 | 2.47930123657414e-05 |
| pos\_2000 | TG(22:1(13Z)/18:4(6Z,9Z,12Z,15Z)/22:6(4Z,7Z,10Z,13Z,16Z,19Z)) | 0.000112389497782664 | 1.85287041681391e-05 | 1.80554080601689e-05 | 1.25182295956814e-05 | 5.51973963160254e-05 | 5.65383778391407e-05 | 5.17666066482598e-05 | 3.98285543821531e-05 | 2.67854000629036e-05 | 4.11537765768377e-05 | 3.94288700139346e-05 | 3.29461295460752e-05 |
| pos\_2002 | beta-D-Xylopyranosyl-(1->5)-alpha-L-arabinofuranosyl-(1->3)-L-arabinose | 5.88572914300251e-06 | 9.65345511845164e-06 | 1.09602632131702e-05 | 1.13553874850521e-05 | 8.05100724805003e-06 | 9.07859734660202e-06 | 7.53429267546e-06 | 7.98727112068427e-06 | 1.77139027880198e-05 | 6.8626307898701e-06 | 9.16514912779274e-06 | 1.07381192026993e-05 |
| pos\_2003 | N-Acetyldopamine | 0.00016296959752918 | 0.000197966695966826 | 6.38066769727913e-05 | 0.000166247605698433 | 4.25740364695007e-05 | 9.79329505330122e-05 | 0.000132011690143417 | 9.68630625368897e-05 | 4.15481026276397e-05 | 0.000157395315943567 | 0.000108707011952373 | 7.67870190653931e-05 |
| pos\_2004 | N-Alpha-acetyllysine | 9.80379925761199e-05 | 0.000118843400392729 | 0.000150213975642815 | 0.000134967961525862 | 0.000103535615260466 | 0.000121173161142331 | 0.000106242375795874 | 0.000120828178476967 | 0.000117667371176968 | 0.000104795933190213 | 0.000118757765815883 | 0.000126347780384516 |
| pos\_2005 | 3-hydroxyguanfacine glucuronide | 1.79853261467024e-05 | 1.14687065442155e-05 | 8.17478712370294e-06 | 1.00651544091942e-05 | 1.4137620136732e-05 | 1.31808802911245e-05 | 1.51476636743508e-05 | 1.37671747202085e-05 | 1.29921611359838e-05 | 1.43370856273795e-05 | 1.50936375292323e-05 | 2.60932095153272e-05 |
| pos\_2006 | Diosmetin 7-O-beta-D-glucuronopyranoside | 8.46669495162335e-05 | 2.03539328809116e-05 | 2.22558135998886e-05 | 1.68251107283127e-05 | 4.49554937462443e-05 | 4.16105254758126e-05 | 4.16846387029298e-05 | 3.76275325622231e-05 | 2.80395581913384e-05 | 3.07483149747966e-05 | 3.24375980914811e-05 | 3.43797445287112e-05 |
| pos\_2008 | 2,2-dichloro-1,1-ethanediol | 0.000268919041750782 | 0.000346831490100556 | 0.000319336939743101 | 0.000286261257683609 | 0.000243608564894768 | 0.000278905188408369 | 0.00025254395632783 | 0.000292779495437226 | 0.000306247353299408 | 0.000261797853068017 | 0.0002828782799185 | 0.000269821758340766 |
| pos\_201 | 2,3,6-Trimethylphenol | 5.55786135383457e-06 | 5.56498550382963e-06 | 4.73890504647133e-06 | 5.60749175685157e-06 | 4.21040617404656e-06 | 4.60606343365737e-06 | 5.5515098029004e-06 | 4.76642881527884e-06 | 5.96854999336179e-06 | 5.57901801329865e-06 | 5.31019160285895e-06 | 5.26758654784921e-06 |
| pos\_2010 | Epilubimin | 0.000541679199317333 | 0.000419247097605632 | 0.000394047911983409 | 0.000374539498351348 | 0.000389418728579676 | 0.000442149189368709 | 0.000426918180553118 | 0.000437036925292565 | 0.00043489688946689 | 0.000401290860921256 | 0.000417697040167076 | 0.000379841644375872 |
| pos\_2012 | Asparaginyl-Cysteine | 2.18348369015636e-05 | 2.82914752242825e-05 | 1.79124528812386e-05 | 2.34191801044172e-05 | 2.03155648183486e-05 | 2.02719414407742e-05 | 2.0653049765368e-05 | 2.11599750297218e-05 | 2.16854831851587e-05 | 2.6620823992214e-05 | 2.08802171405152e-05 | 1.90452430998723e-05 |
| pos\_2013 | Kelampayoside A | 6.39035119168284e-06 | 8.59872409278215e-06 | 2.37412745311231e-06 | 5.07760892825094e-06 | 7.89658507084284e-06 | 4.05022831301072e-06 | 5.24190002347173e-06 | 4.45101994501532e-06 | 3.5773767035611e-06 | 7.64478323793688e-06 | 4.82034591219458e-06 | 7.23756866527483e-06 |
| pos\_2014 | 1-(2-Thienyl)-1,2-propanedione | 0.000291849199717918 | 0.000258606955271391 | 0.000208693507549904 | 0.000198577542381496 | 0.00020620533789976 | 0.000203176221427571 | 0.000215188356570343 | 0.000220772644292325 | 0.000228531541133736 | 0.000178022921120138 | 0.000231244033845576 | 0.000232183916633334 |
| pos\_2015 | N-Ribosylhistidine | 4.91114730283706e-05 | 4.51452982066404e-05 | 1.07737639352737e-05 | 2.12117225549859e-05 | 2.23797182025225e-05 | 1.99284074900758e-05 | 1.66521540991801e-05 | 1.43832034891102e-05 | 1.29443929521663e-05 | 2.66464403160112e-05 | 1.75032727591434e-05 | 1.30092137015368e-05 |
| pos\_2019 | Chinenoside III | 8.76744024115203e-06 | 5.36741983093276e-06 | 7.25769745671347e-06 | 9.0493885152399e-06 | 1.05179977265898e-05 | 9.11435368314526e-06 | 1.00342081385612e-05 | 9.55907762750476e-06 | 1.2820533706625e-05 | 9.75873066673732e-06 | 7.90186982379496e-06 | 9.50705849626112e-06 |
| pos\_202 | Farnesyl Thiosalicylic Acid | 0.00265326099739824 | 0.00167297401494058 | 0.00162502085943215 | 0.00139781050747059 | 0.00162748348332676 | 0.00170472176115386 | 0.00172208101775902 | 0.00164520087900293 | 0.0017162752350363 | 0.00155223564530884 | 0.00164279425997184 | 0.0016050780202879 |
| pos\_2020 | Nandrolone | 8.99333631175617e-06 | 1.09469117885121e-05 | 7.40050568303012e-06 | 9.13135949171549e-06 | 8.20711397229372e-06 | 9.72511776529945e-06 | 9.90451338869441e-06 | 1.33888440936179e-05 | 1.17018850832509e-05 | 9.19242371986833e-06 | 1.30220260206242e-05 | 1.94169826510094e-05 |
| pos\_2021 | Eremopetasinorone A | 4.34161190928075e-05 | 5.47181895588226e-05 | 5.24246361755126e-05 | 4.92968916089671e-05 | 4.17671433625581e-05 | 4.61132558849868e-05 | 4.64228354463699e-05 | 4.95304231647015e-05 | 5.08218911186655e-05 | 4.71005948224416e-05 | 4.73706649936638e-05 | 4.77262430302415e-05 |
| pos\_2022 | Isofucosterol 3-O-[6-O-Hexadecanoyl-b-D-glucopyranoside] | 0.00180442444007625 | 0.00202951134712369 | 0.00294549268407945 | 0.00254259358724995 | 0.00184897112778053 | 0.00201534437896279 | 0.00179338758468162 | 0.00232289301253754 | 0.00250408681843243 | 0.0017919382779541 | 0.00216477105456873 | 0.00201741939016416 |
| pos\_2023 | norerythromycin | 3.61201114078646e-05 | 5.29181884175766e-05 | 4.52373587616484e-05 | 4.66979306389947e-05 | 4.40582716428176e-05 | 5.06316084517153e-05 | 4.21237712110783e-05 | 4.73606702716469e-05 | 5.8758320761467e-05 | 4.94212275309775e-05 | 5.1148084112559e-05 | 4.17570489451419e-05 |
| pos\_2025 | Nb-p-Coumaroyltryptamine | 1.12427478181628e-05 | 1.37123984691986e-05 | 2.50639890330059e-05 | 8.78672976298007e-06 | 1.1034877348056e-05 | 1.01415950170239e-05 | 2.72989025993846e-05 | 3.21154285119862e-05 | 6.7879092587032e-06 | 2.78406424707356e-05 | 2.23259017457921e-05 | 2.73293814279885e-05 |
| pos\_2026 | 7Z,10Z-Hexadecadienoic acid | 9.89998908517696e-05 | 0.00011016438619717 | 0.000105588484289235 | 0.000123421048849353 | 9.05129315340311e-05 | 0.000104730769771689 | 0.000103437939326113 | 0.000110064472427242 | 0.000115205408501083 | 0.000114818638980737 | 0.00011450129384121 | 0.000100771868256316 |
| pos\_2027 | (3b,6a,12b,17a,20S)-Dammar-24-ene-3,6,12,17,20-pentol 20-[glucosyl-(1->2)-[rhamnosyl-(1->6)]-glucoside] 6-xyloside | 7.35298566895993e-06 | 6.17683724826478e-06 | 1.16113123400317e-05 | 7.98635256699231e-06 | 9.57506131970197e-06 | 7.8984520057108e-06 | 1.62495361449432e-05 | 1.47308210407475e-05 | 7.86520135364678e-06 | 1.50682523960723e-05 | 1.24238178014197e-05 | 1.38339070780034e-05 |
| pos\_2028 | Dihydrozeatin | 7.698929085696e-05 | 4.78856893434318e-05 | 5.37889452665826e-05 | 5.21192178731915e-05 | 6.28384158689512e-05 | 5.90986862356818e-05 | 5.83327114803625e-05 | 5.44330468445111e-05 | 6.02326075396744e-05 | 5.52039263449388e-05 | 5.58173815124941e-05 | 5.71272349803784e-05 |
| pos\_2029 | Cembrene | 3.57674774499296e-05 | 3.69463926262844e-05 | 4.23249578612458e-05 | 4.20222368099731e-05 | 4.8177448648331e-05 | 4.00560604558832e-05 | 4.22637121298011e-05 | 3.99616016054126e-05 | 4.51737575494488e-05 | 3.87318510857388e-05 | 3.864597367964e-05 | 6.38358358941228e-05 |
| pos\_203 | Malvidin | 0.000132741818793857 | 6.37044631350504e-05 | 7.66613641801237e-05 | 6.00222196820476e-05 | 4.92931656244339e-05 | 5.62503496940162e-05 | 0.000335233952144765 | 9.543427127498e-05 | 3.72743621910761e-05 | 0.000177193460466096 | 8.00136734769641e-05 | 6.94707569540564e-05 |
| pos\_2030 | CerP(d18:0/16:0) | 3.6940464268118e-06 | 7.7612101979924e-07 | 0 | 0 | 5.09355002547179e-07 | 1.30228077721326e-06 | 1.77876742797446e-06 | 4.75740746386008e-07 | 4.58614839007376e-07 | 7.83640937953249e-07 | 1.16349348193964e-06 | 0 |
| pos\_2031 | 6-Acetylmorphine | 0.000106264346943104 | 0.0001174824554002 | 4.26689760013213e-05 | 7.95102189955814e-05 | 8.05772522721403e-05 | 7.21241797374659e-05 | 6.30273799492246e-05 | 5.94163228738673e-05 | 6.57244928500783e-05 | 7.35889184662508e-05 | 6.80924138130068e-05 | 6.49889283316225e-05 |
| pos\_2033 | Lucidumol A | 0.000115847403424353 | 0.000109134600868127 | 0.000108157959118135 | 0.000132144571874321 | 0.000108149654755634 | 0.000145234072025505 | 0.000137820300895932 | 0.000770174024692494 | 0.000142133580775192 | 0.000148876776852135 | 0.000540544518413469 | 0.00185391566433255 |
| pos\_2034 | 6-Hydroxy-R-acenocoumarol | 4.31225122261622e-06 | 1.55171403404236e-05 | 5.51083825140688e-06 | 7.82334412033692e-06 | 1.0079573571492e-05 | 8.55234946141712e-06 | 9.07042255037923e-06 | 1.2713715165873e-05 | 1.45872558571549e-05 | 7.03542977463081e-06 | 8.84885354383058e-06 | 5.81923037401051e-06 |
| pos\_2035 | PE(14:1(9Z)/22:2(13Z,16Z)) | 1.39338403828139e-05 | 1.16257048335995e-05 | 2.93460977075323e-06 | 1.03939206542284e-06 | 2.22061280909913e-06 | 6.77159008342976e-06 | 2.17652067792621e-06 | 2.4675717370515e-06 | 4.13735732748053e-06 | 0 | 3.75881808475347e-06 | 2.11117291610482e-06 |
| pos\_2037 | Ganglioside GA2 (d18:1/22:0) | 5.1331392180072e-05 | 3.81300684663967e-05 | 2.977730896402e-05 | 3.35889575958354e-05 | 3.92618508627554e-05 | 3.35354411226268e-05 | 4.24435571669917e-05 | 3.97751377256882e-05 | 3.90837182053049e-05 | 3.61412618504793e-05 | 3.84439154480777e-05 | 3.15130229747966e-05 |
| pos\_2039 | Citronellyl anthranilate | 7.97340477300967e-05 | 0.000105453126584152 | 9.39269409072598e-05 | 9.02661519314715e-05 | 7.55574524602759e-05 | 9.05623837297313e-05 | 8.23548200761938e-05 | 8.98624630405508e-05 | 9.71878006570678e-05 | 8.56880187939669e-05 | 9.09025786391684e-05 | 8.48638134631604e-05 |
| pos\_204 | Aminoparathion | 4.88814027300275e-06 | 7.17106581873897e-06 | 8.4711544086286e-06 | 7.07246075240107e-06 | 4.45379549079146e-06 | 6.11216363504351e-06 | 7.1532629548838e-06 | 5.44862422169655e-06 | 9.26397334352415e-06 | 5.17058994298267e-06 | 7.41286944549562e-06 | 5.47208978366004e-06 |
| pos\_2040 | Azaspiracid 2 | 9.54403357247265e-06 | 1.49673645712833e-05 | 3.10455568448245e-05 | 5.49354276204325e-05 | 3.16793924945222e-05 | 3.11173835675199e-05 | 2.6993613469308e-05 | 2.30355267352557e-05 | 1.99424056740351e-05 | 1.10993758036279e-05 | 2.23808422355799e-05 | 2.31141045076237e-05 |
| pos\_2041 | 2-(10-Heptadecenyl)-6-hydroxybenzoic acid | 1.29182487928578e-05 | 1.38808377014861e-05 | 2.6384561332712e-05 | 3.16890253184461e-05 | 7.46342933079883e-05 | 3.5589339080957e-05 | 3.61940691368961e-05 | 2.11100783508666e-05 | 4.71276176685522e-05 | 2.27073952772262e-05 | 3.62034433603319e-05 | 2.99300964234898e-05 |
| pos\_2042 | 1,4-Benzothiazine-O-quinonimine | 0.000161432469347719 | 2.83157723003242e-05 | 3.29244999085706e-05 | 1.68679118945556e-05 | 8.89290756526726e-05 | 8.88733330769181e-05 | 7.18389798997861e-05 | 6.63508864297722e-05 | 4.55474487445532e-05 | 4.84126659042938e-05 | 5.76153162720302e-05 | 5.02162290904049e-05 |
| pos\_2043 | Jaceidin | 2.94677797916273e-05 | 5.4877275974653e-05 | 3.83883664658722e-05 | 3.41930636400911e-05 | 4.41965750510115e-05 | 3.805477354984e-05 | 4.57199732479837e-05 | 3.74109398154354e-05 | 5.47895594911465e-05 | 4.44055095586418e-05 | 4.14181378569132e-05 | 3.66550929509356e-05 |
| pos\_2044 | desmethylclomipramine | 7.91655807417034e-05 | 0.00010392777132189 | 6.55287656327855e-05 | 7.47988306432603e-05 | 7.7533173740744e-05 | 7.54067643924254e-05 | 8.46012813729238e-05 | 7.9237581430478e-05 | 7.82537555941882e-05 | 8.64769365507294e-05 | 7.83322389250605e-05 | 6.60244893239553e-05 |
| pos\_2047 | 8,8-Dimethoxy-2,6-dimethyl-2-octanol | 0.000225198300232017 | 0.000380010157814781 | 0.000186605097658689 | 0.000333480473537675 | 0.000190990667829687 | 0.000269625943243767 | 0.0003506063931035 | 0.000271781361093582 | 0.00025230368686927 | 0.000337664803880301 | 0.000288689126997984 | 0.000202463229688723 |
| pos\_2048 | Phenylacetaldehyde | 0.000397284408812373 | 0.000296974801030976 | 0.000281220794344958 | 0.000242889971500627 | 0.00026320846443042 | 0.000287068102260913 | 0.000276731456151558 | 0.000270188173243888 | 0.000294305713653249 | 0.000262252103396705 | 0.000275627565678736 | 0.000269548637580357 |
| pos\_2049 | 2,4-Nonadien-1-ol | 2.11885140027162e-05 | 2.6316182217529e-05 | 2.40657983440365e-05 | 2.13692980130486e-05 | 1.95090096056671e-05 | 2.13411198498165e-05 | 1.98899478616326e-05 | 2.06595358425979e-05 | 2.2599837319483e-05 | 2.05256314416993e-05 | 2.16247231698364e-05 | 1.80628286336075e-05 |
| pos\_205 | 4-Carboxy-2-hydroxy-6-methoxy-6-oxohexa-2,4-dienoate | 4.07838549573931e-05 | 5.70995766343621e-05 | 5.66509463763112e-05 | 4.62428754430512e-05 | 3.67423233085705e-05 | 4.93020260342162e-05 | 4.35541617594663e-05 | 4.71383690722155e-05 | 5.49489441965588e-05 | 4.9807740027999e-05 | 4.71872041474355e-05 | 4.43948190904659e-05 |
| pos\_2050 | AK toxin I | 0.00013853307940882 | 0.000150231997529532 | 0.000130281533804126 | 0.000123388847661244 | 0.000126467407050402 | 0.000131657617794124 | 0.000141074219523407 | 0.000128283655644584 | 0.000138996657010707 | 0.000112635445469937 | 0.000127558234431521 | 0.00014307385325329 |
| pos\_2051 | 1-Methyl-1,3-cyclohexadiene | 2.38691887353645e-05 | 2.06580872097786e-05 | 4.90487397365785e-05 | 3.89824613644036e-05 | 5.18249869378908e-05 | 3.59406201721373e-05 | 2.07415003392262e-05 | 2.44672763091278e-05 | 4.09940378557116e-05 | 3.88581736681065e-05 | 2.74743057131991e-05 | 3.09371385440592e-05 |
| pos\_2053 | Parasiloxanthin | 0.000125842763776815 | 0.000155102975094845 | 5.14018426090346e-05 | 0.00017044076830477 | 1.98461173354039e-05 | 8.68629394892293e-05 | 8.71065998056687e-05 | 9.77809377215222e-05 | 0.000136589936603857 | 7.48678000862394e-05 | 8.58010851066642e-05 | 8.68391294533354e-05 |
| pos\_2054 | Desmosterol | 1.58872511838223e-05 | 2.01356268784028e-05 | 1.665095543616e-05 | 2.07718951407053e-05 | 2.84304592780695e-05 | 2.07800476683805e-05 | 1.17407275173918e-05 | 1.52667110676966e-05 | 3.04043409679179e-05 | 2.05800080212985e-05 | 2.03895967291763e-05 | 2.00676222330586e-05 |
| pos\_2056 | Capsicum annuum Fluorescent chlorophyll catabolite | 7.80424336271419e-06 | 1.27954428919737e-05 | 1.13939545382865e-05 | 7.27798909392904e-06 | 1.31584179373823e-05 | 1.14045037706128e-05 | 2.17823262436596e-05 | 1.70851811660206e-05 | 7.35375337549712e-06 | 1.93449557797614e-05 | 1.25477724553615e-05 | 9.44095071385321e-06 |
| pos\_2057 | N5-Acetyl-N2-gamma-L-glutamyl-L-ornithine | 2.87823582460224e-05 | 3.57418213349484e-05 | 3.27700821866356e-05 | 2.45607539677717e-05 | 3.27381035799066e-05 | 3.92927062334747e-05 | 4.82122720394676e-05 | 4.09024054913717e-05 | 3.3445086941365e-05 | 4.81189069811282e-05 | 3.85696711271216e-05 | 4.33176542479318e-05 |
| pos\_2058 | Methionyl-Proline | 6.13502762755767e-06 | 1.03698644343842e-05 | 3.17663002212186e-05 | 0.000130369988439492 | 9.14467314717261e-06 | 2.38548410473443e-05 | 1.33027578644254e-05 | 3.45302126524545e-05 | 7.63195306970928e-06 | 9.32683117702339e-06 | 2.65200116448379e-05 | 7.54004411512696e-05 |
| pos\_2059 | 4a-Formyl-5a-cholesta-8,24-dien-3b-ol | 2.57731068987857e-06 | 2.78524265284487e-06 | 5.57386351726445e-06 | 7.40160479470214e-06 | 1.184641902091e-05 | 6.57417410211257e-06 | 1.08334980446796e-05 | 8.21332425476394e-06 | 1.21545553951168e-05 | 1.05626765291654e-05 | 8.18403126048027e-06 | 7.70166333558292e-06 |
| pos\_206 | GSK 4716 | 1.53088089763209e-05 | 2.01126442539715e-05 | 1.50146598248848e-05 | 1.63681765660949e-05 | 1.36808780465253e-05 | 1.52505063843722e-05 | 1.12513461049729e-05 | 1.20401288106539e-05 | 1.93360793035363e-05 | 1.88845605139785e-05 | 1.75571017164282e-05 | 1.60843948423782e-05 |
| pos\_2060 | 2,6-Dimethyl-2,4-heptadiene | 2.31046116842942e-05 | 2.34172863153003e-05 | 3.50024732611579e-05 | 3.82879662435131e-05 | 1.56993869886535e-05 | 2.82058371206543e-05 | 2.04455453939754e-05 | 4.6333181031366e-05 | 1.82419782491323e-05 | 3.29442041948352e-05 | 3.89286479147797e-05 | 8.90331402991113e-05 |
| pos\_2061 | Oenanthoside A | 8.52101709964942e-06 | 3.52903190961401e-06 | 3.12989876730909e-06 | 3.29390806955415e-06 | 5.36459213527061e-06 | 4.8954842773328e-06 | 5.64731299943768e-06 | 4.73981471466243e-06 | 5.53063112375708e-06 | 4.71678283760405e-06 | 5.53637905439581e-06 | 1.86938462891261e-05 |
| pos\_2062 | Piperidine | 0.000208914445129823 | 0.00027999254941845 | 0.000165443873395752 | 0.000244129116077516 | 0.000213486698475702 | 0.000176222433730455 | 0.000217109893490721 | 0.000220684064722754 | 0.000190230182390784 | 0.000215364169917975 | 0.000228374380954502 | 0.000233266120782616 |
| pos\_2063 | 16-Oxoestrone | 9.04293940608713e-05 | 4.76778048249175e-05 | 2.72716744445753e-05 | 3.20372044479293e-05 | 5.99936587291881e-05 | 4.40402583685099e-05 | 5.27885872311615e-05 | 4.22735872962839e-05 | 3.92975816984424e-05 | 3.34282779351259e-05 | 4.24820106509573e-05 | 5.16764361328121e-05 |
| pos\_2064 | Cheritamine | 0.000325094645068699 | 8.14575655902955e-05 | 9.69086872261136e-05 | 7.46677792180597e-05 | 0.000197548612218349 | 0.000196019096096523 | 0.000175631668759791 | 0.000155213661390904 | 0.000118957174382697 | 0.000127895482627963 | 0.000155769134943931 | 0.000133420552778767 |
| pos\_2066 | Isoaustin | 0.00012882635240798 | 0.000176840017930738 | 0.000155522921664014 | 0.00016003549884384 | 0.000139377907118753 | 0.000127823178434266 | 0.000121778131155248 | 0.000150627288778806 | 0.000144057215842846 | 0.000121333004892504 | 0.000151913835693398 | 0.000148540967587957 |
| pos\_2067 | PGP(18:0/20:3(5Z,8Z,11Z)) | 0.000195328718777127 | 5.57815675812421e-05 | 5.04892528297315e-05 | 4.74648604578498e-05 | 0.000103717032218637 | 9.81734480328727e-05 | 0.000101516604298326 | 7.91997180205692e-05 | 6.5648624809543e-05 | 7.77572423184727e-05 | 7.63949364082635e-05 | 7.52522699589423e-05 |
| pos\_2068 | DG(14:0/0:0/20:2n6) | 0.000570297122305866 | 0.000661662291972231 | 0.000481249261239871 | 0.00065023434113348 | 0.000503641109316395 | 0.000599192586625934 | 0.000623443684981751 | 0.00058921710024394 | 0.000686030739827609 | 0.000611786087812385 | 0.000574216412993489 | 0.000553065760994178 |
| pos\_2069 | 2-Acetyl-1,5,6,7-tetrahydro-6-hydroxy-7-(hydroxymethyl)-4H-azepine-4-one | 9.48058033049042e-05 | 0.000122722307554705 | 0.000127762898675956 | 9.9428781695928e-05 | 9.01023178153144e-05 | 0.000102679409568202 | 8.21501751593334e-05 | 0.000101365657349552 | 0.000106726019272076 | 0.000114477017098436 | 0.000102206356111345 | 0.000109803845301653 |
| pos\_207 | Val Ser Val | 0.000674884042701057 | 0.000279154783470925 | 7.79580758511863e-05 | 0.00033998953725746 | 2.81365189586508e-05 | 0.000263932387195746 | 4.95682308267777e-05 | 6.50177745536865e-05 | 0.000133626296305598 | 0.000872912803806677 | 0.000223510726771953 | 0.000100543130898994 |
| pos\_2072 | 2-Hydroxyestradiol | 0.000134245745661704 | 7.60561974607633e-05 | 6.6133508659902e-05 | 6.18404364509905e-05 | 6.97901332218781e-05 | 8.1865584220027e-05 | 8.44759326122005e-05 | 7.85891970573894e-05 | 7.47131148330781e-05 | 7.13422152434155e-05 | 7.54112213630034e-05 | 7.40018224549368e-05 |
| pos\_2073 | Tuberoside J | 0.000133156917835779 | 3.4148078698522e-05 | 3.68929876400151e-05 | 2.57640380346644e-05 | 8.10178868922871e-05 | 7.54680988695566e-05 | 7.81119724274646e-05 | 5.96706539902742e-05 | 4.21353730301057e-05 | 5.35881518392386e-05 | 5.5908031227056e-05 | 5.88412550178107e-05 |
| pos\_2074 | Tauro-b-muricholic acid | 0.00447839821329078 | 0.00176025735604895 | 0.00160858386620262 | 0.00146257785167283 | 0.00294091895242368 | 0.00299476157360884 | 0.00314949314071176 | 0.00238274884919241 | 0.00220014767724385 | 0.00234971772265052 | 0.00243316827907929 | 0.00244179416773595 |
| pos\_2076 | Elenolide | 0.000121020183792292 | 0.000223226511187943 | 6.10482937977259e-05 | 0.000138958722037064 | 0.000149085239837694 | 0.000132709981002531 | 8.51003017696947e-05 | 7.77104343663455e-05 | 0.000216950463972787 | 0.000119402893073898 | 0.0001213126448898 | 8.00885619959643e-05 |
| pos\_2077 | Ibuprofen | 5.87946890280925e-05 | 9.08422613027284e-05 | 5.97117916237096e-05 | 5.55776267880626e-05 | 7.07031815010233e-05 | 6.31540066553434e-05 | 5.83917959135645e-05 | 5.66227096973636e-05 | 6.31387169016645e-05 | 6.5778995355934e-05 | 6.13187106790071e-05 | 7.43147113191972e-05 |
| pos\_208 | D-4-Hydroxyphenylglycine | 1.82555845445908e-05 | 3.14026448937401e-05 | 1.27165316040064e-05 | 5.18372667026291e-05 | 2.90784323451311e-06 | 1.96655040199851e-05 | 5.82154544414988e-05 | 3.74410814806621e-05 | 3.42303438986385e-06 | 9.01848962553237e-05 | 4.3180108291155e-05 | 1.00197728028241e-05 |
| pos\_2080 | Isocarlinoside | 1.16137254644214e-05 | 8.24498421020697e-06 | 1.17938967478694e-05 | 8.05374208524591e-06 | 1.40077661918889e-05 | 1.51361173698308e-05 | 1.09240717481718e-05 | 1.63138415912139e-05 | 1.3576206544193e-05 | 1.26543065678758e-05 | 1.09953646552576e-05 | 4.0619955325501e-05 |
| pos\_2081 | Cassiaside | 7.79816105664718e-06 | 1.63022262815155e-06 | 5.43334443146548e-07 | 0 | 7.8184420400296e-07 | 1.93379870347001e-06 | 0 | 1.83155497540125e-06 | 3.35957625695489e-06 | 0 | 8.54722230918027e-07 | 0 |
| pos\_2084 | Pipereicosalidine | 2.73328300357346e-05 | 2.86999412042469e-05 | 5.64788027516685e-05 | 3.76402034117769e-05 | 7.03715523879728e-05 | 4.27591138857102e-05 | 2.43054190094137e-05 | 5.08178570262411e-05 | 2.80915466767363e-05 | 2.36027507771683e-05 | 3.48615308262499e-05 | 5.5677215440422e-05 |
| pos\_2085 | Asitrilobin D | 6.98442742439916e-05 | 0 | 5.73522591753329e-06 | 1.75335840562104e-05 | 1.32518776273092e-05 | 2.31816019083387e-05 | 2.423688099674e-05 | 2.29050301059679e-05 | 1.41167656295678e-06 | 2.24436983338052e-06 | 1.24649582176416e-05 | 2.18146552392484e-05 |
| pos\_2086 | Licoagroside B | 9.59286142914417e-06 | 1.24785758493866e-05 | 1.12171926005907e-05 | 1.34253822089282e-05 | 8.65106563304702e-06 | 1.52454207615558e-05 | 1.13206939153372e-05 | 8.95794395130438e-06 | 7.6273156790499e-06 | 2.49423988761962e-05 | 1.29625024256256e-05 | 4.98662555520474e-06 |
| pos\_2087 | DIBOA-Glc | 5.24402334132051e-05 | 5.40461750009983e-05 | 4.95686371821785e-05 | 4.88769649891859e-05 | 5.20464522181483e-05 | 6.03318054603873e-05 | 5.26470769095209e-05 | 4.86069313400816e-05 | 6.35027024469175e-05 | 4.67196269133705e-05 | 5.51493010096065e-05 | 6.06261031921816e-05 |
| pos\_2088 | Hovenidulcigenin B | 0.00700249072092642 | 0.00318345192103108 | 0.00300133530402913 | 0.00266023240105667 | 0.00476117699643124 | 0.00479606313951926 | 0.00512799657231442 | 0.00405992758421598 | 0.00383783730602743 | 0.00377944750027391 | 0.00399567708286313 | 0.0041620430434135 |
| pos\_209 | Phenylacetylglycine methyl ester | 4.24848186757095e-05 | 3.42965093755649e-05 | 1.30267674280399e-05 | 3.20237564701112e-05 | 6.46168496785239e-05 | 2.80574646778215e-05 | 1.74839657278742e-05 | 1.66398600181735e-05 | 1.82876874848054e-05 | 2.62053820408948e-05 | 1.98480412549847e-05 | 2.09139656740084e-05 |
| pos\_2093 | Adrenoyl ethanolamide | 0.00131060892496729 | 0.00189648858357202 | 0.00217268504703627 | 0.00202014353332275 | 0.00143463861738714 | 0.00171928356890993 | 0.00142499970112095 | 0.00180756736793717 | 0.00152537652968184 | 0.00148346704073128 | 0.00168568593315237 | 0.00209471667173539 |
| pos\_2096 | Austdiol | 9.21395672942932e-07 | 2.998157744994e-06 | 1.82092105065276e-06 | 4.17694637874683e-06 | 2.04977664066002e-06 | 1.57860687870669e-06 | 1.47739318552262e-06 | 1.58816125540222e-06 | 1.63178592694684e-06 | 1.82567862619846e-06 | 1.6969085873197e-06 | 1.02462683596868e-06 |
| pos\_2097 | 10-Hydroxymelleolide | 0.000148122095930064 | 0.000128781773641097 | 0.000126872556600745 | 0.000103851311511718 | 0.000146264613863212 | 0.000123211619943635 | 0.000134919813115035 | 0.000129827274331743 | 0.000110512221473231 | 0.000135446093955965 | 0.000111571165731073 | 0.000147751504521486 |
| pos\_2098 | 2-Methoxyxanthone | 3.11393491521915e-05 | 4.91511888595943e-05 | 3.03461520689915e-05 | 3.96149708211203e-05 | 4.34204384427858e-05 | 4.03653817542772e-05 | 3.46225661559125e-05 | 3.49569970295321e-05 | 4.46435538801529e-05 | 3.06967068993256e-05 | 3.42856092364558e-05 | 2.53404147050936e-05 |
| pos\_2099 | 6-Acetyl-2,3-dihydro-2-(hydroxymethyl)-4(1H)-pyridinone | 3.31079892789512e-05 | 1.50195455446742e-05 | 4.8963479122039e-05 | 2.1319167512021e-05 | 2.7438307275641e-05 | 2.680256030968e-05 | 2.35702494808294e-05 | 2.42488300262176e-05 | 2.16609322035162e-05 | 2.05745716154567e-05 | 2.21368152552604e-05 | 3.33817733285296e-05 |
| pos\_21 | Acetoin | 0.00105411749482465 | 0.000597895813806859 | 0.000568840091111107 | 0.000513948243827696 | 0.000626212319569453 | 0.000644055189225656 | 0.000688256972240652 | 0.00063951556347853 | 0.000632422065748278 | 0.000586436785228363 | 0.000613857899992435 | 0.000626751668517456 |
| pos\_210 | Mintsulfide | 8.02208348794139e-05 | 0.000102993997755757 | 0.000102107087400342 | 8.67029786248027e-05 | 7.24482362329555e-05 | 8.61201795235919e-05 | 8.00315679587757e-05 | 9.20796513253512e-05 | 8.44789359277244e-05 | 9.14889226568088e-05 | 8.84931826605737e-05 | 8.71845979372555e-05 |
| pos\_2104 | Galabiosylceramide (d18:1/9Z-18:1) | 0.000380364699509173 | 0.000147629072356375 | 0.000146337508012642 | 0.000124630132984311 | 0.000130343750809167 | 0.000122689282348747 | 0.000138132260975826 | 0.000120704903405166 | 0.000119376739041164 | 0.000113894080404048 | 0.000120965108953552 | 0.000126627287157454 |
| pos\_2105 | Glycine | 5.12441255337323e-06 | 6.93910509788228e-06 | 9.38376228619089e-06 | 8.1067883665382e-06 | 5.93501507161674e-06 | 6.91197187156719e-06 | 5.17959322960541e-06 | 7.2790473883206e-06 | 5.96266345633417e-06 | 5.56525618751044e-06 | 6.66375880232517e-06 | 9.62231548102716e-06 |
| pos\_2107 | hydroxyisovaleroyl carnitine | 1.5684807639826e-06 | 3.50562223760737e-06 | 4.72855189930164e-06 | 3.48467233436344e-06 | 2.21029941940599e-06 | 2.37015746390358e-06 | 3.45428516558793e-06 | 3.71596887746404e-06 | 4.41526373792849e-06 | 2.84386517101532e-06 | 3.13408924028431e-06 | 4.34528466246455e-06 |
| pos\_2108 | PC(20:4(8Z,11Z,14Z,17Z)/22:2(13Z,16Z)) | 5.44518873708224e-06 | 1.15955393298556e-05 | 9.04953436478195e-06 | 8.12693445572003e-06 | 6.55707528513703e-06 | 8.29998377762797e-06 | 7.74569246973399e-06 | 6.70749892183246e-06 | 8.42049199712159e-06 | 7.55160465014856e-06 | 8.76544143875036e-06 | 8.45149230091401e-06 |
| pos\_2109 | N-[(3a,5b,7a,12a)-3,7-dihydroxy-24-oxo-12-(sulfooxy)cholan-24-yl]-Glycine | 7.47246522198099e-05 | 6.49697814431792e-05 | 3.42877600443982e-05 | 5.61936965044454e-05 | 4.61371916538487e-05 | 5.65572105041583e-05 | 6.54662541103393e-05 | 5.06858351595253e-05 | 4.18946577298835e-05 | 5.35803155576324e-05 | 5.637652079292e-05 | 4.4962457825152e-05 |
| pos\_211 | Lys Val Gly | 0.000184952607629836 | 0.00017676137513409 | 9.8154681575477e-05 | 8.55930627432335e-05 | 0.000220704459728434 | 0.000147738229696091 | 0.000100908303658983 | 0.000113563498168652 | 9.99360924884693e-05 | 9.0474559079514e-05 | 0.000109792829344319 | 0.000139055963137303 |
| pos\_2112 | Oxymorphone | 1.32740010993957e-05 | 1.11653423417789e-05 | 3.25451690410867e-06 | 4.73029417582897e-06 | 1.6066000712101e-05 | 3.70048373195878e-05 | 8.53223733684147e-06 | 5.69912671729702e-06 | 4.14195819793085e-06 | 7.56416854769425e-06 | 3.1523010057473e-06 | 3.5136763396562e-06 |
| pos\_2113 | PS(DiMe(11,5)/DiMe(9,3)) | 1.64718310161201e-05 | 3.28098120190852e-05 | 2.64239461909456e-05 | 2.66499392184623e-05 | 1.86158769154419e-05 | 2.42902230102253e-05 | 2.19554171472691e-05 | 1.98134108662871e-05 | 2.83763957920808e-05 | 2.37450286260539e-05 | 2.43425287596017e-05 | 2.32488524367236e-05 |
| pos\_2116 | Garcimangosone D | 1.52697762238003e-06 | 2.56135126787903e-06 | 1.09924514507118e-05 | 7.66128109101983e-06 | 1.80169659742993e-06 | 6.44846934652694e-06 | 7.37196863795522e-06 | 8.33411816722981e-06 | 5.17914871013811e-06 | 1.98289427085232e-06 | 1.03433216558079e-05 | 1.18353330205944e-05 |
| pos\_2117 | 2-(4-Allyl-2-methoxyphenoxy)-1-(4-hydroxy-3-methoxyphenyl)-1-propanol | 6.05239955491905e-05 | 8.91779837561626e-05 | 0.000123778381404438 | 7.69121393779189e-05 | 4.67931667680224e-05 | 7.12457531060092e-05 | 7.03882146516678e-05 | 7.42871480959632e-05 | 0.000187457125119579 | 9.87527117008009e-05 | 0.000105237882911601 | 8.70968285166266e-05 |
| pos\_2118 | Serinyl-Tyrosine | 0.000129564708395062 | 0.000159543226897587 | 3.71475730933078e-05 | 6.22339612084608e-05 | 0.000111570961616848 | 9.34201905791098e-05 | 5.30825017477615e-05 | 4.62525466598457e-05 | 8.15585182463953e-05 | 7.30283286744815e-05 | 5.73559206247615e-05 | 3.98695540116375e-05 |
| pos\_2119 | PS(14:0/14:0) | 4.60541483816281e-05 | 1.18695224942481e-05 | 7.16490827691356e-05 | 2.63692633337974e-05 | 4.52471220948806e-05 | 0.000254856904689883 | 7.15583181854285e-05 | 7.06321372200864e-05 | 9.74065981990039e-05 | 0.00014527709803392 | 5.96467024307516e-05 | 4.54543782103048e-05 |
| pos\_212 | Cytidine | 4.98089889274046e-09 | 8.6298752299599e-08 | 1.99602264684694e-07 | 2.453712791461e-05 | 2.58707537832229e-06 | 5.16534633787101e-06 | 9.16648489721465e-06 | 3.55564764229218e-06 | 1.28264028284956e-06 | 9.40658451086792e-06 | 5.12358848666809e-06 | 3.33045556274442e-07 |
| pos\_2120 | Cyanidin 3-(6''-malonylglucoside) | 5.06157564982006e-05 | 5.54654863326717e-05 | 5.08986836105749e-05 | 4.91001953351963e-05 | 4.68880634714245e-05 | 5.34072077145685e-05 | 4.9353065857406e-05 | 5.33345094716802e-05 | 6.03112036154211e-05 | 4.50406252115027e-05 | 5.41332653531285e-05 | 6.12909023535472e-05 |
| pos\_2122 | 6,7-dihydro-12-epi-LTB4 | 5.23114122661673e-05 | 6.61860494865881e-05 | 6.13334743385158e-05 | 5.56622102735305e-05 | 5.18263345575681e-05 | 5.74295970930128e-05 | 9.84123655554039e-05 | 8.71764070639232e-05 | 4.62680251228542e-05 | 9.04131185582185e-05 | 8.13568125371573e-05 | 8.37076014029753e-05 |
| pos\_2124 | N5-(4-Methoxybenzyl)glutamine | 3.90776240387211e-05 | 4.65035848110637e-06 | 6.07397838568024e-06 | 4.09323063580869e-06 | 1.11055137284858e-05 | 1.24038938556444e-05 | 1.82373021060141e-05 | 1.52727834442095e-05 | 9.37069486827221e-06 | 1.13404995794609e-05 | 1.34217001233111e-05 | 4.93892188827998e-05 |
| pos\_2126 | Nitrazepam | 2.94470328063075e-05 | 3.12506413466355e-05 | 3.14030892230066e-05 | 2.94951055523948e-05 | 2.96372316683515e-05 | 3.11676894917513e-05 | 2.27136588227637e-05 | 2.91403055519565e-05 | 3.64209769407456e-05 | 2.66816462234062e-05 | 2.9890703105451e-05 | 3.5329907948511e-05 |
| pos\_2127 | TG(22:4(7Z,10Z,13Z,16Z)/22:5(4Z,7Z,10Z,13Z,16Z)/22:6(4Z,7Z,10Z,13Z,16Z,19Z)) | 0.000137218923530953 | 0.000155750168141423 | 0.000176737741198772 | 8.10324452684475e-05 | 0.000201885247824817 | 0.000142128753633995 | 5.57425343054004e-05 | 0.000120556119824095 | 9.6144861211563e-05 | 0.000370652796686057 | 0.000148604280341751 | 0.000180628986168697 |
| pos\_2128 | Fauronyl acetate | 4.89339735949588e-06 | 1.34590592417407e-05 | 6.87376538679153e-06 | 1.26736726459491e-05 | 5.84914092557654e-06 | 6.91941049159445e-06 | 7.17569066077436e-06 | 1.07480114303727e-05 | 1.08710538218649e-05 | 4.55963843665433e-06 | 5.5228253412457e-06 | 1.09240620960083e-05 |
| pos\_2129 | 9-(2-Carboxyphenyl)-3,6-bis(diethylamino)xanthylium(1+) | 8.39801147997591e-05 | 6.39561287210397e-05 | 3.34394652334176e-05 | 8.21109589629087e-05 | 4.49213734426448e-05 | 6.00898352857905e-05 | 6.80011703451874e-05 | 6.76521821252175e-05 | 4.21473145807367e-05 | 6.75166536243983e-05 | 6.33737208782077e-05 | 3.87940652852906e-05 |
| pos\_213 | 5,6,7,8-Tetrahydro-2-naphthol | 1.03035162287383e-05 | 1.9107049838111e-05 | 8.37629999782233e-06 | 1.16649677550569e-05 | 1.60474505726808e-05 | 1.28797011055539e-05 | 1.44593557097777e-05 | 1.39340052564545e-05 | 1.45802205840684e-05 | 1.06761964206123e-05 | 1.28556554505475e-05 | 1.0789072060322e-05 |
| pos\_2130 | Cedryl acetate | 2.00048272005784e-05 | 2.23183381363897e-05 | 3.2757050475813e-05 | 3.34221672902682e-05 | 4.28799429143063e-05 | 3.1793146568781e-05 | 3.07555801880024e-05 | 2.76297191023579e-05 | 3.32042593139716e-05 | 3.21994513426652e-05 | 3.29849638858477e-05 | 3.01165566194354e-05 |
| pos\_2131 | 2-Decylfuran | 5.60534289838857e-05 | 7.03678216105668e-05 | 7.02552320796946e-05 | 6.08345886424687e-05 | 4.97889096458312e-05 | 6.15147241890116e-05 | 5.33430502753486e-05 | 6.2006997950288e-05 | 7.03793513347929e-05 | 5.98933311434559e-05 | 5.62405543506944e-05 | 5.78998380844529e-05 |
| pos\_2132 | ADP-Ribosyl-L-arginine | 3.04287831169887e-05 | 6.26838168430269e-05 | 2.4907940977663e-05 | 4.00366992293092e-05 | 5.94602779085077e-05 | 4.01552208441327e-05 | 2.53949133367564e-05 | 2.05625825028996e-05 | 6.61515126805021e-05 | 3.74739896622331e-05 | 3.65870287038458e-05 | 3.36338616793184e-05 |
| pos\_2134 | Blumealactone A | 2.82491352529398e-05 | 2.64599573521051e-05 | 9.84187299429003e-06 | 1.94257521587354e-05 | 2.47200777922947e-05 | 2.05263771416752e-05 | 1.81315469055958e-05 | 1.35549569831046e-05 | 1.76580464812433e-05 | 2.02308295139857e-05 | 1.8069021312758e-05 | 1.95414826495243e-05 |
| pos\_2135 | Aldosterone 18-glucuronide | 9.18992950179399e-06 | 1.03366922838581e-05 | 1.65311968977786e-06 | 1.00787110466321e-05 | 7.10299183162954e-06 | 7.16004481271239e-06 | 7.92649876149082e-06 | 4.0077115945412e-06 | 3.28186518452472e-06 | 1.6580413446235e-05 | 7.8056092553946e-06 | 3.55830796604163e-06 |
| pos\_2136 | Ornithokinin | 0.00149176296927288 | 0.00122360750462854 | 0.00111460878140196 | 0.00102307049177474 | 0.00106241876469368 | 0.00119933166155741 | 0.00114338858684588 | 0.00114479216800429 | 0.00121192520456339 | 0.00108751978699645 | 0.00115662049119823 | 0.00113197224637593 |
| pos\_2137 | Ceanothine D | 7.79345031723228e-06 | 1.1151134521959e-05 | 5.52861986444261e-06 | 1.39204909051136e-05 | 7.71826527589527e-06 | 7.89449572845813e-06 | 7.71226385576157e-06 | 1.30576035199736e-05 | 1.07187207956324e-05 | 1.06230057186331e-05 | 1.16748189037945e-05 | 1.32241041903746e-05 |
| pos\_2138 | PS(16:0/18:1(9Z)) | 0.000250793461694152 | 0.000311793792650228 | 0.000458700131231928 | 0.000289459182433455 | 0.000174793569936756 | 0.000267554761993063 | 0.000217740911660793 | 0.000303050573443221 | 0.000261236523095483 | 0.000214098451603201 | 0.000293160235133446 | 0.000246632857759152 |
| pos\_2139 | Desglucodesrhamnoparillin | 8.07664313717267e-06 | 5.29774083865406e-06 | 8.36483105935722e-07 | 3.13252915962222e-06 | 2.31670838968421e-06 | 3.69869840822463e-06 | 1.66591774057428e-06 | 6.05934000530515e-07 | 2.4808863736213e-06 | 7.82911626910462e-07 | 1.88920760193156e-06 | 1.86664800961816e-06 |
| pos\_214 | 2-Methylpropanal O-methyloxime | 0.000162445420980072 | 0.00019410122488313 | 0.000210469640762885 | 0.00014539364089437 | 0.000155517941910136 | 0.00016417816225023 | 0.00012484754206369 | 0.000159678556479845 | 0.000162370474356943 | 0.000214921423441532 | 0.000172520967185662 | 0.000183901928946569 |
| pos\_2140 | PE(18:3(6Z,9Z,12Z)/P-18:1(11Z)) | 0.00384355133372948 | 0.00125134326121691 | 0.00113316411089878 | 0.000954311732549616 | 0.00181657510079009 | 0.00173154489546523 | 0.00179689299790782 | 0.00143754562394017 | 0.00131652692057349 | 0.00128568178466339 | 0.00138615224030422 | 0.00133376205657581 |
| pos\_2141 | beta-D-Glucopyranosyl anthranilate | 5.13025804295272e-05 | 8.45770946159619e-05 | 4.66761725641007e-05 | 6.23242284655517e-05 | 6.24302027530323e-05 | 5.64263415408501e-05 | 4.66340287402742e-05 | 4.62048235730026e-05 | 6.34233984215488e-05 | 6.89034416994524e-05 | 5.27351732583658e-05 | 4.79588699659486e-05 |
| pos\_2142 | 3-carboxy-4-methyl-5-propyl-2-furanpropanoate | 0.000578744580310164 | 0.000652552452454275 | 3.61347431796016e-05 | 0.000304212906614103 | 0.000628584383162663 | 0.000411832961233399 | 3.91212298098121e-05 | 4.0204140868438e-05 | 3.94080373704698e-05 | 0.000313834577719133 | 9.65743642621704e-05 | 3.9624726298035e-05 |
| pos\_2143 | 12-Hydroxy-7-oxo-8,11,13-abietatrien-18-al | 7.24510722312031e-06 | 1.15852969341325e-05 | 6.69341749769901e-06 | 9.18123080438697e-06 | 9.73014945529692e-06 | 8.02785116698327e-06 | 2.71490548700779e-06 | 6.54063252583729e-06 | 6.45819409881474e-06 | 1.13052291576262e-05 | 9.1164914314923e-06 | 5.28686141778742e-06 |
| pos\_2147 | 2,5-Dihydro-2,4-dimethyloxazole | 8.0497164855881e-05 | 9.47661433610126e-05 | 8.81477010424826e-05 | 7.82156882542097e-05 | 7.21495439321551e-05 | 7.92191211417475e-05 | 7.787078039366e-05 | 8.10381066324324e-05 | 9.23394307896001e-05 | 7.75482978943982e-05 | 8.27398182391838e-05 | 7.56962207234552e-05 |
| pos\_2149 | repaglinide aromatic amine | 1.06641987176e-05 | 3.40682536145318e-05 | 1.27978506907918e-05 | 4.9443583034148e-06 | 3.39971701278497e-05 | 2.06963023045575e-05 | 3.2165779343501e-05 | 2.85299231149869e-05 | 6.37793361611532e-06 | 1.86943972603723e-05 | 2.04268700787957e-05 | 2.5465726584697e-05 |
| pos\_215 | 1,4,9-Cadinatriene | 0.000257859117246233 | 0.000424240288123345 | 0.000344425963308747 | 0.000388295317185365 | 0.000277611174519742 | 0.00033826631717196 | 0.000334733009040738 | 0.000317000785266293 | 0.000358380556339558 | 0.00035883326170701 | 0.000331133097772307 | 0.000286192596480138 |
| pos\_2150 | Betamethasone | 5.0151710655341e-06 | 1.71766524500923e-06 | 1.19744427758938e-06 | 1.07809324268472e-06 | 1.80509253646436e-06 | 2.42582701659502e-06 | 2.30429562903013e-06 | 0 | 3.05123107111885e-06 | 0 | 1.92652259092727e-06 | 3.27941948841288e-06 |
| pos\_2151 | PE(14:0/20:5(5Z,8Z,11Z,14Z,17Z)) | 5.60439495577819e-06 | 8.3110387549631e-06 | 1.40273975016928e-06 | 1.16534299441875e-08 | 2.55180524851851e-06 | 3.59307759554853e-06 | 8.70595534513904e-07 | 1.31200238758742e-06 | 0 | 3.89396316450397e-06 | 0 | 0 |
| pos\_2152 | Vomifoliol 9-[glucosyl-(1->4)-xylosyl-(1->6)-glucoside] | 1.07352317859342e-05 | 1.15078659202717e-05 | 8.17944198582465e-06 | 2.29708161633981e-08 | 4.0126230584121e-06 | 2.66242256929835e-06 | 3.10645253186537e-06 | 7.58371121990257e-07 | 2.51632368152903e-06 | 1.89942139699461e-06 | 2.74864173342628e-06 | 2.87648811636005e-06 |
| pos\_2154 | DG(14:0/0:0/18:2n6) | 9.43947471220182e-06 | 1.61400662406367e-05 | 1.06322360472398e-05 | 1.42585121352341e-05 | 1.9586756477983e-05 | 8.86588200264937e-06 | 1.31963704738194e-05 | 1.00240407209151e-05 | 4.18545666956977e-05 | 9.37256066285797e-06 | 9.32586646781506e-06 | 1.27769210961225e-05 |
| pos\_2155 | 3b,16a-Dihydroxyandrostenone sulfate | 1.08192729040346e-06 | 0 | 4.17814025298135e-07 | 1.85919914838539e-07 | 2.08217274487187e-06 | 3.77695031917591e-07 | 1.21693090018307e-06 | 4.95645047606854e-07 | 0 | 2.75098812389528e-07 | 4.9709724393657e-07 | 1.37391630952939e-06 |
| pos\_2157 | (R)-Apiumetin glucoside | 3.95666755654352e-06 | 3.76214175348091e-06 | 5.3917922206611e-06 | 4.52968964114105e-06 | 2.75014064394526e-06 | 4.74716215161591e-06 | 3.07098384491005e-06 | 4.57603222895372e-06 | 4.00040196065976e-06 | 5.10676843761384e-06 | 4.4413750569459e-06 | 5.91072767222246e-06 |
| pos\_2158 | PE(22:6(4Z,7Z,10Z,13Z,16Z,19Z)/24:1(15Z)) | 4.40548327089505e-05 | 4.87096694516003e-05 | 5.33129361740034e-05 | 7.07315417162826e-05 | 3.58098143223364e-05 | 5.31704747701709e-05 | 0.000129902559714265 | 0.000105969454534642 | 6.61846885796234e-05 | 6.21163889855879e-05 | 9.73023897994007e-05 | 6.49979019139092e-05 |
| pos\_2159 | PE(P-16:0/15:0) | 8.09542446849554e-06 | 9.93460440829283e-07 | 0 | 5.98703315857142e-07 | 1.98685515357455e-06 | 1.46111941287424e-06 | 7.79593817315804e-07 | 0 | 1.3441099620438e-06 | 0.000104373707189426 | 1.14750843604747e-05 | 0 |
| pos\_216 | Constanolactone E methyl acetonide | 0.000574761693745954 | 0.000620621680755875 | 0.00058160225420609 | 0.000654426743929019 | 0.000550735148809652 | 0.000596357237349899 | 0.000571819037449935 | 0.000627419711279697 | 0.000623389639000456 | 0.000602321475558477 | 0.000628199580581866 | 0.000684414912849337 |
| pos\_2162 | 2,5-Dimethylpyrazine | 7.14398824044962e-05 | 7.32672432657274e-05 | 6.39615639782503e-05 | 5.70267454781999e-05 | 0.000104376896286482 | 5.41464366854913e-05 | 9.51703792303071e-05 | 8.68419677310598e-05 | 7.07731522351497e-05 | 7.86321378338574e-05 | 7.14385929656564e-05 | 9.6789638178907e-05 |
| pos\_2163 | Vasopressin | 2.4615723921498e-05 | 5.91354617591669e-05 | 3.19932853073162e-05 | 3.15521869984874e-05 | 4.86092071067634e-05 | 4.86649703716165e-05 | 7.44719730599054e-05 | 4.39335285172298e-05 | 8.22422469379374e-05 | 0.000105040059002754 | 3.30770213795332e-05 | 1.29804101122195e-05 |
| pos\_2164 | (3x,5x,10x)-9,10-Didehydroisohumbertiol O-[rhamnopyranosyl-(1->4)-rhamnopyranosyl-(1->2)-[(E)-feruloyl-(->4)-rhamnosyl-(1->6)]-glucoside] | 0.000100586169639116 | 0.000113429636685022 | 0.000120439384923893 | 0.000102261725071727 | 9.49561357965793e-05 | 9.57680784596947e-05 | 0.000121232538573643 | 0.000120281712922964 | 0.000104773404298097 | 0.000110145169093128 | 0.000103692060709169 | 0.000105857266217774 |
| pos\_2165 | Balanitoside | 3.5903734172438e-05 | 6.24140987005419e-05 | 5.8209866581079e-05 | 7.56054659752629e-05 | 6.05196291979813e-05 | 6.79485716015405e-05 | 9.14991984788706e-05 | 7.07323665545011e-05 | 4.9318032768954e-05 | 5.93759151955627e-05 | 6.76258944686424e-05 | 6.19686415324732e-05 |
| pos\_2166 | Prostaglandin A2 | 2.93980159783118e-05 | 3.69461795613185e-05 | 2.35272054472896e-05 | 3.12104116543762e-05 | 1.54429747112814e-05 | 3.41072404510486e-05 | 2.42733270847517e-05 | 2.92619009875326e-05 | 2.52360151933427e-05 | 2.28155357816665e-05 | 3.32427227738562e-05 | 3.53767263666548e-05 |
| pos\_2167 | Trihexosylceramide (d18:1/18:0) | 3.15054270876698e-05 | 3.32816531004582e-05 | 4.12818327350299e-05 | 3.65218030425895e-05 | 2.52313906611318e-05 | 3.08198793481569e-05 | 3.27827479258771e-05 | 3.15640678960429e-05 | 3.53574885890771e-05 | 3.05009159101811e-05 | 3.05610723504779e-05 | 3.05689290125435e-05 |
| pos\_2168 | Trihexosylceramide (d18:1/24:1(15Z)) | 4.92150810108328e-05 | 4.08927922459094e-05 | 3.79487015848523e-05 | 2.57684187346281e-05 | 3.80517288555936e-05 | 2.94889343226261e-05 | 3.68827956742993e-05 | 3.94899839905375e-05 | 3.68605809548253e-05 | 3.66837163292636e-05 | 3.74861986097685e-05 | 4.12076585185785e-05 |
| pos\_2169 | Sapiol | 0.000822334247880501 | 0.00110844899825256 | 0.00104750046893106 | 0.000881089525531699 | 0.000762644825151853 | 0.000886097008599031 | 0.000798734695486567 | 0.000899171894405748 | 0.00102080154232035 | 0.000815151413306196 | 0.000881345811102233 | 0.000834812100558252 |
| pos\_217 | 2-Methyl-1,3-cyclohexadiene | 2.83030404270828e-06 | 4.58126622142134e-06 | 4.24896303371306e-06 | 3.55902605306828e-06 | 2.50371361130897e-06 | 3.3266826149252e-06 | 2.96753550856787e-06 | 3.75133558748415e-06 | 4.43596217085492e-06 | 3.16960400165347e-06 | 3.34669857958829e-06 | 3.39068301326672e-06 |
| pos\_2172 | 19'-Hexanoyloxymytiloxanthin | 1.36432299640174e-05 | 6.45967792115051e-06 | 0 | 0 | 5.32044012666141e-06 | 4.53625146176283e-06 | 5.80707567188402e-07 | 0 | 7.9761549586171e-06 | 3.0721005884084e-06 | 8.40674705117073e-07 | 1.83204678730368e-06 |
| pos\_2173 | Tetronasin | 0.000659504995999968 | 0.000277910391932362 | 0.00021754599993219 | 0.000276184285712328 | 0.000428551450506144 | 0.000433301397552204 | 0.000422524986074744 | 0.000381554069263352 | 0.000314251343118731 | 0.000332477143276451 | 0.000386705713786656 | 0.000367565738502211 |
| pos\_2174 | Myosmine | 3.03094733208626e-05 | 4.57765872434096e-05 | 3.64184316116014e-05 | 2.94026415094545e-05 | 2.8973224880206e-05 | 3.40692889743372e-05 | 3.19031353176944e-05 | 3.29537423305497e-05 | 6.2005614479467e-05 | 2.82650219510926e-05 | 3.99108357769723e-05 | 3.83709484140277e-05 |
| pos\_2175 | CDP-DG(16:0/18:1(11Z)) | 1.26571404627091e-05 | 3.514834352234e-06 | 2.45826884919968e-05 | 8.48288866489688e-06 | 1.72888397103832e-05 | 9.47954471410324e-05 | 2.89695858965e-05 | 3.11617232354661e-05 | 3.7038499506261e-05 | 5.72733810187072e-05 | 2.52259123186743e-05 | 1.79009581712316e-05 |
| pos\_2176 | Megastigmatrienone | 0.000557550286722852 | 0.00075587710148937 | 0.00177911006848421 | 0.00149600714143251 | 0.00125555168056355 | 0.00116230326700438 | 0.00104289352785541 | 0.000935518722699339 | 0.000821070815310878 | 0.000735492341069668 | 0.000910168584134543 | 0.000885009806050383 |
| pos\_2177 | 3'-N-Acetyl-4'-O-(9-octadecenoyl)fusarochromanone | 0.00212085471113024 | 0.00359616167563225 | 0.00202357823825521 | 0.00236357040319947 | 0.00229190814483994 | 0.00269558914947341 | 0.00204565149068489 | 0.00214264149309842 | 0.00279808724031414 | 0.00210604297999432 | 0.0023700188822287 | 0.00212945939356706 |
| pos\_2179 | Licoricesaponin A3 | 5.27306455167551e-06 | 1.25710148448618e-05 | 9.8911000301095e-06 | 8.55857561109726e-06 | 4.66742070742138e-06 | 1.11874362998736e-05 | 5.54497888659997e-06 | 8.42042308498331e-06 | 1.15194108827889e-05 | 8.50169575050714e-06 | 1.08513290758264e-05 | 8.37808647774259e-06 |
| pos\_218 | 1α-hydroxy-2β-(2-hydroxyethoxy)vitamin D3 / 1α-hydroxy-2β-(2-hydroxyethoxy)cholecalciferol | 8.76360013864739e-05 | 0.000103551976745287 | 0.000110778711684949 | 0.000117641511081396 | 9.44952556292891e-05 | 0.000118793088518968 | 0.000104893892937612 | 0.00011162184326099 | 0.000121100324874012 | 8.91871727826089e-05 | 0.000114933372714312 | 8.78973076126596e-05 |
| pos\_2180 | 11-Hydroxyprogesterone 11-glucuronide | 0 | 0 | 3.94663470290798e-05 | 8.7240619559658e-05 | 3.18172902205505e-06 | 2.05254713675505e-05 | 0 | 3.95121100688228e-05 | 0.000111013105296323 | 0 | 3.62688275872492e-05 | 9.38026066897688e-05 |
| pos\_2181 | Quinquenoside IV | 4.23652509600547e-06 | 4.19573530716413e-06 | 5.31142309690933e-06 | 5.73952598327274e-06 | 7.2586242171764e-06 | 8.74452643406539e-06 | 3.91817670424176e-06 | 6.7593870554109e-06 | 1.13952002007271e-05 | 7.12444225568199e-06 | 9.53623443473152e-06 | 1.208342911402e-05 |
| pos\_2182 | 5-Methylcytidine | 0.000113051246716882 | 0.000128016048495834 | 0.000104735723671931 | 0.000103479125893857 | 9.26659832512142e-05 | 0.000108699790030088 | 0.000102562379670151 | 0.000129110036614428 | 9.71292766813087e-05 | 0.000114330264772113 | 0.000102646460298155 | 9.84922427550799e-05 |
| pos\_2183 | Isolariciresinol 9-O-beta-D-glucoside | 3.45389970969578e-05 | 2.72332216242187e-05 | 2.52630513673872e-05 | 2.87392071507409e-05 | 4.48199560153389e-05 | 2.72212721847459e-05 | 4.61433829816745e-05 | 3.93732266537002e-05 | 3.77102443985278e-05 | 3.14265009612004e-05 | 3.58810724538911e-05 | 3.24764252815007e-05 |
| pos\_2184 | Zapotidine | 3.64412276626205e-05 | 4.7024877922682e-05 | 4.88544804019455e-05 | 3.94632195848878e-05 | 3.26242315519055e-05 | 4.21916043524366e-05 | 3.58170532267681e-05 | 3.96191270602544e-05 | 4.69501474326007e-05 | 4.3345054092334e-05 | 4.00700276377977e-05 | 3.78465349325922e-05 |
| pos\_2185 | 2-(1-Propenyl)-delta1-piperideine | 4.99207069411798e-05 | 5.71661209052485e-05 | 5.81346494525513e-05 | 4.67020603470729e-05 | 4.6249993628653e-05 | 5.39114137259738e-05 | 4.31694786849872e-05 | 4.64364675981324e-05 | 4.13609730842022e-05 | 6.53148465185785e-05 | 5.12335243190852e-05 | 5.56013258619121e-05 |
| pos\_2186 | Mucronine D | 0.000389979825150108 | 0.000206514373239962 | 0.000194777469916308 | 0.000179189403658512 | 0.000209707539311342 | 0.000223106377507456 | 0.000231942555227543 | 0.000212395828995147 | 0.00021877130146365 | 0.000193476700771311 | 0.000206835659070127 | 0.000207152643515563 |
| pos\_2187 | 1-Pentanethiol | 2.2300171462593e-06 | 1.49943022433886e-06 | 4.03308690051302e-06 | 3.87411423269537e-06 | 4.20662672690456e-06 | 3.55972008475088e-06 | 3.60560850969083e-06 | 3.51571713468624e-06 | 3.90424515063094e-06 | 3.29596512483362e-06 | 3.21377845443326e-06 | 3.52423100391031e-06 |
| pos\_2189 | N-Methylethanolaminium phosphate | 0.00633480572670358 | 0.00825912747199961 | 0.00746036297543805 | 0.0068003007562015 | 0.0057784313743923 | 0.00680863410800591 | 0.00609827053611266 | 0.00670657820032671 | 0.00745508685587055 | 0.00603060598486737 | 0.0067687600058824 | 0.00642752153024936 |
| pos\_219 | 3,7,11,15-tetramethyl-2E,6E,10E,14-hexadecatetraenal | 7.65998313417718e-05 | 7.49704219051513e-05 | 4.24326899894744e-05 | 5.23187070707616e-05 | 0.000102747887431754 | 7.13979147929281e-05 | 4.18988965679432e-05 | 4.88897012244353e-05 | 5.5336458601679e-05 | 7.79156825284407e-05 | 5.08599634534013e-05 | 5.0654333217414e-05 |
| pos\_2190 | Daidzein 4',7-diglucoside | 1.6468462868374e-06 | 3.14036128589092e-06 | 2.26357177255874e-06 | 4.45456888266427e-06 | 1.69791556784416e-06 | 1.72566667842953e-06 | 2.29251702699859e-06 | 2.38136541496106e-06 | 2.92716733051188e-06 | 2.18611203090226e-06 | 1.97667777710146e-06 | 1.71305279324483e-06 |
| pos\_2191 | Glycinexylidide | 0.000314654877251857 | 0.000187814639919368 | 4.19825912545813e-05 | 0.000100704986804761 | 0.000157240674571781 | 0.000145822520019375 | 6.40206713073059e-05 | 5.42304792506709e-05 | 5.96120754802832e-05 | 0.000133003172185503 | 6.30895311752978e-05 | 6.76672545869567e-05 |
| pos\_2192 | PS(DiMe(11,5)/DiMe(13,5)) | 0.000135497673892434 | 0.000110663171587337 | 0.00010003151668548 | 5.32899373656628e-05 | 7.85400181601917e-05 | 7.82204022482555e-05 | 6.44341042307777e-05 | 4.77398796837856e-05 | 5.45468381921281e-05 | 4.8052170767097e-05 | 4.64403051174801e-05 | 7.07693234307901e-05 |
| pos\_2194 | Myricetin | 3.09760809583367e-06 | 2.80657812120533e-06 | 0.000201337836068982 | 0.000422489750840674 | 3.92642047974313e-05 | 0.000146351459584577 | 1.66354214224596e-06 | 0.00019660097358223 | 0.000500035827655774 | 8.81874282811352e-06 | 0.000252599597788372 | 0.00047059918088289 |
| pos\_2195 | PG(18:0/18:0) | 9.38988700395841e-06 | 5.31365042667359e-06 | 3.63216176742778e-06 | 1.08545827833566e-06 | 5.62397443747261e-06 | 4.01073521123722e-06 | 4.08794814846407e-06 | 3.06225022328521e-06 | 5.91139955692567e-06 | 0 | 1.64260436113706e-06 | 8.72744393542493e-07 |
| pos\_2196 | Pectenotoxin 3 | 1.33596983046455e-05 | 1.79916144750811e-05 | 1.45715430456606e-05 | 1.25738260038678e-05 | 2.18155619504515e-05 | 1.50300876139465e-05 | 1.9475174880179e-05 | 2.44957197869696e-05 | 2.35501092400871e-05 | 3.14564743524474e-05 | 2.69186108559527e-05 | 3.5921053755078e-05 |
| pos\_2199 | Paradisin C | 8.96301121205312e-06 | 1.34148385432485e-06 | 2.28827887026962e-06 | 8.56785380610699e-07 | 0 | 3.81458476479564e-06 | 4.28127851854417e-06 | 0 | 4.63995123855836e-06 | 3.3140755713762e-06 | 0 | 7.66206060565091e-07 |
| pos\_22 | 6-pentadecyl Salicylic Acid | 0.00371059785949516 | 0.00307647757265096 | 0.00411121621840005 | 0.00550001966205316 | 0.00482827859737639 | 0.00468163412906954 | 0.00562559106607099 | 0.00423129240645645 | 0.00444055865986892 | 0.00487875643580297 | 0.00476057552697837 | 0.00422877707210239 |
| pos\_220 | Retinaldehyde | 7.68542489652734e-05 | 6.68515793440878e-05 | 0.000146764783278822 | 2.62070226532705e-05 | 0.000105352568695911 | 8.01733707816757e-05 | 1.9191654832609e-05 | 5.13620875253435e-05 | 2.20082971845503e-05 | 0.000262916035310951 | 8.35820664832983e-05 | 8.82444000624729e-05 |
| pos\_2201 | Isosalicin | 1.52651923572481e-05 | 1.90016764394603e-05 | 6.32411788644893e-06 | 1.05287164950691e-05 | 1.0121259930944e-05 | 1.18744413745884e-05 | 7.60639576133944e-06 | 7.16133565178527e-06 | 1.22966295850384e-05 | 2.34544229142454e-05 | 1.3714876342296e-05 | 1.10207816101057e-05 |
| pos\_2202 | Pipercyclobutanamide B | 3.28261667261905e-06 | 3.44702763379699e-06 | 1.57031847558196e-06 | 5.05840739383282e-06 | 3.14259247618784e-06 | 3.22433074165169e-06 | 4.7493202631896e-06 | 3.35633917975686e-06 | 7.80571365224153e-06 | 2.31591655010121e-06 | 4.41817834778365e-06 | 3.66638877518399e-06 |
| pos\_2203 | 3-Indolehydracrylic acid | 6.86344148122482e-05 | 0.000105401152941636 | 5.84323844963681e-05 | 8.3060997052997e-05 | 8.15515336687234e-05 | 7.33739369095413e-05 | 7.73954700288465e-05 | 6.95631046591944e-05 | 9.02342394164169e-05 | 8.4422797770751e-05 | 7.14082558548101e-05 | 5.63423322446967e-05 |
| pos\_2204 | Diethanolamine | 2.9278266643697e-05 | 3.42953960450761e-05 | 3.74389947876981e-05 | 3.69613595016417e-05 | 3.67895812968327e-05 | 3.0877328982355e-05 | 3.0193757307667e-05 | 3.25942598591676e-05 | 3.40896607002865e-05 | 3.3543608077335e-05 | 3.10888674363378e-05 | 3.39428966263502e-05 |
| pos\_2205 | N-Methylnicotinium | 0.000213984856803759 | 0.000248123633034943 | 0.000251889632773901 | 0.000217787112422728 | 0.000184581321987454 | 0.000209182369448579 | 0.000190796936059469 | 0.000210227588012606 | 0.000222547065941459 | 0.000227712558020664 | 0.000214990481769389 | 0.000212791796179292 |
| pos\_2206 | PE(18:4(6Z,9Z,12Z,15Z)/24:1(15Z)) | 0.00486812864754613 | 0.00686913567366282 | 0.00594041327015174 | 0.00646259650185054 | 0.00507226308488003 | 0.00612123372738505 | 0.00552961554576932 | 0.00566265593727248 | 0.00700979945069218 | 0.00528879958255302 | 0.0060918793625918 | 0.00518277204811119 |
| pos\_2207 | Azukisaponin III | 2.00667054538262e-05 | 1.39424524358297e-05 | 2.60283319874502e-05 | 1.86508333437368e-05 | 6.55572520446865e-06 | 1.31618064101987e-05 | 1.36694826961862e-05 | 1.58529403072893e-05 | 1.56984038547237e-05 | 1.16231586022384e-05 | 8.69901773447783e-06 | 1.06910504262447e-05 |
| pos\_221 | 2-N-Undecyltetrahydrothiophene | 5.17653364983663e-06 | 6.79865453900511e-06 | 1.25424379477192e-05 | 1.40170062837063e-05 | 1.8769883767587e-05 | 1.14309364219755e-05 | 1.02469061458026e-05 | 1.07246788516856e-05 | 1.41926071678221e-05 | 1.06602123395873e-05 | 1.0937324757343e-05 | 1.05177709098244e-05 |
| pos\_2212 | 2'',6''-Di-O-acetylononin | 2.34334080224384e-05 | 2.0405945202407e-05 | 1.82696832915326e-05 | 1.65412300936037e-05 | 2.30996606653594e-05 | 2.64838424482661e-05 | 2.45351018511708e-05 | 2.23092052674788e-05 | 2.20819741840235e-05 | 1.8313717063101e-05 | 2.11755265323452e-05 | 2.10258214602198e-05 |
| pos\_2214 | 4-[(2-Furanylmethyl)thio]-2-pentanone | 3.92518850998298e-06 | 2.84304652951494e-06 | 1.28413190340373e-05 | 2.88161502124256e-05 | 9.17648360279906e-06 | 8.56910428366476e-06 | 1.12022313981609e-05 | 1.34471376564511e-05 | 1.36903295294154e-05 | 1.28838151900407e-05 | 1.37663382827886e-05 | 2.68025805363004e-05 |
| pos\_2216 | 7-Ethyl-2,3,6,7-tetrahydrocyclopent[b]azepin-8(1H)-one | 2.19759680310367e-05 | 1.14925160835313e-05 | 6.6277052182357e-05 | 2.21677070424663e-05 | 3.32615308903227e-05 | 0.000207242196686382 | 4.90407771670413e-05 | 5.54177844373486e-05 | 8.08912922373971e-05 | 0.000119080124438238 | 4.38563273022805e-05 | 3.45241434739189e-05 |
| pos\_2217 | 7-Chloro-3,4',5,6,8-pentamethoxyflavone | 3.50411173562552e-05 | 4.35368822175474e-05 | 3.25983293520926e-07 | 1.74835049950224e-05 | 4.12308757833539e-05 | 2.40837821661211e-05 | 6.98450245997044e-06 | 2.30131437801194e-06 | 1.20898727267271e-06 | 2.19966285201795e-05 | 5.93522047948137e-06 | 3.63275017897762e-07 |
| pos\_2218 | Bilirubin diglucuronide | 1.2398832645844e-05 | 9.24526602173704e-06 | 0 | 1.52361398621242e-06 | 1.52214112274094e-05 | 6.01225476192585e-06 | 0 | 0 | 0 | 2.7848740219841e-06 | 0 | 0 |
| pos\_2219 | LysoPE(0:0/22:5(4Z,7Z,10Z,13Z,16Z)) | 0.000353901094898488 | 0.000161945345053921 | 0.000175014260303738 | 0.000139427418398838 | 0.000231116425011001 | 0.000247274081563488 | 0.0002537704871906 | 0.000223314005801253 | 0.000199263907952587 | 0.000197035281161362 | 0.000214487406436354 | 0.000229770046615859 |
| pos\_222 | 3,5-Dihydroxybiphenyl | 1.00774963503255e-06 | 5.23598151287401e-07 | 3.4298737291759e-06 | 8.91088368257411e-06 | 1.34495805374536e-06 | 3.13736445710773e-06 | 8.82839797347313e-07 | 5.14770553899373e-06 | 3.53158221213209e-07 | 5.94219471647573e-08 | 3.2791862796782e-06 | 1.07833134865832e-05 |
| pos\_2221 | Matsutakic acid A | 0.000242659901320921 | 0.000233134208608594 | 0.000206720193916594 | 0.000235405101099329 | 0.000201940087940583 | 0.000216823628662381 | 0.000285105912362693 | 0.000245726830162415 | 0.00027238371885424 | 0.000272070812398608 | 0.000265439672753517 | 0.000231674805601176 |
| pos\_2222 | Tocophersolan | 0.000368134728883743 | 0.000476284970688403 | 0.000388772327399315 | 0.000377865385305169 | 0.000349112354556853 | 0.000386965811162174 | 0.000372139688290075 | 0.000368654239296548 | 0.0004421557065839 | 0.000352466890647138 | 0.000369771804546218 | 0.000345706828494936 |
| pos\_2224 | DG(18:0/22:5(7Z,10Z,13Z,16Z,19Z)/0:0) | 0.000102113293548936 | 0.000130126206016543 | 0.000114791431546745 | 6.62309336468605e-05 | 8.04035191264517e-05 | 0.000110242857009095 | 0.000124739255913165 | 0.000148292904808106 | 0.000103344102072537 | 0.000100652984411641 | 0.00014100900541563 | 0.000253902939525508 |
| pos\_2225 | Ginsenoside Rh7 | 0.00020407960418521 | 7.81092517688013e-05 | 6.55696236600296e-05 | 6.94810868243843e-05 | 8.37502191307909e-05 | 8.35974757270525e-05 | 7.49422882062228e-05 | 6.92946539192296e-05 | 9.10841071240559e-05 | 6.99877493612297e-05 | 7.12005841110326e-05 | 6.37041911108565e-05 |
| pos\_2226 | 3-Oxooctadecanoic acid | 7.53995883283602e-05 | 7.89418944507548e-05 | 7.30218372098261e-05 | 7.68478885894957e-05 | 9.96527197762701e-05 | 6.5205099775442e-05 | 0.000186815981350436 | 0.000132565861306094 | 8.25813885034055e-05 | 0.000115102788203717 | 9.88364589332852e-05 | 0.000103503562631583 |
| pos\_2227 | Tanakamine | 5.62772064224383e-05 | 6.21325198477085e-05 | 4.67564062850082e-05 | 5.83376093356597e-05 | 4.12690447285176e-05 | 5.13636797932879e-05 | 5.54033243294361e-05 | 5.15691715151627e-05 | 6.72754783870188e-05 | 3.63561430612322e-05 | 5.02553865009697e-05 | 4.07820813633648e-05 |
| pos\_2228 | 3-O-Sulfogalactosylceramide (d18:1/14:0) | 3.58055609564871e-06 | 1.06373175470737e-05 | 9.75607234381912e-06 | 8.53577237553351e-06 | 5.19156992262258e-06 | 7.27145763929625e-06 | 5.53233604819836e-06 | 6.3521395525215e-06 | 9.30956290303535e-06 | 7.01872053976047e-06 | 7.47814489221442e-06 | 6.87411068404669e-06 |
| pos\_2229 | PIP(16:0/22:4(10Z,13Z,16Z,19Z)) | 1.18531416430868e-05 | 1.21221854490714e-05 | 1.13578758031654e-05 | 1.21295261943148e-05 | 1.09707852574034e-05 | 1.39047517751989e-05 | 1.23845837680716e-05 | 1.42305160382724e-05 | 1.26538897229128e-05 | 1.24577164364554e-05 | 1.21775227933782e-05 | 1.30469842333254e-05 |
| pos\_223 | Elaiophylin | 0.000127815415165859 | 2.28606097201211e-05 | 3.10073322926886e-05 | 2.46250149834989e-05 | 7.86281700753341e-05 | 7.07173277793271e-05 | 6.02332237407424e-05 | 5.17108680387223e-05 | 3.37072642059093e-05 | 4.52309887840684e-05 | 4.23660933791002e-05 | 5.00568025478097e-05 |
| pos\_2230 | N-hexadecanoylsphinganine-1-phosphocholine | 0.000243513916796587 | 0.000195902638768357 | 6.8328425824287e-05 | 0.000191857834976668 | 0.00012352584471655 | 0.000183680087644128 | 0.000697716968074904 | 0.000399915900529054 | 6.11364027757567e-05 | 0.000261105533150883 | 0.000304849514856002 | 2.96384285926176e-06 |
| pos\_2231 | Myricetin 3-(6-acetylgalactoside) | 0 | 0 | 1.10666648592802e-05 | 3.33964574397402e-06 | 0 | 3.12314427654278e-06 | 4.32437968330666e-06 | 3.82681556413491e-06 | 0 | 0 | 3.91217387464809e-06 | 6.99970792839155e-06 |
| pos\_2232 | Lucidenic acid L | 8.40435960674468e-05 | 9.59192611552736e-05 | 4.21472645865105e-05 | 4.60046373341267e-05 | 7.90569230720145e-05 | 7.11884119485424e-05 | 6.87843676054408e-05 | 5.49645386420553e-05 | 4.71398622903733e-05 | 8.47472827092534e-05 | 5.95660141497837e-05 | 5.58063352573564e-05 |
| pos\_2233 | L-gamma-glutamyl-L-leucine | 3.97241333157718e-05 | 4.64428228121863e-05 | 3.8187165221488e-05 | 0.000221251557367694 | 2.13600606552801e-05 | 5.99608486258831e-05 | 0.000319354710893287 | 0.000200494599131448 | 0.000175519567327438 | 1.15749241286857e-05 | 0.000172657272316623 | 6.96806821383929e-05 |
| pos\_2234 | 5-(3',4',5'-Trihydroxyphenyl)-gamma-valerolactone-O-methyl-O-sulphate | 1.12976907600878e-05 | 7.05096423883679e-06 | 6.0274798754268e-06 | 6.26184980638616e-06 | 5.73416879861459e-06 | 9.89307227188635e-06 | 1.15116575906677e-05 | 9.1264135153974e-06 | 9.79611225481662e-06 | 9.1253846956016e-06 | 1.00812058859888e-05 | 9.65517142833155e-06 |
| pos\_2235 | Epicatechin-(4beta->8)-epigallocatechin 3-O-gallate | 1.13354272517149e-05 | 2.52801072017513e-05 | 9.1584019756923e-06 | 1.04784636047448e-05 | 1.39344683001281e-05 | 1.24437242429503e-05 | 1.02906212017499e-05 | 1.10350836328013e-05 | 1.47856231978871e-05 | 1.82634896279106e-05 | 1.3352322754129e-05 | 6.02162365127643e-05 |
| pos\_2236 | DG(18:0/0:0/20:4n6) | 0.000318281124348581 | 4.14625400692242e-05 | 5.0474726628569e-05 | 3.37894617472304e-05 | 0.000149980311933155 | 0.000142174700680304 | 0.000126706318038475 | 0.000109803223737999 | 6.89691848080913e-05 | 8.68003454148528e-05 | 0.000101280037798484 | 8.82672968302838e-05 |
| pos\_2237 | 1-(1,2,3,4,5-Pentahydroxypent-1-yl)-1,2,3,4-tetrahydro-beta-carboline-3-carboxylate | 7.5120328095785e-06 | 8.25256800453696e-06 | 0 | 1.8405469886444e-06 | 2.41435354428127e-06 | 6.0503396741447e-06 | 7.39514170445669e-07 | 2.5981375418073e-06 | 1.49644643062806e-06 | 1.36004204641564e-06 | 1.07905829713708e-06 | 1.05283609005274e-06 |
| pos\_2239 | CDP-DG(16:0/22:6(4Z,7Z,10Z,13Z,16Z,19Z)) | 5.45212184994042e-06 | 1.82066138681808e-05 | 1.84277692530972e-05 | 1.85296126499188e-05 | 1.64429695740997e-05 | 1.61972083420225e-05 | 2.12539845509043e-05 | 2.67550951379954e-05 | 1.94512799102411e-05 | 2.73351384257198e-05 | 2.34547744232487e-05 | 1.70754116550424e-05 |
| pos\_224 | Farnesyl acetone | 0.000245119364503586 | 0.00025831556327589 | 0.000198361878475047 | 0.000256805060727065 | 0.00120321732395805 | 0.000271310622763872 | 0.00267262695816822 | 0.00157644789140625 | 0.000288730925613434 | 0.00145230157683969 | 0.000581184568589861 | 0.000820953603793671 |
| pos\_2240 | Ganglioside GA2 (d18:1/20:0) | 2.57408566737434e-05 | 2.74894992325055e-05 | 2.25678907477024e-05 | 2.21972763960649e-05 | 2.12728350040036e-05 | 1.87695147044274e-05 | 2.23221574268588e-05 | 2.19686259339991e-05 | 2.2782755735697e-05 | 2.45460566837777e-05 | 2.18167240189907e-05 | 1.95440801616902e-05 |
| pos\_2241 | Tragopogonsaponin L | 1.10247638215202e-05 | 3.81821147617669e-06 | 1.11862847114496e-06 | 0 | 1.02164107317029e-06 | 2.50603158906447e-06 | 5.75900625476984e-07 | 2.77650192193473e-06 | 7.30837980509104e-08 | 3.67609137819528e-06 | 7.33280619243242e-06 | 0 |
| pos\_2244 | 3beta-Hydroxy-5-cholestenal | 5.04948043445959e-05 | 5.26086979778108e-05 | 3.40341236046842e-05 | 4.50645239480142e-05 | 4.62782961536469e-05 | 4.60228392771215e-05 | 5.4953745293709e-05 | 4.64822191780921e-05 | 5.49450499672159e-05 | 4.95044233334574e-05 | 5.22592778620273e-05 | 5.06366120614069e-05 |
| pos\_2245 | (+)-Setoclavine | 0.000129205787444673 | 0.000121553502491445 | 0.000108000180703382 | 0.000102475577175422 | 0.000102012094934681 | 0.00010744078760863 | 0.000109382600670999 | 0.000111555894405933 | 0.00011302950273933 | 0.000106368460239628 | 0.000112708678862767 | 0.000106790876931723 |
| pos\_2246 | 4-[(2-Methyl-3-furanyl)thio]-5-nonanone | 2.90558525957952e-05 | 3.42277701670884e-05 | 3.58731500555858e-05 | 2.89745443963513e-05 | 2.71896491631735e-05 | 3.36128770689585e-05 | 3.10492811390759e-05 | 3.4615167732066e-05 | 3.75903379919985e-05 | 2.82004489956726e-05 | 2.93867553547869e-05 | 3.14536296205732e-05 |
| pos\_2247 | PS(MonoMe(11,3)/MonoMe(11,5)) | 8.90062026840467e-05 | 6.43044795767284e-05 | 5.34521739219127e-05 | 4.57699515318615e-05 | 4.61975957032653e-05 | 6.44929741106626e-05 | 5.16671470395488e-05 | 5.52362409900828e-05 | 5.47580663124932e-05 | 5.16513626228695e-05 | 4.52533033107515e-05 | 5.28203010986125e-05 |
| pos\_2248 | Flunitrazepam | 2.99691573789716e-08 | 4.30294111109389e-07 | 2.91208607750465e-05 | 1.57304380644631e-07 | 9.40022041565355e-08 | 1.68368391307365e-07 | 7.5776232291637e-07 | 1.76850754870433e-06 | 7.08161611667704e-09 | 2.21813786575566e-06 | 7.35771196453514e-06 | 2.55559907527971e-07 |
| pos\_225 | Grenoblone | 0.000396925678977738 | 0.000398410109505882 | 0.000412181906107489 | 0.000385050663547021 | 0.000367278088235955 | 0.000407616783583029 | 0.000292226131500842 | 0.000374133847425055 | 0.000448219081840334 | 0.000332823778172132 | 0.00038306859582583 | 0.00043748584400595 |
| pos\_2250 | 1alpha,3beta,22R-Trihydroxyergosta-5,24E-dien-26-oic acid 3-O-b-D-glucoside 26-O-b-D-glucosyl ester | 9.90485976966278e-06 | 1.98270390046423e-06 | 1.47505246651392e-06 | 1.73736237314369e-06 | 5.74801109961783e-06 | 4.50468530020132e-06 | 3.69656989183649e-06 | 3.57462431844522e-06 | 2.50081639046681e-06 | 2.92482283255742e-06 | 3.64137505930035e-06 | 2.66820682107507e-06 |
| pos\_2252 | (1S,2S,4R,8S)-p-Menthane-1,2,8,9-tetrol 2-glucoside | 5.59345106969021e-06 | 4.91120431875746e-05 | 2.0733504304233e-05 | 9.46817778333853e-07 | 2.18122501516908e-05 | 1.60287827348891e-05 | 1.13280715796302e-05 | 1.00405981703949e-05 | 1.52343538381578e-06 | 5.22050134872103e-05 | 1.49218976901436e-05 | 1.25453862388707e-05 |
| pos\_2255 | 3,7-Bisaboladiene-2,8-dione | 4.28723990098057e-05 | 4.39129403238659e-05 | 4.73569941425433e-05 | 4.69654381758154e-05 | 5.05243630649869e-05 | 4.7282252982687e-05 | 4.2675110745567e-05 | 4.5107135080527e-05 | 4.62006911449699e-05 | 4.36715085060928e-05 | 4.71271229427647e-05 | 5.49989259228955e-05 |
| pos\_2257 | Tricycloekasantal | 0.000125257746768255 | 0.000168697867280157 | 0.000149061157481297 | 0.000196155302697874 | 0.0001411184368571 | 0.000157865850868064 | 0.000191995153806432 | 0.0001687558585968 | 0.000155801060668625 | 0.000160460666555508 | 0.000163033284500416 | 0.000147923033282729 |
| pos\_2258 | Isopentenyladenine-9-N-glucoside | 3.25428695246486e-05 | 3.36639032533984e-05 | 1.64263384009674e-05 | 2.39256273167684e-05 | 2.68638210016169e-05 | 1.79153336007093e-05 | 3.75368587766606e-05 | 2.77201287773318e-05 | 2.27476165352634e-05 | 2.68440529522964e-05 | 2.53722593690087e-05 | 3.20448773333357e-05 |
| pos\_2259 | Janthitrem E | 5.78653665607303e-06 | 1.60430223035918e-06 | 3.19927936980768e-06 | 1.68925593831867e-06 | 4.06449023736927e-06 | 2.94122785322238e-06 | 2.63350090398143e-06 | 3.06916659596771e-06 | 3.7715219414937e-06 | 3.10373563836443e-06 | 2.35887939090187e-06 | 2.7560125188851e-06 |
| pos\_226 | Harderoporphyrin | 0.00355221864896857 | 0.00213800113249676 | 0.00237303288061545 | 0.0022492167966746 | 0.00413685776848636 | 0.00273440391122377 | 0.00298553969649361 | 0.00335357716695805 | 0.00251916861258794 | 0.00385503962418653 | 0.00221285813533353 | 0.00336943986995608 |
| pos\_2261 | 7-Pentacosanone | 4.709857128318e-05 | 4.88779450612855e-05 | 4.70534104619886e-05 | 5.6661278543225e-05 | 2.94283067719847e-05 | 4.33035460332313e-05 | 3.68552616579396e-05 | 4.33330451866291e-05 | 4.22645037532693e-05 | 5.86089467603922e-05 | 4.48864360952276e-05 | 4.27688710101635e-05 |
| pos\_2262 | TG(24:0/24:0/22:5(4Z,7Z,10Z,13Z,16Z)) | 2.30747570376795e-06 | 3.2205861878453e-06 | 3.88101659571968e-06 | 3.06591646777927e-06 | 2.90769919403156e-06 | 1.25910577702775e-06 | 2.08115064064393e-06 | 2.19847691028294e-06 | 2.60789079916441e-06 | 1.98719644959534e-06 | 2.6542866055901e-06 | 2.59009035697715e-06 |
| pos\_2263 | LysoPC(20:2(11Z,14Z)) | 0.000168472724004162 | 0.000227200949640248 | 0.000391661418165005 | 8.1073879064522e-05 | 0.000237650772459535 | 0.000312586939467875 | 5.27064021882792e-05 | 0.000174812458794954 | 9.51785087096436e-05 | 0.000532250870596888 | 0.000263374911958564 | 0.000329645299167421 |
| pos\_2264 | 25-Acetyl-6,7-didehydrofevicordin F 3-glucoside | 8.73539939910868e-06 | 4.02823325272518e-06 | 3.55962642010008e-06 | 3.40111543795635e-06 | 8.2184372541476e-07 | 2.70558094407792e-06 | 1.7958549539772e-06 | 1.91792813628293e-06 | 4.97030056049441e-06 | 0 | 4.62252054631674e-06 | 5.1526033504334e-06 |
| pos\_2265 | 6-Heptenyl glucosinolate | 0.000209019293523944 | 1.19477014631416e-05 | 1.92026406152446e-05 | 1.07475350682081e-05 | 9.70790022465385e-05 | 8.29577447504533e-05 | 7.17262306601632e-05 | 5.69570898553608e-05 | 2.63585364624761e-05 | 4.08922205977758e-05 | 4.74192805902293e-05 | 3.81657330516871e-05 |
| pos\_2266 | Kaempferol 3-O-caffeoyl-sophoroside 7-O-glucoside | 7.72579374135352e-05 | 2.03867612361379e-05 | 1.69103499203632e-05 | 1.70181886892564e-05 | 4.45814601158141e-05 | 3.86777422889931e-05 | 3.86118207098505e-05 | 3.09876530403073e-05 | 2.41010550295429e-05 | 3.20204817162651e-05 | 2.98033748266212e-05 | 2.88407561340213e-05 |
| pos\_2268 | 12b-Hydroxy-5b-cholanoic acid | 0.000499538648191211 | 0.000365689487396892 | 0.000267341821736112 | 0.000306356998774828 | 0.000367260736442171 | 0.000378794666104633 | 0.000423876596103354 | 0.000350140282096546 | 0.000356394815787741 | 0.000348830469629106 | 0.000348214236509751 | 0.000335267084395012 |
| pos\_227 | Benzhydrylpiperazine | 0.000167121344336185 | 0.000186013190373238 | 0.000173994806546197 | 0.000154236945736063 | 0.0001405536844283 | 0.000162218386921242 | 0.000154210942305518 | 0.000166435652382897 | 0.000174840212159914 | 0.000150291981122399 | 0.000165035041342118 | 0.000156416094221404 |
| pos\_2270 | (2R,2'S)-Isobuteine | 0.000230356624575012 | 0.000366458523292283 | 0.000191724185089291 | 0.000344055167820525 | 0.000274401403179552 | 0.000265057787993706 | 0.00028258137630601 | 0.000240681980797369 | 0.000320353275109393 | 0.000266164451146599 | 0.000264241523930719 | 0.000217041074729733 |
| pos\_2272 | Oleoside dimethyl ester | 2.80496060952016e-05 | 2.75346010918162e-05 | 2.70125798279689e-05 | 2.5127111756632e-05 | 3.02116839517411e-05 | 3.49434397215638e-05 | 3.08075342879977e-05 | 2.81012540153978e-05 | 3.39336735193507e-05 | 2.49075304917241e-05 | 2.96434275299101e-05 | 2.74050033330889e-05 |
| pos\_2273 | Diethylenetriamine crosslinked with epichlorohydrin | 0.000181201431838275 | 0.000110439179503455 | 3.35835456720649e-05 | 5.83452215465452e-05 | 3.59582502166632e-05 | 6.33392064683269e-05 | 4.2373193488739e-05 | 3.9614783348699e-05 | 2.86491441198458e-05 | 7.1546301221552e-05 | 4.43163964665231e-05 | 4.21286546890207e-05 |
| pos\_2274 | N-Acetylornithine | 3.14257269830395e-05 | 2.41011738039024e-05 | 5.05498925459483e-06 | 1.61254220922417e-05 | 2.66840875361516e-05 | 1.94563814718067e-05 | 1.10519958658337e-05 | 8.85790573213916e-06 | 1.04886197056703e-05 | 2.34160022661326e-05 | 1.0773874868667e-05 | 5.52338248480165e-06 |
| pos\_2277 | TG(15:0/18:4(6Z,9Z,12Z,15Z)/o-18:0) | 0.000141091311346479 | 0.000200980008585033 | 0.000207660002618156 | 0.000175885561217529 | 0.000151255333889612 | 0.000191427369543602 | 0.000164775877387715 | 0.000186631330930068 | 0.000205072951492111 | 0.000166362528615165 | 0.000182892682958729 | 0.000177732787977145 |
| pos\_2278 | Isozeaxanthin | 0.00108971721388233 | 0.00148354040617544 | 0.00131468242721586 | 0.00104737745289627 | 0.000910622592568717 | 0.0011731087393779 | 0.000867398317997638 | 0.00110458795557653 | 0.00101055225928873 | 0.00114603276055555 | 0.00115358992714499 | 0.0010755187022257 |
| pos\_2279 | Hydrocinnamic acid | 5.81248732716293e-05 | 6.2767069501219e-05 | 6.64228527851484e-05 | 6.26322847173692e-05 | 5.55356607066907e-05 | 6.03273088319992e-05 | 5.13611861639321e-05 | 5.8861226482394e-05 | 6.66542087076351e-05 | 7.75937490192712e-05 | 6.39995480511805e-05 | 6.31996316704585e-05 |
| pos\_228 | 4-Methylaminobutanal | 7.23469654492012e-06 | 8.59619798579205e-06 | 9.86350273651753e-06 | 9.81470557218437e-06 | 2.58477330694062e-05 | 1.30251659653662e-05 | 6.56154667195006e-06 | 8.39262505360316e-06 | 3.47432043705269e-05 | 7.51692470566269e-06 | 1.51624501354244e-05 | 8.06440603255518e-06 |
| pos\_2280 | 3-(Acetyloxy)-2-hydroxypropyl octadecanoate | 8.34568719483747e-05 | 9.23945627305991e-05 | 7.52077992612186e-05 | 9.23350286377286e-05 | 8.62690083313899e-05 | 8.55313491336415e-05 | 9.38895574334716e-05 | 0.000103255978579377 | 8.81521630564025e-05 | 9.92771469784058e-05 | 0.000101094981640989 | 0.000125463319465811 |
| pos\_2281 | Meloside L | 0.000411153390841209 | 8.60504338370998e-05 | 9.48111938199012e-05 | 6.09038771958646e-05 | 0.000232756044162218 | 0.000219152441352522 | 0.000192851758132416 | 0.000167965949334359 | 0.000127340121308452 | 0.00014850259136256 | 0.000159751352134288 | 0.000135816988830162 |
| pos\_2282 | N-Acetyl-7-O-acetylneuraminic acid | 1.59736204705e-05 | 2.45385115208437e-05 | 8.59615881149103e-06 | 2.30226517823061e-05 | 1.25237309542043e-05 | 1.3779893252429e-05 | 1.25205342925632e-05 | 1.2122672046224e-05 | 1.44056179636113e-05 | 2.81953734666622e-05 | 1.28877082864913e-05 | 1.31284559487604e-05 |
| pos\_2283 | Citalopram aldehyde | 1.76710638055784e-05 | 1.04077311584894e-05 | 6.12961937270168e-06 | 7.27794845902033e-06 | 1.14045190594602e-05 | 9.09010493911645e-06 | 1.04957828442529e-05 | 9.23438270976285e-06 | 9.74627580387215e-06 | 6.91158317343185e-06 | 9.09828003335829e-06 | 9.63962350314723e-06 |
| pos\_2285 | Clozapine glucuronide | 0.000321073426475373 | 0.000339598685620814 | 0.000287843375567507 | 0.000263953557915958 | 0.000337741011266455 | 0.000349004853820895 | 0.000322283074847152 | 0.000306322200952765 | 0.000357086488179215 | 0.000266744188846357 | 0.000318619874390546 | 0.000407588122932162 |
| pos\_2286 | Clofibrate | 2.33979822661598e-05 | 3.22918320078475e-05 | 4.03792545574528e-05 | 3.02183448562528e-05 | 1.97570340419361e-05 | 3.19575668539953e-05 | 2.92529217269304e-05 | 2.86551860942139e-05 | 3.35713849346409e-05 | 3.04881137009767e-05 | 3.12977016911731e-05 | 2.64487534070901e-05 |
| pos\_2288 | PE(24:1(15Z)/20:5(5Z,8Z,11Z,14Z,17Z)) | 0.000477142137136997 | 0.000191357854355168 | 0.000184367704214032 | 0.000172258797673973 | 0.000229847379754405 | 0.000231229563295129 | 0.000259147245654343 | 0.000218960294669881 | 0.000202646863121751 | 0.000208694872469638 | 0.00021461256458898 | 0.000209786096477222 |
| pos\_229 | Lactarazulene | 1.83461499238835e-05 | 2.3904700977618e-05 | 2.87105994439566e-05 | 2.83512192549827e-05 | 2.26481723978056e-05 | 2.54590390492624e-05 | 2.10363688794316e-05 | 2.52686037957727e-05 | 2.16752654901423e-05 | 1.99911309115207e-05 | 2.31493730239486e-05 | 2.90770254515131e-05 |
| pos\_2292 | DG(18:2(9Z,12Z)/22:5(4Z,7Z,10Z,13Z,16Z)/0:0) | 0.000185478622966706 | 0.000110690998664448 | 7.58645463079893e-05 | 0.000145405080803264 | 8.29053033697613e-05 | 0.000132760060340468 | 0.000651686846987198 | 0.000358869341225656 | 7.3847658629377e-05 | 0.000100663904500502 | 0.000267016585653678 | 2.96201652928862e-06 |
| pos\_2293 | Ganglioside GM3 (d18:0/14:0) | 1.95469888525595e-06 | 3.870862549684e-06 | 1.29945847513896e-05 | 3.09974603473294e-06 | 9.44316851058118e-06 | 7.08722107827583e-06 | 0 | 3.54259514520193e-06 | 3.5031437361142e-07 | 2.08102465929921e-05 | 9.88976754617904e-06 | 5.26423191687025e-06 |
| pos\_2296 | Cheirotoxol | 1.43585525733902e-05 | 1.17982282243834e-05 | 9.76298367728436e-06 | 3.17688663229343e-06 | 0 | 7.78096854798695e-06 | 8.4733686661485e-06 | 4.29789065777195e-06 | 6.08637417075597e-06 | 1.97515525678807e-06 | 4.4380289296004e-06 | 8.70712264057576e-06 |
| pos\_2297 | 14-hydroxy-E4-neuroprostane | 0.000182720787055515 | 5.98736021545291e-05 | 5.14084073640743e-05 | 5.01210357395921e-05 | 9.68922701540365e-05 | 9.23517708819243e-05 | 9.9641246159141e-05 | 7.12032548905456e-05 | 6.73725226493991e-05 | 7.12330779632968e-05 | 7.57393025851182e-05 | 7.44592587044907e-05 |
| pos\_2298 | 11beta,20-Dihydroxy-3-oxopregn-4-en-21-oic acid | 7.43944194919758e-05 | 5.46642110736462e-05 | 0.000284910902747502 | 3.5541653694895e-05 | 3.519719951856e-05 | 8.77038132220333e-05 | 2.24685578795922e-05 | 0.000100059929253961 | 2.82440109276578e-05 | 0.000168808084346057 | 0.000114897228183032 | 0.000252909702253356 |
| pos\_2299 | Leucodelphinidin 3-[galactosyl-(1->4)-glucoside] | 1.54255667444901e-05 | 3.3856995248458e-05 | 2.1521995214458e-05 | 1.89933967408921e-05 | 2.4959821117109e-05 | 2.01509588043306e-05 | 1.70987737601672e-05 | 2.46504629192701e-05 | 3.64635194178137e-05 | 2.28798016263603e-05 | 2.39965683703582e-05 | 1.47691303755766e-05 |
| pos\_23 | 6β-Naltrexol | 0.000196971721120794 | 0.000215853390303298 | 0.000422404664055866 | 0.00113268448766104 | 0.000163108475040866 | 0.00038045657851462 | 0.000261966269511697 | 0.000162231054223353 | 9.36450564494191e-05 | 0.000422031050568223 | 0.000189917675551955 | 9.43766336257728e-05 |
| pos\_230 | Anofinic acid | 9.08015486408707e-05 | 0.000129999435857698 | 0.000136243897656403 | 9.73006410675159e-05 | 7.99642802522101e-05 | 0.000107798218218954 | 9.48109797196352e-05 | 0.000103936582152315 | 0.000115834138156289 | 0.000101884301434581 | 0.000105359121819433 | 9.25880975710823e-05 |
| pos\_2300 | Neuraminic acid | 3.80255105026747e-05 | 3.47863591731192e-05 | 3.52930689705235e-05 | 2.99069528854131e-05 | 3.87857778729762e-05 | 3.12543659224743e-05 | 3.76861686781216e-05 | 3.6360146553495e-05 | 2.98440390081665e-05 | 3.84904055284194e-05 | 2.74808136957038e-05 | 3.71842565063756e-05 |
| pos\_2301 | Delphinidin 3-sambubioside | 0.000610895085012235 | 0.000165723680899444 | 0.000177435064617089 | 0.000144366674056956 | 0.000369024655470436 | 0.000358909731978481 | 0.000309519646069185 | 0.000287045479128691 | 0.000222979812516962 | 0.000233255985335782 | 0.000271192241976539 | 0.000248040869100187 |
| pos\_2302 | (3S,3'S,5R,5'R,6R)-3,6-Epoxy-5,6-dihydro-3',5,8'-trihydroxy-beta,kappa-caroten-6'-one | 0.00133706919131595 | 0.00193458375080202 | 0.00117155961653433 | 0.00143775268904157 | 0.0014613465289725 | 0.00156477883797614 | 0.00143569179599051 | 0.00143802021850278 | 0.00161940231215177 | 0.00136888676038185 | 0.00144277550806379 | 0.00141114692455125 |
| pos\_2305 | PE(20:0/22:6(4Z,7Z,10Z,13Z,16Z,19Z)) | 0.000210189976393718 | 0.000238134079526735 | 0.000237092935427665 | 0.000262032704080135 | 0.000214217068604259 | 0.000228723959777915 | 0.000243620857548302 | 0.000227821089897334 | 0.000241478577416682 | 0.000236700917334726 | 0.000234197153823495 | 0.000199786276370488 |
| pos\_2307 | 2',4,4'-Trihydroxy-6'-methoxy-3',5'-diprenylchalcone | 6.06097302199224e-06 | 6.00115815101288e-06 | 1.52457057376009e-06 | 0 | 0 | 1.65833247434613e-06 | 0 | 0 | 6.47258845760221e-06 | 7.45994550353516e-07 | 0 | 0 |
| pos\_2308 | Bassic acid | 1.1283111973446e-05 | 1.31157428635466e-05 | 1.31147066173983e-05 | 1.22744926732681e-05 | 9.95974742145154e-06 | 1.14001572539083e-05 | 1.10279464645974e-05 | 1.18552260178498e-05 | 1.39905848246111e-05 | 1.10114531449386e-05 | 1.15561563773427e-05 | 9.70710894391877e-06 |
| pos\_231 | (R)-11,12,13-Trinor-1(5),6,9-guaiatrien-8-one | 0.000241688230363641 | 0.00028672908276947 | 0.000491825474237094 | 0.000135091332859086 | 0.000314546957450399 | 0.00029050187793884 | 0.000136864313572772 | 0.000250261548251779 | 0.000151974400582771 | 0.000529744085541942 | 0.000307568664447269 | 0.000373513952161016 |
| pos\_2310 | Arginyl-Hydroxyproline | 1.84060856978479e-06 | 5.26461286632021e-07 | 3.65988478431193e-06 | 1.97395539664508e-06 | 4.4482658713453e-06 | 2.53586484813494e-06 | 2.83684302256276e-06 | 1.48896089613704e-06 | 1.93736026333714e-06 | 3.12012998469056e-06 | 1.30421328012412e-06 | 1.93436383600888e-06 |
| pos\_2311 | 2''-(6-Acetylglucosyl)astragalin | 0.000339128080974653 | 9.65118936123282e-05 | 9.74845681342615e-05 | 8.28856620600628e-05 | 0.000199983821735024 | 0.000198175593504174 | 0.00018617523193516 | 0.000162154340299913 | 0.000121267424567265 | 0.000134138053861604 | 0.000148660570763368 | 0.000144103806949667 |
| pos\_2313 | Vaccinoside | 5.49940246887385e-05 | 8.19852724469025e-05 | 0.000123986614636118 | 7.2237584317503e-05 | 4.24856213015472e-05 | 7.65662460066032e-05 | 6.67002633117546e-05 | 7.28677979814315e-05 | 9.19043576447341e-05 | 7.0627289508526e-05 | 8.16442497904375e-05 | 6.2529677745883e-05 |
| pos\_2314 | Capsiate | 0.00139714716215196 | 0.000850795641605517 | 0.000604639031589556 | 0.000651147324170038 | 0.00101601875701667 | 0.00100098461253023 | 0.00105990256888942 | 0.000822080489057872 | 0.000911158518294922 | 0.000855828051546889 | 0.000869506232717264 | 0.000821093279098784 |
| pos\_2316 | 1'-O-Acetylpaxilline | 0.000162861914206007 | 0.000268282348906923 | 0.000193083446556627 | 0.000154559873564942 | 0.000190424449874108 | 0.000178352752394271 | 0.000277751087577364 | 0.000247173713543034 | 0.000162720714561746 | 0.00019251921745154 | 0.000230022562702818 | 0.000206824401143307 |
| pos\_2317 | 2-Galloyl-1,4-galactarolactone methyl ester | 6.57019797794979e-05 | 9.59767505879802e-05 | 9.21445626589482e-05 | 8.35208763538272e-05 | 6.97299692465111e-05 | 7.52897959117742e-05 | 6.31740285457349e-05 | 7.61449448434273e-05 | 9.03413903566177e-05 | 7.14659446550055e-05 | 7.38348090197468e-05 | 7.21282134656777e-05 |
| pos\_2318 | PC(24:0/P-18:1(11Z)) | 0.000171787975170819 | 8.99696766823771e-05 | 0.00042399134463385 | 0.000359567480645653 | 0.000236179720575181 | 0.000206920199390454 | 9.86379448903379e-05 | 0.000178083582754667 | 0.000204408654883913 | 5.80164957118713e-05 | 7.84723974591668e-05 | 0.000118990233356759 |
| pos\_232 | 8-Hydroxygalangin | 6.83912384499776e-05 | 0.000117059954665595 | 6.87987754203305e-05 | 0.000122302863029261 | 8.02603277829455e-05 | 9.529170947551e-05 | 7.31619966231576e-05 | 7.63747056855997e-05 | 0.000188904198945283 | 6.92257935370149e-05 | 0.000134443913998411 | 0.000124644916838896 |
| pos\_2320 | Neryl rhamnosyl-glucoside | 0.000289151133729743 | 0.0002685567943576 | 0.000192845595783422 | 0.0001524153353336 | 0.000193020256044242 | 0.000193422538263953 | 0.000183115880772781 | 0.00015830030445088 | 0.000174826871935417 | 0.000160692092536862 | 0.00017553021948364 | 0.000172285996347244 |
| pos\_2321 | Rebaudioside A | 1.74098899714332e-05 | 2.91198388397907e-05 | 3.02109878548469e-05 | 2.72295118665325e-05 | 1.51845100328356e-05 | 3.03666054377073e-05 | 1.83710452020822e-05 | 2.69232673468737e-05 | 2.81647203619472e-05 | 1.91298085356766e-05 | 2.46839481200146e-05 | 2.03644526115958e-05 |
| pos\_2322 | Sumiki's acid | 9.45619330348328e-05 | 0.000124429638416931 | 0.000114461747646748 | 9.70786912205853e-05 | 8.11388047388244e-05 | 9.56165612673457e-05 | 8.67634748419579e-05 | 9.72920464191459e-05 | 0.000110525730698488 | 9.1810967309245e-05 | 9.67527028225337e-05 | 9.27924185925843e-05 |
| pos\_2323 | Polymyxin B Sulfate | 4.94543728959299e-05 | 2.02414701750751e-05 | 1.57896681574002e-05 | 1.66665220211971e-05 | 2.61009629617459e-05 | 3.00745278495348e-05 | 3.13613974739281e-05 | 1.93624534080314e-05 | 2.0857663080981e-05 | 2.27175192634809e-05 | 2.81154745362542e-05 | 2.51377002847597e-05 |
| pos\_2326 | 19(20)-EpDPE | 0.000189557093541976 | 8.73777297718629e-05 | 0.000226864251258331 | 7.23095662648886e-05 | 8.07665319404296e-05 | 9.94589851398823e-05 | 9.12818114389569e-05 | 0.000102766871979669 | 7.80575726262909e-05 | 0.000155142895395942 | 0.000105162106255553 | 0.000128322263178335 |
| pos\_2327 | alpha-Micropteroxanthin B | 0.000368100283686745 | 0.000351243318524951 | 0.000311723578216857 | 0.000359595157956941 | 0.000364811882437993 | 0.000326871486159312 | 0.000619918454619391 | 0.000438848304314384 | 0.00045558082275077 | 0.000444978046984619 | 0.000453446053916211 | 0.000326987530007893 |
| pos\_2328 | Guanosine 3'-diphosphate 5'-triphosphate | 5.22127375199092e-06 | 0 | 0 | 7.00602347172057e-07 | 1.88489923934352e-06 | 2.41636312454555e-06 | 2.8963679894294e-06 | 3.00682139986641e-06 | 1.92467585623698e-06 | 7.17353857636619e-07 | 2.93317016185102e-06 | 1.09795174024318e-06 |
| pos\_2329 | Hoduloside VII | 4.27039827289438e-05 | 3.1722684206982e-05 | 2.5201473867592e-05 | 1.45855989254215e-05 | 1.85123214328986e-05 | 1.01318625594882e-05 | 1.23251148047795e-05 | 9.97208410346896e-06 | 1.05974930285103e-05 | 7.15365929101591e-06 | 1.47105554517998e-05 | 1.91831585197803e-05 |
| pos\_233 | Germacrene C | 0.000205387194893177 | 0.000246723276630994 | 0.000253646940421986 | 0.000217168087287404 | 0.000182497153153431 | 0.000212589729925899 | 0.00019567839787246 | 0.000244932411064279 | 0.000230553298253078 | 0.000224951045274426 | 0.000225329802307909 | 0.00021269506507874 |
| pos\_2330 | 7,8-Dihydro-7-hydroxy-8-S-glutathionyl-benzo[a]pyrene | 0.000152812366140304 | 4.12982804616924e-05 | 4.63921377182018e-05 | 3.46884616738934e-05 | 9.3761076228894e-05 | 8.95794219986877e-05 | 8.15970981663545e-05 | 7.28739470065598e-05 | 5.7309328630993e-05 | 5.93958590431833e-05 | 6.89913619874362e-05 | 6.39765541654024e-05 |
| pos\_2332 | Enkephalin L | 2.15104065355844e-05 | 1.89554642636277e-05 | 1.87900289686271e-05 | 1.79702222573245e-05 | 1.69793380215273e-05 | 2.2057077003167e-05 | 1.95514531868853e-05 | 4.69561852101513e-05 | 1.56369474734598e-05 | 2.67298138582658e-05 | 5.96111627335851e-05 | 1.32624265426325e-05 |
| pos\_2334 | N2-Fructopyranosylarginine | 9.95263523375336e-05 | 0.000112262154850432 | 9.10968213839375e-05 | 0.000101484491758076 | 8.28172616235878e-05 | 7.16902436151825e-05 | 7.51361919432124e-05 | 9.93464732308828e-05 | 8.75412424177915e-05 | 7.12443565532275e-05 | 9.63854380947206e-05 | 0.000132932302381255 |
| pos\_2337 | 20-dihydroxyleukotriene B4 | 0.000217336733455198 | 0.000264117085481984 | 0.000242188439951855 | 0.000196862902980356 | 0.000207323946927564 | 0.000268569642600063 | 0.00021296632765557 | 0.000232893464485687 | 0.000274808271170358 | 0.00026299592127613 | 0.000239098833641428 | 0.000228683303929826 |
| pos\_2338 | 10,13-epoxy-11,12-dimethyloctadeca-10,12-dienoic acid | 9.72454053712066e-05 | 9.1850122817622e-05 | 0.000118832442968965 | 0.000124336159083111 | 0.000163930957355917 | 0.000119080020548657 | 0.000100349075290403 | 0.000109320473628195 | 0.000127943813558121 | 0.000162097888218942 | 0.00012235094734422 | 0.000127403026184956 |
| pos\_2339 | Catechin 3',5-diglucoside | 3.36663435082313e-06 | 1.1101901745821e-05 | 1.02972308996771e-05 | 1.1675506468171e-05 | 4.50392061465578e-06 | 8.87924902355009e-06 | 2.19901734759324e-05 | 6.98394512962993e-06 | 2.22236098715327e-05 | 9.09706219838129e-06 | 1.75038428368262e-05 | 1.85099037683498e-05 |
| pos\_234 | METHYL ORSELLINATE | 4.48645274109389e-05 | 0.000110252155311197 | 2.01981510666445e-05 | 5.89177868301576e-05 | 5.08237992204618e-05 | 5.55692343696803e-05 | 5.64242896193137e-05 | 3.8960410848429e-05 | 4.31316204211024e-05 | 3.68104916153013e-05 | 4.24093594326669e-05 | 2.29270898199497e-05 |
| pos\_2340 | 2-[4-(3-Hydroxypropyl)-2-methoxyphenoxy]-1,3-propanediol 1-xyloside | 0.000135974139443774 | 0.000132224712395954 | 0.000134589256907584 | 0.000106627063737616 | 0.000133482488793866 | 0.000120605471243034 | 0.000145909778605739 | 0.000127697072440747 | 0.000109866187396225 | 0.000149247213087234 | 0.000106384808136613 | 0.000129706017413007 |
| pos\_2341 | PE(15:0/16:1(9Z)) | 0.00110885765190872 | 0.00150738587914478 | 0.00213941310909843 | 0.00157648751227705 | 0.00102296279860815 | 0.00146330117127722 | 0.00114489808181324 | 0.00154091988244958 | 0.00142426237086143 | 0.00113123515050266 | 0.0014992135052734 | 0.00127076975131841 |
| pos\_2342 | 2,4,12-Octadecatrienoic acid piperidide | 3.99319970557736e-06 | 5.98480693828869e-06 | 6.20510513416461e-06 | 6.9591539307669e-06 | 3.82730122232251e-06 | 4.90780461173879e-06 | 4.21000978059472e-06 | 5.13433720425171e-06 | 4.3145032815227e-06 | 3.4194258519957e-06 | 5.20339874602995e-06 | 0.000135579683907791 |
| pos\_2345 | Methyl (3x,4E,10R)-3,10-dihydroxy-4,11-dodecadiene-6,8-diynoate 10-glucoside | 1.06945947376637e-05 | 1.72582121396828e-05 | 4.79617026094125e-06 | 1.00300149316173e-05 | 1.08755142738873e-05 | 9.95225602172339e-06 | 9.19889739382473e-06 | 9.15692187087346e-06 | 9.23074609050094e-06 | 1.16880795537223e-05 | 7.20781917949039e-06 | 5.13389169294181e-06 |
| pos\_2348 | 2,3-Dihydro-5-(3-hydroxypropanoyl)-1H-pyrrolizine | 5.29817859063337e-05 | 6.35563913414633e-05 | 6.66409090499753e-05 | 6.92578729147482e-05 | 4.77140389908234e-05 | 6.46609502505191e-05 | 5.5663964809694e-05 | 6.53239600145263e-05 | 5.54454539291349e-05 | 5.49379714412444e-05 | 6.29150715876442e-05 | 6.34952142772898e-05 |
| pos\_2349 | Oxprenolol | 3.3147722326525e-05 | 1.25951135571647e-05 | 8.82526336963914e-05 | 2.97193657548255e-05 | 4.37920846628552e-05 | 0.000291195468765919 | 7.36380367868607e-05 | 8.25708126733939e-05 | 0.000108625501513186 | 0.000164488708114983 | 6.84009762200424e-05 | 5.15755076911009e-05 |
| pos\_235 | 17beta-Hydroxy-2-oxa-5alpha-androstan-3-one | 0.000136574954397291 | 0.000318836426541789 | 0.000205335897011534 | 0.000221871087901602 | 0.000158361796217731 | 0.000161233435021077 | 0.000180964025023914 | 0.000166434079503008 | 0.000169746432061933 | 0.000130602613002927 | 0.000138127483861583 | 0.000149994190100458 |
| pos\_2351 | DG(18:1(11Z)/24:0/0:0) | 0.000337916831424007 | 4.25679149155903e-05 | 5.28686468853729e-05 | 3.60530661311422e-05 | 0.000174692007944035 | 0.000155355645661895 | 0.000133572989120873 | 0.000107913224712251 | 7.08176236535908e-05 | 8.07256190468395e-05 | 9.67716512236521e-05 | 8.53742877136671e-05 |
| pos\_2352 | Goyaglycoside e | 4.59958828198363e-06 | 1.36314599300327e-06 | 8.59226588078616e-06 | 1.17708535537978e-05 | 0.000112943998734899 | 3.15906230747137e-05 | 1.0972254853018e-05 | 1.52680744737861e-05 | 1.99559360199324e-05 | 5.90125786638428e-05 | 3.21162751031969e-05 | 3.86541988739917e-05 |
| pos\_2353 | Quinestrol | 7.92067342040611e-05 | 9.08056972041855e-05 | 7.9379272585127e-05 | 7.98690709974299e-05 | 5.46051105419294e-05 | 8.26002411884215e-05 | 6.75580451050178e-05 | 8.34500965564422e-05 | 8.66671670571051e-05 | 6.33638588611179e-05 | 7.53518508000621e-05 | 7.41276480023299e-05 |
| pos\_2354 | N-[(4-Hydroxy-3-methoxyphenyl)methyl]octanamide | 0.000132875624690278 | 0.000149136115948454 | 0.000138197091710108 | 0.000134776641875588 | 0.00014129979428607 | 0.000131501201482498 | 0.000145443516771697 | 0.000122680367241143 | 0.000152371055146974 | 0.00014226685020119 | 0.00013931570664144 | 0.000137901380590394 |
| pos\_2356 | DG(16:1(9Z)/22:4(7Z,10Z,13Z,16Z)/0:0) | 3.95913933302946e-05 | 1.19362618491995e-06 | 1.91660435036542e-06 | 7.45193142829317e-07 | 1.43255559312781e-05 | 1.30240820946376e-05 | 7.79793184340857e-06 | 9.05516945028171e-06 | 3.90395707491179e-06 | 5.7080121247642e-06 | 5.27128028164032e-06 | 6.38466544868061e-06 |
| pos\_2358 | Ganglioside GM3 (d18:1/18:1(11Z)) | 5.54973444371038e-05 | 7.54297775209504e-06 | 5.14759103380473e-06 | 6.19642026861756e-06 | 2.43790312280891e-05 | 1.78118496828395e-05 | 2.26788928415443e-05 | 1.40135487995944e-05 | 9.13224049548333e-06 | 1.47627164884182e-05 | 1.23494835230578e-05 | 1.60802408436391e-05 |
| pos\_2360 | Gomphrenin II | 7.35652848509634e-06 | 1.4147784341755e-05 | 7.15132010990212e-06 | 8.1435328372779e-06 | 9.59035910461247e-06 | 9.18670832311326e-06 | 6.88933180611978e-06 | 6.12094871675329e-06 | 1.15298974406778e-05 | 1.03267340299111e-05 | 8.48362542893147e-06 | 7.02495738695491e-06 |
| pos\_2361 | 4'-Hydroxy-5,7-dimethoxyflavan | 2.77147469639568e-05 | 4.16796115142559e-05 | 2.15427552225039e-05 | 3.59908350968079e-05 | 3.70357481584773e-05 | 3.12237516115649e-05 | 3.66222469940795e-05 | 3.60759467519962e-05 | 3.36373557907755e-05 | 3.95207513263569e-05 | 3.47627450696247e-05 | 3.5176202211265e-05 |
| pos\_2362 | 2-Amino-3-carboxymuconic acid semialdehyde | 6.50419270924965e-06 | 4.04635608163833e-06 | 3.94282820571273e-06 | 4.17005848257969e-06 | 5.07245800359582e-06 | 4.00924496683539e-06 | 5.25647971247537e-06 | 4.9545655489342e-06 | 5.49922530980779e-06 | 3.54715101641098e-06 | 5.62879578065737e-06 | 1.08324615776793e-05 |
| pos\_2363 | Lacto-N-biose I | 6.98835967882063e-05 | 0.000135412860949076 | 6.91607327819511e-05 | 0.000141191629545234 | 7.6666067382952e-05 | 6.90349696884902e-05 | 6.93051040679194e-05 | 0.000118780651834293 | 0.000110533557456827 | 0.000130243818694702 | 8.10166711798327e-05 | 8.30212353636031e-05 |
| pos\_2364 | Allixin | 0.000958277683086518 | 0.000999872944612255 | 0.000946474281863445 | 0.000885938082536561 | 0.000773943903363754 | 0.000904990136460836 | 0.000872027986558194 | 0.000931366685722556 | 0.000968639225447638 | 0.000850356820880262 | 0.000912123724695597 | 0.000913072751119389 |
| pos\_2365 | 3-Hydroxynonyl acetate | 0.000825387718324878 | 0.000548183162712368 | 0.00110115336295095 | 0.00323430767560947 | 0.00234711526184988 | 0.00164097386649371 | 0.00281559653719966 | 0.00174752122358735 | 0.000534583554775372 | 0.000537875871985338 | 0.00133603358940991 | 0.000521985443322247 |
| pos\_2366 | (Cyclohexylmethyl)pyrazine | 7.59448384930621e-05 | 9.36683498561704e-05 | 6.85876168521065e-05 | 8.98007967737822e-05 | 6.76700161372892e-05 | 7.23247509448201e-05 | 6.41839636828368e-05 | 7.36158078669565e-05 | 7.03802184086331e-05 | 6.26910019320541e-05 | 6.66719797222162e-05 | 8.17478116108622e-05 |
| pos\_2367 | Cyclopassifloside V | 0.00135780036812065 | 0.00083404755449229 | 0.000829082805634884 | 0.000696988703729157 | 0.000793655567246409 | 0.000845499094161949 | 0.000870646490533344 | 0.000878298840333095 | 0.000885022992124957 | 0.000755348700688269 | 0.000811305023031306 | 0.000772183040592241 |
| pos\_2368 | N-(2R-Hydroxyhexadecanoyl)-2S-amino-9-methyl-4E,8E-octadecadiene-1,3R-diol | 0.00030403652858651 | 7.67169635801132e-05 | 9.20981417201493e-05 | 7.07493425682574e-05 | 0.000185346436045112 | 0.000177744750360485 | 0.000167348804343251 | 0.000143660086977495 | 0.000107380024535701 | 0.000123477629452332 | 0.000137634347967197 | 0.000125180692472058 |
| pos\_2369 | DG(22:1(13Z)/22:5(7Z,10Z,13Z,16Z,19Z)/0:0) | 0.000478887526606532 | 6.30050228395459e-05 | 7.22474451112687e-05 | 5.20300948479404e-05 | 0.000235877132799705 | 0.000236941231378488 | 0.000192910169064795 | 0.000163515942806562 | 9.7046421901332e-05 | 0.000119091973789305 | 0.000159035490557299 | 0.000130768840886889 |
| pos\_2370 | Cynarasaponin F | 8.11024478207463e-05 | 0.000143067785489457 | 0.000145054379702656 | 9.80657788802937e-05 | 8.3065976540967e-05 | 0.000115849437153238 | 8.79941833075566e-05 | 0.000108800054999529 | 0.0001411538090685 | 8.78652995172454e-05 | 0.000112603549770195 | 9.1802281809478e-05 |
| pos\_2372 | Pelargonic acid | 6.02138340026811e-05 | 7.36079469649114e-05 | 7.53457208768264e-05 | 7.70775634276424e-05 | 5.19160421464917e-05 | 7.12149028151023e-05 | 6.29897201124529e-05 | 7.67588151466174e-05 | 6.8959703180926e-05 | 5.78122819697776e-05 | 7.39209640944951e-05 | 6.95555525561897e-05 |
| pos\_2373 | 6'-Methoxypolygoacetophenoside | 1.47394318304597e-06 | 2.72473657777219e-06 | 6.32208103774947e-06 | 0.000134523997638246 | 0.000108193619217767 | 4.81499632991071e-05 | 0.000123171613807175 | 6.1408485751463e-05 | 0 | 6.63566807728104e-06 | 4.02251864718044e-05 | 5.08187192953212e-06 |
| pos\_2375 | N-Acetyl-6-O-L-fucosyl-D-glucosamine | 2.36662513724694e-05 | 3.0625927810392e-05 | 1.03779091775046e-05 | 1.581914660579e-05 | 1.69591165103255e-05 | 1.4892337438295e-05 | 1.6984275800607e-05 | 2.06617930218498e-05 | 1.59941317542951e-05 | 1.57123438833654e-05 | 1.55386535877236e-05 | 2.1979712564529e-05 |
| pos\_2376 | N-Deisopropyl-fluvastatin | 6.4091905421325e-05 | 7.81714511367182e-05 | 3.72685078039671e-05 | 6.86895085266384e-05 | 5.305041680768e-05 | 5.07343434296194e-05 | 4.51019749042049e-05 | 4.49126557730878e-05 | 5.1677316918423e-05 | 7.23920947797718e-05 | 5.14511268815484e-05 | 7.18062535365974e-05 |
| pos\_2377 | Glutamate, gamma-methyl ester | 2.77915772823481e-05 | 3.83992449909033e-05 | 5.33779478891249e-05 | 3.99519198308146e-05 | 3.15632337839323e-05 | 3.78632705628757e-05 | 4.88533377318759e-05 | 4.19861912940036e-05 | 3.33158465639292e-05 | 3.5424905235057e-05 | 3.92402251650082e-05 | 4.28342974564714e-05 |
| pos\_2378 | Tangeraxanthin | 0.00513606779463539 | 0.00187626227242738 | 0.00190914668504251 | 0.00159562105571703 | 0.0023186961491191 | 0.00237294897396803 | 0.00257873706832193 | 0.00223227462516075 | 0.00209171336865072 | 0.00205127826289518 | 0.00225267253222958 | 0.00228069014500702 |
| pos\_2379 | 3-Methylcyclohexanol | 3.86090007700794e-05 | 6.06849623394355e-05 | 5.2081928453725e-05 | 6.29019050592845e-05 | 4.87082989473051e-05 | 4.76563194814349e-05 | 3.52256443618118e-05 | 4.55641017850022e-05 | 5.58438406293181e-05 | 4.78683307415893e-05 | 5.45242453017078e-05 | 5.12302364228269e-05 |
| pos\_238 | (R)-Apiumetin | 7.49905239091231e-06 | 3.29811045074954e-06 | 2.54863049081412e-06 | 0.000144187337163334 | 2.48408012305789e-07 | 7.58702873830038e-06 | 5.30559522759072e-06 | 4.32777241851711e-06 | 3.17453142050703e-06 | 3.39924663913297e-06 | 3.09253375819861e-06 | 1.62531970811098e-05 |
| pos\_2380 | Chloroquine | 0.000196445854013536 | 0.000173234922433091 | 0.000173057162306537 | 0.00114264216410713 | 0.000140947363789885 | 0.000416811073913203 | 0.000161448824167207 | 0.000163308708926911 | 0.000154732276685466 | 0.000163926683305467 | 0.00016222789977218 | 0.000156879300446923 |
| pos\_2382 | MG(18:0e/0:0/0:0) | 0.000345417891112186 | 0.000319048204693631 | 0.000259158519364071 | 0.000305746442932563 | 0.00036301841437384 | 0.000324183769933264 | 0.000318351450198052 | 0.00028842465949542 | 0.000350585119221804 | 0.000319065527099413 | 0.000320872390910453 | 0.000271272094493671 |
| pos\_2383 | TG(22:6(4Z,7Z,10Z,13Z,16Z,19Z)/22:5(7Z,10Z,13Z,16Z,19Z)/22:6(4Z,7Z,10Z,13Z,16Z,19Z))[iso3] | 0.00139079631537972 | 0.000852563326016087 | 0.000772423297552756 | 0.000684649289127126 | 0.000778114138578578 | 0.000798869526314543 | 0.000858400604162433 | 0.000817564325589787 | 0.000803146426334236 | 0.000775952436173985 | 0.00077000941018699 | 0.000753761910546719 |
| pos\_2384 | Ticarcillin | 2.85814240986e-05 | 5.01070214202047e-05 | 3.3212091944228e-05 | 3.50213504874462e-05 | 4.24563147268801e-05 | 3.52112867499924e-05 | 2.54109879645517e-05 | 2.99419971519163e-05 | 5.74845482190082e-05 | 3.56303454938445e-05 | 3.7320528528433e-05 | 3.44991840703467e-05 |
| pos\_2385 | Artobiloxanthone | 4.97814755321638e-05 | 6.88025958260558e-05 | 9.75112337616202e-06 | 4.35970758556301e-05 | 2.57369090188838e-05 | 3.4031858908755e-05 | 2.88875945957296e-05 | 2.01753071805059e-05 | 5.85907123942699e-06 | 4.79146386418179e-05 | 2.71044452031417e-05 | 1.46227215459085e-05 |
| pos\_2387 | Tuberoside B (Ullucus tuberosus) | 1.09136011484554e-05 | 1.12959557842661e-05 | 1.03967455059518e-05 | 6.59483566313821e-06 | 9.16213535400378e-06 | 1.30384833657619e-05 | 1.18728230447247e-05 | 1.06399244980393e-05 | 1.21943436023697e-05 | 8.77101706746228e-06 | 9.6233471337923e-06 | 8.28742100465696e-06 |
| pos\_2388 | Omega-Carboxy-trinor-leukotriene B4 | 5.63180481419547e-05 | 6.68987874057057e-05 | 6.24494930929475e-05 | 5.67611251367099e-05 | 7.58033909396618e-05 | 5.45547848160515e-05 | 0.000147526780909741 | 0.000109232925816315 | 8.83317074936292e-05 | 9.54412918124529e-05 | 8.65664332553675e-05 | 8.92508223399601e-05 |
| pos\_2389 | Aframodial | 3.92673517754288e-05 | 3.42682552337498e-05 | 8.98784970002413e-05 | 1.07473454893288e-05 | 4.97297470588419e-05 | 4.46379031652351e-05 | 2.96122152014469e-06 | 3.27533148538371e-05 | 6.53516203681906e-06 | 0.000119904743673666 | 4.53976605161347e-05 | 6.23175371054809e-05 |
| pos\_239 | 3-(2,4-Cyclopentadien-1-ylidene)-5alpha-androstan-17beta-ol | 0.0013214890170955 | 0.00122251668029481 | 0.00286268325856734 | 0.00257802779403919 | 0.00322641847412911 | 0.00249853310974918 | 0.00215002029726296 | 0.00202068777796143 | 0.00294397849824682 | 0.00232874385656426 | 0.0024423746230049 | 0.00228771462421306 |
| pos\_2392 | 4Alpha-hydroxymethyl-5alpha-cholesta-8-en-3beta-ol | 0.001538462908499 | 0.00159075737392506 | 0.00159810729761374 | 0.00134562753623272 | 0.00123643583301143 | 0.00147046806592357 | 0.00165004130889447 | 0.00163957795680806 | 0.00150090529257942 | 0.00185111201167434 | 0.00164642531816513 | 0.00149401262391014 |
| pos\_2394 | 1,1,2-Trimethyl-3,5-bis(1-methylethenyl)cyclohexane | 5.40743856942161e-05 | 2.91269343470001e-05 | 4.68405060789731e-05 | 2.77474169757399e-05 | 3.22028382026003e-05 | 4.68479506495799e-05 | 2.66224682270602e-05 | 4.63261193339308e-05 | 2.66442562214238e-05 | 3.70837547171611e-05 | 3.85475528426171e-05 | 3.53131949877253e-05 |
| pos\_2395 | S-adenosyl-L-methioninate | 3.5551676172132e-07 | 1.6704949803189e-07 | 7.77726400864349e-06 | 1.93490966756571e-07 | 5.93896078310064e-06 | 2.72959386393825e-06 | 6.43388826116455e-08 | 6.56298279145969e-07 | 2.78396700780416e-07 | 7.93425257478883e-06 | 2.99760119791676e-06 | 5.02318284092798e-06 |
| pos\_2396 | 3,3',7-Trihydroxy-4'-methoxyflavone | 9.66173402228825e-06 | 2.02413533725463e-05 | 6.30005255696863e-06 | 1.41207680842798e-05 | 2.76318178022312e-05 | 1.52446437733585e-05 | 9.63785992122141e-06 | 8.84030461217182e-06 | 1.47452347796082e-05 | 2.30829373816506e-05 | 1.25752861503894e-05 | 6.88449985918931e-06 |
| pos\_2397 | Lupeoside | 0.000244755469047869 | 8.57011979889575e-05 | 8.00989324364577e-05 | 6.50958329037023e-05 | 0.000137878486096303 | 0.000133375972079352 | 0.000135039580418837 | 0.000135731290984211 | 9.39334193343628e-05 | 0.000108497077713722 | 0.000120294933485124 | 0.000141509783109327 |
| pos\_2398 | Phentolamine | 0.000526232727623883 | 0.00022175066077204 | 0.000216426804663497 | 0.000187980026739692 | 0.000343844702729502 | 0.000363060794728724 | 0.000373882018759382 | 0.00029868082698016 | 0.000275797185917896 | 0.000275687910152934 | 0.000297654972788203 | 0.000301124093523725 |
| pos\_24 | Pregeijerene | 4.31967143397912e-05 | 5.55166578276255e-05 | 5.05025760045397e-05 | 5.37555088136287e-05 | 4.98126572049303e-05 | 5.05983479018331e-05 | 5.81023453254669e-05 | 5.44401940073126e-05 | 5.44208877616471e-05 | 5.20064894876656e-05 | 5.30476721333883e-05 | 5.81091827695296e-05 |
| pos\_240 | 11,11-Difluoro-9Z-dodecenyl acetate | 4.17752569596032e-05 | 3.48029976660336e-05 | 2.90679921402125e-05 | 3.55328318826888e-05 | 3.5372726564161e-05 | 3.21342246271601e-05 | 3.28684566550092e-05 | 3.28366270411349e-05 | 3.84365576752243e-05 | 2.88632723457427e-05 | 3.41297318832712e-05 | 3.44868251584377e-05 |
| pos\_2405 | Mulberrofuran T | 0.000311368122981849 | 1.44495754348593e-05 | 1.9796735172266e-05 | 1.21977302468597e-05 | 0.000146072445130955 | 0.000135340100935325 | 0.000107137757660796 | 7.62504859118458e-05 | 2.68326812446694e-05 | 5.11825126588183e-05 | 4.91543764929227e-05 | 4.43915737513203e-05 |
| pos\_2406 | P1,P4-Bis(5'-uridyl) tetraphosphate | 0.00010446950010812 | 5.66198303312269e-05 | 4.96254067634599e-05 | 4.51193897593529e-05 | 5.64403830866036e-05 | 6.67103980964479e-05 | 6.68642823816414e-05 | 5.93952781755706e-05 | 5.90587134688033e-05 | 5.53434062843376e-05 | 5.4086177421034e-05 | 5.87351524612905e-05 |
| pos\_2407 | Edultin | 1.68557597617705e-05 | 1.19739504582608e-05 | 7.23852289289557e-06 | 3.17766480874798e-05 | 3.66871186488959e-06 | 1.63318644811377e-05 | 2.40273446233938e-05 | 3.27740086910142e-06 | 1.40622079457053e-05 | 2.08491263641502e-05 | 7.76361124300033e-06 | 1.92382507897198e-05 |
| pos\_2409 | CDP-glucose | 5.56081603182163e-06 | 2.9839235410234e-06 | 2.13876141608214e-06 | 2.04420176027783e-06 | 6.61044740479185e-06 | 2.49732141040081e-06 | 7.2016183973862e-06 | 4.33740336468357e-06 | 2.9548219999843e-06 | 1.64860651512288e-06 | 2.71405472991867e-06 | 8.55228988014647e-07 |
| pos\_241 | Pyrocurzerenone | 2.71072746101918e-05 | 3.08937043501573e-05 | 3.83068435416126e-05 | 5.93574746242763e-05 | 0.000126922532891998 | 7.28692027479819e-05 | 4.52396689678178e-05 | 6.62348804012264e-05 | 5.15530655349433e-05 | 8.68880447616813e-05 | 7.66468982420012e-05 | 9.67073821836055e-05 |
| pos\_2410 | Sulindac sulfone | 7.45564521350911e-05 | 0.000103125132044607 | 0.000102099081206779 | 8.12851964141011e-05 | 6.81772107519798e-05 | 8.36126024702003e-05 | 8.18993357424875e-05 | 9.00691736488847e-05 | 9.44763799035613e-05 | 7.36349943825033e-05 | 8.66355672109073e-05 | 7.81194000310232e-05 |
| pos\_2411 | 3'-(2'',3''-Digalloylglucosyl)-phloroacetophenone | 1.98373909264664e-05 | 2.9936141154856e-05 | 2.43007499445076e-05 | 2.31684338613619e-05 | 1.72474043849342e-05 | 2.16457810227971e-05 | 2.06179067568542e-05 | 3.03049357021501e-05 | 2.47135316597702e-05 | 2.25649187508779e-05 | 2.4982428830539e-05 | 2.40903830548644e-05 |
| pos\_2412 | Trihexosylceramide (d18:1/9Z-18:1) | 8.36034418547715e-05 | 1.49647892874268e-05 | 1.68133169421209e-05 | 9.1696357000602e-06 | 5.32685663320589e-05 | 4.62995138495669e-05 | 4.13263349391777e-05 | 3.0599681297352e-05 | 2.15551949969199e-05 | 2.84942925204119e-05 | 3.36344785305833e-05 | 2.91249392596495e-05 |
| pos\_2413 | 5-Hydroxyindoleacetaldehyde | 3.28399889458844e-07 | 9.48620136244056e-07 | 3.55554902916474e-07 | 9.27545345695235e-08 | 5.47456611866807e-07 | 4.55980551108682e-07 | 4.04529890790192e-07 | 8.24978903659588e-07 | 3.08458224751874e-07 | 7.91101751457911e-07 | 9.50465432834025e-07 | 6.25057307880283e-07 |
| pos\_2414 | Artocarbene | 4.61441479038178e-06 | 9.71816038277922e-06 | 2.16790563417465e-06 | 6.17231960452608e-06 | 7.52966451250694e-06 | 4.05943893852208e-06 | 5.21375200513414e-06 | 3.21339948673331e-06 | 7.38307397751444e-06 | 6.6165753117257e-06 | 3.22543371693969e-06 | 4.237908433804e-06 |
| pos\_2415 | 3-Hydroxy-6,8-dimethoxy-7(11)-eremophilen-12,8-olide | 7.07718865316573e-06 | 5.10566261535506e-06 | 1.63924300263969e-06 | 2.748594046883e-06 | 5.7431712556681e-06 | 4.59965310127439e-06 | 4.42091039154591e-06 | 3.76633086883453e-06 | 2.86963864900982e-06 | 4.17830831295956e-06 | 4.54801690791791e-06 | 3.26566947620857e-06 |
| pos\_2416 | Marmesin galactoside | 6.25859549010847e-06 | 0 | 7.79459827912168e-07 | 6.03243766281136e-07 | 3.42889188946298e-06 | 2.83591669939626e-06 | 3.25520687532569e-06 | 1.76385467177361e-06 | 1.13320316424919e-06 | 1.4792734889386e-06 | 2.7792392174194e-06 | 1.82580186988464e-06 |
| pos\_2417 | Vinaginsenoside R13 | 0.00128748432436443 | 0.000708915314438539 | 0.000668760828074267 | 0.000566656004453609 | 0.000649980817113767 | 0.000702524103824013 | 0.000727590317560905 | 0.000702559202184105 | 0.000690474507631154 | 0.000660152258322872 | 0.000717017688463927 | 0.000670554452847568 |
| pos\_2419 | TG(10:0/10:0/8:0) | 0.00158734542015543 | 0.00193753642792667 | 0.00160041954786175 | 0.00164832633525209 | 0.00138197526511277 | 0.00173111324402182 | 0.00176778067393635 | 0.00172059398214526 | 0.00182698053030561 | 0.001730480901923 | 0.00185052111208561 | 0.00152945823461664 |
| pos\_242 | Sudan Brown RR | 5.34829926283691e-05 | 0.000109362402785721 | 5.81909013691973e-05 | 4.58607586560228e-05 | 2.94429112628529e-05 | 6.16058516606609e-05 | 6.03709374451336e-05 | 3.70312166098778e-05 | 8.44789337788343e-05 | 7.91524511483741e-05 | 6.38690192055106e-05 | 2.6487624640517e-05 |
| pos\_2420 | 4-Methoxybenzyl O-(2-sulfoglucoside) | 0 | 1.08131334131202e-06 | 2.30734425740459e-06 | 4.48829355736841e-06 | 9.77260329314577e-06 | 3.8062810071973e-06 | 6.47852893478406e-05 | 4.07154150923019e-05 | 8.69662861344325e-06 | 8.4983775077695e-07 | 2.65402726392721e-05 | 1.02486609514875e-05 |
| pos\_2422 | Ganglioside GM3 (d18:0/18:0) | 2.95903138831715e-05 | 2.06694889342525e-05 | 2.1731514321488e-05 | 1.831990667644e-05 | 2.01485194598694e-05 | 2.03710931770755e-05 | 2.12000752205269e-05 | 2.45460785660811e-05 | 1.78474280027175e-05 | 2.11184711414607e-05 | 1.91268046065604e-05 | 1.91512191365454e-05 |
| pos\_2423 | (6R\*,8S\*)-6,8-Tetratriacontanediol | 0.000292200051505082 | 0.000412994932838689 | 0.00034750460415048 | 0.000326861564277756 | 0.000236960737922245 | 0.000338319660803282 | 0.000422552351438772 | 0.000379978645912324 | 0.000517574250566844 | 0.000325550093266297 | 0.00035628388496056 | 0.000322614898165514 |
| pos\_2424 | Asticolorin A | 0.00022200824454767 | 0.000110032272093294 | 0.000130773360959771 | 0.000396919085982993 | 0 | 0.000166388811034595 | 1.46853211373573e-06 | 7.67475489074302e-05 | 3.89496765928934e-05 | 1.93946737512001e-05 | 4.81758053308387e-05 | 0.000184771027842307 |
| pos\_2425 | DG(18:0/18:3(9Z,12Z,15Z)/0:0) | 3.28675311739543e-07 | 1.66192398421635e-07 | 3.48220343075852e-07 | 3.91010887627141e-08 | 5.44665999767249e-07 | 0 | 3.57621282943055e-08 | 1.50655070360162e-07 | 1.53356041602607e-08 | 3.41898904379757e-07 | 5.97413914234528e-09 | 5.07913404682787e-07 |
| pos\_2426 | Sanguisorbin E | 0.000242829984545169 | 0.00023269492689175 | 0.000244249711606093 | 0.000161557717433389 | 0.000199455057859338 | 0.000195352861334368 | 9.75740022819709e-05 | 0.000266954239850292 | 6.37148947471215e-05 | 0.000164492805325821 | 0.00021388811127094 | 0.000533589008139883 |
| pos\_2427 | Alliosterol 1-rhamnoside 16-galactoside | 1.92845299942563e-06 | 5.38033105663223e-07 | 8.64686329255335e-07 | 6.08702879058604e-07 | 2.51744584893638e-05 | 1.32404498512364e-06 | 7.22048952819024e-07 | 2.01496400315626e-05 | 8.82143661173754e-07 | 6.01592207808692e-07 | 1.10580281843496e-05 | 5.93259587493638e-05 |
| pos\_2428 | Torvoside B | 0.00027214336625764 | 0.00029299043374777 | 0.000371652551359969 | 0.000185080990828676 | 0.000249325528619522 | 0.000278800024275103 | 0.000184490260103581 | 0.000255268544428027 | 0.000209093480443328 | 0.000328340014645593 | 0.00028211862711226 | 0.000295460885520611 |
| pos\_2429 | 2,3-Dihydrowithanolide E | 0.000127384523885907 | 9.14786367910374e-05 | 9.37807064434116e-05 | 0.000108290187905119 | 8.573149633607e-05 | 0.000100156369176581 | 0.000115390414181589 | 0.000110353808507772 | 9.68139760043642e-05 | 9.28294856983599e-05 | 0.000103361501684007 | 9.79315751963975e-05 |
| pos\_243 | Alisol C | 0.000485462565869364 | 0.000566610455487053 | 0.00134564403757729 | 0.00090789562621235 | 0.000526413541976121 | 0.000740982643095331 | 0.000688930415175148 | 0.000856388102264118 | 0.000543053188925919 | 0.000825520276984083 | 0.000798596003332935 | 0.00115122667359338 |
| pos\_2430 | Ginsenoside F2 | 0.000504104238632527 | 0.000372769037970874 | 0.000238431267183373 | 0.000188354129214614 | 0.000198822065909414 | 0.000232848176704314 | 0.000180289040926048 | 0.000179008919533856 | 0.000198875361693921 | 0.000159397941345368 | 0.000166044133404793 | 0.000165594659383224 |
| pos\_2431 | 6-Methylnicotinamide | 5.2767107940808e-05 | 6.94694110907714e-05 | 0.000101111108934104 | 7.92590644583323e-05 | 8.45792166864327e-05 | 7.4135139069021e-05 | 6.28436348839556e-05 | 6.46767133474121e-05 | 7.6446559373054e-05 | 7.38590317512617e-05 | 6.59754402953455e-05 | 7.80663474491479e-05 |
| pos\_2432 | LysoPE(0:0/24:1(15Z)) | 0.000439531281894513 | 0.000189991539408382 | 0.00018032460907419 | 0.000178896002123759 | 0.000293803864582384 | 0.000310382272199809 | 0.000316773933116171 | 0.000260575966779448 | 0.000233558241288715 | 0.000268154507042323 | 0.000256051966820447 | 0.000259849192370962 |
| pos\_2433 | 2-Methylpropyl glucosinolate | 4.62909732811988e-06 | 7.43212584228288e-06 | 5.64218467521365e-06 | 5.92086018817476e-06 | 5.27535285887455e-06 | 5.9949464069384e-06 | 4.46683529449396e-06 | 6.45591685156147e-06 | 6.82248518420545e-06 | 5.72971867168189e-06 | 5.64158472225204e-06 | 5.68710959147528e-06 |
| pos\_2434 | 2'',6''-Digalloyliriflophenone 3-C-glucoside | 2.71138878855124e-05 | 5.67058722166829e-05 | 2.27356827026058e-05 | 2.94871489370752e-05 | 4.26798169517287e-05 | 3.20893354660049e-05 | 2.23664511037802e-05 | 2.26580289223531e-05 | 4.80849035711413e-05 | 3.58333620305126e-05 | 3.19609740910686e-05 | 3.4890662282645e-05 |
| pos\_2435 | 2,4-Dihydroxyacetophenone 5-sulfate | 2.24753427404525e-05 | 2.724324835939e-05 | 2.35019948035419e-05 | 2.51053436148455e-05 | 2.17194262482223e-05 | 2.47849335268606e-05 | 2.32574889264542e-05 | 2.44098991645716e-05 | 2.75928390533056e-05 | 2.33391757554535e-05 | 2.59907533166469e-05 | 2.72334529406759e-05 |
| pos\_2436 | 3-Methylbutyl formate | 0 | 1.26860221211936e-05 | 0 | 1.80703206484355e-08 | 1.52792520189029e-10 | 4.94344974393575e-06 | 0 | 3.07564538132463e-09 | 0 | 6.27285690256681e-09 | 4.67646118804161e-12 | 0 |
| pos\_2437 | Cinnzeylanol | 0.00116516136008227 | 0.000627959449355881 | 0.000496081920918085 | 0.00050464108761012 | 0.00090745130390866 | 0.000849398183264967 | 0.000929793017013598 | 0.000709517997237221 | 0.00059871126169202 | 0.000796568041660228 | 0.000736057088055064 | 0.000687420297946113 |
| pos\_2438 | Acetyl tributyl citrate | 0.00380471028414881 | 0.00492283167566553 | 0.00486115766099175 | 0.00441955998505312 | 0.00340357954066029 | 0.00436640792312695 | 0.00397383545775824 | 0.00429429283609375 | 0.0045545030794792 | 0.00423000617180833 | 0.0040862250742413 | 0.0039183068194884 |
| pos\_2439 | Sitaxentan | 8.47121080014638e-05 | 0.000105976920491846 | 0.000110578813157716 | 8.96557274813029e-05 | 7.03242646261179e-05 | 8.98636570565857e-05 | 7.87866610836459e-05 | 8.24613933656935e-05 | 9.45934343931497e-05 | 7.33464241059544e-05 | 8.91116518280092e-05 | 8.52389659685712e-05 |
| pos\_2443 | 4-hydroxy-D4-neuroprostane | 2.44408778186996e-05 | 2.05168165532044e-06 | 7.3968280165898e-06 | 1.78431209904984e-05 | 5.77495176343126e-07 | 9.6676997630357e-06 | 6.69910442769162e-06 | 2.13529490795324e-05 | 1.1527392858706e-06 | 1.18579101646655e-06 | 1.35313722167544e-05 | 4.17823167735399e-05 |
| pos\_2444 | Camelliagenin C | 0.000702048694553241 | 0.000709277297197037 | 0.000552428079649909 | 0.000572350889728526 | 0.000701021663054444 | 0.000680155807418786 | 0.000580677868536607 | 0.000602275608674815 | 0.000648047230644676 | 0.00066449173602273 | 0.000633956949505906 | 0.000616911789121983 |
| pos\_2445 | Muricoreacin | 4.25899969776236e-05 | 5.41924926261131e-05 | 4.97132202514867e-05 | 3.02513447887427e-05 | 3.75950905940302e-05 | 4.62191461257939e-05 | 5.07637341791398e-05 | 4.34302161155473e-05 | 4.15624507577994e-05 | 3.47999428610525e-05 | 4.35195807477189e-05 | 6.95154738090095e-05 |
| pos\_2447 | Glutaminyl-Proline | 2.31809863394626e-05 | 1.68118526066031e-05 | 2.16875307192583e-05 | 2.66690191780064e-05 | 2.15490000989338e-05 | 2.48220411469834e-05 | 4.66765717519064e-05 | 2.77757692397742e-05 | 1.45283036969128e-05 | 2.93480915364013e-05 | 2.92378240877724e-05 | 2.47614876343263e-05 |
| pos\_2448 | 2-Methyl-1-methylthio-2-butene | 2.10566181780601e-06 | 2.81812203614476e-06 | 2.64541593276773e-06 | 7.01353572411076e-06 | 2.31940270672947e-06 | 6.37512888430624e-06 | 5.42534175502752e-06 | 6.05845625924512e-06 | 6.13637922698417e-06 | 9.81371104127437e-06 | 7.03885312964125e-06 | 5.09400111598504e-06 |
| pos\_245 | (22E)-3α,12α-Dihydroxy-5β-chol-22-en-24-oic Acid | 0.00078989775921188 | 0.000826504221973486 | 0.00125749667321076 | 0.00166492443030804 | 0.00167938617763971 | 0.00119280137596291 | 0.00141058201921447 | 0.00144915112949915 | 0.00135303550472736 | 0.00108997620293824 | 0.00121771229804525 | 0.000997809933797416 |
| pos\_2450 | 29-Norcycloartane-3,24-dione | 0.0001903564092994 | 0.000261260359643923 | 0.00017262856897534 | 0.000220554410481346 | 0.000231559222365111 | 0.000216735483057882 | 0.000236308669348806 | 0.000230651096334408 | 0.000203581103874919 | 0.000252121753337559 | 0.000225774980237443 | 0.000237437605647349 |
| pos\_2451 | Mocimycin | 1.34181463511195e-05 | 7.2967604328533e-06 | 1.38751179762921e-06 | 2.65794734131898e-06 | 5.30726378434057e-06 | 3.64284635939445e-06 | 2.56442465866907e-06 | 1.53936094980424e-06 | 6.32206268464368e-06 | 3.17620500695039e-06 | 2.1910626903825e-08 | 2.17972924650479e-06 |
| pos\_2452 | 1-Methylcyclohexanol | 5.95479017811488e-05 | 1.16823883827063e-05 | 1.14208114410881e-06 | 2.65057029695509e-06 | 2.12781192678533e-05 | 1.03895390670114e-05 | 1.65711667301303e-06 | 5.01753910285891e-07 | 8.8516445590414e-07 | 4.96438049610751e-06 | 9.97357002489492e-07 | 3.76100295139489e-07 |
| pos\_2454 | 2-(2-Furanyl)piperidine | 0.000337905760034986 | 0.000317987633483106 | 0.000152987131161874 | 0.000441004775179913 | 4.98267537063989e-05 | 0.000254553533159748 | 0.000321546524584577 | 0.000255237043287465 | 5.63203726639883e-05 | 0.000417594055088122 | 0.00025116559695426 | 0.000184577942786173 |
| pos\_2455 | Oleanolic acid 3-[rhamnosyl-(1->4)-glucosyl-(1->6)-glucoside] | 0.000200732850001168 | 8.28561771527811e-05 | 7.71109366071558e-05 | 8.32998119445091e-05 | 9.70740324886366e-05 | 0.000103945092730392 | 0.000122387542864416 | 0.000105710767927996 | 7.99018309864348e-05 | 9.42505812380809e-05 | 0.00010383978130272 | 0.000102320329439656 |
| pos\_2458 | LysoPE(0:0/24:6(6Z,9Z,12Z,15Z,18Z,21Z)) | 1.43414878794522e-07 | 4.69965320083898e-07 | 5.66949657327768e-08 | 3.62063415374496e-05 | 3.38637408416942e-09 | 2.79805381098318e-06 | 0 | 7.5666550590198e-08 | 5.33264494213015e-08 | 3.49791617601314e-08 | 1.08360520945546e-07 | 7.94386805919932e-09 |
| pos\_2459 | DG(22:4(7Z,10Z,13Z,16Z)/22:6(4Z,7Z,10Z,13Z,16Z,19Z)/0:0) | 0.000287552783881547 | 0.000141526420758727 | 0.000129493608833978 | 0.000121579057384733 | 0.000146447853932501 | 0.000143165779800958 | 0.000145989171933206 | 0.000152062666177559 | 0.00014328367142036 | 0.000135285584246468 | 0.000135073176667039 | 0.000134864757929873 |
| pos\_246 | Ser Arg Tyr Trp | 0.000417785095076963 | 0.000444537071219797 | 0.000236543234290743 | 9.14025383251152e-05 | 0.000528150742271857 | 0.000399798694383201 | 0.00012030013051234 | 0.000198268486444855 | 0.000267396618279624 | 0.00011414169864931 | 0.000204076090246773 | 0.000333685096021293 |
| pos\_2461 | Polysorbate 60 | 8.79419200681525e-05 | 6.01422363386559e-05 | 5.96240067770599e-05 | 5.22469227240212e-05 | 4.39011433353771e-05 | 5.76621498332308e-05 | 5.47672378241363e-05 | 6.83813827449343e-05 | 5.49796190916125e-05 | 5.99602725398654e-05 | 5.34146923873687e-05 | 5.91969189937421e-05 |
| pos\_2463 | 5-(3-Pyridyl)-2-hydroxytetrahydrofuran | 1.62355721941313e-05 | 2.15709364261615e-05 | 2.30000771529874e-05 | 1.9278150353792e-05 | 1.59572856815974e-05 | 1.79573335869918e-05 | 1.86170726805081e-05 | 2.00984297787559e-05 | 1.81264812400351e-05 | 1.8475269088663e-05 | 1.60418037414261e-05 | 2.00386722930214e-05 |
| pos\_2464 | PE(18:3(6Z,9Z,12Z)/P-16:0) | 0.000982819054100846 | 0.00105979945765721 | 0.000987437815589617 | 0.000955997561850481 | 0.000851432300892654 | 0.000945717597517421 | 0.000890083367833227 | 0.000964448241930506 | 0.00108211290470771 | 0.000882466434842218 | 0.000964319843731948 | 0.000942849534956141 |
| pos\_2465 | 2-Propyl-2,4-pentadienoic acid | 0.00011965603312769 | 0.000140355272809682 | 0.000127692064280408 | 0.000127086626960497 | 0.000106535958651109 | 0.00011475825813913 | 0.000115624298250676 | 0.000122823953966033 | 0.000121090357354994 | 0.000125754275392634 | 0.000119873955989885 | 0.000120524367447054 |
| pos\_2466 | Delta-Tocopherol | 0.000212310508826832 | 0.000210718920362896 | 0.000168489045334057 | 0.000215680487667553 | 0.000242740191313674 | 0.000205848890864187 | 0.000229641841845906 | 0.000196708029231623 | 0.000239049303999205 | 0.000223959235262587 | 0.00021593824573392 | 0.000159656504026834 |
| pos\_2469 | Aquifoliunine EIII | 0.000166607106658045 | 1.12302425206619e-05 | 1.31312111964694e-05 | 1.00442097790371e-05 | 8.13593033053848e-05 | 6.86912851220908e-05 | 6.32076083348043e-05 | 5.28297265421099e-05 | 2.05198260776519e-05 | 4.01747451175141e-05 | 4.72941896952058e-05 | 3.9028138720378e-05 |
| pos\_247 | Eupatoriopicrin | 0.000110393560096272 | 3.84258981134582e-05 | 0.000356614623397224 | 0.000929139152764679 | 0.000116660571391675 | 0.000313100160985077 | 5.11982727006365e-05 | 0.000608552789835729 | 3.07222483217532e-05 | 4.67308447352022e-05 | 0.000421200169206038 | 0.00143711016972093 |
| pos\_2470 | Methyl (2E,4Z,6E,8E,10E)-4,8-dimethyl-12-oxo-2,4,6,8,10-dodecapentaenoate | 2.4253872399052e-06 | 0 | 0 | 0 | 3.6549941466311e-07 | 3.22793957149293e-07 | 1.06046668826873e-06 | 2.39356216464499e-07 | 0 | 0 | 2.63706613226206e-07 | 0 |
| pos\_2473 | Asparaginyl-Serine | 0.000221942782090166 | 0.000340967724994265 | 0.000153560811252256 | 0.000177458840675257 | 0.000277289839003368 | 0.000233943293987755 | 0.000215526760875793 | 0.000187677229954768 | 0.000306163437746986 | 0.000178408840150453 | 0.000209929381415277 | 0.000168784113511587 |
| pos\_2474 | 1,3-Butadiene | 3.10128240225659e-05 | 2.96452726661854e-05 | 4.59104414086286e-05 | 4.31589335817736e-05 | 4.87262225780163e-05 | 4.28657173126743e-05 | 3.97209988399675e-05 | 3.8176809907322e-05 | 4.55668101971666e-05 | 3.97079570285442e-05 | 4.04051871860272e-05 | 4.18709844900889e-05 |
| pos\_2477 | Calenduloside B | 0.00014569153581729 | 3.50872149892967e-05 | 3.64804255094643e-05 | 2.78603779988715e-05 | 8.20475324509619e-05 | 7.98933164677473e-05 | 7.7714831546005e-05 | 6.1682536751159e-05 | 4.55326579973453e-05 | 5.8223934837991e-05 | 6.15119519440773e-05 | 5.54674481970003e-05 |
| pos\_2478 | Alpha-Trisaccharide | 2.51676539887598e-05 | 1.96134638245471e-06 | 4.14707769081668e-06 | 1.95911026239566e-06 | 1.4203199234513e-05 | 9.65907996878074e-06 | 1.11180609866394e-05 | 4.81081573084345e-06 | 4.57636555733242e-06 | 4.23815531604159e-06 | 6.2730938274898e-06 | 4.19533308044137e-06 |
| pos\_2479 | Hydroxypropionylcarnitine | 2.46472428247456e-07 | 1.80307037102888e-06 | 1.37321239503023e-06 | 4.03628120176365e-06 | 6.89545813656618e-07 | 2.70719988180865e-06 | 1.43565575719275e-06 | 2.33867252316053e-06 | 2.66671178658334e-06 | 1.24553458857329e-06 | 1.57768639081023e-06 | 2.32739945305157e-06 |
| pos\_248 | beta-Hydroxy-2',6'-dimethoxy-3',4'-methylenedioxydihydrochalcone | 0.0105177725409358 | 0.016733406022194 | 0.00854221066703499 | 0.0143663863773992 | 0.013387423544099 | 0.0121915274879058 | 0.012745271654071 | 0.0108057441430979 | 0.0151789518122954 | 0.0125244362121346 | 0.0122617980920402 | 0.00820294709589478 |
| pos\_2480 | DG(18:2n6/0:0/22:4n6) | 1.87980030276452e-05 | 6.28065889756083e-06 | 3.61878785198666e-06 | 0 | 2.75253011050685e-06 | 3.58959796033381e-06 | 6.94858583452415e-06 | 0 | 4.34526861826475e-06 | 1.14960229550314e-06 | 2.08162950726451e-06 | 8.25922817114535e-07 |
| pos\_2481 | 5alpha-Androst-16-en-3-one | 3.08139700162343e-05 | 3.3609181910169e-05 | 2.61650086157998e-05 | 2.70710028292846e-05 | 2.96099164964361e-05 | 2.86824350475611e-05 | 4.20555534145686e-05 | 3.95303491622409e-05 | 2.01504311599943e-05 | 4.10908617044075e-05 | 3.78924211542901e-05 | 4.34559610668537e-05 |
| pos\_2482 | Thyrotropin releasing hormone | 3.53186567160188e-06 | 5.04701545359071e-06 | 5.12188724210015e-06 | 2.45501564951924e-06 | 4.48969499002613e-06 | 3.50657546396496e-06 | 5.72494201785312e-06 | 5.48319817318222e-06 | 4.55349934694708e-06 | 5.94951286616722e-06 | 5.64773775873412e-06 | 5.61722742569338e-06 |
| pos\_2483 | Panaxynol linoleate | 0.000186181270303378 | 0.000236054602394262 | 0.000208922960767784 | 0.0002067005552441 | 0.000165855656377426 | 0.000201753373444296 | 0.000215607603250618 | 0.000204281756533901 | 0.000252231806811226 | 0.000197364639914754 | 0.000211501981467008 | 0.000191931058058424 |
| pos\_2484 | TG(18:3(6Z,9Z,12Z)/20:5(5Z,8Z,11Z,14Z,17Z)/22:5(4Z,7Z,10Z,13Z,16Z)) | 0.000101223314700819 | 0.000104792486396773 | 7.96633526369734e-05 | 0.000143983457438949 | 0.000125169101245074 | 0.000120013007346834 | 7.10039641302654e-05 | 9.16141653293204e-05 | 0.000236149254164603 | 0.000115881371332141 | 0.000134817133448307 | 0.000136834360375849 |
| pos\_2485 | LysoPE(0:0/22:4(7Z,10Z,13Z,16Z)) | 4.03979117958818e-05 | 1.50916352474123e-05 | 1.50488969004533e-05 | 1.77447071273852e-05 | 2.39113016903609e-05 | 2.15954086571908e-05 | 3.19845291618804e-05 | 2.10649601140344e-05 | 1.76142657016657e-05 | 1.88868284936359e-05 | 2.14732165506957e-05 | 1.89380450049548e-05 |
| pos\_2487 | Orciprenaline-3-O-sulfate | 0 | 0 | 3.7070553323935e-07 | 0 | 5.50079271474477e-05 | 0 | 4.2772083818159e-07 | 0 | 0 | 0 | 0 | 0 |
| pos\_2489 | 5,6-epoxy,18R-HEPE | 5.19472041063166e-05 | 5.78064296333908e-05 | 7.38099588744685e-05 | 3.37345697137391e-05 | 9.93609130563438e-05 | 6.36382801682552e-05 | 2.81691083005288e-05 | 3.97670132466415e-05 | 4.06229531882351e-05 | 0.000190239810809849 | 7.18706418208083e-05 | 5.47776101432955e-05 |
| pos\_249 | Lys His Lys His | 0.000126113635219666 | 6.54275216599904e-05 | 4.85418462552588e-05 | 4.83188232344704e-05 | 8.14425018552405e-05 | 7.58179502386204e-05 | 7.6253665402619e-05 | 6.58412736866386e-05 | 5.96017569298189e-05 | 6.13263117394739e-05 | 6.48099166669467e-05 | 6.02772172134717e-05 |
| pos\_2491 | 6-Hydroxyhexanoic acid | 5.22863357853225e-05 | 4.71520089551599e-05 | 2.67894848385891e-05 | 3.45682925051863e-05 | 4.4232645644285e-05 | 4.15203913003371e-05 | 3.70214831998335e-05 | 3.15138417621978e-05 | 3.2890601084393e-05 | 3.38238994927004e-05 | 3.29040442038209e-05 | 3.1340065180099e-05 |
| pos\_2492 | N,N'-Bis(gamma-glutamyl)cystine | 8.30399942172302e-06 | 7.509231405537e-06 | 7.251462622139e-06 | 6.39376226894116e-06 | 7.65843198885685e-06 | 7.89084668205486e-06 | 6.84328051917302e-06 | 7.27222470573264e-06 | 9.43826526527505e-06 | 6.45825478069312e-06 | 7.31932840276376e-06 | 6.75340594124197e-06 |
| pos\_2493 | Aflatoxin B2 | 1.05440756427274e-05 | 1.80609903590885e-05 | 1.11559049112591e-05 | 8.39002859034394e-06 | 1.26146774558189e-05 | 1.05510332989713e-05 | 1.90036239507784e-05 | 1.64014764301374e-05 | 9.96648958594254e-06 | 1.0717121959238e-05 | 1.5293032730623e-05 | 1.27297829222479e-05 |
| pos\_2494 | 12-Dehydroporson | 2.41332183329903e-05 | 2.09793840887961e-05 | 2.04008651906412e-05 | 1.65186487439353e-05 | 2.4002892706121e-05 | 1.99603222207551e-05 | 2.15236690008745e-05 | 2.10762014906803e-05 | 1.81499821033647e-05 | 2.34173401882721e-05 | 1.90408093247072e-05 | 2.44625356758918e-05 |
| pos\_2495 | (2E,11Z)-5-[5-(Methylthio)-4-penten-2-ynyl]-2-furanacrolein | 2.11095426557735e-06 | 1.3766368814109e-05 | 1.78476470395949e-07 | 9.39678934228033e-06 | 7.45585448147292e-06 | 4.6946945806039e-06 | 2.7454878033119e-06 | 2.18065286386368e-06 | 2.7429923704692e-05 | 3.59476089549864e-06 | 7.57752887583572e-06 | 1.71936779705604e-06 |
| pos\_2497 | Maclurin | 4.1867302898161e-06 | 6.60732270084177e-06 | 2.24625489163179e-07 | 3.63505769332061e-08 | 6.82196906146685e-06 | 4.2238537513956e-06 | 2.11359125008737e-06 | 0 | 6.7194812060049e-05 | 0 | 1.85404500249452e-05 | 2.43091955575473e-09 |
| pos\_2498 | Ubiquinone 6 | 0.000232258314081475 | 0.000372232155043399 | 0.000250593308988378 | 0.000240428581855567 | 0.00029360610366939 | 0.000279278719481049 | 0.000286145467215585 | 0.000257185692497049 | 0.000283853347812748 | 0.000361889541850605 | 0.000263783138061454 | 0.00021714213947047 |
| pos\_2499 | Nb-Lignoceroyltryptamine | 0.000312483033766758 | 0.000397872306414143 | 0.000339457723743958 | 0.000357944318966762 | 0.000276503780643919 | 0.000322499772584936 | 0.000290965921287374 | 0.000331266816421276 | 0.000355925571594243 | 0.000294759638685652 | 0.000330668050245447 | 0.000344340369753164 |
| pos\_25 | 9S,10R-Epoxy-6Z-octadecene | 0.000153990941112849 | 0.000177603040331368 | 8.13164042610102e-05 | 7.80320239001556e-05 | 9.46050624215945e-05 | 7.73299782856646e-05 | 7.0496500677293e-05 | 7.66374352340859e-05 | 9.62917279386766e-05 | 9.14360375651152e-05 | 8.4701327132151e-05 | 7.27644416131749e-05 |
| pos\_250 | DG(18:3(9Z,12Z,15Z)/18:3(9Z,12Z,15Z)/0:0) | 0.000431408726313067 | 0.000567848561544843 | 0.000430881727150147 | 0.000387675845360868 | 0.000401890305966086 | 0.000475826762061653 | 0.000369218779338367 | 0.000495802326308361 | 0.000443361631769962 | 0.000321566677038588 | 0.000371145681184238 | 0.000766899339761446 |
| pos\_2506 | Kentsin | 0.000337313028391849 | 0.000252083173296131 | 0.000230227047743043 | 0.000219148106023012 | 0.000217334016130527 | 0.000256828316860786 | 0.000249758156314654 | 0.000249777180330849 | 0.000252828468021193 | 0.000221440599107128 | 0.000241777059431734 | 0.000238818042334455 |
| pos\_2507 | PC(20:4(5Z,8Z,11Z,14Z)/22:1(13Z)) | 1.07040577431824e-05 | 9.81265224900472e-06 | 4.31482715553699e-05 | 2.03860826924467e-05 | 9.90143749173278e-06 | 1.80387216644884e-05 | 2.83215661437649e-05 | 5.29045259413594e-05 | 1.3086066011738e-05 | 1.45255635755154e-05 | 4.13173861473298e-05 | 8.01565551290706e-05 |
| pos\_2509 | 1-Nitro-5-hydroxy-6-glutathionyl-5,6-dihydronaphthalene | 0 | 1.31441136713916e-06 | 1.1660647479767e-05 | 0 | 1.17985972032071e-07 | 2.02927189226777e-06 | 0 | 1.01449383211653e-05 | 1.58074185741961e-05 | 2.01313021091912e-07 | 5.87336288597306e-06 | 2.76419978001768e-06 |
| pos\_251 | Melanostatin | 4.38020779967484e-05 | 0.000194417420818586 | 7.75198606467813e-05 | 3.26943037931574e-05 | 5.25596433511269e-05 | 6.22996754895094e-05 | 3.00914817265459e-05 | 4.57501553768693e-05 | 2.46918801155712e-05 | 0.000100732027946334 | 6.1379365747893e-05 | 0.000102497022865116 |
| pos\_2510 | Methyl 3-(2,3-dihydroxy-3-methylbutyl)-4-hydroxybenzoate | 8.23975298715076e-06 | 9.55382203048644e-06 | 4.69934630837858e-06 | 9.1673920108783e-06 | 7.65521709442052e-06 | 6.92462708797155e-06 | 7.07752245910167e-06 | 6.6595464768087e-06 | 6.91881323721833e-06 | 8.61894066940986e-06 | 7.25193969250128e-06 | 6.16464015352265e-06 |
| pos\_2511 | D-erythro-D-galacto-octitol | 0.000227993984861769 | 0.00061792108386283 | 0.000293727289161461 | 0.000222456865584201 | 0.000447491853975806 | 0.000319779874241041 | 0.000289808992953938 | 0.000275501906959151 | 0.000233442056611426 | 0.000406125567535773 | 0.00029575529955424 | 0.000271050327783896 |
| pos\_2512 | Dipivefrin | 0.000199237064660531 | 0.000280649472934069 | 0.000257676324820858 | 0.000218916522878535 | 0.000194944331401026 | 0.000239722443217579 | 0.000200764208378094 | 0.000233915616280884 | 0.000260137115246717 | 0.000209285114280883 | 0.000234892528254504 | 0.000208746131249508 |
| pos\_2513 | 2,2,4,4,-Tetramethyl-6-(1-oxopropyl)-1,3,5-cyclohexanetrione | 3.36548736988156e-05 | 2.99552204875407e-05 | 7.22708867156125e-05 | 1.78498059127225e-05 | 2.73643539267595e-05 | 3.38397000824036e-05 | 1.62187703909293e-05 | 2.70533900929533e-05 | 1.97865357802857e-05 | 0.000115482236156877 | 3.37399697811279e-05 | 4.89483259830906e-05 |
| pos\_2514 | PI(18:1(11Z)/18:1(11Z)) | 0.000244770999422418 | 0.00247932541710712 | 0.00258973328009714 | 0.00377111402736185 | 0.00221942555011873 | 0.00194476328920662 | 0.00169753867198032 | 0.00182595175469721 | 0.00304495384302324 | 0.0017710082578687 | 0.00119299614774424 | 0.00225582924272357 |
| pos\_2515 | Helianthoside B | 4.21144503259397e-05 | 7.42104038588207e-05 | 2.25859119724252e-05 | 3.99095548821571e-05 | 7.87708428446978e-05 | 5.38814023463355e-05 | 8.19118726491169e-05 | 7.34000496134874e-05 | 5.816947601842e-05 | 7.43661100668789e-05 | 7.81928145225747e-05 | 5.09058969295718e-05 |
| pos\_2516 | Pyrohyperforin | 0.000162585138229998 | 0.00013815369054573 | 0.000124167302558482 | 0.000116336510862394 | 0.000102806063820092 | 0.000128104804522825 | 0.000114224372567633 | 0.000122079528444267 | 0.000127506053243486 | 0.00010638036527525 | 0.000122262602425791 | 0.000125190756996134 |
| pos\_2517 | 2-(3-carboxy-3-(trimethylammonio)propyl)-L-histidine | 0.000363285470009658 | 0.000505521149828874 | 0.000359084173619076 | 0.000399705208178397 | 0.000272823077403001 | 0.000422246737761249 | 0.000369590521619527 | 0.000403484172119154 | 0.000415033808222874 | 0.000338315649054638 | 0.000427698187402319 | 0.000309157305998138 |
| pos\_2518 | 3, 5-Tetradecadiencarnitine | 1.35412891230623e-05 | 1.38573262559768e-05 | 1.24344524433854e-05 | 1.4642561805685e-05 | 6.69738087179705e-05 | 1.42346074062898e-05 | 1.2834465334907e-05 | 1.67960022541207e-05 | 1.39761779325816e-05 | 2.16021286513912e-05 | 1.393065826551e-05 | 1.49883591183907e-05 |
| pos\_2519 | N6-Galacturonyl-L-lysine | 1.11936476923638e-05 | 2.19257289647726e-05 | 8.09549121895838e-06 | 1.19802000743558e-05 | 1.5427162319353e-05 | 9.87956431828371e-06 | 1.31782647445316e-05 | 1.12999681649703e-05 | 2.17354360253433e-05 | 8.83049273174512e-06 | 1.27223426726039e-05 | 2.17532265209563e-05 |
| pos\_252 | Pseudaminic acid | 0.00027426840391063 | 0.000420158680022191 | 0.000241274026642376 | 0.000271078960312446 | 0.000312176701420862 | 0.000289577590142251 | 0.000340785082549709 | 0.000270176737380963 | 0.000250765841777579 | 0.000349793755446594 | 0.000285398203325568 | 0.000198224101163184 |
| pos\_2520 | N-Methylcalystegine C1 | 9.02059401866093e-05 | 0.000101107004094538 | 5.54819766167434e-05 | 6.55434426282851e-05 | 8.48550006166936e-05 | 6.31060377744696e-05 | 7.2598304608989e-05 | 6.80815725713873e-05 | 8.49886004454943e-05 | 6.65967026702587e-05 | 7.27543908304443e-05 | 7.72246013690668e-05 |
| pos\_2521 | CDP-DG(18:0/22:3(10Z,13Z,16Z)) | 1.64792776355478e-05 | 6.33876359934369e-06 | 3.09629009138558e-05 | 2.09730002819536e-05 | 1.85791942614296e-05 | 1.80441570775684e-05 | 9.96515645313579e-06 | 8.0942112799317e-06 | 1.88193671268594e-05 | 2.23641089104321e-05 | 1.68683011521122e-05 | 2.00202539589416e-05 |
| pos\_2524 | TG(16:0/14:0/18:0)[iso6] | 8.1039689728586e-06 | 4.35803791749774e-06 | 3.41385195450209e-06 | 2.22742358146156e-06 | 8.36738141888255e-06 | 4.41081323965608e-06 | 1.65875083973825e-06 | 1.82711656972351e-06 | 4.60807953809335e-06 | 2.9524563327241e-06 | 3.70586314234483e-06 | 5.55783100423788e-06 |
| pos\_2525 | Aspartyl-Glutamine | 5.23567740719502e-06 | 6.10852455395543e-06 | 4.77625395537121e-06 | 6.38941794700283e-06 | 3.89204884018037e-06 | 5.67507013199788e-06 | 6.85183372892717e-06 | 4.57153834818318e-06 | 3.43925703170368e-06 | 5.94576026930914e-06 | 5.47139150509515e-06 | 4.34004115015581e-06 |
| pos\_2526 | Rizatriptan | 1.47818999464143e-06 | 5.35258470911397e-06 | 6.0036511793396e-05 | 0 | 1.05564961627657e-06 | 2.16400010018138e-06 | 0 | 2.70658361312514e-06 | 5.93137462020422e-07 | 6.23798761108606e-06 | 1.68281891700194e-05 | 7.79413059431838e-06 |
| pos\_2527 | Quinquenoside II | 0.000105269556080405 | 0.000118252190014297 | 0.000114232957662997 | 9.66782615559802e-05 | 9.64349481341119e-05 | 0.000101079850209075 | 0.000102778392349572 | 0.000104930746109167 | 0.000118855616533879 | 9.85040192554956e-05 | 0.000107865482330217 | 0.000101152518996876 |
| pos\_2528 | (3'x,5'a,9'x,10'b)-O-(3-Hydroxy-6-oxo-7-drimen-11-yl)umbelliferone | 0.000128251730232898 | 0.000163460220814854 | 0.000160118379341609 | 0.000133165855758902 | 8.31364848856954e-05 | 0.000132570158119979 | 0.000128310052099191 | 0.000154664632676384 | 0.000120247162978059 | 0.00010181704779579 | 0.000118759010680975 | 0.000124073557922427 |
| pos\_253 | Val Glu | 6.50569985508459e-05 | 3.88531598940386e-05 | 0.000261833433841192 | 2.4644626892151e-05 | 2.31833532572606e-05 | 7.26799355403682e-05 | 1.44235102874569e-05 | 8.49082870116661e-05 | 1.28435798080013e-05 | 0.000141154214122962 | 0.000101945204001838 | 0.000227717309188201 |
| pos\_2530 | 5-Methyltetrahydrofolic acid | 3.0631211829128e-05 | 3.10700212699311e-05 | 1.83444281834334e-05 | 2.88919121121592e-05 | 2.9056730140632e-05 | 2.69474581804323e-05 | 2.52172050575113e-05 | 2.38811808205173e-05 | 1.50493725551229e-05 | 2.99886893239885e-05 | 2.59235143364407e-05 | 1.78948995171464e-05 |
| pos\_2531 | Cellulose, microcrystalline | 1.20240455763733e-05 | 2.05117805805916e-06 | 3.38125797060265e-05 | 4.84981382894595e-06 | 7.49769914856812e-06 | 8.26871048399801e-05 | 8.94337764678185e-06 | 9.13661414860887e-06 | 2.6070449155582e-05 | 8.53173557836718e-05 | 7.74135966907602e-06 | 6.99644344810066e-06 |
| pos\_2532 | Pentylbenzene | 1.46273394693293e-05 | 1.95760410676616e-05 | 1.61107808616947e-05 | 1.46053726880258e-05 | 1.63555534556035e-05 | 1.94497101349195e-05 | 1.21813983890383e-05 | 1.52154934589437e-05 | 1.54681745033204e-05 | 4.95868157181232e-05 | 1.52623314717108e-05 | 2.16189280747004e-05 |
| pos\_2534 | 2'-Deoxysepiapterin | 0.000180652089211543 | 0.000223946488691023 | 0.000155709303687548 | 0.000204017723239879 | 0.000186344082090371 | 0.000151361223767612 | 0.000197633362733642 | 0.000199205501031399 | 0.000210371908052917 | 0.000224232072848342 | 0.00017452028196234 | 0.000164147680938831 |
| pos\_2535 | TG(18:3(9Z,12Z,15Z)/14:0/22:6(4Z,7Z,10Z,13Z,16Z,19Z))[iso6] | 4.16187359486496e-06 | 1.18935007515658e-06 | 1.34922456796113e-05 | 7.91583230012348e-07 | 1.34099975753244e-05 | 2.25545099053081e-06 | 3.19408506507884e-06 | 2.22036227574492e-06 | 1.16134390533405e-06 | 2.44423567381065e-06 | 5.16136396216016e-08 | 1.63480095711742e-05 |
| pos\_2536 | Dexfenfluramine | 1.01187003818157e-05 | 9.44706186968478e-06 | 8.85671508372007e-06 | 8.6085044540635e-06 | 8.35690273363424e-06 | 8.63465361934171e-06 | 9.18435635311745e-06 | 9.34452106953925e-06 | 9.66152836431683e-06 | 8.2625262445071e-06 | 9.05573447350962e-06 | 8.41293594383881e-06 |
| pos\_2538 | SM(d18:1/24:0) | 2.46191408449343e-05 | 4.13329608806806e-05 | 3.87468135795868e-05 | 3.29473173072875e-05 | 1.64146079183835e-05 | 2.45405554426215e-05 | 9.42865630088846e-06 | 1.68900101350101e-05 | 3.03055227676533e-05 | 1.80708437508523e-05 | 2.42307468119173e-05 | 1.71922357480036e-05 |
| pos\_2539 | Mangostenol | 1.14570829089823e-05 | 0 | 0 | 0 | 0 | 5.89959489388373e-05 | 5.66888737309304e-08 | 0 | 0 | 4.05774414400369e-05 | 1.36416447250283e-08 | 0 |
| pos\_254 | DRB | 1.07021742746193e-06 | 0 | 6.345624467106e-05 | 8.24636013635903e-05 | 9.01701222653302e-06 | 2.9512419235653e-05 | 0 | 3.10111667131475e-05 | 0.000112626046693934 | 1.23871483839286e-06 | 4.33946399307031e-05 | 7.93174828974737e-05 |
| pos\_2541 | Histidinal | 2.96803544198392e-05 | 3.74311305118157e-05 | 4.3293856610723e-05 | 3.20214713430757e-05 | 2.62182214791165e-05 | 3.15799992975341e-05 | 3.59450337178528e-05 | 3.89333154294411e-05 | 4.31418394881663e-05 | 3.47031492655254e-05 | 3.94323080978663e-05 | 3.95586378289077e-05 |
| pos\_2542 | Allylestrenol | 1.09896241895143e-05 | 1.24904172135582e-05 | 1.50788954293823e-05 | 1.1491030725248e-05 | 2.06594911205591e-05 | 1.3659416794747e-05 | 9.21055416265615e-06 | 1.16977516211864e-05 | 5.42175174305388e-05 | 1.7310662488263e-05 | 2.10696395836396e-05 | 1.31216448748649e-05 |
| pos\_2543 | ent-15,16-Epoxy-1(10),13(16),14-halimatrien-19-oic acid | 0.000425908069764086 | 0.000307544283970919 | 0.00023882159085169 | 0.000270129421887008 | 0.000194787366508952 | 0.000284666560752313 | 0.000265015086484378 | 0.0002700742193403 | 0.000254767642251191 | 0.000236592966188242 | 0.000252111463501331 | 0.000252398241617323 |
| pos\_2544 | Soyasaponin V | 0.000148323213513991 | 3.49660332603153e-05 | 3.93837155010981e-05 | 3.1374432417564e-05 | 8.8464367229339e-05 | 8.47518414606377e-05 | 8.12542723078489e-05 | 6.41843200765479e-05 | 4.88983022708435e-05 | 6.02737511665428e-05 | 6.40541319796577e-05 | 5.96495666525213e-05 |
| pos\_2545 | Pipermethystine | 7.0901747245348e-05 | 0.000113988381340402 | 5.8274003546733e-05 | 0.000102914954388013 | 8.49918568684619e-05 | 8.33179292980038e-05 | 8.47123938047862e-05 | 6.38980873857014e-05 | 9.83515233422554e-05 | 8.20416545504879e-05 | 8.13422567362128e-05 | 5.59043564759193e-05 |
| pos\_2546 | 2-(Methylthiomethyl)furan | 3.17605958139711e-05 | 5.4707709699493e-05 | 2.51327582248597e-05 | 3.07986237126703e-05 | 3.94481264302595e-05 | 3.65101538582063e-05 | 4.38022730484263e-05 | 3.63002501639507e-05 | 3.49377863270476e-05 | 3.54299399954126e-05 | 3.78080808961867e-05 | 2.91436034275017e-05 |
| pos\_2548 | N-Methylserotonin | 1.58344486374134e-05 | 2.22679781042675e-05 | 1.36426991570246e-05 | 1.44506340881881e-05 | 1.90294210438755e-05 | 1.60938419923443e-05 | 1.3656250866892e-05 | 1.46743004999234e-05 | 1.84986983085626e-05 | 1.11055372136484e-05 | 1.50693653487401e-05 | 1.18890859049669e-05 |
| pos\_2549 | Corchorusoside E | 0.00022861841819666 | 0.000458789887513757 | 0.000396113844600145 | 0.0003305743860551 | 0.000394485865773998 | 0.000314353686457029 | 0.000388174442661521 | 0.000459502800579658 | 0.000477859980334896 | 0.000327290413048308 | 0.000420739989948233 | 0.000301249265514088 |
| pos\_255 | Nigroxanthin | 0.000239382087983262 | 0.000160193169994184 | 0.000146324005221377 | 0.00031002789447295 | 0.00018684964357056 | 0.000225769125169077 | 0.000250630526690715 | 0.000231045608656179 | 0.000255206969215497 | 0.000248825520324775 | 0.000257073735118376 | 0.000255256236511819 |
| pos\_2552 | p-Ethylacetophenone | 6.62907313017105e-06 | 4.18934472843828e-06 | 3.56054782944348e-06 | 1.96512509045179e-05 | 1.00233837653847e-06 | 7.1330895070776e-06 | 1.09206012867093e-05 | 7.42232906160522e-06 | 9.95386046704359e-07 | 1.18707092897763e-05 | 7.32391900096052e-06 | 6.7117930139059e-06 |
| pos\_2553 | N-Monodesmethyl-rizatriptan | 0.00012508407240431 | 3.71708192026817e-05 | 7.67347546973075e-06 | 5.56025126501246e-05 | 9.12196120175171e-06 | 3.79885656032865e-05 | 7.75401332935037e-06 | 8.16289855051776e-06 | 1.86609852290504e-05 | 0.000121805741897046 | 2.84979434409809e-05 | 1.17325864674505e-05 |
| pos\_2554 | Melongoside O | 2.68822581672191e-06 | 3.07997062670546e-06 | 1.44278422192959e-06 | 1.33047655697517e-06 | 2.88703141514821e-06 | 1.80360182017325e-06 | 6.57422552541767e-06 | 3.14348888086015e-06 | 3.37934566747709e-06 | 4.18133554812681e-06 | 3.26088871288975e-06 | 2.48113420115545e-06 |
| pos\_2555 | Quinaprilat | 1.60076463371593e-05 | 1.38041446717545e-05 | 9.66885216738173e-06 | 8.864010439617e-06 | 1.9985645919524e-05 | 1.37390957244672e-05 | 1.33501343280909e-05 | 1.29753200811514e-05 | 1.40296142056044e-05 | 1.31315404869492e-05 | 1.0657653659296e-05 | 1.79137506639091e-05 |
| pos\_2556 | Pomonic acid | 0.000158029336247826 | 0.000190629821561473 | 0.000343972929746201 | 7.30191321841535e-05 | 0.000177463043482129 | 0.000243488389945332 | 5.09562698142763e-05 | 0.000157029697172116 | 8.33617521256331e-05 | 0.000393697148849041 | 0.000217798543384647 | 0.000282236612994075 |
| pos\_2557 | TG(8:0/10:0/i-16:0) | 0.00828374034143372 | 0.00940077607693951 | 0.00709174357539526 | 0.00550412646275679 | 0.00470221897267896 | 0.0105907136480411 | 0.00614769060086394 | 0.0102316643889816 | 0.00596652649767274 | 0.00935830414992716 | 0.0104424754406445 | 0.00848451079544205 |
| pos\_2559 | PE(14:0/14:0) | 0.000544727638976925 | 0.000380128300014322 | 0.00031578320501969 | 0.000359389371107796 | 0.000313142366106549 | 0.000398680962692749 | 0.000380705457793481 | 0.000367782031814779 | 0.000364558027011601 | 0.00033378698715755 | 0.000352166151869275 | 0.000368346070772485 |
| pos\_256 | Pterosin O | 1.48042248295362e-06 | 1.51214180526283e-06 | 2.12305613131637e-06 | 1.09108728293658e-06 | 6.72113644228453e-06 | 2.3799048065479e-06 | 2.28514397779588e-06 | 8.22056854187029e-06 | 9.20910508878147e-07 | 9.60204983920656e-06 | 6.35323922153918e-06 | 2.15977952246844e-05 |
| pos\_2560 | DG(18:1(11Z)/22:5(7Z,10Z,13Z,16Z,19Z)/0:0) | 3.35700459685172e-06 | 8.53022028763727e-06 | 3.18313477722567e-06 | 1.94869876836947e-06 | 1.16357941017589e-06 | 4.29079676881982e-06 | 0 | 0 | 0 | 1.59622090187457e-06 | 1.99472937528883e-06 | 1.09662460070409e-06 |
| pos\_2561 | DG(20:2(11Z,14Z)/22:5(4Z,7Z,10Z,13Z,16Z)/0:0) | 2.33541365243932e-05 | 2.9603250650573e-05 | 7.79964993748026e-06 | 4.83029178687148e-06 | 6.86434976181779e-06 | 5.84352012688452e-06 | 2.81665993863711e-06 | 9.44305659041683e-06 | 9.47812489132107e-06 | 3.08452865640491e-06 | 5.73223919856015e-06 | 5.55510407325477e-06 |
| pos\_2562 | 1-[1,4-Dihydro-4-nonyl-5-(1-oxodecyl)-3-pyridinyl]-1-dodecanone | 3.01503526850557e-05 | 2.63019102952351e-06 | 4.50724971753832e-06 | 1.73273849522917e-06 | 1.1895460328623e-05 | 9.34344439074327e-06 | 8.01062375118181e-06 | 5.29889041358385e-06 | 2.82509120993476e-06 | 5.00528160083114e-06 | 3.74368251008043e-06 | 2.80739337989001e-06 |
| pos\_2563 | Quercetin 7-(rhamnosylglucoside) | 1.89204843123998e-05 | 8.22772547189543e-07 | 4.77834601208687e-07 | 0 | 5.26912732179718e-06 | 1.97668920760849e-06 | 3.45236659756525e-06 | 2.86207344892826e-06 | 0 | 9.00837741176844e-07 | 8.14963148010191e-07 | 2.62027643369006e-06 |
| pos\_2564 | Chloramphenicol | 4.14677943783303e-05 | 2.23426277468754e-06 | 7.21659339893269e-06 | 4.69474357072721e-06 | 2.36253759964185e-05 | 1.88244886856501e-05 | 1.73427031066853e-05 | 1.32396692612446e-05 | 7.49329937851211e-06 | 1.08983115699546e-05 | 1.37971607032426e-05 | 1.00853646729404e-05 |
| pos\_2565 | Phytosulfokine b | 1.21011420215864e-05 | 1.28082555267511e-05 | 0 | 9.00809993142853e-06 | 1.31091222232293e-05 | 1.01549231861361e-05 | 0 | 0 | 0 | 8.84953248856816e-06 | 2.12273876301124e-06 | 1.8192695533641e-07 |
| pos\_2566 | 4-Hydroxy duloxetine glucuronide | 2.16327684462331e-05 | 1.1566600584301e-05 | 7.62135251938309e-06 | 8.0340160987918e-06 | 1.62216353775046e-05 | 1.16377918214777e-05 | 1.57360248881672e-05 | 1.17859386867249e-05 | 1.25684281971292e-05 | 9.49337161779851e-06 | 1.20093839524664e-05 | 1.59737457764242e-05 |
| pos\_2567 | Carbamazepine | 1.86916581753196e-05 | 2.32391509617441e-05 | 2.0922219918081e-05 | 3.89418165062847e-05 | 1.52492697840683e-05 | 1.79144695337293e-05 | 3.63953661442669e-05 | 2.77587205549202e-05 | 3.09339164059838e-05 | 1.79777513644513e-05 | 2.61121431279403e-05 | 1.79330441737817e-05 |
| pos\_2568 | Ramontoside | 0.000242644339159757 | 0.000217729606293515 | 2.05332612217408e-07 | 6.66342777296451e-05 | 0.000266394159727697 | 0.000129364256904878 | 2.05633266207401e-06 | 2.92008604518797e-07 | 2.85150101923268e-06 | 8.26105194462058e-05 | 3.9964472938893e-06 | 2.82703405727531e-06 |
| pos\_257 | Isognaphalin 8-butyrate | 4.2395225904706e-06 | 1.58282101127207e-05 | 1.06017582581873e-05 | 2.05090651927647e-05 | 3.19795775706057e-05 | 1.06560919918051e-05 | 2.13382183355026e-05 | 1.79828786464474e-05 | 5.51637964125135e-05 | 1.43070155606541e-05 | 2.35569148285465e-05 | 1.8185514065257e-05 |
| pos\_2571 | Quercetin 3-O-(6"-malonyl-glucoside) 7-O-glucoside | 0.000241285975752402 | 5.52560565111994e-05 | 6.13775493407415e-05 | 5.15384566862644e-05 | 0.00013919985804936 | 0.000137877460569218 | 0.000128666059974346 | 0.000114364827351696 | 7.84329881702453e-05 | 9.18116954846874e-05 | 9.80360886618645e-05 | 9.29234040390045e-05 |
| pos\_2572 | Phenylacetic acid | 1.67199395185248e-06 | 3.23591535638792e-06 | 1.34494236562553e-05 | 2.23598351993206e-06 | 9.1018936700864e-07 | 5.03579668445133e-06 | 9.59355729285779e-06 | 9.36535116964775e-06 | 2.05041272615849e-06 | 1.26169219431028e-06 | 6.3394542439504e-06 | 9.71883077543858e-06 |
| pos\_2573 | Methylthiomethyl hexanoate | 0.000240107505502013 | 0.000273001645860926 | 0.000236893346022484 | 0.000234796892084767 | 0.00027484450852713 | 0.000224998612606628 | 0.000316783712279081 | 0.000290676393569102 | 0.000224774427234256 | 0.000284860851857344 | 0.000253843559755103 | 0.000351353606834885 |
| pos\_2575 | Paromomycin | 6.40881556995137e-06 | 3.33793911522889e-06 | 8.26811761841625e-06 | 2.04441317756713e-05 | 1.33628300733371e-05 | 7.67958437692962e-06 | 8.57144318267568e-06 | 1.28808527191101e-05 | 1.21989066855124e-05 | 9.00341273344152e-06 | 1.07311110707339e-05 | 2.67481697662334e-05 |
| pos\_2576 | Ganoderic acid U | 8.9562602564984e-05 | 0.000135434771791295 | 0.000158393851090148 | 0.00015218509141268 | 0.000116675387062521 | 0.000133493503493901 | 0.000157592764054044 | 0.000189344946287217 | 0.00014126070687177 | 0.000131526325457493 | 0.000149432589770252 | 0.000160410768689247 |
| pos\_2577 | (3beta,22E,24R)-23-Methylergosta-5,7,22-trien-3-ol | 4.22657387177802e-05 | 5.30164073750522e-05 | 5.02323101517479e-05 | 7.67353948194543e-05 | 3.72750874068235e-05 | 4.7806716055611e-05 | 4.76165441702343e-05 | 4.56021193952734e-05 | 2.72711991769228e-05 | 4.55431513286759e-05 | 3.97119466365803e-05 | 4.53444969119272e-05 |
| pos\_2578 | TG(14:1(9Z)/14:1(9Z)/o-18:0) | 8.74660428373685e-06 | 1.04490570919532e-05 | 1.79953957107142e-05 | 9.02075376518089e-06 | 1.1696688939659e-05 | 1.46388391989801e-05 | 8.91864774664675e-06 | 1.23401231386614e-05 | 1.62364342583346e-05 | 1.36991625544846e-05 | 1.47790286592089e-05 | 1.26202814232952e-05 |
| pos\_2579 | DG(20:4(5Z,8Z,11Z,14Z)/14:0/0:0) | 5.74641050700464e-06 | 8.46916905963131e-06 | 1.08337701199529e-05 | 1.20484369379059e-05 | 1.36484107841492e-05 | 1.4277946283185e-05 | 1.61551539593263e-05 | 1.12408932008659e-05 | 1.8873088992216e-05 | 1.38755247028298e-05 | 1.44146797825502e-05 | 9.95623623692577e-06 |
| pos\_258 | Ala Ala Ser Pro | 3.40895282706212e-05 | 4.77869516941917e-05 | 1.96730377013266e-05 | 4.22501338758585e-05 | 2.97654837260369e-05 | 3.30612540144046e-05 | 3.38766085549492e-05 | 2.86220597296157e-05 | 4.23561411428939e-05 | 4.89761823384719e-05 | 3.43797914858613e-05 | 2.26532765230405e-05 |
| pos\_2580 | Adouetine Y | 0.000792556103695019 | 0.00081356482012843 | 0.00050827945159905 | 0.000411277831077791 | 0.000488710409378786 | 0.000610924524875745 | 0.000465012868969362 | 0.000571043757874537 | 0.000504541631169951 | 0.000506482304154964 | 0.000562887763130639 | 0.000784567463026934 |
| pos\_2581 | Ganglioside GA2 (d18:1/12:0) | 5.11238598427932e-06 | 5.56354859170235e-06 | 4.56681867217786e-06 | 4.34785367806144e-06 | 5.512610156894e-06 | 5.24000111923025e-06 | 5.54061509835234e-06 | 5.49397173657182e-06 | 4.61758259017143e-06 | 5.50380815822222e-06 | 4.30363435184566e-06 | 4.79909884348406e-06 |
| pos\_2582 | Pimentol | 2.25941324372133e-05 | 5.99744902977763e-06 | 4.72419668646686e-06 | 3.85706133474216e-06 | 1.32630688384619e-05 | 1.19445659161415e-05 | 1.36235232455528e-05 | 8.95449332700837e-06 | 6.52604822297348e-06 | 1.133635303402e-05 | 7.17726619342342e-06 | 8.28586861325428e-06 |
| pos\_2583 | Adrenochrome o-semiquinone | 6.80954248026754e-05 | 9.64659412498125e-05 | 5.57806879067264e-05 | 6.51210688404687e-05 | 8.43868562234508e-05 | 6.41069505218919e-05 | 5.972672590761e-05 | 6.00287896782271e-05 | 7.51261437289625e-05 | 5.21795513963824e-05 | 6.39777471087447e-05 | 6.799520686661e-05 |
| pos\_2584 | N-Phenylacetyl pyroglutamic acid | 0.000160764720996139 | 0.000205709198019299 | 0.00010609283590226 | 0.000144384026658091 | 0.000125737579213678 | 0.000122135468002903 | 0.000161267332460841 | 0.000132863501587275 | 9.06923597699995e-05 | 0.000192159373397606 | 0.000142527635536402 | 0.000121150031300752 |
| pos\_2585 | Leonuriside A | 0.000166443714974721 | 7.99073789422112e-05 | 5.37701216634469e-05 | 6.69602040710461e-05 | 0.000102194454473522 | 0.000107133162876969 | 9.3410958740943e-05 | 8.24550612726146e-05 | 6.34862496627339e-05 | 8.0200945927218e-05 | 7.99836441054371e-05 | 7.22520443827848e-05 |
| pos\_2587 | Lettucenin A | 8.99753318480094e-05 | 0.000115657337407928 | 0.000194040976161546 | 0.00021206175454169 | 0.00020274511850803 | 0.000150834257136382 | 0.000200881166944013 | 0.000226628374829862 | 0.000257801848493885 | 0.000238965176817526 | 0.000223440614646888 | 0.000203841299739229 |
| pos\_2588 | Citroside A | 0.000138361437007321 | 0.00021138795766799 | 0.000128301556995495 | 0.000201685486239539 | 0.000176418165904124 | 0.00016104442126008 | 0.000184660749197027 | 0.000175407343311839 | 0.000171566861561433 | 0.000136133853596129 | 0.000173680112065264 | 0.000185247193408686 |
| pos\_2589 | Galabiosylceramide (d18:1/24:0) | 9.41142854784056e-05 | 1.52082480562499e-05 | 1.81207063093703e-05 | 1.3027442863834e-05 | 4.87365359187478e-05 | 4.58530395613078e-05 | 4.14173342817697e-05 | 2.79668595324117e-05 | 2.43657194461659e-05 | 3.32216898319554e-05 | 2.62806084618215e-05 | 2.6921320802051e-05 |
| pos\_259 | RETUSOQUINONE | 1.74795508773294e-05 | 2.39357856677179e-05 | 1.38412274664502e-05 | 2.06312853572244e-05 | 1.65337397417784e-05 | 1.89050555617991e-05 | 3.11100161243635e-05 | 2.46069352693525e-05 | 1.83720843466101e-05 | 2.29261432781532e-05 | 2.24199626534121e-05 | 1.38045949611583e-05 |
| pos\_2590 | Progoitrin | 6.71245569889964e-05 | 4.73590757380254e-06 | 6.94682829518072e-06 | 4.34031542746719e-06 | 2.77517643292907e-05 | 2.59987312998249e-05 | 1.82841007697627e-05 | 1.19670665547861e-05 | 4.77981668719049e-06 | 1.09086978465145e-05 | 1.59059848290462e-05 | 8.19107472622015e-06 |
| pos\_2591 | Guanosine tetraphosphate adenosine | 2.26012957598124e-05 | 7.54206782434312e-06 | 1.29345317030223e-05 | 8.87384291884018e-06 | 1.52688465808484e-05 | 1.32627086995405e-05 | 1.73624579953366e-05 | 1.34109809852893e-05 | 8.18101966910037e-06 | 7.62819643636935e-06 | 1.07521052954862e-05 | 1.25024947875429e-05 |
| pos\_2592 | 12,14-Octacosanedione | 1.08905613159377e-05 | 1.78145043203552e-05 | 1.55682755799906e-05 | 1.80588373438088e-05 | 1.80278648684339e-05 | 1.34856063868507e-05 | 1.77832177966935e-05 | 1.36296908171829e-05 | 1.87973231305542e-05 | 1.40984368053836e-05 | 1.73329621468878e-05 | 1.35455963473884e-05 |
| pos\_2593 | 5-Androstenediol | 2.50548650661536e-05 | 2.76165033882918e-05 | 2.48308283270829e-05 | 2.43012375418344e-05 | 2.09931616612332e-05 | 2.5090682649701e-05 | 2.18381731628036e-05 | 2.46415028265626e-05 | 2.46694664201742e-05 | 2.63671922219607e-05 | 2.53590403823299e-05 | 2.41719326089779e-05 |
| pos\_2594 | PS(MonoMe(13,5)/DiMe(11,5)) | 0.000341702404204824 | 0.000376607880526836 | 0.000357542411005626 | 0.000344701595853548 | 0.000302181126270102 | 0.000309231027374163 | 0.000306444523126778 | 0.000334505670731907 | 0.000319485006054664 | 0.000355801565511832 | 0.000328541048030009 | 0.000323726722623897 |
| pos\_2595 | Sanguisorbin B | 0.000440631711845507 | 0.00106542532991702 | 0.000961483506861328 | 0.000925438981453857 | 0.000588393598529989 | 0.000635925489581276 | 0.000578693197784452 | 0.000537012454994328 | 0.000533329611067062 | 0.000721344077249394 | 0.000798753770572904 | 0.000493918491921796 |
| pos\_2596 | Sakuranetin | 1.41865130030162e-07 | 0 | 0 | 4.11817296911475e-05 | 0 | 3.9015460397005e-07 | 0 | 0 | 0 | 2.29271103492789e-07 | 0 | 2.11823103357887e-06 |
| pos\_2597 | Forasartan | 4.8897555979896e-05 | 2.44615884788046e-05 | 2.34487850949537e-05 | 2.34414743308556e-05 | 2.62102923233104e-05 | 2.68693904644206e-05 | 2.39357535859048e-05 | 2.79243763105828e-05 | 2.43715309791098e-05 | 1.90069841155258e-05 | 2.41281564093107e-05 | 3.42452555314782e-05 |
| pos\_2598 | PE(20:2(11Z,14Z)/P-18:1(11Z)) | 1.00065925609953e-05 | 0 | 1.39885106579711e-06 | 9.78341040225147e-07 | 7.55312121157693e-07 | 1.03764683334986e-06 | 0 | 8.21107638895384e-07 | 1.26803010022503e-06 | 1.56006829390701e-06 | 7.47095257542791e-07 | 8.87440422856187e-07 |
| pos\_2599 | N-Acetylglutamic acid | 4.88893104233074e-05 | 5.64254440011774e-05 | 6.31993308693238e-05 | 5.48792158419953e-05 | 5.51813435167576e-05 | 4.51414247436176e-05 | 4.47738830434078e-05 | 5.42227361568071e-05 | 5.53149622823581e-05 | 7.39771311398314e-05 | 4.60187047636781e-05 | 5.31676180517487e-05 |
| pos\_26 | 3,4-Dimethylstyrene | 8.05601787430062e-05 | 0.000111854623742039 | 0.000121965086681268 | 0.000110911677821122 | 9.6846366633406e-05 | 0.00010122605548856 | 0.000119372582178657 | 0.000115589724544942 | 9.88079464156822e-05 | 0.000112709696430345 | 0.000111933445819291 | 0.000111309151659614 |
| pos\_260 | PA(18:3(6Z,9Z,12Z)/16:0) | 0.000297408497618493 | 0.000381431476182294 | 0.000467838172688707 | 0.000347717391232166 | 0.00024822024055482 | 0.000293324075869217 | 0.000293863658274156 | 0.000347588836574223 | 0.000347924239561906 | 0.000286012233541594 | 0.000322178726726634 | 0.000336743175244021 |
| pos\_2601 | NAD | 3.91101563380337e-06 | 1.65023703491074e-05 | 8.14748076290553e-06 | 1.1651497515915e-05 | 1.04663684851581e-05 | 6.29712161088246e-06 | 3.83149556668853e-06 | 9.47251147881467e-06 | 8.54964600169767e-06 | 7.6698307097145e-06 | 4.01225875486823e-06 | 5.5006413296803e-06 |
| pos\_2602 | (S)-Esomeprazole | 1.69456105290472e-05 | 1.29010374122944e-05 | 1.33232343163086e-05 | 1.15516882911234e-05 | 1.81775078629932e-05 | 1.15326053384553e-05 | 1.65643369871073e-05 | 1.4705215869362e-05 | 1.17776761266574e-05 | 1.48720728336546e-05 | 1.18483727387785e-05 | 1.50204039216527e-05 |
| pos\_2603 | 7-O-Acetylaustroinulin | 0.000298273107067563 | 0.000295330138668931 | 0.000275950547014698 | 0.000266287970276373 | 0.000236362780452809 | 0.000279052256695013 | 0.00027212738948041 | 0.000275914485954569 | 0.000293468027780569 | 0.000262202299205358 | 0.000282462598978459 | 0.000280902487729047 |
| pos\_2604 | Margrapine B | 2.4972644399794e-05 | 4.23593870366917e-05 | 2.41388302053765e-05 | 2.38032480482999e-05 | 2.78348557399536e-05 | 2.59047784879948e-05 | 4.22716321299042e-05 | 4.02524834423218e-05 | 2.54201141974624e-05 | 2.37549413015651e-05 | 3.55840244315269e-05 | 3.02119965860262e-05 |
| pos\_2605 | Arborinine | 0.000236124582464488 | 0.000338140164961007 | 0.000157929579037224 | 0.000229552468502753 | 0.000267408931579528 | 0.000244173230614755 | 0.000216746866799229 | 0.000193078201509399 | 0.000245477289444679 | 0.000273902192533796 | 0.000226131827905009 | 0.000187912830759202 |
| pos\_2606 | Methyl 5-hydroxyoxindole-3-acetate | 4.15704365081531e-07 | 0 | 3.27640695591403e-06 | 9.67895148924085e-06 | 3.25043151951463e-06 | 0 | 2.07687407073991e-06 | 2.99058110554927e-07 | 4.6946549516038e-06 | 2.80031235295537e-06 | 5.57870703759476e-06 | 6.51152605889781e-06 |
| pos\_2607 | PS(DiMe(11,5)/MonoMe(9,5)) | 0.000578857471670631 | 0.000443168355895264 | 0.000357259992928781 | 0.00022015014270156 | 0.000329474279219508 | 0.000327417855780569 | 0.00028696279116321 | 0.000263218204200946 | 0.00031727029442554 | 0.000242329334063514 | 0.000258323147768887 | 0.00025035785589854 |
| pos\_2608 | Hoduloside I | 6.46936117789252e-05 | 7.42535689743216e-05 | 7.21217691945652e-05 | 6.05251733025153e-05 | 5.88064816886072e-05 | 6.60135223600918e-05 | 6.19701249090267e-05 | 6.37974535003557e-05 | 7.2929123938605e-05 | 6.26100298356625e-05 | 6.60986396296764e-05 | 6.02842487522772e-05 |
| pos\_2609 | Pibutidine | 1.20158697427259e-05 | 1.7143282184349e-05 | 2.21806400966383e-05 | 2.00946276892841e-05 | 2.07834503200168e-05 | 1.70086897851291e-05 | 2.50090864607798e-05 | 2.25845899699257e-05 | 1.83860343757798e-05 | 1.96389377253813e-05 | 1.94065689153452e-05 | 3.1253090160902e-05 |
| pos\_261 | 5,6-dehydro Arachidonic Acid | 0.000179428379581738 | 0.000221448282969291 | 0.000203543861559034 | 0.000200927589902023 | 0.000151357822327817 | 0.000193656651866008 | 0.000165291098040345 | 0.000193998993040339 | 0.000212206881959395 | 0.000168437466105303 | 0.000189655114564521 | 0.000174900372990387 |
| pos\_2611 | Kanzonol W | 9.60313158748836e-06 | 1.59753230254369e-05 | 1.19410600274171e-05 | 1.04347786745713e-05 | 9.91200688688158e-06 | 1.09679915327708e-05 | 1.50174374383525e-05 | 1.39927831158471e-05 | 1.26796570652182e-05 | 9.55853556600188e-06 | 1.26007065850005e-05 | 1.0573245178261e-05 |
| pos\_2612 | Thiosulfate | 1.61842435318096e-05 | 1.95244640379494e-05 | 2.00647174824632e-05 | 1.72499737067871e-05 | 1.42298111337889e-05 | 1.67922491743628e-05 | 1.48003330599151e-05 | 1.85665760385837e-05 | 1.94210599464742e-05 | 1.50701189464502e-05 | 1.61477142270422e-05 | 1.64891487601941e-05 |
| pos\_2613 | LPA(18:0e/0:0) | 0.000484206959178751 | 0.000442814039109861 | 0.000464270687925337 | 0.000401799420958882 | 0.000423286368042145 | 0.00109868783511714 | 0.000351992217276292 | 0.00108561381551744 | 0.000401571655375294 | 0.00247873489847143 | 0.00204127355806662 | 0.000384830040725994 |
| pos\_2614 | 1,3-Octadiene | 9.05533321730807e-06 | 8.14008722493443e-06 | 8.3095380248906e-06 | 8.08727932991682e-06 | 7.98905405480313e-06 | 8.06460971736695e-06 | 9.35858868770022e-06 | 9.5924540508493e-06 | 9.00326591543497e-06 | 8.86787264993559e-06 | 8.13915119824859e-06 | 7.67070718798593e-06 |
| pos\_2615 | PC(20:5(5Z,8Z,11Z,14Z,17Z)/20:1(11Z)) | 0.000786058950734136 | 0.000879474229693813 | 0.000748932358603571 | 0.000986111154525099 | 0.0009322604545892 | 0.00086146303212008 | 0.000770041063789157 | 0.000787751771134466 | 0.00090279930826381 | 0.000897234618469162 | 0.000854538228908768 | 0.000742903590827136 |
| pos\_2616 | Ganglioside GM3 (d18:1/16:0) | 0.00010091059135145 | 6.93604044477036e-05 | 5.22373901535393e-05 | 5.09268930373526e-05 | 5.06473479145007e-05 | 6.13558132882948e-05 | 6.15671414706596e-05 | 6.33446259764978e-05 | 5.9589805777903e-05 | 6.27665577714981e-05 | 6.27516243531895e-05 | 6.08820294514038e-05 |
| pos\_2617 | 2-Acetyl-4,5-dihydrothiazole | 3.18200673789147e-05 | 4.52727615446724e-05 | 4.14433315809752e-05 | 3.38947121389248e-05 | 3.02001364459962e-05 | 3.36427564332278e-05 | 2.9319912447283e-05 | 3.40969068361802e-05 | 4.23184027977601e-05 | 3.21093365726748e-05 | 3.30446164171191e-05 | 3.19116201440629e-05 |
| pos\_2618 | 1,2-Di-O-myristoyl-3-O-(6-sulfoquinovopyranosyl)glycerol | 3.94796039663737e-05 | 4.60367103498759e-05 | 0.000115270658030935 | 9.09911195104772e-06 | 3.31320328123671e-05 | 3.85274315819764e-05 | 8.575384793412e-07 | 3.03082043374877e-05 | 2.16336170030226e-06 | 9.77294304411927e-05 | 3.34199538101225e-05 | 7.05686370405747e-05 |
| pos\_262 | BW 723C86 | 5.29718341543191e-06 | 1.15089462808637e-05 | 8.76940486664575e-06 | 2.42866917309858e-06 | 6.71890376398743e-06 | 7.21764851190075e-06 | 3.70372007221334e-06 | 5.35850879293618e-06 | 1.92562980724214e-05 | 5.52660466888584e-06 | 8.34414156533331e-06 | 9.160394007529e-06 |
| pos\_2622 | 4-(Hydroxymethyl)benzenediazonium(1+) | 0.000301122915944176 | 0.000334610066031765 | 5.09358593701699e-05 | 0.000171108038970938 | 0.000282023754933637 | 0.000232323787068686 | 7.60022149223299e-05 | 7.21170875134866e-05 | 0.000146576333712696 | 0.000184742180379443 | 0.000110759982737507 | 6.69713239542355e-05 |
| pos\_2623 | Rheinoside A | 2.88028209258431e-05 | 4.18844063703111e-05 | 7.16193214234321e-05 | 3.5607667955433e-05 | 2.10422733063127e-05 | 4.55713415067171e-05 | 3.43711433889431e-05 | 3.74434951532265e-05 | 4.74269460176737e-05 | 3.66137216362605e-05 | 4.50933348634262e-05 | 3.48417321583212e-05 |
| pos\_2624 | DL-2-Aminooctanoic acid | 1.29105223818325e-05 | 2.21595369282794e-05 | 1.92394513115872e-05 | 2.26391325460726e-05 | 2.04404752701184e-05 | 1.63053781715853e-05 | 2.9997695221891e-05 | 2.70632259402607e-05 | 2.18241762387119e-05 | 1.88293238177274e-05 | 2.60146335953122e-05 | 5.46515811999137e-05 |
| pos\_2625 | Tetrahydro-2-methyl-3-furanol | 3.38965178563492e-05 | 3.35535351822031e-05 | 1.7336716619337e-05 | 2.09899332849832e-05 | 2.10654924259202e-05 | 1.94186364673103e-05 | 1.65179104928299e-05 | 1.7316839467909e-05 | 3.73341580527283e-05 | 1.5388421559725e-05 | 2.32097308119941e-05 | 3.03795219176845e-05 |
| pos\_2626 | Allyl nonanoate | 6.40209351937474e-05 | 8.36187874880198e-05 | 9.42598079739901e-05 | 8.7718050943596e-05 | 8.95618197792322e-05 | 7.9381364461897e-05 | 8.84647790935073e-05 | 9.74057443504298e-05 | 8.25405157581504e-05 | 7.66855181207664e-05 | 8.36289457659447e-05 | 0.000100075013333925 |
| pos\_2628 | Oleoylcarnitine | 0.000990578007489836 | 0.00153512158798474 | 0.00111227557314106 | 0.00156725515392047 | 0.00160619741823269 | 0.00134394834851859 | 0.00161145334779814 | 0.00105287611824723 | 0.00169062274177355 | 0.0011898064156875 | 0.00118900454026606 | 0.000710389882104339 |
| pos\_2629 | Notoginsenoside R1 | 0.000106736907964084 | 9.47978906420778e-05 | 0.000110981550517934 | 0.000142592155097802 | 6.16759768151825e-05 | 0.000105304642803883 | 0.000117966865988552 | 0.000126611130812628 | 0.000103054366408321 | 9.52524693081828e-05 | 0.000122587134942757 | 9.66013190636712e-05 |
| pos\_2630 | 7-Isopropyl-5-methylbicyclo[2.2.2]oct-5-en-2-one | 9.0735262030785e-06 | 1.17828953639591e-05 | 1.16169174084233e-05 | 9.14186274452291e-06 | 7.54317534772037e-06 | 1.07582399208674e-05 | 8.95237181368622e-06 | 9.56139020191554e-06 | 1.04332037082986e-05 | 9.25131825026919e-06 | 8.82778111895881e-06 | 1.00956520996356e-05 |
| pos\_2631 | Melleolide H | 2.30563242279855e-05 | 3.30696507458647e-05 | 2.69157195299742e-05 | 2.84093221712121e-05 | 2.50196038190218e-05 | 2.31531840863414e-05 | 3.67169307804228e-05 | 3.44116380222525e-05 | 2.16764064761061e-05 | 2.21532788551299e-05 | 3.19941937061356e-05 | 2.75162460002868e-05 |
| pos\_2632 | PI(18:2(9Z,12Z)/22:2(13Z,16Z)) | 0.000242736553716839 | 0.000127993613486279 | 0.000206757574330761 | 4.59707180048228e-05 | 6.77917509716125e-05 | 7.63366805058419e-05 | 1.83558258379618e-05 | 6.21630219195337e-05 | 3.35508961242796e-05 | 0.000139143853087296 | 8.70005511265065e-05 | 9.73691241617493e-05 |
| pos\_2634 | 6-[2,3-Dihydroxy-1-(hydroxymethyl)propyl]-1,2-dihydro-7-hydroxy-9-methoxy-cyclopenta[c][1]benzopyran-3,4-dione | 6.85942659891695e-05 | 7.12554438325447e-05 | 0.000157101738795084 | 4.54871671921662e-05 | 6.92089360744615e-05 | 7.53648552958747e-05 | 4.14177190649448e-05 | 6.80410020010183e-05 | 4.43811632564306e-05 | 0.000168145755344103 | 7.65361113580924e-05 | 0.000135872160632909 |
| pos\_2635 | Spirolide D | 0.000735497689150831 | 0.000885037628982217 | 0.00124831077498261 | 0.000701529123659018 | 0.00078152828333015 | 0.000855200698303151 | 0.0008448629768549 | 0.000957382232122152 | 0.000738767890515888 | 0.00105828650129418 | 0.00114055808405621 | 0.00104620335622384 |
| pos\_2637 | Tipranavir | 0.000307847318545635 | 2.83102297684623e-05 | 3.87709221694153e-05 | 2.46489787782118e-05 | 0.000148216273471311 | 0.000131848498036633 | 0.000123446652809637 | 0.000105132297792452 | 5.13008155351633e-05 | 7.71592501840565e-05 | 8.6170557086701e-05 | 7.67465628904586e-05 |
| pos\_2638 | Geranylfarnesyl diphosphate | 0.000104479802849284 | 0.000103161691244092 | 0.000115603477869147 | 0.000135780975696915 | 0.000176031151039962 | 0.000162666416851554 | 0.000202226184318192 | 0.000162174198635727 | 0.000116691942639717 | 0.000172820799167028 | 0.000169186358071712 | 0.000137089719874055 |
| pos\_2639 | PI(16:0/18:0) | 6.59858776798191e-06 | 2.06580966015575e-05 | 1.66266239263864e-05 | 1.73741877442253e-05 | 1.22337188039572e-05 | 1.73340800737099e-05 | 1.32010547711839e-05 | 1.39182744598592e-05 | 1.92441132155473e-05 | 1.52296840627033e-05 | 1.7263408922975e-05 | 1.37459450502179e-05 |
| pos\_264 | Arg Ala Leu Ser | 9.39209893006896e-05 | 0.000172195417762958 | 9.45711092100057e-05 | 9.79025072350743e-05 | 8.17023198450347e-05 | 0.000102439239560057 | 0.000163550582245949 | 0.000133172041473016 | 9.93137394319781e-05 | 0.000102664490119037 | 0.000121507463335473 | 8.77813339692913e-05 |
| pos\_2640 | (N-acetylneuraminosyl(a2-6)lactosamine) | 4.41631182538893e-05 | 3.59247453845231e-05 | 0.000144462328205974 | 6.35109888428913e-05 | 4.19528294133412e-05 | 4.70401167481755e-05 | 4.0843001789911e-05 | 4.25217247570684e-05 | 8.71331664523238e-06 | 7.94373584834568e-05 | 4.37904136526941e-05 | 7.66244599806793e-05 |
| pos\_2641 | PE(16:1(9Z)/22:0) | 4.74885414338882e-05 | 5.63932061811834e-05 | 5.12399735493742e-05 | 9.60291665988755e-05 | 6.56294635957697e-05 | 5.16390600428672e-05 | 4.86388690435929e-05 | 5.6791117542612e-05 | 8.06640782742951e-05 | 6.47464361970101e-05 | 6.89321052546649e-05 | 5.32172757060938e-05 |
| pos\_2645 | (-)-cis-Rotenolone | 0 | 8.2050639769942e-08 | 0 | 3.25986399193232e-05 | 0 | 2.33092537320823e-07 | 2.30902371364624e-07 | 0 | 0 | 0 | 0 | 1.90169315902483e-07 |
| pos\_2646 | Methyl 2-(2-methoxy-4-hydroxyphenyl)-6-methoxy-3-benzofurancarboxylate | 2.1473814136239e-07 | 1.26488643608043e-07 | 0 | 0 | 3.13614350255049e-06 | 3.31326351726453e-08 | 0 | 2.86333602754474e-05 | 2.01573780944845e-06 | 6.33518831228592e-06 | 0 | 1.71312803512538e-08 |
| pos\_2647 | Cyanidin 3-O-(6''-acetyl-arabinoside) | 4.12373283574826e-05 | 6.31188206326908e-06 | 7.42789828496002e-06 | 7.199355451695e-06 | 2.39350656780255e-05 | 2.16252273483953e-05 | 2.11372401161284e-05 | 1.57997969096102e-05 | 1.13017621208025e-05 | 1.73773252703273e-05 | 1.4344279219683e-05 | 1.45640758222301e-05 |
| pos\_2648 | Carthamin | 0.00012121445642916 | 3.01233760572171e-05 | 2.8448237795361e-05 | 2.52107709618003e-05 | 6.74192299744451e-05 | 6.33159910017021e-05 | 6.42901611577918e-05 | 5.11391469502951e-05 | 3.46944124505787e-05 | 4.89124583990786e-05 | 4.69455500343075e-05 | 4.55963206648234e-05 |
| pos\_2649 | Chondroitin 4-sulfate | 0 | 0 | 3.48464569400877e-05 | 3.54884043708186e-07 | 0 | 1.1378019526312e-06 | 9.83165832460491e-06 | 6.49669213127219e-06 | 0 | 0 | 1.65806409012599e-06 | 6.60947797147066e-06 |
| pos\_265 | 4,10-undecadiynal | 1.84498451604234e-05 | 2.8621620908817e-05 | 2.80192440503271e-05 | 2.13823366129655e-05 | 2.06890096239768e-05 | 2.3500865631922e-05 | 2.24907344500729e-05 | 2.2686042603043e-05 | 2.38935197209312e-05 | 2.23156864107094e-05 | 2.45016436942106e-05 | 2.27434959616266e-05 |
| pos\_2650 | Glycerol 2-(9Z,12Z-octadecadienoate) 1-hexadecanoate 3-O-[alpha-D-galactopyranosyl-(1->6)-beta-D-galactopyranoside] | 0.000250295847544822 | 0.000420445125954866 | 0.000194235572555777 | 0.000323051222365757 | 0.000130968601078514 | 0.000235844857287646 | 0.00050941636491531 | 0.000292299192753149 | 9.20461839255092e-05 | 0.00039480061326193 | 0.000202315026556372 | 0.00019022846158256 |
| pos\_2651 | 15(R)-hydroperoxy-EPE | 0.000854415516490218 | 0.000349323941706794 | 0.000299952834332926 | 0.000301460495074293 | 0.000617066299828302 | 0.00061885582063819 | 0.00056542644263158 | 0.000510128035048921 | 0.000451799011558692 | 0.000442691312334165 | 0.000440422456438852 | 0.000505070749299198 |
| pos\_2653 | R-138727 | 8.74118821189913e-05 | 8.31779640302273e-05 | 9.20816220131921e-05 | 7.55333522440746e-05 | 8.03130889959435e-05 | 8.70799419885248e-05 | 6.32457325037639e-05 | 8.12991231525841e-05 | 9.72454164138909e-05 | 6.9127200495674e-05 | 8.9046502616249e-05 | 0.000100920520390995 |
| pos\_2655 | PC(DiMe(9,3)/DiMe(13,5)) | 0.000209491240677212 | 0.000110744668565213 | 0.000115652762437556 | 0.000162744898315917 | 0.000116395055275201 | 0.000142980270854587 | 0.000176215303193778 | 0.00015967574926165 | 0.000115921419163139 | 0.000127306810673621 | 0.000140589152615417 | 0.000152750049774572 |
| pos\_2656 | Scleroglucan | 2.73517560429772e-05 | 3.9879321323178e-05 | 2.66682373107605e-05 | 3.58879505493609e-05 | 3.23764675650897e-05 | 3.0216337107654e-05 | 2.99666405194484e-05 | 2.91575346765997e-05 | 3.40391347484604e-05 | 3.16096835518497e-05 | 3.00438743836575e-05 | 2.37633344554476e-05 |
| pos\_2658 | Methylphosphate | 0.000578490496146921 | 0.000751468867815889 | 0.000686997389941389 | 0.000610315962234195 | 0.000522305194515345 | 0.000600323859397811 | 0.000546184672238363 | 0.000625061802912379 | 0.00067711952252924 | 0.000566813404726513 | 0.000603938876021583 | 0.000583637079481425 |
| pos\_2659 | 3-Methyl-3-butenyl apiosyl-(1->6)-glucoside | 1.6687942304752e-05 | 1.87695587066218e-05 | 2.2890088471515e-05 | 1.57448194719578e-05 | 1.39241951684861e-05 | 1.69953251955841e-05 | 1.73517416758225e-05 | 1.98068937407565e-05 | 1.80986052173335e-05 | 1.71335967817895e-05 | 1.70378060212764e-05 | 2.02052921865816e-05 |
| pos\_266 | S-4-benzyl-3-((S)-3-hydroxy-2,2-dimethyloctanoyl)-5,5-dimethyloxazolidin-2-one | 3.5025093681202e-05 | 3.70373753786493e-05 | 3.53649400040863e-05 | 6.85821387717233e-05 | 5.61100079512384e-05 | 5.55314333858553e-05 | 3.20474926213124e-05 | 4.07470577611032e-05 | 6.32159212592189e-05 | 3.23829863899882e-05 | 3.90918663785988e-05 | 5.51761303215189e-05 |
| pos\_2660 | Isolinderanolide | 0.000967462485582657 | 0.00110541648391961 | 0.00107242445931698 | 0.000858095258630156 | 0.000708515080854387 | 0.000600578363215533 | 0.000635516823561436 | 0.000659991114355796 | 0.000788641314976542 | 0.000629645824067759 | 0.000646529558374018 | 0.000630607256323466 |
| pos\_2661 | Momordicoside I | 0.000395612262932945 | 0.000496605538321511 | 0.000313450431004373 | 0.000399153700741314 | 0.000277543925235139 | 0.000456756566066983 | 0.000327534312065011 | 0.000415349919449628 | 0.000441080580001209 | 0.000320531383090192 | 0.000431567090844708 | 0.000384022633304414 |
| pos\_2664 | DG(20:5(5Z,8Z,11Z,14Z,17Z)/22:2(13Z,16Z)/0:0) | 2.38167027049076e-05 | 1.84398491374838e-05 | 7.08022021074028e-06 | 2.01574251242166e-06 | 2.98101209907358e-06 | 2.21718040301903e-06 | 5.29651371832441e-06 | 4.67477487619647e-06 | 2.9259513437649e-06 | 1.8296156691307e-06 | 7.07684388219868e-06 | 7.82092913566065e-07 |
| pos\_2666 | 5-cis-8-cis-Tetradecadienoyl-CoA | 2.25816179426485e-05 | 3.69086356479264e-05 | 2.14079008022221e-05 | 3.46721718318787e-05 | 2.66322379984898e-05 | 2.70066225245455e-05 | 2.86027858278049e-05 | 2.56255202259439e-05 | 3.26263274464427e-05 | 2.84048094738919e-05 | 2.69756412159349e-05 | 1.6517448615957e-05 |
| pos\_2668 | Phlorisobutyrophenone 2-glucoside | 8.76122563693226e-06 | 7.48134759219656e-06 | 2.67016966884243e-06 | 5.81811611222635e-06 | 6.02120124704375e-06 | 5.60118185576896e-06 | 4.1049899929343e-06 | 3.73693943016811e-06 | 5.89513395617505e-06 | 5.59950540364434e-06 | 5.4732403522611e-06 | 4.04541942960719e-06 |
| pos\_267 | m-Xylene | 3.52006158895164e-05 | 5.23335311828754e-05 | 5.30412003122594e-05 | 4.91537432778951e-05 | 3.98196006621955e-05 | 4.45723839842207e-05 | 4.04303814118328e-05 | 4.56021542867809e-05 | 4.37950832166438e-05 | 4.266227443159e-05 | 4.39863886420075e-05 | 4.90105745935216e-05 |
| pos\_2670 | Rifaximin | 0.000664383610209987 | 8.42160624600345e-05 | 0.000114311410172359 | 8.81392759377841e-05 | 0.00035157291253267 | 0.000343766010276238 | 0.000298606064765979 | 0.000236395035313025 | 0.000157108735201252 | 0.000211121781657081 | 0.000232150193615406 | 0.000202003724167541 |
| pos\_2672 | MG(0:0/18:3(6Z,9Z,12Z)/0:0) | 0.000347591036710439 | 0.000327047892749177 | 0.000276709601904694 | 0.000290263742725009 | 0.000746542628201516 | 0.000372800589643363 | 0.00106388485444809 | 0.000628253401411172 | 0.000344121671354845 | 0.000470350224343736 | 0.000468582108813413 | 0.000701895877091643 |
| pos\_2675 | Zearalenone 4-sulfate | 4.3632997722374e-06 | 3.52920956615777e-06 | 1.37402536022573e-06 | 2.38734028853289e-06 | 5.4528186403506e-06 | 3.24184166962635e-06 | 3.60679759156445e-06 | 2.87507938755706e-06 | 3.29715460036219e-06 | 2.77001327735482e-06 | 3.39982967681533e-06 | 4.56218579414552e-06 |
| pos\_2678 | Blennin D | 1.31918634207039e-06 | 2.14326546184496e-06 | 2.55848402770987e-06 | 3.52656409122857e-06 | 2.1510783529691e-06 | 5.89731804892491e-07 | 1.20865846295757e-06 | 1.87114871317157e-06 | 2.35224291892581e-06 | 3.6245048736744e-06 | 2.21430256423033e-06 | 2.37320173489539e-06 |
| pos\_2679 | DG(15:0/18:1(11Z)/0:0) | 4.54613515502366e-06 | 3.94909371733196e-06 | 2.17980260279104e-06 | 1.71244690841797e-06 | 8.96601547492148e-06 | 4.32709509504909e-06 | 8.65804024294666e-06 | 5.67147200858763e-06 | 7.71415661843228e-06 | 4.89284666739301e-06 | 4.69301440429001e-06 | 2.94790874054022e-06 |
| pos\_268 | PYRITHYLDIONE | 0.00011982766901343 | 6.39763751329079e-05 | 3.18871000396651e-06 | 2.1986203658583e-05 | 6.90906509893135e-05 | 4.20297773678383e-05 | 4.31025476324088e-06 | 4.99413679820928e-06 | 4.55988558354288e-06 | 1.82894832277181e-05 | 6.83679418527155e-06 | 3.09099463268671e-06 |
| pos\_2680 | 6-beta-hydroxydexamethasone | 3.04336249590677e-07 | 4.27089542476816e-07 | 0 | 3.4891757908147e-07 | 1.05912516643588e-05 | 5.51692900533858e-07 | 1.99187614775294e-05 | 1.21241226726522e-05 | 8.3898028349497e-07 | 1.24724747560662e-05 | 3.77550264310477e-06 | 6.76562756098792e-06 |
| pos\_2681 | Dukunolide B | 4.5873176170116e-07 | 9.85811788674072e-06 | 5.09962743838088e-06 | 7.30728316696103e-06 | 2.65234352831484e-07 | 4.86733596780964e-06 | 1.13722696043115e-05 | 8.52349892362375e-06 | 2.2295255498568e-06 | 1.59304931081016e-05 | 1.05322851284378e-05 | 3.91297661717355e-06 |
| pos\_2682 | Prostaglandin E3 | 0.000314637145955568 | 0.000210318021447846 | 0.000194268113308641 | 0.000168513479029225 | 0.000200878103299634 | 0.0002190983089848 | 0.000226831081401648 | 0.000220218785068824 | 0.000217065656852394 | 0.000194407485916059 | 0.000203451193219657 | 0.000209418787965723 |
| pos\_2683 | PS(18:3(9Z,12Z,15Z)/20:4(5Z,8Z,11Z,14Z)) | 0.000144924149313717 | 0.000104516205444466 | 7.62692441852248e-05 | 6.54710034053828e-05 | 7.95974833690492e-05 | 7.95904891951302e-05 | 7.72145199376143e-05 | 7.82547233777296e-05 | 7.49308768797797e-05 | 6.9471930379464e-05 | 6.9784162097572e-05 | 7.60519348460574e-05 |
| pos\_2684 | Acetaldehyde 1,3-octanediol acetal | 1.32959056709472e-05 | 2.37163729669853e-05 | 1.92467238413807e-05 | 2.12018283675847e-05 | 2.37100982874683e-05 | 2.02730321387063e-05 | 1.87799887587994e-05 | 1.81014502508919e-05 | 2.04013553358833e-05 | 1.8630528573979e-05 | 1.91836129779367e-05 | 1.93042845933898e-05 |
| pos\_2687 | 4-Fumarylacetoacetic acid | 0.00483045090618742 | 0.00649952542840324 | 0.0059650011539632 | 0.00537362008631663 | 0.00452186451235938 | 0.0054059861972777 | 0.00470986126408356 | 0.00524925901606932 | 0.00586646343456515 | 0.00485459713529972 | 0.00535893149322621 | 0.0049486744857357 |
| pos\_2688 | 2-(2-Furanyl)-3-methyl-2-butenal | 4.37808503072985e-05 | 5.64780494996087e-05 | 7.63789950140104e-05 | 7.50874387685559e-05 | 7.90026233196409e-05 | 6.4701908402488e-05 | 7.29007246687391e-05 | 8.11250970930978e-05 | 6.47936981230335e-05 | 6.41350551911928e-05 | 6.64890503182846e-05 | 7.25193573768001e-05 |
| pos\_2689 | Cefpirome | 6.87255332169672e-06 | 4.3805407601974e-06 | 9.53287628307005e-06 | 0.000165476683121405 | 0.00013601448831174 | 5.8280356253001e-05 | 0.000147011533202541 | 8.04085788859977e-05 | 0 | 1.8011140064112e-05 | 5.22663357939627e-05 | 5.43667501441183e-06 |
| pos\_2691 | Dimethylbenzyl carbinyl crotonate | 6.22450684285582e-05 | 8.56848778082514e-05 | 8.58602720871774e-05 | 8.38735733867906e-05 | 9.9865033556633e-05 | 7.35128612100108e-05 | 7.66531508500714e-05 | 8.62545956023656e-05 | 9.03906021206777e-05 | 7.19241788813201e-05 | 8.54050969065992e-05 | 0.0001091795004353 |
| pos\_2693 | Neotussilagolactone | 0.000144972494973305 | 5.59483255585217e-05 | 5.36517112903416e-05 | 4.66540583499442e-05 | 8.87327611835317e-05 | 8.83894693141225e-05 | 8.8240456911477e-05 | 7.90008126640935e-05 | 6.33512657026564e-05 | 6.86673626307741e-05 | 7.11942812989598e-05 | 6.49598064122128e-05 |
| pos\_2695 | DG(15:0/16:1(9Z)/0:0) | 2.20114945477439e-05 | 0.000493410711688166 | 6.59144083768215e-07 | 0 | 3.04881512729099e-05 | 9.5764674885152e-05 | 6.31330312976895e-05 | 2.8990152426495e-05 | 0.000184968134744374 | 5.97669567914876e-07 | 6.28633342538826e-05 | 3.92611625692746e-07 |
| pos\_2696 | Pentacosanoic acid | 1.59194986978312e-05 | 1.88856104113249e-05 | 1.77878043034317e-05 | 1.92990353575966e-05 | 1.6249651743551e-05 | 1.74253910754013e-05 | 1.9285057284933e-05 | 1.88034457030805e-05 | 1.87327412518787e-05 | 1.84733693237962e-05 | 1.76392235637214e-05 | 1.73977583624714e-05 |
| pos\_2697 | (R)-Amphetamine | 0.000211418173873323 | 0.000184296311611197 | 0.000162240629710701 | 0.000154232668368434 | 0.000147133959220445 | 0.00017508325539394 | 0.000135865675885357 | 0.000171625844493471 | 0.000143991138303858 | 0.000173943751215438 | 0.000160941863615557 | 0.000212712438595186 |
| pos\_27 | Diosmin | 0.000122831587835226 | 0.000383195288763028 | 0.00184490460957996 | 3.60232992284665e-05 | 0.000113090662344688 | 0.000494362204270199 | 2.21728175291721e-05 | 0.00278271185820675 | 0.00267992519946483 | 0.000229135172321272 | 0.00105170988278017 | 0.000499242554222661 |
| pos\_270 | Lynestrenol | 7.02293313161923e-05 | 7.03819954154266e-05 | 0.000152114009272423 | 5.13006241911389e-05 | 7.81777603685293e-05 | 8.4419908232973e-05 | 4.38508199757414e-05 | 7.51266199375871e-05 | 4.73134739742588e-05 | 0.000241659899790611 | 9.51886245241142e-05 | 0.000110904992068656 |
| pos\_2700 | 7-Acetoxy-2-methylisoflavone | 8.64622045230975e-06 | 2.29489053126522e-05 | 9.98220216880113e-06 | 3.30014939150902e-06 | 2.42680149868707e-06 | 4.27042915357679e-06 | 1.207092649748e-05 | 3.96770776063662e-06 | 5.8133739623576e-06 | 1.73455417208618e-05 | 8.6950024015914e-06 | 2.05549207796143e-06 |
| pos\_2703 | LysoPE(0:0/20:3(8Z,11Z,14Z)) | 2.43711611547172e-05 | 4.38622878927207e-06 | 3.69840402137593e-06 | 3.97290601244684e-06 | 1.25489795455036e-05 | 1.02310253912232e-05 | 1.11208189877138e-05 | 7.27345733673055e-06 | 5.89810844651628e-06 | 6.93583834865524e-06 | 6.54544706348562e-06 | 7.87259586963359e-06 |
| pos\_2706 | Hexahydro-6,7-dihydroxy-5-(hydroxymethyl)-3-(2-hydroxyphenyl)-2H-pyrano[2,3-d]oxazol-2-one | 0.000466405761155769 | 0.000808578163036744 | 0.000547182627448363 | 0.000887053586872959 | 0.000590029417941922 | 0.000703469710907378 | 0.00051746234856388 | 0.000598726313946634 | 0.00128817777548864 | 0.000503187787318301 | 0.00093967967269753 | 0.000893031438279754 |
| pos\_2708 | Arecaidine | 5.08691225345983e-05 | 7.0578189331046e-05 | 3.46849568017501e-05 | 4.75088950988966e-05 | 5.30380979038144e-05 | 4.84091712109487e-05 | 4.98789603795512e-05 | 4.44049234832173e-05 | 5.35053483570593e-05 | 4.78924653409187e-05 | 4.66944753252659e-05 | 3.66330258651969e-05 |
| pos\_2709 | 7-beta-D-Glucopyranosyloxybutylidenephthalide | 0 | 7.09657521181548e-07 | 0 | 3.44085559238131e-07 | 3.60121160408886e-07 | 2.08721797527322e-07 | 9.61307531158691e-08 | 0 | 4.9347188802203e-07 | 8.0400694148534e-07 | 0 | 9.40632130717569e-05 |
| pos\_271 | 8,11,14,18-Eicosatetraynoic acid | 0.000457025537290329 | 0.000452455727347218 | 0.000510290085542277 | 0.000319563285546615 | 0.000273646249550384 | 0.000407026263554647 | 0.000124063015084409 | 0.000281753506518555 | 0.000137599826622595 | 0.000522297865305603 | 0.000291483242470037 | 0.000526616496348162 |
| pos\_2710 | Glycodiazine | 5.63635271632787e-06 | 6.30714124887327e-06 | 8.53310184589211e-06 | 1.42497367577021e-05 | 8.31783548806343e-06 | 9.77024428822896e-06 | 6.76072811685551e-06 | 7.00140937079487e-06 | 8.89443683951604e-06 | 9.05192936330824e-06 | 6.82673519766664e-06 | 6.55434106285101e-06 |
| pos\_2711 | (7'R)-(+)-Lyoniresinol 9'-glucoside | 3.34484071139057e-06 | 4.99680034201956e-06 | 3.93242188761917e-06 | 5.91112566499888e-06 | 3.46633678495013e-06 | 3.20564016017936e-06 | 5.3125520923536e-06 | 4.76591408964865e-06 | 4.91211040782187e-06 | 5.78463924529416e-06 | 4.90764556663515e-06 | 4.60801385377776e-06 |
| pos\_2712 | Capsianoside IV | 0.00215927681048513 | 0.00267564377796986 | 0.00243532561413788 | 0.0032660544839504 | 0.00239966783715559 | 0.00242457126650391 | 0.00213195982153504 | 0.00222675373925636 | 0.00177342837750843 | 0.00189341800964783 | 0.00216475594622591 | 0.00242878928105859 |
| pos\_2713 | Nigellic acid | 0 | 7.6135479030892e-06 | 0 | 0 | 3.70364043780711e-06 | 8.17851062817422e-07 | 4.25489803836538e-06 | 4.18864964507146e-06 | 7.45642739109551e-07 | 6.08187996428406e-07 | 3.98794982197952e-06 | 2.24995492332451e-06 |
| pos\_2714 | Tryptophyl-Aspartate | 1.72007986908107e-05 | 2.58025517956837e-05 | 2.25738196962507e-05 | 2.15413895254117e-05 | 1.75548883957284e-05 | 2.1297467718663e-05 | 1.77974008215437e-05 | 2.14068217396426e-05 | 2.3460118928116e-05 | 1.94895866175411e-05 | 2.23290652083053e-05 | 1.9091996542795e-05 |
| pos\_2715 | Ganglioside GA2 (d18:1/24:0) | 9.66651572225508e-05 | 6.08856888011354e-05 | 5.69026402383616e-05 | 5.77457908019314e-05 | 6.6490141213859e-05 | 7.25816153525943e-05 | 7.58191600355696e-05 | 6.77465592487378e-05 | 8.43017245480291e-05 | 6.37695235503733e-05 | 6.02205330808928e-05 | 6.74474997745636e-05 |
| pos\_2716 | 3,5-Diiodothyronine | 0.000723911467671707 | 0.000519942693955422 | 0.000456716957463282 | 0.000403761048010607 | 0.000409132808718183 | 0.000505253155317993 | 0.000505340258801171 | 0.00050295078539047 | 0.000498915730786082 | 0.000450219111435768 | 0.000476866096948965 | 0.000469779702373455 |
| pos\_2717 | dADP | 8.79535231667916e-06 | 1.75435138571152e-06 | 1.24789097791254e-06 | 1.53914112810364e-06 | 2.62715864446118e-06 | 1.92014291317243e-06 | 3.92341355289929e-06 | 2.62790474218414e-06 | 2.4163979808042e-06 | 3.23262768068682e-06 | 4.26480650072228e-06 | 6.29884169790701e-06 |
| pos\_2718 | triazolopyridinone epoxide | 1.46649281201516e-05 | 7.80797772597592e-06 | 4.16762313642079e-06 | 1.57555445473007e-06 | 2.06104885007301e-06 | 2.0816989160572e-06 | 2.57859641324757e-06 | 2.92001860014302e-06 | 0 | 4.56871729378806e-06 | 1.12540576358813e-06 | 2.43911524694663e-06 |
| pos\_2719 | Clofazimine glucuronide | 0.000747249732989348 | 0.000178406792191317 | 0.000199896336619856 | 0.000153490276212793 | 0.000428583288170584 | 0.000413209531835457 | 0.000379294416008829 | 0.000325499978701041 | 0.000243149621252812 | 0.000282599287259573 | 0.000311777520405938 | 0.000295833052428477 |
| pos\_272 | Geranylhydroquinone | 5.11542111239528e-05 | 6.33850608646717e-05 | 0.00010824666052773 | 8.17531334772085e-05 | 4.78709911534714e-05 | 7.09290528405119e-05 | 7.11602132212011e-05 | 8.48746960293324e-05 | 5.83816826724365e-05 | 8.61906263899058e-05 | 8.48333744691942e-05 | 0.000122233822325827 |
| pos\_2720 | Adenosyl cobyrinic acid a,c diamide | 0.000153667714809123 | 0.000155399863051336 | 5.23277734750838e-05 | 8.41087767271159e-05 | 0.000121434733008807 | 0.000126149448110565 | 0.000196730399809622 | 0.000153897385167027 | 0.000275889604839376 | 6.56242384712145e-05 | 0.000220864216104876 | 9.49008132162378e-05 |
| pos\_2721 | Carbadox | 8.30239319686937e-06 | 9.9054449050134e-06 | 6.85768412427439e-06 | 8.43674889221539e-06 | 7.1251175300446e-06 | 8.40012618054151e-06 | 7.06000867116265e-06 | 5.4870567810148e-06 | 8.06817703120555e-06 | 7.76031415368112e-06 | 7.4599920640364e-06 | 5.69842262915584e-06 |
| pos\_2722 | methyl-1,4-benzoquinol | 1.36802292905289e-05 | 3.28896387309961e-05 | 1.60477929085612e-05 | 1.0804947712029e-05 | 2.04949860573827e-05 | 1.76050708923788e-05 | 1.53021502291806e-05 | 1.5745156311542e-05 | 1.25543856822151e-05 | 2.60407793848442e-05 | 1.63205359383515e-05 | 1.48509025273222e-05 |
| pos\_2723 | Ethylcinnamate | 6.33429880918061e-07 | 4.25797072442395e-07 | 7.62149196845355e-06 | 1.27587487184048e-05 | 8.74481892476824e-07 | 3.57015550621708e-06 | 6.3799240531487e-07 | 4.3683518430479e-06 | 4.65354712931256e-07 | 8.02559786598736e-07 | 2.5494695047957e-06 | 1.29792363930132e-05 |
| pos\_2724 | (2S,3S,4R)-2-aminohexadecane-1,3,4-triol | 0.000577563811297232 | 0.000632328256703711 | 0.000795164966871898 | 0.000757451408994644 | 0.000723571721071616 | 0.000828642449212498 | 0.000878224954585378 | 0.000939991933968553 | 0.000761844693620069 | 0.000856735401603917 | 0.000733277591167959 | 0.000738956692393365 |
| pos\_2725 | Methyl succinate | 1.91606045702276e-05 | 1.5608299740946e-05 | 1.01914216616763e-05 | 1.29844090759315e-05 | 2.84346481980758e-05 | 1.31353758108753e-05 | 1.66330501317258e-05 | 1.47239462784082e-05 | 1.1616566124935e-05 | 1.89604410959452e-05 | 1.22190733825518e-05 | 1.84178350676873e-05 |
| pos\_2726 | Sorbic acid | 5.8069857317188e-06 | 6.0007016439007e-06 | 3.38439851025723e-07 | 2.64767465405844e-06 | 6.3111508135066e-06 | 4.25095002201264e-06 | 5.03961231750085e-07 | 6.41862472184432e-07 | 4.76183283719477e-07 | 3.49093648047599e-06 | 1.00734504411042e-06 | 4.98726197099223e-07 |
| pos\_2727 | OPC4-CoA | 5.38159104096222e-06 | 1.1743978527265e-05 | 4.19022335756091e-06 | 7.71706406657296e-06 | 1.3747054441161e-05 | 8.78863843328977e-06 | 5.11369748008449e-06 | 2.98383048499997e-06 | 1.45373992295246e-05 | 7.60321847809142e-06 | 7.55854345587177e-06 | 6.44572981113025e-06 |
| pos\_2728 | PG(14:0/18:1(11Z)) | 0.000884030850305768 | 0.000453274128850688 | 0.000412577834894319 | 0.000379295776847445 | 0.000439038225088093 | 0.000470777385045281 | 0.000498996411442122 | 0.000449785354371935 | 0.00045275372623239 | 0.000410107903503359 | 0.000441489412540393 | 0.000423258021304381 |
| pos\_2729 | Myrcene | 3.04380755922652e-05 | 7.67265915600289e-05 | 5.05613062423678e-05 | 6.42869828755269e-05 | 4.13102967851202e-05 | 4.43468707931651e-05 | 6.28532038630696e-05 | 5.84790194870374e-05 | 4.92579340219449e-05 | 4.13224521537171e-05 | 4.30403552245645e-05 | 5.39081953037593e-05 |
| pos\_273 | N-arachidonoyl glutamine | 0.000116649392258678 | 0.000128082946033019 | 0.000105904266827121 | 0.000148713364557041 | 9.89093569682492e-05 | 0.00011994361664337 | 0.000161377632647948 | 0.000142418926589389 | 0.000105037987459771 | 0.000130132702933533 | 0.000130195268806435 | 0.00011949442002986 |
| pos\_2730 | Histamine | 2.08830247284401e-05 | 2.80666512652216e-05 | 3.43938062690179e-05 | 2.04166788853323e-05 | 2.49810486023836e-05 | 2.65448643399368e-05 | 1.39124553549415e-05 | 2.57140463731412e-05 | 1.92114981963593e-05 | 3.73179033994834e-05 | 2.84116377631862e-05 | 3.42466870633396e-05 |
| pos\_2731 | 2-Dehydro-D-gluconate | 2.13280743649978e-06 | 2.71886080462635e-06 | 3.20797684876546e-06 | 3.0567035978232e-06 | 4.82574362768698e-06 | 3.08945909369549e-06 | 5.64031919967479e-06 | 3.60603925528613e-06 | 3.81425978599546e-06 | 4.91857068350647e-06 | 5.10912697626294e-06 | 6.69596731760669e-07 |
| pos\_2732 | Ethyl butanoate | 2.74393591434596e-06 | 3.36495919196954e-06 | 2.50690924972886e-06 | 3.21006228274556e-06 | 2.80290153500419e-06 | 3.14584119156963e-06 | 2.6971901309638e-06 | 2.51512970680046e-06 | 3.71325633838502e-06 | 3.2756050080796e-06 | 3.64498128316018e-06 | 3.35739447470041e-06 |
| pos\_2734 | L-histidinol phosphate | 4.9332346197701e-05 | 6.37253634403989e-05 | 6.02507914796559e-05 | 4.92439239846308e-05 | 4.65910736102414e-05 | 5.37698103274383e-05 | 4.90222895265381e-05 | 5.38263971639904e-05 | 6.35169760837989e-05 | 5.1277213379641e-05 | 5.56717445749945e-05 | 5.07149538151887e-05 |
| pos\_2735 | 7,8-Didehydrodesmosterol | 7.30951422286157e-05 | 0.000101723116164959 | 0.000105906065477977 | 0.000158870515659584 | 8.9077003158659e-05 | 0.000108943785582256 | 0.000173126184263709 | 0.000131177702951061 | 0.000109726610639009 | 0.000119632051228993 | 0.00013362199531977 | 0.000106259743866518 |
| pos\_2736 | 3-Deoxy-D-manno-octulosonate 8-phosphate | 1.11787067654593e-07 | 2.0557660451714e-07 | 0.000264990901862693 | 0.000874246788459787 | 5.27949206784895e-05 | 0.000241928640677327 | 5.43637655990883e-07 | 0.000185738169539449 | 0.00113929019213654 | 6.20833839541062e-06 | 0.000373917568601477 | 0.000466543507528774 |
| pos\_2737 | pteroyl-gamma-glutamyl-gamma-glutamylglutamate | 2.55023566498116e-06 | 1.81407312785405e-05 | 3.82579197181569e-06 | 8.68294569215599e-06 | 1.34625994547058e-05 | 4.35345449490217e-06 | 2.50137873279698e-06 | 5.17644536629934e-06 | 7.16512911523491e-06 | 4.13124895237234e-06 | 2.51325985665258e-06 | 2.84470680719737e-06 |
| pos\_2738 | alpha-methylmethionine | 7.01692126790736e-06 | 5.84960620482168e-06 | 1.21927172692882e-05 | 3.75154448542791e-06 | 4.09612521520271e-06 | 5.99969620604146e-06 | 3.0150753455252e-06 | 4.18636790032736e-06 | 3.21594112055432e-06 | 1.77972692718862e-05 | 5.59909634552628e-06 | 1.20255115005699e-05 |
| pos\_2739 | mercaptosuccinate | 7.41472452880871e-06 | 1.05059080241903e-05 | 1.06514462848218e-05 | 9.04274660167013e-06 | 7.38546449245958e-06 | 7.79804650609572e-06 | 8.42208252506972e-06 | 9.45062702815506e-06 | 9.93672248668454e-06 | 7.86258924474188e-06 | 9.70129618172322e-06 | 9.89993646302495e-06 |
| pos\_2740 | alpha-(2,6-anhydro-3-deoxy-D-arabino-heptulopyranosid)onate 7-phosphonate | 9.31547370473195e-05 | 0.000181990623097861 | 0.00011020009802691 | 0.000108341639390936 | 0.000152867049094075 | 0.000122448275293624 | 0.000177207239047486 | 0.000195445662167898 | 0.000159802819591472 | 0.000122688324547439 | 0.000156190624035131 | 0.000143290417064979 |
| pos\_2741 | 3-mercaptohexyl acetate | 4.17297148054925e-05 | 4.89691340668744e-05 | 4.48189612960984e-05 | 5.3986625312883e-05 | 2.83735583905414e-05 | 3.85804712448059e-05 | 4.83100942258723e-05 | 4.85846144096681e-05 | 3.55977111310652e-05 | 5.27891495428728e-05 | 4.68141057310578e-05 | 4.78643623876157e-05 |
| pos\_2742 | 2-furfurylmethylsulfide | 4.15559422790995e-05 | 5.95311536622335e-05 | 2.77199945359295e-05 | 5.18597063463288e-05 | 3.122721732735e-05 | 4.0023339010852e-05 | 4.7396506009263e-05 | 3.36746409586217e-05 | 3.59685937554747e-05 | 5.68644401643685e-05 | 4.16211327618602e-05 | 3.38709733584537e-05 |
| pos\_2743 | PG(16:0/16:1(9Z)) | 1.92900344349437e-05 | 1.44799779171011e-05 | 8.00304631230998e-06 | 8.24036774645244e-06 | 9.53619194453684e-06 | 8.79585445911474e-06 | 9.45999280065404e-06 | 1.09815415365435e-05 | 5.56080378375729e-06 | 9.71998841114174e-06 | 1.14759154844593e-05 | 1.94394921032513e-05 |
| pos\_2744 | L-Palmitoylcarnitine | 3.01857408900765e-06 | 2.49756824714511e-05 | 1.14327862702399e-06 | 1.7237365595549e-05 | 8.15409011901608e-06 | 1.08729473520126e-05 | 8.48687097153203e-06 | 7.26422528979486e-06 | 6.00768771412215e-06 | 9.6265663865168e-06 | 6.11827490560181e-06 | 5.02727019110176e-06 |
| pos\_2745 | polymixin B | 4.57254507925384e-06 | 1.46216777216542e-05 | 9.32893476585051e-06 | 1.13817966989591e-05 | 7.33513697698633e-07 | 7.59178387050274e-06 | 4.68948695429971e-06 | 1.20936344702148e-05 | 7.40985476435832e-06 | 4.40873426862649e-06 | 8.17335297218632e-06 | 6.66372956013287e-06 |
| pos\_2747 | Ethyl 3-hydroxy hexanoate | 6.80795857221563e-06 | 1.00916667571916e-05 | 6.35423360610188e-06 | 6.86114059619218e-06 | 5.23412510344005e-06 | 5.42488720210095e-06 | 5.71620746976197e-06 | 6.94649164002757e-06 | 8.94874104491402e-06 | 5.17728048755516e-06 | 8.18922910265912e-06 | 4.71415315295765e-06 |
| pos\_2748 | dodecaprenyl diphosphate | 0.000199016122636585 | 6.92700712122589e-05 | 6.50569490480825e-05 | 5.92099378360171e-05 | 7.97612269549017e-05 | 8.03539700324436e-05 | 8.96157906194326e-05 | 7.31820373014219e-05 | 8.02358092420645e-05 | 7.12720412177511e-05 | 7.85893589512968e-05 | 7.50878903814561e-05 |
| pos\_275 | Flaviolin | 7.40827571651746e-06 | 1.10287890028008e-05 | 1.52129243003989e-05 | 9.16815067362895e-06 | 5.6212701434774e-06 | 9.1466485766146e-06 | 8.80509665954345e-06 | 8.55621162354733e-06 | 1.05628334712507e-05 | 1.02420027877675e-05 | 1.02457544908686e-05 | 9.08686862403572e-06 |
| pos\_2752 | PA(16:1(9Z)/16:1(9Z)) | 2.0609277675357e-05 | 8.58325604312383e-07 | 1.47598399817303e-06 | 2.04386533698554e-07 | 7.63276912707867e-06 | 6.16551590503075e-06 | 8.82249623936538e-06 | 4.19015090174184e-06 | 1.81490194636022e-06 | 3.68241669867008e-06 | 4.41209690089811e-06 | 4.90952871583161e-06 |
| pos\_2753 | N-Acetyl-D-leucine | 6.03031852816888e-06 | 2.68565741517838e-05 | 3.04086072502673e-06 | 1.28052432731677e-06 | 1.41925983558903e-06 | 6.33411393739679e-06 | 1.69557458893837e-06 | 1.5475372225404e-06 | 2.52684985825369e-06 | 9.36056872543543e-05 | 1.43931630267056e-05 | 2.72365894218465e-06 |
| pos\_2755 | PC(14:0/16:1(9Z)) | 6.84014450656378e-05 | 2.87593767807755e-05 | 2.12610477792011e-05 | 8.67664449948989e-06 | 8.01302775200906e-05 | 2.65408353104735e-05 | 0.000116256817425742 | 9.16049420824484e-05 | 1.99133764829804e-05 | 5.83820118673346e-05 | 6.25137491833304e-05 | 7.36024015475985e-05 |
| pos\_2757 | 3-amino-1,2,4-triazole | 2.15205527164738e-05 | 3.02132599047813e-05 | 2.82428559769806e-05 | 2.25274429388188e-05 | 1.89540049520389e-05 | 2.51367712121653e-05 | 1.89541200389673e-05 | 2.22475110160323e-05 | 2.69948205629164e-05 | 1.97289860069739e-05 | 2.47715722171779e-05 | 2.12463151540937e-05 |
| pos\_2758 | IPC 18:0;3/20:0;1 | 0.000603369935150405 | 0.000284393283487294 | 0.000258534293119845 | 0.000226684618876298 | 0.000299471220902603 | 0.00032582743718205 | 0.000324042764401641 | 0.000284051910719001 | 0.000288453867764072 | 0.000274755597720049 | 0.000308106895232031 | 0.000275766430676921 |
| pos\_2759 | L-Acetylcarnitine | 9.35928582758496e-06 | 1.69541254068543e-05 | 2.58719591928231e-05 | 2.33738044931618e-05 | 6.76947183697749e-06 | 1.25714315957161e-05 | 1.94740019141223e-05 | 1.92684522195973e-05 | 1.34290941984434e-05 | 6.10745797251663e-06 | 7.85740141381344e-06 | 1.24543280891785e-05 |
| pos\_276 | Dihydrocoumarin | 2.46155291945635e-05 | 4.16300786759593e-05 | 1.66725822106403e-05 | 2.4423311017462e-05 | 3.20809509703335e-05 | 2.65367056146014e-05 | 2.21152206352762e-05 | 1.9229986090221e-05 | 3.86572759009248e-05 | 2.65508938461647e-05 | 2.57212581399028e-05 | 2.01067897356661e-05 |
| pos\_2760 | Myristoleic acid | 0.00016195764801317 | 0.000140548261416716 | 0.000116961791503783 | 0.00013141292521644 | 0.000108935039944358 | 0.000138578735213668 | 0.000119481565541221 | 0.000118566004777534 | 0.000103521633512794 | 0.000136153756599422 | 0.000120499319797595 | 0.000111210512498158 |
| pos\_2761 | 3beta-hydroxy-4beta-methyl-5alpha-cholest-7-ene-4alpha-carboxylic acid | 6.04505375612756e-05 | 4.231742721866e-05 | 3.74546656415774e-05 | 4.758377941847e-05 | 6.50211043413814e-05 | 5.12620781994116e-05 | 0.000269409130980949 | 0.000170789649323227 | 5.50293517233045e-05 | 4.4064966894535e-05 | 0.000126015847479443 | 6.17589094139949e-05 |
| pos\_2762 | N-amino DAP | 2.85703366762899e-05 | 4.65946916865864e-05 | 4.72295510148998e-05 | 4.41296877364402e-05 | 3.10825060467566e-05 | 3.67551754116518e-05 | 3.47608037833296e-05 | 3.74281903078208e-05 | 4.54323152046334e-05 | 3.04998273617385e-05 | 3.67274750529538e-05 | 3.22576847795281e-05 |
| pos\_2766 | CDPglucose | 3.17072854242329e-06 | 4.49583115942195e-06 | 6.87366637135611e-06 | 5.44201536588405e-06 | 2.86936513751557e-06 | 4.24252245964469e-06 | 4.30140293599615e-06 | 3.78604308488437e-06 | 3.26271169515821e-06 | 3.74744250251531e-06 | 4.58493243158321e-06 | 5.12046216294495e-06 |
| pos\_2768 | N-(5-Phospho-D-ribosyl)anthranilate | 6.11942663656145e-05 | 3.19835828753534e-06 | 7.59244005962561e-06 | 4.46499094796542e-06 | 3.49465768103949e-05 | 2.84114057402356e-05 | 2.19142343231384e-05 | 2.0294589505273e-05 | 9.52696460063664e-06 | 1.31712914278152e-05 | 1.5937865869504e-05 | 1.46006727136729e-05 |
| pos\_2769 | PC(16:1(9Z)/0:0) | 5.58109619344737e-06 | 0 | 6.08059699628525e-06 | 7.11745347429729e-06 | 1.20675804813102e-06 | 4.14015763622937e-06 | 0 | 2.25571146015454e-06 | 5.07999683008954e-06 | 0 | 1.76050382886483e-06 | 1.46043850038308e-08 |
| pos\_277 | Costunolide | 7.90813878739955e-05 | 8.9093440923743e-05 | 7.14921773258489e-05 | 7.85013663043598e-05 | 6.30075082344852e-05 | 7.70260213563818e-05 | 7.05796968551127e-05 | 7.71752049678965e-05 | 6.97731882267127e-05 | 7.17595403365244e-05 | 7.72874156418793e-05 | 7.1571895246678e-05 |
| pos\_2771 | {[(2S,3S,4R)-2-amino-3,4-dihydroxyicosyl]oxy}phosphonic acid | 0.000756474876346933 | 0.000841336934019672 | 0.00100771708905041 | 0.00118237019681098 | 0.000972279830942075 | 0.00088942986391038 | 0.000873039382716033 | 0.00100668275635439 | 0.00110174488151164 | 0.000960336804231982 | 0.000960576712030418 | 0.000996657222303634 |
| pos\_2775 | alpha-Isomethyl-ionone | 0.000293134449742319 | 0.00047655169561088 | 0.000465213792938142 | 0.000468170896520863 | 0.000344932450248036 | 0.000403766405702478 | 0.000364901754794339 | 0.000390803765290745 | 0.000398705692185861 | 0.000420023934720099 | 0.000401001521295014 | 0.000404494140673703 |
| pos\_2776 | vitispirane | 7.7339174692271e-05 | 8.58208568803379e-05 | 0.000153036518045403 | 0.000132188467666806 | 0.000195936653196919 | 0.00012225115190769 | 0.000101224999432687 | 0.000101861395799025 | 0.00015823056521131 | 0.000151901161675355 | 0.000124931098140931 | 0.000109784923269106 |
| pos\_2779 | CDP-DG(18:1(9Z)/22:3(10Z,13Z,16Z)) | 2.59209257354365e-05 | 2.87778025274585e-05 | 2.85263913545012e-05 | 2.28156300396053e-05 | 1.34521917642753e-05 | 2.30394713378518e-05 | 2.35710963138596e-05 | 2.95732288091607e-05 | 1.60653989024232e-05 | 1.67157068799438e-05 | 2.0547948057455e-05 | 1.95981603790493e-05 |
| pos\_278 | Maackiaflavanone | 0.000113877880404028 | 0.000118208588711647 | 0.000118923916430271 | 0.000103081433133425 | 8.75686950493904e-05 | 0.000107052725548561 | 9.01065577315253e-05 | 0.00010513708286559 | 0.000117486722914208 | 9.51579086551963e-05 | 0.000102122447226859 | 9.39350220594112e-05 |
| pos\_2780 | 4-deoxypyridoxine | 8.40027932782565e-06 | 1.17086616709048e-05 | 1.08197631559745e-05 | 9.40668273822107e-06 | 7.27089143622898e-06 | 8.9405090040736e-06 | 7.20398358225312e-06 | 9.97672665403604e-06 | 1.12393828553785e-05 | 8.45958915142494e-06 | 9.8692045449735e-06 | 1.03109708643704e-05 |
| pos\_2782 | Delta(6)-trans,Delta(8)-cis-leukotriene B4 | 8.05934475433336e-09 | 0 | 0 | 6.46998614865635e-06 | 9.98284626725042e-08 | 2.05233929644021e-06 | 7.10993554586885e-07 | 4.65873131456837e-08 | 2.37351068875632e-08 | 4.38172018263654e-06 | 5.81560767178638e-08 | 9.45412619500859e-09 |
| pos\_2784 | zymosterol intermediate 2 | 0.000433133067011865 | 0.000609013686443929 | 0.000334033062623358 | 0.000551704928471423 | 0.000595831936410389 | 0.000509622552836068 | 0.000831367802342218 | 0.000622241222433037 | 0.000570689374699338 | 0.000526935328089538 | 0.000620070477031532 | 0.000354517884909671 |
| pos\_2785 | Raffinose | 1.01476222583948e-05 | 6.81247857027674e-06 | 1.33926287613256e-05 | 1.30859352231756e-05 | 3.6759223272093e-06 | 4.97996798310628e-06 | 2.24567698361869e-05 | 5.18637288967372e-06 | 7.02491342258529e-06 | 7.12634179653923e-06 | 5.2199454294574e-06 | 1.5110485890227e-05 |
| pos\_2787 | PS(17:0/14:0) | 0.000125258070052911 | 4.19173289281369e-05 | 3.53479512078808e-05 | 2.79283390282336e-05 | 7.34878307191109e-05 | 6.84017379616876e-05 | 7.56917538334884e-05 | 6.24680010096138e-05 | 4.33757276921115e-05 | 4.98681815295248e-05 | 5.92093215604046e-05 | 5.78254458852496e-05 |
| pos\_2788 | 2-phenylethanol | 2.25678812381924e-05 | 3.01807233211187e-05 | 3.896096924463e-05 | 3.19448452784181e-05 | 2.49622862537771e-05 | 2.8042704009196e-05 | 2.56366262035881e-05 | 3.48185560264537e-05 | 2.31464328502683e-05 | 3.49188734865726e-05 | 3.3149988130366e-05 | 4.94668399049562e-05 |
| pos\_2789 | Purine | 8.0480258779396e-06 | 1.16732633323067e-05 | 9.2928454646283e-06 | 9.46266620891499e-06 | 7.79800707424424e-06 | 8.02408177508058e-06 | 8.35739915228656e-06 | 9.31245662076757e-06 | 1.01472741869316e-05 | 8.72355622461614e-06 | 8.90837291204389e-06 | 8.26154265658567e-06 |
| pos\_279 | SWIETENINE | 0.00114900308621982 | 0.000803101187826185 | 0.0008574299951002 | 0.000925945331740467 | 0.00112740535689989 | 0.00112551830644579 | 0.00180374941634016 | 0.00131907217601089 | 0.000904091260710648 | 0.00134770149088008 | 0.00141470900428277 | 0.00140859114426971 |
| pos\_2791 | NMNH | 1.03192884097109e-05 | 1.31905921142635e-05 | 1.2854001139997e-05 | 1.15681358593212e-05 | 9.51949515636633e-06 | 9.49893039184832e-06 | 1.14376651402226e-05 | 1.20951143120128e-05 | 1.07316361151993e-05 | 1.17605148813355e-05 | 9.99379477331988e-06 | 1.14933973180876e-05 |
| pos\_2792 | 2'-Deoxycytidine | 3.36049293825393e-06 | 9.12176553249598e-06 | 7.21356665568222e-07 | 1.0682689384728e-05 | 5.8536815220386e-06 | 5.84028084656219e-06 | 2.67506739930995e-06 | 3.82641307415612e-06 | 1.61514860545941e-05 | 4.49572971673688e-06 | 7.27755122504538e-06 | 6.90729420373725e-06 |
| pos\_2793 | Malvidin 3-O-(cafeoylglucoside) | 2.75989925882588e-06 | 4.25072787331808e-06 | 0 | 2.23985090929156e-06 | 3.52030418897124e-06 | 2.08288515179213e-06 | 2.03778901249464e-06 | 1.08018068655077e-06 | 3.34432381830817e-06 | 4.04745632108495e-06 | 3.15448660067244e-06 | 5.58064560075885e-05 |
| pos\_2794 | PE(18:1(11Z)/14:0) | 2.04266428390492e-05 | 1.14006117829884e-05 | 7.51011626179454e-06 | 2.4407107762944e-06 | 5.41064447304272e-06 | 5.5686258334173e-06 | 3.09696297525971e-06 | 1.83539068382186e-06 | 4.60201941900662e-06 | 1.93113722299448e-07 | 4.53759101464241e-06 | 3.28261011851109e-06 |
| pos\_2795 | Gamma-glutamyl-L-putrescine | 1.17029875441548e-05 | 1.20748738051294e-05 | 4.49486137497123e-06 | 1.15261180731596e-05 | 1.16790515333163e-05 | 7.41952096084156e-06 | 1.32185083263196e-05 | 9.26217945028988e-06 | 1.04174942401875e-05 | 1.29966021908469e-05 | 1.21674392509265e-05 | 1.25484319834336e-05 |
| pos\_2796 | undecaprenyl-N-acetyl-alpha-D-glucosaminyl-pyrophosphate | 8.33202635938762e-06 | 1.34156074305227e-05 | 1.10750904861883e-05 | 1.44349067888675e-05 | 1.03805595562519e-05 | 1.08152088970339e-05 | 1.27558522067274e-05 | 1.01974939704351e-05 | 1.66502236302485e-05 | 1.14402261667259e-05 | 9.15761140436514e-06 | 9.44739584703193e-06 |
| pos\_2797 | 9-[6(S),9-diamino-5,6,7,8,9-pentadeoxy-beta-D-ribo-nonafuranosyl]-9H-purin-6-amine | 0.000432214181345245 | 0.000273533088934704 | 0.000260145684946738 | 0.000247548387183039 | 0.000260609488007427 | 0.000299509073264358 | 0.00030896899824524 | 0.00028556554213307 | 0.000295091605187336 | 0.000263042303832377 | 0.000275881544251542 | 0.00027769653040449 |
| pos\_2799 | N-(24-Hydroxytetracosanoyl)phytosphingosine | 0.00147551501683089 | 0.00205821094532621 | 0.00189377427040587 | 0.00158016193519605 | 0.00140245965172438 | 0.00162736595940528 | 0.00145026063793072 | 0.00163247048420185 | 0.00192876548398897 | 0.00144045764734887 | 0.00160923581146045 | 0.00147548565531109 |
| pos\_28 | Cer(d18:0/13:0) | 5.98559743531411e-05 | 0.000132171448657631 | 7.60775565330198e-05 | 7.84389201739906e-05 | 5.70775276421746e-05 | 5.79468902279909e-05 | 6.30099329568608e-05 | 7.19349365385466e-05 | 6.20829557212219e-05 | 7.43997504737982e-05 | 6.87472048290182e-05 | 4.08656048377188e-05 |
| pos\_280 | Aspergillic acid | 2.06149734081913e-05 | 2.29988713564897e-05 | 4.44534822932771e-05 | 3.44922271103917e-05 | 4.23269895827761e-05 | 3.27309978191819e-05 | 2.0434081780338e-05 | 2.31370784574817e-05 | 3.72264438384525e-05 | 3.27740302353863e-05 | 2.64671854840425e-05 | 2.90302323702121e-05 |
| pos\_2800 | DG(18:0/18:1(9Z)/0:0) | 4.14197118142509e-06 | 1.6671805159175e-05 | 2.18521682612101e-05 | 2.39243166954391e-05 | 3.76803365541333e-06 | 9.53978864037908e-06 | 1.10076734115423e-05 | 7.76583228313465e-06 | 1.72447062969445e-05 | 7.53996194489536e-06 | 7.01034282862024e-06 | 1.00183040160281e-05 |
| pos\_2801 | PS(18:1(9Z)/0:0) | 0.000874883196018017 | 0.000808385440133148 | 0.000800026227927467 | 0.000778342568697492 | 0.000857159851532075 | 0.000836877670387435 | 0.00103359608066165 | 0.000872068416192902 | 0.000782785852517504 | 0.000888128149027762 | 0.000846297110074959 | 0.000908205440892715 |
| pos\_2802 | Ubiquinone Q1 | 4.80096796466203e-05 | 5.97214038509913e-05 | 7.35614080162364e-05 | 3.81768741110878e-05 | 4.91324728503188e-05 | 5.37945113675415e-05 | 3.04278553073407e-05 | 4.77819074095792e-05 | 3.95324239279907e-05 | 7.80834801634761e-05 | 5.49170846644489e-05 | 6.13888234058134e-05 |
| pos\_2803 | UDP-N-acetylmuramyl-L-Ala | 0.000178227290256739 | 1.50347923830312e-05 | 1.88967589919483e-05 | 5.79970047208917e-06 | 6.63905853464947e-05 | 5.23395108215116e-05 | 4.50948316729543e-05 | 3.22707872605439e-05 | 1.4403917642382e-05 | 2.6716770188453e-05 | 2.4688146267957e-05 | 2.62927215279678e-05 |
| pos\_2805 | UDP-N-acetylmuramoyl-L-alanyl-D-gamma-glutamyl-meso-2,6-diaminopimelate | 5.38975660549039e-05 | 1.25006349802034e-05 | 1.24623024475815e-05 | 9.93200601750163e-06 | 3.2526277869219e-05 | 3.19121036214198e-05 | 3.28678561904743e-05 | 2.17993134761355e-05 | 1.90120657892461e-05 | 2.61206464547064e-05 | 2.33796663837738e-05 | 2.20258092873434e-05 |
| pos\_2806 | Cyanidin 3-O-(coumaroylglucoside) | 0.00026442046901516 | 7.6803164981018e-05 | 8.20644734686307e-05 | 6.37261366613894e-05 | 0.00015914569041474 | 0.000153495790147459 | 0.000143346096778692 | 0.000130712767055356 | 0.000102339696570157 | 0.000104985541135283 | 0.000119623140384522 | 0.000108109203439431 |
| pos\_2809 | PS(17:0/16:1(9Z)) | 2.27229451419711e-05 | 3.92797101036321e-05 | 3.71454753373561e-05 | 3.32249116775676e-05 | 2.64065406207524e-05 | 3.13306932664506e-05 | 2.7739702042285e-05 | 2.94377760046672e-05 | 3.60439602707413e-05 | 3.11868098841446e-05 | 3.33881742236237e-05 | 3.12135761414214e-05 |
| pos\_281 | 6-methyl-heptanoic acid | 3.04374127287942e-05 | 3.43039844844823e-05 | 3.17824720961234e-05 | 3.15724407619479e-05 | 2.75592843564145e-05 | 3.03069476956085e-05 | 3.00730611519866e-05 | 3.10289632603351e-05 | 3.87221659309056e-05 | 3.04316759836738e-05 | 3.14736825135128e-05 | 2.72450888908428e-05 |
| pos\_2810 | 4-(4-hydroxyphenoxy)-3,5-diiodophenyl acetate | 1.221667425742e-05 | 7.51736715985374e-06 | 4.93175128352405e-06 | 7.1338277222366e-06 | 5.31502017102976e-06 | 8.51625736143115e-06 | 9.057059770196e-06 | 9.15004083617937e-06 | 6.06899194474677e-06 | 1.26381776691806e-05 | 8.40947204283678e-06 | 6.27921578889556e-06 |
| pos\_2811 | Cyclosporin A | 5.27222371214285e-05 | 2.96267681459464e-05 | 2.35331586594443e-05 | 2.39919628310557e-05 | 3.70524902751887e-05 | 3.61436853696632e-05 | 3.88982185431648e-05 | 3.06199784855619e-05 | 2.67847307589091e-05 | 3.34111684492552e-05 | 2.73365208863729e-05 | 3.18673521075915e-05 |
| pos\_2812 | Stearidonic Acid | 2.13013509374298e-05 | 2.49378317587598e-05 | 3.21187817042316e-05 | 2.59089516632285e-05 | 6.0501316463144e-05 | 3.01954953153377e-05 | 2.0421305047739e-05 | 2.66501369702845e-05 | 8.55559320883283e-05 | 3.46905798235878e-05 | 3.80927449925637e-05 | 3.57377106803129e-05 |
| pos\_2813 | Histidinyl-Cysteine | 1.64757572448517e-05 | 2.49021109207883e-05 | 3.11641571751395e-05 | 1.95239781394769e-05 | 1.39822202823169e-05 | 2.01503113751361e-05 | 2.14542190760907e-05 | 2.06457977291222e-05 | 2.32143009974573e-05 | 2.02910969015449e-05 | 2.49665388939515e-05 | 2.00233533636552e-05 |
| pos\_2814 | Glutethimide | 1.96353319419805e-05 | 1.06899198695569e-06 | 7.89243580319576e-06 | 1.61700569480695e-05 | 6.89821971155028e-07 | 8.92444963574717e-06 | 7.57621214046594e-06 | 1.69431027571937e-05 | 3.75189579235044e-06 | 1.24285203485527e-06 | 1.20427080101861e-05 | 3.88544864485091e-05 |
| pos\_2815 | Viloxazine | 7.30532840085533e-05 | 5.84971278589907e-05 | 4.40665582057343e-05 | 7.10186645021956e-05 | 7.15923443558943e-05 | 6.63315496669185e-05 | 8.55552708020682e-05 | 6.32190985666596e-05 | 4.9622654615533e-05 | 6.0488616637537e-05 | 6.3429826042325e-05 | 4.98927538171646e-05 |
| pos\_2816 | N6-Methyl-L-lysine | 1.14369569124877e-05 | 1.38883705685333e-05 | 1.59548038118263e-05 | 1.67798519490242e-05 | 1.81030335460371e-05 | 1.51200193945582e-05 | 1.40881369609968e-05 | 1.38401374895876e-05 | 1.52878758112305e-05 | 1.48130471822578e-05 | 1.53801008107608e-05 | 1.48210257760341e-05 |
| pos\_2817 | 2E-Eicosenoic acid | 6.89909950492116e-05 | 8.8187256521915e-05 | 9.3767075823223e-05 | 8.03437397313911e-05 | 7.15886150070403e-05 | 7.09343052625096e-05 | 7.21015024099684e-05 | 6.25526762937908e-05 | 7.92955004898139e-05 | 6.440542183071e-05 | 6.65169542785969e-05 | 6.41284362832968e-05 |
| pos\_2818 | C16 Sphingosine | 0.00235824385694157 | 0.00235263455369134 | 0.00198967333607334 | 0.00269144446945568 | 0.00171112266121827 | 0.00224540180128855 | 0.00240390475094754 | 0.00255838605457016 | 0.00211726016582463 | 0.00257618137716595 | 0.00240450867738198 | 0.00201991994975389 |
| pos\_2819 | Diethyl decanedioate | 2.25773783290523e-05 | 1.97864935545942e-05 | 2.22848921712028e-05 | 2.49044107899811e-05 | 1.5336523539208e-05 | 1.92531045595998e-05 | 1.87625375297194e-05 | 2.50435491740722e-05 | 2.01327059989706e-05 | 1.90928395342569e-05 | 2.22702058796621e-05 | 2.57030401078661e-05 |
| pos\_282 | 11,12-Dihydrolactaroviolin | 0.000136341113523015 | 0.000151756605262686 | 0.000140856146044068 | 0.000147455036959398 | 0.000123913406782345 | 0.000139579169801946 | 0.000131916991214025 | 0.000139029568140718 | 0.000134905302437447 | 0.000136685857202436 | 0.000136801703867772 | 0.000131894502772502 |
| pos\_2820 | 1,3-Dipropyl-8-cyclopentylxanthine [DPCPX] | 0.000111576349447508 | 7.38761043451011e-05 | 1.82938802125939e-05 | 5.97267321080274e-05 | 7.98718974633845e-05 | 6.38922409585642e-05 | 1.86523252585342e-05 | 1.89038472740544e-05 | 1.82915840133383e-05 | 5.54491861195574e-05 | 2.35011687175363e-05 | 2.31349307678286e-05 |
| pos\_2821 | Geldanamycin | 0.000705790781640949 | 0.000334160648613993 | 0.000249800055203588 | 0.000233891620129423 | 0.000502159708901208 | 0.000484628340482954 | 0.000497171767563199 | 0.000376278176939712 | 0.000342490328413615 | 0.000378534090483606 | 0.000386271815618004 | 0.000393377019234836 |
| pos\_2822 | dodecanamide | 1.93427893676954e-05 | 2.55594375094274e-05 | 2.35793160713346e-05 | 2.08992064881873e-05 | 1.82428972336352e-05 | 2.06027687527544e-05 | 1.81965812738461e-05 | 2.13007003685719e-05 | 2.36208550296787e-05 | 1.95969967680938e-05 | 2.13791712223969e-05 | 2.02136947449542e-05 |
| pos\_2823 | 5-propylideneisolongifolane | 9.51843540594402e-06 | 1.09469388164756e-05 | 2.92029214126703e-05 | 7.83459624310084e-08 | 1.660167659189e-05 | 1.26741020674042e-05 | 0 | 6.26605829246181e-06 | 2.78075768609119e-07 | 3.08777845380272e-05 | 1.34326461933003e-05 | 2.27940766926646e-05 |
| pos\_2824 | Desacetylvindoline | 1.43461433106537e-07 | 3.83481879494337e-08 | 2.5403889639727e-07 | 1.49621632551202e-07 | 6.44974156237776e-08 | 6.2791665405808e-08 | 1.74174797226003e-07 | 1.37232201415738e-05 | 2.17709691675066e-07 | 1.55929147055474e-07 | 9.20212031703006e-06 | 3.51694277848467e-05 |
| pos\_2825 | 1-Aminocyclohexanecarboxylic acid | 9.60520915279789e-05 | 0.000117437198936782 | 0.000119711809032517 | 0.000110689307948017 | 0.000122736919689441 | 0.000109725536284839 | 0.000101953712207757 | 0.000111069434695938 | 0.00010277681828228 | 0.000133991582620556 | 0.000111365521375697 | 0.000121557204813883 |
| pos\_2826 | 4-Nonylphenol | 0.000404861636509009 | 0.000483038131866026 | 0.000451138118940161 | 0.000521010997072993 | 0.000419229981918149 | 0.000464681186265599 | 0.000403326072004339 | 0.000446976676017693 | 0.000544688932606345 | 0.000509718748852966 | 0.000491376004129385 | 0.000457709032308074 |
| pos\_2827 | Benzoylecgonine | 8.48113315926854e-06 | 1.07266906527842e-05 | 3.21795724151174e-06 | 7.88721482162813e-06 | 6.29338873777651e-06 | 7.05835397769847e-06 | 7.50250978633416e-06 | 5.89234395268783e-06 | 5.41882426631068e-06 | 7.20380723476398e-06 | 5.98772605343459e-06 | 4.150216038149e-06 |
| pos\_2829 | Arginyl-Arginine | 0.000620840652116465 | 0.000677765022001631 | 0.00138842023726121 | 0.00013890540064727 | 0.000699938739642679 | 0.000713114348966563 | 5.39746412317889e-05 | 0.000461912803018387 | 0.000134727515684514 | 0.00155869468909286 | 0.00070034572253521 | 0.00112382510931959 |
| pos\_283 | Acutifolane A | 2.63286761178948e-06 | 6.91573875986977e-06 | 1.19961934734002e-05 | 1.04090641326352e-05 | 4.73762516991841e-06 | 8.08244248973827e-06 | 4.79776039593688e-06 | 7.52745548978734e-06 | 3.11865580436463e-06 | 3.9461042756494e-06 | 7.00352898846965e-06 | 1.55209983423398e-05 |
| pos\_2830 | Zearalenone | 2.68136503945575e-05 | 2.26663162761449e-05 | 2.73272731654938e-05 | 2.64676634481454e-05 | 2.16640067046812e-05 | 3.22165902487836e-05 | 2.14857013674773e-05 | 2.51010781987797e-05 | 2.37241796503673e-05 | 2.64958360432013e-05 | 2.81399892331598e-05 | 2.5412560070782e-05 |
| pos\_2831 | Veratramine | 0.000347108414104814 | 0.000349680961081118 | 0.000413046312744589 | 0.000467913455035196 | 0.000340880123747621 | 0.000387389437012218 | 0.000527770690235027 | 0.000769875561914112 | 0.000550019130196206 | 0.000331813841597337 | 0.000686325446601936 | 0.00118029347439198 |
| pos\_2832 | Ginsenoside Rg3 | 1.72342842147331e-05 | 1.83028072756596e-05 | 1.72587084539204e-05 | 1.7449753386355e-05 | 1.54543645074989e-05 | 1.98861565093231e-05 | 2.03332066637816e-05 | 1.57428275136947e-05 | 2.05009405939227e-05 | 1.72503121631803e-05 | 1.8996268587714e-05 | 2.07919761838658e-05 |
| pos\_2833 | Trihexyphenidyl N-oxide | 9.88496161586943e-06 | 8.90881998699478e-06 | 8.00947013632722e-06 | 1.25386963754141e-05 | 5.11821275846888e-06 | 1.11491779358064e-05 | 5.91154674073184e-06 | 9.50608106799001e-06 | 9.27866581194472e-06 | 6.27337982529305e-06 | 1.08614519398928e-05 | 1.03164679997589e-05 |
| pos\_2834 | Fungichromin | 0.000683168862796821 | 0.000371917673725052 | 0.000350107188128648 | 0.000303592089059952 | 0.000372501057526381 | 0.000398434271017866 | 0.000417012791866833 | 0.000377554942969083 | 0.000394993886307255 | 0.000347962656716343 | 0.000375509268407022 | 0.000376426023897228 |
| pos\_2836 | Adynerin | 0.000871537351586481 | 0.000380665908115182 | 0.00030694352440718 | 0.000304627460035737 | 0.000585561286341329 | 0.000591876000398917 | 0.000627737109101068 | 0.00047016029422734 | 0.000414204315507871 | 0.000473980674435659 | 0.000479874659003112 | 0.000819290349242416 |
| pos\_2837 | 5,6,7,8-tetrahydro-2-Naphthoic Acid | 3.31182879090538e-05 | 4.37010028570084e-05 | 3.4264929961903e-05 | 3.4676798625109e-05 | 3.68873665362316e-05 | 3.99865992109383e-05 | 4.93272229771575e-05 | 3.69811053540263e-05 | 5.44288110788318e-05 | 4.9877517998968e-05 | 5.14052482249149e-05 | 3.27162991793496e-05 |
| pos\_2838 | Nervonyl carnitine | 0.000963306836327923 | 0.00100774690398936 | 0.00134930707261919 | 0.00213549042139409 | 0.00147709381357821 | 0.00144615789536463 | 0.00146633467244745 | 0.00161344378764692 | 0.00121364810026974 | 0.00119046341029994 | 0.00149425616212448 | 0.00171020517196606 |
| pos\_2839 | Benzamide | 2.64689842187041e-05 | 4.52588609312893e-05 | 2.22845407435301e-05 | 2.59411231732962e-05 | 3.7462762831355e-05 | 2.88278731420781e-05 | 2.85817783421174e-05 | 2.43198105097024e-05 | 4.11445846255646e-05 | 2.67092783310875e-05 | 2.9856409622931e-05 | 1.71552291735583e-05 |
| pos\_284 | Pro Tyr Lys | 9.07112088761872e-05 | 0.000141315089837222 | 0.000124041904543127 | 0.000118697181288717 | 0.000110399427132565 | 0.000123519233932231 | 0.000123789284780909 | 0.000113990768190543 | 0.000151471308389706 | 0.00012181597794895 | 0.000138726856380071 | 0.000148457303146485 |
| pos\_2841 | Famciclovir | 6.25272210181293e-05 | 7.79301196029486e-05 | 2.39634942807885e-05 | 7.88257821141631e-05 | 4.20809602478822e-05 | 5.68241468993315e-05 | 4.37400872640401e-05 | 2.67037312642742e-05 | 1.32926293037135e-05 | 4.41060027706796e-05 | 3.71409500485301e-05 | 8.54379418460404e-06 |
| pos\_2842 | 13,14-dihydro-15-keto-PGF2alpha | 0.00020285797941797 | 0.000678875713312761 | 0.00129132475858992 | 0.00124503952781934 | 0.00065046747082747 | 0.000640603131769103 | 0.000345127975058815 | 0.000808139920061348 | 0.000744459057001593 | 0.000168396260195658 | 0.000246878136428936 | 0.000432602348770928 |
| pos\_2843 | d-Dethiobiotin | 5.54651866044626e-06 | 1.8027228507601e-05 | 1.59625278082279e-05 | 2.24963605127782e-06 | 4.62047854055239e-06 | 8.42353227821079e-06 | 3.59249311312444e-06 | 2.99355438010119e-06 | 4.92278047625333e-06 | 6.45670319756975e-06 | 2.06608504061412e-06 | 6.19199488865342e-07 |
| pos\_2844 | 6-Aminocaproic acid | 6.95520737875956e-05 | 9.14393466113684e-05 | 0.000119304672601133 | 9.39835697446357e-05 | 6.14799549726085e-05 | 8.6944639496807e-05 | 9.10874491445775e-05 | 0.000111335180288767 | 9.27676513203159e-05 | 8.45233223709531e-05 | 0.000106278938779019 | 0.000145915672409268 |
| pos\_2846 | Mestranol | 0.000321759256780673 | 0.000235965604281949 | 0.00021235629434014 | 0.000203611333088064 | 0.000198245248509717 | 0.000199289714692852 | 0.000211664476029677 | 0.000205001407854034 | 0.000193426213758076 | 0.000192434798420396 | 0.000189351268446962 | 0.000212018360593539 |
| pos\_2847 | Conessine | 2.27633981992979e-05 | 2.726435052971e-05 | 2.14522928749454e-05 | 2.35254767096789e-05 | 2.29027338363162e-05 | 1.95250348225065e-05 | 2.06918353084694e-05 | 2.18835274947915e-05 | 2.44606626768564e-05 | 1.93170336116306e-05 | 2.05889788070281e-05 | 2.09440658532089e-05 |
| pos\_2848 | Isoleucyl-Methionine | 0.000310383895180713 | 0.000515196485138234 | 0.000325973757482833 | 0.000310905434297933 | 0.00034893723109389 | 0.000338590492880795 | 0.000535417423450922 | 0.000476415471745051 | 0.000323701434948505 | 0.000323828089335385 | 0.000444095562639742 | 0.000396530931326576 |
| pos\_2849 | N,N'-Diethylthiourea | 4.57191061431665e-05 | 3.27646282094408e-05 | 2.7563981531705e-05 | 2.71272873591861e-05 | 3.39016443988473e-05 | 3.50875549470376e-05 | 3.42264745284002e-05 | 3.29152804930515e-05 | 3.39375196049308e-05 | 3.24402995721455e-05 | 3.25007766532936e-05 | 3.32725088779764e-05 |
| pos\_285 | (S)-gamma-Calacorene | 0.000199701374075458 | 0.000298152871970356 | 0.000371019693967157 | 0.000395900617056014 | 0.000271347788111204 | 0.000306141791435863 | 0.000301436638470756 | 0.000332196534821779 | 0.000246714500535525 | 0.000266153457638988 | 0.000307613963634516 | 0.000359596304588493 |
| pos\_2850 | Sinapyl alcohol | 2.93895229296932e-06 | 3.43483264302417e-06 | 1.65005767705157e-06 | 4.24618997800066e-06 | 2.93402333035816e-06 | 3.22845288581625e-06 | 2.07267745794393e-06 | 1.6094710083322e-06 | 2.52576615315859e-06 | 2.25276779062976e-06 | 1.88595495929847e-06 | 1.99334726831587e-06 |
| pos\_2851 | Myriocin | 0.000206981453957507 | 6.43436951003937e-05 | 5.68032388653147e-05 | 5.48074684796619e-05 | 0.000117421005487829 | 0.000108075711808795 | 0.000109497829330086 | 7.86397967252522e-05 | 7.78040698146072e-05 | 8.99301646365804e-05 | 8.61513642191227e-05 | 8.52479017516532e-05 |
| pos\_2853 | alpha-Tocotrienol | 0.0024280790023019 | 0.00203482501379061 | 0.00369900275489402 | 0.0197232520685724 | 0.0134590389663741 | 0.00925622689891784 | 0.00960513081089198 | 0.00643636972065964 | 0.00240452073646798 | 0.00324345824851021 | 0.00527761139273764 | 0.00169014949901359 |
| pos\_2854 | Fexofenadine | 5.44281638586318e-05 | 7.95182058548396e-05 | 0.000105755086514166 | 7.34937662420216e-05 | 6.1102224031123e-05 | 7.04938739768474e-05 | 5.08900416648102e-05 | 8.34957832535838e-05 | 6.3912497201173e-05 | 4.99645599404707e-05 | 6.39775054124072e-05 | 0.000115071226759096 |
| pos\_286 | 4-(2,6,6-Trimethylcyclohexa-1,3-dienyl)but-3-en-2-one | 0.000223869503777332 | 0.000260739605242808 | 0.000223623936807514 | 0.000231908072866112 | 0.000226263214335516 | 0.000231656214243772 | 0.000250450233844394 | 0.000249974214468431 | 0.000252942355857194 | 0.000282781261171758 | 0.000251825519588848 | 0.0002388889438579 |
| pos\_287 | 2-HYDROXYXANTHONE | 0.000264400463782323 | 0.000518449237712393 | 0.00208794794740726 | 0.000387454523698877 | 0.000242142284139392 | 0.000741646609703269 | 0.00127075494946957 | 0.00120363476726186 | 0.000395872357684471 | 0.00027281903983495 | 0.000901659502714648 | 0.0014879224805292 |
| pos\_288 | 3,4-Dimethyl-5-pentyl-2-furanheptanoic acid | 6.87438078150281e-05 | 6.60099505487511e-05 | 5.64668635721933e-05 | 6.93217300303439e-05 | 5.58933125441224e-05 | 5.62221583018675e-05 | 6.09053037687913e-05 | 5.77213120330672e-05 | 6.39502347061845e-05 | 6.54337008541722e-05 | 6.06271038550592e-05 | 6.75626060412414e-05 |
| pos\_289 | Vernoflexin | 1.65891429319965e-05 | 1.632930368599e-05 | 0.000195565165749351 | 0.0017318475627225 | 3.63064889537523e-05 | 0.000123447372634476 | 2.29113871264558e-05 | 0.000296263049367027 | 1.29790637655863e-05 | 1.69988242505502e-05 | 0.000169254842639847 | 0.000861924442614433 |
| pos\_29 | pentanamide | 0.000137361767333589 | 0.000158217190366615 | 0.000186684673904031 | 0.0001339624542894 | 0.000113444111046642 | 0.000145507031413381 | 0.000132561987975703 | 0.000159733591737369 | 0.000144020062008248 | 0.000155544283985574 | 0.000167761754245299 | 0.000147605881047961 |
| pos\_290 | ent-16beta-Methoxy-19-kauranoic acid | 0.000553154203844569 | 0.000539259341244687 | 0.000211763704543693 | 0.000286469980491555 | 0.000853387843347088 | 0.00047591924509413 | 0.000278662027719883 | 0.000306058086880735 | 0.000439037717779628 | 0.000526161590626772 | 0.000339038070092995 | 0.000297592228515113 |
| pos\_291 | Arg Gly His | 3.16337979091637e-07 | 3.63994247759155e-05 | 1.56725306932235e-07 | 8.0448636503087e-08 | 1.29007374641707e-09 | 1.70913653622587e-07 | 1.28248869101536e-07 | 3.58440561700191e-07 | 2.02972523278198e-07 | 3.63550029318045e-07 | 1.89126954731642e-07 | 1.98222053265381e-06 |
| pos\_293 | 5-Nitro-2-furancarboxaldehyde (2-hydroxyethyl)hydrazone | 5.0269455999357e-06 | 4.08872868501637e-06 | 3.5651957286917e-06 | 4.31750253417011e-06 | 5.63199608526506e-06 | 4.41659650822312e-06 | 6.1019066942461e-06 | 5.2474771573769e-06 | 6.69428246217586e-06 | 5.77591076000133e-06 | 5.12673728516006e-06 | 3.84633209519107e-06 |
| pos\_294 | ε-Caprolactam | 6.23379563821086e-05 | 6.79404793946435e-05 | 3.13496484088996e-06 | 2.78481921436138e-06 | 4.29618450065227e-06 | 1.9986739274558e-05 | 5.50423781443053e-06 | 2.72046265905986e-06 | 4.59162104057987e-06 | 3.23365002980898e-06 | 3.64085460030578e-06 | 2.87350269051946e-06 |
| pos\_295 | 3-(2-Heptenyloxy)-2-hydroxypropyl undecanoate | 0.000289338178295592 | 0.000304044026560969 | 0.000270874698121129 | 0.000353287713237636 | 0.000311765051221819 | 0.000293490069419141 | 0.00113427976830182 | 0.000473876099604433 | 0.000273728973574333 | 0.000423232092210875 | 0.000397255809098748 | 0.000578282378889457 |
| pos\_296 | Ichthyotherol | 7.13976921490219e-06 | 3.47021215523975e-06 | 8.7500378082579e-06 | 7.95851942409737e-06 | 4.25113825610881e-06 | 5.03854611541903e-06 | 3.30198316145187e-06 | 8.4599004881442e-06 | 2.98316369783971e-06 | 5.7375161166198e-06 | 3.03999269230402e-06 | 4.86260211006806e-06 |
| pos\_297 | 2',4',6',3,4-Pentahydroxy-3'-geranyl-5-prenyldihydrochalcone | 0.000434148532262969 | 0.000448204250643325 | 0.000437149332006349 | 0.000404175720934803 | 0.000332691520078428 | 0.000391788026876023 | 0.000340414984849113 | 0.000401926020496301 | 0.000497408943229768 | 0.000382362582189415 | 0.000388704454660659 | 0.000380583220612698 |
| pos\_298 | α-CEHC | 1.4294642826203e-05 | 2.02929370339018e-05 | 3.8009424427953e-05 | 1.37807434151741e-05 | 7.47192975115064e-06 | 1.56580705575613e-05 | 1.29378606719633e-05 | 1.70513839676952e-05 | 1.59567754304482e-05 | 1.58661235905848e-05 | 1.9858949953125e-05 | 1.24247491123733e-05 |
| pos\_299 | Bethanechol | 2.56306084400894e-05 | 4.33691679536198e-05 | 1.94883236795307e-05 | 1.67449414373579e-05 | 2.65996712424098e-05 | 2.5292221213448e-05 | 2.30890508757252e-05 | 1.84661207326432e-05 | 3.13733118810445e-05 | 2.68665774202928e-05 | 2.25250702919623e-05 | 1.24331387774723e-05 |
| pos\_3 | Cholic acid | 0.000681061777059528 | 0.000615213140507532 | 0.00120586526971254 | 0.00105297433801477 | 0.00225046425568565 | 0.00108457295217596 | 0.00119465163171093 | 0.000796271074644122 | 0.00096710284468086 | 0.00116871431554733 | 0.00082521305658184 | 0.00102876387521583 |
| pos\_30 | Pregnan-20-one, 17-(acetyloxy)-3-hydroxy-6-methyl-, (3b,5b,6a)- | 0.00412902946631151 | 0.00447544504282561 | 0.008370180016442 | 0.0103382007754085 | 0.0117677775601055 | 0.00707769967834939 | 0.00954302893789898 | 0.00968009861926303 | 0.00799448712499944 | 0.00602285827654647 | 0.00696238874177006 | 0.00566315561143653 |
| pos\_300 | Stearoylethanolamide | 0.000400881696891731 | 0.000432348304162443 | 0.000682278393120289 | 0.000579406146959451 | 0.000511629690406722 | 0.000498066511942338 | 0.000776695486994779 | 0.000611569412361917 | 0.000344884838489315 | 0.000474429465630802 | 0.000552606834945444 | 0.000327756019698545 |
| pos\_301 | Cycloviolaxanthin | 0.000369050316969034 | 0.000399437152898816 | 0.000336385075852386 | 0.000431463555666562 | 0.000339673917036701 | 0.000436228213177677 | 0.000373499708850124 | 0.000413519119022351 | 0.000427935820056485 | 0.00041750606732212 | 0.000439798345661491 | 0.000376042455784294 |
| pos\_302 | Ser Ala Ala Leu | 4.23877796841617e-05 | 5.64245717946983e-05 | 0.000146140916663578 | 2.80822364550842e-05 | 5.88305082485184e-05 | 5.70138448297839e-05 | 2.41267429108731e-05 | 4.14458441984896e-05 | 2.86846464874101e-05 | 0.00020540053867033 | 5.54979829635731e-05 | 7.4058226925473e-05 |
| pos\_303 | Methasulfocarb | 8.25927469432443e-08 | 0 | 1.38727691233709e-07 | 0 | 0 | 0 | 0 | 1.57243236978886e-05 | 1.24213401995263e-07 | 0 | 9.17956335371669e-06 | 3.649682643063e-05 |
| pos\_304 | pentyl-Benzene | 4.51902200352049e-05 | 5.40263362648714e-05 | 5.15899256853957e-05 | 4.94456882132097e-05 | 3.73044844431376e-05 | 4.40757730842688e-05 | 4.28262137924942e-05 | 4.88817276293957e-05 | 5.1692086390972e-05 | 4.38941654095796e-05 | 4.46625904360769e-05 | 4.12766044687833e-05 |
| pos\_305 | Ethyl 1-naphthylacetic acid | 9.55532989679281e-06 | 5.59717364292478e-06 | 2.98945190938638e-05 | 1.46923794680426e-06 | 6.32828492011731e-06 | 1.00073136320847e-05 | 9.43647137929113e-07 | 6.49290803441682e-06 | 9.49068020322265e-07 | 5.39565281979159e-05 | 9.58458034844637e-06 | 1.92581018700434e-05 |
| pos\_306 | Clerodendrin A | 0.0120507674866704 | 0.0205933479165707 | 0.0115776238388694 | 0.0138917223848389 | 0.0146156107107347 | 0.0134792150055096 | 0.0229652664940286 | 0.0199689962433109 | 0.0122883063319421 | 0.0113288402697543 | 0.017931693919227 | 0.015163411435472 |
| pos\_307 | (+)-Elaeocarpine | 6.10564422231261e-07 | 1.03607519991947e-06 | 5.32802963096979e-07 | 5.80157102585445e-07 | 2.65028113306758e-06 | 2.16611949864151e-07 | 1.07901540313886e-06 | 1.00510359533476e-06 | 8.13861697615259e-07 | 7.22253797695724e-07 | 6.24985274315519e-07 | 1.9908184441896e-05 |
| pos\_308 | 3,5-Dinitro-4-hydroxyphenylpyruvate | 1.34237647261533e-05 | 1.19689801242491e-05 | 7.50775250750602e-05 | 5.83650105798033e-05 | 2.60398686059775e-05 | 3.6676622408601e-05 | 6.71956663911386e-05 | 4.83251669657219e-05 | 1.84207866384053e-05 | 2.67101346313181e-05 | 4.03579935895008e-05 | 3.58913281546312e-05 |
| pos\_309 | Ile Ile Leu Trp | 0.000832866591521636 | 0.000449461224213505 | 0.000405130701965137 | 0.00038299946066305 | 0.000432566005685397 | 0.000487339506900576 | 0.000497514011727567 | 0.000512705692719816 | 0.000478088443164759 | 0.000412212589972818 | 0.00048689950568383 | 0.000591407276386653 |
| pos\_31 | 2-Linoleoyl Glycerol | 0.000561989528727867 | 0.000630987640626864 | 0.000519117710745463 | 0.000842749156825843 | 0.00188911741055201 | 0.000655413620473975 | 0.00977120173406569 | 0.00424189181160187 | 0.000547218107029077 | 0.00329012302650722 | 0.00173089468632442 | 0.00257660609928002 |
| pos\_310 | APHYLLIC ACID | 9.6696014883964e-06 | 5.67001802007564e-06 | 8.51142959772735e-06 | 8.21399283952853e-06 | 8.05947709061507e-06 | 6.04726346559018e-06 | 7.42873745363954e-06 | 8.9740518961635e-06 | 6.76742566732306e-06 | 7.84654677213207e-06 | 7.1729294602699e-06 | 9.46036584920788e-06 |
| pos\_311 | Cystophorene | 4.73260994421713e-06 | 5.39339756696086e-06 | 4.57158799423378e-06 | 4.4945035132473e-06 | 9.01350582426328e-06 | 4.84755146680013e-06 | 1.52956772434734e-05 | 1.05482999746714e-05 | 5.46666092858629e-06 | 1.11178732637353e-05 | 6.76454078752787e-06 | 8.3419296581653e-06 |
| pos\_312 | 1-(2,3-Dihydro-5,6-dimethyl-1H-pyrrolizin-7-yl)ethanone | 2.99102116680013e-06 | 1.33685535389428e-06 | 1.85234483149978e-06 | 2.00495331606937e-06 | 1.52955010838755e-06 | 1.9408035629611e-06 | 1.21671271966693e-05 | 5.33304628175138e-06 | 1.96446312837142e-06 | 2.87920695554998e-06 | 4.68129882774372e-06 | 1.31213926126176e-06 |
| pos\_313 | SCOPOLINE | 7.38147648295993e-05 | 0.000752265297190563 | 9.460875596408e-05 | 0.000576933358909931 | 0.000216284287107403 | 0.000193997878893388 | 0.000574752624901461 | 0.000368579974269957 | 0.00125099028596355 | 0.00016299077968461 | 0.000609422774438156 | 0.000151102127918644 |
| pos\_314 | DALBERGIONE, 4-METHOXY-4'-HYDROXY- | 4.13567734242507e-06 | 9.04739802051969e-06 | 4.79636226524331e-05 | 1.73240910821814e-05 | 6.66812896837766e-06 | 1.9293873757925e-05 | 1.94668884604236e-05 | 2.57464522759875e-05 | 4.54515535651958e-06 | 8.05590245875916e-06 | 2.13980578178222e-05 | 3.87809416480239e-05 |
| pos\_315 | Methylsainfuran | 5.00749452896426e-05 | 2.2514417413413e-05 | 0.000201893389948355 | 0.000157620463360899 | 1.42339743963792e-05 | 9.55421628413351e-05 | 2.9216103927737e-05 | 0.000123070688728872 | 3.86106315832869e-05 | 8.37524168224232e-05 | 0.000112170800295893 | 0.000289192509010926 |
| pos\_316 | Trandolaprilat glucuronide | 0.000378964462603139 | 0.000514647197554584 | 0.000242600675603022 | 0.000238249540952636 | 0.000580233210577376 | 0.000390415777828668 | 0.00029916565468293 | 0.000367260564021639 | 0.000415916051121613 | 0.000211670824144788 | 0.000320718507783913 | 0.000468957721864574 |
| pos\_317 | Pro Pro Thr Thr | 1.64932354087099e-05 | 2.33889033659177e-05 | 1.87448531565871e-05 | 1.84031061144171e-05 | 1.19346401654263e-05 | 1.80685799760925e-05 | 1.75973793581748e-05 | 1.83381519265846e-05 | 1.64593435362383e-05 | 1.34153207077493e-05 | 1.51845975498153e-05 | 1.80157746261369e-05 |
| pos\_318 | 16-Oxosteroid | 5.84471212392977e-05 | 5.2527217109423e-05 | 5.12361664600679e-05 | 5.52377955447115e-05 | 5.58964120848986e-05 | 4.97953505966624e-05 | 5.85343729178849e-05 | 5.65892310183433e-05 | 5.34469776293902e-05 | 5.25612690047369e-05 | 5.2664446353247e-05 | 5.58819785468395e-05 |
| pos\_319 | Cammaconine | 9.29713937135203e-06 | 3.41399152479275e-05 | 4.43188079892796e-05 | 4.25423384614352e-06 | 1.13848545446515e-05 | 2.03525354301203e-05 | 9.72464619751115e-06 | 0.000153167051033456 | 6.32656318605315e-05 | 7.99298974913827e-05 | 0.000106959034803383 | 0.000365089008215312 |
| pos\_32 | ANETHOLE | 8.51394166529041e-05 | 6.50961170951931e-05 | 7.09271955386828e-05 | 4.79576467433966e-05 | 5.9895689763784e-05 | 6.37413965156816e-05 | 5.71138131132841e-05 | 6.11596599510956e-05 | 6.02747269551464e-05 | 7.03213075291111e-05 | 6.47294028567253e-05 | 7.16542450719265e-05 |
| pos\_320 | 3,6-Dihydronicotinic acid | 1.77350681159829e-05 | 2.66839489006737e-05 | 2.19852704424417e-05 | 3.42374114559091e-05 | 1.86869366654581e-05 | 1.63245291492181e-05 | 2.55992044289946e-05 | 2.67821847633612e-05 | 2.47927952556116e-05 | 3.17452174303325e-05 | 2.11432103269056e-05 | 1.65805364022689e-05 |
| pos\_321 | Pro Ile Leu Ile | 0.00699427692401941 | 0.00633282176521621 | 0.00675468431022208 | 0.00801961672800048 | 0.00556911675691724 | 0.00659965807494753 | 0.00891936001060466 | 0.0100286944609159 | 0.00624102113313941 | 0.00730632563897749 | 0.00874545618890723 | 0.0119955564809833 |
| pos\_322 | Met Trp Arg Glu | 0.00558069484515237 | 0.0104269599237355 | 0.00798591979178407 | 0.00495609251665075 | 0.00646931439773849 | 0.00543606110048982 | 0.0101651686803005 | 0.00909397161880915 | 0.00534072600277927 | 0.00581221109331251 | 0.00839490794538671 | 0.00783919706508203 |
| pos\_323 | 4-Nitroanisole | 2.06785130904332e-05 | 1.97963370747351e-05 | 9.69678839650556e-06 | 1.36480650299038e-05 | 1.5603146612349e-05 | 1.37630749599685e-05 | 1.71772442786919e-05 | 1.46155007487101e-05 | 1.5059487031452e-05 | 1.36673453425371e-05 | 1.53542724647789e-05 | 1.62280558820418e-05 |
| pos\_324 | (20S)-1α,20-dihydroxy-22,23,24,25,26,27-hexanorvitamin D3 / (20S)-1α,20-dihydroxy-22,23,24,25,26,27-hexanorcholecalciferol | 1.58717285920674e-05 | 2.03398968198083e-05 | 1.64318212012648e-05 | 3.36679395320108e-05 | 2.49540735043603e-05 | 2.71797417933747e-05 | 2.36085999536146e-05 | 1.8151476562531e-05 | 1.46302152567096e-05 | 1.77672043857763e-05 | 2.09461435010491e-05 | 1.50846115552385e-05 |
| pos\_325 | Dibutyl sulfide | 7.04251548292102e-05 | 9.6205268211153e-05 | 9.95639776914305e-05 | 0.000102251786349887 | 8.67346625380842e-05 | 9.13360289229461e-05 | 0.000107425798164475 | 0.000101063802886481 | 8.65818600110105e-05 | 0.000100412109498915 | 9.39987381566299e-05 | 0.000102828570590378 |
| pos\_326 | 1-Naphthol | 1.17429509656659e-07 | 0 | 1.3930484872148e-06 | 6.0686579969595e-07 | 1.62932727931556e-06 | 4.46749425977303e-07 | 1.13739870203969e-07 | 2.73554464327753e-07 | 7.301337955824e-07 | 8.24812643800734e-07 | 3.16588042150543e-07 | 5.48908607472166e-07 |
| pos\_327 | PGF2α 1,15-lactone | 2.0161709735204e-05 | 2.84286827836909e-05 | 7.17031135174287e-06 | 0.000102340134245985 | 6.42896060951603e-05 | 4.17036822382774e-05 | 5.26867355483134e-05 | 3.20898405449011e-05 | 1.05685361481896e-05 | 1.30003838148115e-05 | 2.71520481862076e-05 | 1.13875474158367e-05 |
| pos\_328 | Cys Cys Phe Asp | 0.00030825588871888 | 0.000360109932737682 | 7.0713815613665e-05 | 0.000355006480008446 | 0.000198503671816442 | 0.000233584807488466 | 0.000210921984165038 | 0.000131901482344033 | 3.79982613309924e-05 | 0.000468321069603532 | 0.000179555354544677 | 5.22386821434339e-05 |
| pos\_329 | Met Arg Asp Trp | 0.000454366226968635 | 0.000391989645412172 | 0.000389239592760065 | 0.000270392383057386 | 0.000394442129227395 | 0.000393704202348313 | 0.000333087772510945 | 0.000378427180671882 | 0.000310666850020709 | 0.000382074321428893 | 0.000351796452072241 | 0.000549981568530541 |
| pos\_33 | 7-Hydroxy-2-methylisoflavone | 0.000138002575468653 | 0.000270438748441505 | 0.000120629867885539 | 0.000152582205505102 | 0.000244024498072692 | 0.000172994954005316 | 0.000196032106298725 | 0.000182340706114034 | 0.000226858791480002 | 0.000165022806741012 | 0.000196864163263195 | 0.000204367728890314 |
| pos\_330 | decoquinate | 1.33536090680343e-05 | 3.49565490365866e-05 | 8.27989764141246e-06 | 1.34885058876579e-05 | 1.57895335472947e-05 | 1.47655748738065e-05 | 1.0528180282213e-05 | 1.18918774090614e-05 | 1.84949470593839e-05 | 1.82484828409207e-05 | 1.48295068561098e-05 | 1.59613856212922e-05 |
| pos\_331 | PE(22:2(13Z,16Z)/18:4(6Z,9Z,12Z,15Z)) | 0.00040987633942936 | 0.000329833051640469 | 0.000305744263509701 | 0.000269751649485571 | 0.000292610885293011 | 0.000329467556581558 | 0.000325669130671682 | 0.000310476579150677 | 0.000332051070646799 | 0.000294387000965571 | 0.000321357526322382 | 0.000309440833195217 |
| pos\_332 | Thr-Ser-OH | 3.05353734830962e-06 | 6.03991970822285e-06 | 2.27818669486772e-06 | 3.2305356572852e-06 | 3.38509579980247e-06 | 1.88564978096068e-06 | 9.97624990809003e-06 | 6.49242839736574e-06 | 3.87818224437326e-06 | 1.88209762352937e-06 | 4.5739452599673e-06 | 3.05224126648463e-06 |
| pos\_333 | CETRIMONIUM | 0.00812769576346074 | 0.00292440914013126 | 0.00378367856611616 | 0.00252970335727539 | 0.00389913866487585 | 0.004061997050846 | 0.00406032435767216 | 0.00376283583362314 | 0.00326986642360162 | 0.00328275615814251 | 0.0041477567821256 | 0.00321572271353903 |
| pos\_334 | 1-(Methylthio)-1-butene | 2.23497646473938e-06 | 2.63490844739216e-06 | 3.12159720854427e-06 | 2.74229144746219e-06 | 2.80573001630819e-06 | 2.96339360087566e-06 | 2.92280503450837e-06 | 3.21128212595431e-06 | 2.52455387783001e-06 | 2.9440334043896e-06 | 2.87780817215461e-06 | 2.77445202321873e-06 |
| pos\_335 | 2'-Methoxy-4'-hydroxydihydrochalcone | 2.73835497722197e-05 | 3.26954590094984e-05 | 1.68201118317735e-05 | 2.37437029481335e-05 | 2.29041959555763e-05 | 2.33250637535294e-05 | 2.3719366581582e-05 | 1.77191365615712e-05 | 2.25707960012124e-05 | 2.73256688635317e-05 | 2.18505422793903e-05 | 1.7051985503931e-05 |
| pos\_336 | MG(0:0/18:3(9Z,12Z,15Z)/0:0) | 0.00104726669054364 | 0.000744438426719033 | 0.000374476224192914 | 0.000626012407828394 | 0.000874108600642789 | 0.000767159283377265 | 0.000519659544161642 | 0.000683440535015957 | 0.000725542160193879 | 0.000596695409900136 | 0.000662812230129225 | 0.00085523004644435 |
| pos\_337 | Ethyl cinnamate | 7.88058635301444e-05 | 6.7386053146826e-05 | 9.44260592928616e-05 | 9.02642776222631e-05 | 0.000106437127584103 | 8.91996009585216e-05 | 8.49778803562542e-05 | 8.41377824630067e-05 | 9.64444384634324e-05 | 8.59180764585961e-05 | 8.57403265962281e-05 | 8.59541728209972e-05 |
| pos\_338 | PG(P-16:0/20:1(11Z)) | 0.00127646598224361 | 0.00145218059259737 | 0.00142399856485821 | 0.00151285475161768 | 0.00156136705024009 | 0.00136280111746217 | 0.00150882346993902 | 0.00137154355696821 | 0.00176139958060797 | 0.00152097502615265 | 0.00148939537552653 | 0.00141517391183258 |
| pos\_339 | Ser-TyrMe-OH | 4.40927045390849e-06 | 5.03170204619579e-06 | 8.30887307603543e-06 | 1.00084771375939e-05 | 4.38903438533133e-06 | 5.26366357171447e-06 | 6.64007715634311e-06 | 8.02662267135548e-06 | 1.54642417432594e-05 | 7.43668877636308e-06 | 8.60451549158566e-06 | 6.98708861330526e-06 |
| pos\_34 | p-Cymene | 5.85257686358482e-05 | 5.94192399916295e-05 | 6.73484100365482e-05 | 6.25190567239409e-05 | 4.98630313112285e-05 | 5.80998775265359e-05 | 5.63789082107774e-05 | 6.61826200082541e-05 | 6.02542286633809e-05 | 6.72399293805283e-05 | 6.33443684050659e-05 | 6.15079520922165e-05 |
| pos\_340 | Norchelerythrine | 2.67274942059776e-06 | 1.08011694625764e-05 | 2.69469706878023e-05 | 1.78111673324469e-05 | 1.24616294460533e-05 | 1.46620134766884e-05 | 2.60189936031034e-05 | 2.85088338395626e-05 | 3.25493897258705e-05 | 2.18809219421123e-05 | 8.16324112558009e-05 | 3.74389114702975e-05 |
| pos\_341 | Isoelemicin | 0.00036502369672636 | 0.000238334217484536 | 0.000226191861858413 | 0.000202625915645116 | 0.000236559429662607 | 0.000244143394629588 | 0.00025202609875931 | 0.000236844467021555 | 0.000246989268472235 | 0.000226118329519591 | 0.000235474468743734 | 0.00023496512064823 |
| pos\_343 | Thr Glu Met | 0.00146322924043247 | 0.000645016721393939 | 7.62837759084766e-05 | 0.000124274702751089 | 3.11061638099424e-05 | 0.000435086229160305 | 4.22374232857493e-05 | 7.96511160316536e-05 | 0.000286184645787942 | 0.000458610431015023 | 0.000233885568025901 | 0.000136142787523297 |
| pos\_344 | 1,3,6,8-Naphthalenetetrol | 4.04955251553622e-05 | 7.25529701515681e-05 | 3.66321405509645e-05 | 5.13070313439172e-05 | 4.70387001297091e-05 | 4.90071786821265e-05 | 4.23579595691291e-05 | 4.66079941362217e-05 | 7.80712891269402e-05 | 4.18143207343164e-05 | 5.15893027254388e-05 | 4.818767770093e-05 |
| pos\_345 | alpha-Bergamotene | 0.000145857473839306 | 0.0001822105078544 | 0.000176865147061464 | 0.000175042432681358 | 0.000144784619610717 | 0.000160999498834943 | 0.000187472713355883 | 0.000185355355368358 | 0.00016261476956602 | 0.000186873391775777 | 0.0001741445922964 | 0.000176071738473985 |
| pos\_346 | 1-Hydroxy-6-methoxypyrene | 0.00176967773074051 | 0.00469813868161192 | 0.00150562097727423 | 0.00198141393231628 | 0.00351400261262185 | 0.00260291915038488 | 0.00228004912632698 | 0.0018881271051375 | 0.00406774926100364 | 0.00192282501051613 | 0.00241143348071113 | 0.00151402607675513 |
| pos\_347 | 4'-HYDROXYCHALCONE | 0.000111256945840614 | 0.000291153796174013 | 0.000106228287297212 | 0.000146008241712455 | 0.00021876391581898 | 0.00016435849913891 | 0.000166484734737378 | 0.0001370927758193 | 0.000266178173765838 | 0.000131864611770264 | 0.000164430462330693 | 0.000104712610873746 |
| pos\_348 | 8-C-alpha-L-Arabinosylluteolin | 9.54221105847798e-06 | 6.52084671019422e-06 | 2.82402911820171e-06 | 4.68866044612796e-06 | 9.86785468925039e-06 | 7.20003182571608e-06 | 8.73001094704883e-06 | 5.67152705563953e-06 | 7.27338290253797e-06 | 5.26291447622394e-06 | 7.11138496330018e-06 | 9.0578196458822e-06 |
| pos\_349 | Propamocarb | 9.36891139046527e-05 | 0.000135386621388468 | 0.000176537328446951 | 0.000153561320158193 | 0.000147224564546668 | 0.000141709458043072 | 0.000171773608446534 | 0.000180903903665479 | 0.000136697929823718 | 0.000145567768790424 | 0.000153760059947193 | 0.000180631504080656 |
| pos\_35 | 3α-Hydroxy-5β-chola-7,9(11)-dien-24-oic Acid | 0.00150084084769481 | 0.00168175171872051 | 0.00283687715996448 | 0.00335727793703856 | 0.00385699939608826 | 0.00244607124693061 | 0.00324754957163531 | 0.00334108070387393 | 0.0027498166560256 | 0.00218700246801354 | 0.00245065120677606 | 0.00212452290416144 |
| pos\_350 | (23R)-23,25-dihydroxyvitamin D3 / (23R)-23,25-dihydroxycholecalciferol | 0.000610504807208111 | 0.00072393870131201 | 0.000363585595653199 | 0.00110655336690553 | 0.000686327719033741 | 0.000712125847622297 | 0.000556433951737452 | 0.000495340760859413 | 0.000479691981415104 | 0.0010385862261949 | 0.000606869124663833 | 0.000393316053490399 |
| pos\_351 | 2-(Methylthio)propane | 0.000347078297794174 | 0.000443860055559999 | 0.000358386002709592 | 0.00036206192827444 | 0.000312449502502598 | 0.000349516298544794 | 0.000319439398367201 | 0.000337913891728457 | 0.000365294341137333 | 0.000337944924960571 | 0.000344467956402594 | 0.000324871572871567 |
| pos\_352 | Ser Lys Met Trp | 0.00770903016752279 | 0.00601591709426555 | 0.00717118689559943 | 0.00849648734948203 | 0.00864748285337493 | 0.00952639632642864 | 0.0102246710891441 | 0.00758183582167656 | 0.0071800824232425 | 0.0105476519949014 | 0.00901424385395034 | 0.00741217693955996 |
| pos\_353 | Cinnamyl isobutyrate | 3.11755064910338e-05 | 3.65699713376489e-05 | 4.88121286526992e-05 | 2.15434970956378e-05 | 3.20989653456358e-05 | 3.32206399370549e-05 | 1.66186660597083e-05 | 2.87523203247549e-05 | 1.97773523512267e-05 | 5.39875518671913e-05 | 3.48529751267187e-05 | 4.19287574906922e-05 |
| pos\_354 | 4-Gingerol | 2.04439198166941e-05 | 9.94850297600681e-05 | 9.98965433203418e-06 | 1.2954639557619e-05 | 3.1288059037103e-05 | 3.19568989227523e-05 | 7.35152347841739e-05 | 4.24300123384494e-05 | 2.39020869654253e-05 | 1.53819953202115e-05 | 3.18807747725431e-05 | 9.33608800943136e-06 |
| pos\_355 | (E)-4,8-Dimethyl-1,3,7-nonatriene | 5.01966682476533e-06 | 6.01830556155366e-06 | 5.08755099389006e-06 | 6.98137159648489e-06 | 1.18748767743499e-05 | 5.52437629243271e-06 | 3.4725884977851e-05 | 1.92314855732834e-05 | 5.21255603879189e-06 | 1.57781317469681e-05 | 1.03835436074451e-05 | 1.27153231260111e-05 |
| pos\_356 | Dehydroanonaine | 0.000126090490540871 | 0.000319248393678642 | 0.00100175203958519 | 0.000325448108057457 | 0.00027578965089065 | 0.000406974119023448 | 0.000618398933988961 | 0.000496447126816094 | 0.000606449820279933 | 0.000941848377338815 | 0.000668689973251271 | 0.000323939492293493 |
| pos\_357 | Pro Gly Ser Asn | 0.000276612137936427 | 0.000279330488389335 | 0.000110140596194435 | 0.000204720506865015 | 0.000446975636232738 | 0.000243116561809951 | 0.000104798420797274 | 0.000121706602166102 | 0.000119501677757697 | 0.000301371699176403 | 0.000161394771616668 | 0.000161407245232325 |
| pos\_359 | beta-Sitostenone | 0.00105217471739811 | 0.001815686909577 | 0.00155849149964898 | 0.00110241936276682 | 0.000993835592857582 | 0.00154165127522092 | 0.00150596573089052 | 0.00158996704053635 | 0.00108860246286355 | 0.00136597356698271 | 0.00141359728228875 | 0.00124192943164303 |
| pos\_36 | Phenol | 2.63144237733091e-06 | 2.96997335153369e-06 | 2.15353368628034e-06 | 2.48591850455253e-06 | 3.52759700184399e-06 | 2.00596637485885e-06 | 2.98730094610678e-06 | 2.67172093232122e-06 | 2.51397957867515e-06 | 2.94961352492252e-06 | 2.35294060295347e-06 | 3.26533264401712e-06 |
| pos\_360 | Gitogenin | 0.000422657109573755 | 0.00043437115681212 | 0.000200587521415228 | 0.00101798675842231 | 0.000335538637541869 | 0.000493035650484907 | 0.000398742057755038 | 0.000331850961735969 | 0.000243749060515126 | 0.000712533519535583 | 0.000381907096918642 | 0.000270309217709879 |
| pos\_361 | 4-(Methylthio)-2-pentanone | 5.84870000024551e-06 | 3.92137865295844e-06 | 1.79814633244854e-06 | 4.04828858199333e-07 | 2.26232518457385e-06 | 3.01274323122303e-06 | 2.55542860392774e-07 | 3.47849836977254e-07 | 2.52374190654552e-07 | 6.11891006451638e-06 | 1.04009587275734e-06 | 3.14666396177676e-07 |
| pos\_362 | CAPSAICIN | 0.00321429148199038 | 0.00119320952915625 | 0.000352940832110498 | 0.00241914205211154 | 4.2433455951506e-05 | 0.00138898930387232 | 0.000192036515705298 | 0.000343995330278537 | 0.000547875987824616 | 0.00573706046458473 | 0.00132005516501419 | 0.000572736996619415 |
| pos\_363 | o-Cymene | 2.87109639720932e-05 | 9.05357939836172e-05 | 4.36453481049037e-05 | 1.99651914514203e-05 | 5.20683808442526e-05 | 4.36222252526175e-05 | 3.74451554338602e-05 | 3.55774938841194e-05 | 2.31496665606333e-05 | 7.69173902692253e-05 | 4.18640449755913e-05 | 3.37963386707577e-05 |
| pos\_364 | Ile His Val His | 7.32010209053675e-05 | 0.000125215385142173 | 7.71511779603665e-05 | 6.96839955791112e-05 | 8.45559954196934e-05 | 7.98914964708867e-05 | 0.000132989164345813 | 0.000114813483969075 | 7.43006067934245e-05 | 7.73561343873344e-05 | 0.000107780745674639 | 9.24345673782215e-05 |
| pos\_365 | Dehydromyodesmone | 1.46477922842559e-05 | 1.79372670251272e-05 | 4.07173615354036e-05 | 7.7785986502674e-05 | 2.0596944699155e-05 | 3.27677669293971e-05 | 2.48451124209214e-05 | 3.59216973412194e-05 | 1.34961321510251e-05 | 2.08694828744894e-05 | 2.71383950766889e-05 | 5.90104008383319e-05 |
| pos\_366 | 12-amino-octadecanoic acid | 7.13707474293769e-05 | 8.43704696617057e-05 | 7.81552965825958e-05 | 8.70053695009088e-05 | 5.60442041496291e-05 | 7.3305132874084e-05 | 7.4559508365506e-05 | 7.77005708706332e-05 | 6.66762246987091e-05 | 6.92247866266137e-05 | 7.17574968692968e-05 | 6.90764638969861e-05 |
| pos\_367 | Thr Asn Thr Asn | 0.000216097617504226 | 0.000255449165180804 | 0.000184437181435312 | 0.00020961996475486 | 0.000260805404975882 | 0.000233774257675022 | 0.000271818036148036 | 0.000260038800660341 | 0.000265373558087726 | 0.000229051504061019 | 0.000281453948663418 | 0.000307218494405299 |
| pos\_368 | Phthalic acid Mono-2-ethylhexyl Ester | 0.000637793324800147 | 0.000815047057242882 | 0.0009667751645386 | 0.000567290442327044 | 0.000553772504109584 | 0.000640277872868276 | 0.000502963204211478 | 0.000642569331757944 | 0.000628217765923623 | 0.000859767285689322 | 0.000649861723269564 | 0.000701935447058828 |
| pos\_369 | (+/-)-3-(Methylthio)heptanal | 1.30373070211055e-05 | 1.540129293015e-05 | 1.17806153979189e-05 | 1.58728311946136e-05 | 1.03874980485021e-05 | 1.32556268360622e-05 | 2.0767584139404e-05 | 1.7149072423779e-05 | 1.6406870431263e-05 | 1.52654721293832e-05 | 1.70427887716273e-05 | 1.22217649451441e-05 |
| pos\_37 | 1,2-Didecanoyl-sn-glycerol | 0.000278336563984244 | 0.000138350046963712 | 0.00019453140965213 | 0.000264232368527589 | 0.000170602639573322 | 0.000218015085399805 | 0.000174207106265528 | 0.000193354596322249 | 0.000163046613290385 | 0.000165375978829117 | 0.000192282108393824 | 0.000238498274239633 |
| pos\_370 | 3-Mercaptohexyl hexanoate | 2.51572021210585e-05 | 1.79723160201922e-05 | 1.45326849390666e-05 | 1.41652360926901e-05 | 2.72275318566365e-05 | 2.13959403262681e-05 | 1.78587347491686e-05 | 0.000145392586413647 | 1.54223689232773e-05 | 1.7533917936296e-05 | 0.000114363651679685 | 0.000386819981864059 |
| pos\_371 | Yucalexin B5 | 6.4295141882521e-05 | 5.24214958728056e-05 | 0.000135088668987694 | 2.156959022398e-05 | 0.000129118348798017 | 7.71677654027036e-05 | 1.47179729685711e-05 | 4.28690009434657e-05 | 2.93967940141922e-05 | 0.000316578245827684 | 8.39501447574635e-05 | 7.57951537277177e-05 |
| pos\_372 | Verruculotoxin | 2.08130906329061e-05 | 1.69536781912437e-05 | 2.49767232122401e-05 | 1.85448706953731e-05 | 2.5595462220891e-05 | 2.01760359118202e-05 | 1.31336322601156e-05 | 1.83507975389231e-05 | 9.36076993521142e-06 | 3.00001391696643e-05 | 1.92696705889044e-05 | 2.10486022083342e-05 |
| pos\_373 | Palmitoyl Serotonin | 0.00068201964550218 | 0.000694472335110317 | 0.000265155349416237 | 0.000942760545272871 | 0.000347037474226162 | 0.000609055602324551 | 0.000258290998134366 | 0.000219250495527349 | 0.000357474974326236 | 0.000954528768768032 | 0.000402660662184452 | 0.00017760102097413 |
| pos\_374 | Butanoyl PAF | 0.00101148642753778 | 0.000952435159597152 | 0.00236294020092609 | 0.000960510173249723 | 0.00150424990348782 | 0.00132952357132809 | 0.00166638190482699 | 0.00146480077628252 | 0.000823763618892104 | 0.00125851544156223 | 0.00124051989343119 | 0.00110378339105691 |
| pos\_375 | Isobutyrylmallotochromene | 0.000195677340198524 | 0.000125720434885379 | 9.40998399727389e-05 | 0.000136197273282615 | 8.6900281123637e-05 | 0.000136383312035498 | 5.47712094406931e-05 | 8.30628216582262e-05 | 4.88696830581599e-05 | 8.59308028173874e-05 | 8.04176241587173e-05 | 0.000128650303897588 |
| pos\_376 | Okanin 4'-O-(4'',6''-di-O-acetylglucoside) | 2.00460296381039e-06 | 1.0185081168135e-05 | 1.63165709685793e-06 | 5.2728809772144e-06 | 2.08449298714682e-06 | 2.83852159669793e-06 | 3.27147186002774e-06 | 2.33512257843949e-06 | 1.20166827903524e-06 | 9.68185544759852e-06 | 2.66959418507513e-06 | 0.00107122856224672 |
| pos\_377 | Bluensidine 6-phosphate | 5.56163214384277e-05 | 9.1066050797455e-05 | 3.06718372629266e-05 | 4.59394915763122e-05 | 9.50981657102877e-05 | 6.0342936138963e-05 | 5.70468279193933e-05 | 4.66098818955705e-05 | 0.000116167261054085 | 5.6166344667264e-05 | 6.01962409707394e-05 | 3.06958631523466e-05 |
| pos\_378 | (6alpha,7alpha,10alpha)-1(5),3-Aromadendradiene | 0.000454378292763916 | 0.000572452584415887 | 0.000419011325531331 | 0.000489279188833747 | 0.000482751298853779 | 0.000485576223673235 | 0.000609970599103845 | 0.000549248087305358 | 0.000554800645216923 | 0.000548069500856292 | 0.000545129524627621 | 0.000446583536903603 |
| pos\_379 | Bryononic acid | 0.00279829863371838 | 0.00266094181330622 | 0.00268875108046697 | 0.00241705748184377 | 0.00175894135132069 | 0.00239563433894509 | 0.00231893360600444 | 0.00256098197723019 | 0.00204397119796101 | 0.00262279207551854 | 0.00259113090231394 | 0.00278843118424591 |
| pos\_38 | 1α-hydroxy-22-(3-methylphenyl)-23,24,25,26,27-pentanorvitamin D3 / 1α-hydroxy-22-(3-methylphenyl)-23,24,25,26,27-pentanorcholecalciferol | 0.00299085974483149 | 0.00331211716869942 | 0.00261205710860345 | 0.00365225807591462 | 0.00241983905277594 | 0.00302510488226318 | 0.00570671047199091 | 0.00386329923290998 | 0.00412698402218457 | 0.00352561023125064 | 0.00401722663655608 | 0.0016030004551979 |
| pos\_380 | Pro His Arg Gly | 8.19008362084318e-05 | 0.000143965699437197 | 0.000211146223564659 | 9.45020943523976e-05 | 0.00016956984319763 | 0.000129584332658028 | 4.58173198426486e-05 | 5.53893626401211e-05 | 0.000260611842338239 | 4.84807326747526e-05 | 9.49018461266509e-05 | 7.0718896901312e-05 |
| pos\_381 | Methyl-liquiritigenin | 1.02980626001664e-05 | 1.62320696314108e-05 | 1.15607508069727e-05 | 1.34048096463067e-05 | 1.65915022204371e-05 | 1.28263400281745e-05 | 1.53915860075655e-05 | 1.12864893105821e-05 | 1.4066242196961e-05 | 1.41658473785501e-05 | 1.2364319625784e-05 | 9.39895457194839e-06 |
| pos\_382 | Cycloartomunoxanthone | 4.04118419755249e-05 | 3.79272871486798e-05 | 2.6155830852214e-05 | 2.52745273684022e-05 | 1.97494311755154e-05 | 3.23583123862912e-05 | 3.39905900249892e-05 | 3.75686793269993e-05 | 2.4853475868536e-05 | 2.58281230167722e-05 | 2.38941656500039e-05 | 2.9539388565994e-05 |
| pos\_383 | 3-Methoxyestra-1,3,5(10),16-tetraene | 6.50713347134789e-05 | 5.59899186219032e-05 | 0.000134197248455698 | 3.70254681797984e-05 | 0.000146759291954196 | 8.52138208534187e-05 | 7.82064243481126e-05 | 7.04071412876651e-05 | 3.53221191939252e-05 | 0.000249500090178977 | 8.96320722226019e-05 | 8.79829125195698e-05 |
| pos\_384 | Glu Trp Phe Trp | 2.1184797660288e-06 | 1.59191473107096e-05 | 1.72094156163786e-06 | 2.69599133746957e-06 | 2.20634019153538e-05 | 5.47200798450547e-06 | 8.58379868149854e-06 | 1.11242705483722e-05 | 6.11189038747841e-06 | 6.3253551624485e-06 | 9.22463014247926e-06 | 1.24631819238526e-06 |
| pos\_385 | Lobelanine | 5.04514565733945e-05 | 9.54289458222396e-05 | 9.95490209652035e-06 | 1.69723665358974e-05 | 2.01872849133441e-05 | 1.69029034525646e-05 | 2.05730074834937e-05 | 1.46346850969865e-05 | 1.76923434845699e-05 | 1.84698033242706e-05 | 1.69270599661439e-05 | 1.45061367201933e-05 |
| pos\_386 | Thr Arg Met Trp | 0.00760847986873856 | 0.00527513963024299 | 0.00530424515497035 | 0.00456563085153474 | 0.00930455523107257 | 0.00690968795861468 | 0.00638411374913282 | 0.00610840792975687 | 0.00566342860406698 | 0.00546229584621451 | 0.00607107620294686 | 0.00789163930427972 |
| pos\_387 | 7-Ethoxy-4-methyl-2H-1-benzopyran-2-one | 1.5498942497392e-06 | 2.71215778027187e-06 | 6.82143032243661e-06 | 1.36408358641221e-05 | 4.29140475946554e-06 | 6.57618721074927e-06 | 4.36105705601177e-06 | 9.31092013256655e-06 | 1.57804099699514e-06 | 2.52181955620545e-06 | 7.58022135286725e-06 | 2.29647511523821e-05 |
| pos\_388 | Demethylphylloquinone | 0.000449402744038741 | 0.00106995438779577 | 0.00119710105841041 | 0.000809289620122085 | 0.000777654809013148 | 0.000838418947059923 | 0.000855229740331671 | 0.00116781501765228 | 0.000924101053757498 | 0.00115921418136173 | 0.0010889977074054 | 0.00112060458773437 |
| pos\_389 | 2',5'-Dihydroxyflavone | 2.76968256415078e-05 | 8.60128878260902e-05 | 9.65529586061646e-05 | 3.22681264943859e-05 | 6.81680528845699e-05 | 6.88756224498202e-05 | 6.16325626920578e-05 | 5.86047389166625e-05 | 4.66933138092056e-05 | 6.12511762979694e-05 | 5.46488889777415e-05 | 8.51946380253864e-05 |
| pos\_39 | Choline | 6.6613822984998e-05 | 6.82317648693005e-05 | 4.68878999231789e-05 | 5.32014466010217e-05 | 0.000138778474589391 | 5.45711169400529e-05 | 0.000142133806240331 | 0.000228914691663387 | 7.3165985784274e-05 | 0.000219023871879198 | 0.000102767951404759 | 0.000305516516127399 |
| pos\_390 | Bis(2-ethylhexyl) phthalate | 0.000465477234250507 | 0.000656090586109973 | 0.000866519560155979 | 0.000544071619128942 | 0.000351649860094421 | 0.000513921655224452 | 0.000501470797621015 | 0.000603679800352167 | 0.000592412330820539 | 0.000487766909669712 | 0.000603436790969024 | 0.000436830785930701 |
| pos\_391 | Rubrofusarin | 4.15262988047571e-06 | 0 | 0 | 0 | 0 | 1.62731314395573e-05 | 0 | 0 | 0 | 1.05104444820377e-05 | 0 | 0 |
| pos\_392 | Ethyl 10-undecenoate | 0.00798175534592316 | 0.0120702659852653 | 0.0109656458607829 | 0.0130777971912258 | 0.0114299699411069 | 0.0112379213717448 | 0.0111598797173231 | 0.010370395663137 | 0.0126496071907665 | 0.0104256835812672 | 0.0109486780526892 | 0.00901329887250157 |
| pos\_393 | Furanodiene | 1.00331225405065e-05 | 1.83552580533947e-05 | 1.70271415056468e-05 | 2.00719759088507e-05 | 1.31422127090737e-05 | 1.43881570561717e-05 | 1.38328393432007e-05 | 1.6122936156702e-05 | 1.37214192509594e-05 | 1.38351740777893e-05 | 1.26142145121742e-05 | 1.62802040169553e-05 |
| pos\_394 | alpha-Amylcinnamyl isovalerate | 2.53773987254545e-05 | 3.19611949582398e-05 | 2.21838243791592e-05 | 2.97286438647586e-05 | 3.0990437901268e-05 | 2.76274577292335e-05 | 2.63448077787767e-05 | 2.48815483213009e-05 | 2.35549738070376e-05 | 3.23612150274253e-05 | 2.82091890250172e-05 | 2.85734310452568e-05 |
| pos\_395 | S-[2-(N7-Guanyl)ethyl]-N-acetyl-L-cysteine | 7.26444928249644e-06 | 6.2635079833115e-06 | 5.62665737142095e-06 | 4.31232255750456e-06 | 6.61534388317932e-06 | 6.14905131547532e-06 | 5.9995621440872e-06 | 6.41382218655107e-06 | 4.31273130063771e-06 | 5.94956504872307e-06 | 4.58759614326209e-06 | 6.18131648134413e-06 |
| pos\_396 | 5-Cholestene-3beta,7alpha,12alpha,24,25-pentol | 0.000459914655540221 | 0.000216265677312805 | 0.000412342673393558 | 0.000445172532850542 | 0.000185691128088792 | 0.000340021482792786 | 0.00035739300445741 | 0.000360295352933905 | 0.000227258543905218 | 0.000369931580188339 | 0.000398043787825998 | 0.000370340149530639 |
| pos\_397 | 4,4'-Methylenebis(2,6-di-tert-butylphenol) | 0.00548364610891642 | 0.0102643225778177 | 0.00743345887303006 | 0.0101472213982848 | 0.00885510562814571 | 0.00846297019797387 | 0.00838153076105747 | 0.00719111773076182 | 0.0108333782966999 | 0.00772121100872575 | 0.00819103804336123 | 0.00487497469850253 |
| pos\_398 | Purothionin AII | 0.0304157310226918 | 0.0370582867396509 | 0.0344285653711987 | 0.0293115130805751 | 0.0269503756019216 | 0.0284843108026613 | 0.037381981593272 | 0.0325051654125888 | 0.0283534543327731 | 0.0391470117250641 | 0.0249068899382498 | 0.0274718974734259 |
| pos\_4 | L-Proline | 9.15738053998581e-05 | 0.000129439438364555 | 8.7050951573961e-05 | 0.000118757544492256 | 0.000125054196657801 | 7.63506916537527e-05 | 0.00013746018411638 | 0.000146424513581234 | 0.000104463117622897 | 0.000175281085928984 | 0.000100086875284302 | 0.000151745157164841 |
| pos\_40 | cis-8, trans-10, cis-12-octadecatrienoic acid; C18:3n-6,8,10 | 2.67866402056648e-05 | 0.00010421468919077 | 0.000204621455790207 | 0.000198583215541061 | 0.000111888980466723 | 0.000100477603479025 | 5.54530434244305e-05 | 0.000125419093407734 | 0.000121206067474334 | 2.62879701730449e-05 | 3.93099215362849e-05 | 7.54480457265736e-05 |
| pos\_401 | Flazine methyl ether | 1.0971590719592e-05 | 1.25215958850994e-05 | 1.31309509417191e-05 | 1.60942855317296e-05 | 2.51957643209172e-05 | 1.9245672258379e-05 | 1.6227850628574e-05 | 1.57832307387831e-05 | 1.96891545939825e-05 | 2.60825910524523e-05 | 1.77512218912371e-05 | 1.626428925208e-05 |
| pos\_402 | Procerin | 2.44500542598874e-05 | 2.1089353534723e-05 | 5.90613478199414e-05 | 0.000176292618621516 | 2.91794803952485e-05 | 6.16563491083352e-05 | 2.41440232175881e-05 | 0.000117305165848435 | 1.6006133427076e-05 | 1.86426185219249e-05 | 7.89543400703301e-05 | 0.000265283421653424 |
| pos\_403 | CAY10408 | 1.76512654008535e-05 | 1.57279420943696e-05 | 1.65112128909468e-05 | 0.000137646897457147 | 0.00269940451711878 | 4.07496219913079e-05 | 3.98991660478139e-05 | 2.90392041154443e-05 | 2.27381157017304e-05 | 2.13261959659573e-05 | 2.16814388347779e-05 | 2.44756885954004e-05 |
| pos\_404 | 9(S)-HETE | 0.000107866679959699 | 0.000117557084531025 | 7.74188753953171e-05 | 8.01969514534529e-05 | 7.42550690847155e-05 | 9.00573402805044e-05 | 7.01005106982675e-05 | 7.80133321028455e-05 | 6.26807753675769e-05 | 9.25263769741048e-05 | 8.72855408671192e-05 | 9.52401084197748e-05 |
| pos\_405 | 3-Oxo-delta1-steroid | 4.36367261994956e-05 | 4.17656988202881e-05 | 3.96797693781415e-05 | 3.99286927632274e-05 | 3.55275573329625e-05 | 4.04648385413501e-05 | 3.47997135597514e-05 | 3.98940738458123e-05 | 3.50258396767062e-05 | 4.50433655415199e-05 | 3.99991833890013e-05 | 4.20968314871363e-05 |
| pos\_406 | Meclizine | 0.000301064239315955 | 0.000300980275546456 | 0.000305200046777133 | 0.000292506554876843 | 0.000283749004220903 | 0.000306463499954706 | 0.000223244602088314 | 0.000284770171337879 | 0.000331009789430326 | 0.00025342467775976 | 0.000281807142998853 | 0.000331597962550345 |
| pos\_407 | 2,3-Dimethyl-2-cyclohexen-1-one | 1.26213391106643e-05 | 1.68182844056758e-05 | 1.69739627582497e-05 | 1.79165923436912e-05 | 1.34441272796568e-05 | 1.39636069467514e-05 | 1.2067893983028e-05 | 1.54066966384165e-05 | 1.67012696652627e-05 | 1.47572401251692e-05 | 1.62718049862437e-05 | 1.46248075805528e-05 |
| pos\_408 | His Pro Asn Arg | 0.000169532744697581 | 0.000131089488744809 | 0.00014342806213103 | 0.000176673302683817 | 0.000118391784062387 | 0.000146041836532296 | 0.000138347438308202 | 0.000156203893833526 | 0.000133845530744554 | 0.000158474271322293 | 0.000155337781090605 | 0.000179861074621504 |
| pos\_41 | Mesitylene | 0.000112494772211575 | 0.000153453425111674 | 0.000159889250167952 | 0.000161063888435716 | 0.000137174767422085 | 0.000141794370257374 | 0.000170744924090231 | 0.000164346160346335 | 0.00013827903225168 | 0.000145655530981278 | 0.000147676825406493 | 0.000164383120665388 |
| pos\_410 | Stigmastentriol | 0.00437525144061444 | 0.00532066518794296 | 0.00486437748390228 | 0.00484770506267225 | 0.00447111821913969 | 0.00472920061298679 | 0.00441393907519631 | 0.00485910303629336 | 0.00605521495821001 | 0.00422226496311316 | 0.00504352056391372 | 0.00476249503242346 |
| pos\_412 | 1,9Z,16-heptadecatrien-4,6-diyn-3,8-diol | 7.63744780597284e-06 | 9.2501988412404e-06 | 8.3732207132336e-06 | 8.41658492250212e-06 | 7.61142870147749e-06 | 8.45420047257598e-06 | 7.10959120555605e-06 | 9.81479896451643e-06 | 8.3899563320158e-06 | 8.88388971795461e-06 | 9.40548904242588e-06 | 1.43930226887623e-05 |
| pos\_413 | Lys-Val-OH | 0.000106452085574319 | 0.000323617141835564 | 0.000172161955428825 | 0.000149945453324301 | 0.000353107644068208 | 0.000216249383676931 | 0.000312746580190652 | 0.000272060648238689 | 0.000815250538381301 | 0.000487965632063558 | 0.00046765506936402 | 0.000255403027919397 |
| pos\_414 | Thr Asp Met Glu | 1.03821754772241e-06 | 7.57742473656497e-06 | 1.17992884340202e-06 | 3.85845417568109e-06 | 2.88577078603362e-06 | 2.28413591361623e-06 | 7.57721654005312e-06 | 3.32158670769465e-06 | 7.75910334050848e-07 | 5.11047080294169e-06 | 3.8414755491e-06 | 2.61627694610074e-07 |
| pos\_415 | 1,4-Dideoxy-1,4-Imino-D-Arabinitol | 4.7832349764368e-05 | 5.18041703405813e-05 | 3.29011659762856e-05 | 4.60334192719133e-05 | 5.81106024631144e-05 | 3.79885635930868e-05 | 4.23762733831356e-05 | 4.3387655227254e-05 | 6.24287619868362e-05 | 3.51678581575929e-05 | 4.50540313760606e-05 | 4.89125374941396e-05 |
| pos\_416 | Bufalin | 0.000109498747680866 | 0.000227896529539198 | 0.000163271918566575 | 0.000144491880826264 | 0.000143586623873032 | 0.000113452058137876 | 0.000485628581848269 | 0.000488720315045481 | 0.000142642816224674 | 0.0001911694148929 | 0.000134357684793777 | 0.000148709158372027 |
| pos\_417 | Onchidal | 0.000143847233229564 | 0.000141044145446847 | 0.000121491823679 | 0.000116840593985921 | 0.000133618281270975 | 0.000131065561275121 | 0.000129304503134989 | 0.000122792265624447 | 0.000130301065203974 | 0.00020454682601545 | 0.000133281132791888 | 0.000136228458566575 |
| pos\_418 | 3,3',4,4'-Tetrahydroxy-5,5'-diisopropyl-2,2'-dimethylbiphenyl | 1.09463753285684e-05 | 6.82200491224141e-06 | 0.000146782078187734 | 0.00080980196166501 | 1.5786381389653e-05 | 8.12872943854177e-05 | 8.5228141570933e-06 | 0.00014164869057028 | 4.82266707258537e-06 | 9.6851327734343e-06 | 8.90677640781537e-05 | 0.000413123723768279 |
| pos\_419 | Neoporrigenin B | 0.000182964573036966 | 0.000212639216308164 | 0.000133121238135541 | 0.000184400964872297 | 0.000155771432101585 | 0.000170691773405037 | 0.000152504838807068 | 0.000151312022758197 | 0.000145197899128016 | 0.000223536381370891 | 0.000159397445660442 | 0.000144670771135831 |
| pos\_42 | (R)-Pterosin B | 2.88502344489009e-05 | 2.89067207795792e-05 | 0.000124754746278667 | 0.000223168882982389 | 3.90058701033279e-05 | 8.64589489368579e-05 | 2.5272487342216e-05 | 8.57525285482419e-05 | 2.12579997096021e-05 | 2.67230499051283e-05 | 6.46717742789077e-05 | 0.000183152410679882 |
| pos\_420 | Rosifoliol | 1.36000649458418e-05 | 8.10180670383839e-06 | 1.70269671677495e-05 | 4.80840419259971e-05 | 8.05837313917604e-06 | 1.98621677980212e-05 | 7.287056776959e-07 | 9.24624793502544e-06 | 9.9610734091816e-06 | 1.93190407548679e-05 | 1.0171889195321e-05 | 1.79038294434396e-05 |
| pos\_421 | KHAYASIN | 2.57886757897702e-05 | 2.80230509282061e-05 | 2.47743552201828e-05 | 2.31724902140043e-05 | 2.92241192389448e-05 | 2.62980736164137e-05 | 2.47356245527503e-05 | 2.70411163939905e-05 | 2.62204948186019e-05 | 2.835388881058e-05 | 2.61737453829544e-05 | 2.4891341095674e-05 |
| pos\_422 | Retinol / Retinol skeleton | 2.58521127107266e-05 | 2.78299148828915e-05 | 2.78669970878298e-05 | 2.5012240822681e-05 | 2.17419909295774e-05 | 2.45933201597222e-05 | 1.94446332514303e-05 | 2.55010528007207e-05 | 2.01900395796003e-05 | 3.04311891117266e-05 | 2.19238303024574e-05 | 2.62953952830509e-05 |
| pos\_423 | PE(18:4(6Z,9Z,12Z,15Z)/22:6(4Z,7Z,10Z,13Z,16Z,19Z)) | 0.000170514210140214 | 0.000163375651155326 | 0.00013695874089453 | 0.000146793958016172 | 0.000125893739151298 | 0.00012998507205705 | 0.000152413866251079 | 0.000123834150896507 | 0.000148854681208197 | 0.00013829962124118 | 0.000127253549998859 | 0.000141987948956023 |
| pos\_424 | TBS-Corey Lactone Aldehyde | 3.16616084351354e-05 | 3.6797057898563e-05 | 2.29399719607092e-05 | 3.3776491683956e-05 | 3.0086860892638e-05 | 3.12960341567206e-05 | 3.10673799657073e-05 | 3.1794960724163e-05 | 2.04705896857305e-05 | 3.63773438815402e-05 | 3.28352507994198e-05 | 4.13048889857522e-05 |
| pos\_425 | 2,2,7,7-Tetramethyl-1,6-dioxaspiro[4.4]nona-3,8-diene | 0.00014334201542221 | 0.000537890524601042 | 0.000252205020187092 | 9.49869839338912e-05 | 0.000285901859263366 | 0.000246568094301389 | 0.000207455409066662 | 0.000195218546016992 | 0.00011787887797276 | 0.000462457749702585 | 0.000235634660306874 | 0.000194741422158225 |
| pos\_426 | 1,3,11(13)-Eudesmatrien-12-oic acid | 0.000115194958975412 | 0.000136594343270812 | 0.000143949511807172 | 0.000118466335652596 | 0.00010326282977024 | 0.000125979298514684 | 0.000120606073776661 | 0.000131916234755038 | 0.000119282149375515 | 0.000154716859930025 | 0.000131687463517926 | 0.00014425230351188 |
| pos\_427 | Infectocaryone | 3.20780289781952e-06 | 7.4929008214956e-06 | 1.23165008808286e-06 | 5.80238930342875e-06 | 2.94285681765558e-06 | 3.91628400619176e-06 | 5.88648550140456e-06 | 1.50340879133459e-06 | 2.81838889955485e-06 | 2.73674981929683e-06 | 3.46052795396468e-06 | 1.55597700002826e-06 |
| pos\_428 | Tyr Lys Gly | 1.78319611385133e-05 | 2.62544651742787e-05 | 2.21059015428429e-05 | 1.74483627503338e-05 | 1.36474077528434e-05 | 1.96393815442623e-05 | 1.42108975772953e-05 | 1.62132949050698e-05 | 1.89868884979672e-05 | 1.54748353033883e-05 | 1.71588800926742e-05 | 1.33411039032122e-05 |
| pos\_429 | Leukotriene A3 methyl ester | 4.46663741225565e-05 | 5.17894550150367e-05 | 4.46346614508765e-05 | 4.10482094271111e-05 | 4.2643838871994e-05 | 4.62365242698292e-05 | 4.47938091191772e-05 | 5.1037467641823e-05 | 4.62834740890693e-05 | 3.38962354422486e-05 | 3.84683422779975e-05 | 0.000107304992265537 |
| pos\_43 | 4-Vinylcyclohexene | 6.27136757279376e-05 | 5.7256313895899e-05 | 5.83958031380764e-05 | 7.14401666821205e-05 | 4.95468426246903e-05 | 6.11962649837102e-05 | 6.00465885813051e-05 | 6.87222726537903e-05 | 6.30891954282121e-05 | 6.46796618717365e-05 | 6.84947876736975e-05 | 7.14376392933127e-05 |
| pos\_431 | PPA | 5.53390972560189e-06 | 5.19982745786813e-06 | 8.60257572750816e-06 | 9.15324883718979e-06 | 1.12809849267776e-05 | 9.26987602738107e-06 | 7.63852329708562e-06 | 6.83452398324002e-06 | 1.02791859546504e-05 | 8.06257396893723e-06 | 8.34633249811065e-06 | 9.15268615367691e-06 |
| pos\_432 | (8S,Z)-6-((S)-3-hydroxy-2-methylpropylidene)-8-methyloctahydroindolizin-8-ol | 8.22876214681414e-05 | 0.000437908376940053 | 0.000166995656251941 | 0.000108992729490403 | 7.95734891723184e-05 | 0.000141918119951212 | 0.000145280622726047 | 0.000199756097567807 | 0.000150045165715359 | 9.5039770884335e-05 | 0.00014952279235853 | 0.000201607462801338 |
| pos\_433 | Thr Asp His Glu | 5.87639610839522e-05 | 7.45950876635666e-05 | 7.59476475333652e-05 | 6.59156404458601e-05 | 5.95360304516366e-05 | 6.30317647704191e-05 | 5.36898735919489e-05 | 6.4055821575752e-05 | 6.81837862986909e-05 | 6.23210511385767e-05 | 6.37772005611766e-05 | 6.11890630837545e-05 |
| pos\_434 | 2-(1-Pyrrolidinyl)-3-pentanone | 4.8063500541604e-05 | 1.9613243962012e-05 | 3.42895478025642e-06 | 1.2575306510089e-05 | 2.65186294982331e-05 | 1.86882878046255e-05 | 7.44002678574519e-06 | 4.36100390303156e-06 | 3.43256570769086e-06 | 1.38938174990085e-05 | 7.87563399544254e-06 | 1.5144881601952e-06 |
| pos\_436 | (9R,13R)-1a,1b-dinor-10,11-dihydro-12-oxo-15-phytoenoic acid | 1.44408509969608e-05 | 1.89313503151776e-05 | 1.61332693606851e-05 | 1.51318485687441e-05 | 1.49914208430132e-05 | 1.53202881398892e-05 | 1.44409041369233e-05 | 1.45234222622411e-05 | 1.33480967445818e-05 | 1.92443376489009e-05 | 1.68036107356852e-05 | 1.61355578538238e-05 |
| pos\_437 | 1,3-Diphenylpropane | 1.59603438026207e-05 | 2.12549144473673e-05 | 1.81441168274092e-05 | 2.30831697243284e-05 | 1.72092155606542e-05 | 1.8687003780707e-05 | 1.98260757141176e-05 | 2.06395837594609e-05 | 1.80040974144243e-05 | 2.02013519256367e-05 | 2.01662989461112e-05 | 2.29272356485373e-05 |
| pos\_438 | nandrolone | 4.25686197456046e-05 | 5.39213678667477e-05 | 3.1038532470264e-05 | 4.53079582165509e-05 | 3.76657713108916e-05 | 3.71864848709841e-05 | 4.96762628049544e-05 | 4.01841063705611e-05 | 4.25562860703892e-05 | 3.55385945743884e-05 | 3.99950903692562e-05 | 3.96918569559843e-05 |
| pos\_439 | 4-Isopropyl-3-methylphenol | 6.11843985261778e-05 | 0.000143794740784227 | 6.34109842715896e-05 | 6.22325668578188e-05 | 0.000111837658679231 | 8.42646016774003e-05 | 8.42449495376195e-05 | 7.6133589436557e-05 | 7.61576725810303e-05 | 9.98372541452447e-05 | 7.75937084714716e-05 | 6.62269601057845e-05 |
| pos\_44 | 3-oxoglycyrrhetinic acid | 0.00173695121507966 | 0.00238367765022431 | 0.00300606678489765 | 0.00301813617784007 | 0.00179119003577206 | 0.00223731829680848 | 0.00300511445248268 | 0.00308557909548833 | 0.002602382349115 | 0.00219052033506473 | 0.00283184877528092 | 0.00382017085847451 |
| pos\_441 | PA(20:1(11Z)/17:0) | 1.6951147773309e-05 | 5.74415636120688e-06 | 2.95390866141779e-06 | 1.74700903129284e-06 | 1.87365111498547e-06 | 2.67580809788964e-06 | 0 | 1.56722805750432e-06 | 5.74365690707333e-06 | 2.06979543553848e-06 | 1.71729736448524e-06 | 2.62125692793214e-06 |
| pos\_442 | Val Ala Leu Met | 0.000400409869188798 | 0.000483571243585896 | 0.000494370405504 | 0.000421150382632066 | 0.000349872511537846 | 0.000396748150176267 | 0.00041251613198391 | 0.000442173229253597 | 0.000467158551632116 | 0.000428908726277122 | 0.000391694182180531 | 0.000439717389023541 |
| pos\_443 | Magnosalicin | 0.000233691992839301 | 0.000245132219096109 | 0.000237498886582208 | 0.000198899611145562 | 0.000226381083056679 | 0.000211852226844759 | 0.000254993861798488 | 0.000232062688663756 | 0.000204166940714897 | 0.000275743603721786 | 0.000185053794462378 | 0.000215586013099237 |
| pos\_444 | Isomethiozin | 7.43881494527549e-05 | 9.83573563433694e-05 | 9.62695174677134e-05 | 8.33136629754188e-05 | 6.74495604290969e-05 | 8.52451454366294e-05 | 7.38755053320666e-05 | 8.16262981661961e-05 | 9.31147833296961e-05 | 8.55332200565853e-05 | 8.15653750425107e-05 | 7.76684701699371e-05 |
| pos\_445 | 17(R)-HETE | 0.000500507115779313 | 0.000389205266814471 | 0.000367597736812039 | 0.000332665022801012 | 0.000357585800548605 | 0.000382836986178755 | 0.000399372994338443 | 0.000385698045176769 | 0.000391904983089368 | 0.000361923562751139 | 0.000377035608114666 | 0.000379241531299277 |
| pos\_446 | Idoxanthin | 0.00115326614996404 | 0.00124195234669622 | 0.00102550512453796 | 0.00117427569005014 | 0.000639941527618212 | 0.00106864135687317 | 0.00068702005297335 | 0.00103348720824763 | 0.00112985349850605 | 0.000940756111222878 | 0.00108601135383376 | 0.00125385943726107 |
| pos\_447 | Peimine | 2.13242631700291e-05 | 1.56616352270017e-06 | 2.23530713535295e-08 | 2.14521873058057e-06 | 1.03204207344069e-07 | 5.42463995771331e-06 | 1.45057909569877e-06 | 2.29405334880421e-07 | 5.48191629445635e-06 | 3.03957998490785e-05 | 4.61971143588715e-06 | 1.57515648858303e-07 |
| pos\_448 | Met His Lys | 0.00384178521513875 | 0.00456953708683339 | 0.00470111182923556 | 0.00548242110227299 | 0.00351668795362955 | 0.00403186991085755 | 0.00410808820364637 | 0.00413953832849168 | 0.00433129738165433 | 0.00411609376358452 | 0.00455937563162381 | 0.00408923333057423 |
| pos\_449 | Thr Lys Arg | 0.000240571826514778 | 0.00206951661035143 | 0.00148099493311014 | 0.00162284494018566 | 0.00051154426315852 | 0.000710735470501764 | 0.000718368365965772 | 0.000756880261262242 | 0.000683426727301277 | 0.000159486498874544 | 0.000260195433505102 | 0.000487334080819942 |
| pos\_45 | (3beta,22R,23R,24S)-3,22,23-Trihydroxystigmastan-6-one | 0.00177635218439728 | 0.00231342980422474 | 0.00215932860159392 | 0.00228670680836158 | 0.00180852252867085 | 0.00258263446158069 | 0.0022973879528943 | 0.0028609218100813 | 0.00249522330614528 | 0.00178960885344882 | 0.00239876131005013 | 0.00197289877717803 |
| pos\_450 | 9-hydroxy-hexadecan-1,16-dioic acid | 0.000246984629493455 | 0.00027444757969913 | 0.000501029730442869 | 8.2408893266455e-05 | 0.000274104849248827 | 0.000274785177445687 | 4.41998402361603e-05 | 0.000194337169620501 | 7.52300529595229e-05 | 0.000599890492896098 | 0.000278015747254555 | 0.0004176572189607 |
| pos\_451 | Dicrocin | 0.000113392663591567 | 0.000126593872037827 | 0.000141559597749296 | 0.000154080161163572 | 8.49884385123665e-05 | 0.000141057588427873 | 9.21111966039418e-05 | 0.000148217910393749 | 0.000134155960205709 | 0.000122254815590237 | 0.000139871573392112 | 0.000120604653418341 |
| pos\_452 | Nitropolyzonamine | 1.59050202470765e-05 | 2.13292615368986e-05 | 2.15659073607217e-05 | 1.78368693607449e-05 | 1.55306281036762e-05 | 1.91183636001351e-05 | 1.56276356411011e-05 | 1.77587025936687e-05 | 1.77439223781695e-05 | 1.86635065178525e-05 | 1.82567695935148e-05 | 1.73578349864486e-05 |
| pos\_453 | Ricinoleic Acid methyl ester | 0.0012504137406963 | 0.00151198633809553 | 0.0013414405778016 | 0.00120488535241767 | 0.00107155654371662 | 0.00139895088972454 | 0.00138015676964758 | 0.00133527855635631 | 0.00136783555242157 | 0.00117093822639348 | 0.0012468417966044 | 0.00118628016441468 |
| pos\_454 | 5,8,11-Eicosatriynoic Acid | 0.000233691733241036 | 0.000184185974719178 | 0.000407218776445279 | 5.48581236413385e-05 | 0.000212840588888881 | 0.000166765232057221 | 3.33415083944579e-05 | 0.000144680563476355 | 7.92227490738707e-05 | 0.000525847530029166 | 0.000190144285639721 | 0.000318494653108618 |
| pos\_455 | 1α,25-dihydroxy-19-nor-22-oxavitamin D3 / 1α,25-dihydroxy-19-nor-22-oxacholecalciferol | 0.000414403054854663 | 0.000828835152658452 | 0.00104209058704752 | 0.000643778197596014 | 0.000617999802275897 | 0.000752518366950398 | 0.000782142835111184 | 0.00111694009796736 | 0.000720006594797748 | 0.000781326819447728 | 0.00089488408794331 | 0.00143511062550468 |
| pos\_456 | QH(2) | 5.06987261177069e-05 | 5.46268760985437e-05 | 7.65610899571986e-05 | 6.38038433048527e-05 | 7.45836376391216e-05 | 6.60558485511407e-05 | 4.49374276681915e-05 | 4.91063279776679e-05 | 7.5439519476182e-05 | 6.86567607710366e-05 | 5.55302229142894e-05 | 6.00468409250395e-05 |
| pos\_457 | 2-Oxosuberate | 8.05934350202912e-05 | 0.00021280686342484 | 6.34567398263294e-05 | 7.52801188107435e-05 | 0.000166141934593856 | 0.000112887111881145 | 7.64626177547543e-05 | 7.49579864196928e-05 | 9.8847470731117e-05 | 0.000110520183928167 | 8.76363673434802e-05 | 7.45199748762476e-05 |
| pos\_458 | Gly Cys Glu Cys | 3.74818959020059e-05 | 5.03150136946755e-05 | 2.51878452445356e-05 | 3.41730820970625e-05 | 4.39954233548444e-05 | 3.41500926340537e-05 | 3.27715252371309e-05 | 2.92968425615655e-05 | 3.94726282964539e-05 | 2.56497508122061e-05 | 2.9872100979128e-05 | 2.34260441063595e-05 |
| pos\_459 | Isopropyl tiglate | 3.73081401652002e-06 | 6.18911148848388e-06 | 2.53161502062225e-06 | 3.03450743354319e-06 | 4.94087956392963e-06 | 4.078556855588e-06 | 4.86505657903301e-06 | 2.99701282456161e-06 | 5.17501414639826e-06 | 5.11673578247552e-06 | 3.89825541542504e-06 | 2.38913247321171e-06 |
| pos\_46 | 6-Ethyl-4-methyl-3E,5E,7E-decatriene | 4.97066303692986e-06 | 7.04776367707154e-06 | 6.78528436462126e-06 | 6.82881593618377e-06 | 5.19454133630534e-06 | 4.55763023479665e-06 | 5.18160965144622e-06 | 6.36044702560422e-06 | 6.84737872163707e-06 | 7.2218419465774e-06 | 6.9846194578425e-06 | 6.64724175819094e-06 |
| pos\_460 | Norcapsaicin | 3.05131797153482e-05 | 3.4712690051585e-05 | 3.37806450520341e-05 | 4.64811894970706e-05 | 3.74378627918438e-05 | 3.66107559885209e-05 | 4.20889437070227e-05 | 4.23524187218399e-05 | 3.14637942743458e-05 | 3.05182540467133e-05 | 3.68320183780097e-05 | 3.48740554303748e-05 |
| pos\_461 | Palmitic amide | 0.000328276060687588 | 0.000409741390369279 | 0.000379287756679411 | 0.00039479199285123 | 0.000286783872001675 | 0.000355072952528103 | 0.00035581756539463 | 0.00036963395029661 | 0.000334228553819982 | 0.000342197437491278 | 0.000351344676640583 | 0.000336420185146016 |
| pos\_462 | Stearoylglycine | 3.3000788718373e-05 | 3.27675505340235e-05 | 3.80499224696696e-05 | 3.52151340515532e-05 | 3.72585956784219e-05 | 3.22926258619516e-05 | 5.71771923147123e-05 | 5.87193588189898e-05 | 3.65917172468918e-05 | 3.25413593381306e-05 | 4.88183732679336e-05 | 0.000123655823856737 |
| pos\_463 | Jasmonic acid | 9.12181578341184e-05 | 0.000342069105802936 | 8.84240531883025e-05 | 6.15827210818722e-05 | 2.47398060691083e-05 | 9.57205996224439e-05 | 5.81079197198833e-05 | 7.97476294952066e-05 | 5.69720032450291e-05 | 4.52474384352448e-05 | 6.82775069298899e-05 | 0.000124107130082494 |
| pos\_464 | Tacrine | 5.55309957507437e-06 | 5.19172781956586e-06 | 3.65611830058492e-06 | 9.00888872056823e-06 | 8.21449949899373e-06 | 1.13735845261561e-05 | 4.03430734114031e-06 | 8.84538880696593e-06 | 1.077741226706e-05 | 5.7121830832156e-06 | 7.3537395103629e-06 | 1.28877258046943e-05 |
| pos\_465 | 3-Methyl-alpha-ionyl acetate | 7.65943916185264e-05 | 8.9951508972092e-05 | 8.53893026914888e-05 | 9.5660631658088e-05 | 7.26191928980829e-05 | 8.52475894298813e-05 | 8.77724645495951e-05 | 8.8343092451186e-05 | 8.77101607842317e-05 | 8.82148526479326e-05 | 8.91124599652608e-05 | 7.74012946717905e-05 |
| pos\_466 | (S,Z)-Lyratol acetate | 5.87562593623579e-05 | 9.31302089581699e-05 | 8.06587760214579e-05 | 7.64071747776017e-05 | 5.73197498565965e-05 | 7.2347538622297e-05 | 7.84952738426552e-05 | 8.28918957137695e-05 | 7.5562106673768e-05 | 7.19687314811584e-05 | 8.05464128130077e-05 | 9.05154957556293e-05 |
| pos\_467 | Capsanthin 3,6-epoxide | 0.00721251736683687 | 0.0086396849526427 | 0.00684415409260309 | 0.00824707366195016 | 0.00419288420764488 | 0.0080749008758764 | 0.00568998941312847 | 0.00788201755875259 | 0.00826876756559777 | 0.00537476309077882 | 0.00795273145722749 | 0.00728377353285828 |
| pos\_468 | Tricycloekasantalol | 1.9456714742994e-05 | 3.2752746862174e-05 | 1.77745910019743e-05 | 1.32126593738261e-05 | 2.20169177013356e-05 | 1.94815478973485e-05 | 2.27315046001801e-05 | 1.63922564883749e-05 | 2.40040406416636e-05 | 2.47609402417251e-05 | 1.96607583432884e-05 | 1.00676750343543e-05 |
| pos\_469 | Moracin I | 0.000138990802009447 | 0.000215231645954302 | 8.72313844940937e-05 | 0.000128314141820291 | 0.000120030321580308 | 0.000142783664585479 | 0.000137351539300693 | 0.000115109383472991 | 0.000143641357480707 | 0.00015268131660228 | 0.000134463155244198 | 8.00661142648312e-05 |
| pos\_47 | Carnosol | 0.000281492842175376 | 0.000294866458579958 | 0.000788621258119985 | 9.08915583903242e-05 | 0.000434267283705221 | 0.000364864425490376 | 4.42386061179222e-05 | 0.00026179680398123 | 8.64223692664398e-05 | 0.00129570960834289 | 0.000343344109525845 | 0.00059806613420896 |
| pos\_470 | Methyl (2E,4Z)-decadienoate | 5.20185143667817e-05 | 6.47152244100661e-05 | 6.22889130748565e-05 | 5.96780662126449e-05 | 4.92493786710409e-05 | 5.47507263378828e-05 | 5.42642697473633e-05 | 6.14170010093586e-05 | 5.66684780406346e-05 | 5.8434974215406e-05 | 5.93590889212236e-05 | 6.18554716159023e-05 |
| pos\_471 | Piperalol | 9.66965515349159e-05 | 9.79280127930531e-05 | 0.000107454041266904 | 0.000132921577857889 | 0.00018984676110044 | 0.000128973581968307 | 0.000115635236811174 | 0.000326855413224106 | 7.45758550255405e-05 | 0.000102454858459739 | 0.000249261207411269 | 0.000687511621676605 |
| pos\_472 | Heliespirone A | 8.50022643970497e-05 | 0.000131051972352227 | 6.54472047645062e-05 | 7.51784734625052e-05 | 9.05662467616028e-05 | 8.20020150812127e-05 | 7.95844410382783e-05 | 7.60198809341743e-05 | 9.03943243720819e-05 | 8.83046557329003e-05 | 7.48190362500105e-05 | 6.00303263895396e-05 |
| pos\_473 | 3'-Ketolactose | 0 | 6.18143512068655e-10 | 0 | 8.6439455871609e-06 | 7.9331476722926e-06 | 1.66725850231714e-06 | 8.92265499082164e-06 | 3.55715667755019e-06 | 3.49844116850397e-08 | 1.24760461746662e-08 | 1.92603243691255e-06 | 0 |
| pos\_474 | (3S,6E)-Nerolidol | 2.61581484402873e-05 | 4.1363800006775e-05 | 4.16023644015544e-05 | 6.27915564941021e-05 | 3.48129056632035e-05 | 4.31222057965197e-05 | 8.3570776411693e-05 | 5.66856306444175e-05 | 4.34895213511503e-05 | 5.1317239346948e-05 | 5.49552378060747e-05 | 4.41155774495983e-05 |
| pos\_475 | Ganodermic acid TQ | 0.00050796587151869 | 0.000132137582548286 | 0.000249568474314018 | 0.000572752209196239 | 8.33501673904333e-05 | 0.000322599457935943 | 0.000431547658687602 | 0.000715919709931467 | 0.000177804088559116 | 0.000285578243595981 | 0.000521279488591607 | 0.00117130027584542 |
| pos\_476 | (2R)-2-Hydroxy-2-methylbutanenitrile | 2.99466406887494e-05 | 3.27012188699916e-05 | 3.07434346478781e-05 | 2.77726179855708e-05 | 2.76187259577362e-05 | 2.78603765886216e-05 | 2.85734186082052e-05 | 2.92586054376656e-05 | 2.52040995781022e-05 | 2.98555441491986e-05 | 2.92033866213406e-05 | 2.91743412064077e-05 |
| pos\_477 | N-Glycolylneuraminic acid | 7.28296186607106e-06 | 1.21079649818607e-05 | 6.75419470652335e-06 | 1.160046731505e-05 | 8.03865824511026e-06 | 7.66466100540067e-06 | 8.92153353911968e-06 | 6.86030615691842e-06 | 1.38370108798411e-05 | 9.07354909135254e-06 | 1.01067668206999e-05 | 6.81603653201991e-06 |
| pos\_478 | Caprylic acid | 2.84197220954961e-05 | 3.31452033482575e-05 | 9.68574049844933e-06 | 3.02519495850198e-05 | 1.82292054157581e-05 | 2.25825138065152e-05 | 1.98524506575971e-05 | 1.52468409467321e-05 | 6.13754174908103e-06 | 3.81376243450628e-05 | 1.86059857566477e-05 | 8.01494460441848e-06 |
| pos\_479 | Aspartyl-L-proline | 2.90030352934544e-05 | 4.58672838273604e-05 | 2.79881590586377e-05 | 3.79509941347009e-05 | 3.55944228361117e-05 | 3.10889077664365e-05 | 3.46423847886506e-05 | 3.51453991737433e-05 | 3.66931760680582e-05 | 3.48070950156087e-05 | 3.52543694848067e-05 | 4.04801049869308e-05 |
| pos\_48 | (Z)-2-Decene-4,6,8-triyn-1-ol | 1.13340313050466e-05 | 2.00282560456467e-05 | 1.13620586594357e-05 | 1.64512732515474e-05 | 1.72641007927368e-05 | 1.45829965136036e-05 | 1.06729337957819e-05 | 9.90517595758945e-06 | 2.54776932325692e-05 | 1.53386406686877e-05 | 1.39738829197405e-05 | 1.11928292890142e-05 |
| pos\_480 | (13R,14R)-8-Labdene-13,14,15-triol | 6.31792740282317e-05 | 6.61251518758961e-05 | 8.56463043547466e-05 | 6.81921266791464e-05 | 6.71587709403905e-05 | 6.57570026377333e-05 | 7.31749102136135e-05 | 8.02327384175324e-05 | 5.32539982266738e-05 | 0.000116290094330828 | 6.93452523708435e-05 | 8.04236415687857e-05 |
| pos\_481 | Tetrahydro-2-furanmethanol | 2.20921508491378e-05 | 1.30719565998463e-05 | 1.28423693304222e-05 | 1.07628836486115e-05 | 1.32512968785743e-05 | 1.44184592007675e-05 | 1.48059113448261e-05 | 1.36516576967209e-05 | 1.41948422696337e-05 | 1.24753901328296e-05 | 1.32165151322896e-05 | 1.37162260457085e-05 |
| pos\_482 | [6]-Gingerdiol 3-acetate | 1.31641340993887e-05 | 1.65004425327829e-05 | 7.54266436413251e-06 | 1.26068652357149e-05 | 1.17904877765898e-05 | 1.12393646048775e-05 | 1.03411766507943e-05 | 2.58676201438713e-05 | 1.06626420181671e-05 | 1.15056129956915e-05 | 2.22719701613468e-05 | 5.546569348474e-05 |
| pos\_483 | 2(R)-hydroxydocosanoic acid | 4.09575503012032e-05 | 3.35842293597904e-05 | 6.33327673700226e-05 | 8.69867656616911e-05 | 4.46911855602067e-05 | 5.3103090529549e-05 | 4.39534715744406e-05 | 4.85749770460492e-05 | 3.97950711885375e-05 | 4.60431713812347e-05 | 4.49508283117864e-05 | 5.77035487540006e-05 |
| pos\_484 | Acorusdiol | 4.81574703521556e-05 | 4.98997638713477e-05 | 6.23193712269466e-05 | 9.6476156417573e-05 | 3.2215297737195e-05 | 6.022319598577e-05 | 8.14504680065577e-05 | 8.0033310671245e-05 | 8.02132087820679e-05 | 4.19907986049658e-05 | 7.59715020738212e-05 | 9.00117562265995e-05 |
| pos\_485 | Trospium | 1.58655014359478e-07 | 2.70919729528098e-07 | 5.9010341106399e-06 | 8.5752598414646e-08 | 1.55924717562255e-07 | 1.70280156219312e-07 | 0 | 1.38507372174845e-05 | 9.65000009628433e-06 | 2.32110916041544e-07 | 1.04709765231008e-05 | 3.93185737607021e-05 |
| pos\_486 | Biochanin A | 3.34356924463877e-05 | 6.75284224130096e-05 | 2.37983007314302e-05 | 3.75561581927792e-05 | 6.70990755081879e-05 | 4.17723410052813e-05 | 3.86281336130581e-05 | 3.78887812995691e-05 | 5.46003371455595e-05 | 2.53827506226025e-05 | 3.98852868251129e-05 | 4.76803599249174e-05 |
| pos\_487 | (3b,20R,22R)-3,20,27-Trihydroxy-1-oxowitha-5,24-dienolide 3-glucoside | 5.80824845967063e-06 | 6.46974032523375e-06 | 5.98817936688622e-06 | 8.07655734697611e-06 | 6.1101959942681e-06 | 1.17293837589833e-05 | 1.03775775433099e-05 | 1.33832737426798e-05 | 4.01150470592156e-06 | 1.37677915291854e-05 | 1.33231882398193e-05 | 3.75144599379181e-06 |
| pos\_488 | 3,4,5,6-Tetrahydrohippuric acid | 6.98920634425824e-05 | 4.09865495504691e-05 | 1.49808863385266e-05 | 4.49337430873169e-05 | 3.05201500534651e-05 | 2.64886035950766e-05 | 1.62246274066655e-05 | 1.29083499541467e-05 | 1.74165335741013e-05 | 2.30889065246238e-05 | 1.85321853357624e-05 | 1.54005902531664e-05 |
| pos\_489 | Maltoxazine | 0.00040742856133622 | 0.000281832754779845 | 4.22091665010311e-05 | 0.000281469358940993 | 0.000102938925185397 | 0.000176798409267162 | 0.000109793072872156 | 7.60581520132175e-05 | 2.75413141744048e-05 | 0.000244705649866969 | 0.000101328542119896 | 4.97200827266073e-05 |
| pos\_49 | 5-(1-hydroxypropan-2-yl)isolongifol-5-ene | 0.000379297799948573 | 0.000115997791864654 | 6.59845735366147e-05 | 9.19128026976478e-05 | 8.09076624171076e-05 | 7.48142815425405e-05 | 8.42604947561701e-05 | 7.97223143303768e-05 | 9.55868353972665e-05 | 9.52219417846427e-05 | 7.80474700807311e-05 | 7.03440679767848e-05 |
| pos\_490 | Aurantiumal | 0.000114503601888004 | 4.48230375826428e-05 | 0.000346732163150618 | 0.000265795204754609 | 2.88119380104739e-05 | 0.000117760162584035 | 2.83671440631421e-05 | 0.000280341686031273 | 0.00020870181702199 | 2.87579094746336e-05 | 0.000230389752776836 | 0.000716140489996198 |
| pos\_491 | Sotalol | 2.59041894837446e-05 | 4.00742995608078e-05 | 2.27194106626301e-05 | 3.72434155056336e-05 | 2.59814564106699e-05 | 2.783799698442e-05 | 3.40617829183562e-05 | 3.39258901211867e-05 | 2.55611282071472e-05 | 3.54792718181616e-05 | 2.94872048862967e-05 | 2.27411088434078e-05 |
| pos\_492 | Phthalic acid | 0.000153341036005689 | 0.000212903063390111 | 0.000215559844800622 | 0.000166177440830458 | 0.000138566867657203 | 0.00017534549200115 | 0.000164054840992936 | 0.000174691011306292 | 0.000189728356294583 | 0.000166139080876746 | 0.000173565028743269 | 0.000158404169122928 |
| pos\_493 | Isopentenyl acetate | 1.30133744940583e-05 | 1.45390656241974e-05 | 1.51839541019051e-05 | 1.54490667133743e-05 | 1.30042442623314e-05 | 1.39776017932269e-05 | 1.33111564996254e-05 | 1.49776743537207e-05 | 1.62927076546973e-05 | 1.47142654044785e-05 | 1.423349561271e-05 | 1.39881963069243e-05 |
| pos\_494 | Methyl nonanoate | 1.77255139246607e-06 | 4.48346441356838e-06 | 8.32264453365061e-06 | 7.71257988258013e-06 | 1.06773681786501e-06 | 3.45354573158006e-06 | 6.78771325473267e-06 | 5.50450320485847e-06 | 3.82643419823304e-06 | 3.89324993134596e-07 | 8.33964716131121e-07 | 2.85358942439627e-06 |
| pos\_495 | Vildagliptin | 1.67937101017376e-05 | 2.66259582043866e-05 | 1.41008020500817e-05 | 2.47163596806427e-05 | 2.46487539400755e-05 | 1.8595650846333e-05 | 2.35222836192346e-05 | 2.15508387546974e-05 | 1.84823064888643e-05 | 2.17767716780272e-05 | 2.04363752814215e-05 | 1.63125511760561e-05 |
| pos\_496 | Rishitin | 9.59271293881556e-05 | 3.63992231095985e-05 | 2.8440212379531e-06 | 1.99393218821838e-05 | 5.26087361127946e-05 | 4.020931612617e-05 | 1.37009820625277e-06 | 1.74123443259143e-06 | 1.30798496820286e-06 | 2.91206373639248e-05 | 6.20527752725508e-06 | 2.80351544625248e-06 |
| pos\_497 | Sterebin D | 0.000108254262777334 | 9.72525050384548e-05 | 0.0002139280355542 | 0.000164320104578206 | 7.74175258871023e-05 | 0.00012919198348139 | 0.000187509886582556 | 0.000189938983187056 | 0.000204436690197697 | 8.4558408295312e-05 | 0.000182365105643598 | 0.00027018071457303 |
| pos\_498 | MG(0:0/20:2(11Z,14Z)/0:0) | 6.70131270325825e-05 | 5.2002475396746e-05 | 4.35015196136471e-05 | 5.89691174783698e-05 | 6.07690287549578e-05 | 5.64163340563594e-05 | 6.13277019215371e-05 | 5.36102377314601e-05 | 6.21725171570197e-05 | 5.26969264904997e-05 | 5.76483992435896e-05 | 4.83113062297691e-05 |
| pos\_499 | Oleic acid | 0.0001220273147553 | 0.000152793493742802 | 0.00021821705632745 | 0.000158674963288286 | 0.000161892546970291 | 0.000152849080719901 | 0.000248685416943156 | 0.000199621653919405 | 0.000131722691800292 | 0.000173278144895891 | 0.000175514261104457 | 0.000121046131461511 |
| pos\_5 | Citric acid | 1.7141949875766e-05 | 2.32628659038781e-05 | 2.35551606369242e-05 | 1.88136284972956e-05 | 1.52107053614633e-05 | 1.99408127632178e-05 | 1.70803339165001e-05 | 2.00523856788373e-05 | 2.15427918565246e-05 | 2.10442485791111e-05 | 1.97036685645681e-05 | 1.89848086342893e-05 |
| pos\_50 | 8(S)-HETrE | 0.000103852211997956 | 0.000115225720999551 | 7.00812443636403e-05 | 4.42500410484102e-05 | 0.000207468785562173 | 0.000112674077096062 | 7.18923110585073e-05 | 6.5449433195669e-05 | 4.39174819899173e-05 | 7.94586093436374e-05 | 6.98663991004923e-05 | 7.04894132789419e-05 |
| pos\_500 | Leukotriene B4 ethanolamide | 0.000401096927137529 | 0.000267260425385752 | 0.000232328961711273 | 0.000230104831865457 | 0.000238522863033748 | 0.000273710451593784 | 0.000281101856364037 | 0.00025916617306818 | 0.000264849582963216 | 0.000261092923801465 | 0.000268225947606608 | 0.000237167278858166 |
| pos\_501 | 4-Hydroxy-3-(16-methylheptadecyl)-2H-pyran-2-one | 0.000105288381190567 | 0.000127398103336865 | 5.03325190241598e-05 | 0.000128570705716462 | 7.55837301000112e-05 | 0.000110720620756051 | 9.89702327049012e-05 | 8.35059292342852e-05 | 7.41366863323154e-05 | 0.00012677907537723 | 8.15865515970495e-05 | 7.88522431957845e-05 |
| pos\_502 | Dyphylline | 1.02415741967494e-05 | 9.9904659729542e-06 | 5.70195284908242e-06 | 1.09760236450611e-05 | 6.36571863656091e-06 | 9.09533579335524e-06 | 1.01602205819667e-05 | 9.58388422190119e-06 | 1.39491177705114e-05 | 1.19387222447328e-05 | 1.03652206927167e-05 | 9.65719499063558e-06 |
| pos\_503 | 1-Phenyl-1,3-dodecanedione | 1.06235042192574e-05 | 1.30050033635833e-05 | 2.21570942328184e-05 | 1.82382968562636e-05 | 1.85912001998783e-05 | 1.62042668414312e-05 | 1.5235724712333e-05 | 1.67203930296126e-05 | 1.37217726228852e-05 | 1.15912897047535e-05 | 1.43880062751852e-05 | 1.69957461506836e-05 |
| pos\_504 | (2E,4E)-2,4-Hexadienoic acid | 0.000145504845377382 | 0.000129744693599964 | 0.000110247488089171 | 8.86036860443325e-05 | 0.000146612590197978 | 8.94784013295093e-05 | 0.000114400278720885 | 0.000170809356923202 | 7.94438463606398e-05 | 0.000140170237143411 | 0.00012225381865465 | 0.000200070326937177 |
| pos\_505 | Cyclopassifloic acid D | 0.00111616384559738 | 0.00105622333200682 | 0.000858420805832697 | 0.00132645506399055 | 0.00081166286178549 | 0.0010753307186363 | 0.00106198924425161 | 0.00294306118870581 | 0.00150752211063608 | 0.000867389164199693 | 0.00242429901680086 | 0.00482810293085539 |
| pos\_506 | 13S-hydroxyoctadecadienoic acid | 9.4982909910173e-05 | 0.000138472490341802 | 0.00013338078558643 | 0.000174393304409531 | 0.000107880978532644 | 0.000130042827068457 | 0.000115657119540229 | 0.000146777709152828 | 0.000112501990707474 | 0.000115535192847132 | 0.000133785447044438 | 0.00015965553630017 |
| pos\_507 | Androstanedione | 3.75117603407831e-05 | 5.47150378853964e-05 | 2.89197147111e-05 | 3.50639868847066e-05 | 4.41666268333361e-05 | 3.91153235159176e-05 | 3.63212195145251e-05 | 3.13791046705225e-05 | 3.70734993499642e-05 | 4.7209661693271e-05 | 3.80263395841014e-05 | 3.42055447509914e-05 |
| pos\_508 | Salbutamol | 9.25891193637466e-06 | 1.26706973484119e-05 | 5.95192616630801e-06 | 1.18906648100523e-05 | 7.278301852016e-06 | 7.63867521376203e-06 | 9.65457873164495e-06 | 9.97869032994471e-06 | 1.12035898309051e-05 | 9.69129122067269e-06 | 8.85265657369809e-06 | 7.4187574934448e-06 |
| pos\_509 | Riesling acetal | 1.20368825970894e-06 | 2.17077767222521e-06 | 0 | 2.99727348659282e-06 | 1.52453029962736e-07 | 4.32584112072782e-07 | 3.82390865603579e-06 | 1.30285262098871e-06 | 0 | 1.04557246923022e-05 | 3.04600605865141e-06 | 0 |
| pos\_51 | Ile Val | 4.93142255404694e-05 | 7.41278759505312e-05 | 7.03065842556898e-05 | 5.59143019174884e-05 | 0.000150767529714057 | 5.33627257223697e-05 | 7.91902026447362e-05 | 8.27758456543979e-05 | 0.000101467087820668 | 6.07685311790151e-05 | 6.69057085511329e-05 | 9.93653020679153e-05 |
| pos\_510 | Yamogenintetroside B | 2.78933989792199e-05 | 1.02101205737151e-05 | 9.78685453914574e-06 | 6.18191006166697e-06 | 1.20379014616345e-05 | 9.13835912072461e-06 | 5.1013128906542e-06 | 0.0017969994271082 | 4.08142962050539e-06 | 6.31426781232375e-06 | 1.00232876946001e-05 | 2.71578884291517e-05 |
| pos\_511 | Glycyl-Serine | 0.000115688643989372 | 0.000120437096448191 | 4.10910621173792e-05 | 5.5894350131234e-05 | 7.91262271198653e-05 | 5.01794837554278e-05 | 7.08898104748157e-05 | 6.3530049874962e-05 | 5.26906255690063e-05 | 9.12690044041112e-05 | 5.36479807475491e-05 | 6.18898247638895e-05 |
| pos\_512 | 3-Hydroxymugineic acid | 2.26935549072875e-05 | 4.72738677770358e-05 | 6.68848014067442e-06 | 2.05028283774541e-05 | 4.01983932741874e-05 | 2.4288863303427e-05 | 2.0289254029474e-05 | 1.34398593167977e-05 | 1.3741186234385e-05 | 1.93363577971013e-05 | 1.58740565585524e-05 | 7.86116537190732e-06 |
| pos\_513 | N-Oleoyl GABA | 7.33778583536667e-05 | 8.24639424225374e-05 | 0.000138070506773942 | 0.000113145263591644 | 9.97180565006747e-05 | 9.76055921742108e-05 | 8.66679193030045e-05 | 0.000115523128252319 | 9.90178840267099e-05 | 0.000142443817045184 | 0.00011933871565157 | 0.000176745105358049 |
| pos\_514 | Alpha-Linolenoyl ethanolamide | 0.000325637365883268 | 0.000342214189364501 | 0.0006493702505471 | 0.000359656032097035 | 0.000340515083849131 | 0.00040867479061172 | 0.000468897314764264 | 0.000536306742579799 | 0.00032768660070651 | 0.000533946667816136 | 0.000668653830991475 | 0.000589777666522768 |
| pos\_515 | 1-(1-Methylethenyl)-4-(1-methylethyl)benzene | 3.21471521335448e-05 | 4.14125893891365e-05 | 3.9103855742548e-05 | 3.61582461159093e-05 | 2.57101309351492e-05 | 3.42475855962747e-05 | 3.53190078490993e-05 | 3.98628153266783e-05 | 3.46745115368613e-05 | 3.45100413344938e-05 | 3.73235767332199e-05 | 4.66164981179393e-05 |
| pos\_516 | 8-Propanoylneosolaniol | 2.06437678262022e-06 | 1.63273341107559e-06 | 2.21800859053192e-05 | 1.46090151043422e-06 | 1.03188301336327e-05 | 8.01998873078224e-06 | 1.17851879190434e-06 | 1.03619732530222e-05 | 2.17756108505456e-06 | 6.59301091885913e-06 | 7.64598700398476e-06 | 2.28288399198133e-05 |
| pos\_517 | 18-Oxocortisol | 0.000233488180377192 | 0.000135473575047223 | 0.000114978328165134 | 0.000117747327340169 | 0.0001677711192476 | 0.000162507787935069 | 0.000179439390300078 | 0.000148852080002229 | 0.000149444060608259 | 0.000145978859902239 | 0.000147819135772639 | 0.000147786051576002 |
| pos\_518 | Miglustat | 9.89057179458797e-06 | 6.42579432853906e-06 | 2.10272080824438e-06 | 3.17947852136284e-06 | 2.14085932391838e-05 | 3.64735965492536e-06 | 3.24556622997761e-06 | 4.77712840008817e-06 | 4.87992500278252e-06 | 1.16306737700567e-05 | 3.03197018493157e-06 | 2.56514746025043e-05 |
| pos\_519 | 3,11,12-Trihydroxy-1(10)-spirovetiven-2-one | 8.93098762098798e-06 | 8.13900878750723e-06 | 6.2072053257716e-06 | 5.54006517545147e-06 | 1.24394136387624e-05 | 7.95556784333833e-06 | 9.44579587617424e-06 | 9.97672028050323e-06 | 1.3380887338057e-05 | 6.92706367557003e-06 | 8.95401914746044e-06 | 8.48342769122638e-06 |
| pos\_52 | Palmitoyl glucuronide | 0.000238620919983854 | 0.000282903323945906 | 0.000382356376816977 | 0.000405412673011317 | 0.000319068970871977 | 0.000328759960242235 | 0.00048117577068524 | 0.000369767497121807 | 0.000258349263572796 | 0.000317997517341968 | 0.00034283059221155 | 0.0002547505844129 |
| pos\_520 | Momordol | 0.000139416691797614 | 0.000192961452948783 | 0.000278528856450123 | 0.00017053120019998 | 0.000456659667635354 | 0.000240610020652843 | 0.000198572852377827 | 0.000189692652104846 | 0.000578200703534059 | 0.000212850869904067 | 0.000294447230234165 | 0.000210248308288852 |
| pos\_521 | (E,E)-2,4-Octadienal | 4.71432293465448e-05 | 5.78368323115043e-05 | 5.50399749104526e-05 | 5.32209878454788e-05 | 4.50138579107675e-05 | 5.01317311945755e-05 | 4.76119706100821e-05 | 5.33605004091318e-05 | 5.35417219213715e-05 | 5.26701878855522e-05 | 5.219075464152e-05 | 4.84851677814914e-05 |
| pos\_522 | Dihomo-linoleate (20:2n6) | 3.49549176630984e-05 | 4.48282414691188e-05 | 4.04056051726579e-05 | 3.87746195508504e-05 | 6.81956963581218e-05 | 4.8406331325837e-05 | 3.90299172173637e-05 | 4.90325385535382e-05 | 4.84711071942408e-05 | 7.42796558133709e-05 | 4.96745803976948e-05 | 4.74605019916503e-05 |
| pos\_523 | (-)-Nopol | 0.000104701682593734 | 0.000124320705856162 | 0.000118261948232223 | 0.000128358026255926 | 0.000124723760690874 | 0.000113888406446905 | 0.000212325399435373 | 0.000167409274951635 | 0.000117156094281564 | 0.00015629042789099 | 0.000132445892043721 | 0.000135618477286002 |
| pos\_524 | (3S,3'R,5R,6R)-7',8'-Didehydro-3,6-epoxy-5,6-dihydro-beta,beta-carotene-3',5-diol | 2.71325003401138e-05 | 2.88411965389025e-05 | 2.06067784424539e-05 | 3.26308843573943e-05 | 2.20507822762732e-05 | 2.93284605243176e-05 | 3.18661112165202e-05 | 2.70031340423313e-05 | 3.35496726786402e-05 | 1.92714803834065e-05 | 2.98951703549525e-05 | 1.83529539143786e-05 |
| pos\_525 | 2-Dodecanone | 4.40772350459278e-05 | 3.98037565817332e-05 | 8.26323848300024e-05 | 8.46798366333504e-05 | 5.1412029742948e-05 | 6.12747857102854e-05 | 9.54642911993177e-05 | 7.09777020685043e-05 | 5.69218782234482e-05 | 7.07772377119221e-05 | 7.20872317006727e-05 | 5.12810987659365e-05 |
| pos\_526 | Bovolide | 1.31545876425708e-05 | 1.64873876034627e-05 | 1.11617593652718e-05 | 1.22255121807952e-05 | 1.33451380426564e-05 | 1.17785926349661e-05 | 1.15000994527795e-05 | 1.19163196964962e-05 | 1.44974923543866e-05 | 1.29203473698319e-05 | 1.24003166848149e-05 | 1.45646935329027e-05 |
| pos\_527 | Gingerglycolipid C | 0.000201491614933826 | 7.37624668225895e-05 | 6.35906791326212e-05 | 5.78195178699991e-05 | 0.000122276137639992 | 0.000115270680379387 | 0.000105402263363246 | 0.000101652377536013 | 8.15878755188426e-05 | 8.94149653817825e-05 | 9.32609458071116e-05 | 9.59993043726363e-05 |
| pos\_528 | Armexifolin | 2.43985264760864e-07 | 9.928239499794e-08 | 1.00120655311539e-05 | 2.6624346496039e-05 | 1.70906376723384e-06 | 6.00544032028858e-06 | 5.77645527787051e-06 | 2.06309269044215e-05 | 1.2014487304873e-07 | 8.3895282011034e-07 | 1.32320935465271e-05 | 4.7734764498785e-05 |
| pos\_529 | 5-Aminopentanal | 1.14918674895642e-05 | 1.51010737266287e-05 | 1.75181696797191e-05 | 1.80480378755745e-05 | 1.28158077133298e-05 | 1.4635777320698e-05 | 1.93788425786964e-05 | 1.94310263635561e-05 | 1.56852530727645e-05 | 1.58653021741188e-05 | 1.80747672969931e-05 | 2.53674031350223e-05 |
| pos\_53 | Toluene | 1.74178471054485e-05 | 1.41866580604275e-05 | 1.3621529975826e-05 | 1.15332593558026e-05 | 1.24097340098221e-05 | 1.30722922363684e-05 | 1.35653169026241e-05 | 1.33581512597534e-05 | 1.42824551966588e-05 | 1.28973851490553e-05 | 1.31408007083858e-05 | 1.39555520005323e-05 |
| pos\_530 | Phenethyl decanoate | 8.03554711788943e-06 | 9.33788852863654e-06 | 1.09562157659643e-05 | 1.0376938242967e-05 | 9.34480391078835e-06 | 8.90201136179124e-06 | 1.05443081316051e-05 | 1.29060549661608e-05 | 1.00259823724353e-05 | 1.25816149820267e-05 | 1.1447050346349e-05 | 8.45470130412197e-05 |
| pos\_531 | 5-Methyldeoxycytidine | 2.27750298682565e-05 | 2.49090442214905e-05 | 4.55050536484654e-05 | 2.06164411988989e-05 | 1.70211443467653e-05 | 2.58509205615822e-05 | 1.59732360284713e-05 | 2.55543282846474e-05 | 1.92349541936635e-05 | 3.37655388635384e-05 | 2.68354351826145e-05 | 4.12942102892507e-05 |
| pos\_532 | Turmeronol B | 0.000101357540379979 | 7.38980574356583e-05 | 0.000104768487735063 | 0.000105844920062609 | 0.000229134136526987 | 0.000140181496709297 | 0.000175260791425658 | 0.000389101652093631 | 7.29314287665289e-05 | 7.88238074366957e-05 | 0.000277477964065798 | 0.000860120694093544 |
| pos\_533 | Canavaninosuccinate | 6.44227461575229e-08 | 1.81561102823495e-06 | 8.26679251766232e-06 | 8.08783304027327e-07 | 4.33158419460421e-08 | 7.46560718815969e-07 | 9.1453193810445e-07 | 3.589447089193e-07 | 3.98313068000273e-06 | 7.28854368649122e-06 | 1.82566559776486e-06 | 9.20784988246131e-07 |
| pos\_534 | (E)-5-(3,4,5,6-Tetrahydro-3-pyridylidenemethyl)-2-furanmethanol | 0.000197209063517988 | 0.000224736921593063 | 0.000126545809595387 | 0.000215989460466603 | 0.000191588333824087 | 0.000197127074347727 | 0.00021332081532901 | 0.000159035924232289 | 0.000186506943234674 | 0.000230707081947581 | 0.000203593213920081 | 0.000136051960374461 |
| pos\_535 | Proline betaine | 4.5806077378782e-06 | 5.11974761794695e-05 | 4.49553638146234e-06 | 3.74478800997496e-06 | 3.47688944271328e-06 | 1.05994088489768e-05 | 2.17279242071405e-06 | 7.76334077386114e-06 | 2.88223698163553e-06 | 3.63592971562816e-06 | 6.01419216118813e-06 | 2.01813633352723e-05 |
| pos\_536 | Glutamyl-Methionine | 1.99917897073106e-05 | 2.06650851103214e-05 | 2.11862575068564e-05 | 1.94691407026592e-05 | 1.17412507340699e-05 | 1.98914634217417e-05 | 2.01514791534409e-05 | 2.29726811081407e-05 | 1.46014051842312e-05 | 1.70357981198678e-05 | 1.65460749093399e-05 | 1.73161330653289e-05 |
| pos\_537 | 2-(Methylthio)phenol | 2.86398227090749e-05 | 9.09907235605305e-06 | 6.3735396211599e-06 | 1.09287334011104e-05 | 0 | 1.11844773159795e-05 | 0 | 1.33761076598662e-05 | 6.21001168455665e-06 | 2.94445314768683e-06 | 1.08976779773022e-05 | 3.62745579190437e-05 |
| pos\_538 | (E,Z)-2,4-Heptadienal | 1.32804159853335e-05 | 1.75027567954181e-05 | 1.63204270068963e-05 | 1.53196378317263e-05 | 1.51686939956415e-05 | 1.47624928407634e-05 | 1.88690508509279e-05 | 1.85291460616665e-05 | 1.56110578437254e-05 | 1.73335059932873e-05 | 1.61351462468185e-05 | 1.5609542865906e-05 |
| pos\_539 | 5-(12-Heneicosenyl)-1,3-benzenediol | 0.000266137127546409 | 0.000246942095053851 | 0.00036229345687369 | 0.000336534589880867 | 0.000469746944350199 | 0.000331487891420327 | 0.000314281900331671 | 0.00024920345333834 | 0.000420950856961348 | 0.000359239876995837 | 0.000316246732611672 | 0.000204821880475533 |
| pos\_54 | 5,7alpha-Dihydro-1,4,4,7a-tetramethyl-4H-indene | 4.46190459006809e-05 | 6.82317258461423e-05 | 4.80623794616953e-05 | 5.57269725929973e-05 | 4.00703184987928e-05 | 4.90484115445869e-05 | 6.54938463281345e-05 | 5.67447091637185e-05 | 4.42624902442022e-05 | 5.98257413442625e-05 | 5.09792337392416e-05 | 4.56390333479725e-05 |
| pos\_540 | Nonadeca-10(Z)-enoic acid | 1.59624647480711e-05 | 2.34108457087338e-05 | 4.02061727884679e-05 | 2.58009082366361e-05 | 1.70365667859647e-05 | 2.2131692468147e-05 | 1.77409686779092e-05 | 1.71961388482058e-05 | 1.41451688800969e-05 | 1.23036312373006e-05 | 1.8748097929058e-05 | 1.75663332370857e-05 |
| pos\_541 | Tridecanal | 1.34332550549961e-05 | 1.39950753612867e-05 | 2.50699446705901e-05 | 2.08067400881904e-05 | 1.28585311774994e-05 | 1.85665756509579e-05 | 2.58220417372642e-05 | 2.48048649218289e-05 | 1.29399049258107e-05 | 1.74472223849911e-05 | 2.34391058810825e-05 | 2.25250897547846e-05 |
| pos\_542 | 1-(alpha-Methyl-4-(2-methylpropyl)benzeneacetate)-beta-D-Glucopyranuronic acid | 9.4108834942266e-06 | 5.71765806169901e-06 | 4.56018081721467e-06 | 1.01485773195894e-06 | 3.83836222392221e-06 | 2.52082073274049e-06 | 2.92923965800247e-06 | 3.45632151344332e-06 | 4.0820742389298e-06 | 2.81613657186e-06 | 4.0977237585274e-06 | 1.88614495218008e-06 |
| pos\_543 | Acetaminophen glucuronide | 2.39129333178087e-05 | 4.47380093894789e-05 | 2.0725629862753e-05 | 2.86886463189491e-05 | 2.14672540722598e-05 | 2.67973352535063e-05 | 2.15721741956422e-05 | 2.50145711679169e-05 | 0.000140862608934724 | 2.40737449268781e-05 | 5.16072065428865e-05 | 3.36646520549369e-05 |
| pos\_544 | Butyl lactate | 3.09811621211641e-06 | 4.84780694935208e-06 | 5.36089123672398e-07 | 3.47270673645852e-06 | 1.57554643615551e-06 | 2.47913696295078e-06 | 2.13165323654522e-06 | 1.77326260481614e-06 | 3.74951149025193e-07 | 3.89040564151641e-06 | 1.9142519788464e-06 | 4.87125840310949e-07 |
| pos\_545 | NADPH | 8.511028404738e-05 | 1.4920103013366e-05 | 1.98004429371739e-05 | 1.60921607238424e-05 | 4.39509621917918e-05 | 4.49464777215766e-05 | 4.05706478903409e-05 | 3.55000840222893e-05 | 2.63231044376817e-05 | 3.22064198783124e-05 | 2.95965823866339e-05 | 2.95949976086896e-05 |
| pos\_546 | Madasiatic acid | 0.00047347584158972 | 0.000199595631254946 | 0.000151960779804561 | 0.00040759378124557 | 0.00012007733294693 | 0.000287691463596255 | 0.000357295904626928 | 0.000767493411774489 | 0.000318082462931057 | 0.000222338853227525 | 0.000583749466643638 | 0.00132561532451663 |
| pos\_547 | xi-10-Hydroxyoctadecanoic acid | 0.00886883353303315 | 0.0111098068917875 | 0.0103162633031531 | 0.0103957822695137 | 0.00752858544628034 | 0.0095953778106684 | 0.0091787763554891 | 0.00975290798003879 | 0.00935961683548275 | 0.00903773483624899 | 0.00953637383186072 | 0.00897298301039949 |
| pos\_548 | xi-4-Hydroxy-4-methyl-2-cyclohexen-1-one | 1.97600301780675e-05 | 5.47437379426455e-05 | 2.23571003036181e-05 | 1.8194174805177e-05 | 4.01687579652937e-05 | 2.88122426812441e-05 | 2.58285214154328e-05 | 2.20103823238793e-05 | 1.83423374015154e-05 | 3.96136894573019e-05 | 2.47801239935021e-05 | 2.16813624230163e-05 |
| pos\_549 | PG(18:0/22:6(4Z,7Z,10Z,13Z,16Z,19Z)) | 0.0031941530644539 | 0.00181488810019306 | 0.00174804780127426 | 0.0016000451323228 | 0.00192514220007985 | 0.00207532949287492 | 0.00218513992816184 | 0.0020210930748109 | 0.00198009286155382 | 0.00178297125044236 | 0.00194772948154918 | 0.00200804197087076 |
| pos\_55 | iso-Debromo-laurinterol | 1.45447061630854e-05 | 2.10576235721812e-05 | 1.69018885245799e-05 | 2.30121213918114e-05 | 1.82933925842719e-05 | 2.10264807160763e-05 | 1.74073903892021e-05 | 2.1532093995322e-05 | 1.9283077856766e-05 | 1.68165889063981e-05 | 1.88387058257206e-05 | 2.12366324033319e-05 |
| pos\_550 | Avenanthramide 1f | 4.07960217389282e-05 | 0.000137749552255913 | 0.000751838954028861 | 0.00015594058552023 | 4.07290383483853e-05 | 0.000214805216960238 | 0.000137239115551171 | 0.000159741964168342 | 0.00023186156975873 | 0.000477710637646322 | 0.00023290413498367 | 0.00017484658026255 |
| pos\_551 | Torvoside C | 0.000132997958893343 | 4.15612359665249e-05 | 3.79475146232993e-05 | 3.5367025765108e-05 | 7.50790291831682e-05 | 7.50463335968072e-05 | 7.05434420372366e-05 | 6.26506049960872e-05 | 5.12180631807778e-05 | 5.70789216723577e-05 | 5.97925090991556e-05 | 5.75268679457813e-05 |
| pos\_552 | Dihydrodioscorine | 4.18587726901725e-05 | 4.1101077672708e-05 | 3.67557573829384e-05 | 4.49670305154574e-05 | 5.30108401617105e-05 | 4.24173519880864e-05 | 4.31313880061729e-05 | 4.23889643239894e-05 | 4.68383690003549e-05 | 3.79749632175892e-05 | 4.0681909042336e-05 | 3.46903092706545e-05 |
| pos\_553 | 2-Undecyl-4(1H)-quinolinone N-oxide | 4.25421933703633e-05 | 7.51961345227452e-06 | 6.57593377283731e-05 | 3.66570403855907e-05 | 2.82367834432739e-05 | 3.31710133759963e-05 | 3.91471193184135e-05 | 3.19660038749806e-05 | 7.51183530708437e-05 | 0.000101191136040068 | 5.67998860959448e-05 | 3.35709599148199e-05 |
| pos\_554 | Hypogeic acid | 0.000108791190633001 | 0.000150188771088944 | 0.000144383346122002 | 0.000117268853100193 | 8.75874749807656e-05 | 0.000115815425708174 | 9.37291583178701e-05 | 0.000121250967873842 | 0.000145532289267686 | 0.000133447070013296 | 0.000131800301719539 | 0.000128881421100936 |
| pos\_555 | Jasmolone | 2.11505586656537e-05 | 2.10681297640056e-05 | 3.07522727359741e-05 | 2.84540769012029e-05 | 1.98181849127965e-05 | 2.46588841458642e-05 | 2.6480720513762e-05 | 3.01887878699911e-05 | 2.75778735503238e-05 | 2.77020699590005e-05 | 3.12814534739904e-05 | 3.7727886926487e-05 |
| pos\_556 | 5-Hydroxylysine | 6.27018621720282e-05 | 5.6754862312342e-05 | 0.00015370011323259 | 3.75281742938152e-05 | 6.54407166525097e-05 | 7.35396663005011e-05 | 2.73990250584824e-05 | 6.37919141660397e-05 | 2.87684579023928e-05 | 0.000300034522070067 | 7.13008227354274e-05 | 0.000112125869942037 |
| pos\_557 | 2-(2-Methylpropoxy)naphthalene | 1.28571092725151e-05 | 1.69288432421304e-05 | 2.08175208145406e-05 | 2.32711143578859e-05 | 1.85240875951646e-05 | 1.79277822592251e-05 | 2.04347515421285e-05 | 2.04731260451537e-05 | 1.58434546048919e-05 | 1.81552438324705e-05 | 1.92830153564602e-05 | 1.91345009371772e-05 |
| pos\_558 | Homodolicholide | 0.000989022094696876 | 0.00122600399578699 | 0.0012694288772375 | 0.00144888277004211 | 0.00136997465201588 | 0.00126074419566659 | 0.00136814824327124 | 0.00114574451254622 | 0.001169875974523 | 0.0012067495662454 | 0.00117206072146509 | 0.000949336065942851 |
| pos\_559 | 3-Oxododecanoic acid | 2.44887725280841e-06 | 5.24754643057889e-06 | 1.26041870326795e-05 | 2.34547107441732e-06 | 2.13142229986111e-06 | 4.76163902366883e-06 | 4.96256149565593e-06 | 4.15275917028204e-06 | 6.43331700095926e-06 | 1.06653543644505e-06 | 5.16777411883044e-06 | 6.595763378285e-06 |
| pos\_56 | Diosmetin | 1.81674078173458e-05 | 6.49330992293098e-05 | 0.000105101324916849 | 1.63518528248773e-05 | 1.45691951121943e-05 | 3.09910180769802e-05 | 3.56433603019867e-05 | 7.36963039230452e-05 | 0.000188433838062614 | 2.0858604882951e-05 | 6.37609808981355e-05 | 3.65934938199054e-05 |
| pos\_560 | Dodecanedioic acid | 1.78917403303856e-05 | 1.29067051488684e-05 | 7.47245164022502e-06 | 6.36839387671803e-05 | 1.0514132945972e-05 | 1.95564573812467e-05 | 4.11122858104308e-05 | 3.13327267796068e-05 | 1.36404738105465e-05 | 3.32305302138678e-05 | 2.64757669943043e-05 | 1.21853043084788e-05 |
| pos\_561 | Dihydro-2,4-dimethyl-6-(2-methylpropyl)-4H-1,3,5-dithiazine | 4.72381538562916e-06 | 5.76817746517895e-06 | 2.03571422170023e-06 | 4.71864340117701e-06 | 5.45821276289129e-06 | 4.28943519289376e-06 | 6.40853151068507e-06 | 3.77352188523764e-06 | 5.82448433417855e-06 | 4.3093584776515e-06 | 5.05893969379052e-06 | 3.81185971492079e-06 |
| pos\_562 | 11beta-Hydroxy-3,20-dioxopregn-4-en-21-oic acid | 2.16933867639075e-05 | 8.82902503102333e-05 | 5.28360433849556e-05 | 1.21368811714332e-06 | 6.72095359515325e-06 | 3.11011407796033e-05 | 5.63401178568276e-06 | 0.000193473731622224 | 5.63949235845018e-05 | 0.000113178070761147 | 0.000137670543398474 | 0.000466815563658623 |
| pos\_563 | Propenoylcarnitine | 1.07127575623797e-05 | 1.7507910102455e-05 | 1.89748048787713e-05 | 1.47604092794388e-05 | 5.93773413413619e-05 | 1.33168068593452e-05 | 1.68168364700871e-05 | 2.18345810469891e-05 | 3.28166840741414e-05 | 1.07781274099096e-05 | 1.690846424905e-05 | 2.76555326773713e-05 |
| pos\_564 | 5-Hydroxyisourate | 8.0452263493735e-06 | 4.70216381382907e-06 | 2.28085894191934e-06 | 2.82729486949469e-06 | 4.82470785084129e-06 | 4.1910691790661e-06 | 5.34092927433689e-06 | 4.30143446398852e-06 | 3.61266657037324e-06 | 3.45625263939187e-06 | 4.09764988094088e-06 | 3.94775072765851e-06 |
| pos\_565 | (R)-1-O-[b-D-Glucopyranosyl-(1->6)-b-D-glucopyranoside]-1,3-octanediol | 2.66003787523533e-06 | 3.09292007297056e-06 | 2.41233951626678e-05 | 2.84359655805598e-06 | 2.55133217314134e-06 | 3.21530953210135e-06 | 1.76609823712769e-06 | 9.38034511102043e-06 | 2.29012330578879e-06 | 1.6112387998935e-05 | 3.24291714277067e-06 | 1.42348739132397e-05 |
| pos\_566 | 5-(3',5')-Dihydroxyphenyl-gamma-valerolactone | 2.89161297205833e-05 | 2.89760406143326e-05 | 2.58242388454603e-05 | 2.47621045942551e-05 | 2.73091644278558e-05 | 2.71387056378369e-05 | 3.75952614081566e-05 | 2.9027896872106e-05 | 2.61028885515642e-05 | 2.9176330178644e-05 | 2.88513204271666e-05 | 5.53588399181399e-05 |
| pos\_567 | Kaempferol 3-neohesperidoside-7-(2''-p-coumaryllaminaribioside) | 1.52177492429349e-05 | 5.89544923173465e-05 | 1.74977907832139e-05 | 2.65689756651267e-05 | 5.00167846304179e-05 | 3.37915041270083e-05 | 3.57137690293923e-05 | 2.79613226575966e-05 | 6.44696677793019e-05 | 2.11443687189386e-05 | 3.45203490150834e-05 | 1.3283494947502e-05 |
| pos\_568 | 2-Butyl-3-phenyl-2-propen-1-al | 3.96649900950669e-06 | 3.9998945085757e-06 | 6.32515563302387e-06 | 6.14079959949648e-06 | 4.18706050138836e-06 | 4.3388882713076e-06 | 5.56199549939458e-06 | 6.47614868526963e-06 | 5.86413475857289e-06 | 6.05132604814639e-06 | 7.19721488191482e-06 | 8.87040069612297e-06 |
| pos\_569 | Ethyl 2-methylpropanoate | 3.02009666744969e-06 | 3.16101124020039e-06 | 3.62223663822921e-06 | 3.68383855749779e-06 | 4.62304557004538e-06 | 3.28955630337819e-06 | 1.45906386321681e-05 | 8.17567585396086e-06 | 2.9062206647172e-06 | 7.4545635491191e-06 | 5.28558516659657e-06 | 6.47078659710528e-06 |
| pos\_57 | 7-dehydrocholesterol | 0.000317111426469982 | 0.000268266541362598 | 0.000532060590120334 | 0.000342552331579332 | 0.000649105837472185 | 0.000398401189796934 | 0.000356808409143134 | 0.000350587079459861 | 0.000466537391317175 | 0.000465023581727458 | 0.000497351827349046 | 0.000372565808830903 |
| pos\_570 | 6-Hydroxypentadecanedioic acid | 2.66717754728916e-05 | 4.4801673949915e-05 | 0.000109565589986337 | 4.99395132994309e-05 | 3.217356653279e-05 | 5.53122805043279e-05 | 2.54398427255405e-05 | 4.59440019373915e-05 | 2.90905162762114e-05 | 3.54612023143156e-05 | 4.70801752582846e-05 | 5.29600667650057e-05 |
| pos\_571 | (2E,4E)-2,4-Octadien-1-ol | 3.4553801808475e-06 | 5.46383724554258e-06 | 2.18234618563403e-06 | 5.28206508177087e-06 | 2.46000231546952e-06 | 3.64001979871678e-06 | 5.00689973460086e-06 | 3.74647462818941e-06 | 1.51857391903865e-06 | 3.89216607714955e-06 | 3.41646930822821e-06 | 1.70024646303377e-06 |
| pos\_572 | Melilotigenin | 0.00215043171298853 | 0.00200511804472654 | 0.00107741169843167 | 0.00133203103618578 | 0.00298727522494962 | 0.00180211453708181 | 0.00132644453181859 | 0.00125238141285853 | 0.00177501240670595 | 0.00198377275536854 | 0.0014198060210068 | 0.0014227685049987 |
| pos\_573 | Mollicellin A | 6.77819416525189e-06 | 2.77674379497741e-06 | 2.94103757820985e-06 | 1.11604631872984e-06 | 0 | 1.10366704475088e-06 | 4.64976125823559e-07 | 1.05569848508698e-06 | 0 | 0 | 0 | 6.43886043755804e-07 |
| pos\_574 | Oxymesterone | 0.000147711094742385 | 0.000155678060160215 | 0.000406967216003716 | 3.84992970706408e-05 | 0.000226105004280835 | 0.000187451993339507 | 9.90552493746298e-06 | 0.000124874966209826 | 3.16419911544724e-05 | 0.000569798735372742 | 0.000192326958065645 | 0.000293138075515805 |
| pos\_575 | 7,8-Dihydrovomifoliol 9-[rhamnosyl-(1->6)-glucoside] | 2.75354847541096e-05 | 3.90591033898664e-05 | 3.271844656772e-05 | 2.04236479440893e-05 | 2.51370606390775e-05 | 3.20413384844328e-05 | 2.13330019904398e-05 | 3.50464837672864e-05 | 4.51118625014759e-05 | 2.17026069619108e-05 | 3.11221073894797e-05 | 3.6017075649683e-05 |
| pos\_576 | (2S,4R,5S)-Muscarine | 1.98363965719766e-05 | 1.70262099925586e-05 | 1.6563135368695e-05 | 1.92347472818681e-05 | 2.21013989726016e-05 | 1.86777748400444e-05 | 1.71542779908511e-05 | 1.58407328191757e-05 | 2.27876160971963e-05 | 1.72485914966042e-05 | 1.82433377633013e-05 | 1.49823026624159e-05 |
| pos\_577 | Prostaglandin J2 | 3.89068437579715e-05 | 4.7009071832981e-05 | 3.52116041540282e-05 | 9.26893710261284e-05 | 6.74666959768868e-05 | 5.41252701355745e-05 | 5.60962033630596e-05 | 5.19822909366583e-05 | 2.91598763847597e-05 | 3.93958180516179e-05 | 4.88319156822079e-05 | 4.37299227022814e-05 |
| pos\_578 | 2-Methyl-2-cyclopenten-1-one | 5.17078771364461e-05 | 5.14476343591422e-05 | 2.64599744654588e-06 | 2.40572886170949e-05 | 5.23913205674296e-05 | 3.63520918933274e-05 | 2.55969856939223e-06 | 2.82106061566809e-06 | 1.95384253080134e-06 | 2.83574841912859e-05 | 8.2105151227664e-06 | 3.7919636745499e-06 |
| pos\_579 | Sterebin G | 0.00139530620259265 | 0.00154742649200381 | 0.00313964487222849 | 0.00246629061853637 | 0.00481937985164955 | 0.00234261881506753 | 0.00245153750855231 | 0.00184653132767615 | 0.0023083135235239 | 0.00216956953433529 | 0.00157859887640337 | 0.00227697647974371 |
| pos\_58 | Gln Val Asn | 0.000149380036394272 | 0.000109645752526162 | 1.24523210446998e-05 | 7.45264970293775e-05 | 9.29616098739696e-05 | 7.6248103928916e-05 | 1.70274486456102e-05 | 1.44738441518101e-05 | 3.05503657513902e-05 | 8.7587345736383e-05 | 3.07704137044168e-05 | 1.14235651718216e-05 |
| pos\_580 | 6-Decanolide | 5.33383318638246e-06 | 7.01170603834648e-06 | 7.75873215330771e-06 | 9.59290656469534e-06 | 1.0657094605675e-05 | 8.12841487980788e-06 | 7.28633381427673e-06 | 7.60402318280906e-06 | 9.54276797111498e-06 | 7.99566955132166e-06 | 8.95610926001544e-06 | 9.07335783914401e-06 |
| pos\_581 | 1-Tridecene | 1.32549248067285e-05 | 1.09276118891169e-05 | 1.99453865817898e-05 | 2.49371284577949e-05 | 1.77583616271869e-05 | 1.67631949569031e-05 | 1.70192780991888e-05 | 2.87187514518789e-05 | 1.41123104664826e-05 | 2.07710153347753e-05 | 2.28110904046388e-05 | 5.01941679698368e-05 |
| pos\_582 | Ubiquinone-4 | 0.000399323478466076 | 0.00045341232896203 | 0.000521758091932458 | 0.000603024292975482 | 0.000469695136729972 | 0.000505532135385327 | 0.000464735907138769 | 0.000638763482858397 | 0.000498194505156401 | 0.000451197958652254 | 0.000589079157614424 | 0.000919594368851895 |
| pos\_583 | alpha-Tocotrienoxyl radical | 0.000321925359708052 | 0.000358087025734941 | 0.000375747415777573 | 0.000628574903354356 | 0.000495538531250355 | 0.000423508410027666 | 0.000692621262731968 | 0.000553044510811446 | 0.000458846800230623 | 0.000382560078094361 | 0.000501816202817537 | 0.00037778175707785 |
| pos\_584 | Ganolucidic acid B | 0.000174441720764614 | 0.000153795756008002 | 0.000260402905000973 | 0.000236115402238829 | 0.000197953665816843 | 0.000202206452961568 | 0.000417715538233496 | 0.00027427098050331 | 0.000201218658938377 | 0.000212279033237798 | 0.00025269169919509 | 0.000157580853949674 |
| pos\_585 | Phenethyl rutinoside | 0.000438621327822153 | 0.000594926313720711 | 0.000615319322298814 | 0.000710068975965633 | 0.000425887662362368 | 0.000509001502892768 | 0.000529321079107219 | 0.000550488689262437 | 0.00269786479696491 | 0.000535905233383054 | 0.00110253794843686 | 0.000535497848055695 |
| pos\_586 | 12S-HHT | 9.44868075277733e-07 | 8.81406730052692e-08 | 2.11672055257866e-05 | 8.98316016632954e-08 | 6.01301897984704e-07 | 4.22870322406704e-06 | 9.53999109924905e-08 | 2.12952111729984e-07 | 3.17929378854087e-08 | 9.03206719027508e-10 | 5.0770435962377e-08 | 2.23851280037282e-06 |
| pos\_587 | (Z)-7-Hexadecen-1,16-olide | 0.000140072305179633 | 0.000211385195147633 | 0.000176115013091755 | 0.00019755846886711 | 0.000153513104421447 | 0.000174097158536012 | 0.000174683191980329 | 0.000169910133926872 | 0.000190357547499827 | 0.000178106067229475 | 0.000173762234561147 | 0.000158787959078916 |
| pos\_588 | Hovenine A | 0.000441610597224793 | 0.000468261231088348 | 0.000545887247089362 | 0.000597672044438043 | 0.000383233743845296 | 0.000494342682969838 | 0.000532842044136917 | 0.000730366172106616 | 0.000484261704723483 | 0.000401253234330359 | 0.000619294687575156 | 0.00106627882429794 |
| pos\_589 | 15beta-Hydroxydesogestrel | 4.31801570239604e-05 | 4.85659336362393e-05 | 4.21458710310222e-05 | 5.60557509289394e-05 | 5.49032756923666e-05 | 5.45182332473852e-05 | 5.07026558575961e-05 | 5.19279788097304e-05 | 4.97914096753045e-05 | 4.79315305563656e-05 | 5.51780179189588e-05 | 6.06268606618378e-05 |
| pos\_59 | Tyr Phe Phe Phe | 0.00477500413107303 | 0.00600304076101079 | 0.00380371677298607 | 0.00417382264221376 | 0.00547041431492711 | 0.00498875179768075 | 0.00970570895336915 | 0.0087655758858821 | 0.00378494890964076 | 0.0063179932073182 | 0.00671447913101434 | 0.00480797322167162 |
| pos\_590 | Asteltoxin | 0 | 1.09763467868206e-05 | 1.48262364970679e-06 | 0 | 1.95357067054651e-06 | 1.98251969178779e-06 | 0 | 8.12146598677647e-11 | 6.14970240971851e-09 | 8.84046946202307e-06 | 1.5278010065829e-06 | 5.42835656439686e-06 |
| pos\_591 | 19-Hydroxydeoxycorticosterone | 0.000697221826584182 | 0.000473202901571311 | 2.85243125345063e-05 | 2.25054559544737e-05 | 1.70156307326056e-05 | 0.000261289820270171 | 1.89455074967437e-05 | 2.24734209088743e-05 | 1.91739710304297e-05 | 1.81827762636247e-05 | 1.95923537464932e-05 | 2.37156631460568e-05 |
| pos\_592 | Frangulanine | 0.00043398476715908 | 0.0002351280547972 | 0.00022974756228096 | 0.000213359872773926 | 0.000249298446540875 | 0.000259344194123542 | 0.000312447128956717 | 0.000271924125186231 | 0.000248069146890046 | 0.00023832508361828 | 0.000258504138049664 | 0.000259092666242395 |
| pos\_593 | Glucosamine 6-sulfate | 0 | 0 | 0 | 0 | 1.82179650556977e-06 | 0 | 0.000154440885196523 | 0.000112100883115947 | 0 | 2.8239875112424e-07 | 3.50120300622161e-05 | 3.42689200099971e-07 |
| pos\_594 | 19-Norandrosterone | 3.21896786878922e-05 | 3.64017689918814e-05 | 3.08874298982448e-05 | 3.89825789080144e-05 | 4.35416032993489e-05 | 3.63096412942134e-05 | 3.89970815296153e-05 | 4.94832479587252e-05 | 5.53152476175583e-05 | 3.25144247266432e-05 | 4.75316132218211e-05 | 6.38582476443202e-05 |
| pos\_595 | alpha-Amylcinnamyl acetate | 2.222921192298e-05 | 4.1049680437184e-05 | 7.15503023652677e-05 | 6.11872918188289e-05 | 4.05051960328473e-05 | 4.89902362926552e-05 | 3.33333556861879e-05 | 4.46771564885139e-05 | 2.97517112509271e-05 | 2.81662678672743e-05 | 3.75734241320966e-05 | 5.71701201444546e-05 |
| pos\_596 | Homodolichosterone | 0.00043050466431318 | 0.000291286712268489 | 0.00026022389660324 | 0.000391114804972863 | 0.000336526604386629 | 0.000309331667781071 | 0.000447462227999776 | 0.000371633365856943 | 0.000396653633343979 | 0.000341357967751976 | 0.000326197736501811 | 0.000280841787904368 |
| pos\_597 | Heteroxanthin | 0.00323525496873492 | 0.00380872655669279 | 0.00355028250822219 | 0.00392125501630276 | 0.002004681420621 | 0.00381012112313133 | 0.00248922314380684 | 0.0034687844916153 | 0.00445690689321737 | 0.00262025876912165 | 0.00366222611068427 | 0.00346163198770379 |
| pos\_598 | Boswellic acid | 0.00850156447252206 | 0.0107434944177884 | 0.0111357150605334 | 0.0107236478521243 | 0.00810255098021482 | 0.00971843188194255 | 0.0114864891203429 | 0.0114334469826326 | 0.00957360529454295 | 0.0109018362293483 | 0.0108143254782125 | 0.0101966340860061 |
| pos\_599 | 4-(2,6,6-Trimethylcyclohex-1-enyl)but-2-en-4-one | 6.18658742715251e-05 | 8.74098416504708e-05 | 7.34375582319608e-05 | 8.00616748462071e-05 | 6.37164926637838e-05 | 7.27688543156814e-05 | 7.30432172075612e-05 | 7.07553736289892e-05 | 7.89872108435446e-05 | 7.51789069719852e-05 | 7.29753845689733e-05 | 6.27792521782937e-05 |
| pos\_6 | Glycocholic acid hydrate | 0.0128970169875572 | 0.00919076631319798 | 0.0413654460622154 | 0.0272998676202912 | 0.0388246654154162 | 0.0249013139930427 | 0.0110966697988415 | 0.0131500546607033 | 0.0296460093909491 | 0.0269697584520874 | 0.0186025487722596 | 0.0203743641706074 |
| pos\_60 | Vitamin D2 (Ergocalciferol) | 0.000555632562070993 | 0.000620303640115327 | 0.000504795006434508 | 0.000615229731726095 | 0.000507496164553619 | 0.000530207192162903 | 0.000537470926168654 | 0.00055466349785428 | 0.000554775676269156 | 0.000526941065498644 | 0.000527885432083086 | 0.000492078655306418 |
| pos\_600 | Misoprostol | 2.04243251051495e-05 | 2.64813338042548e-05 | 1.78529969795664e-05 | 2.51785966402729e-05 | 3.29497321637978e-05 | 2.43399358506982e-05 | 2.64913533978413e-05 | 2.27853749996118e-05 | 1.89304146735061e-05 | 2.46770109613546e-05 | 2.14551399148902e-05 | 1.93850291162739e-05 |
| pos\_601 | Wyerone epoxide | 1.75061515444507e-07 | 0 | 3.21460915714752e-06 | 6.43461072406986e-06 | 2.37169961499403e-06 | 2.87557551177864e-06 | 5.94683962559322e-07 | 5.09185438113613e-06 | 0 | 3.21728914845737e-08 | 3.64183018147112e-06 | 1.14920156066565e-05 |
| pos\_602 | 2'-Deoxyinosine triphosphate | 5.73959409973564e-06 | 2.15689206539173e-07 | 4.93752919821568e-07 | 1.94157924792372e-07 | 2.53589956682144e-06 | 1.93559658332083e-06 | 2.12755792364357e-06 | 1.38284397494942e-06 | 5.2910955479304e-07 | 1.18591209438977e-06 | 9.84205825903132e-07 | 8.0304548049179e-07 |
| pos\_603 | N-Acetyl-L-tyrosine | 3.02996997649147e-06 | 3.04386983584162e-06 | 1.74375673509531e-06 | 4.28118574230405e-06 | 8.38505103894276e-07 | 2.26916647505199e-06 | 3.74315186199969e-06 | 2.74883126486506e-06 | 9.72494124863458e-07 | 4.5034108657402e-06 | 2.28548120636516e-06 | 2.10839959949205e-06 |
| pos\_604 | Isobutyryl-L-carnitine | 3.73612810041708e-05 | 1.94238819401464e-05 | 1.69079039270105e-05 | 1.90581070574452e-05 | 2.31021724247194e-05 | 2.87628348887176e-05 | 7.11768841617866e-05 | 3.04942704035266e-05 | 3.86119400760111e-05 | 1.30977517702863e-05 | 4.65785504480392e-05 | 6.58776074758063e-05 |
| pos\_605 | Trifolirhizin | 0.000187985353090952 | 0.000150167139763978 | 2.77189881191008e-06 | 5.96786090692995e-05 | 0.000115697510289205 | 9.55602434386027e-05 | 2.27846143645199e-06 | 2.94989121588585e-06 | 3.34088765328241e-06 | 9.75866835780976e-05 | 1.78207061470256e-05 | 1.36235545961718e-05 |
| pos\_606 | 6-Deoxocastasterone | 0.000169216035469563 | 0.000197486541300825 | 0.000159429962464343 | 0.000201322214778289 | 0.000138270980572908 | 0.00019393010940336 | 0.000201765360101703 | 0.000210530735226558 | 0.000156350205698652 | 0.000174581843341222 | 0.000179096754650632 | 0.000187424198849807 |
| pos\_607 | 11-Dehydro-thromboxane B2 | 1.69161386433916e-05 | 1.81069662366979e-05 | 2.85208572625809e-05 | 1.35082442631829e-05 | 1.97937308251504e-05 | 1.89732682655914e-05 | 8.5248497494879e-06 | 1.65117322617721e-05 | 1.22746632887429e-05 | 3.3982500205806e-05 | 1.97697694705651e-05 | 2.52738871908328e-05 |
| pos\_608 | Linden ether | 1.51568781686621e-05 | 1.39337354236216e-05 | 2.2895045502489e-05 | 7.64044759017959e-06 | 1.50277475919411e-05 | 1.71251255805117e-05 | 5.63111548052124e-06 | 1.08292444128517e-05 | 1.91340690458924e-06 | 2.68899077647433e-05 | 1.5492886200473e-05 | 2.13508403830799e-05 |
| pos\_609 | Yuzu lactone | 6.13268593789407e-05 | 6.86196093492e-05 | 6.2295827463967e-05 | 7.35482144791538e-05 | 6.02075900924594e-05 | 6.6306273398183e-05 | 5.91231748984328e-05 | 6.37421188293782e-05 | 7.84154056259019e-05 | 6.66293847013855e-05 | 6.91833023321981e-05 | 6.52594360285066e-05 |
| pos\_61 | 5-Aminoimidazole | 4.26427142678807e-07 | 6.82312864471188e-07 | 5.24068470201801e-07 | 4.93852854832546e-07 | 5.74054583607346e-07 | 3.57993624343856e-07 | 6.1189437859581e-07 | 7.86062368559414e-07 | 5.78870627095859e-07 | 4.04688649415776e-07 | 4.65784180587645e-07 | 9.05332385585907e-06 |
| pos\_610 | Humulinic acid A | 3.17503139739636e-05 | 3.04766015372494e-05 | 5.41448335825821e-05 | 3.22858757554075e-05 | 3.2529107364157e-05 | 2.88123241006414e-05 | 2.08788984137851e-05 | 3.19102364286509e-05 | 1.77275199740329e-05 | 5.84088016599636e-05 | 3.33782960631897e-05 | 5.36564880266685e-05 |
| pos\_611 | (3beta,5alpha,9alpha,22E,24R)-3,5,9-Trihydroxy-23-methylergosta-7,22-dien-6-one | 0.000296999156727174 | 0.000450099424196489 | 0.000278738757182311 | 0.000370113721089681 | 0.000239665268509495 | 0.000320135802582468 | 0.000441171627352305 | 0.00033721636421905 | 0.00026197078733033 | 0.000425033663963688 | 0.000320970115140556 | 0.000212288118358894 |
| pos\_612 | Isorhamnetin | 5.48598413356536e-06 | 0 | 7.82037857975936e-06 | 9.24984547492578e-06 | 2.16594096011924e-06 | 5.47686709023132e-06 | 2.75931814400822e-06 | 3.78098952201236e-06 | 2.7832026578342e-07 | 2.02874031069299e-06 | 3.10801921675926e-06 | 4.79092252641912e-06 |
| pos\_613 | Lucidone B | 0.000190322367600786 | 0.000234937477209338 | 0.000183945221182099 | 0.00019577086687035 | 0.00017847780220551 | 0.000195426747679609 | 0.000169323233373484 | 0.000171255705870762 | 0.000196291462347969 | 0.000203004586982117 | 0.000175606788125871 | 0.000175610428929161 |
| pos\_614 | Fumaric acid | 1.225322730967e-05 | 2.15848907912596e-06 | 5.79251423824653e-09 | 4.88515567118733e-06 | 3.96953458032898e-06 | 4.44914863843261e-06 | 1.26944182144564e-07 | 3.65586288698172e-08 | 1.38615881849787e-07 | 3.84980973540426e-06 | 5.94399002161634e-07 | 4.41359390177307e-08 |
| pos\_615 | N-lactoyl-Leucine | 1.76039856181228e-05 | 2.59211155041504e-05 | 8.68398465128838e-06 | 2.31851575161759e-05 | 6.72198828077506e-06 | 1.51563741100091e-05 | 1.93136609457405e-05 | 1.44916235045092e-05 | 6.68441763618838e-06 | 3.09050478979705e-05 | 1.58404587493593e-05 | 1.26768572025354e-05 |
| pos\_616 | 2-Propenyl phenylacetate | 7.15598545917536e-06 | 1.25522190309115e-05 | 5.37691998374127e-06 | 5.61611641196677e-06 | 7.09637514551889e-06 | 7.31902644182335e-06 | 7.76365050612988e-06 | 7.40637078176442e-06 | 7.88820012786889e-06 | 6.35330491222189e-06 | 8.538212537228e-06 | 1.14151325926958e-05 |
| pos\_617 | 5-Hexyltetrahydro-2-furanoctanoic acid | 6.17368910211235e-06 | 1.44438012345838e-05 | 1.05471702432159e-05 | 8.31000237650506e-06 | 3.7408912369518e-06 | 5.9742591590044e-06 | 6.73081959441149e-06 | 6.89622129974083e-06 | 8.55780754380661e-06 | 7.69051446942671e-06 | 8.61073726235646e-06 | 1.4351855802264e-05 |
| pos\_618 | 11-Ketoetiocholanolone | 3.88494758794828e-05 | 6.93118132154328e-05 | 6.55261184033731e-05 | 2.91581982151937e-05 | 7.77666858076647e-05 | 5.62282753913442e-05 | 2.376998211229e-05 | 4.24039575246725e-05 | 2.61379108525659e-05 | 0.000146140057205093 | 5.17240399306027e-05 | 7.58219218515971e-05 |
| pos\_619 | (E)-5,8-Megastigmadien-4-one | 3.76639356073162e-05 | 6.11932964949795e-05 | 5.4618751234678e-05 | 4.92734079159614e-05 | 3.37663488435036e-05 | 4.65048094892232e-05 | 5.16414404340508e-05 | 5.21255418402809e-05 | 5.15793346350112e-05 | 4.71130441486621e-05 | 5.28866724771312e-05 | 5.72672031979061e-05 |
| pos\_62 | 2,3-dihydrobenzofuran | 0.000266169141670256 | 0.000255960480671547 | 0.00024456447592123 | 0.000195839554671954 | 0.000414130491710455 | 0.000189881571861076 | 0.000378961674879197 | 0.000351004889692497 | 0.000272993085371096 | 0.000327453912440808 | 0.000265669141003076 | 0.000403775548729464 |
| pos\_620 | (3alpha,5alpha,22R,23R)-Cholestane-3,22,23-triol | 0.000236352755873301 | 0.000266751407150645 | 0.00040866364002866 | 0.000390796754409268 | 0.00051210090554533 | 0.000352795328979226 | 0.000327622112680452 | 0.000313007388017298 | 0.00043360560590024 | 0.000454194783617638 | 0.000365024423858677 | 0.000333586149961254 |
| pos\_621 | (R)-1-O-b-D-glucopyranosyl-1,3-octanediol | 4.25432484326998e-05 | 4.91960153638907e-05 | 4.04207625330721e-05 | 5.45063725174609e-05 | 5.96568821098145e-05 | 5.97813002983094e-05 | 5.4665114627879e-05 | 5.43919662160392e-05 | 4.31358821767929e-05 | 6.64174299111858e-05 | 5.90466613830832e-05 | 5.15731133042868e-05 |
| pos\_622 | Venlafaxine | 4.39560472284405e-06 | 2.54185317466323e-06 | 2.3030416968052e-06 | 2.30200751207857e-05 | 1.5006388447792e-06 | 6.06469879274878e-06 | 0 | 1.60270770697033e-06 | 0 | 1.45034191154553e-06 | 9.77801102791842e-07 | 4.34427464161179e-06 |
| pos\_623 | Hydroxytyrosol | 1.49172123598269e-05 | 1.03721138664145e-05 | 1.94641370013245e-06 | 9.25032064199127e-06 | 9.36750223807354e-06 | 8.58367090022182e-06 | 4.11952446630867e-06 | 4.66476413930863e-06 | 2.60735927526665e-06 | 9.74518614486434e-06 | 4.36548800903753e-06 | 3.47204797926638e-06 |
| pos\_624 | Ganoderiol C | 0.000263537721826919 | 0.000340130426494548 | 0.000442770800260075 | 0.000235123589470042 | 0.000284589848611309 | 0.000287326677787498 | 0.00144325414359421 | 0.000993144709806768 | 0.000221654624527421 | 0.00261024534116614 | 0.00118070796833839 | 0.000384684055339534 |
| pos\_625 | Momilactone B | 4.17457409751457e-05 | 3.61723624508968e-05 | 0.000466639198502231 | 0.00140982761543094 | 5.89815055541915e-05 | 0.000225995498526642 | 4.7578178097001e-05 | 0.000433147406254041 | 2.9964583130153e-05 | 4.98693167396028e-05 | 0.000257864101136945 | 0.00113288037292507 |
| pos\_626 | Desglucocoroloside | 4.42655584999599e-05 | 4.51617106145944e-06 | 1.20974497987662e-05 | 1.06379917732939e-05 | 2.17067595902027e-05 | 1.80460103400061e-05 | 2.47939879424849e-05 | 2.10256673769707e-05 | 1.51272403590329e-05 | 1.74974873838562e-05 | 2.34667088956901e-05 | 3.6353875735311e-05 |
| pos\_627 | Cerebronic acid | 2.71742340134253e-05 | 3.54707404183851e-05 | 4.66084054736011e-05 | 3.15530282922871e-05 | 3.38163419382232e-05 | 3.69139721134316e-05 | 2.66678528003933e-05 | 3.589068971504e-05 | 3.38129988233489e-05 | 2.59482688119822e-05 | 3.29892032584901e-05 | 3.28866737042952e-05 |
| pos\_628 | N-Despyridinyl rosiglitazone | 3.36637037118329e-05 | 4.37979617390438e-05 | 4.41134856634531e-05 | 3.76029935822636e-05 | 3.27206335616509e-05 | 3.77912053987938e-05 | 3.43533455122791e-05 | 3.92252303315918e-05 | 4.54733793886955e-05 | 3.55100778003974e-05 | 3.88329977763431e-05 | 3.641282062148e-05 |
| pos\_629 | 11-nitro-1-undecene | 8.519417364834e-05 | 8.58743603630867e-05 | 0.000112723640701346 | 0.000128263124921042 | 0.000293850775105057 | 0.000132265402034943 | 7.86448205799748e-05 | 9.38515002296674e-05 | 0.000137864623474607 | 9.58333163658932e-05 | 9.93330145662708e-05 | 9.82975078523172e-05 |
| pos\_63 | 2-Ethoxy-1-methyl-4-(1-methylethyl)benzene | 1.03667095802709e-05 | 8.39059230870901e-06 | 8.32742772950019e-06 | 1.11403399164706e-05 | 9.77050225431448e-06 | 1.07322191682811e-05 | 1.44390640726621e-05 | 1.05676353613802e-05 | 8.98936458440119e-06 | 9.66339131735332e-06 | 1.09483792491851e-05 | 1.08798219461982e-05 |
| pos\_630 | Linoleamide | 1.36167569255999e-05 | 2.18413235776611e-05 | 1.88311402162448e-05 | 1.87441397759315e-05 | 1.78313996450799e-05 | 1.80675592135646e-05 | 1.64392606953554e-05 | 1.53280102123313e-05 | 1.75637466091872e-05 | 1.83079616798445e-05 | 1.72672959245169e-05 | 1.39079636003651e-05 |
| pos\_631 | Lithocholic acid glycine conjugate | 0.000203394266782354 | 0.00024299662315369 | 0.000131306746626038 | 0.000212303678259394 | 0.000158613641101249 | 0.000185232620581929 | 0.000184902134390936 | 0.000179257148831012 | 0.000152896753933349 | 0.000444877017709728 | 0.000214812038935674 | 0.000170470275048644 |
| pos\_632 | Aminocaproic acid | 8.30151727352664e-06 | 9.49867424972946e-06 | 6.34948561640731e-06 | 6.8516695690348e-06 | 6.94166808737125e-06 | 8.31644913670453e-06 | 1.41342072452094e-05 | 1.41509946367229e-05 | 6.22747193165916e-06 | 7.20774741756862e-06 | 9.69012603276646e-06 | 1.09971364568657e-05 |
| pos\_633 | DG(18:1(11Z)/18:3(6Z,9Z,12Z)/0:0) | 1.29927426583221e-05 | 0.000156544664502999 | 2.08891742060302e-05 | 6.83717275931778e-07 | 5.49614306247589e-06 | 3.4320096487284e-05 | 3.71209525025988e-06 | 1.32478563649358e-05 | 3.41712678418756e-06 | 1.69803811313987e-05 | 4.39774297490102e-06 | 2.09582418995788e-05 |
| pos\_634 | Azythromycin | 0.00810451793319367 | 0.00299173952634722 | 0.00284185025065871 | 0.00250376296829646 | 0.00359534414611747 | 0.00358402455758492 | 0.00383511529288801 | 0.00323318134013391 | 0.00308340553243032 | 0.00306813373471041 | 0.00325000027661451 | 0.00319225873574181 |
| pos\_635 | Demethoxyshogaol | 0.000359222248346786 | 0.00017188701863773 | 3.24825179574629e-05 | 7.46544312062184e-05 | 0.000302991109659571 | 0.000178970464721177 | 0.0005943765158742 | 0.00030932976547552 | 0.00053644734789251 | 0.000290177745956644 | 0.000380095311104121 | 7.28692677487495e-05 |
| pos\_636 | N-Deschlorobenzoyl indomethacin | 5.03298944325199e-05 | 9.17205769842751e-05 | 5.54334970404978e-05 | 5.81022504430982e-05 | 6.56242520718776e-05 | 5.95023596563443e-05 | 4.98934649298721e-05 | 5.24245997468254e-05 | 7.16923309393762e-05 | 5.72688970927125e-05 | 5.59904294213443e-05 | 5.30521308265344e-05 |
| pos\_637 | Leontogenin | 0.000183467945718383 | 0.000130069629674545 | 6.522208052539e-05 | 0.000181926112718783 | 0.000103470769328334 | 0.000135516382246957 | 0.000194879787501632 | 0.00014672047647273 | 9.93385926183494e-05 | 0.000201687262931817 | 0.000158850779491102 | 0.000103452516015841 |
| pos\_638 | 3-Methyl-2-butenal | 3.29106301489375e-05 | 4.3459447825067e-05 | 5.57783476239294e-05 | 3.57148314498279e-05 | 2.95586120877549e-05 | 3.46011288470141e-05 | 3.88160869544015e-05 | 4.41158442117613e-05 | 3.39589445873807e-05 | 4.9573935638887e-05 | 4.20628224343153e-05 | 3.95806666982588e-05 |
| pos\_639 | 4-Hydroxybenzoic acid | 2.66754803129652e-05 | 3.34423623655489e-05 | 3.34619479057325e-05 | 2.59131430266871e-05 | 2.25476268127916e-05 | 2.73574548963389e-05 | 2.60083500519171e-05 | 2.69613169377119e-05 | 3.12204878860758e-05 | 2.79918542743566e-05 | 2.81029259476863e-05 | 2.57482843557697e-05 |
| pos\_64 | Zerumbone | 0.0005104771915586 | 0.000733013503430025 | 0.000722495144312125 | 0.000610536018087228 | 0.000497591864705976 | 0.000613194314977947 | 0.000535969898564337 | 0.000596341441673775 | 0.00071105982608516 | 0.000592190904360231 | 0.000586169038858396 | 0.000577008454068358 |
| pos\_640 | Ginsenoyne G | 1.33758810326036e-05 | 1.39387493985922e-05 | 1.72509008491131e-05 | 1.08503353873641e-05 | 2.72821342987871e-05 | 1.5449640511643e-05 | 9.2325236914094e-06 | 1.14303637948131e-05 | 9.64215162306611e-06 | 4.91867262724236e-05 | 1.66312087371746e-05 | 1.43457224083969e-05 |
| pos\_641 | Sufentanil | 4.32095036628846e-05 | 6.57125224628059e-05 | 5.16678881521121e-05 | 0.000130721748098981 | 5.43257932997666e-05 | 6.36634495751887e-05 | 8.32454422759954e-05 | 9.71711427201366e-05 | 4.46453601280269e-05 | 3.87881055165418e-05 | 7.70750735134565e-05 | 0.000122620730178913 |
| pos\_642 | Yucalexin P15 | 3.64758828573405e-05 | 3.49118325683417e-05 | 7.38295127785624e-05 | 2.56412162721196e-05 | 4.58024367248019e-05 | 4.42560450509906e-05 | 2.33200748977115e-05 | 2.95395433653528e-05 | 2.144928987401e-05 | 7.56007601747813e-05 | 3.82427863346327e-05 | 4.02864088340098e-05 |
| pos\_643 | 2-Hydroxy-6-pentadecylbenzoic acid | 0.000234723368216704 | 0.000155336563980896 | 0.000246874209904949 | 0.000300975747799681 | 0.000202482167469629 | 0.000246121475596114 | 0.000238527461356578 | 0.000388898882222957 | 0.000225303438273199 | 0.000260376582240754 | 0.000259128376620346 | 0.000265871110741339 |
| pos\_644 | Isomasticadienonalic acid | 0.000344122137731848 | 0.000311564398622452 | 0.000284996955134949 | 0.000259169148353744 | 0.000261987855786794 | 0.000284025523660952 | 0.000400894763734309 | 0.000388889466742849 | 0.000350342831665443 | 0.000420750311279888 | 0.000396844013026753 | 0.000404107008488973 |
| pos\_645 | gamma-Tocopheryl quinone | 0.000217989799011873 | 0.000374336254054199 | 0.000254654871194837 | 0.000313322571182529 | 0.000220574027311749 | 0.000280213794499496 | 0.000273072947431275 | 0.000238925433219437 | 0.000291995619433566 | 0.000270568483383906 | 0.000267324366212486 | 0.000198495517132201 |
| pos\_646 | Sorgoleone 358 | 5.89994112596587e-05 | 7.98213466018211e-05 | 4.78092708009057e-05 | 5.82492176575559e-05 | 5.87121438315926e-05 | 5.49199729773835e-05 | 5.76209867609302e-05 | 5.10011173276208e-05 | 6.5151464082612e-05 | 6.64114599196444e-05 | 5.73129231385398e-05 | 5.40323009576271e-05 |
| pos\_647 | 2-hydroxyimipramine | 0.000238556377118179 | 0.000360822376704742 | 0.00021193090997441 | 0.000250659016900546 | 0.000248321091224745 | 0.000245343932964873 | 0.000253371828907311 | 0.000226499456520797 | 0.000265035984623453 | 0.000294645146122881 | 0.000248532345972257 | 0.000207974375268217 |
| pos\_648 | 2-Hexyl-3-phenyl-2-propenal | 9.86380068602443e-06 | 1.43315238009563e-05 | 1.76722269835742e-05 | 1.74076851587728e-05 | 1.14455699500032e-05 | 1.56849585377974e-05 | 1.2428595515232e-05 | 1.74812573891751e-05 | 1.39917560877126e-05 | 1.31705450494222e-05 | 1.58414457087612e-05 | 2.55342689067178e-05 |
| pos\_649 | (S1)-Methoxy-3-heptanethiol | 7.00894184281206e-06 | 6.96676264507099e-06 | 8.64091041823721e-06 | 7.76319219303772e-06 | 7.90035136279744e-06 | 7.1947347126843e-06 | 7.71521993910901e-06 | 8.48368051487771e-06 | 8.0652831015584e-06 | 8.22518454201803e-06 | 8.39925903991586e-06 | 1.06868997144595e-05 |
| pos\_65 | o-Xylene | 3.18549536258197e-06 | 5.35992384486952e-06 | 6.0959046287711e-06 | 5.01476008744978e-06 | 4.38079510344245e-06 | 4.57118277500349e-06 | 5.0859513760165e-06 | 5.67455643402464e-06 | 4.87398054912102e-06 | 5.00269794071354e-06 | 5.02711672918897e-06 | 6.47388625565607e-06 |
| pos\_650 | 4-Hydroxyproline | 1.9961869892202e-05 | 2.82964530762259e-05 | 2.63544929915045e-05 | 2.31649661464362e-05 | 1.9872957184962e-05 | 2.37398038515069e-05 | 2.08640192611359e-05 | 2.33709934009496e-05 | 2.66281370692497e-05 | 2.30463736120143e-05 | 2.25551960953728e-05 | 2.31768153154265e-05 |
| pos\_651 | 1-Acetyl-2-methylcyclopentene | 5.89122130112643e-06 | 5.69368232247247e-06 | 1.08712398064005e-05 | 8.30855610468376e-06 | 5.70939154964813e-06 | 7.92537009624985e-06 | 7.7396151114552e-06 | 8.94201975205297e-06 | 7.66218430922436e-06 | 7.37830940515874e-06 | 8.68802614036398e-06 | 1.14780774534492e-05 |
| pos\_652 | (E,E)-2,6-Octadienal | 1.47672909389116e-05 | 1.09664768321758e-05 | 1.22583044591407e-05 | 1.12895700682823e-05 | 4.89630589027053e-06 | 6.46539758454678e-06 | 8.45141160362783e-06 | 8.97907274229215e-06 | 7.21744946562271e-06 | 1.06439482541199e-05 | 8.54279196765953e-06 | 9.0835554570823e-06 |
| pos\_653 | 1-Heptadecene-4,6-diyne-3,9-diol | 1.36207931094873e-05 | 1.52532125751132e-05 | 1.59967857481488e-05 | 1.83333084020539e-05 | 1.12276845427338e-05 | 1.467294647058e-05 | 2.14862309754213e-05 | 1.87004831609169e-05 | 1.52678212089331e-05 | 1.39765206876644e-05 | 1.87079502158321e-05 | 1.61155391406528e-05 |
| pos\_654 | 2-O-a-L-Fucopyranosyl-galactose | 8.8851479955328e-07 | 1.81110560936568e-07 | 1.22528493024062e-07 | 1.37524451828979e-05 | 9.15183976025702e-08 | 2.12562811027272e-06 | 1.04668918914782e-05 | 4.70238306346148e-06 | 6.3048946410054e-08 | 1.67731421331027e-06 | 3.11415392429472e-06 | 7.11605876086631e-07 |
| pos\_655 | Pterosin E | 1.62788497531985e-05 | 1.24715871476715e-05 | 3.58382719231011e-05 | 7.34331734164378e-05 | 2.29844285175184e-05 | 3.02634415025455e-05 | 1.91266843939816e-05 | 5.39166085046881e-05 | 1.02125769438949e-05 | 1.63615380775235e-05 | 3.81632546493956e-05 | 0.000140193672272642 |
| pos\_656 | O-Desmethylangolensin | 3.77787389955581e-05 | 6.2912327874375e-05 | 3.70917079068217e-05 | 4.25793838774015e-05 | 5.31026640786702e-05 | 4.50283146847695e-05 | 4.32820671671454e-05 | 3.33276190553677e-05 | 5.65201839980896e-05 | 4.58103422397929e-05 | 4.62638311382011e-05 | 3.88477672580984e-05 |
| pos\_657 | Sporotrichiol | 0.00015454704542132 | 0.000148582675521 | 0.000207679706364782 | 0.00058355151560152 | 0.000182044513101203 | 0.000281928070718289 | 0.000147621053583143 | 0.000394792468918732 | 0.000150258901940779 | 0.000148807024116467 | 0.000295270483584121 | 0.000754012273639615 |
| pos\_658 | Neuromedin N (1-4) | 3.44966765344169e-05 | 1.75545541269262e-05 | 9.91758262384742e-06 | 1.36177959683263e-05 | 2.09712454916153e-05 | 2.040076541143e-05 | 2.2906721300896e-05 | 1.5322809257404e-05 | 1.57592687037796e-05 | 1.91987341192284e-05 | 1.74849013677538e-05 | 1.886341885005e-05 |
| pos\_659 | Stearidonic acid | 1.13773937606671e-05 | 1.52586066896035e-05 | 1.40714402639578e-05 | 1.55587587260224e-05 | 1.23713590292549e-05 | 1.32086357406202e-05 | 1.55471697304735e-05 | 1.67875201867441e-05 | 1.41676100222133e-05 | 1.60212715065971e-05 | 1.63811009868414e-05 | 1.87447260724773e-05 |
| pos\_66 | (3beta,17alpha,23S)-17,23-Epoxy-3,28,29-trihydroxy-27-norlanost-8-en-24-one | 0.000576361864217375 | 0.000872991262615206 | 0.000684315850568961 | 0.000958138957873105 | 0.000656593171138687 | 0.000758946353073769 | 0.000833902835091884 | 0.000680020479904648 | 0.00117312695164009 | 0.000671773526125478 | 0.00082157067860241 | 0.000602013259367142 |
| pos\_660 | Octadecanol | 3.31569606701791e-05 | 2.78873193101446e-05 | 2.91930370799155e-05 | 2.54810840070789e-05 | 2.36061905750624e-05 | 2.76099711029694e-05 | 2.69214809816126e-05 | 3.0259597945697e-05 | 2.37402766475948e-05 | 2.85152611403015e-05 | 2.64646312890733e-05 | 2.80836848999902e-05 |
| pos\_661 | 2-O-Feruloyltartronic acid | 9.74252129685079e-05 | 3.59661899476995e-05 | 4.13445582494076e-05 | 0.000111746245024714 | 0 | 6.46951791796957e-05 | 6.19669303693829e-07 | 2.16612351179209e-05 | 9.99365745267877e-06 | 8.69592431023054e-06 | 1.79368855762721e-05 | 5.17316639449486e-05 |
| pos\_662 | All-trans-retinoic acid | 0.000664926843617681 | 0.00087003485426278 | 0.000657320442466358 | 0.000785408108914734 | 0.000466563259652138 | 0.000763996352629228 | 0.00074540944478361 | 0.000828338920527822 | 0.000653155533398609 | 0.000512777100993602 | 0.000751937006058039 | 0.000661059758011816 |
| pos\_663 | Myristic acid | 1.4310497691325e-05 | 1.32664465124293e-05 | 1.79509990813566e-05 | 1.76437909442251e-05 | 1.29513039772099e-05 | 1.52016725454366e-05 | 2.02035645639391e-05 | 1.96319659838073e-05 | 1.77701415678675e-05 | 1.59444122389141e-05 | 1.77189415746886e-05 | 1.93326342918752e-05 |
| pos\_664 | DG(15:0/20:1(11Z)/0:0) | 6.84814096705035e-05 | 2.8272873950208e-05 | 1.23027610144326e-05 | 4.3372899932027e-05 | 3.52163725457263e-05 | 3.82283252488609e-05 | 0.000141765636568425 | 8.45799391074499e-05 | 1.48530996335767e-05 | 3.54397229821669e-05 | 6.55304167564071e-05 | 1.87296363685284e-05 |
| pos\_665 | Isoleucyl-Leucine | 2.70056010385288e-05 | 3.02232308543421e-05 | 3.35000036899708e-05 | 2.30234404906326e-05 | 3.23990983690751e-05 | 2.82168970146825e-05 | 2.04921573490667e-05 | 3.90794261538681e-05 | 1.96559157522007e-05 | 3.02438212313876e-05 | 3.51545123558786e-05 | 6.50610751028217e-05 |
| pos\_666 | CE(20:5(5Z,8Z,11Z,14Z,17Z) | 0.000191747896162005 | 3.90011143649703e-05 | 4.69658995102017e-05 | 3.51492144351073e-05 | 0.000104396381168974 | 9.92603891493601e-05 | 9.03969558075347e-05 | 8.01154241499319e-05 | 5.98987696486692e-05 | 6.07016274045588e-05 | 7.37180628446925e-05 | 6.40612046460785e-05 |
| pos\_667 | 3-Pentadecylphenol | 2.51662438010521e-05 | 3.02962009302937e-05 | 3.16833049151314e-05 | 3.04393847646694e-05 | 2.85674424876353e-05 | 3.21099723462169e-05 | 2.75840937787269e-05 | 2.82962134803731e-05 | 3.17483245605737e-05 | 2.82283954625532e-05 | 2.92027924849591e-05 | 3.24082727235577e-05 |
| pos\_668 | N-Methyl-1-deoxynojirimycin | 8.21479406413203e-06 | 6.12468281109906e-07 | 6.27099704323515e-06 | 1.57925332012464e-05 | 3.08479511139467e-06 | 7.43800561331183e-06 | 4.95657183102352e-06 | 1.08441680079646e-05 | 4.31915305667465e-07 | 1.45866814423317e-06 | 8.20197939079529e-06 | 2.69349807486514e-05 |
| pos\_669 | Glycochenodeoxycholic acid 3-glucuronide | 1.8992224867132e-05 | 2.98417416802036e-05 | 1.18150025929521e-05 | 2.59343310900183e-05 | 4.9916159036052e-05 | 2.74795634397194e-05 | 3.77437258400952e-05 | 2.55583621326289e-05 | 1.97772564030531e-05 | 3.09372473618405e-05 | 2.61739521788582e-05 | 1.74249694944703e-05 |
| pos\_67 | Theasapogenol E | 0.000142859482296795 | 0.00019913509411545 | 9.20229905449816e-05 | 0.000203246811194444 | 6.59926607748409e-05 | 0.000151880448448434 | 0.000120780281394882 | 0.00101352782027482 | 0.000383341273535329 | 7.85284986827763e-05 | 0.000770057536953024 | 0.00202709231737715 |
| pos\_670 | Arborine | 1.85277455714231e-05 | 6.96668025826381e-06 | 4.65587721336798e-06 | 4.17111006557007e-06 | 8.11770780020597e-06 | 5.73131468256602e-06 | 6.83238409445701e-06 | 5.32378331368111e-06 | 4.7499861305747e-06 | 4.43332202798866e-06 | 5.56679365799268e-06 | 7.146967979969e-06 |
| pos\_671 | L-Methionine | 2.01244347666402e-05 | 3.85200184788455e-05 | 1.19055113112656e-05 | 1.9006669058403e-05 | 3.12324919152433e-05 | 2.31039891178303e-05 | 1.12225878389298e-05 | 9.16081067271588e-06 | 2.83738184700423e-05 | 1.77448743717067e-05 | 1.7222075059549e-05 | 9.47010738053908e-06 |
| pos\_672 | Caffeic acid 3-O-glucuronide | 1.33678379694594e-08 | 1.43018464496792e-07 | 4.50124948983438e-06 | 4.32428401443181e-08 | 5.72113042841327e-08 | 3.30376655999706e-08 | 3.89933023311523e-08 | 9.42663487580235e-09 | 2.20620767362426e-09 | 3.86854439638482e-08 | 3.71351680791999e-05 | 7.76220780097233e-09 |
| pos\_673 | Methacycline | 5.72733361819359e-06 | 9.48994892356799e-06 | 1.6324421197564e-05 | 5.11500078096775e-05 | 1.62271252941008e-05 | 4.15054555268959e-06 | 9.78929771772543e-06 | 7.45302618242386e-06 | 1.67138278468914e-05 | 8.32619871722233e-06 | 3.28913108176281e-05 | 2.30789068846998e-05 |
| pos\_674 | 12-Hydroxydodecanoic acid | 0.000198224843621704 | 0.000217541855056894 | 0.000193641420606006 | 0.000207013998162587 | 0.000143623085499421 | 0.000209642496521904 | 0.000222153322607593 | 0.000235222890454292 | 0.000176381823414194 | 0.00017889808019504 | 0.000196621353840337 | 0.000207722387402979 |
| pos\_675 | Crocin 3 | 1.16531481606977e-05 | 7.8396933175969e-06 | 5.25123177566147e-06 | 5.04624147895683e-06 | 7.31500318467026e-06 | 4.28180646621542e-06 | 5.0272322653273e-06 | 2.27376434068084e-06 | 3.28439293971163e-06 | 6.52275168207002e-06 | 3.9615087846994e-06 | 2.61766307248173e-06 |
| pos\_676 | Oxamniquine | 9.85196592743929e-07 | 1.97905756833367e-06 | 4.39697708606564e-06 | 5.14736132543853e-07 | 3.48543672797283e-06 | 1.87256958030991e-06 | 6.43161216397332e-08 | 1.42357353471531e-06 | 1.24775303634512e-07 | 6.70623665968904e-06 | 1.70870117717297e-06 | 3.76869238880342e-06 |
| pos\_677 | 8(17),12-Labdadiene-15,16-dial | 8.06792116643456e-05 | 0.000114760887555833 | 0.000101478180467303 | 9.83566354192708e-05 | 7.36852219620292e-05 | 9.41569886563769e-05 | 8.43541694782079e-05 | 9.25982690253879e-05 | 0.000105426902494084 | 8.26257481832934e-05 | 9.43460681821731e-05 | 8.38762792522399e-05 |
| pos\_678 | Ginsenoyne J | 1.36191399605411e-05 | 1.2068648254828e-05 | 1.07917230086903e-05 | 1.47228429338735e-05 | 1.54686300224511e-05 | 1.43946397886656e-05 | 1.77820820524747e-05 | 1.23364231853683e-05 | 1.23223866926236e-05 | 1.51940514718437e-05 | 1.19976716971501e-05 | 7.16338600409286e-06 |
| pos\_679 | 10,20-Dihydroxyeicosanoic acid | 1.97007264317338e-05 | 1.9467435451815e-05 | 1.34603901265774e-05 | 1.68593804760388e-05 | 1.90203403331741e-05 | 1.75785614203016e-05 | 3.36918710455543e-05 | 2.52155451424681e-05 | 1.72102254789234e-05 | 2.15472817269225e-05 | 2.4057636040264e-05 | 1.83531587708929e-05 |
| pos\_68 | Palmatine | 3.32326556089722e-05 | 4.37864689187511e-05 | 2.48474000837116e-05 | 3.21966555097607e-05 | 0.000106210005365124 | 4.74055081273276e-05 | 0.00269069924247092 | 0.00155163296413404 | 2.78543847064148e-05 | 3.33211491299787e-05 | 0.000980606604310935 | 9.97928299039796e-05 |
| pos\_681 | 2-(3-Carboxy-3-(methylammonio)propyl)-L-histidine | 8.8237083874053e-06 | 1.38636061328586e-05 | 6.27076734986311e-06 | 1.17348581177159e-05 | 9.90392003443817e-06 | 1.01076562255278e-05 | 9.26114288278224e-06 | 7.51703036512747e-06 | 6.07638040487586e-06 | 1.50658406498813e-05 | 8.18544090422151e-06 | 6.36052280669498e-06 |
| pos\_682 | Gonyautoxin VI | 1.27401428796852e-05 | 1.42613321915448e-05 | 3.25680688057449e-06 | 9.49675851007718e-06 | 9.76488056034566e-06 | 9.74837356563592e-06 | 6.89416984307078e-06 | 5.3965065122681e-06 | 3.433244936134e-06 | 1.11786231182836e-05 | 6.49610439181871e-06 | 3.56518039669096e-06 |
| pos\_683 | Linamarin | 1.58279217591608e-05 | 1.54616697170891e-05 | 6.23469511540692e-06 | 1.14881109159001e-05 | 1.14929833625376e-05 | 9.33013063564255e-06 | 9.78815167384329e-06 | 8.68071419025997e-06 | 1.18429082826838e-05 | 8.33900061783522e-06 | 1.13203785363521e-05 | 9.21501664780492e-06 |
| pos\_684 | Cymorcin diglucoside | 9.1334043614106e-05 | 6.78580982921958e-05 | 2.82092602902238e-05 | 2.6565438156603e-05 | 5.67712682422634e-05 | 2.0951478230888e-05 | 4.11809790101411e-05 | 4.52964384644589e-05 | 2.1313952658696e-05 | 6.76309956093165e-05 | 3.32545519989415e-05 | 4.25374268024546e-05 |
| pos\_685 | 4-Hydroxy-2,3,9-trimethoxypterocarpan | 0.000619070978395588 | 0.00153924272672345 | 0.000574914303911769 | 0.000802959016642793 | 0.00117417665531126 | 0.000928934243726128 | 0.00104666022609183 | 0.000806425658764019 | 0.0013437833615968 | 0.000738109246187735 | 0.000916110498448788 | 0.000502547203703577 |
| pos\_686 | Maltol propionate | 6.83180296308139e-05 | 6.90596373594824e-05 | 6.15111299877116e-05 | 5.56440293239698e-05 | 0.000102087180048801 | 5.48865522461095e-05 | 9.08588114514844e-05 | 8.65773805716299e-05 | 7.27789980228488e-05 | 8.01724270605945e-05 | 7.28908658769236e-05 | 9.70993272042588e-05 |
| pos\_687 | Ganoderic acid A | 0.00102085897925639 | 0.00124559845601154 | 0.00122096252166104 | 0.0010227807116113 | 0.000922531321066539 | 0.00110114627980757 | 0.000974935553302013 | 0.0010246072196007 | 0.00123087297624515 | 0.00107087459333202 | 0.00102967334307827 | 0.00100905138496405 |
| pos\_688 | Cyperolone | 0.000140811783914409 | 0.000162246806214411 | 0.000179934968112868 | 0.000159210272601866 | 0.000134261183722508 | 0.000151911374856218 | 0.000131769988027506 | 0.000160259598551729 | 0.00015722959106659 | 0.00016658522961981 | 0.000161353337874434 | 0.0001765524315696 |
| pos\_689 | N-Stearoyl phenylalanine | 3.11175719016698e-06 | 2.0250171680705e-05 | 3.31027825295364e-05 | 2.29848423446978e-06 | 3.46672133520361e-06 | 1.15190025053744e-05 | 3.10786161958304e-06 | 2.83596317647661e-07 | 2.69670087165606e-06 | 1.56414310773519e-06 | 4.48977913852182e-07 | 1.17457791290062e-07 |
| pos\_69 | 3-hydroxyadipic acid 3,6-lactone | 0.000648469700280066 | 0.000475852122277354 | 0.000256570437748426 | 0.000347616375132138 | 0.000491290627272429 | 0.000406914931658778 | 0.000342788603213195 | 0.000316275996425652 | 0.00031521279409279 | 0.000354652343394359 | 0.000315608392832371 | 0.000417880648386063 |
| pos\_690 | Xenognosin A | 4.72786457333103e-05 | 8.14728105151331e-05 | 4.32685128981567e-05 | 6.68862945789461e-05 | 7.31935704931177e-05 | 6.24613569278125e-05 | 4.74001669405667e-05 | 4.20137475570689e-05 | 0.000104381364682598 | 6.74690159934715e-05 | 6.2190544314115e-05 | 4.28731198827385e-05 |
| pos\_691 | Hydroxyisonobilin | 0.000141803073836972 | 0.000599310900117602 | 0.000299139096096789 | 7.08501208785231e-05 | 0.000246604633694292 | 0.000314062704554237 | 4.68480751673857e-05 | 0.00025450715575962 | 7.32935462067634e-05 | 0.000765100888409246 | 0.000285130082566969 | 0.00049201635257771 |
| pos\_692 | IV2Fuc-nLc4Cer | 8.81833752087605e-06 | 9.11945774897101e-06 | 4.23148129084309e-06 | 6.01434273972654e-06 | 9.95166805328634e-06 | 1.03479965469292e-05 | 1.44900264312689e-05 | 1.89726938402228e-05 | 9.46399646787797e-06 | 1.25629335967814e-05 | 1.60331745476873e-05 | 1.36372213968164e-05 |
| pos\_693 | (±)-2-Hydroxy-4-(methylthio)butanoic acid | 1.32116192434419e-05 | 2.00319203531375e-05 | 1.79110085977547e-05 | 1.59974559349861e-05 | 1.12326198066566e-05 | 1.284115093655e-05 | 1.57830965179358e-05 | 1.58513023882035e-05 | 1.96071853949856e-05 | 1.12836211980173e-05 | 1.65892340739719e-05 | 1.18397260808529e-05 |
| pos\_694 | Glucosylsphingosine | 0.000132696397337558 | 0.000174922042980962 | 0.000164365411336634 | 0.000160204388818688 | 0.000134494614545836 | 0.0001926896712056 | 0.000147847086864062 | 0.000179046648638278 | 0.000165159842978206 | 0.000148720493002874 | 0.000180925537586967 | 0.000150176175556809 |
| pos\_695 | Gamma-Linolenic acid | 0.000100248153614844 | 0.000122099226044064 | 0.000174707872634489 | 5.42367113504641e-05 | 0.000104543163428318 | 0.000121333645509616 | 4.3820602727619e-05 | 7.23876654347946e-05 | 5.26930889685398e-05 | 0.000219573565190007 | 0.000101676382716112 | 0.000150367602818436 |
| pos\_696 | 2,5-Dimethyl-4-ethoxy-3(2H)-furanone | 0.00024931827957502 | 0.000144620904310643 | 0.000133431154570173 | 0.000117626458336973 | 0.000148092771306099 | 0.000151905489483268 | 0.000161944758805904 | 0.000151581498908401 | 0.000150100862606852 | 0.000138837116183191 | 0.000143975682904465 | 0.000148134406904711 |
| pos\_697 | 4-Hydroxy-8-methoxy-2H-furo[2,3-h]-1-benzopyran-2-one | 0 | 0 | 0 | 0 | 0 | 1.68302073364577e-07 | 0 | 0 | 5.35383263923174e-07 | 0.000137729724164871 | 7.89122104054784e-07 | 0 |
| pos\_698 | 3-keto Fusidic acid | 0.000991050547480695 | 0.000813961323438916 | 0.000769010672484456 | 0.000781198008155487 | 0.000869909947417332 | 0.00089155780015881 | 0.00158084470483485 | 0.00133638894257461 | 0.000791905141920287 | 0.00114388790406267 | 0.00132928474183864 | 0.00136988322696543 |
| pos\_699 | Ginsenoside C | 0.000178010354432722 | 6.28322576861326e-05 | 4.64600121153038e-05 | 2.52017915023168e-05 | 8.08642651518676e-05 | 8.11735780600598e-05 | 7.11610532857773e-05 | 5.63607921813139e-05 | 4.87668428129021e-05 | 5.11784072117459e-05 | 4.83839525978219e-05 | 5.31549707155666e-05 |
| pos\_7 | mono-Ethyl malonate | 3.78886088570775e-05 | 2.20079422903446e-05 | 1.90638401869903e-05 | 1.73590681322978e-05 | 2.77463653780983e-05 | 3.00512512484781e-05 | 3.09045795289772e-05 | 2.54407155120296e-05 | 2.44433593907572e-05 | 2.39400068801129e-05 | 2.53824734297359e-05 | 3.10924350276327e-05 |
| pos\_70 | Tetralin | 4.00124925046065e-05 | 4.2839432740253e-05 | 3.85023872851492e-05 | 4.10863420515135e-05 | 3.72950776123889e-05 | 4.0607350890926e-05 | 4.08144447912837e-05 | 4.18205425528022e-05 | 4.15365062598204e-05 | 4.21423448787292e-05 | 4.29566819888549e-05 | 4.02228935868626e-05 |
| pos\_700 | 3-keto-Digoxigenin | 1.15043949358866e-05 | 1.32082656219498e-05 | 9.52390327588847e-06 | 1.60785220087485e-05 | 1.48360355605824e-05 | 1.31828742964619e-05 | 7.73432931114024e-06 | 6.12220117355298e-06 | 3.43073986968312e-05 | 1.68062435827027e-05 | 1.67738919435046e-05 | 1.15143689027822e-05 |
| pos\_701 | DG(18:1(11Z)/22:6(4Z,7Z,10Z,13Z,16Z,19Z)/0:0) | 4.67740436106681e-05 | 1.4346166279952e-05 | 1.07329601451441e-05 | 1.52487099070283e-06 | 5.03226597464405e-05 | 2.14445304017033e-05 | 2.60597377801208e-05 | 2.39924960711039e-05 | 1.01982098709336e-05 | 6.27155670543255e-05 | 2.60890109963316e-05 | 2.73543647611793e-05 |
| pos\_702 | CE(18:3(6Z,9Z,12Z)) | 0.000106038853478111 | 6.36864620342559e-05 | 1.04851283264126e-05 | 0.000100499483906207 | 4.95657797514191e-05 | 7.28136212937573e-05 | 0.000149841595003453 | 0.000102057710627905 | 1.07251277781293e-05 | 6.79994088839095e-05 | 7.97995939389615e-05 | 4.73790494587765e-05 |
| pos\_703 | (3beta,22E,24R)-Ergosta-4,6,8(14),22-tetraen-3-ol | 0.00044307572605234 | 0.000358833612174246 | 0.000350694515035789 | 0.00035572829949636 | 0.000309673929102639 | 0.000357346057334937 | 0.000342152383300644 | 0.000337172055203505 | 0.000278799314069497 | 0.000350285058499387 | 0.00033798092078596 | 0.000358194673118355 |
| pos\_705 | 1-(8Z,11Z,14Z-eicosatrienoyl)-glycero-3-phosphate | 7.76024484953493e-05 | 4.51833219174312e-05 | 6.31818079706812e-05 | 6.54832497962747e-05 | 9.60724834269833e-05 | 6.98443616268515e-05 | 0.000145187461304356 | 8.51743365058171e-05 | 5.17850908605798e-05 | 0.000117124857852418 | 9.25524155351138e-05 | 8.71159661218262e-05 |
| pos\_706 | MG(0:0/20:3(8Z,11Z,14Z)/0:0) | 4.64655136254949e-05 | 8.72735694541759e-05 | 4.67788756072958e-05 | 5.10950642012151e-05 | 4.78128306769526e-05 | 5.28711669884595e-05 | 4.02623139269417e-05 | 4.40924549887162e-05 | 7.40336456728572e-05 | 4.49921129364358e-05 | 4.49338218932283e-05 | 3.6621541048378e-05 |
| pos\_707 | 22-Deoxoisocucurbitacin D | 0.000203881133403934 | 0.000248627276401985 | 0.000266651503111499 | 0.000272111158760264 | 0.00013694531267317 | 0.000254455545104402 | 0.000158623464001345 | 0.000232053482253012 | 0.000313058280872905 | 0.000207361235023959 | 0.000241776417229401 | 0.000256879106468888 |
| pos\_708 | alpha-Rotunol | 3.04389640901733e-05 | 4.28090928652104e-05 | 5.99812785695171e-05 | 4.52495716845471e-05 | 3.65257800516232e-05 | 4.14592501978376e-05 | 3.94169876222201e-05 | 5.45846633673887e-05 | 3.5743580560268e-05 | 4.80090372032471e-05 | 4.9308678283424e-05 | 7.53751299477087e-05 |
| pos\_709 | 5-Aminopentanoic acid | 3.84636441905541e-06 | 5.14501265682642e-06 | 4.09599203155669e-06 | 1.40566928600498e-06 | 1.67214121222436e-06 | 2.39614371849852e-06 | 1.91310574577552e-06 | 2.39091187784249e-06 | 1.94878165321629e-06 | 1.65852235472761e-06 | 2.70568730290587e-06 | 2.89401910387595e-06 |
| pos\_71 | Arg Tyr Asp Trp | 0.001590267248154 | 0.00185131798160443 | 0.00109883343622668 | 0.00136901069539682 | 0.00192142760215945 | 0.00165523535723641 | 0.00292279971227078 | 0.00316064783401917 | 0.00133383480448326 | 0.00229403956594142 | 0.00236162036660967 | 0.00156205722808701 |
| pos\_710 | D-Galactopyranosyl-(1->3)-D-galactopyranosyl-(1->3)-L-arabinose | 4.36195328154247e-07 | 6.75066842927753e-07 | 3.75439583286656e-07 | 2.52208468881545e-06 | 2.12352260730988e-09 | 0 | 1.37014083292618e-05 | 4.6712805449675e-07 | 7.05575840897554e-06 | 8.49458117819248e-07 | 3.52636148155893e-07 | 4.35741471873491e-06 |
| pos\_711 | Thioproperazine | 6.33273591918296e-06 | 9.93945069763255e-06 | 7.74726047168391e-06 | 1.28478964055564e-05 | 1.07102213193588e-05 | 8.76759675049788e-06 | 1.10329711368399e-05 | 6.1242149643055e-06 | 1.26497753357389e-05 | 1.92277987389989e-05 | 9.14083787142129e-06 | 1.81839296452655e-06 |
| pos\_712 | Nor-psi-tropine | 9.12446574142677e-06 | 1.02995616398503e-05 | 6.22264874383259e-06 | 1.67435780363199e-05 | 9.986111516374e-06 | 8.19919648349784e-06 | 1.53025072604026e-05 | 1.1598255629159e-05 | 1.21023278636396e-05 | 1.60773069472738e-05 | 1.47580027916707e-05 | 9.82554437988577e-06 |
| pos\_713 | Dodecaprenyl diphosphate | 0.000116341065500429 | 8.47460098345952e-05 | 9.05098715233694e-05 | 9.66370081894302e-05 | 8.69672017300705e-05 | 8.29698741153802e-05 | 0.000101312613970415 | 8.5056398530341e-05 | 0.000103081903351904 | 8.22017027202051e-05 | 8.88503237396158e-05 | 8.4523534996398e-05 |
| pos\_714 | Mevalonolactone | 3.10092999549715e-06 | 3.57359227557217e-06 | 2.81661886975184e-06 | 2.53360654819151e-06 | 4.43761861244226e-06 | 1.99296025766418e-06 | 4.40840176823809e-06 | 3.65068338292367e-06 | 3.47803614499675e-06 | 3.51904492098991e-06 | 3.46216709968228e-06 | 4.84879685607957e-06 |
| pos\_715 | Pogostol | 1.15080186110383e-05 | 2.72580802819857e-05 | 1.58793347434807e-05 | 1.04437966090257e-05 | 8.94821583987525e-06 | 1.43510183411749e-05 | 8.18992067224901e-06 | 1.21627133788029e-05 | 1.80509976611209e-05 | 1.62029862479716e-05 | 1.6438790741265e-05 | 2.0951513266194e-05 |
| pos\_716 | Acetylsoyasaponin A6 | 8.50918560179352e-05 | 0.000173461493517852 | 0.000125800732928003 | 6.61323124667396e-05 | 0.000106104271257001 | 8.15783517828843e-05 | 0.000192334772243538 | 0.000166058088785314 | 8.24471143115015e-05 | 9.2070322064846e-05 | 0.000131999729467206 | 0.000117232305082983 |
| pos\_717 | 2-Hydroxybutyric acid | 1.79739684975258e-05 | 9.40509493768574e-06 | 9.47805022130115e-06 | 8.06478542243687e-06 | 1.00152169485193e-05 | 1.03524220651463e-05 | 1.14807191725708e-05 | 1.04812792847763e-05 | 1.03284011445848e-05 | 9.59001808141937e-06 | 1.02620316628006e-05 | 1.02115789260133e-05 |
| pos\_718 | PC(14:0/18:4(6Z,9Z,12Z,15Z)) | 0.000648971754774824 | 0.000332174176322878 | 0.000324831662779552 | 0.000315901011370951 | 0.000363289969199097 | 0.000405631209734839 | 0.000424102537838008 | 0.000369163915838674 | 0.000372453497071595 | 0.000339972352754739 | 0.000374044490070176 | 0.000387819746237461 |
| pos\_719 | (3beta,23E)-3-Hydroxy-27-norcycloart-23-en-25-one | 9.06078980539471e-05 | 8.44258136324646e-05 | 0.000156202502844254 | 0.000127414503905167 | 8.17180890906108e-05 | 0.000108712958096133 | 0.000148308185600366 | 0.000138233105272336 | 7.99481515159967e-05 | 0.000126343866310212 | 0.000128659578443184 | 0.000114183940197999 |
| pos\_72 | 3,5,7-Trimethyl-2E,4E,6E,8E-decatetraene | 0.000751173256464883 | 0.000944915057635538 | 0.000803430748828013 | 0.000934612390803237 | 0.000760601248323366 | 0.000845549649051623 | 0.000785031908281032 | 0.000810801201420549 | 0.000972311805173099 | 0.000891906707368798 | 0.000883916672228177 | 0.000789213064160627 |
| pos\_720 | Zedoarondiol | 8.43931227198555e-05 | 0.000135202737944185 | 0.000267578385669808 | 0.000191698063087963 | 0.000117698847498851 | 0.000157716047825297 | 0.000131721126942895 | 0.000240016180097681 | 0.000114768213262089 | 9.50591731147064e-05 | 0.00019225843567329 | 0.00100413185712081 |
| pos\_721 | L-2-Aminoethyl seryl phosphate | 1.63937086026899e-06 | 7.35448533859452e-06 | 1.55974133497183e-06 | 4.25528895325979e-06 | 1.94018275320807e-06 | 7.26677890799489e-06 | 3.54434989897288e-06 | 1.50036536241073e-06 | 6.89792501421067e-06 | 4.52937633623956e-06 | 7.52584468427222e-06 | 3.93478475726539e-06 |
| pos\_722 | Eicosenoic acid | 7.35845249779876e-05 | 7.49559048947909e-05 | 9.04469345649543e-05 | 7.24492684244334e-05 | 6.27206538000431e-05 | 7.633456895991e-05 | 4.68301464522697e-05 | 8.57576620817847e-05 | 9.23685671909006e-05 | 5.72498884495349e-05 | 8.50586708513465e-05 | 0.000136963789293327 |
| pos\_723 | Leukotriene A4 | 6.74714769911425e-05 | 0.000104376768319072 | 5.71275258472725e-05 | 7.60749335414565e-05 | 6.4434821112808e-05 | 6.85980183730949e-05 | 6.82052699108457e-05 | 7.16879095134045e-05 | 5.93842883380393e-05 | 7.75647191203714e-05 | 7.93901303081482e-05 | 0.000102922982801291 |
| pos\_724 | 5-Megastigmen-7-yne-3,9-diol 9-glucoside | 3.07557432673601e-05 | 3.57069103717888e-05 | 0.000103508434612739 | 0.000311228846481201 | 4.23826776800271e-05 | 0.000103983541137368 | 3.94345508793051e-05 | 0.000171775764305576 | 2.97533466257696e-05 | 3.43830662352064e-05 | 0.000121533587637615 | 0.000471240930929452 |
| pos\_725 | O-Desmethyltramadol | 1.74173157920491e-05 | 1.31579240636981e-05 | 1.48798621074007e-05 | 1.05836329205182e-05 | 1.15418552770605e-05 | 1.39513322811787e-05 | 1.18568925302823e-05 | 1.26547877840605e-05 | 8.33744328291916e-06 | 1.93793988712722e-05 | 1.30974607090463e-05 | 1.49055150338547e-05 |
| pos\_726 | 5,8,12-Trihydroxy-9-octadecenoic acid | 1.75067267402086e-05 | 3.17011168973286e-05 | 3.37163793815353e-05 | 2.38553697343312e-05 | 2.64698719619103e-05 | 2.41231298846007e-05 | 2.57582783788813e-05 | 3.12862671202697e-05 | 2.36740328087731e-05 | 3.28063004340469e-05 | 2.92084050304168e-05 | 0.000322247164704088 |
| pos\_727 | Cysteinyl-Histidine | 5.68776200170399e-06 | 6.70622114505193e-06 | 9.08181617920631e-06 | 6.66064121318833e-06 | 4.37174501601308e-06 | 7.06913129113044e-06 | 6.23127219493106e-06 | 7.12842858957429e-06 | 6.69150050774805e-06 | 7.23970142251501e-06 | 6.92063622399984e-06 | 5.76886068917702e-06 |
| pos\_728 | Feruloylcholine | 1.43694907468685e-05 | 2.65895155399461e-05 | 2.93527584828289e-06 | 6.06508645058621e-06 | 7.22988850194741e-06 | 6.42541598172304e-06 | 9.41226227960648e-06 | 5.22016621852968e-06 | 7.01291810077975e-06 | 3.34599387158822e-06 | 6.78200599881307e-06 | 6.17606507020573e-06 |
| pos\_729 | Unshuoside A | 0.00230691054739661 | 0.00187797878296073 | 0.00184134995043469 | 0.00153558288627434 | 0.00168123707592745 | 0.00184472358209912 | 0.00177533408994877 | 0.0018301884707914 | 0.00183545415944052 | 0.00177031579195731 | 0.00181488238994744 | 0.00193827145503154 |
| pos\_73 | Benzarone | 0.00136947574564972 | 0.00214854826514763 | 0.00103825458749034 | 0.0017536738310701 | 0.00166630440437014 | 0.00152222246409442 | 0.00160505564890005 | 0.00135103676540319 | 0.00184803787486713 | 0.00161307192975695 | 0.0015350339083709 | 0.00102780662119778 |
| pos\_730 | CE(22:6(4Z,7Z,10Z,13Z,16Z,19Z)) | 7.32914829718659e-06 | 3.20728595847363e-06 | 2.6634165143636e-06 | 1.54900656040647e-06 | 0 | 0 | 9.79957678035747e-07 | 1.1923468115683e-06 | 0 | 1.96719626208425e-06 | 9.19501219554178e-07 | 4.70445665243282e-08 |
| pos\_732 | Isoliensinine | 2.79901092227419e-05 | 5.8876409927532e-05 | 2.10785882940651e-05 | 3.94356157383583e-05 | 3.39661227944123e-05 | 3.23819443692383e-05 | 3.12725125030303e-05 | 2.8453141772642e-05 | 3.72595843800057e-05 | 4.08707474699069e-05 | 3.17611487302882e-05 | 2.34205185164372e-05 |
| pos\_733 | Valyl-Serine | 6.64775802209195e-05 | 8.71705963692301e-05 | 4.04908709762394e-05 | 9.9574053670721e-05 | 2.20938461345481e-05 | 6.13268003751131e-05 | 8.29179909692678e-05 | 6.0085336493065e-05 | 2.70627112463737e-05 | 0.000112386467325524 | 6.5723789923626e-05 | 4.38877153327767e-05 |
| pos\_734 | 5-Hydroxy-4-pentenoic acid d-lactone | 8.51617216831155e-06 | 5.65279280126074e-06 | 4.31866131469692e-06 | 4.52559354753226e-06 | 6.1310635771302e-06 | 6.68722025178919e-06 | 7.33715570763668e-06 | 5.85436800967502e-06 | 5.63766697274822e-06 | 5.53531071110123e-06 | 5.79666603235005e-06 | 7.93832420938372e-06 |
| pos\_735 | Abietinol | 0.000196175860349254 | 0.000204704200994352 | 0.000521047684372174 | 0.00020415530840075 | 0.000154308195843168 | 0.000253349273768681 | 0.000314569696647728 | 0.000271766003621345 | 0.000133523366178511 | 0.0002939393422157 | 0.000248863679986728 | 0.000259563775147533 |
| pos\_736 | 2-Methylpropyl formate | 9.33700879949189e-05 | 0.000101917000749561 | 8.04430295528371e-06 | 5.85902738638851e-05 | 9.55484734785898e-05 | 7.95304484676048e-05 | 5.70906927610043e-06 | 8.16954530371788e-06 | 6.51235243010823e-06 | 7.40526928333921e-05 | 2.40885191279982e-05 | 1.1595345672377e-05 |
| pos\_737 | Norchalciporyl propionate | 2.26490902081681e-06 | 6.7182140027668e-07 | 0 | 4.89499496396846e-07 | 1.30754798014573e-06 | 1.11992690821871e-06 | 9.64122490324092e-07 | 9.74903337090271e-07 | 7.80891089949379e-07 | 1.10973404419494e-06 | 6.57009780684838e-07 | 1.36152190963968e-06 |
| pos\_739 | N(tele)-methylhistaminium | 5.64119558199121e-06 | 6.55065570773027e-06 | 5.11992347791505e-06 | 5.44498640569631e-06 | 4.33425983065358e-06 | 5.26934193393969e-06 | 4.69912263079878e-06 | 5.45039054309844e-06 | 5.43460710544431e-06 | 4.93394786740761e-06 | 5.14613454460263e-06 | 4.91159369946366e-06 |
| pos\_74 | Thr Lys Trp Ser | 0.000111818547926929 | 1.85483064695404e-05 | 2.53886602331968e-05 | 6.13742775210385e-05 | 1.446865107199e-05 | 4.24463848384043e-05 | 1.34287365931353e-05 | 3.13657847680492e-05 | 4.46271722809034e-05 | 1.61949067315788e-05 | 3.68873403229546e-05 | 7.49684994970206e-05 |
| pos\_740 | (3Z,6Z)-3,6-Nonadienal | 5.85153178161076e-05 | 7.02456114393657e-05 | 6.38185619353912e-05 | 7.45129250377935e-05 | 5.76728251613077e-05 | 6.54170797619813e-05 | 6.134876030523e-05 | 6.7793315545622e-05 | 7.66789912274183e-05 | 6.83843183620597e-05 | 6.99798245726129e-05 | 6.33392737272957e-05 |
| pos\_742 | N-acetyl-5-methoxykynuramine | 1.62643856475737e-05 | 1.8589651326806e-05 | 1.0782355748511e-05 | 2.07909980478647e-05 | 1.6642613264265e-05 | 1.62005907049012e-05 | 1.38443909185062e-05 | 1.26714265047982e-05 | 1.11417806322752e-05 | 1.4595352741914e-05 | 1.22014310825296e-05 | 1.079001269186e-05 |
| pos\_743 | Crustecdysone | 0.000111498772023545 | 0.000111149498181917 | 6.04528554392901e-05 | 0.000197713275856894 | 9.52249377054124e-05 | 0.000116130138468871 | 0.000209107003534803 | 0.000140869202976909 | 7.73365170822378e-05 | 0.000248057048031931 | 0.000140806782506903 | 7.85921374199327e-05 |
| pos\_744 | 3-Methyl-3-buten-2-one | 2.57172698169856e-06 | 2.9745590281303e-06 | 2.90694075667324e-06 | 2.73217517403082e-06 | 8.03566120005502e-06 | 4.06676358147248e-06 | 2.32231514116391e-06 | 2.19673983943424e-06 | 1.13002739078602e-05 | 3.12557086164772e-06 | 4.88638855318241e-06 | 2.78625181952933e-06 |
| pos\_745 | Ramipril | 1.73749789284934e-05 | 1.353464842701e-05 | 2.80401743838655e-05 | 2.8624548439457e-06 | 2.92498108060903e-05 | 1.736142696542e-05 | 3.22322825314949e-05 | 2.76576033117089e-05 | 5.00030735438028e-06 | 4.39280675627102e-05 | 2.31887983036095e-05 | 5.1589452665336e-05 |
| pos\_746 | (Z)-10-Hydroxy-8-decene-4,6-diynoic acid | 4.65687637682491e-06 | 3.48882450014912e-06 | 1.00686605455412e-05 | 2.0138405034154e-06 | 5.2553999666292e-06 | 5.41373408917878e-06 | 3.10624643317736e-07 | 4.60508565494373e-06 | 1.61294050863282e-06 | 1.78857681791906e-05 | 5.68611244145298e-06 | 6.13912803364592e-06 |
| pos\_747 | 1-Isothiocyanato-4-phenylbutane | 3.4388280679532e-06 | 2.6409164085044e-06 | 3.85508835594937e-06 | 3.44364807250525e-06 | 2.92719000102254e-06 | 2.77237523020557e-06 | 2.28626650085047e-06 | 3.30110647375336e-06 | 3.29059491261449e-06 | 3.19005044323414e-06 | 3.29987051963003e-06 | 3.53768695107735e-06 |
| pos\_748 | 15d PGD2 | 8.60628576342801e-05 | 0.000108693037643085 | 0.000109689835420493 | 0.000104222793909324 | 7.68287554777533e-05 | 0.000109225006011129 | 7.55308773363374e-05 | 0.000100609368906932 | 9.97181680762889e-05 | 0.000101437388653796 | 0.000101994040973962 | 9.75647443098808e-05 |
| pos\_75 | (R)-4-Phosphopantoate | 0.00148281726260276 | 0.000745062349032702 | 0.000617930255374685 | 0.000233085495048171 | 0.000211766514941123 | 0.000716454424683826 | 7.40341034770505e-05 | 0.000733465847828468 | 0.000206803301195743 | 0.000215420965580574 | 0.000572322438225208 | 0.00174123904990702 |
| pos\_750 | Cepanone | 8.17939791036655e-06 | 2.16535416219702e-05 | 1.92072932618386e-05 | 1.80425909321288e-05 | 7.71721677644854e-06 | 1.24347141426282e-05 | 9.30822240727083e-06 | 2.34928263076072e-05 | 1.82985699337819e-05 | 7.8504404206243e-06 | 1.21382583430125e-05 | 9.85939366686954e-06 |
| pos\_751 | Jubanine B | 1.12305506488488e-05 | 6.27499848243819e-06 | 2.30826334612597e-05 | 1.27986577145982e-05 | 1.38995608071833e-05 | 1.20373856182694e-05 | 4.8753813813226e-06 | 5.8068648675021e-06 | 8.17887235609514e-06 | 1.43856202790042e-05 | 5.47093409050728e-06 | 1.14318733269201e-05 |
| pos\_752 | 3-Methoxy-4-hydroxyphenylglycol glucuronide | 4.97297049717252e-06 | 1.50299793818013e-05 | 8.75531575950258e-06 | 5.25479742916294e-06 | 5.04308296412609e-06 | 1.05154237140285e-05 | 7.43959010789253e-06 | 5.0603480352109e-06 | 8.46660139180586e-06 | 1.68813221501024e-05 | 1.15585592522548e-05 | 9.52285980970723e-06 |
| pos\_753 | Riboflavine 2',3',4',5'-tetrabutanoate | 0.000527254369294535 | 0.000413715653018287 | 0.000217077490023153 | 0.000170256993926771 | 0.000298038872801176 | 0.000349751677819401 | 0.000275433453135815 | 0.000276958238703695 | 0.000322142851177312 | 0.000235447324355372 | 0.000252935771057226 | 0.000263956937065438 |
| pos\_754 | Biocytin | 0.000151635792753487 | 0.000199759569531904 | 0.000149368539300922 | 0.000137634902208214 | 0.000112174727347252 | 0.000138345915451429 | 0.000102732887064961 | 0.0001185649783482 | 0.000162410133632968 | 0.000151344439117337 | 0.000135235742143327 | 0.000138063134944057 |
| pos\_755 | Undecylenic acid | 6.73485081946692e-06 | 1.03642454318242e-05 | 6.39452994082494e-06 | 8.02212529169015e-06 | 7.23215045622873e-06 | 6.24961547197193e-06 | 7.85250194658167e-06 | 8.14965529531878e-06 | 6.03579720074984e-06 | 8.00882774972279e-06 | 7.5912355804042e-06 | 6.03472264168058e-06 |
| pos\_756 | 4-Hydroxycinnamoylagmatine | 8.74356833202825e-06 | 7.70054007339858e-06 | 9.16137504246451e-06 | 4.02913933632412e-05 | 9.63825209048011e-06 | 1.52988838085894e-05 | 2.64279172848849e-05 | 1.79987092315105e-05 | 3.40290364011774e-05 | 2.23637716207015e-05 | 2.38792089673405e-05 | 1.16745656604878e-05 |
| pos\_757 | Humuladienone | 0.000381501335123079 | 0.000486708828581618 | 0.000457338862552796 | 0.000433394377766383 | 0.000364276829362577 | 0.000417450709673149 | 0.000415214509823547 | 0.000472423390436895 | 0.000411328712997253 | 0.000446891414490225 | 0.000445178971958666 | 0.000448107886544816 |
| pos\_758 | ent-6,16-Kauradien-19-oic acid | 8.82434822746657e-05 | 9.69207134174753e-05 | 4.81497039094416e-05 | 6.76939283051002e-05 | 0.000113197468416533 | 7.82963705144123e-05 | 6.26629329260507e-05 | 5.52567980818281e-05 | 7.32547945384977e-05 | 7.99349537647659e-05 | 6.52511178976602e-05 | 6.26390435855922e-05 |
| pos\_759 | 5-L-Glutamyl-L-alanine | 1.25239992886676e-05 | 1.62220733449104e-05 | 6.20395677457765e-07 | 1.34273571957176e-05 | 2.69602249599357e-06 | 6.97375976696273e-06 | 4.73060862180358e-06 | 1.32301726579332e-06 | 1.74482258664973e-08 | 8.03822013144347e-06 | 2.08407762498867e-06 | 1.24633142177034e-06 |
| pos\_76 | (+)-Vulgraon B | 8.8771364516558e-05 | 0.00010886168480892 | 0.000108421229560751 | 0.000100247657166886 | 8.22167807143936e-05 | 9.63441786505119e-05 | 9.37164412332399e-05 | 0.000105101771009337 | 9.60275989406485e-05 | 0.000100723774290722 | 0.000104830776384259 | 9.3916190185865e-05 |
| pos\_760 | 6beta-hydroxybudesonide | 1.32690845071985e-05 | 2.03926340514541e-05 | 1.08062389123216e-05 | 1.58814542573125e-05 | 1.37919495749052e-05 | 1.43411047097191e-05 | 2.85954473590035e-05 | 2.07789572534062e-05 | 1.72148863322547e-05 | 2.60256002751565e-05 | 2.12788302416365e-05 | 1.6478917485083e-05 |
| pos\_761 | 3,4-Methylenedioxyamphetamine | 1.62856703411973e-05 | 1.86079128745075e-05 | 2.08620706998788e-05 | 2.33354610010693e-05 | 1.8657056596267e-05 | 1.85231837658345e-05 | 2.2278779796538e-05 | 2.07962879864502e-05 | 1.84646037175869e-05 | 1.94072506385114e-05 | 1.95617253907738e-05 | 2.02403744705076e-05 |
| pos\_762 | Ondansetron | 3.76280178267886e-06 | 5.14568324797586e-06 | 3.26503846725856e-06 | 4.04033117418594e-06 | 2.44692358733112e-06 | 3.58980709998255e-06 | 4.46207628028193e-06 | 3.72144825641361e-06 | 2.48630207964976e-06 | 4.20065358859001e-06 | 3.5908732434494e-06 | 2.77291627334956e-06 |
| pos\_763 | PC(o-16:0/20:4(8Z,11Z,14Z,17Z)) | 0.0020059518129776 | 0.000551425457708937 | 0.00055159381486817 | 0.000498827874887991 | 0.000832489527865788 | 0.000789951534348184 | 0.00096502807611939 | 0.000724940661272269 | 0.000657689923875457 | 0.000636175650178524 | 0.000703631563355752 | 0.000700093635028893 |
| pos\_764 | Mitomycin | 2.47890004363168e-05 | 6.33313917544329e-06 | 1.30882323098223e-05 | 4.52585653652184e-06 | 4.06046901205346e-05 | 1.73959352510677e-05 | 1.38931812063337e-05 | 9.91548809171893e-06 | 1.65081738097929e-06 | 4.67399327292236e-06 | 7.23729910850697e-06 | 7.03662141249226e-06 |
| pos\_765 | (R)-Shinanolone | 1.75395297869792e-05 | 2.62767296586444e-05 | 8.53925793629469e-05 | 3.41472023964784e-05 | 2.24040689604143e-05 | 3.99402255496914e-05 | 6.47241364362209e-05 | 4.99571932185246e-05 | 2.19228633854961e-05 | 2.23918266725266e-05 | 4.28192019508614e-05 | 5.19100425654579e-05 |
| pos\_766 | L-Arginine | 6.07090346228418e-06 | 3.70880905192575e-06 | 2.76140294782936e-06 | 2.69612904032759e-06 | 1.06608461022956e-06 | 3.29378597233021e-06 | 3.30249905737534e-06 | 4.56558346376901e-06 | 8.34223510463739e-06 | 2.03500181737814e-06 | 5.620879315878e-06 | 7.60708761016944e-06 |
| pos\_767 | N(6)-Methyllysine | 1.62659910597791e-05 | 1.92353333213438e-05 | 2.12354488627685e-05 | 2.38289756555196e-05 | 2.30353585627694e-05 | 2.02189134105444e-05 | 2.13795391804818e-05 | 2.1360149782582e-05 | 2.20395571934913e-05 | 2.12259338554342e-05 | 2.1461878082813e-05 | 2.27517201028997e-05 |
| pos\_768 | (E)-9-(3-Furanyl)-2,6-dimethyl-2,6-nonadien-4-one | 6.39776410495905e-06 | 6.98012791592657e-06 | 6.82319236506707e-06 | 9.0092092227628e-06 | 6.80001415027567e-06 | 7.32022941529872e-06 | 7.23838873468797e-06 | 7.8715757432194e-06 | 7.11559403839812e-06 | 7.2350993183292e-06 | 7.30180857068741e-06 | 7.22851272812407e-06 |
| pos\_769 | Cucurbitaxanthin A | 0.000175473282272463 | 0.000157050365708167 | 0.000119475301527834 | 0.000125680343654282 | 0.000109886080217008 | 0.00013370098499313 | 0.00018367115687446 | 0.00012952231571036 | 0.000138611237182574 | 0.000140848712658297 | 0.000140466994088945 | 0.000123177481470589 |
| pos\_77 | 1-Phenyl-1-pentanone | 4.44252273588882e-05 | 5.20239667217682e-05 | 7.46389551449587e-05 | 7.30737753128931e-05 | 6.7578249441977e-05 | 6.26145526543012e-05 | 6.57066665965372e-05 | 6.12180953115391e-05 | 5.41261027384077e-05 | 5.72268766529809e-05 | 5.77978297671676e-05 | 6.18634277851911e-05 |
| pos\_770 | Valyl-Hydroxyproline | 4.23297519645428e-05 | 5.75983713997858e-05 | 6.32716732765444e-05 | 5.12808713693742e-05 | 4.30562798124434e-05 | 5.09608739078674e-05 | 4.35091725783677e-05 | 4.97659892315111e-05 | 5.18400663383476e-05 | 4.6256062263402e-05 | 4.86028951776953e-05 | 5.2597779469957e-05 |
| pos\_771 | Torvoside E | 0.000597180387317255 | 0.000190299756175465 | 0.000162137231524305 | 0.000137331564233429 | 0.000333786732796369 | 0.000326233174865176 | 0.000297841531440315 | 0.000274343210058319 | 0.000201337772593057 | 0.000221471580518869 | 0.000249088434692633 | 0.000218484946349651 |
| pos\_772 | Azimsulfuron | 0 | 0 | 2.19256740963868e-05 | 9.36830770318069e-06 | 0 | 0 | 0 | 1.41712224674342e-05 | 0 | 1.05699229998112e-05 | 0 | 6.3593061281423e-06 |
| pos\_773 | Dihydroxyacidissiminol | 1.45727048503225e-05 | 1.7640482808802e-05 | 9.40316401514896e-06 | 1.35376743829252e-05 | 1.26236626006136e-05 | 1.34976349327812e-05 | 1.428788520956e-05 | 1.23184737352867e-05 | 1.32361462964835e-05 | 1.91913258385543e-05 | 1.17916202882951e-05 | 1.05348892523221e-05 |
| pos\_774 | Rhubafuran | 2.30074587608427e-05 | 2.17733253817927e-05 | 1.34494950068809e-05 | 3.45281097892059e-05 | 5.42305597905183e-06 | 1.86129486810726e-05 | 2.68836269334884e-05 | 2.21865362285294e-05 | 7.28388083495631e-06 | 2.59842554607723e-05 | 1.97613741319204e-05 | 1.65473456238581e-05 |
| pos\_775 | 1-Acetoxy-2-hydroxy-16-heptadecyn-4-one | 0.000132339544091937 | 0.000145609213388996 | 0.000230106655041302 | 0.000336245636799675 | 0.000219623358545524 | 0.000177495228950707 | 0.000216162814978094 | 0.000286756060706653 | 0.000335500783337831 | 0.000192890262929772 | 0.000196404772185534 | 0.000212825021446379 |
| pos\_776 | Pandamarilactam 3x | 0.000216061658234805 | 0.000143747411270809 | 7.03413114784582e-05 | 0.000105618956903748 | 0.00013308505304734 | 0.000117916080363604 | 0.000120822649984705 | 0.000120981601103501 | 9.36376543043731e-05 | 0.000123181517847502 | 0.000104560029096236 | 0.000101545362213019 |
| pos\_777 | 3'-N'-Acetylfusarochromanone | 4.43606781867282e-06 | 1.05351129929035e-05 | 1.26801515850153e-05 | 1.08409800777121e-05 | 4.93459027684852e-06 | 9.19890252118821e-06 | 6.51653612415438e-06 | 7.73275523405609e-06 | 2.99994103474187e-05 | 1.33336193810654e-05 | 1.28908781427497e-05 | 1.19281092186283e-05 |
| pos\_778 | trans- and cis-2,4,8-Trimethyl-3,7-nona-dien-2-ol | 3.66051897337312e-05 | 4.72952687588243e-05 | 8.72838019075631e-05 | 8.62977809298452e-05 | 0.000104097135263947 | 7.1578421004427e-05 | 4.83199199254534e-05 | 4.94159797888336e-05 | 4.54621097693464e-05 | 6.24474069388977e-05 | 5.09048467890064e-05 | 7.06547540904751e-05 |
| pos\_779 | Neuroprotectin D1 | 3.62285991057562e-05 | 4.90156569197193e-05 | 3.96984731698254e-05 | 4.04792823225938e-05 | 4.42768758913102e-05 | 4.09695935585847e-05 | 2.99836766029916e-05 | 4.00681467553063e-05 | 3.54097042843599e-05 | 5.01321833284996e-05 | 4.05481523307178e-05 | 6.27353352684081e-05 |
| pos\_78 | Chamazulene | 2.20293539055368e-05 | 2.99114703689423e-05 | 3.23515039039629e-05 | 3.28122135174044e-05 | 2.12565232839239e-05 | 2.73831178814384e-05 | 3.19457241714092e-05 | 3.59917192954163e-05 | 2.57096565969071e-05 | 2.62146926302708e-05 | 3.30655657420486e-05 | 4.09373178861702e-05 |
| pos\_780 | Imidazolepropionic acid | 6.14881969940003e-05 | 6.12468972137842e-05 | 9.98308392381382e-05 | 4.15807866287584e-05 | 3.48244756896624e-05 | 5.9705785108423e-05 | 3.4832739228211e-05 | 5.3483552565344e-05 | 7.74297525381186e-05 | 4.79257698826416e-05 | 5.90563135153341e-05 | 7.88607433951961e-05 |
| pos\_781 | Mesifurane | 5.92347335070572e-06 | 5.53097665739494e-06 | 6.82407609906218e-06 | 2.67352598164126e-06 | 5.40735497006843e-06 | 5.7072609423303e-06 | 3.35884721714796e-06 | 3.48963801375799e-06 | 4.45524164085112e-06 | 6.77093917177701e-06 | 4.96862877263016e-06 | 5.27186111740817e-06 |
| pos\_782 | Dopamine 4-sulfate | 1.70174059336797e-05 | 6.9988918423412e-06 | 3.52885157692897e-07 | 3.20022976190233e-08 | 4.59751147366845e-06 | 7.22446913271327e-06 | 9.96769991222835e-07 | 1.1292884213192e-05 | 1.87510683744031e-05 | 1.64616030747029e-11 | 9.29937175207419e-06 | 2.41755080283504e-05 |
| pos\_783 | Limocitrol 3-glucoside | 1.89840910020624e-05 | 2.4328174824409e-05 | 7.20052169739542e-06 | 1.35311865228193e-05 | 1.72806149705917e-05 | 9.09680186941937e-06 | 1.27068731701581e-05 | 8.35372138671339e-06 | 1.33231231703306e-05 | 2.1477284872546e-05 | 8.82911865959922e-06 | 7.45544382009441e-06 |
| pos\_784 | 4-Imidazolone-5-propionic acid | 4.86172910431733e-05 | 6.34320102153401e-05 | 4.2556706635381e-05 | 4.94790213455054e-05 | 4.93719708053957e-05 | 4.46482515065335e-05 | 5.74288592834724e-05 | 5.13092305568723e-05 | 6.33327660523152e-05 | 4.78837136606626e-05 | 5.50152868728133e-05 | 6.24717337215231e-05 |
| pos\_785 | Ixocarpanolide | 0.000782713590380297 | 0.000972816636877189 | 0.000882990176032613 | 0.000785870451457075 | 0.000711369107209524 | 0.000831381298826784 | 0.00075252585309625 | 0.000777171348834158 | 0.000926957924717042 | 0.000843899236383095 | 0.00078083031077136 | 0.000762369564217929 |
| pos\_786 | Oleacein | 4.35961086323528e-05 | 5.31003168085553e-05 | 1.72985725741605e-05 | 2.75096892222773e-05 | 3.50869949755493e-05 | 3.17837078504564e-05 | 2.47695226798784e-05 | 2.26863412640072e-05 | 2.98574269299153e-05 | 3.47078534370807e-05 | 2.42357515544226e-05 | 2.30278690578581e-05 |
| pos\_787 | Amlexanox | 4.84334857776385e-05 | 6.9532606508231e-05 | 6.70646356974576e-05 | 6.01563588864547e-05 | 4.87152311227749e-05 | 6.89434045889667e-05 | 5.27260296236063e-05 | 6.27620175132449e-05 | 6.28447943336518e-05 | 7.22053648037267e-05 | 5.77807475523785e-05 | 5.74072370919069e-05 |
| pos\_788 | Cucurbitacin D | 0.000168127480883667 | 0.00019488696449303 | 0.000469789809187053 | 0.000304855029293355 | 0.000149347362083093 | 0.00022288814807394 | 0.000121467401026728 | 0.000291245107470733 | 0.000176454848726816 | 0.00011866809494542 | 0.000189735628440158 | 0.000373572391165496 |
| pos\_789 | Deserpidine | 3.74113939195627e-06 | 8.15865819679268e-06 | 6.28416973753884e-06 | 4.75912352313128e-06 | 5.69610070420909e-06 | 5.61984525218783e-06 | 1.08350618990939e-05 | 9.73112241421872e-06 | 3.11163969080171e-06 | 4.37004819898107e-06 | 1.11008541265968e-05 | 1.32194872681073e-05 |
| pos\_79 | 7alpha,17beta-Dihydroxyandrosta-1,4-dien-3-one | 0.000342564243791493 | 0.000405126910711075 | 0.000753834778659823 | 0.000104808855727014 | 0.000449207685650533 | 0.000422674175621741 | 4.09429622371884e-05 | 0.000276792229315957 | 0.000100495809246759 | 0.000947032133589361 | 0.000407072277839852 | 0.000614540063991692 |
| pos\_790 | Petasalbin | 1.13246180202467e-05 | 1.61184030406619e-05 | 1.56335391190289e-05 | 1.50450381621121e-05 | 1.13264435747687e-05 | 1.36970562162887e-05 | 1.49934335350397e-05 | 1.61554217459015e-05 | 1.40743215322712e-05 | 1.66784927050904e-05 | 1.56798001286502e-05 | 1.82797519706699e-05 |
| pos\_791 | 3'-O-Methyl-(-)-epicatechin-5-O-sulphate | 8.94011629656901e-06 | 8.73984124689404e-06 | 5.16949272499164e-06 | 4.67649903593026e-06 | 3.86688665710266e-06 | 7.54254342114876e-06 | 9.03801002804621e-06 | 8.53896421998452e-06 | 5.74427731773114e-06 | 5.58174874767063e-06 | 5.90410022964088e-06 | 6.95797497514686e-06 |
| pos\_792 | Bethanidine | 3.70669771540861e-06 | 1.72100534195383e-07 | 5.42636394394296e-07 | 3.03953218728413e-06 | 1.37188185843633e-06 | 1.81354669110114e-06 | 1.67468118967039e-07 | 2.48270594460581e-06 | 2.11297995001013e-06 | 9.88268957669496e-09 | 1.3202588400722e-07 | 9.97810843637119e-07 |
| pos\_793 | O-Phosphotyrosine | 1.16700972601404e-06 | 4.64924545431314e-07 | 2.79453583207232e-06 | 4.8793432354e-06 | 7.55514656142029e-07 | 2.4852896666329e-06 | 3.98001401318848e-06 | 2.0831457647835e-06 | 2.71014534228185e-06 | 2.24986890390449e-06 | 3.36489290624688e-06 | 8.44114995237718e-07 |
| pos\_794 | (2R\*,3R\*)-1,2,3-Butanetriol | 2.0624122974569e-05 | 1.46379570615423e-05 | 1.10338078495372e-05 | 1.06239099973428e-05 | 1.55945931870363e-05 | 1.57580525824399e-05 | 1.564923094553e-05 | 1.43959360626442e-05 | 1.45437546726915e-05 | 1.36312807290962e-05 | 1.42672130325977e-05 | 1.41193864939848e-05 |
| pos\_795 | 2a-Hydroxygypsogenin 3-O-b-D-glucoside | 0.00257281336358537 | 0.00334287591930492 | 0.00345384119801687 | 0.00284686026326806 | 0.00248154714595578 | 0.00323969251172421 | 0.00267721809771985 | 0.00294652780252474 | 0.00355113884764176 | 0.0030974259938808 | 0.00292722627350316 | 0.00279094207108832 |
| pos\_796 | DG(14:1(9Z)/22:2(13Z,16Z)/0:0) | 8.00320851820167e-05 | 0.000472889819549469 | 0.000188403929269286 | 1.30825326270966e-05 | 5.19665694463527e-06 | 0.000143585923887035 | 4.51099557054268e-05 | 7.24897696464101e-05 | 2.92301929068626e-05 | 8.41768770235757e-05 | 6.4016712019458e-05 | 0.000107223184138435 |
| pos\_797 | 4',7-Dihydroxy-2',5-dimethoxyisoflavone | 9.74098282751402e-06 | 1.02987339641388e-05 | 1.12669253671025e-05 | 7.49810222989929e-06 | 9.32303536011331e-06 | 9.37631122317981e-06 | 6.50473049576552e-06 | 8.15904848004673e-06 | 9.73957734971371e-06 | 9.56190261872453e-06 | 8.88114341758611e-06 | 1.09788764553245e-05 |
| pos\_798 | gamma-L-Glutamyl-L-methionine sulfoxide | 1.6956750080396e-06 | 7.77793873331401e-06 | 8.00830256888349e-06 | 1.47276165540698e-05 | 1.503468386856e-05 | 1.0532336708053e-05 | 1.03040667462538e-05 | 1.02725438747236e-05 | 1.88154000531412e-05 | 2.40528488564514e-05 | 1.31779001560826e-05 | 8.45664959978502e-06 |
| pos\_799 | Diacetone alcohol | 1.8979968658301e-06 | 2.86704723557212e-06 | 4.17704110088075e-06 | 3.22793255259153e-06 | 2.18223508355408e-06 | 2.78329922492643e-06 | 2.00000517282158e-06 | 3.17852739502151e-06 | 2.21799361988241e-06 | 2.24113230274494e-06 | 2.70471291020569e-06 | 4.71319830807031e-06 |
| pos\_8 | Taurodeoxycholic acid | 0.00138202319064426 | 0.00068987652824343 | 0.00237337771977071 | 0.00215658417843218 | 0.00242383231171403 | 0.00171436206332092 | 0.00109435970511982 | 0.00113143384477504 | 0.00239670867363341 | 0.00181911068439579 | 0.00145140349089305 | 0.00163006775409085 |
| pos\_80 | Elongatin | 1.29741489831768e-07 | 4.71488581526651e-07 | 8.84900869298086e-07 | 3.93748816067242e-07 | 5.21422692070065e-07 | 1.01970952162469e-07 | 2.3510280224479e-07 | 4.60896655341829e-07 | 3.47250947561731e-05 | 7.36011903063509e-07 | 6.24619507206395e-06 | 2.89211874857326e-07 |
| pos\_800 | DG(18:0/20:4(8Z,11Z,14Z,17Z)/0:0) | 0.000187431361258745 | 2.09890453403597e-05 | 1.9565414940183e-05 | 8.60981255251959e-05 | 3.57012089739008e-05 | 8.4101707846436e-05 | 5.77852265496502e-05 | 6.35223886732849e-05 | 1.90962012111712e-06 | 1.47975116155188e-05 | 4.52500330932546e-05 | 7.35907317951491e-05 |
| pos\_801 | Gamma-linolenyl carnitine | 6.97683097040118e-05 | 5.96156186872091e-05 | 6.52672017095633e-05 | 0.000109405885108811 | 5.83630267140533e-05 | 7.39720482676335e-05 | 8.40383767195575e-05 | 7.12412672988057e-05 | 5.75247935833479e-05 | 5.86540818509133e-05 | 6.59137087922086e-05 | 5.69089842552736e-05 |
| pos\_802 | Narirutin | 0.000196594603105606 | 4.72389342461669e-05 | 5.42374819273774e-05 | 3.63379868805037e-05 | 0.000107413970992254 | 0.000102733820994722 | 9.41610224731319e-05 | 8.26241305921812e-05 | 6.00090790535083e-05 | 7.26255036727867e-05 | 7.83566203450096e-05 | 7.19057962472423e-05 |
| pos\_803 | Epinephrine | 6.86669452931909e-05 | 0.000122283585744826 | 6.93960335599908e-05 | 8.38148514981303e-05 | 0.000103591954901645 | 8.42669491310297e-05 | 9.49686015149925e-05 | 8.53576082272459e-05 | 9.99255357758371e-05 | 8.21367131628352e-05 | 9.22181725083663e-05 | 9.57626385238534e-05 |
| pos\_804 | Methyl 7-epi-12-hydroxyjasmonate glucoside | 8.45137350652132e-06 | 1.34383846834291e-05 | 5.2531728701498e-06 | 1.52721985727794e-05 | 8.78540391025812e-06 | 9.21031979091285e-06 | 1.10979235198924e-05 | 9.75441029980688e-06 | 5.05267523448091e-06 | 8.94838415302336e-06 | 9.21927249350821e-06 | 1.19379307584791e-05 |
| pos\_805 | Capric acid | 7.29352756533225e-06 | 9.20903054066515e-06 | 1.43875785419919e-05 | 5.21106475251105e-06 | 8.82119839495221e-06 | 9.72254893704205e-06 | 3.6443256660268e-06 | 8.15935030171057e-06 | 4.77071063455193e-06 | 1.7116682074388e-05 | 9.04488443008461e-06 | 1.22532162319028e-05 |
| pos\_806 | Norfloxacin | 5.44318300704795e-06 | 7.94883998825168e-06 | 8.23520139561596e-06 | 2.65830048823034e-06 | 1.19695178024044e-05 | 3.46063669690038e-06 | 2.40118799640582e-05 | 2.19302712184242e-05 | 4.55848847859621e-06 | 1.45979998649e-05 | 1.41267154417351e-05 | 6.47776556045351e-06 |
| pos\_807 | Aldosine | 1.97447598417514e-05 | 1.03847952918874e-05 | 1.14993824725489e-05 | 8.78073402808556e-06 | 3.98077271375439e-06 | 9.27302365462779e-06 | 1.44580766585693e-05 | 1.22467618507821e-05 | 3.71139585853179e-06 | 9.83197945074737e-06 | 1.00841828336362e-05 | 1.21713395973334e-05 |
| pos\_808 | Araliasaponin I | 3.79494730207912e-06 | 4.37167043206166e-06 | 3.25415502550225e-06 | 6.73807427346288e-06 | 7.72687574639407e-06 | 5.48721792675902e-06 | 1.16356599319975e-05 | 6.21551340963164e-06 | 4.37679684462625e-06 | 8.81561243800398e-06 | 4.02323879020341e-06 | 4.97870350831171e-06 |
| pos\_809 | Hexadecanedioic acid | 2.62242161238662e-05 | 3.1378480293161e-05 | 3.03824838983682e-05 | 2.50221920465808e-05 | 2.37760213158706e-05 | 2.77403092613314e-05 | 2.92259611970587e-05 | 2.76615545608348e-05 | 2.51044519898211e-05 | 3.50720335803352e-05 | 2.7743768221175e-05 | 3.17164425005923e-05 |
| pos\_81 | Ectocarpen | 0.000116015989928382 | 0.000130553276458248 | 0.000116046833426209 | 0.000137799370756874 | 0.000110800459759268 | 0.000122806133995643 | 0.00011430229148844 | 0.000124828370058281 | 0.000145466596297361 | 0.00012873898167392 | 0.000133420510992441 | 0.000121542178173877 |
| pos\_810 | Saxitoxin | 6.09170863808321e-06 | 7.89113464894228e-07 | 3.99655005535057e-06 | 8.53641208033819e-06 | 3.34984670355161e-06 | 1.1296872291249e-05 | 6.94790817134106e-06 | 6.30742730852459e-06 | 3.70322490599167e-06 | 1.07978363387114e-05 | 1.27108641887196e-05 | 1.1064379275126e-05 |
| pos\_811 | Rubinic acid | 0.00049382102571755 | 0.000680308252499246 | 0.00085616487439204 | 0.000719826050533111 | 0.000566311412917396 | 0.000703648274158961 | 0.000811566529372944 | 0.00106899390856675 | 0.000653116355542902 | 0.000714479486041159 | 0.000875816118039618 | 0.00133020561312734 |
| pos\_812 | 8,12-Epoxy-4(15),7,11-eudesmatrien-1-one | 1.25057756901375e-05 | 8.96486965554965e-06 | 6.97721102588106e-05 | 0.000180668116823825 | 3.40066727918712e-05 | 5.75337371894125e-05 | 2.83001611340426e-05 | 0.000124437924969558 | 3.10805561841111e-06 | 1.08243995206599e-05 | 7.85724492071432e-05 | 0.000354532097888784 |
| pos\_813 | Phytosphingosine | 9.72596848981928e-06 | 1.13037571231188e-05 | 9.10520836111222e-06 | 8.83613406000483e-06 | 7.09041326264312e-06 | 1.14171847861244e-05 | 1.09383550251675e-05 | 1.26596486636994e-05 | 9.40904221610306e-06 | 8.22420125613484e-06 | 9.5994514088847e-06 | 1.12648724938104e-05 |
| pos\_814 | Hemorphin-4 | 1.0738373343933e-05 | 1.47154777823085e-05 | 2.89835448573403e-06 | 6.07969355097099e-06 | 8.9619060477228e-06 | 8.46433799639524e-06 | 6.23045474675728e-06 | 6.7633322753261e-06 | 1.00125657799167e-05 | 8.5601215628813e-06 | 6.93023810147299e-06 | 6.52758166864501e-06 |
| pos\_815 | 1-(3-Methylbutanoyl)-6-apiosylglucose | 6.59303283485476e-06 | 8.74426190816302e-06 | 2.35958586088883e-06 | 5.94801956215331e-06 | 9.19695706212216e-06 | 3.90033812835989e-06 | 5.92965969142726e-06 | 4.23156806676959e-06 | 5.75032290151693e-06 | 8.26585370972369e-06 | 6.51698380012221e-06 | 6.00938834805273e-06 |
| pos\_816 | Imidazoleacetic acid ribotide | 1.44114819032603e-05 | 6.10711937539558e-06 | 4.46190293266386e-06 | 5.49185118671047e-06 | 8.83980367078559e-06 | 6.92389937045398e-06 | 8.45360375498761e-06 | 6.69499963705981e-06 | 6.30497975284693e-06 | 5.55735253438049e-06 | 6.28168330160551e-06 | 6.37618195740471e-06 |
| pos\_817 | Methyl (3b,11x)-3-Hydroxy-8-oxo-6-eremophilen-12-oate | 0.00224539704641052 | 0.00283318667504751 | 0.00266580603353988 | 0.00233851133997847 | 0.00207180163089456 | 0.00245943495603129 | 0.0021885896541515 | 0.00235980084458219 | 0.00271750870787656 | 0.002440519183431 | 0.00229352318240284 | 0.00224828027145024 |
| pos\_818 | Avocadene 1-acetate | 1.54324823277126e-05 | 2.23190875477985e-05 | 1.78498786555006e-05 | 1.83153903418727e-05 | 2.6293813054112e-05 | 2.19862742659369e-05 | 2.51285978256046e-05 | 2.55559780826444e-05 | 2.46724068803385e-05 | 2.19012754888708e-05 | 2.49916228113165e-05 | 2.77120968425034e-05 |
| pos\_819 | Endomorphin-2 | 5.74960849988007e-06 | 5.35911969517964e-07 | 6.5890584275701e-06 | 1.78858764188996e-07 | 2.66056552948666e-06 | 3.77287843313558e-06 | 6.09958751485501e-06 | 1.57341497728672e-06 | 0 | 1.56281929270167e-05 | 5.47983343577749e-06 | 2.47749940256845e-06 |
| pos\_82 | 16-fluoro-7Z-hexadecenoic acid | 2.07666765183851e-05 | 2.46813112311243e-05 | 1.93717908158377e-05 | 2.15434672497794e-05 | 2.01526193498974e-05 | 2.02033000732503e-05 | 1.62853617774829e-05 | 1.99465874788915e-05 | 1.55849627349054e-05 | 3.03740323239457e-05 | 2.0366595027642e-05 | 2.40423512091953e-05 |
| pos\_822 | Sulpiride | 5.61053192709378e-06 | 1.44662088616248e-06 | 0 | 0 | 0 | 0 | 0 | 9.51402138343371e-07 | 0 | 0 | 0 | 1.46020547612873e-06 |
| pos\_823 | Dioxinoacrimarine A | 8.68488508785975e-07 | 0 | 1.86866272943136e-06 | 2.73355986713711e-06 | 3.84773738849446e-06 | 0 | 2.61545230513456e-07 | 0 | 5.040883677526e-07 | 0 | 0 | 1.85336330312343e-06 |
| pos\_824 | Epoxyganoderiol A | 0.000529768615929712 | 0.000517602294842494 | 0.000620234482820004 | 0.000929878062773674 | 0.000500502432357358 | 0.00068756034905288 | 0.000831983650175596 | 0.00495569354573001 | 0.000893946010573125 | 0.000644093479156946 | 0.00341182790360078 | 0.011208410197216 |
| pos\_825 | 1-(6Z,9Z,12Z-octadecatrienoyl)-glycero-3-phosphate | 7.73077863576895e-05 | 9.6007879325217e-05 | 4.30351681036817e-05 | 9.47196954435109e-05 | 7.4332703514344e-05 | 0.000102209712769746 | 9.17854188407516e-05 | 7.07133253591981e-05 | 7.36219833924926e-05 | 7.70046124310854e-05 | 8.06406053141084e-05 | 7.61544015540135e-05 |
| pos\_826 | Hydroxy-lacosamide | 0.00731076733652429 | 0.00782210116328703 | 0.0087297598156241 | 0.00770722822840196 | 0.00561658300033379 | 0.00734825552112787 | 0.00550800215048666 | 0.00695706238772638 | 0.00649385054239722 | 0.00865811576051699 | 0.00749494615215313 | 0.00923507654908089 |
| pos\_827 | DG(14:1(9Z)/22:6(4Z,7Z,10Z,13Z,16Z,19Z)/0:0) | 0.00119832615362031 | 0.00176012218247979 | 0.00107432637786879 | 0.00331715500158526 | 0.000768434184750632 | 0.00164680234345206 | 0.0011217340863159 | 0.00100165521492793 | 0.000584069833573253 | 0.00132602521194529 | 0.000990328509691827 | 0.000866597456502282 |
| pos\_828 | Cinnamyl cinnamate | 2.00270189605618e-05 | 8.63973869705012e-05 | 2.04034903860035e-05 | 2.06976573890697e-05 | 4.62987047656456e-05 | 3.4357096049956e-05 | 1.69724254046796e-05 | 1.59645488733919e-05 | 2.49257456749605e-05 | 2.66144262377432e-05 | 2.13477938250138e-05 | 1.63277036553567e-05 |
| pos\_829 | Blasticidin S | 7.57532728812839e-05 | 9.98325291226322e-05 | 2.59034121557481e-05 | 0.000138942658303505 | 1.93210839543061e-05 | 6.1715964130205e-05 | 7.46094745247814e-05 | 6.13227728506629e-05 | 2.24781600950699e-05 | 0.000244658556921608 | 7.92568043606821e-05 | 3.85774991191363e-05 |
| pos\_83 | 8Z,11Z,14Z-heptadecatrienoic acid | 0.000109180432297386 | 6.48127336591441e-05 | 0.000119142848218602 | 9.97165066872565e-05 | 6.08211266368244e-05 | 8.84420071811391e-05 | 0.000136803277041327 | 0.000133553408260513 | 0.000119033449686916 | 9.18186445404411e-05 | 0.000125166949184939 | 0.000150647820531752 |
| pos\_830 | Isoamyl 2-furonpropionate | 8.99211543070126e-06 | 1.43024571858294e-05 | 7.40512906237002e-06 | 8.60190776074709e-06 | 1.68283797200518e-05 | 1.12097237767689e-05 | 1.87334246395453e-05 | 1.42107780131476e-05 | 1.00640034526114e-05 | 1.03749466410394e-05 | 1.31452402953562e-05 | 8.98793572186929e-06 |
| pos\_831 | Hydralazine acetone hydrazone | 2.98766807025069e-06 | 6.89984921568349e-06 | 4.37342674968698e-05 | 4.12678696716367e-06 | 5.67650831327344e-06 | 1.27356278076834e-05 | 6.90947636335895e-06 | 9.52193608305621e-06 | 5.92298796637916e-06 | 7.66475370982503e-06 | 9.03222089664721e-06 | 1.62552726838807e-05 |
| pos\_832 | Ginsenoside Rh5 | 0.000152466373969504 | 0.000195731153382122 | 0.000267487542675061 | 0.000188349784230865 | 0.000129421147398168 | 0.000148713655660928 | 0.000136939623605528 | 0.000172625683617628 | 0.000173798066068194 | 0.000128518652488131 | 0.00016915934895999 | 0.00016885383092206 |
| pos\_833 | 4-(2,6,6-Trimethyl-1-cyclohexenyl)-2-butanol | 9.77307287911229e-07 | 2.01099790692981e-06 | 1.6341362291915e-06 | 1.9216876051551e-06 | 4.3394257616769e-07 | 8.84338134761504e-07 | 1.28329758459991e-06 | 1.66307956526403e-06 | 2.28428158453305e-06 | 6.21230073728798e-07 | 1.11446643886681e-06 | 1.60516067066913e-06 |
| pos\_834 | Prehumulinic acid | 1.78613618425816e-05 | 1.31676139618269e-05 | 1.32897277886038e-05 | 1.69229280312198e-05 | 2.32439560863918e-05 | 2.75616632073365e-05 | 4.74613702712651e-05 | 1.88130843990133e-05 | 8.15486901395277e-06 | 3.43473648453915e-05 | 2.72885947553268e-05 | 1.9914915619817e-05 |
| pos\_835 | 3-Dehydroxycarnitine | 6.05027657711424e-06 | 7.23735670370723e-06 | 7.7703683109418e-06 | 9.3162978191674e-06 | 9.80741076492771e-06 | 8.06393935267552e-06 | 7.78560625401135e-06 | 7.12427881921785e-06 | 9.61243995795789e-06 | 8.06398504752811e-06 | 7.77750124913579e-06 | 9.78161335511707e-06 |
| pos\_836 | 24,25-Diacetylvulgaroside | 7.1516728163128e-05 | 7.95073117712059e-06 | 7.39657347857564e-06 | 4.36404344007323e-06 | 3.82781325001399e-05 | 2.84608095244135e-05 | 2.91847142419053e-05 | 1.3424329537045e-05 | 1.06474572163224e-05 | 1.59872511172483e-05 | 1.94788143885522e-05 | 1.7222168443421e-05 |
| pos\_837 | Gibberellin A105 | 0.000685355832246262 | 0.000612263112456943 | 0.000195608683929505 | 0.000378686743209057 | 0.000170236561432734 | 0.000378883350886824 | 0.000190234044319468 | 0.000184008997476186 | 0.000277743581291956 | 0.000914218880778395 | 0.000350884142389945 | 0.000218347835682219 |
| pos\_838 | 5-Amino-2,3-dihydro-6-(3-hydroxy-4-methoxy-1-oxobutyl)-2,2-dimethyl-4H-1-benzopyran-4-one | 2.01346222901608e-05 | 2.00562077175379e-05 | 1.88873879093177e-05 | 7.33255580728523e-05 | 1.25350766531538e-05 | 2.32099518511617e-05 | 1.78799972781263e-05 | 2.35116379285634e-05 | 1.17777198141047e-05 | 1.92005115103003e-05 | 2.08173670568027e-05 | 3.64530246886589e-05 |
| pos\_839 | M-Secociguatoxin 4A | 5.63336074410812e-05 | 3.42999524705369e-05 | 5.16910455869195e-05 | 5.54203272912755e-05 | 3.24214212103272e-05 | 4.93448957008332e-05 | 3.29903084697147e-05 | 4.76898981052685e-05 | 4.21385156833923e-05 | 6.57572274503612e-05 | 5.25026737454057e-05 | 9.12850287626323e-05 |
| pos\_84 | GATIFLOXACIN | 0.000182017368520405 | 0.000115119789983944 | 2.06267576056499e-05 | 7.57378118982191e-05 | 6.38969723660283e-05 | 8.39768884581726e-05 | 1.02688430477963e-05 | 7.16620742177369e-06 | 2.73129974765128e-05 | 6.54968220420819e-05 | 2.1694430647646e-05 | 1.33932084155007e-05 |
| pos\_840 | DG(20:3(5Z,8Z,11Z)/22:6(4Z,7Z,10Z,13Z,16Z,19Z)/0:0) | 0.000371854884686684 | 0.000521113807433651 | 0.000470635162790533 | 0.000418345758336657 | 0.000646535397121394 | 0.000390644369507376 | 0.000986370561627485 | 0.00116705103356387 | 0.000459049920378506 | 0.000963338498961197 | 0.000505268756219604 | 0.00112196069131797 |
| pos\_841 | (3S,3'R,4xi)-beta,beta-Carotene-3,3',4-triol | 0.000311503515325723 | 0.000296807476175022 | 0.000199411524107886 | 0.000286541690684461 | 0.000223292906655179 | 0.000256722458369563 | 0.000310391620608564 | 0.000262216809676373 | 0.000262834451533554 | 0.000269274779458473 | 0.000268814288457315 | 0.000229071664319523 |
| pos\_843 | Camelledionol | 4.07726983695842e-05 | 7.31662361634177e-05 | 9.62243905104313e-05 | 0.000101057128018279 | 7.11202055374832e-05 | 7.18130824723524e-05 | 8.7615277575817e-05 | 0.000100167682210057 | 5.23490089636481e-05 | 5.19823008734447e-05 | 8.11399497416223e-05 | 0.000309449217144149 |
| pos\_844 | 3,4-Methylenesebacic acid | 4.91063433360505e-05 | 6.72482715980431e-05 | 3.4074509994752e-05 | 4.49550715563836e-05 | 5.09681813152045e-05 | 4.59536047069585e-05 | 3.90414062550474e-05 | 3.67616189945171e-05 | 5.29767564107013e-05 | 4.76228070407521e-05 | 4.35320160477361e-05 | 3.89211019210976e-05 |
| pos\_845 | Ribalinium | 6.92035923385439e-06 | 1.02168406942264e-05 | 1.56068064920766e-06 | 3.77039849417552e-06 | 5.08825311295045e-06 | 3.68737436744857e-06 | 2.99109736921552e-06 | 3.19288300213116e-06 | 2.859508617406e-06 | 5.06111078699643e-06 | 3.96337513868406e-06 | 3.15992340497357e-06 |
| pos\_846 | 10-Epijunenol | 1.62393851788136e-06 | 2.41799491118818e-06 | 1.00475898515724e-06 | 3.63882047541971e-06 | 5.60527286678188e-06 | 2.72990782656538e-06 | 2.07270135859439e-06 | 1.42375253516715e-06 | 2.50035238592319e-06 | 2.26043602762008e-06 | 2.56336616241357e-06 | 2.42300914876988e-06 |
| pos\_848 | Procaine | 0.00013874988057756 | 0.000138182257496163 | 0.00021851280929365 | 0.000106596057879268 | 0.000131260382016368 | 0.000128426185928342 | 8.81575379608079e-05 | 0.00013549232694689 | 9.44796493354947e-05 | 0.000246875310960605 | 0.0001400354179788 | 0.000199321866632618 |
| pos\_849 | 24-Oxo-1alpha,25-dihydroxyvitamin D3 | 0.00564252252358959 | 0.0059265042038135 | 0.00611577358430982 | 0.00680019615160024 | 0.00563322002512106 | 0.00539566084616787 | 0.00934771530679631 | 0.00766220717552571 | 0.00683509475704201 | 0.00737456378030664 | 0.00736855631143196 | 0.00760723898602265 |
| pos\_85 | Ipomeamaronol | 1.49551547459525e-05 | 1.33826078311573e-05 | 3.55450942183456e-05 | 1.17647570186936e-05 | 1.37375608104132e-05 | 1.72834533144394e-05 | 1.56304778793458e-05 | 1.69148910926806e-05 | 1.03315910407229e-05 | 2.67886052581449e-05 | 1.78558506808693e-05 | 2.29840734873878e-05 |
| pos\_850 | 5-Ethyl-4-methyl-2-octylthiazole | 5.58459174916774e-06 | 5.40307420237309e-06 | 6.22100327820671e-06 | 5.67694466812683e-06 | 6.04924156105914e-06 | 5.11090151518337e-06 | 5.28336807678119e-06 | 5.90210877913338e-06 | 6.33019738134882e-06 | 5.75344337521898e-06 | 5.78004645973047e-06 | 6.74657975955342e-06 |
| pos\_851 | Panaxatriol | 0.000128425643357036 | 0.000145314103995473 | 0.000114656723530673 | 0.000129042287945757 | 9.64679331959859e-05 | 0.000142371497111627 | 0.000150205797183894 | 0.00016271321114096 | 0.00012006522414776 | 0.000117449052725242 | 0.000128344628039822 | 0.00013099279712651 |
| pos\_853 | Repaglinide | 1.54769820480489e-05 | 2.20625459590828e-05 | 6.03462606220167e-05 | 3.50486151222543e-05 | 3.34355583104327e-05 | 3.06901377709519e-05 | 6.70006766340986e-06 | 9.40131876113152e-06 | 4.76413917813484e-05 | 1.51966997200441e-05 | 1.89078868960395e-05 | 1.55785117647766e-05 |
| pos\_854 | 7-Hydroxy-2,5-dimethyl-4H-1-benzopyran-4-one | 3.21155348395974e-06 | 3.73486335886597e-06 | 1.04997037696923e-05 | 4.85223384475221e-06 | 3.71414437693947e-06 | 5.59484065116475e-06 | 8.59928528778344e-06 | 7.12087603234659e-06 | 3.36124025158839e-06 | 4.13726648778644e-06 | 6.12780775486902e-06 | 7.20588326192703e-06 |
| pos\_855 | Pyranomammea C | 1.89246978205972e-05 | 9.61258860811048e-06 | 3.30252392225099e-06 | 0 | 1.5362257946496e-05 | 1.12169501621349e-05 | 1.47559834415684e-06 | 6.82581618172501e-06 | 6.02623380330954e-06 | 7.81625807302333e-06 | 4.12536575718543e-06 | 3.61365250389133e-06 |
| pos\_856 | N-Ethyl trans-2-cis-6-nonadienamide | 6.13675035195553e-05 | 7.73974044007027e-05 | 7.1507296358416e-05 | 6.43179885921933e-05 | 5.33136814298419e-05 | 6.29535295909077e-05 | 5.79046542278442e-05 | 6.53469028513875e-05 | 7.07934189587622e-05 | 5.92442924441694e-05 | 6.41378914281802e-05 | 5.73689802721005e-05 |
| pos\_857 | Small bacteriocin | 0.000132900983900758 | 0.000247814243498378 | 0.000160647144943754 | 0.000157387741598969 | 0.000139429843642771 | 0.000166239138573402 | 0.000120580682967 | 0.000112401364239793 | 0.000205566893894757 | 0.000171597994162706 | 0.000151124510018935 | 0.000111310765532387 |
| pos\_858 | 12-Hydroxynevirapine glucuronide | 3.0999153402892e-05 | 2.07396230819105e-05 | 3.83025675699248e-06 | 2.15901924996577e-05 | 2.50105750543045e-05 | 1.6099620938435e-05 | 8.56795831933448e-06 | 7.030734169527e-06 | 4.78159293049833e-06 | 2.77717259027639e-05 | 1.02070269765906e-05 | 5.76065773937593e-06 |
| pos\_86 | (11Z)-8,18-propano-retinal | 7.89031442268668e-05 | 0.000139330377561209 | 8.4177943523702e-05 | 0.000121994870642122 | 7.85449987846475e-05 | 7.20323628764274e-05 | 0.000242841018298443 | 0.000288786148704657 | 9.89842674406451e-05 | 9.02075030545051e-05 | 8.58102082393467e-05 | 7.06499344503125e-05 |
| pos\_860 | Isopropyl beta-D-glucoside | 2.53727396835157e-05 | 3.49403132110808e-05 | 1.92552750586181e-05 | 2.76126749704415e-05 | 2.95219427271976e-05 | 2.72334871287928e-05 | 2.54824309796899e-05 | 2.36129936250723e-05 | 2.84928870920281e-05 | 2.23127625056859e-05 | 2.63451868345648e-05 | 2.47989442870049e-05 |
| pos\_862 | L-Lysine | 1.40882577149982e-05 | 1.49795215851795e-05 | 1.94069838080405e-05 | 2.22285504774361e-05 | 2.20343378994176e-05 | 1.88291333222404e-05 | 1.82683196969172e-05 | 1.84451493591937e-05 | 2.08501172736495e-05 | 1.8788604963025e-05 | 1.9302653556862e-05 | 2.11743321396411e-05 |
| pos\_863 | trans-1,2-Dihydrobenzene-1,2-diol | 2.69938244051565e-05 | 2.09374064934565e-05 | 1.97884188544345e-05 | 1.64064452286766e-05 | 1.92062212979638e-05 | 1.98199353288902e-05 | 1.98957830213721e-05 | 1.94451948480445e-05 | 2.00310074076795e-05 | 1.94325601625973e-05 | 1.97562348746551e-05 | 2.07156313784031e-05 |
| pos\_864 | 2,4,4-Trimethylcyclopentanone | 1.05707914891659e-05 | 1.48356182392103e-05 | 1.38207350642739e-05 | 1.28586055342034e-05 | 8.97094997628571e-06 | 1.12701258021319e-05 | 1.02264623184753e-05 | 1.25024009572811e-05 | 1.2840136249351e-05 | 1.04713014237026e-05 | 1.19004803864125e-05 | 1.16304833828767e-05 |
| pos\_866 | N-Oleoylethanolamine | 0.000102814196232674 | 0.000130956325831409 | 0.000118175052445121 | 9.55386692036802e-05 | 7.13415733671289e-05 | 6.74589033685474e-05 | 6.22681335639282e-05 | 6.99830976775714e-05 | 8.91995288084702e-05 | 5.58449205575878e-05 | 6.31495034349558e-05 | 5.9759937705342e-05 |
| pos\_867 | 25-Hydroxyvitamin D3-26,23-lactone | 0.00106570457600249 | 0.00117365352246723 | 0.00115226450881007 | 0.0015936713459501 | 0.000829177612621144 | 0.00126482594670258 | 0.000983788672029224 | 0.00108749389405202 | 0.00114426784814655 | 0.00116963805868619 | 0.00112187239084317 | 0.00100832005913653 |
| pos\_868 | N1,N8-Diacetylspermidine | 4.54641273308152e-06 | 8.11679217077887e-06 | 1.41926547787844e-05 | 1.07203578708224e-05 | 3.29721873212477e-06 | 5.61485489835012e-06 | 9.18566942086098e-06 | 9.89654959969116e-06 | 5.32405440639646e-06 | 4.62208244941999e-06 | 2.93655335439077e-06 | 5.59761576584408e-06 |
| pos\_869 | Benzene | 1.98367360274206e-06 | 1.64546360906378e-06 | 9.73800651976399e-07 | 1.51701704497377e-06 | 2.76609279211669e-06 | 1.2531817715449e-06 | 2.04663011915854e-06 | 1.45399286258567e-06 | 1.25217326414268e-06 | 2.07789951701449e-06 | 1.41563923261678e-06 | 2.29533249131014e-06 |
| pos\_87 | 9,12,15-Octadecatrien-1-ol | 0.00111468709851658 | 0.00170835684453371 | 0.00151975428911609 | 0.00181032318622998 | 0.00133626274963028 | 0.0012664149324104 | 0.001170791466644 | 0.00139106787418216 | 0.00151000914522348 | 0.00133524204447775 | 0.00148608876216282 | 0.00147582995805103 |
| pos\_870 | 3b,12a-Dihydroxy-5a-cholanoic acid | 0.000303474637073442 | 0.0002812609959848 | 0.000541591638818769 | 0.000664638157525816 | 0.000714540534192549 | 0.000587152470221884 | 0.00045514249248963 | 0.000419857473389122 | 0.000658351895888961 | 0.000525858376663057 | 0.000524958390535918 | 0.00053476302299252 |
| pos\_871 | Chenodeoxycholic acid glycine conjugate | 8.12391082842294e-05 | 0.000113490022716904 | 0.000111215757364608 | 0.000108914345655066 | 0.000422600920915443 | 0.000160568251440147 | 0.000115753552161459 | 0.000100081709876676 | 8.35525151085616e-05 | 0.000109114110535522 | 9.68464942520515e-05 | 8.68420425455266e-05 |
| pos\_872 | Canavalioside | 0.000170793242907397 | 0.00028649948924022 | 0.000160181256371338 | 0.000191691939202173 | 0.000210572956975173 | 0.00018845421385442 | 0.000336807270111473 | 0.00029028544943565 | 0.000170605428714171 | 0.000164247027728803 | 0.000261864692878195 | 0.000225074116229425 |
| pos\_873 | Austalide L | 0.00133424896272041 | 0.000654339019747734 | 0.000604794012830411 | 0.000535902407113102 | 0.000910051555844573 | 0.000912869267486733 | 0.00100893124276981 | 0.000820941021289473 | 0.000749399998640229 | 0.000737282644397119 | 0.000820341823507832 | 0.000843034435811174 |
| pos\_874 | 4-OH-Retinal | 8.07579681941971e-05 | 9.90917193012254e-05 | 7.0848952653108e-05 | 7.12888368464328e-05 | 5.71553268493321e-05 | 6.8972408853871e-05 | 6.40552337136333e-05 | 6.77157991708165e-05 | 6.63740766205936e-05 | 6.88788878116149e-05 | 7.17188665838601e-05 | 6.2433547632778e-05 |
| pos\_875 | Isoleucyl-Histidine | 0.000517826269632518 | 0.000546697206353365 | 0.000447047430484389 | 0.000468166481649887 | 0.000428941490632378 | 0.000464966492034116 | 0.000415531458819848 | 0.000419672104601328 | 0.00046972263399406 | 0.000472224643318092 | 0.000421120483418058 | 0.000395292546370162 |
| pos\_876 | 3-(5-Methyl-2-furyl)prop-2-enal | 4.62513995088181e-05 | 4.74019654012192e-05 | 4.37774463543668e-05 | 3.86939687671052e-05 | 8.4324020975285e-05 | 3.80931084569911e-05 | 7.77112470816294e-05 | 6.72092548863841e-05 | 6.07132152706198e-05 | 6.85606512206845e-05 | 5.26164756799795e-05 | 7.86119380495662e-05 |
| pos\_877 | 3-Methylpyrrolo[1,2-a]pyrazine | 4.5486945655565e-06 | 4.65449173623191e-06 | 4.09781016788738e-05 | 3.76958333838482e-06 | 8.80132177122102e-06 | 1.00979567197489e-05 | 3.53116857407426e-06 | 8.53222955577115e-06 | 3.91906652749925e-06 | 8.91281309036805e-05 | 1.98288075768653e-05 | 1.9939247602889e-05 |
| pos\_878 | 7-Ketodeoxycholic acid | 4.85472395051718e-05 | 7.39198936418882e-05 | 9.8257260227954e-05 | 0.000111976328221411 | 8.5604891885949e-05 | 6.53139663230642e-05 | 7.23474348894014e-05 | 8.12357433379413e-05 | 9.24085719821786e-05 | 6.36532035643063e-05 | 5.22406809731814e-05 | 6.56784340693339e-05 |
| pos\_879 | Acetylcholine | 3.88510394359509e-06 | 5.35554327523994e-06 | 3.76125367219311e-06 | 3.64694530332491e-06 | 2.81976420075477e-06 | 4.0687364790989e-06 | 3.50220713693997e-06 | 4.6941530054887e-06 | 2.37765153766076e-06 | 3.44486479320032e-06 | 3.40633410265309e-06 | 4.06805823563371e-06 |
| pos\_88 | 3-Thiatetradecanoic Acid | 8.46871600893736e-05 | 0.000132618456858284 | 0.000172561494897252 | 0.000129054504206375 | 8.46379651717809e-05 | 0.000117467529354642 | 0.000139976352377379 | 0.000158361802044733 | 0.000142328621003814 | 0.000120165224249043 | 0.000156250316125939 | 0.000193033945700666 |
| pos\_880 | 3,4-Diethylthiophene | 6.2457215621961e-06 | 7.99579920510603e-06 | 6.98082930525316e-06 | 6.22258203920629e-06 | 4.70188759520062e-06 | 5.97092822357743e-06 | 5.67812314210909e-06 | 6.27609655838334e-06 | 6.89835788231978e-06 | 5.94510220135286e-06 | 6.65762305917594e-06 | 6.92437869576541e-06 |
| pos\_881 | 2,5-Dimethyl-3(2H)-furanone | 3.25433686310523e-06 | 4.06051605040294e-06 | 3.20328566453436e-06 | 4.36832193922064e-06 | 3.43104082431935e-06 | 3.73317101277534e-06 | 3.30487490637989e-06 | 3.47170803379351e-06 | 4.713458831318e-06 | 3.95600730329279e-06 | 3.82562325887083e-06 | 3.60617182296344e-06 |
| pos\_883 | Tsugarioside B | 0.000176134702404369 | 0.000274252925323451 | 0.000131758197568401 | 0.00121321424271998 | 0.000117895201362326 | 0.000440888482368858 | 0.000119413534043496 | 0.000129893510076141 | 0.000137521491478737 | 0.000127466868655133 | 0.000128729521552569 | 0.000133431278025669 |
| pos\_884 | Anandamide | 0.000326474388767202 | 0.000202052055228842 | 0.000252443236299998 | 0.000251892506974771 | 0.000312650541294357 | 0.000280304153730443 | 0.000216085196630485 | 0.000198416544073272 | 0.000266609997791069 | 0.000196795404491322 | 0.000228645282316864 | 0.000233877990698396 |
| pos\_885 | 4a-Methylzymosterol | 0.000724688350029705 | 0.00118969973182758 | 0.00171927354512984 | 0.00223240096236365 | 0.00158547935750136 | 0.00153332243533067 | 0.00181846962378515 | 0.00164815623046578 | 0.00136050222689507 | 0.000892529214336997 | 0.00147461695859747 | 0.00146889327981393 |
| pos\_886 | (4S,6S)-3,4,5,6-Tetrahydro-4-hydroxy-6-methyl-2H-pyran-2-one | 8.81355795805098e-06 | 1.36281574533461e-05 | 1.1997793133731e-05 | 1.10465765380876e-05 | 9.22423301312377e-06 | 1.04612078029444e-05 | 9.962223059837e-06 | 1.13072786626805e-05 | 1.18576364184809e-05 | 1.0704053550186e-05 | 1.10846375619153e-05 | 1.01054673668065e-05 |
| pos\_887 | Dolichyl b-D-glucosyl phosphate | 7.28569539864307e-05 | 0.000103602209562953 | 4.14728030292487e-05 | 6.06422895130832e-05 | 6.32944977845786e-05 | 6.64763941438499e-05 | 7.49624678691655e-05 | 6.06955500081043e-05 | 5.28206747709494e-05 | 7.15913745563732e-05 | 6.35394941784498e-05 | 5.87732019616653e-05 |
| pos\_889 | PI(16:0/20:0) | 0.00463348656644667 | 0.00263759961276178 | 0.00257969754618833 | 0.00231012360576558 | 0.00277064404127384 | 0.002979530660571 | 0.00312880856309926 | 0.00294479356636967 | 0.0028442041920204 | 0.00259033589639858 | 0.00277493551456171 | 0.00296662213434131 |
| pos\_89 | Strobopinin | 6.2946218783191e-05 | 0.000109082132939997 | 5.1077524119389e-05 | 6.89627082084009e-05 | 8.18210831717563e-05 | 7.02524437625048e-05 | 8.28472932861785e-05 | 6.54052078897055e-05 | 8.97583299161802e-05 | 7.67017144497737e-05 | 7.60954938965715e-05 | 6.28889720765437e-05 |
| pos\_890 | 2'-O-Methylphaseollinisoflavan | 2.32870013799855e-05 | 3.25051820951816e-05 | 7.52084660739774e-05 | 2.97802009032457e-05 | 2.8492893272465e-05 | 3.19757014538018e-05 | 3.91302281699145e-05 | 3.51190233911174e-05 | 2.72977812274901e-05 | 7.22879316376334e-05 | 0.000479620121305167 | 3.92323675445065e-05 |
| pos\_891 | Hericenone D | 0.000145514080746844 | 0.000135708464112621 | 0.00011009518128215 | 0.000277106420977813 | 0.000117273729542928 | 0.000152093865062533 | 0.000194464740727471 | 0.000181046828260833 | 0.000117506546445977 | 0.000265687547906051 | 0.000174170477292138 | 0.000193891180257932 |
| pos\_892 | Amphotericin B | 1.29420855438871e-05 | 1.22811008773924e-05 | 1.16335917020948e-05 | 2.77235450816482e-05 | 9.49089473770427e-06 | 1.28451730699858e-05 | 1.14131596996937e-05 | 2.58264651098501e-05 | 4.92848801347676e-05 | 1.33477341028667e-05 | 3.54666132438966e-05 | 2.47007275306854e-05 |
| pos\_893 | TetraHCA | 0.00549106106759159 | 0.00769168497222293 | 0.00603948751993308 | 0.0060071560615191 | 0.0104618259540638 | 0.00726209046102404 | 0.00773641624846038 | 0.00620142545339039 | 0.00865713426104739 | 0.0121288895527733 | 0.00767202920977123 | 0.00492132618197881 |
| pos\_894 | PA(P-16:0e/18:2(9Z,12Z)) | 8.87042432384244e-05 | 0.00012082297763198 | 0.000159545689491481 | 0.000125030327686337 | 8.75334182025512e-05 | 0.000124113865100561 | 9.94581784233183e-05 | 0.00012585101245769 | 0.000125485593765059 | 9.77157290579291e-05 | 0.000127059391242989 | 0.00010686518102616 |
| pos\_895 | Humulol | 3.45074607454387e-05 | 6.85223551784798e-05 | 5.79341280237328e-05 | 4.66720696438088e-05 | 3.01095060174205e-05 | 4.00977921515341e-05 | 3.99038881632921e-05 | 5.20525117434638e-05 | 5.65129421531631e-05 | 3.43290251328242e-05 | 4.04818115847894e-05 | 4.16887535252426e-05 |
| pos\_896 | 3-(3-Ethyloxiranyl)-2-propenal | 2.16433938777797e-05 | 2.39942080639701e-05 | 2.09892277375176e-05 | 2.33475519325568e-05 | 2.03736948725822e-05 | 2.18316382618107e-05 | 2.11678996943615e-05 | 2.21411001287255e-05 | 2.29577895002214e-05 | 2.27103579487035e-05 | 2.16991120172978e-05 | 2.11330471673366e-05 |
| pos\_897 | Lamivudine sulfoxide | 5.57242968625202e-06 | 1.20177005035507e-05 | 8.84284824812158e-06 | 9.28866554147432e-06 | 7.55076217498207e-06 | 8.24471137504102e-06 | 8.62872952670836e-06 | 7.31169110008103e-06 | 8.765071136851e-06 | 8.06658244512619e-06 | 8.1287488844586e-06 | 5.0562497010696e-06 |
| pos\_898 | 4-Methylphenyl dodecanoate | 1.29162700774798e-05 | 1.51691257550474e-05 | 9.39184538840748e-06 | 1.52698643764669e-05 | 1.07385288598764e-05 | 1.23284070100544e-05 | 1.40488551450883e-05 | 1.35859589841396e-05 | 1.16857665284159e-05 | 1.98554638570716e-05 | 1.35247205999268e-05 | 1.22821064158079e-05 |
| pos\_899 | Menthyl pyrrolidone carboxylate | 0.000120622863635643 | 0.000105742242606993 | 0.000105362069423325 | 8.78296930916505e-05 | 0.000108552693174143 | 0.000108747021864812 | 0.000100447448236507 | 0.000102358764877486 | 9.81379929181328e-05 | 0.000111740359662299 | 9.75737285810426e-05 | 0.00010632249958201 |
| pos\_9 | Retinoic acid | 8.2444852887116e-05 | 7.01737470608763e-05 | 7.63782769848594e-05 | 4.13174591669595e-05 | 5.21499691786979e-05 | 6.54555189268032e-05 | 3.81790636922574e-05 | 5.27560722622885e-05 | 3.5967062697896e-05 | 0.000166411195574705 | 6.32462136957048e-05 | 9.54675468163532e-05 |
| pos\_90 | Peltogynan | 2.8757101846307e-06 | 8.7495380616821e-07 | 0 | 9.26477886906991e-05 | 0 | 1.97790190783278e-06 | 1.92351000420009e-06 | 2.18778792156042e-07 | 1.43746922052324e-06 | 1.12083890988852e-06 | 0 | 5.29865358674646e-06 |
| pos\_900 | Methyl nomilinate 17-glucoside | 1.83316970266969e-05 | 8.67168602618264e-06 | 3.11793637681295e-06 | 5.11444096790915e-06 | 1.87583679589301e-06 | 2.54182754893925e-06 | 2.36716424524634e-06 | 2.7879645787041e-06 | 4.41268795474201e-06 | 2.33598006453601e-06 | 3.09242861240444e-06 | 3.84182220973752e-06 |
| pos\_901 | Furanofukinin | 6.6561021944933e-05 | 5.76072793400567e-05 | 1.69559709286618e-05 | 2.79011828000862e-05 | 9.18151870039986e-05 | 4.68475423089396e-05 | 0.000139960825897567 | 8.02484125779131e-05 | 3.75467812778316e-05 | 3.56181024154409e-05 | 6.51805014608139e-05 | 2.36387121900153e-05 |
| pos\_902 | Aginoside progenin | 6.36301967899158e-06 | 1.19186440167424e-05 | 5.339340291111e-06 | 5.2414682257e-06 | 6.90630741225923e-06 | 5.48600291381198e-06 | 8.32036765127379e-06 | 7.27452993625815e-06 | 9.1140690576964e-06 | 6.9537301313554e-06 | 5.62755688186786e-06 | 4.64335425738675e-06 |
| pos\_903 | Lysinoalanine | 0.000101103581754307 | 0.000263552233985968 | 2.69483253645948e-05 | 3.23494719823862e-05 | 0.000177593307862401 | 0.000103931708345029 | 2.98776031144011e-05 | 3.23069751234474e-05 | 3.54092993817523e-05 | 3.4304896112625e-05 | 3.36105119956271e-05 | 4.62315442396958e-05 |
| pos\_904 | Carpaine | 0.000765875719930546 | 0.000904028931063944 | 0.00107781562531022 | 0.0010695968398845 | 0.000894773651297901 | 0.00108742381628677 | 0.000913365803430154 | 0.00100331368959255 | 0.00104312437587447 | 0.000855617733490801 | 0.00101405367806858 | 0.000894807240777894 |
| pos\_905 | Kanokoside D | 9.38066007886226e-05 | 6.13499288043576e-05 | 3.81837932551373e-05 | 1.88200530340049e-05 | 0.000156596516850164 | 7.63623812704565e-05 | 3.64614017799739e-05 | 4.33018150417571e-05 | 4.10548854664278e-05 | 5.32897830086274e-05 | 4.91762703329656e-05 | 6.83849485627638e-05 |
| pos\_906 | L-argininium(1+) | 1.65540497380371e-05 | 2.74209047985839e-05 | 2.03583013997669e-05 | 1.93648812754359e-05 | 1.48459483210952e-05 | 2.04068119138777e-05 | 1.57389055342805e-05 | 2.09648502195169e-05 | 1.94267091717256e-05 | 1.61278549671858e-05 | 2.02209979858412e-05 | 2.10749298864371e-05 |
| pos\_908 | 35S-Methylokadaic acid 7-hexadecanoate | 0.000122951238998922 | 3.71053896976696e-05 | 3.65150327956295e-05 | 3.31056862012655e-05 | 4.51198927959147e-05 | 4.77829878508764e-05 | 5.32386543113412e-05 | 4.3353712140447e-05 | 4.16500955965405e-05 | 4.05102190980289e-05 | 4.21683443462283e-05 | 4.43334257959915e-05 |
| pos\_909 | N,N-Didesmethyltramadol | 0.000353349631300685 | 0.000347829197003268 | 0.000332533976022751 | 0.000377524710128626 | 0.000264862038999728 | 0.000362080319958935 | 0.000389575764767854 | 0.000441462124941685 | 0.000435941447237555 | 0.000310715427103631 | 0.000422197941794553 | 0.000870773106148476 |
| pos\_91 | 4-Quinolinecarboxylic acid, 2,8-bis(trifluoromethyl)- | 3.54791959034441e-05 | 5.38681176333823e-05 | 1.9408526595414e-05 | 5.03556703891618e-05 | 3.50474334887379e-05 | 3.43169384609459e-05 | 2.80201762501222e-05 | 2.11059893024115e-05 | 7.64849259736815e-06 | 5.69800929342777e-05 | 2.76086947675546e-05 | 9.12891440989262e-06 |
| pos\_910 | 8,8-Diethoxy-2,6-dimethyl-2-octanol | 3.32796033588517e-05 | 3.6100069324349e-05 | 4.91347732326888e-05 | 3.48671950185528e-05 | 4.2686932497578e-05 | 3.97200360665068e-05 | 3.89656418357747e-05 | 3.96272951813721e-05 | 2.66255875119515e-05 | 5.36142335643694e-05 | 4.248580066701e-05 | 5.20578349059701e-05 |
| pos\_911 | 4-Pyridoxic acid | 4.11564949986041e-06 | 3.08172345481163e-06 | 1.83563977916861e-06 | 1.9441882957479e-06 | 7.76830181616356e-06 | 3.15720959140439e-06 | 2.03178363058514e-06 | 2.86223078960514e-06 | 6.45442495718927e-06 | 1.15080523510687e-06 | 4.01540197809027e-06 | 4.27250351055664e-06 |
| pos\_912 | 5''-(4-Hydroxy-(E)-cinnamoyl) alpha-L-arabinofuranosyl-(1->3)-beta-D-xylopyranosyl-(1->4)-D-xylopyranoside | 1.51039614252908e-05 | 1.16651148779743e-06 | 1.11911039919465e-06 | 0 | 8.34811201967831e-06 | 6.97706543586291e-06 | 6.44479830475458e-06 | 2.79461944270079e-06 | 2.14554117715142e-06 | 2.35473981463475e-06 | 1.87215309444061e-06 | 3.79739658087711e-06 |
| pos\_913 | Spermine | 5.2822100627192e-06 | 5.86988594340794e-06 | 6.4040638905428e-06 | 8.53026757684722e-06 | 8.41592305051146e-06 | 6.79132914102594e-06 | 5.52083586374025e-06 | 6.04607344980499e-06 | 8.19783410848277e-06 | 7.04141109340551e-06 | 7.0604835780012e-06 | 6.53696665550671e-06 |
| pos\_914 | TG(18:3(9Z,12Z,15Z)/20:4(5Z,8Z,11Z,14Z)/22:6(4Z,7Z,10Z,13Z,16Z,19Z))[iso6] | 3.19158835730115e-05 | 3.03292236461187e-05 | 4.83620586505823e-05 | 4.23911897649794e-05 | 3.88638131324079e-05 | 4.33757446811439e-05 | 6.52756054840604e-05 | 5.33201792203514e-05 | 3.56325211832381e-05 | 4.29880010000352e-05 | 5.48423343993755e-05 | 4.41836204322972e-05 |
| pos\_915 | Avocadienofuran | 1.70087669863476e-05 | 2.46513311485526e-05 | 1.7166872022818e-05 | 2.08574461338493e-05 | 1.28526213251158e-05 | 1.82043280060989e-05 | 2.45853630541638e-05 | 2.07927667699364e-05 | 1.37391363780716e-05 | 2.11192217638619e-05 | 1.86315314733869e-05 | 1.68851675947221e-05 |
| pos\_916 | Lysyl-Proline | 6.12366310812763e-05 | 7.13698984929063e-05 | 0.000117912415507811 | 3.92891298862505e-05 | 9.63774140900636e-05 | 6.94307313074564e-05 | 3.97453604245002e-05 | 6.65507454727626e-05 | 3.34680579785757e-05 | 0.000159036711902984 | 7.16260949677322e-05 | 0.000115041768223428 |
| pos\_917 | 1-O-beta-D-Glucopyranosyl-2,3-di-O-(8-hexadecenoyl)glycerol | 3.20244646242746e-05 | 1.20008778401793e-05 | 5.66764795165095e-06 | 6.31988543743682e-06 | 6.34610588411288e-05 | 1.77077534761519e-05 | 6.2140769572406e-05 | 5.29705269322758e-05 | 1.5794807782188e-05 | 9.31679031201463e-05 | 5.3836452265062e-05 | 3.98242997293542e-05 |
| pos\_918 | Soyasapogenol B 24-O-b-D-glucoside | 0.00057775245884409 | 0.000193863542758639 | 0.000158533120144795 | 0.000140829832266711 | 0.000322849981981537 | 0.00031489411913289 | 0.000280777535459518 | 0.000247279785443678 | 0.000210088856565372 | 0.000221708232706912 | 0.000250283274541604 | 0.000220186192520741 |
| pos\_919 | 4-Hydroxyandrostenedione glucuronide | 6.37564623010247e-05 | 3.1562196983594e-05 | 2.9539245066911e-05 | 2.63593768680042e-05 | 2.96132796068536e-05 | 3.54307638386484e-05 | 8.41396649236735e-06 | 7.50606668613485e-05 | 0.000121764923665996 | 1.38014231763451e-05 | 7.10107822770101e-05 | 0.00016998406621792 |
| pos\_92 | Adenine | 5.29028485328433e-05 | 7.11503937330297e-05 | 3.3354059267059e-05 | 5.79216517651703e-05 | 5.58854209040276e-05 | 3.94816282752042e-05 | 7.59100228633702e-05 | 5.16736517874884e-05 | 4.46776165216451e-05 | 9.84042192172715e-05 | 5.41491578575664e-05 | 3.99646573506956e-05 |
| pos\_920 | MG(0:0/20:1(11Z)/0:0) | 0.000180366235691653 | 0.000171919935053565 | 0.000188709684343773 | 0.000186058679679127 | 0.000178781495833667 | 0.000190105229905631 | 0.000104549951468493 | 0.00012666803967967 | 0.000233099821398592 | 0.000165042712957458 | 0.000159235201898316 | 0.000133347563298101 |
| pos\_921 | Methoxamine | 0.000258275125732371 | 0.00028470820890187 | 0.000247578407369933 | 0.000206776072172652 | 0.000430151028605833 | 0.000203190105710681 | 0.000321941392198233 | 0.00031515013815182 | 0.000266443475234122 | 0.000255348442389944 | 0.000255080275504462 | 0.000398821310089078 |
| pos\_922 | MG(0:0/14:1(9Z)/0:0) | 1.66318965526039e-05 | 2.49175945071981e-05 | 4.16487128185295e-05 | 2.37190227884637e-05 | 2.107866714094e-05 | 2.6955569170145e-05 | 1.47261940788669e-05 | 2.94667662169651e-05 | 3.22948524321828e-05 | 2.90312808703804e-05 | 2.68566160967221e-05 | 4.18453147335445e-05 |
| pos\_923 | T2 Triol | 3.13504170584751e-05 | 3.05529326511988e-05 | 4.81735677536413e-05 | 0.000118238829539518 | 3.34974605083131e-05 | 5.38470867953083e-05 | 2.8930638133193e-05 | 7.88411066913286e-05 | 2.41842656919113e-05 | 4.04911321464123e-05 | 6.18826483253132e-05 | 0.000157430345334831 |
| pos\_924 | Epiacoronene | 1.64121338508565e-05 | 2.2658781155292e-05 | 2.54051006166913e-05 | 2.7332891291706e-05 | 1.81938411203219e-05 | 2.11257504491426e-05 | 2.43192911942775e-05 | 2.46217150191836e-05 | 1.81219926389653e-05 | 2.53684090538052e-05 | 2.2967932258891e-05 | 2.72554835758122e-05 |
| pos\_925 | 13'-Hydroxy-alpha-tocotrienol | 0.00106062511297169 | 0.000436660226303493 | 0.000465144722811589 | 0.000390449703237286 | 0.000522994347748071 | 0.000534929424531801 | 0.000569140623819115 | 0.000514633771449375 | 0.000467981732295529 | 0.000508205597970794 | 0.000534427474394045 | 0.00054529572626116 |
| pos\_926 | Isoleucyl-Hydroxyproline | 3.63219777687382e-05 | 2.91513212270358e-05 | 5.59211899549252e-05 | 8.68657326391743e-06 | 1.75793242818532e-05 | 3.17467881613286e-05 | 2.31733965885969e-06 | 2.18202638871657e-05 | 3.75969148118978e-06 | 8.12962859077594e-05 | 2.75489876392285e-05 | 6.14677690435225e-05 |
| pos\_927 | 2-(4-Methyl-5-thiazolyl)ethyl formate | 2.65685639341734e-05 | 3.54686370050695e-05 | 3.36387947677917e-05 | 2.91542105265107e-05 | 2.44499296994263e-05 | 2.84674966101962e-05 | 2.67968417042368e-05 | 2.88659094192887e-05 | 3.26569660269613e-05 | 2.58680949736579e-05 | 2.9762359253733e-05 | 2.76624124915525e-05 |
| pos\_929 | Fagomine | 1.42948379185399e-06 | 8.53125423365086e-07 | 6.94018991535382e-07 | 2.78383673469728e-06 | 9.15805348603116e-07 | 1.80127826740193e-06 | 1.76497882604629e-06 | 3.55518435092359e-06 | 1.45531788179507e-06 | 4.65518944994938e-07 | 4.62522640172193e-07 | 1.6160762914982e-06 |
| pos\_93 | DIHYDROROBUSTIC ACID | 2.11523434174405e-05 | 2.16060611217389e-05 | 2.16518417774212e-05 | 1.84311636031511e-05 | 1.54923332611093e-05 | 1.84150834299879e-05 | 1.6229989490048e-05 | 1.93005579597304e-05 | 2.0915262295436e-05 | 1.76096488208031e-05 | 1.77736297059814e-05 | 1.79417396736628e-05 |
| pos\_931 | (S)-Abscisic acid | 8.45647469383701e-08 | 1.22049378123436e-06 | 2.03403329834328e-05 | 6.90421002429676e-05 | 2.30852872254567e-06 | 9.45909612540733e-06 | 1.17900456625734e-06 | 2.35651506864975e-05 | 7.85824330649991e-07 | 2.4796360401204e-06 | 1.30717009244611e-05 | 6.48345283225144e-05 |
| pos\_932 | Falcarindiol | 2.857010218125e-06 | 6.54753933707898e-06 | 6.98133570560923e-06 | 5.55587677309006e-06 | 1.34515271078941e-05 | 7.2291216146358e-06 | 6.32259070770715e-06 | 5.19104722517422e-06 | 8.11703435898129e-06 | 5.75464618784632e-06 | 6.49489999535287e-06 | 6.94543372304183e-06 |
| pos\_933 | Perindoprilat | 1.04790384356864e-05 | 7.91279872432221e-06 | 7.50564737906774e-07 | 5.91670252506796e-06 | 1.25636441078707e-06 | 1.44905794639597e-06 | 7.40212773154008e-07 | 9.40799824856652e-07 | 7.80820913919065e-07 | 1.11621361882206e-05 | 3.23753838591732e-06 | 3.40907218290434e-06 |
| pos\_934 | Cyanidin 3-O-[4-Hydroxy-E-cinnamoyl-(->6)-b-D-glucopyranosyl-(1->2)-b-D-glucopyranoside] 5-glucoside | 9.18011149359585e-08 | 7.80322732376474e-07 | 5.25929233061498e-06 | 0 | 8.02311615709173e-08 | 1.5141603008525e-06 | 0 | 5.35921590122039e-06 | 8.22656271814225e-06 | 4.39784538157966e-07 | 2.77350675478615e-06 | 2.27522175263118e-06 |
| pos\_935 | Vanylglycol | 2.47537626456761e-05 | 3.24624478968275e-05 | 1.87516849845551e-05 | 1.59666182067309e-05 | 2.01577552872362e-05 | 2.24149503246038e-05 | 1.53478909417707e-05 | 1.75121285753291e-05 | 4.14326830585369e-05 | 1.80740520178519e-05 | 2.35774054462644e-05 | 1.77539211194199e-05 |
| pos\_936 | Neohesperidin dihydrochalcone | 0.000207128580771505 | 2.73524553827355e-05 | 2.0925291741388e-05 | 1.29380634655431e-05 | 9.863944672972e-05 | 8.48819239070431e-05 | 7.51774209675511e-05 | 6.25764566399719e-05 | 3.24436186076612e-05 | 5.97539142036239e-05 | 6.31847350426022e-05 | 4.94330812196763e-05 |
| pos\_937 | (Z)-9-Cycloheptadecen-1-one | 5.63924683449776e-06 | 1.34890699939728e-05 | 2.40297437490546e-05 | 2.22347544423547e-05 | 3.51494997895559e-06 | 9.23794798611549e-06 | 1.86310104279385e-05 | 1.69282852827515e-05 | 1.11972860094461e-05 | 2.24231848147187e-06 | 2.82869507383903e-06 | 8.72110624537796e-06 |
| pos\_938 | 7-HETE | 3.05625482587164e-05 | 2.95259470584321e-05 | 3.68600167007192e-05 | 2.93051437351008e-05 | 3.95192527061858e-05 | 3.25278817304162e-05 | 3.59302945807232e-05 | 3.79280854253874e-05 | 1.82959713853264e-05 | 3.87737145761257e-05 | 3.47497046101324e-05 | 3.72322650724554e-05 |
| pos\_939 | [10]-Dehydrogingerdione | 0.000270150762200779 | 0.000243279697701336 | 0.000734176538647059 | 0.00018099081528482 | 0.000158338089629813 | 0.000294467267654485 | 0.000137236769220693 | 0.000319484635184727 | 0.000157060927582149 | 0.000485313944299983 | 0.000364116068553298 | 0.000625289375175836 |
| pos\_940 | 12alpha-Hydroxy-13,18-dehydroparain | 6.56776684593414e-06 | 1.22283368403448e-05 | 1.2890220396184e-05 | 8.11687325393759e-06 | 7.58076839244989e-06 | 8.53032696251064e-06 | 1.09259959483694e-05 | 1.08449949094875e-05 | 1.06729892699224e-05 | 8.05898902337817e-06 | 1.02725567586011e-05 | 9.65758343238095e-06 |
| pos\_941 | Hebevinoside VI | 2.51970980826834e-05 | 2.32505532946474e-05 | 5.16000439775807e-05 | 0.000115972423019904 | 3.42667775404601e-05 | 5.39801215527414e-05 | 2.32114088308932e-05 | 5.13391509624142e-05 | 2.24138091437882e-05 | 2.11576947045814e-05 | 4.37611754112211e-05 | 9.55715292338429e-05 |
| pos\_942 | Hydratopyrrhoxanthinol | 0.000313939687389906 | 0.000128562858458419 | 0.000115935978282943 | 0.000111397873168059 | 0.000201899704401772 | 0.000203612424979762 | 0.000193962447018897 | 0.000170916228455742 | 0.000146835419902528 | 0.000152042070930462 | 0.000166149378736242 | 0.000154184913929182 |
| pos\_943 | Geranyl phenylacetate | 5.71150979714232e-05 | 3.12812364494094e-05 | 3.13056962312571e-05 | 0.00021684433150059 | 1.16164659726143e-05 | 5.81715086613274e-05 | 0.000149698765580855 | 8.66900110502191e-05 | 2.14326877412344e-05 | 0.000179497036331085 | 9.18137314438307e-05 | 1.36685605624798e-05 |
| pos\_944 | MG(18:0/0:0/0:0) | 0.00053877210111778 | 0.000678313727690027 | 0.000626996380475382 | 0.000461812226769569 | 0.000431722473491646 | 0.000591278286473064 | 0.000531483832573401 | 0.0005774251875183 | 0.00063362741787435 | 0.000517633823627261 | 0.000542078720696545 | 0.000491243704631384 |
| pos\_945 | 15-Hexadecanolide | 2.04017418753684e-05 | 2.00066229910876e-05 | 2.13386385784511e-05 | 1.94715182230668e-05 | 1.46793864853736e-05 | 1.84854668395144e-05 | 1.58825076946561e-05 | 1.73541492791287e-05 | 1.66625962311626e-05 | 1.86660197548166e-05 | 1.89284626442661e-05 | 1.99458478072205e-05 |
| pos\_947 | (-)-(E)-1-(4-Hydroxyphenyl)-7-phenyl-6-hepten-3-ol | 5.28489561165567e-05 | 7.42960268595057e-05 | 7.14556457222744e-05 | 5.96285262237171e-05 | 3.65278723150367e-05 | 6.18791655987858e-05 | 3.19767220502533e-05 | 4.53170234752115e-05 | 6.36025054945168e-05 | 4.59815276482843e-05 | 5.380417484786e-05 | 5.43281856687777e-05 |
| pos\_948 | N-[2-(3,4-Dimethoxyphenyl)ethyl]-3,4-dimethoxycinnamic acid amide | 1.78798267975654e-05 | 1.77104700423362e-05 | 1.00173873586141e-05 | 2.50031340545901e-06 | 5.41644088962596e-06 | 3.40520820777479e-06 | 5.0636327297415e-06 | 4.13960039896172e-06 | 6.9001050004221e-06 | 3.41158649893786e-06 | 1.00836876655452e-06 | 2.12653430232397e-06 |
| pos\_949 | (3-Methyl-2-butenyl)-benzene | 1.40004728208629e-05 | 2.14853215277526e-05 | 2.27363479388946e-05 | 1.90115018382221e-05 | 1.9542647002028e-05 | 1.8208155023445e-05 | 1.99340173336568e-05 | 2.44794903235338e-05 | 1.82158433439914e-05 | 1.8626081470521e-05 | 2.09714264193839e-05 | 2.48912695828831e-05 |
| pos\_95 | (2S,6R,7S,8S)-2-allyl-7-((E)-but-1-en-3-yn-1-yl)-1-azaspiro[5.5]undecan-8-ol | 0.000164733505267883 | 5.52521246929405e-05 | 1.57258697035843e-05 | 0.000123294110612356 | 2.92622296013594e-07 | 6.68484183611925e-05 | 6.94595829841447e-06 | 1.47559988357517e-05 | 2.39461567643528e-05 | 0.000309738863360856 | 6.15596896602817e-05 | 2.59463258315259e-05 |
| pos\_950 | Isopropamide | 7.98680314376175e-06 | 7.79798694363487e-06 | 0 | 0 | 2.04473408948629e-06 | 2.01199926124227e-06 | 1.87714612208256e-06 | 3.23419097165871e-06 | 2.08149486710372e-06 | 0 | 1.39052043599217e-06 | 6.79855065857315e-07 |
| pos\_952 | Ximelagatran | 0.000107544411949338 | 5.56236567067834e-05 | 4.220505475316e-05 | 4.06685763262137e-05 | 4.73289007156352e-05 | 6.02061594565886e-05 | 1.98833054333362e-05 | 9.34535560360203e-05 | 0.000148257281121809 | 2.03191516812926e-05 | 8.84548076834038e-05 | 0.000213380325253679 |
| pos\_953 | Cysteinyl-Valine | 9.29014971814535e-06 | 9.05916823305443e-06 | 5.73267560650297e-06 | 6.00952262680797e-06 | 1.28765210198521e-05 | 6.96681910043404e-06 | 1.07765782445523e-05 | 8.02578992772353e-06 | 8.95653186560162e-06 | 7.53621213660111e-06 | 8.09780709110575e-06 | 1.19919650956692e-05 |
| pos\_954 | Benzphetamine | 3.78018387805029e-05 | 4.49041896432237e-05 | 4.44414678173575e-05 | 5.01673803451172e-05 | 4.12901880639364e-05 | 4.6336672275165e-05 | 5.01865984037481e-05 | 4.92298439089944e-05 | 4.56532272233892e-05 | 4.24000686128336e-05 | 4.90357666183538e-05 | 3.98401852366099e-05 |
| pos\_958 | Moringyne | 3.45849162902638e-06 | 3.40532599785383e-07 | 6.55811252819412e-06 | 3.27065309126751e-05 | 1.31556292487756e-06 | 2.00223301876939e-06 | 1.88685889055175e-06 | 1.12154633547466e-06 | 9.12574258785712e-07 | 5.25579062945504e-05 | 1.83108762976048e-06 | 7.2336062772094e-07 |
| pos\_959 | Macrophorin A | 0.000137896768884628 | 0.000187505719695172 | 0.000108457184707475 | 0.000186947625093077 | 0.000138907204152237 | 0.000142988256540613 | 0.000159787371597859 | 0.00014394246767713 | 0.000161344386199542 | 0.000166643292017073 | 0.000142282198245626 | 0.000135002393201733 |
| pos\_960 | Castamollissin | 1.73084433747964e-06 | 1.35215116942954e-06 | 1.07842813637247e-05 | 6.06789255826534e-06 | 4.23873131829984e-06 | 2.6787124262238e-06 | 3.33424040727367e-06 | 7.44305960999304e-06 | 3.77500314609713e-06 | 5.53380849195393e-06 | 2.30683216729057e-06 | 3.96638389729906e-06 |
| pos\_961 | 2-Octenedioic acid | 2.42301594677271e-06 | 3.14453359431656e-06 | 3.01129442875517e-06 | 3.20426702980981e-06 | 2.61151272178569e-06 | 2.46587920194764e-06 | 3.08296971494107e-06 | 3.27837810520113e-06 | 2.74559446467539e-06 | 2.91159105042013e-06 | 3.22180851041906e-06 | 3.689265697222e-06 |
| pos\_962 | L-Pyridosine | 1.37497305787657e-06 | 7.38576730759744e-06 | 9.28663080321125e-06 | 9.59277784997712e-07 | 1.36643108961613e-05 | 5.73247937583997e-06 | 1.06108851467168e-06 | 4.09713901654321e-06 | 9.39615393678798e-07 | 1.57386726148977e-05 | 6.95809731845748e-06 | 1.25299270369962e-05 |
| pos\_963 | Permetin A | 3.34582456441859e-06 | 1.76019251121238e-06 | 5.38043283148596e-07 | 8.88687809860171e-07 | 1.08806344507762e-06 | 1.01635341164324e-06 | 2.17342958792564e-05 | 1.14213360102459e-05 | 2.76889810024298e-06 | 2.27011202466147e-05 | 9.90608485694238e-06 | 0 |
| pos\_965 | 9,10,13-Trihydroxystearic acid | 7.92469961644955e-05 | 8.94180757685107e-05 | 9.17027545342534e-05 | 8.61924875606766e-05 | 7.1225327895973e-05 | 8.28072710118366e-05 | 7.2012681256095e-05 | 8.31755817951171e-05 | 8.45761050086941e-05 | 7.62993107185152e-05 | 8.12181165609921e-05 | 7.99500824816078e-05 |
| pos\_966 | (-)-trans-3,4-Dihydro-4,8-dihydroxy-3-methyl-1H-2-benzopyran-1-one | 3.12460389882989e-06 | 4.02501151092373e-06 | 3.48347610631358e-06 | 2.6145188279491e-06 | 1.74452186399228e-06 | 4.20083265606714e-06 | 4.26875537687016e-06 | 3.81996359296843e-06 | 2.58052444852175e-06 | 5.27631932431173e-06 | 2.39559897548787e-06 | 3.33386591598109e-06 |
| pos\_967 | 2,4,5,7alpha-Tetrahydro-1,4,4,7a-tetramethyl-1H-inden-2-ol | 7.63851967310908e-05 | 8.03511171568225e-05 | 6.24619485140068e-05 | 8.63253969924198e-05 | 7.77870018525161e-05 | 8.06092140466352e-05 | 7.23337891004689e-05 | 7.98931638653943e-05 | 8.52243688101911e-05 | 7.96050858106168e-05 | 8.37330366415676e-05 | 7.45231604112728e-05 |
| pos\_968 | 4-Coumaroylputrescine | 2.26171641269009e-05 | 2.51760244858064e-05 | 2.55295645799573e-05 | 3.44270211318229e-05 | 2.57113633700723e-05 | 2.28237504263242e-05 | 3.35911400001531e-05 | 2.70252133203652e-05 | 2.93151885277717e-05 | 2.91209946638595e-05 | 2.77454793888426e-05 | 2.84174763908178e-05 |
| pos\_969 | Isoleucyl-Serine | 1.35006194784331e-05 | 1.44279468879365e-05 | 2.15215047207214e-05 | 2.43112387687797e-05 | 1.74749334051441e-05 | 1.70079463098589e-05 | 1.79666074189726e-05 | 1.80005297546929e-05 | 1.44885706338156e-05 | 1.47897719376986e-05 | 1.57685570487875e-05 | 1.84388210660851e-05 |
| pos\_97 | 5-(1-oxopropan-2-yl)isolongifol-5-ene | 0.000285778486658287 | 0.000283642355066444 | 0.000572326924773284 | 0.000529487172661912 | 0.000646417243091881 | 0.000506892856063062 | 0.00045876136315447 | 0.000437728669493113 | 0.000585554214864233 | 0.00048582690865483 | 0.000501774496035283 | 0.000487170595650033 |
| pos\_970 | Hydroxydestruxin B | 0.000610723288716618 | 0.000382848791285527 | 0.000367569528766819 | 0.000329831158685696 | 0.000364775719290852 | 0.000408126272031427 | 0.000398815624746096 | 0.000394041730671781 | 0.000385236584229087 | 0.000351879540815996 | 0.000382712774990027 | 0.000382246563090133 |
| pos\_971 | (3b,5a,22E,24S)-Stigmasta-7,22,25-trien-3-ol 3-glucoside | 1.12955365921022e-05 | 7.14392244789698e-07 | 2.60790238155012e-05 | 1.53098596211252e-05 | 3.30691206714626e-05 | 1.82164326063688e-05 | 5.15958917839878e-06 | 4.72312635060431e-06 | 1.88524756115106e-05 | 1.80877884425898e-05 | 9.22527889384595e-06 | 8.89887411120735e-06 |
| pos\_972 | Torvoside D | 0.000603219554386427 | 0.00017910147437315 | 0.000184618144691838 | 0.000156679481591236 | 0.000347675431352025 | 0.000345615891136754 | 0.000307755408138405 | 0.00027221279325855 | 0.000215725302416793 | 0.000239876691119851 | 0.000262442866394062 | 0.000243166564798555 |
| pos\_973 | 2'-O-Methylglabridin | 1.06652071254627e-05 | 1.35186307135579e-05 | 5.71386924104013e-06 | 9.89058117937375e-06 | 1.13011518710414e-05 | 1.30231434645198e-05 | 1.38210478304703e-05 | 8.07829066283023e-06 | 1.0364371652273e-05 | 1.13585458971561e-05 | 1.16747366824701e-05 | 7.52408666716328e-06 |
| pos\_974 | 3,3'-Dihydroxy-4',5,7-trimethoxyflavan | 3.35338705041458e-05 | 5.56781400207411e-05 | 4.05796151964594e-05 | 4.57001712259488e-05 | 4.01677293037858e-05 | 3.99014438631595e-05 | 5.70736813362866e-05 | 3.64236253806632e-05 | 7.17903075023123e-05 | 4.42442247412391e-05 | 4.97856118434298e-05 | 2.99738548510118e-05 |
| pos\_975 | Carnosine | 4.98945576258402e-05 | 6.27752589707045e-05 | 6.46451619029511e-05 | 7.10638499707988e-05 | 5.97659958124646e-05 | 5.82210780047009e-05 | 4.97151033163376e-05 | 4.72515736013136e-05 | 8.41958678500804e-05 | 7.58252048745615e-05 | 5.90976703379154e-05 | 5.68797931333117e-05 |
| pos\_976 | Nonoxynol-9 | 0.0106995147871808 | 0.00411522283135404 | 0.0038934144835909 | 0.00351256978687009 | 0.00496229662000229 | 0.00510709899826235 | 0.00530286165686184 | 0.00455382766341752 | 0.0045334128521402 | 0.00431981308668291 | 0.00468392894350906 | 0.00457688137612614 |
| pos\_977 | 2-Phenylethyl 2-furancarboxylate | 4.07068177633012e-05 | 7.07188152909303e-05 | 3.29018835811878e-05 | 4.24565526803143e-05 | 5.21962623618468e-05 | 4.35826970142218e-05 | 4.44900215025511e-05 | 4.02032207893992e-05 | 5.51955538946731e-05 | 4.75796980703165e-05 | 4.54580802079227e-05 | 4.02382579986332e-05 |
| pos\_978 | LysoPE(0:0/20:2(11Z,14Z)) | 2.5123042005791e-05 | 2.77490192246814e-05 | 2.72962343153592e-05 | 2.30380375999052e-05 | 2.33521433649718e-05 | 2.54475949310112e-05 | 2.19233382555801e-05 | 2.45315644989383e-05 | 2.78812502567022e-05 | 2.54607495364634e-05 | 2.42795587367613e-05 | 2.33691316900172e-05 |
| pos\_979 | Piperundecalidine | 3.25741807967988e-05 | 3.61698704939995e-05 | 7.6235431930791e-05 | 2.53516795954673e-05 | 6.16864709398326e-05 | 3.69437341778515e-05 | 1.78873448794949e-05 | 3.38829486612197e-05 | 2.2540808849378e-05 | 0.000103023115257234 | 3.853315989676e-05 | 6.38941714644171e-05 |
| pos\_98 | 2-(5,8-Tetradecadienyl)cyclobutanone | 0.000299369885033566 | 0.000312450988224534 | 0.000257380898583894 | 0.000479608713765577 | 0.00115910302535461 | 0.000355318061079944 | 0.00557356396273543 | 0.00261068465265924 | 0.000234661480110084 | 0.00198646760394563 | 0.0010520017115658 | 0.00159446404893244 |
| pos\_980 | N-Carbamoylputrescine | 5.5047413245507e-05 | 5.62296857313714e-05 | 5.66584542675308e-05 | 4.40094156066114e-05 | 6.08199622032845e-05 | 5.67056991091036e-05 | 4.28820078800525e-05 | 4.76058774421087e-05 | 3.54590823699089e-05 | 0.000106396713558894 | 5.73039292463835e-05 | 6.00950918907727e-05 |
| pos\_981 | Dopaxanthin quinone | 3.27146964389559e-06 | 4.74409556419454e-06 | 4.47291235538066e-06 | 3.76663973698608e-06 | 2.73504257444639e-06 | 4.18150463461712e-06 | 3.64199928166939e-06 | 3.56236944261863e-06 | 4.57457999999587e-06 | 4.39654680089135e-06 | 3.78413423529054e-06 | 3.58235784214712e-06 |
| pos\_982 | Histidinyl-Lysine | 1.26349867130569e-05 | 1.96515165741143e-05 | 8.41258029586985e-06 | 1.27084165002523e-05 | 6.74721339738429e-06 | 1.10034146694106e-05 | 1.78345294207343e-05 | 1.31872326504512e-05 | 7.66741328909261e-06 | 1.08962812789664e-05 | 1.3487469962031e-05 | 8.83441975880705e-06 |
| pos\_983 | 8-Deoxy-11-hydroxy-13-chlorogrosheimin | 2.40114688960187e-05 | 3.14422938381088e-05 | 2.9925391480731e-05 | 2.51138531336483e-05 | 2.30525138094472e-05 | 2.43895745824799e-05 | 2.36139378702409e-05 | 2.57904232184679e-05 | 2.91827026640743e-05 | 2.4906933827363e-05 | 2.51624350961911e-05 | 2.45710889151986e-05 |
| pos\_986 | 2-(5-Methyl-2-furanyl)-3-piperidinol | 4.04053844181967e-05 | 4.2999933502207e-05 | 4.16779428243298e-05 | 4.160773965779e-05 | 3.62336621790334e-05 | 3.67478868261379e-05 | 3.72751506474551e-05 | 4.18820872619498e-05 | 3.25871312670959e-05 | 4.0343220424184e-05 | 3.67831261717528e-05 | 6.51089686672152e-05 |
| pos\_987 | Propofol glucuronide | 0.000147914916061451 | 0.000214243139990332 | 0.000117838450826822 | 0.000144900107172948 | 0.000144662880931239 | 0.000147507113923144 | 0.000165093986158406 | 0.000139006873223241 | 0.000156808991020304 | 0.000191246965927554 | 0.000169523376806358 | 0.000127239817264249 |
| pos\_988 | 4-Hydroxy-5-methyl-3(2H)-thiophenone | 6.30558694777471e-06 | 7.90502348592751e-06 | 9.88349828224998e-06 | 6.79695573201972e-06 | 5.25389751769173e-06 | 6.58081226638962e-06 | 6.37709497854613e-06 | 8.02856859242262e-06 | 8.84914772284032e-06 | 5.51662109539408e-06 | 8.1809130762295e-06 | 7.59789681664675e-06 |
| pos\_989 | Benzyl 2,6-dihydroxybenzoate 2-glucoside | 1.15540709083112e-05 | 1.72348596413917e-05 | 2.37323318326776e-05 | 1.60557441917882e-05 | 9.29736592217916e-06 | 1.68469171452889e-05 | 1.61169128254502e-05 | 1.49654237648919e-05 | 1.78080521540163e-05 | 1.6275945835555e-05 | 1.7340248502863e-05 | 1.44089312316889e-05 |
| pos\_99 | 1,2,3,4,tetrahydro-1,5,7-trimethylnapthalene | 7.08118228135781e-05 | 7.09616135310128e-05 | 0.000119517661749641 | 0.000112957176361841 | 0.000119591673964877 | 0.000107153446238781 | 9.57634228727018e-05 | 9.6211008646581e-05 | 0.000116568060679118 | 0.000104128301467945 | 0.000105502259387462 | 0.000109071281799653 |
| pos\_990 | Afzelechin 7-apioside | 1.09512752503749e-05 | 1.52541125220512e-05 | 2.54986121290955e-05 | 1.37433312008812e-05 | 9.98582414824242e-06 | 1.46497249678033e-05 | 1.45169302246077e-05 | 1.51471046176083e-05 | 1.59251649458174e-05 | 1.53337567489313e-05 | 1.6671761889035e-05 | 1.25435984805839e-05 |
| pos\_992 | 11,12-Dimethoxydihydrokawain | 0.000156899868608402 | 0.000177219038608602 | 8.76804038783517e-05 | 0.000127079802969823 | 0.000195919356654929 | 0.000153438661839047 | 0.000255875143880939 | 0.000187193602952321 | 0.000123294176181926 | 0.000154771620030326 | 0.00017580853271695 | 0.000133936070378751 |
| pos\_994 | Ponasteroside A | 0.00014558870830708 | 9.5432777862458e-05 | 8.27211488776346e-05 | 8.61379980876646e-05 | 9.52810843247133e-05 | 9.62811618181878e-05 | 9.98231726787498e-05 | 0.000104637770835296 | 8.43624285608598e-05 | 9.34854464435467e-05 | 9.18014148994608e-05 | 0.000107551182031307 |
| pos\_996 | Capsianoside II | 2.51709961583501e-05 | 2.98400084409965e-05 | 3.67868619204118e-05 | 3.27219931280479e-05 | 2.65858342634618e-05 | 3.97255469237579e-05 | 3.58800184221811e-05 | 4.66174089580093e-05 | 3.58159957218388e-05 | 4.20850143521857e-05 | 3.69918935214519e-05 | 4.83479952436867e-05 |
| pos\_997 | 5,6-Dihydroxyprostaglandin F1a | 0.000297980775912837 | 0.000581294829149363 | 0.00037926842867303 | 0.00030260773367841 | 0.000394788926513476 | 0.000324592520730037 | 0.00032685893032077 | 0.00035457314144889 | 0.000396084422452812 | 0.00026236711293751 | 0.00027142437529564 | 0.000355774701952169 |
| pos\_998 | Isosyringinoside | 1.85893989422655e-05 | 3.67977295304872e-05 | 1.33525508708903e-05 | 2.31740579623342e-05 | 2.5147901782226e-05 | 2.06013245864187e-05 | 2.94751466298554e-05 | 2.52656150397695e-05 | 2.48582315397789e-05 | 1.71676255818012e-05 | 2.33879092712937e-05 | 1.82295070873306e-05 |
| pos\_999 | Tryptophyl-Proline | 1.7051481967083e-05 | 2.26402986209478e-05 | 1.77948906860027e-05 | 2.28325523596713e-05 | 2.11080336150975e-05 | 1.70180260093283e-05 | 1.52232924392004e-05 | 1.59006614074095e-05 | 2.021434126724e-05 | 1.79216353403867e-05 | 1.70914038408556e-05 | 2.17563758538368e-05 |
